# Development and characterization of Hanwoo oviduct epithelial cell line for heat stress study

**DOI:** 10.5713/ab.250962

**Published:** 2026-09-01

**Authors:** Ismail Shaleh, Sung Woo Kim, Tae Sub Park, Joonghoon Park

**Affiliations:** 1 Graduate School of International Agriculture Technology, Green Bio Science & Technology, Seoul National University, Pyeongchang, Korea; 2 Department of Biology, Faculty of Mathematics and Natural Science, IPB University, Bogor, Indonesia; 3 Hanwoo Research Institute, National Institute of Animal Science, Rural Development Administration, Pyeongchang, Korea; 4 Department of Agricultural Biotechnology, Research Institute of Agriculture and Life Science, Seoul National University, Seoul, Korea

**Keywords:** Cattle Reproduction, Hanwoo Cattle, Heat Stress, Immortalized Oviduct Epithelial Cells, Proteomics, Transcriptomics

## Abstract

**Objective:**

Heat-induced stress (HS) amid global warming compromises cattle reproductive performance causing economic loss including the vulnerable Korean native beef cattle, Hanwoo. Nevertheless, knowledge of response and effect in cattle oviduct epithelial cells (OEC) under HS remains lacking due to restricted commercial cell line and multi-omics data available. This research attempts to develop immortalized OEC derived from Hanwoo cattle as HS cell culture model and to investigate its HS-specific response by utilizing biomarkers comprising transcriptomic and proteomic analysis.

**Methods:**

Primary OEC was isolated from fresh Hanwoo oviduct and immortalized by using *piggyBac* transposon-mediated SV40T expression system. HS optimization was performed by detection of reactive oxygen species (ROS) and oxidative-endoplasmic reticulum stress biomarkers under varied HS and recovery time (4, 12, and 24 h). Optimized period was applied to generate differentially expressed genes (DEG) and protein (DEP) analyzed using gene ontology (GO) and pathway enrichment.

**Results:**

Immortalized OEC was successfully developed under high level of CDH1 positivity (98.2%) sorting. Increased intracellular ROS was detected at 4 h HS followed by rapid substantial upregulation of HSP70 and BiP protein and delayed upregulation of oxidative stress genes (*SOD1*, *CAT*, *GPX1*) peaked at optimized 24 h HS period. In total there were 510 DEG (fold change≥|2|) and 258 DEP (fold change≥|1.5|) were significantly altered under 24 h HS condition. Furthermore, GO and pathway enrichment analysis of the DEG-DEP list revealed highlighted upregulation process related to oxidative-thermal stress and protein folding. Other altered processes involve immune response, cellular senescence, response to starvation, and extracellular matrix structures as enriched terms suggesting possible hindering effects to OEC reproductive role.

**Conclusion:**

Developed immortalized OEC presents stable cell culture system to evaluate HS response in cattle oviductal tract. Transcriptomic and proteomic data generated in this study are valuable sources for future research regarding targeted molecular approach attenuating detrimental HS effect in cattle reproduction.

## INTRODUCTION

Heat stress (HS) caused by global warming challenges cattle productivity by compromising reproductive performance. Elevated temperatures have been found to disturb mitochondria membrane integrity and accumulating intracellular reactive oxygen species (ROS) leading to metabolic imbalance and apoptosis in cattle reproductive cells [1,2]. The hyperthermic stress at thermal-humidity index bigger than 72 significantly decrease cattle reproductive performance by hindering oocyte maturation and embryo development at early stage [3]. Furthermore, there are evidences that the HS negative effects continue post-HS period impairing calves quality which subsequently decline livestock productivity across multiple generations [4]. Despite this, well-known mitigation approach on the field remains constrained to technical methods like thermal regulator system and assisted reproductive technology [5] whereas specific strategy such as molecular targeting approach to relieve HS detrimental effect to cattle reproduction is currently understudied.

Hanwoo cattle, a native Korean beef cattle breed, has been known to be vulnerable to hyperthermal stress causing economic loss. In 2022, artificial insemination data from National Hanwoo Association of Korean Beef Policy Research Institute presented significant decrease in reproductive efficiency of Hanwoo during and after summer season from August to November [6]. *In vitro* study also revealed that summer heat waves cause mitochondrial dysfunction and ROS overaccumulation in Hanwoo oocytes leading to delayed cytoplasmic maturation and impaired developmental competency of the Hanwoo preimplanted embryo [7]. Moreover, Hanwoo calves born during summer also had remarkable lower birth weight compared to calves born in other seasons [8]. Therefore, Hanwoo cattle are a breed of concern because HS negatively affects their reproductive performance which requires effective protective measures in the future.

Oviduct epithelial cells (OEC) are valuable cells lining the oviduct - reproductive tract connecting ovary and uterus, that provide supporting nutrients and microenvironments for successful fertilization and pre-implanted embryo development [9]. The OEC interact physically and chemically with gametes such as inducing fertile sperm hyperactivation, initiating attachment of ovulated cumulus-oocyte complex (COC), and transporting incoming sperm and early developing embryo via cilia movement, secretory fluid, and extracellular matrix (ECM) functioning [9–12]. In addition, there is evidence that OEC also provide anti-oxidative protection by secreting follicular fluid containing hypotaurine, taurine, and ascorbic acid as well as expressing superoxide dismutase (SOD), glutathione peroxidase (GPX), and catalase (CAT) to maintain ROS exposure to oocyte and embryo [13]. Furthermore, OEC have been found to interact with embryo to stimulate anti-inflammatory response via interferon-tau (IFNT) [14] which is in particular substantial to prevent subfertility due to overacting immune cells in bovine OEC [15]. However, despite its prominent complex mechanism upholding initial phase of reproduction, recently there is limited stable bovine OEC cell line available [16,17]. Consequently, establishment of bovine OEC line could become progression step to further study the response of OEC under global thermal stress.

Hyperthermal stress impairs specific OEC functions reducing bovine reproductive capacity. Elevated temperature at 41°C for 7 days reduces cleavage and significantly diminishes the number of embryos that develop into blastocyst stage when co-cultured with OEC [18]. Under HS, bovine OEC also increases HSP70 expression and decreases OVGP1 secretion, a specific embryotropic glycoprotein in oviduct, compromising OEC support to reproductive process [18]. Some transcriptomic and proteomic studies of bovine OEC under HS also identify altered mRNA expression and oviductal protein composition related to immune response, muscle contraction, gamete protection, endocrine resistance, cellular senescence, notch and TGF-beta signaling, and long non-coding RNA [19,20]. Upregulation of oxidative and endoplasmic reticulum (ER) stress response of other bovine reproductive cells are also reported under thermal stress [21]. Unfortunately, recent comprehensive study that specifically evaluates bovine OEC molecular responses under HS are substantially lacking. Bovine OEC specific response during recovery post-HS also remains understudied.

Therefore, this study aims to develop immortalized OEC derived from Hanwoo (a Korean local breed) to be utilized for HS *in vitro* research and to evaluate its cellular HS-related molecular response by applying transcriptomic and proteomic approaches.

## MATERIALS AND METHODS

Experimental steps of this study were summarized in Figure 1.

### Primary cells, cell line establishment and characterization

Oviducts from female Hanwoo cattle were collected at a local slaughterhouse and transported on ice. Primary OEC was collected from the fresh oviduct tissue by isolating the thin inner layers of the oviductal lumen epithelia around ampulla region. The tissue layer was physically dissociated using fine-tip scissors and enzymatically dissociated with 0.5% trypsin-EDTA. The sample was then incubated at 37°C for 10 minutes with 3–4 pipetting followed by filtration through 70 μm cell strainers. After centrifuging, the cells were resuspended and grown in pre-warmed complete media containing high-glucose DMEM supplemented with 10% FBS and 1% antibiotic–antimycotic. After passage one, the OEC were immortalized by introducing an SV40 large T antigen (SV40T) insert carried on a piggyBac transposon system together with its transposase (Vector Builder) (Figures 1A, 2A). Transfections were performed using Lipofectamine 3000 (Thermo Fisher Scientific) according to the supplier’s protocol. After lipofection, cells were subjected to puromycin selection (4 μg/mL) to obtain stable lines, and SV40T integration was confirmed by polymerase chain reaction (PCR). Cultures were maintained in high-glucose DMEM supplemented with 10% FBS and 1% antibiotic–antimycotic incubated at 37°C in a humidified incubator with 5% CO_2_.

### Immunohistochemistry

Cells at passage 13 were seeded triplicate at 10,000 cells per well in a 48-well plate and allowed to adhere overnight before initiating the immunocytochemistry procedure. After rinsing with DPBS, cultures were fixed in 4% paraformaldehyde for 20 min at room temperature (RT), followed by three 5-min washes in TBST. The samples were then incubated with a blocking solution (5% donkey serum in TBST) for 30 min at RT. Without additional wash step, primary antibody of anti-mouse IgG E-cadherin or CDH1 (13–5700; Thermo Fisher Scientific) was added and incubated for 5 h at 4°C. Afterward, cells were washed in TBST (3×5 min) and incubated with the secondary antibody of goat anti-mouse IgG Alexa Fluor 488 (A11029; Thermo Fisher Scientific) for 1 h at RT in the dark. Following another set of TBST washes (3×5 min), nuclei were counterstained with DAPI (1×) for 10 min at RT in the dark. The cells were then washed 2–3 times with DPBS and prepared for imaging.

### Florescence-associated cell sorting

Cells were detached with 0.5% trypsin–EDTA, pelleted by centrifugation at 700×g for 3 min, and then resuspended thoroughly in 1 mL of 1% bovine serum albumin prepared in DPBS. The primary antibody of anti E-cadherin or CDH1 (13–5700; Thermo Fisher Scientific) was added and incubated for 30 min on ice with gentle mixing every 5 min. After a DPBS rinse, cells were exposed to a secondary antibody of goat anti-mouse IgG Alexa Fluor 488 (A11029; Thermo Fisher Scientific) for another 30 min on ice, again with intermittent agitation. Following a final DPBS wash, the samples were processed for florescence-associated cell sorting (FACS) using a BD FACSAria Cell Sorter (BD Biosciences) according to the manufacturer’s instructions. The CDH positive sorted cell population was then propagated and applied for subsequent HS treatments.

### Heat stress condition and DNA/RNA/protein extraction

Cells were cultured in triplicate 60-mm dishes and were sampled at 60%–80% confluence for all treatment condition including control, HS, and recovery (Supplement 1). HS was applied at 41.5°C, followed by recovery at 37°C, for exposure periods of 4, 12, or 24 h (Figure 1B) in a 5% CO_2_ incubator maintained at approximately 95% relative humidity. Following collection, genomic DNA (gDNA), total RNA, and proteins were isolated using the DNeasy Blood & Tissue Kit (Qiagen), the RNeasy Plus Mini Kit (Qiagen), and RIPA buffer (Thermo Fisher Scientific), respectively, according to the manufacturers’ guidelines. Protease and phosphatase inhibitors were added to the lysis buffer prior to extraction. DNA and RNA yield and purity were assessed with a nanodrop spectrophotometer, while protein concentrations were measured using the Pierce BCA Protein Assay Kit (Thermo Fisher Scientific) following the supplier’s instructions. The total RNA and protein lysates were pooled from three biological replicates for subsequent transcriptomic and proteomic analysis.

### Total reactive oxygen species detection

Approximately 10,000 cells per well were plated into 48-well dishes and assigned to either the control or HS group. On the following day, HS samples were exposed to 41.5°C for 4 h in a humidified incubator with 5% CO_2_. Intracellular ROS levels were assessed by loading the cells with 10 μM chloromethyl-2′,7′-dichlorodihydrofluorescein diacetate (CM-H_2_-DCFDA; C6827; Thermo Fisher Scientific) prepared in DPBS, followed by a 5-min incubation at 37°C. Without a wash step, cells were immediately subjected to fluorescence imaging.

### Quantitative reverse-transcription polymerase chain reaction and genomic polymerase chain reaction

Complementary DNA (cDNA) was synthesized from 1 μg of total RNA using the Superscript III First-Strand Synthesis System (Thermo Fisher Scientific) according to the manufacturer’s instructions. Quantitative reverse-transcription polymerase chain reaction (qRT-PCR) reactions (25 μL total volume) contained 2 μL cDNA, 2.5 μL PCR buffer, 1 μL of a 2.5 mM dNTP mix, 1 μL SYBR dye, 1 U Taq DNA polymerase, and 10 pmol each of forward and reverse primers (all primer sequences are listed in Supplement 2). Amplification was performed on a CFX Duet Real-Time PCR System (Bio-Rad, Hercules, CA, USA) with the following program: 95°C for 3 min, then 40 cycles of 95°C for 30 s, 60°C for 30 s, and 72°C for 30 s. Agarose gel electrophoresis was conducted to confirm specificity of the primers showing single specific bands at targeted amplicon size (Supplement 3). Gene expression levels were normalized to GAPDH and quantified using the 2^−ΔΔCt^ method. The antioxidant genes assessed were *superoxide dismutase 1* (*SOD1*), *catalase* (*CAT*), and *glutathione peroxidase 1* (*GPX1*) (Figure 1A). Confirmatory mRNA expressions based on microarray data were also performed on selected genes i.e., BAG3, HSPB1, FOXO3, and CHAC1. Genomic PCR of SV40T insertion verification was conducted under the same protocol above with replacing SYBR with the same volume of DNAse/RNAse-free water by using T100 Thermal Cycler (Bio-Rad). Electrophoresis was performed under 1.2% agarose, 120V for 23 min (Figure 2B).

### Western blot

Protein extracts were diluted with DPBS to equal concentrations, combined with Laemmli buffer, and denatured at 95°C for 5 min. Following a brief centrifugation (20,000×g for 1 min), the samples were loaded onto 10% SDS–PAGE gels and electrophoresed at 50 V for 5 min followed by 100 V for 90 min. Proteins were then transferred onto 0.45 μm PVDF membranes using a Mini Trans-Blot Cell (Bio-Rad) at 100 V for 3 h. Membranes were blocked and incubated 24–48 h at 4°C with primary rabbit anti-HSP70 (10995-1-AP; Proteintech), rabbit anti-BiP/GRP78 (11587-1-AP; Proteintech), rabbit anti-BAG3 (10599-1-AP; Proteintech), rabbit anti-COL1A1 (AB34710; Abcam), and mouse anti-ACTB (SC-47778; Santa Cruz Biotechnology). After 5×5-min washes in TBST, HRP-conjugated secondary antibodies (Goat anti-mouse IgG AS003, goat anti-rabbit AS014; ABclonal) were applied for 1 h at RT, and immunoreactive bands were detected using a luminol/hydrogen peroxide chemiluminescent substrate.

### Microarray

Three biological replicates of RNA were pooled into one tube prior microarray analysis. RNA quality was assessed using an Agilent Bioanalyzer, yielding RNA integrity numbers (RIN) between 8.4 and 9.3 and A260/280 ratios≥2.0, suitable for processing on the GeneChip (Bovine) Gene 1.0 ST Array (Affymetrix). cDNA synthesis was carried out with the GeneChip WT Amplification Kit (Affymetrix) following the manufacturer’s instructions. The resulting sense cDNA was subsequently fragmented and biotin-labeled using terminal deoxynucleotidyl transferase and the GeneChip WT Terminal Labeling Kit (Affymetrix). Approximately 5.5 μg of the labeled target was then hybridized to the Affymetrix GeneChip Array at 45°C for 16 h. After hybridization, arrays were washed and stained on the GeneChip Fluidics Station 450 and scanned using the GCS3000 Scanner (Affymetrix). Probe-level intensity values and CEL files were generated with the Affymetrix GeneChip Command Console (AGCC) software. Normalized fold-change gene expression results are provided in Supplement 4.

### Proteomic liquid chromatography–mass spectrometry

Three biological replicates of protein lysate were pooled into one tube prior protein liquid chromatography–mass spectrometry (LC/MS) analysis. A total of 100 μg protein was processed using S-Trap mini columns (Protifi), followed by TMT10 labeling (Thermo Fisher Scientific) and fractionation via reverse-phase liquid chromatography (Agilent Technologies). Peptide samples were examined on a Q Exactive HF-X mass spectrometer coupled to an Ultimate 3000 RSLCnano system (Thermo Fisher Scientific). Full MS spectra were collected from m/z 400–2,000 at a resolution of 60,000 in a top 10 data-dependent acquisition workflow. Fragmentation was performed by HCD at a normalized collision energy of 32, and MS/MS spectra were acquired at a resolution of 45,000. Data analysis was conducted using Proteome Discoverer 3.0 (Thermo Fisher Scientific) with CHIMERYS searching against the UniProt database, requiring at least two unique peptides and a 1% FDR threshold. Normalized log_2_ fold-change values are provided in Supplement 5.

### Data analysis

Protein band intensities were quantified using ImageJ software. Data processing and visualization were carried out in GraphPad Prism 8 and R version 4.5.1. Statistical comparisons (p<0.05) were performed using one-way ANOVA, and groupings were assigned automatically using the “multicompView” package. Protein–protein interaction networks were constructed with STRING (https://string-db.org/) version 12.0 under high-confidence score at 0.07 and other default parameters. Gene ontology (GO) enrichment for both transcriptomic and proteomic datasets was conducted using the R package “clusterProfiler,” applying significance thresholds of p<0.05 and q<0.2. Pathway enrichment (KEGG and Reactome) was carried out in Metascape (https://metascape.org/) version v3.5.20250701 [22] through batch analyses, using p<0.01, a minimum of three genes per term, and an enrichment factor greater than 1.5. Heatmaps were generated with the “ComplexHeatmap” R package, and pathway–gene networks were visualized using “igraph.” All omics analyses were performed using human reference databases.

## RESULTS

### Development and characterization of Hanwoo oviduct epithelial cell line

Immortalized OEC have been developed by transfecting SV40T antigen by using *piggybac* transposon expression vector (Figure 2A). PCR result of gDNA of the OEC line exhibited detection of SV40T specific 351 bp amplicon which was not detected in intact cattle gDNA (Figure 2B, Supplement 6). cDNA from total mRNA also showed specific SV40T amplicon confirming transcription of SV40T antigen gene in the OEC line (Figure 2B, Supplement 6). This result suggests successful transfection of SV40T gene into the cells during immortalization. Cadherin-1 (CDH1) or epithelial cadherin is specific molecular marker for epithelial cells involved in cell-cell adhesion during fertilization in bovine OEC [10]. The CDH1 positive cells were specifically selected by FACS sorting compared to background florescence signal (Supplement 7). Immunofluorescence staining in the immortalized OEC presented prominent florescence signal of cells bound to specific anti-CDH1 antibody in the sorted cells (Figure 2C). Together with cell line stabilization under puromycin selection during early passages, the sorting process increased the homogeneity of the cell population and the cells were actively proliferating until passage 23 (Figure 2D) prior HS optimization in the following treatment. In addition, the OEC characterization using transcriptomic and proteomic data also presented major expressed genes, specific protein biomarkers, and similar functional enrichment to the original bovine oviduct epithelium (Supplement 8).

### Oxidative stress evaluation

Elevated temperature at 41.5°C for 4 h has increased total intracellular ROS level in the OEC line represented by intensified DCF fluorescence signal compared to control group (Figure 3A). Nevertheless, none of the three oxidative stress marker genes (*SOD1*, *CAT*, *GPX1*) expression were altered during 4 h HS and 4 h recovery period (Figure 3B–3D). Upregulated anti-oxidative response was significantly detected at 12 h HS period (p<0.05) for all the gene markers, i.e., *SOD1* expression at 1.40±0.07-fold increase, *CAT* expression at 1.50±0.06-fold increase, and *GPX1* expression at 1.49±0.02-fold increase relative to control treatment (Figure 3B–3D; mean±SEM, p<0.05). During 12 h recovery, *SOD1* and *CAT* mRNA level returned to normal conditions (Figure 3B, 3C), but *GPX1* mRNA upregulation was prolonged at 1.59±0.04-fold increase relative to control (Figure 3D). The antioxidant gene response was amplified and peaked at 24 h HS. Hyperthermic condition at 24 h elevated *SOD1*, *CAT*, and *GPX1* transcription level to 1.71±0.03-fold, 1.78±0.06-fold, and 2.10±0.09-fold increase against the control level, respectively (Figure 3B–3D). After 24 h recovery period, *SOD1* and *CAT* expression were reverted to baseline (Figure 3B, 3C); however, the *GPX1* displayed decreasing expression to 1.46±0.07-relative fold change while it was still significantly higher in comparison to the control (Figure 3D). Collectively, accumulated cellular ROS in OEC did not immediately trigger upregulation of the antioxidant genes at 4 h HS whereas HS starting from 12 h and 24 h period induced *SOD1*, *CAT*, and *GPX1* upregulation with peaked increased expression at 24 h HS. All the oxidative stress marker genes presented recovering trend when returned to thermoneutral condition, except *GPX1* during 12 h recovery. These results confirmed direct antioxidant gene upregulation in the OEC line to maintain cellular oxidative balance under HS condition.

### Heat-shock and endoplasmic reticulum stress evaluation

Unlike oxidative stress gene response, significant upregulation of the common heat-shock protein, HSP70, in OEC was detected in all HS periods (4 h, 12 h, and 24 h) and remain significantly high during all recovery periods compared to control group of this study (Figure 3E, 3F, Supplement 9). During 4 h HS period, HSP70 protein level increased up to 10.85± 0.83-fold higher than control treatment (p<0.05) and maintained this expression level during 4 h recovery (Figure 3F). Furthermore, a significantly higher HSP70 protein expression was also exhibited during 12 h HS reaching relative 17.24± 0.52-fold increase. It showed declining trend during 12 h recovery while persist at 14.10±0.26-fold change upregulation (Figure 3F). Under 24 h HS condition, OEC increased HSP70 protein expression to relative fold change at 6.24±0.11 and continue this expression level during 24 h recovery (Figure 3F). Similarly, the ER-specific chaperone, BiP protein, revealed upregulation since 4 h HS treatment at 2.46±0.08-fold increase relative to control (Figure 3E, 3G, Supplement 9). Interestingly, the increasing trend continued during 4 h recovery periods reaching significant 4.31±0.17-fold change. HS condition for 12 h and 24 h period elevated BiP protein expression of OEC up to 2.19±0.21-fold and 2.56±0.07-fold change, respectively, and the protein level remained steadily high under 12 h and 24 h recovery (Figure 3G). Overall, these heat-shock and ER stress protein upregulation represented OEC protective response to repair misfolded proteins, not only during hyperthermal stress periods but also during recovery periods under normal temperature. Finally, along with the oxidative stress gene markers expression results described above, we considered 24 h as optimal HS period for the OEC line and adopt this treatment to sample transcriptomic and proteomic data.

### Differentially expressed genes, proteins, and STRING analysis

Under 24 h HS treatment, there were 510 differentially expressed genes (DEGs) with 276 upregulated DEGs and 234 downregulated DEGs compared to control group (HS/CON) from the total 1079 DEGs presented under log_2_ fold-change threshold≥|2| (Figure 4A, left Venn diagram). Among 218 overlapping DEGs between HS/CON and recovery group (REC/CON) there were 121 upregulated genes and 97 downregulated genes. Additionally, fewer differentially expressed proteins (DEPs) were altered under 24 h HS conditions with total 258 altered DEPs (fold-change threshold≥|1.5|) comprising 88 upregulated DEPs and 170 downregulated DEPs in comparison to control (HS/CON) from total 479 DEPs of all groups (Figure 4A, right Venn diagram). Among these, 30 DEPs and 92 DEPs remained upregulated and downregulated, respectively, during recovery periods as shown in overlapping DEPs of HS/CON and REC/CON group. These overlapping genes represent gene sets that displayed prolonged alteration post-HS in OEC.

Alternatively, corresponds to Venn diagram of Figure 4A, heat maps of DEG and DEP demonstrate dynamic gene and protein expression alteration during HS and recovery period in the OEC line (Figure 4B). At mRNA level, most of the DEGs clusters upregulated during HS (HS/CON) continued their increased level during recovery (REC/CON) (Figure 4B, left). Similar patterns appeared in downregulated DEGs. The HS/REC column showed the gene expression trend in which positive number (red color) and negative number (blue color) indicated DEGs with declining and increasing trend, respectively, during recovery compared to HS group. In the same way, DEPs heat map also showed most genes cluster with either prolonged upregulation or downregulation (Figure 4B, right) with some noticeable recovering genes.

Limited number of DEG-DEP overlapping genes (DEG ∩ DEP) were identified in this study (Figure 4C). Among the total 28 genes concordantly expressed at both mRNA and protein level, 9 genes were identified in HS/CON group (5 upregulated, 4 downregulated) and 19 genes identified in REC/CON group (15 upregulated, 4 downregulated). Remarkably, 15 upregulated DEG-DEPs during recovery compared to control (REC/CON) exhibited significant STRING association (protein-protein interaction enrichment p-value<1.0e-16) and enrichment to GO (BP) and pathways including carboxylic acid metabolic process (FDR = 0.0372), biosynthesis of amino acids (FDR = 0.0065), and amino acids transport across plasma membrane (FDR = 0.0046) (Figure 4D). These identified genes in DEG ∩ DEP list might be particularly important to elucidate OEC concordant molecular response during HS and post-HS periods.

### Validation of mRNA and protein expression level of representative genes

To validate DEG and DEP under pooling samples (n = 1), we performed confirmatory qRT-PCR and WB of representative genes under suitable biological replicates (n = 3) (Figure 4E). Four upregulated DEG i.e. *BAG3*, *HSPB1, FOXO3*, and *CHAC1*, showed significant (p<0.05) increased relative mRNA level under either HS or recovery periods compared to control group coherent with microarray data. *BAG3* and *HSPB1* qRT-PCR results presented higher relative fold changes (~3 to 4-fold increase) compared to DEG scores (~1.3 to 1.9-fold increase). Both show decreased pattern during recovery after HS. The *FOXO3* and *CHAC1* mRNA level were significantly upregulated (p<0.01) during recovery periods based on qRT-PCR. Additionally, coherent pattern of protein alteration between DEP and WB approach were validated in BiP (also known as HSPA5), BAG3, and COL1A1 protein (Figure 4E bottom, HSPA5/BiP WB was redrawn from Figure 3 data). Higher relative fold-changes (~2.5 to 4-fold increase) were detected in HSPA5/BiP and BAG3 based on WB and comparable fold changes of DEP-WB protein level were measured for COL1A1. Discrepancy in magnitudes of expression level in microarray compared to qRT-PCR were common due to differences in sensor sensitivity, signal saturation, and detection ranges among the two approaches [23,24] which also expected between LC/MS and WB-based protein level detections. Finally, based on these results, DEG/DEP alteration resulted from pooled sample analysis were validated which support the reliability of subsequent analysis.

### Gene ontology and pathways enrichment analysis

Gene list of DEG and DEP identified in the OEC line under HS exhibited significant enrichments (p≤0.05, q≤0.2) to various GO terms (BP, MF, and CC) (Supplements 10–13). Among the list, we highlighted GO terms related to protein folding, stress response, immune response, development, and major structural and functional terms addressing possible HS cellular response in the cells (Figure 5, Supplements 10–13). As expected, upregulated DEG and DEP demonstrated increased HS-related responses and localization such as apoptotic signaling, oxidative stress, ER stress, and protein refolding, including HSP binding during HS over control group (Figure 5A, 5B, HS/CON). Interestingly, “response to starvation” was also identified as a top significant GO term enriched from DEG list in both HS and recovery over control level (Figure 5A) which may suggest alteration in cellular energy molecule metabolism. Response to oxidative stress and protein folding also presented significant enrichment in REC/CON group suggesting pro-longed alteration of these functions during recovery (Figure 5A, 5B). In addition, upregulated DEP list also displayed enriched terms related to epidermal development and cellular structure such as cytoskeleton and ECM under recovery relative to control level (Figure 5B, REC/CON). The ECM structure terms were also significantly enriched in both downregulated mRNA and protein level during recovery (REC/CON) and HS group (HS/CON), respectively (Figure 5C, 5D). Several immune related enrichment terms such as cytokine activity, interleukin (IL)-1 response, and inflammation regulation were also detected in upregulated and downregulated DEG (Figure 5A, 5C). Furthermore, hemostasis, chromatin modification process, and survival regulation such as MAPK and ERK functioning were also exhibited in the enriched GOs (Figure 5B–5D). Overall, the GO enrichment results indicated activation of common HS cellular response and alteration of structural and functional response in the OEC line.

Supporting GO enrichment results, pathway enrichment also demonstrated significant terms (logP<2, minimum count = 3, enrichment factor>1.5) to stress and HS responses in both upregulated DEG and DEP during HS and recovery periods (Figure 6A, 6B, Supplements 14–17). This HS-related pathway includes upregulation of common HSP proteins, chaperones, and co-chaperones such as CRYAB and BAG proteins (Figure 6B). Mitochondrial unfolded protein response and glutathione metabolism pathway involving increased expression of *HSPA9* and *GPX3*, respectively, were also presented in REC/CON group suggesting possible delayed HS response (Figure 6A, 6B; DEG). Remarkably, along with the response to starvation pathway (Figure 6A) there were alterations in major nutrients metabolism such as amino acids and lipids in upregulated DEG-DEP (Figure 6A) as well as carbohydrates, lipids, and nucleotides in downregulated DEG-DEP (Figure 6C). Small molecules transport functions including several SLC genes were also altered during or after HS (Figure 6A, 6B). Moreover, ECM related pathways were enriched in downregulated DEG-DEP particularly collagen (COL) proteins related to “degradation of ECM” which might indicate protection against ECM damage under HS (Figure 6C, 6D). Immune related pathways including ILs and cytokine signaling process were also altered based on downregulated DEG-DEP enrichment (Figure 6C). Additionally, downregulated DEG indicated alteration in apoptotic and ovarian steroidogenesis functions with some prominent signaling pathway enriched such as GPCR, FOXO, MAPK, and TGF-beta signaling (Figure 6C). The given pathway-gene networks presented resolution at individual gene or protein level of highlighted pathways providing more details elaboration of important processes and genes altered during HS and recovery (Figure 6B, 6D). Collectively, the pathway enrichment analysis demonstrated findings on altered functions regarding HSP and glutathione response, nutritional metabolism, molecule transport, ECM structure, and immune-related signaling in the immortalized cattle OEC.

## DISCUSSION

The importance of OEC roles in supporting fertilization and early embryo development has gained scientific attention in evaluating negative effect of HS to the OEC functioning. Notably, most in vitro HS studies of cattle OEC were performed by using primary cells [18,19]. Apparently, it provides direct physiological relevance yet triggers other challenges such as sample batch variability, limited growth, and laborious-resourceful protocols intricating elucidation of specific HS response in cattle OEC. While reports of using spontaneous and immortalized cattle OEC lines do exist [16,17]; unfortunately, there is no commercialized cattle OEC recently available. This study establishes specific immortalized CDH1 (+) OEC from Hanwoo cattle by conducting gene-editing techniques of piggyBac transposon mediated insertion of SV40T gene to investigate HS response in OEC in more reproducible ways (Figure 2). Complementing this single antibody-based characterization, the immortalized OEC characterization using multiomic data also displayed expression of epithelial and or bovine OEC-specific functional biomarkers at mRNA and protein level (Supplement 8). This result further confirms the specificity of our established OEC cell line comparable to authentic bovine OEC at normal physiological condition. Overall, expression of specific molecular HS response of the cell line has been confirmed such as upregulation of oxidative stress, chaperone, and ER-stress markers (Figures 3, 4) supported by transcriptomic and proteomic enrichment analysis (Figures 5, 6) suggesting its suitability for HS-related study.

Hyperthermic conditions caused elevated total intracellular ROS at 4 h HS and upregulated gene expression of oxidative stress markers i.e. SOD1, CAT, and GPX1, starting at 12 h HS and peaked at 24 h HS period (Figure 3A–3D). This response indicates direct adaptation to balance cellular oxidative level of the cattle OEC, coherent with previous reports of cattle reproductive cells under HS [1,2,25]. Under overaccumulation of intracellular ROS, the stressed cells upregulated SOD1 enzyme expression to transform primary ROS i.e. superoxide radicals (O_2_^•−^) into hydrogen peroxide (H_2_O_2_) which is subsequently metabolized into water through both direct reaction by CAT and indirect glutathione-coupled reaction by GPX1 enzymes [26–28]. An existing *in vitro* study found that thermal stress increased ROS and H_2_O_2_ level in mouse oviduct causing significant 2-cell embryo growth arrest and loss of developmental competence [29]. Interestingly, this study revealed general returning patterns of the oxidative stress genes during recovery periods post-HS (except GPX1 at 12 h treatment, Figure 3B–3D) suggesting restoring oxidative stress response and maintained level of intracellular ROS up to 24 h HS period. Indeed, this oxidative balancing mechanism is crucial in OEC supportive functions preventing cellular dysfunction and death caused by over accumulated ROS [1,2]. However, it is worth noting that in field conditions, cattle may be exposed to more than 24 h pro-longed HS, such as during summer season. HS carry-over negative effect in cattle progeny has also been previously reported [4].

Both heat shock and ER-stress protein markers investigated in this study, HSP70 and BiP/GRP78, are normally expressed and secreted in bovine OEC to maintain stress protection and gametes interaction [18,30]. Under hyperthermic environment, HSP70 protein expression is significantly upregulated to 10, 17, and 6-fold higher than normal conditions during 4 h, 12 h, and 24 h HS period, respectively (Figure 3E, 3F). These are expected results because previous study has detected *HSP70* mRNA and protein upregulated expression up to 2-fold increase in bovine OEC under 7-days HS compared to control [18]. These data also suggest behavior of HSP70 expression that markedly upregulated during early period of HS while later maintaining its significant higher protein expression relative to control during a longer HS period (up to 7 days [18]). Definitely, the HSP70 protein is a versatile chaperone which is rapidly induced under thermal shock that guide proper protein folding and prevent aggregation of misfolded proteins to mitigate cellular damage and apoptosis [31]. Interestingly, the HSP70 activation response under HS was retained even after recovery time (Figure 3F) indicating pro-longed survival and supportive response in the immortalized cattle OEC.

Upregulation of the inducible ER-stress marker, BiP protein (also known as GRP78 or HSPA5), in cattle OEC under HS has firstly been reported in this study (Figure 3E, 3G). This is in line with other cattle reproductive cell (granulosa) response under HS [21]. Under thermoneutral ovulatory period, BiP is one major protein identified in bovine oviductal fluids [30,32]. Heat shock treatment increases the BiP protein expression to about 2-fold higher than normal condition in 4 h, 12 h, and 24 h HS period with remarkable 4-fold increase during 4 h recovery period (Figure 3G). In this context, this prominent BiP upregulation suggest two reaction i.e. survival functions of OEC to prevent aggregation and to repair excessive misfolded protein in ER lumen due to HS [33] and sustaining response to pertain BiP production involved in Ca^2+^-dependent sperm capacitation and sperm-zona pellucida binding modulation [34], which is a key process for successful fertilization. BiP activation also protect cells from damaging ROS effect, maintain normal ER functioning in supporting environment for preimplantation embryo [33]. Unfortunately, even though evidence shows cattle OEC is survived under HS, there was delayed detrimental effect of thermal-stressed OEC to embryo development [18]. Therefore, the increased chaperone and ER-stress markers might be a concerning sign of possible disruption on fertilization and pre-implanted embryo growth occurred in cattle oviductal lumen, not only during HS but also post-HS period [4]. Taken together, the immortalized cattle OEC in this study highlight direct protective molecular responses by activating oxidative stress genes, HSP70, and BiP proteins to balance OEC viability, ROS-protein homeostasis, and reproductive functioning during and after elevated temperature stress.

Under 24 h HS period, DEG-DEP list highlighted enriched terms that attenuate thermal stress and possibly hinder the cattle OEC reproductive physiology. In general, upregulated transcriptomic and proteomic enrichment analysis shows consistent highlighted GO terms and pathways related to response to oxidative stress, ER-stress, and protein refolding (Figures 5A, 5B, 6A, 6C). These results suggest synchronized process involving HSPs upregulation in bovine OEC to alleviate cellular damage under HS as previously known [20]. This response persists during recovery time (Figure 6A) indicating a pro-longed alteration. Notably, similar term of “cellular senescence” from upregulated DEP in this study (Figure 6A) is coherent with transcriptomic analysis in bovine oviduct organoids under HS [20] which might indicate a possible hindering effect in OEC reproductive function [35]. Intriguingly, we also identified top enriched term on “response to starvation” and some alteration in nutritional metabolism and transport including carbohydrates, amino acids, and lipids (Figures 4C, 5A, 5C, 6). This finding might be concerning because mammalian OEC secretes oviductal fluid containing various nutritional and physiological molecules and proteins that facilitate gametes/embryo survival and complex interactions including bicarbonate and potassium ions, glucose, pyruvate, lactate, amino acids, albumin, glycoproteins, and immunoglobulin G [13,36]. Cillia movement of OEC that plays important role in fertilization and COC-embryo migration [12] also requires high supply of energy sources. Therefore, upregulation of nutritional metabolic response might be a compensatory mechanism to sustain OEC normal functions, survival, and energetic requirement under HS. Additionally, significant enrichment to immune-related terms and pathways, particularly the ILs and cytokine signaling of downregulated DEG (Figures 5D, 6B) in this study are in agreement with previous omics results [19,20]. However, further study is required to specifically evaluate alteration of immune response of cattle OEC under HS, especially in elucidating the essential anti-inflammatory of OEC-embryo interaction in oviductal lumen [14]. Of note, our findings also indicate evidence of pro-longed downregulated immune function post-HS (Figure 6B). Furthermore, function alteration in ECM organization (i.e. downregulation of ECM degradation including COL1A1 reduced protein level) during HS and recovery (Figures 4E, 5C, 5D, 6B) and major hormonal-intracellular signaling pathways (Figure 6A, 6B) might represent perturbation in OEC structure, direct communication, and cellular interaction that support cattle sperm, COC, and pre-implanted embryo [11,12]. Collectively, the immortalized cattle OEC signifies prevalent DEG-DEP enriched HS responses and imply potential compromising effect of HS to OEC reproductive performance supported by previous findings.

We acknowledge that the results obtained from SV40T-immortalized OEC in this study should be interpreted carefully. Cellular HSP activation under HS often associated with tumor generation and p53 pathway inhibition [37] but SV40 large T-antigen is known to interact directly with p53 transcription factor reducing its activity [38]. Hence, the cattle OEC cell line here is not suitable for studying this specific modulation. Furthermore, we also concede that pooled sampling of mRNA and protein of this study can potentially cause biased interpretation on the multiomic-based analysis due to limited statistical derivation. To address this issue, additional confirmatory analysis was conducted and alteration of representation genes and proteins under HS and recovery were confirmed (Figure 4E). Therefore, despite these constraints, our immortalized cattle OEC exhibits typical HS-induced response in agreement with prior research. Consequently, the developed cattle OEC line in this study is acceptable and appropriate to be utilized for HS-related study and OEC biology in general.

Beyond that, this research provides valuable transcriptomic and proteomic data that enhance our understanding of cattle OEC cellular mechanisms exposed to heat shock conditions, which are currently have limited data available [19,20]. Not only physical and environmental intervention, future studies should also be focused on identifying molecular targets driving the impairment of cattle reproductive cells functioning [39] which would lead to more comprehensive and effective strategy to mitigate HS negative effect in cattle reproduction. Ultimately, the development of immortalized cattle OEC in this study offers a meaningful *in vitro* model for studying the impact of HS on bovine reproduction providing enhanced reproducibility under optimized experimental systems.

## CONCLUSION

Heat shock conditions trigger intracellular ROS accumulation and interfere with protein folding of the cattle OEC impairing its supportive function for sperm reservoir, gametes transport and interaction, and pre-implanted embryo development. The cattle OEC response the hyperthermic stress by upregulating antioxidant genes (*SOD1*, *CAT*, and *GPX1*) and molecular chaperone protein in cytoplasm (HSP70) and ER (BiP) to alleviate the harmful effects of ROS and sustain protein homeostasis for normal cellular metabolism. GO and pathway enrichment analysis at transcriptomic and proteomic level also exhibits increased function in oxidative and heat-induced stress response in the cattle OEC during and after HS. Additionally, DEG and DEP list from pooled mRNA and proteins displayed significant enriched terms and pathways related to immune response, cellular senescence, response to starvation, and ECM structures which altogether potentially compromising OEC supporting capacity and overall cattle reproduction performance.

Finally, recent development of immortalized cattle OEC as HS *in vitro* model system enhances our knowledge of response and potential cellular process alteration in OEC under elevated temperature amid global warming. The multi-omics data provided in this study also gives substantial contribution to OEC physiological research during and post-HS which can be applied for future research including elaboration of molecular driving targets for mitigating negative HS effects on livestock production.

## Figures and Tables

**Figure 1:**
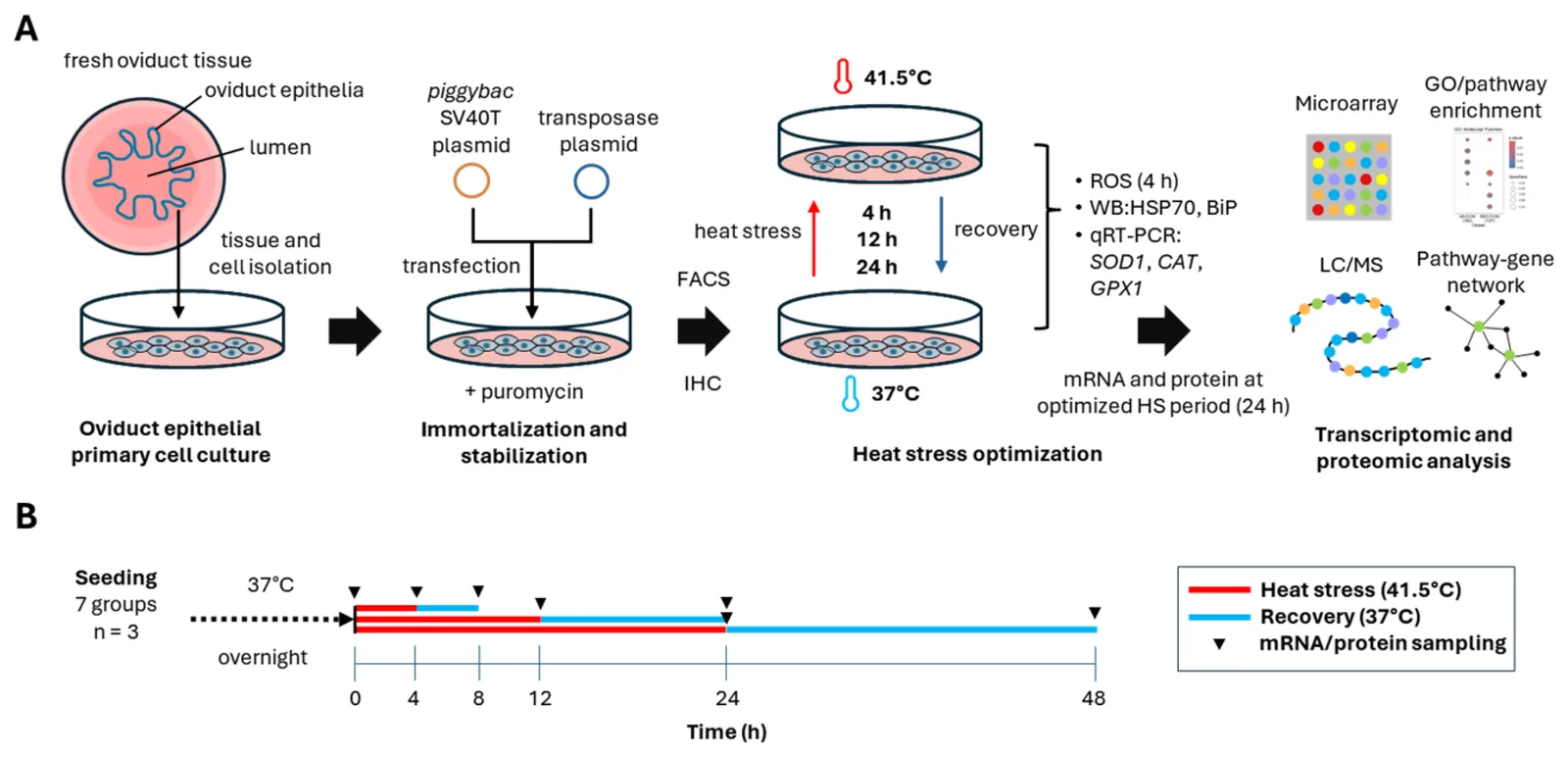
Schematic figure of (A) development of immortalized cattle oviduct epithelial cells, heat stress optimization, and multi-omics analysis in this study and (B) sampling time of the heat stress and recovery under three different periods (4 h, 12 h, and 24 h). FACS, florescence-associated cell sorting; IHC, immunohistochemistry; ROS, reactive oxygen species; WB, western blot; HSP70, heat shock protein 70; BiP, binding immunoglobulin protein; qRT-PCR, quantitative reverse-transcription polymerase chain reaction; *SOD1*, *superoxide dismutase 1*; *CAT*, *catalase*; *GPX1*, *glutathione peroxidase 1*; LC/MS, liquid chromatography–mass spectrometry; GO, gene ontology.

**Figure 2:**
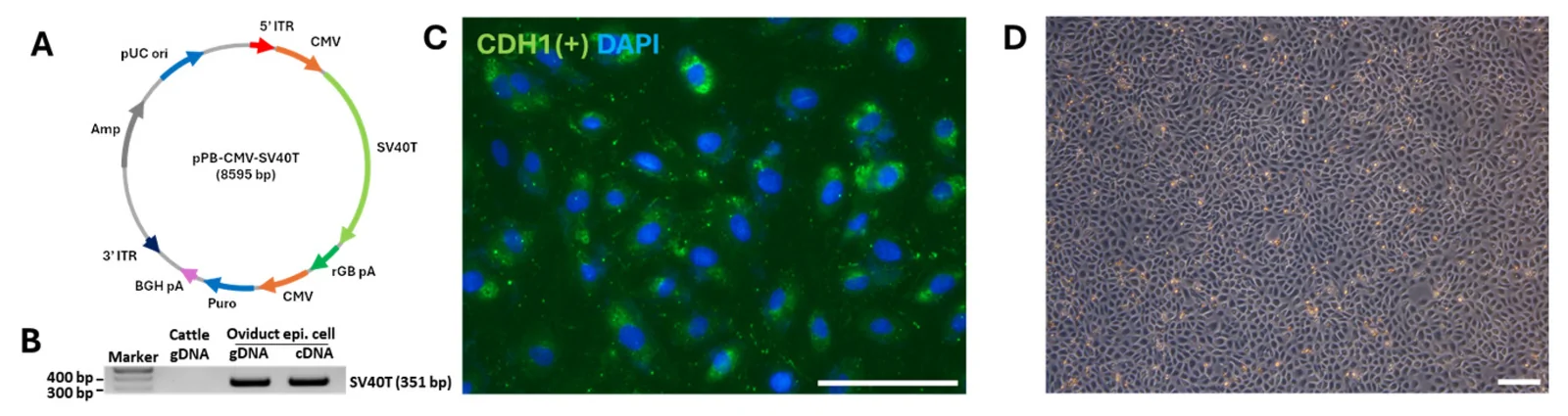
Development of an immortalized cattle oviduct epithelial cell (OEC) line for heat stress study. (A) Vector map of the *piggyBac*-based SV40T expression system utilized for immortalization. (B) PCR verification of SV40T insertion in OEC genomic DNA (gDNA) and complementary DNA (cDNA) of total mRNA in comparison with intact Hanwoo cattle gDNA. (C) Immunofluorescence signal of anti-CDH1 (green) and DAPI nuclear staining (blue) of the immortalized OEC (passage 13); scale bar = 100 μm. (D) Phase-contrast view of the developed immortalized OEC (passage 23); scale bar = 200 μm. PCR, polymerase chain reaction; CDH1, cadherin 1; DAPI, 4′,6-diamidino-2-phenylindole.

**Figure 3:**
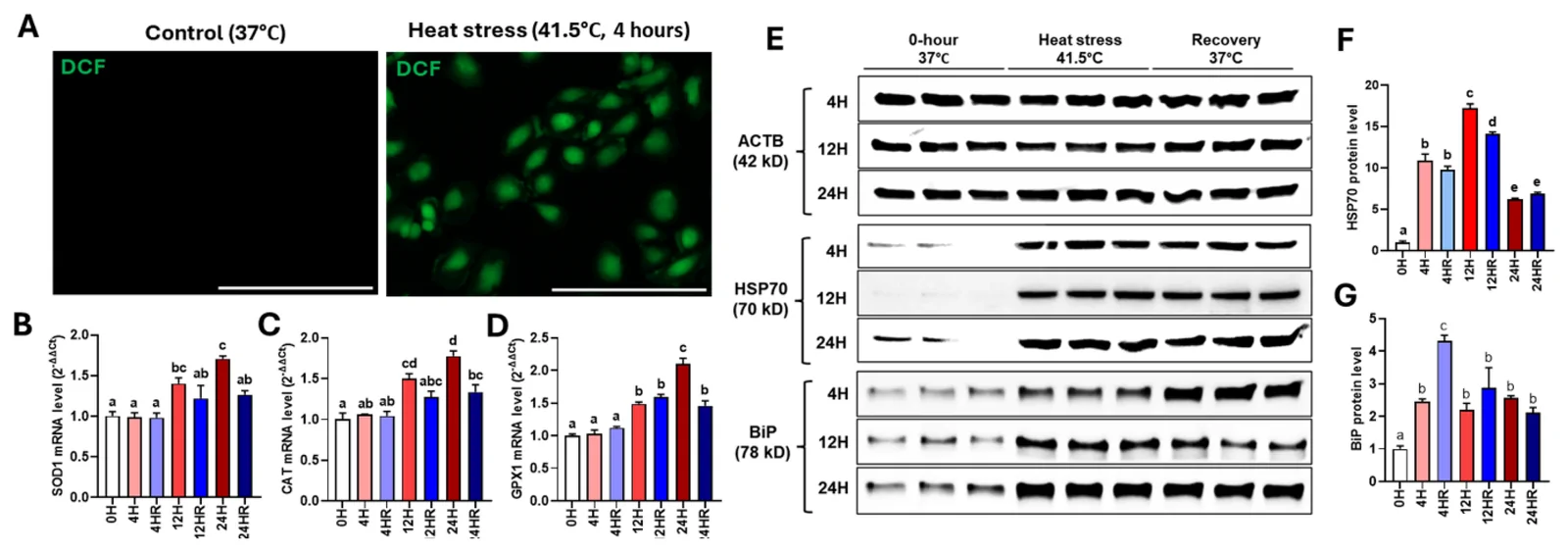
Optimization of heat-stress conditions in the immortalized cattle OEC. (A) Intracellular ROS detected by DCF signals after 4 h heat stress treatment at 41.5°C. (B–D) Relative value (2^−ΔΔCt^) of mRNA expression of oxidative-stress genes *SOD1* (B), *CAT* (C), and *GPX1* (D) after 4 h, 12 h, and 24 h period of heat stress and recovery quantified by qRT-PCR. (E) Protein relative expression to ACTB and control (0H) of heat-stress biomarkers (HSP70 and BiP) evaluated using HRP-conjugated antibody detection from 10% SDS-PAGE electrophoresis across the same treatment periods. (F–G) Normalized expression levels of HSP70 (F) and BiP (G) protein. Bar heights represent mean±SEM (n = 3). Statistical differences were evaluated by one-way ANOVA with Tukey’s test. ^a–e^ Different letters indicate significant differences (p≤0.05). 0H, control group; –H, hours of heat stress; –HR, hours of recovery; OEC, oviduct epithelial cell; ROS, reactive oxygen species; *SOD1*, *superoxide dismutase 1*; *CAT*, *catalase*; *GPX1*, *glutathione peroxidase 1*; qRT-PCR, quantitative reverse-transcription polymerase chain reaction; SDS-PAGE, sodium dodecyl sulfate–polyacrylamide gel electrophoresis; HSP70, heat shock protein 70; BiP, binding immunoglobulin protein.

**Figure 4:**
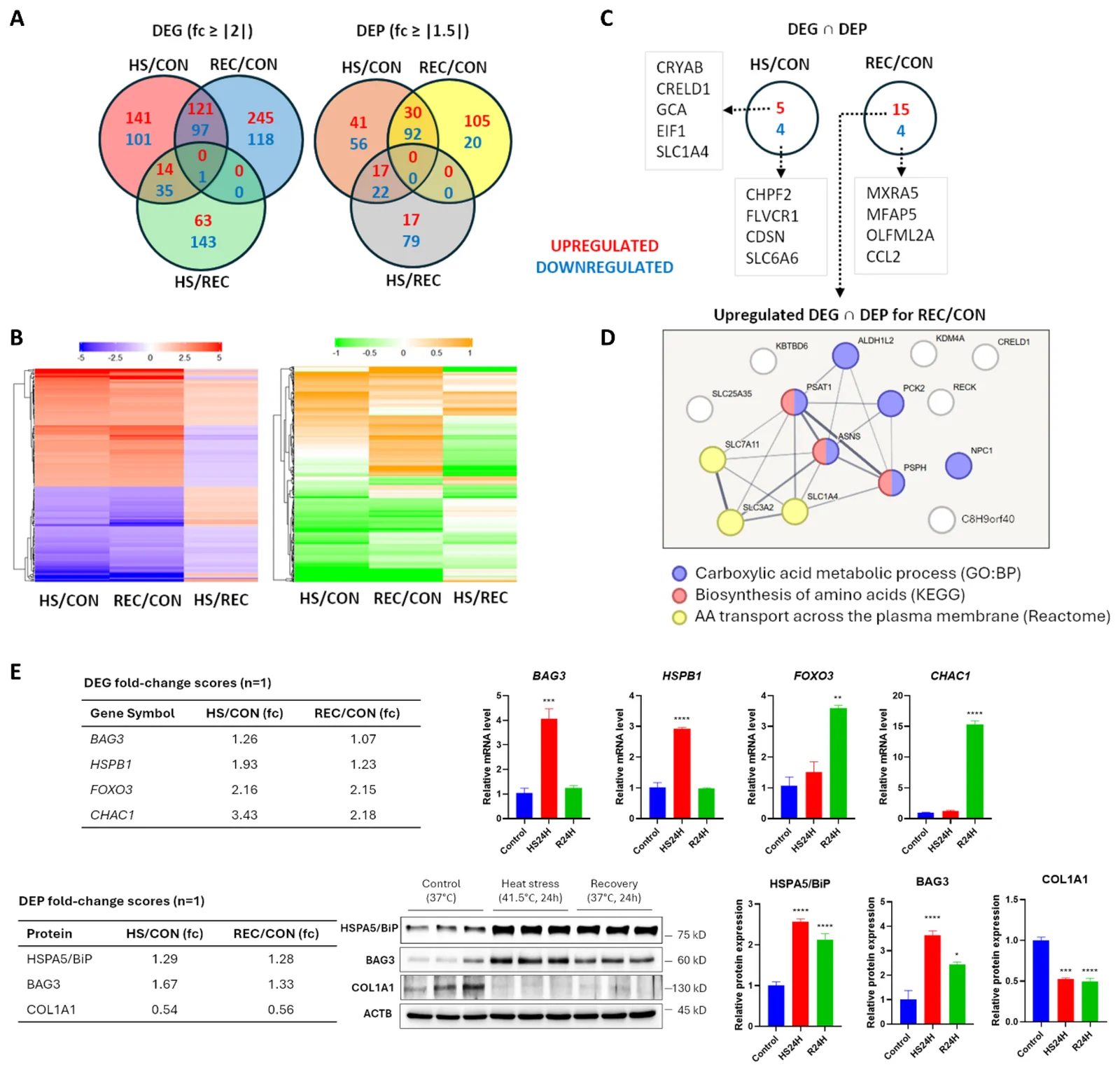
Differentially expressed gene (DEG) and protein (DEP) of the immortalized cattle OEC treated with 24 h thermal stress with subsequent 24 h recovery. (A) Venn diagram displaying upregulated and downregulated DEG (fc≥|2|) and DEP (fc≥|1.5|) overlaps. (B) Heatmap of DEG (fc score) and DEP (log2 score) grouped using Euclidean distance algorithm with scores≥|5| for DEG and ≥|1| for DEP are displayed at maximum level color. (C) Concordant DEG and DEP gene list from heat stress group (HS/CON) and recovery group (REC/CON) with (D) STRING protein–protein interaction networks and major enriched terms of REC/CON upregulated DEG. (E) Confirmatory mRNA and protein expression level of representative genes using qRT-PCR and Western Blotting (WB) (n = 3) compared to microarray and protein LC/MS results of pooled sample (n = 1) under heat stress and recovery periods. Fc, normalized fold-change; HS/CON, heat stress vs control; REC/CON, recovery vs control; HS/REC, heat stress vs recovery; HS24H, heat stress 24 h; R24H, recovery 24 h; CON, control treatment; OEC, oviduct epithelial cell; qRT-PCR, quantitative reverse-transcription polymerase chain reaction; LC/MS, liquid chromatography–mass spectrometry.

**Figure 5:**
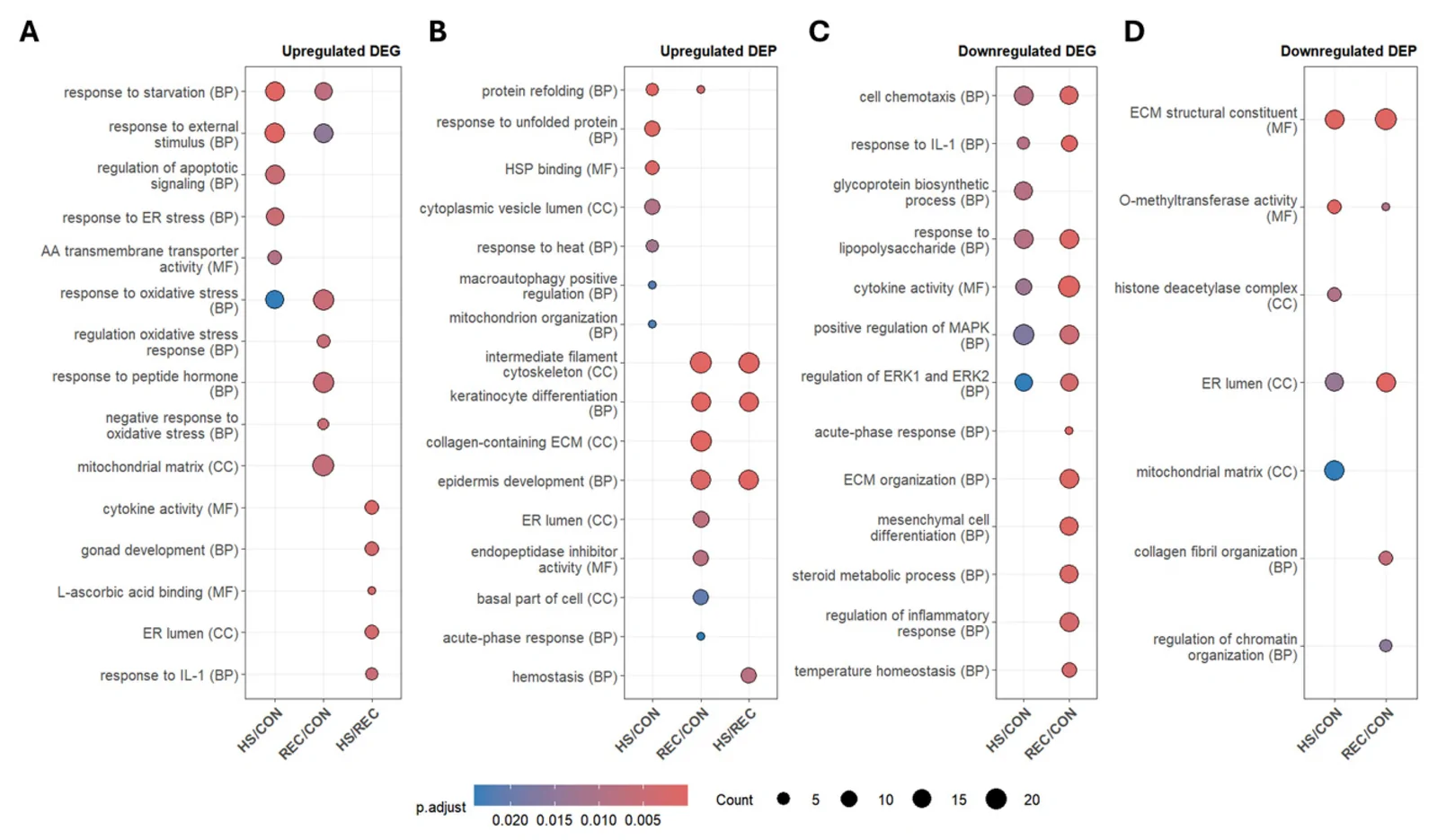
Dot plot of significantly enriched gene ontology terms (p≤0.05, q≤0.2) for biological processes (BP), molecular functions (MF), and cellular component (CC) analyzed from upregulated DEG (A), upregulated DEP (B), downregulated DEG (C), and downregulated DEP (D). Group x-axis labels which are not significant are not shown. DEG, differentially expressed gene; DEP, differentially expressed protein; HS/CON, heat stress vs control; REC/CON, recovery vs control; HS/REC, heat stress vs recovery.

**Figure 6:**
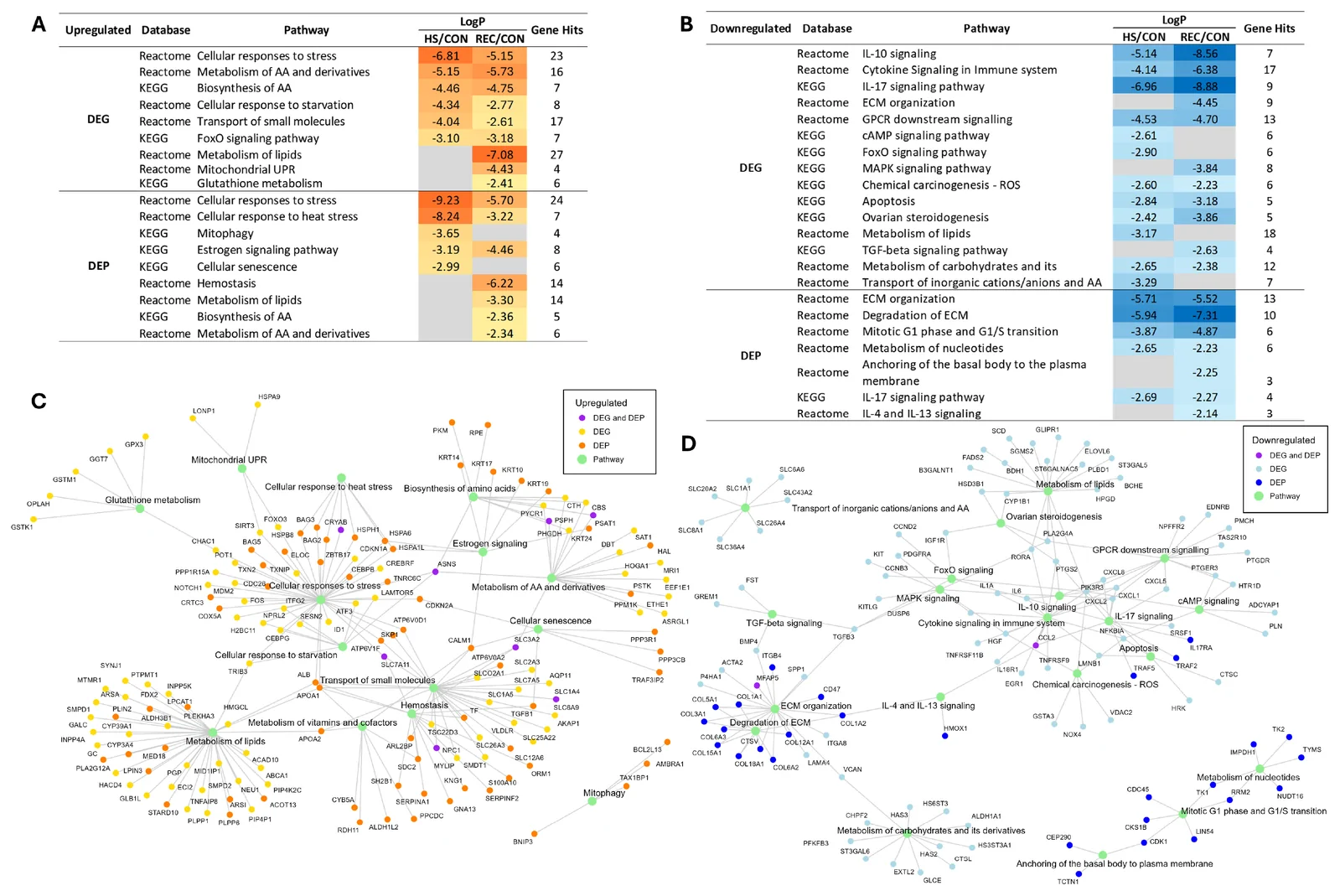
Pathway enrichment and pathway-gene network under heat stress based on KEGG and Reactome database. (A, B) Highlighted significantly enriched pathways of upregulated (A) and downregulated (B) DEG and DEP from HS/CON and REC/CON group showing logP enrichment score and number of genes matched with each pathway database. (C, D) Pathway-gene network of upregulated (C) and downregulated (D) DEG-DEP gene list. All dots were labelled with pathway names or gene symbols. Pathway enrichments were conducted under batch enrichment analysis in Metascape with p<0.01, minimum count = 3, and enrichment factor>1.5. HS/CON, heat stress vs control; REC/CON, recovery vs control; DEG, differentially expressed gene; DEP, differentially expressed protein.

## Data Availability

Upon reasonable request, the datasets of this study can be available from the corresponding author.

## SUPPLEMENTARY MATERIAL

**Supplement 1**. Immortalized oviduct epithelial cells figures under heat stress and recovery periods.

**Supplement 2**. List of primers used in this study.

**Supplement 3**. Gel electrophoresis of RT-PCR of the oxidative stress marker genes.

**Supplement 4**. Microarray normalized fold-change of immortalized oviduct epithelial cells under heat stress.

**Supplement 5**. Proteomics normalized log2 of immortalized oviduct epithelial cells under heat stress.

**Supplement 6**. Uncropped electrophoresis imaging of SV40T confirmation PCR.

**Supplement 7**. Scatter plot of FACS sorting of the oviduct epithelial cells using specific anti-CDH1 antibody.

**Supplement 8**. Oviduct epithelial cells characterization using transcriptomics and proteomics data.

**Supplement 9**. Uncropped Western Blot imaging of HSP70 and BiP.

**Supplement 10**. Oviduct epithelial cells Upregulated DEG - GO Enrichment (BP, MF, CC).

**Supplement 11**. Oviduct epithelial cells Downregulated DEG - GO Enrichment (BP, MF, CC).

**Supplement 12**. Oviduct epithelial cells Upregulated DEP - GO Enrichment (BP, MF, CC).

**Supplement 13**. Oviduct epithelial cells Downregulated DEP - GO Enrichment (BP, MF, CC).

**Supplement 14**. Oviduct epithelial cells Upregulated DEG Pathway Enrichment (KEGG and Reactome).

**Supplement 15**. Oviduct epithelial cells Downregulated DEG Pathway Enrichment (KEGG and Reactome).

**Supplement 16**. Oviduct epithelial cells Upregulated DEP Pathway Enrichment (KEGG and Reactome).

**Supplement 17**. Oviduct epithelial cells Downregulated DEP Pathway Enrichment (KEGG and Reactome).

## References

[1] Ascari IJ, Alves NG, Jasmin J (2017). Addition of insulin-like growth factor I to the maturation medium of bovine oocytes subjected to heat shock: effects on the production of reactive oxygen species, mitochondrial activity and oocyte competence. Domest Anim Endocrinol.

[2] Marquez-Acevedo AS, Hood WR, Collier RJ, Skibiel AL (2023). Graduate Student Literature Review: mitochondrial response to heat stress and its implications on dairy cattle bioenergetics, metabolism, and production. J Dairy Sci.

[3] Dash S, Chakravarty AK, Singh A, Upadhyay A, Singh M, Yousuf S (2016). Effect of heat stress on reproductive performances of dairy cattle and buffaloes: a review. Vet World.

[4] Dado-Senn B, Laporta J, Dahl GE (2020). Carry over effects of late-gestational heat stress on dairy cattle progeny. Theriogenology.

[5] Khan I, Mesalam A, Heo YS, Lee SH, Nabi G, Kong IK (2023). Heat stress as a barrier to successful reproduction and potential alleviation strategies in cattle. Animals.

[6] National Hanwoo Association (2022). Hanwoo cattle trend analysis November 2022 issue. National Hanwoo Association.

[7] Sa SJ, Jeong J, Cho J, Lee SH, Choi I (2018). Heat waves impair cytoplasmic maturation of oocytes and preimplantation development in Korean native cattle (Hanwoo). Korean J Agric Sci.

[8] Cho K, Song Y, Yeo JM (2021). Analysis of seasonal effect on Korean native cattle (Hanwoo) birth weight. J Anim Sci Technol.

[9] Li S, Winuthayanon W (2017). Oviduct: roles in fertilization and early embryo development. J Endocrinol.

[10] Caballero JN, Gervasi MG, Veiga MF (2014). Epithelial cadherin is present in bovine oviduct epithelial cells and gametes, and is involved in fertilization-related events. Theriogenology.

[11] Mahé C, Marco L, Laffont L (2025). Bovine ampullary and isthmic epithelial spheroids: proteomic profile and physiological features for in vitro studies of gamete-oviduct interactions. Reprod Biol.

[12] Kölle S, Dubielzig S, Reese S, Wehrend A, König P, Kummer W (2009). Ciliary transport, gamete interaction, and effects of the early embryo in the oviduct: ex vivo analyses using a new digital videomicroscopic system in the cow. Biol Reprod.

[13] Guérin P, El Mouatassim S, Ménézo Y (2001). Oxidative stress and protection against reactive oxygen species in the pre-implantation embryo and its surroundings. Hum Reprod Update.

[14] Talukder AK, Rashid MB, Yousef MS (2018). Oviduct epithelium induces interferon-tau in bovine day-4 embryos, which generates an anti-inflammatory response in immune cells. Sci Rep.

[15] Nakamura Y, Aihara R, Iwata H, Kuwayama T, Shirasuna K (2021). IL1B triggers inflammatory cytokine production in bovine oviduct epithelial cells and induces neutrophil accumulation via CCL2. Am J Reprod Immunol.

[16] Miyashita N, Akagi S, Somfai T, Hirao Y (2024). Serum-free spontaneously immortalized bovine oviduct epithelial cell conditioned medium promotes the early development of bovine in vitro fertilized embryos. J Reprod Dev.

[17] Schoen J, Bondzio A, Topp K, Einspanier R (2008). Establishment and characterization of an adherent pure epithelial cell line derived from the bovine oviduct. Theriogenology.

[18] Rapała L, Starzyński RR, Trzeciak PZ (2018). Influence of elevated temperature on bovine oviduct epithelial cells (BOECs). PLOS ONE.

[19] Stamperna K, Giannoulis T, Cañon-Beltrán K (2022). Oviductal epithelial cells transcriptome and extracellular vesicles characterization during thermoneutral and heat stress conditions in dairy cows. Theriogenology.

[20] Menjivar NG, Gad A, Thompson RE, Meyers MA, Hollinshead FK, Tesfaye D (2023). Bovine oviductal organoids: a multi-omics approach to capture the cellular and extracellular molecular response of the oviduct to heat stress. BMC Genom.

[21] Alemu TW, Pandey HO, Salilew Wondim D (2018). Oxidative and endoplasmic reticulum stress defense mechanisms of bovine granulosa cells exposed to heat stress. Theriogenology.

[22] Zhou Y, Zhou B, Pache L (2019). Metascape provides a biologist-oriented resource for the analysis of systems-level datasets. Nat Commun.

[23] Draghici S, Khatri P, Eklund AC, Szallasi Z (2006). Reliability and reproducibility issues in DNA microarray measurements. Trends Genet.

[24] Wang Y, Barbacioru C, Hyland F (2006). Large scale real-time PCR validation on gene expression measurements from two commercial long-oligonucleotide microarrays. BMC Genom.

[25] Joyce K, Gad A, Menjivar NG (2024). Seasonal environmental fluctuations alter the transcriptome dynamics of oocytes and granulosa cells in beef cows. J Ovarian Res.

[26] Auchère F, Rusnak F (2002). What is the ultimate fate of superoxide anion in vivo?. J Biol Inorg Chem.

[27] Koopman WJH, Nijtmans LGJ, Dieteren CEJ (2010). Mammalian mitochondrial complex I: biogenesis, regulation, and reactive oxygen species generation. Antioxid Redox Signal.

[28] Lei XG, Cheng WH, McClung JP (2007). Metabolic regulation and function of glutathione peroxidase-1. Annu Rev Nutr.

[29] Ozawa M, Matsuzuka T, Hirabayashi M, Kanai Y (2004). Redox status of the oviduct and CDC2 activity in 2-cell stage embryos in heat-stressed mice. Biol Reprod.

[30] Mahé C, Lavigne R, Com E (2022). Spatiotemporal profiling of the bovine oviduct fluid proteome around the time of ovulation. Sci Rep.

[31] Alagar Boopathy LR, Jacob-Tomas S, Alecki C, Vera M (2022). Mechanisms tailoring the expression of heat shock proteins to proteostasis challenges. J Biol Chem.

[32] Lamy J, Labas V, Harichaux G, Tsikis G, Mermillod P, Saint-Dizier M (2016). Regulation of the bovine oviductal fluid proteome. Reproduction.

[33] Ali I, Shah SZA, Jin Y, Li ZS, Ullah O, Fang NZ (2017). Reactive oxygen species-mediated unfolded protein response pathways in preimplantation embryos. J Vet Sci.

[34] Marin-Briggiler CI, González-Echeverría MF, Munuce MJ (2010). Glucose-regulated protein 78 (Grp78/BiP) is secreted by human oviduct epithelial cells and the recombinant protein modulates sperm–zona pellucida binding. Fertil Steril.

[35] Velarde MC, Menon R (2016). Positive and negative effects of cellular senescence during female reproductive aging and pregnancy. J Endocrinol.

[36] Beier HM (1974). Oviducal and uterine fluids. J Reprod Fertil.

[37] Calderwood SK, Khaleque MA, Sawyer DB, Ciocca DR (2006). Heat shock proteins in cancer: chaperones of tumorigenesis. Trends Biochem Sci.

[38] Lilyestrom W, Klein MG, Zhang R, Joachimiak A, Chen XS (2006). Crystal structure of SV40 large T-antigen bound to p53: interplay between a viral oncoprotein and a cellular tumor suppressor. Genes Dev.

[39] Shaleh I, Kim SW, Park TS, Park J (2025). Mechanistic understanding of heat stress in cattle reproduction toward pharmacological strategies: review. Anim Biosci.

