# Supplementary material for: Development and characterization of Hanwoo oviduct epithelial cell line for heat stress study
Source: Anim Biosci. 2026 Sep 1;39(11):250962. doi: 10.5713/ab.250962 (PMC13740644; doi:10.5713/ab.250962)

Supplement 1. Immortalized oviduct epithelial cells figures under heat stress and recovery periods.

Immortalized oviduct epithelial cells (p24) were treated with heat stress (HS-) at 41.5°C and subsequent recovery (R-) at 37°C for 4h, 12h, and 24h periods. Scale bar = 200 µm.

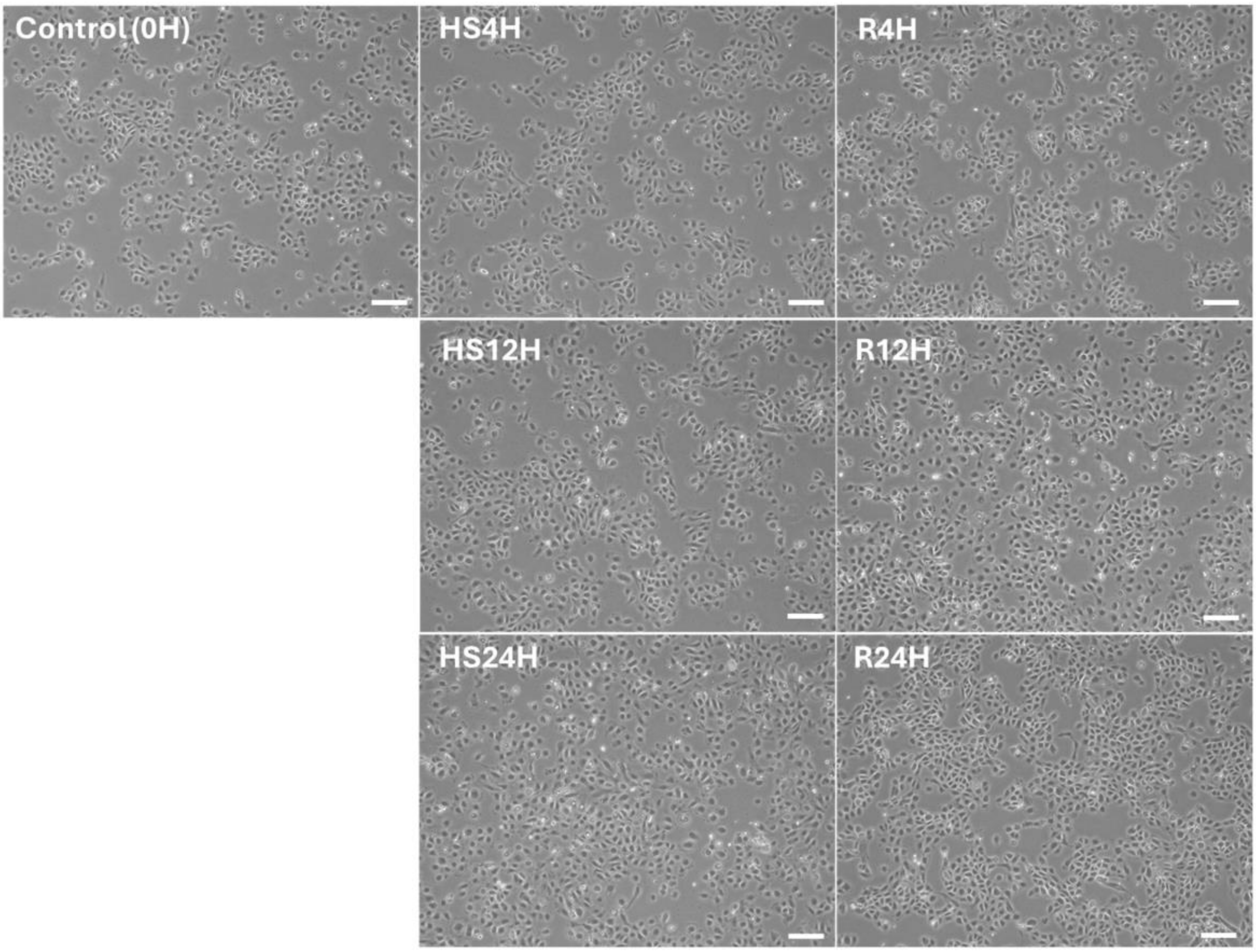

Supplement: Supplementary file 1 [file ab-250962-Supplementary-1.pdf]
