# Supplementary material for: Development and characterization of Hanwoo oviduct epithelial cell line for heat stress study
Source: Anim Biosci. 2026 Sep 1;39(11):250962. doi: 10.5713/ab.250962 (PMC13740644; doi:10.5713/ab.250962)
Supplement: Supplementary file 2 [file ab-250962-Supplementary-10.pdf]

Supplement 10. Oviduct epithelial cells Upregulated DEG - GO Enrichment (BP, MF, CC).

Cluster	ONTOLOGY	ID	Description	GeneRatio	BgRatio	pvalue	p.adjust	qvalue	geneID	Count
HS/REC	MF	GO:0005125	cytokine activity	7/56	237/18496	7.05542E-06	0.001396973	0.001188281	INHBB/SPP1/NRG1/KITLG/IL1A/EDN1/IL6	7
HS/REC	CC	GO:0005788	endoplasmic reticulum lumen	7/56	313/19886	2.67989E-05	0.003162272	0.002933776	P4HA1/SPP1/P4HA2/VCAN/MXRA8/EDN1/IL6	7
REC/CON	CC	GO:0005759	mitochondrial matrix	21/297	487/19886	1.50524E-05	0.005975786	0.005799119	HMGCL/FOXO3/DBT/NUDT9/ETHE1/SIRT3/ISCA2/NME4/ETFB/NSUN3/LONP1/ACAD10/ALDH1L2/HOGA1/PPM1K/GLRX2/HSPA9/MTHFS/BTD/PYCR1/PCK2	21
HS/CON	BP	GO:0042594	response to starvation	16/218	218/18870	5.25249E-09	1.62827E-05	1.45909E-05	LRP11/ITFG2/PMAIP1/FOXO3/STK26/JMY/ATF3/ULK1/CDKN1A/NPRL2/KLF10/SESN2/FOS/SLC7A5/ASNS/PCK2	16
HS/CON	BP	GO:0071496	cellular response to external stimulus	18/218	346/18870	1.19366E-07	9.25085E-05	8.28964E-05	ITFG2/PMAIP1/GDAP1/FOXO3/STK26/JMY/LTBR/ATF3/ULK1/CDKN1A/NPRL2/KLF10/SESN2/GADD45A/FOS/MDM2/SLC7A5/ASNS	18
HS/REC	BP	GO:0008406	gonad development	7/56	233/18870	5.5438E-06	0.002615501	0.001953368	INHBB/SF1/KITLG/CYP1B1/IL1A/ESR1/FST	7
REC/CON	BP	GO:0071375	cellular response to peptide hormone stimulus	17/290	310/18870	6.66697E-06	0.003633497	0.0033791	GKAP1/ECHDC3/PIP4K2C/CPEB1/TRIB3/INPP5K/RB1/AGTRAP/SGCB/VAMP2/AGTR1/LONP1/DDR2/MDM2/MIR107/GDF15/PCK2	17
REC/CON	BP	GO:0043434	response to peptide hormone	20/290	430/18870	1.20816E-05	0.004886592	0.00454446	GKAP1/ECHDC3/PIP4K2C/CPEB1/TRIB3/INPP5K/RB1/SESN2/AGTRAP/CITED1/EIF6/SGCB/VAMP2/AGTR1/LONP1/DDR2/MDM2/MIR107/GDF15/PCK2	20
REC/CON	BP	GO:1902882	regulation of response to oxidative stress	6/290	35/18870	1.39576E-05	0.004886592	0.00454446	H19/OXR1/TBC1D24/SESN2/GGT7/SLC7A11	6
REC/CON	BP	GO:0006979	response to oxidative stress	19/290	400/18870	1.49437E-05	0.004886592	0.00454446	TXNIP/H19/GPX3/FOXO3/STK26/OXR1/TBC1D24/SESN2/GGT7/KCNA5/LONP1/DDR2/ALDH3B1/GLRX2/MDM2/PTPRK/MIR107/PYCR1/SLC7A11	19
REC/CON	BP	GO:1902883	negative regulation of response to oxidative stress	4/290	11/18870	1.65613E-05	0.004923208	0.004578512	H19/OXR1/TBC1D24/GGT7	4
HS/CON	BP	GO:0034976	response to endoplasmic reticulum stress	13/218	266/18870	1.39498E-05	0.00499369	0.004474821	CLU/FLOT1/FOXRED2/PARP6/PMAIP1/ATF3/TRIB3/AQP11/SESN2/RHBDD1/PPP1R15A/CHAC1/CTH	13
HS/CON	BP	GO:2001233	regulation of apoptotic signaling pathway	16/218	398/18870	1.66519E-05	0.00499369	0.004474821	CLU/STRADB/GHITM/PMAIP1/LTBR/ATF3/MAP2K5/ARHGEF2/PRKRA/MIR27B/EEF1E1/ICAM1/TPD52L1/MDM2/CTH/PYCR1	16
HS/REC	BP	GO:0071347	cellular response to interleukin-1	5/56	111/18870	1.93485E-05	0.005146026	0.003843272	INHBB/EDN1/CAMP/EGR1/IL6	5
REC/CON	BP	GO:0034599	cellular response to oxidative stress	14/290	255/18870	4.30378E-05	0.008278455	0.007698843	H19/FOXO3/OXR1/TBC1D24/SESN2/LONP1/DDR2/ALDH3B1/GLRX2/MDM2/PTPRK/MIR107/PYCR1/SLC7A11	14
REC/CON	BP	GO:0071496	cellular response to external stimulus	16/290	346/18870	9.68813E-05	0.015085804	0.014029579	GABARAP/PMAIP1/FOXO3/STK26/JMY/CDKN1A/NPRL2/KLF10/SESN2/CPEB4/GLRX2/TLR4/GADD45A/MDM2/CBS/ASNS	16
HS/REC	BP	GO:0008585	female gonad development	4/56	101/18870	0.000229336	0.019819467	0.014802025	INHBB/KITLG/ESR1/FST	4
HS/CON	BP	GO:0034599	cellular response to oxidative stress	11/218	255/18870	0.000194007	0.02147938	0.019247567	FOXO3/SESN2/PRKRA/BRF2/DDR2/EDN1/FOS/MDM2/MIR107/PYCR1/SLC7A11	11
HS/REC	BP	GO:0000302	response to reactive oxygen species	5/56	205/18870	0.000351048	0.023697286	0.017698146	CRYAB/CYP1B1/IL1A/EDN1/IL6	5
HS/CON	BP	GO:0006979	response to oxidative stress	14/218	400/18870	0.000241698	0.024169761	0.021658404	CRYAB/TXNIP/FOXO3/STK26/SESN2/PRKRA/BRF2/DDR2/EDN1/FOS/MDM2/MIR107/PYCR1/SLC7A11	14
HS/REC	BP	GO:0045765	regulation of angiogenesis	6/56	349/18870	0.000571219	0.024656446	0.018414488	CYP1B1/VEGFC/IL1A/CAMP/F3/IL6	6
HS/REC	BP	GO:0035270	endocrine system development	4/56	139/18870	0.000769538	0.024776098	0.018503849	INHBB/CYP1B1/EDN1/IL6	4
HS/REC	BP	GO:0044849	estrous cycle	2/56	15/18870	0.000886033	0.027396811	0.02046111	CYP1B1/EGR1	2
HS/CON	BP	GO:0062197	cellular response to chemical stress	12/218	317/18870	0.000333211	0.027917666	0.025016883	FOXO3/SESN2/ARHGEF2/PRKRA/BRF2/DDR2/EDN1/FOS/MDM2/MIR107/PYCR1/SLC7A11	12
HS/CON	BP	GO:0072593	reactive oxygen species metabolic process	10/218	234/18870	0.000406936	0.029337223	0.026288941	CRYAB/RNF41/PMAIP1/CDKN1A/SESN2/SIRT3/MIR27B/DDIT4/GADD45A/EDN1	10
HS/REC	BP	GO:0032354	response to follicle-stimulating hormone	2/56	16/18870	0.001010682	0.029634629	0.022132407	CYP1B1/EDN1	2
HS/REC	BP	GO:0035234	ectopic germ cell programmed cell death	2/56	18/18870	0.001283721	0.033997892	0.025391079	KITLG/IL1A	2
HS/REC	BP	GO:0009266	response to temperature stimulus	4/56	171/18870	0.001656848	0.040605143	0.030325656	CRYAB/SCN9A/CHORDC1/IL1A	4
HS/CON	BP	GO:0000302	response to reactive oxygen species	9/218	205/18870	0.000650642	0.041163034	0.036885992	CRYAB/TXNIP/STK26/SESN2/DDR2/EDN1/FOS/MDM2/MIR107	9
REC/CON	BP	GO:0062197	cellular response to chemical stress	14/290	317/18870	0.000417909	0.045552095	0.042362788	H19/FOXO3/OXR1/TBC1D24/SESN2/LONP1/DDR2/ALDH3B1/GLRX2/MDM2/PTPRK/MIR107/PYCR1/SLC7A11	14
HS/REC	BP	GO:0010623	programmed cell death involved in cell development	2/56	25/18870	0.002483799	0.049736569	0.037145396	KITLG/IL1A	2
HS/REC	MF	GO:0031418	L-ascorbic acid binding	3/56	20/18496	2.88943E-05	0.002860538	0.002433206	P4HA1/PLOD2/P4HA2	3
HS/CON	MF	GO:0015171	amino acid transmembrane transporter activity	7/223	85/18496	7.49478E-05	0.009218584	0.008658448	SLC3A2/SLC25A22/SLC6A9/SLC1A4/SLC1A5/SLC7A5/SLC7A11	7
REC/CON	MF	GO:0034593	phosphatidylinositol bisphosphate phosphatase activity	5/293	26/18496	4.82652E-05	0.027849005	0.025453528	MTMR1/SYNJ1/INPP5K/INPP4A/PTPMT1	5
HS/REC	MF	GO:0030246	carbohydrate binding	5/56	273/18496	0.001395465	0.030121205	0.02562144	P4HA1/PLOD2/P4HA2/GALNT16/VCAN	5
HS/CON	MF	GO:0008514	organic anion transmembrane transporter activity	10/223	242/18496	0.000735111	0.048908395	0.045936639	SLCO2A1/SLC3A2/SLC25A22/SLC26A3/SLC6A9/SLC1A4/SLC2A3/SLC1A5/SLC7A5/SLC7A11	10
HS/CON	MF	GO:0015179	L-amino acid transmembrane transporter activity	7/223	66/18496	1.43504E-05	0.004423192	0.004154431	SLC3A2/SLC25A22/SLC6A9/SLC1A4/SLC1A5/SLC7A5/SLC7A11	7
HS/CON	MF	GO:0015175	neutral L-amino acid transmembrane transporter activity	6/223	46/18496	1.79805E-05	0.004423192	0.004154431	SLC3A2/SLC6A9/SLC1A4/SLC1A5/SLC7A5/SLC7A11	6
HS/REC	MF	GO:0008083	growth factor activity	5/56	162/18496	0.000129055	0.008517597	0.007245165	INHBB/NRG1/KITLG/VEGFC/IL6	5
HS/CON	MF	GO:0015172	acidic amino acid transmembrane transporter activity	4/223	18/18496	5.51162E-05	0.00903905	0.008489822	SLC25A22/SLC1A4/SLC1A5/SLC7A11	4
HS/REC	MF	GO:0031543	peptidyl-proline dioxygenase activity	2/56	12/18496	0.000582785	0.026620187	0.022643434	P4HA1/P4HA2	2
HS/REC	MF	GO:0016706	2-oxoglutarate-dependent dioxygenase activity	3/56	60/18496	0.000796063	0.026620187	0.022643434	P4HA1/PLOD2/P4HA2	3
HS/REC	MF	GO:0070851	growth factor receptor binding	4/56	138/18496	0.000806672	0.026620187	0.022643434	VEGFC/IL1A/ESM1/IL6	4
REC/CON	MF	GO:0052866	phosphatidylinositol phosphate phosphatase activity	5/293	30/18496	9.92781E-05	0.028641741	0.026178076	MTMR1/SYNJ1/INPP5K/INPP4A/PTPMT1	5
HS/REC	MF	GO:0019842	vitamin binding	4/56	150/18496	0.001100099	0.029298301	0.024921469	P4HA1/PLOD2/P4HA2/SCLY	4
HS/REC	MF	GO:0005506	iron ion binding	4/56	153/18496	0.00118377	0.029298301	0.024921469	P4HA1/PLOD2/P4HA2/CYP1B1	4
HS/REC	MF	GO:0048029	monosaccharide binding	3/56	75/18496	0.001521273	0.030121205	0.02562144	P4HA1/PLOD2/P4HA2	3
HS/REC	MF	GO:0016705	oxidoreductase activity, acting on paired donors, with incorporation or reduction of molecular oxygen	4/56	179/18496	0.00210482	0.037886758	0.032226908	P4HA1/PLOD2/P4HA2/CYP1B1	4
HS/REC	MF	GO:0051213	dioxygenase activity	3/56	93/18496	0.00281335	0.043689779	0.037163023	P4HA1/PLOD2/P4HA2	3
HS/REC	MF	GO:0031406	carboxylic acid binding	4/56	195/18496	0.002868521	0.043689779	0.037163023	P4HA1/PLOD2/P4HA2/SCLY	4
HS/CON	MF	GO:0005310	dicarboxylic acid transmembrane transporter activity	4/223	31/18496	0.000501223	0.048908395	0.045936639	SLC25A22/SLC26A3/SLC1A4/SLC1A5	4
HS/CON	MF	GO:0015556	C4-dicarboxylate transmembrane transporter activity	3/223	15/18496	0.000706864	0.048908395	0.045936639	SLC25A22/SLC1A4/SLC1A5	3
HS/CON	MF	GO:0046943	carboxylic acid transmembrane transporter activity	8/223	164/18496	0.000860035	0.048908395	0.045936639	SLC3A2/SLC25A22/SLC26A3/SLC6A9/SLC1A4/SLC1A5/SLC7A5/SLC7A11	8
HS/CON	MF	GO:0005342	organic acid transmembrane transporter activity	8/223	165/18496	0.000894666	0.048908395	0.045936639	SLC3A2/SLC25A22/SLC26A3/SLC6A9/SLC1A4/SLC1A5/SLC7A5/SLC7A11	8
HS/CON	BP	GO:0031669	cellular response to nutrient levels	16/218	251/18870	3.86526E-08	5.99115E-05	5.36864E-05	ITFG2/PMAIP1/GDAP1/FOXO3/STK26/JMY/ATF3/ULK1/CDKN1A/NPRL2/KLF10/SESN2/FOS/MDM2/SLC7A5/ASNS	16
HS/CON	BP	GO:0031667	response to nutrient levels	22/218	495/18870	7.33727E-08	7.58184E-05	6.79405E-05	LRP11/ITFG2/PMAIP1/GDAP1/FOXO3/STK26/JMY/ATF3/MKKS/ULK1/CDKN1A/PPARG/NPRL2/KLF10/SESN2/FOS/MDM2/SLC7A5/ASNS/GDF15/PSPH/PCK2	22
HS/CON	BP	GO:0009267	cellular response to starvation	13/218	179/18870	1.6835E-07	9.45459E-05	8.47221E-05	ITFG2/PMAIP1/FOXO3/STK26/JMY/ATF3/CDKN1A/NPRL2/KLF10/SESN2/FOS/SLC7A5/ASNS	13
HS/CON	BP	GO:0031668	cellular response to extracellular stimulus	16/218	281/18870	1.82992E-07	9.45459E-05	8.47221E-05	ITFG2/PMAIP1/GDAP1/FOXO3/STK26/JMY/ATF3/ULK1/CDKN1A/NPRL2/KLF10/SESN2/FOS/MDM2/SLC7A5/ASNS	16
REC/CON	BP	GO:0008652	amino acid biosynthetic process	10/290	77/18870	2.78548E-07	0.000850937	0.000791359	MTHFR/MRI1/PHGDH/DPYD/CBS/ASNS/PSPH/CTH/PSAT1/PYCR1	10
REC/CON	BP	GO:0006520	amino acid metabolic process	18/290	291/18870	6.40337E-07	0.000850937	0.000791359	HMGCL/DBT/ASRGL1/ETFB/SLC7A7/MTHFR/HOGA1/MRI1/PHGDH/DPYD/MTHFS/CBS/ASNS/PSPH/CTH/PSAT1/PYCR1/SLC7A11	18
REC/CON	BP	GO:1901605	alpha-amino acid metabolic process	15/290	207/18870	7.80676E-07	0.000850937	0.000791359	HMGCL/ASRGL1/SLC7A7/MTHFR/HOGA1/MRI1/PHGDH/MTHFS/CBS/ASNS/PSPH/CTH/PSAT1/PYCR1/SLC7A11	15
REC/CON	BP	GO:1901607	alpha-amino acid biosynthetic process	9/290	69/18870	1.06812E-06	0.000873189	0.000812053	MTHFR/MRI1/PHGDH/CBS/ASNS/PSPH/CTH/PSAT1/PYCR1	9
REC/CON	BP	GO:0031667	response to nutrient levels	23/290	495/18870	2.69538E-06	0.001762779	0.001639359	LRP11/GABARAP/PMAIP1/FOXO3/STK26/JMY/TMEM135/CDKN1A/KDM4A/ABCA1/NPRL2/KLF10/SESN2/CPEB4/SST/MTHFR/BMP8A/MDM2/CBS/ASNS/GDF15/PSPH/PCK2	23
HS/REC	BP	GO:0008584	male gonad development	6/56	150/18870	5.33992E-06	0.002615501	0.001953368	INHBB/SF1/KITLG/CYP1B1/IL1A/ESR1	6
HS/REC	BP	GO:0046546	development of primary male sexual characteristics	6/56	151/18870	5.54825E-06	0.002615501	0.001953368	INHBB/SF1/KITLG/CYP1B1/IL1A/ESR1	6
HS/REC	BP	GO:0045137	development of primary sexual characteristics	7/56	238/18870	6.3715E-06	0.002615501	0.001953368	INHBB/SF1/KITLG/CYP1B1/IL1A/ESR1/FST	7
HS/CON	BP	GO:0000101	sulfur amino acid transport	4/218	12/18870	7.97682E-06	0.003532592	0.003165538	SLC3A2/SLC1A4/SLC7A5/SLC7A11	4
HS/REC	BP	GO:0046661	male sex differentiation	6/56	174/18870	1.24945E-05	0.00410318	0.00306443	INHBB/SF1/KITLG/CYP1B1/IL1A/ESR1	6
REC/CON	BP	GO:0009070	serine family amino acid biosynthetic process	5/290	21/18870	1.3773E-05	0.004886592	0.00454446	PHGDH/CBS/PSPH/CTH/PSAT1	5
HS/CON	BP	GO:1901607	alpha-amino acid biosynthetic process	7/218	69/18870	1.46587E-05	0.00499369	0.004474821	MRI1/PHGDH/ASNS/PSPH/CTH/PSAT1/PYCR1	7
HS/CON	BP	GO:1902475	L-alpha-amino acid transmembrane transport	7/218	71/18870	1.77195E-05	0.00499369	0.004474821	SLC3A2/SLC25A22/SLC6A9/SLC1A4/SLC1A5/SLC7A5/SLC7A11	7
HS/REC	BP	GO:0007548	sex differentiation	7/56	288/18870	2.1938E-05	0.005146026	0.003843272	INHBB/SF1/KITLG/CYP1B1/IL1A/ESR1/FST	7
REC/CON	BP	GO:0032868	response to insulin	15/290	269/18870	1.91781E-05	0.005226031	0.004860133	GKAP1/ECHDC3/PIP4K2C/CPEB1/TRIB3/INPP5K/RB1/SESN2/CITED1/EIF6/SGCB/VAMP2/LONP1/MIR107/PCK2	15
REC/CON	BP	GO:1901653	cellular response to peptide	18/290	374/18870	2.11001E-05	0.005307475	0.004935875	GKAP1/ECHDC3/PIP4K2C/CPEB1/TRIB3/INPP5K/RB1/AGTRAP/SGCB/VAMP2/AGTR1/LONP1/DDR2/TLR4/MDM2/MIR107/GDF15/PCK2	18
HS/CON	BP	GO:0089718	amino acid import across plasma membrane	6/218	50/18870	2.30749E-05	0.005502485	0.00493075	SLC3A2/SLC6A9/SLC1A4/SLC1A5/SLC7A5/SLC7A11	6
HS/CON	BP	GO:0042149	cellular response to glucose starvation	6/218	50/18870	2.30749E-05	0.005502485	0.00493075	ITFG2/PMAIP1/FOXO3/SESN2/SLC7A5/ASNS	6
HS/REC	BP	GO:0048608	reproductive structure development	7/56	305/18870	3.16592E-05	0.006275834	0.004687061	INHBB/SF1/KITLG/CYP1B1/IL1A/ESR1/FST	7
HS/REC	BP	GO:0061458	reproductive system development	7/56	309/18870	3.43986E-05	0.006275834	0.004687061	INHBB/SF1/KITLG/CYP1B1/IL1A/ESR1/FST	7
HS/CON	BP	GO:0008652	amino acid biosynthetic process	7/218	77/18870	3.02199E-05	0.006691546	0.005996262	MRI1/PHGDH/ASNS/PSPH/CTH/PSAT1/PYCR1	7
HS/REC	BP	GO:0018126	protein hydroxylation	3/56	23/18870	4.20353E-05	0.006902201	0.005154858	P4HA1/PLOD2/P4HA2	3
REC/CON	BP	GO:0009069	serine family amino acid metabolic process	6/290	40/18870	3.09458E-05	0.007157353	0.006656235	PHGDH/CBS/PSPH/CTH/PSAT1/SLC7A11	6
REC/CON	BP	GO:0042594	response to starvation	13/290	218/18870	3.44972E-05	0.007157353	0.006656235	LRP11/GABARAP/PMAIP1/FOXO3/STK26/JMY/CDKN1A/NPRL2/KLF10/SESN2/CPEB4/ASNS/PCK2	13
REC/CON	BP	GO:0006563	L-serine metabolic process	4/290	13/18870	3.50207E-05	0.007157353	0.006656235	PHGDH/CBS/PSPH/PSAT1	4
HS/REC	BP	GO:0070555	response to interleukin-1	5/56	140/18870	5.89722E-05	0.008802936	0.006574409	INHBB/EDN1/CAMP/EGR1/IL6	5
HS/CON	BP	GO:0070482	response to oxygen levels	14/218	343/18870	4.81008E-05	0.009940835	0.008907933	CRYAB/PLOD1/PMAIP1/PLAT/CDKN1A/NOTCH1/PPARG/DDR2/DDIT4/RGCC/EDN1/FOS/MDM2/SLC7A5	14
HS/REC	BP	GO:0034698	response to gonadotropin	3/56	28/18870	7.69432E-05	0.010011359	0.007476911	INHBB/CYP1B1/EDN1	3
HS/REC	BP	GO:0002687	positive regulation of leukocyte migration	5/56	149/18870	7.92617E-05	0.010011359	0.007476911	KITLG/VEGFC/IL1A/EDN1/IL6	5
HS/CON	BP	GO:0015804	neutral amino acid transport	6/218	58/18870	5.44283E-05	0.010545482	0.009449755	SLC3A2/SLC6A9/SLC1A4/SLC1A5/SLC7A5/SLC7A11	6
HS/REC	BP	GO:0010575	positive regulation of vascular endothelial growth factor production	3/56	30/18870	9.49565E-05	0.011137045	0.008317621	CYP1B1/IL1A/IL6	3
REC/CON	BP	GO:0046394	carboxylic acid biosynthetic process	16/290	334/18870	6.4098E-05	0.011644466	0.010829185	TRIB3/CYP39A1/MID1IP1/GGT7/EIF6/MTHFR/HOGA1/MRI1/PHGDH/DPYD/CBS/ASNS/PSPH/CTH/PSAT1/PYCR1	16
REC/CON	BP	GO:0016053	organic acid biosynthetic process	16/290	337/18870	7.12106E-05	0.012255725	0.011397647	TRIB3/CYP39A1/MID1IP1/GGT7/EIF6/MTHFR/HOGA1/MRI1/PHGDH/DPYD/CBS/ASNS/PSPH/CTH/PSAT1/PYCR1	16
HS/CON	BP	GO:0015807	L-amino acid transport	7/218	90/18870	8.27175E-05	0.013729821	0.012303225	SLC3A2/SLC25A22/SLC6A9/SLC1A4/SLC1A5/SLC7A5/SLC7A11	7
HS/CON	BP	GO:2000047	regulation of cell-cell adhesion mediated by cadherin	4/218	21/18870	8.88801E-05	0.013729821	0.012303225	FLOT1/TJP1/NOTCH1/RGCC	4
HS/CON	BP	GO:0009070	serine family amino acid biosynthetic process	4/218	21/18870	8.88801E-05	0.013729821	0.012303225	PHGDH/PSPH/CTH/PSAT1	4
HS/CON	BP	GO:0097193	intrinsic apoptotic signaling pathway	13/218	319/18870	9.11155E-05	0.013729821	0.012303225	CLU/PMAIP1/JMY/CDKN1A/TRIB3/ARHGEF2/PRKRA/MIR27B/DDIT4/PPP1R15A/MDM2/CHAC1/PYCR1	13
HS/CON	BP	GO:0009069	serine family amino acid metabolic process	5/218	40/18870	9.30462E-05	0.013729821	0.012303225	PHGDH/PSPH/CTH/PSAT1/SLC7A11	5
HS/CON	BP	GO:0035966	response to topologically incorrect protein	9/218	159/18870	9.88853E-05	0.013729821	0.012303225	CLU/CREBRF/PARP6/KLHL15/ATF3/RHBDD1/PPP1R15A/CHAC1/CTH	9
HS/CON	BP	GO:1904705	regulation of vascular associated smooth muscle cell proliferation	7/218	93/18870	0.000101866	0.013729821	0.012303225	CDKN1A/PPARG/MIR27B/DDR2/CALCRL/EDN1/MDM2	7
REC/CON	BP	GO:0032869	cellular response to insulin stimulus	12/290	206/18870	8.62398E-05	0.014100201	0.013112982	GKAP1/ECHDC3/PIP4K2C/CPEB1/TRIB3/INPP5K/RB1/SGCB/VAMP2/LONP1/MIR107/PCK2	12
HS/CON	BP	GO:1990874	vascular associated smooth muscle cell proliferation	7/218	95/18870	0.000116528	0.015051485	0.013487561	CDKN1A/PPARG/MIR27B/DDR2/CALCRL/EDN1/MDM2	7
REC/CON	BP	GO:0009267	cellular response to starvation	11/290	179/18870	0.000105586	0.015693968	0.014595163	GABARAP/PMAIP1/FOXO3/STK26/JMY/CDKN1A/NPRL2/KLF10/SESN2/CPEB4/ASNS	11
REC/CON	BP	GO:0000097	sulfur amino acid biosynthetic process	4/290	17/18870	0.000111047	0.015787926	0.014682543	MTHFR/MRI1/CBS/CTH	4
REC/CON	BP	GO:0031668	cellular response to extracellular stimulus	14/290	281/18870	0.000121569	0.016563817	0.01540411	GABARAP/PMAIP1/FOXO3/STK26/JMY/CDKN1A/NPRL2/KLF10/SESN2/CPEB4/GLRX2/MDM2/CBS/ASNS	14
HS/REC	BP	GO:0071548	response to dexamethasone	3/56	39/18870	0.000209739	0.019819467	0.014802025	CYP1B1/CLDN1/EDN1	3
HS/REC	BP	GO:0045766	positive regulation of angiogenesis	5/56	185/18870	0.000218592	0.019819467	0.014802025	CYP1B1/VEGFC/IL1A/CAMP/F3	5
HS/REC	BP	GO:1904018	positive regulation of vasculature development	5/56	185/18870	0.000218592	0.019819467	0.014802025	CYP1B1/VEGFC/IL1A/CAMP/F3	5
HS/REC	BP	GO:0042542	response to hydrogen peroxide	4/56	101/18870	0.000229336	0.019819467	0.014802025	CRYAB/CYP1B1/EDN1/IL6	4
HS/REC	BP	GO:0001666	response to hypoxia	6/56	298/18870	0.000246719	0.020255601	0.015127749	CRYAB/PLOD2/VEGFC/IL1A/EDN1/EGR1	6
HS/REC	BP	GO:0046545	development of primary female sexual characteristics	4/56	105/18870	0.000266155	0.020810771	0.015542373	INHBB/KITLG/ESR1/FST	4
REC/CON	BP	GO:0000096	sulfur amino acid metabolic process	5/290	34/18870	0.000159928	0.020918574	0.019453971	MTHFR/MRI1/CBS/CTH/SLC7A11	5
HS/CON	BP	GO:0003333	amino acid transmembrane transport	7/218	101/18870	0.000171097	0.021215973	0.019011529	SLC3A2/SLC25A22/SLC6A9/SLC1A4/SLC1A5/SLC7A5/SLC7A11	7
HS/CON	BP	GO:0001666	response to hypoxia	12/218	298/18870	0.000189517	0.02147938	0.019247567	CRYAB/PLOD1/PMAIP1/PLAT/NOTCH1/PPARG/DDR2/DDIT4/RGCC/EDN1/FOS/MDM2	12
HS/CON	BP	GO:0009612	response to mechanical stimulus	10/218	213/18870	0.000192113	0.02147938	0.019247567	SCN9A/TXNIP/LRP11/LTBR/MKKS/DDR2/GADD45A/EDN1/FOS/PSPH	10
HS/CON	BP	GO:0010212	response to ionizing radiation	8/218	138/18870	0.000204697	0.021881422	0.019607834	CRYAB/RAD1/NABP2/CDKN1A/SMPD1/NABP1/GADD45A/MDM2	8
HS/REC	BP	GO:0048732	gland development	7/56	443/18870	0.000322047	0.023697286	0.017698146	INHBB/NRG1/CYP1B1/CLDN1/EDN1/ESR1/IL6	7
HS/REC	BP	GO:0036293	response to decreased oxygen levels	6/56	315/18870	0.000332045	0.023697286	0.017698146	CRYAB/PLOD2/VEGFC/IL1A/EDN1/EGR1	6
HS/REC	BP	GO:0140353	lipid export from cell	3/56	47/18870	0.000365931	0.023697286	0.017698146	SPP1/IL1A/EDN1	3
HS/REC	BP	GO:1901654	response to ketone	5/56	208/18870	0.000375231	0.023697286	0.017698146	SPP1/CYP1B1/CLDN1/EDN1/PTGDR	5
HS/CON	BP	GO:0070778	L-aspartate transmembrane transport	3/218	11/18870	0.000234337	0.024169761	0.021658404	SLC25A22/SLC1A4/SLC1A5	3
HS/REC	BP	GO:0008202	steroid metabolic process	6/56	327/18870	0.000405094	0.024635692	0.018398988	SF1/SPP1/CYP1B1/IL1A/ESR1/EGR1	6
HS/REC	BP	GO:0048545	response to steroid hormone	6/56	330/18870	0.000425185	0.024656446	0.018414488	SPP1/CYP1B1/CLDN1/EDN1/ESR1/IL6	6
HS/REC	BP	GO:0046660	female sex differentiation	4/56	120/18870	0.000442557	0.024656446	0.018414488	INHBB/KITLG/ESR1/FST	4
HS/REC	BP	GO:0010332	response to gamma radiation	3/56	51/18870	0.000466037	0.024656446	0.018414488	CRYAB/IL1A/EGR1	3
HS/REC	BP	GO:0019511	peptidyl-proline hydroxylation	2/56	11/18870	0.000467664	0.024656446	0.018414488	P4HA1/P4HA2	2
HS/REC	BP	GO:0070482	response to oxygen levels	6/56	343/18870	0.000521434	0.024656446	0.018414488	CRYAB/PLOD2/VEGFC/IL1A/EDN1/EGR1	6
HS/REC	BP	GO:0051384	response to glucocorticoid	4/56	127/18870	0.00054829	0.024656446	0.018414488	CYP1B1/CLDN1/EDN1/IL6	4
HS/REC	BP	GO:0033083	regulation of immature T cell proliferation	2/56	12/18870	0.000560128	0.024656446	0.018414488	TNFRSF9/IL1A	2
HS/REC	BP	GO:0033084	regulation of immature T cell proliferation in thymus	2/56	12/18870	0.000560128	0.024656446	0.018414488	TNFRSF9/IL1A	2
HS/REC	BP	GO:0002685	regulation of leukocyte migration	5/56	230/18870	0.000593206	0.024656446	0.018414488	KITLG/VEGFC/IL1A/EDN1/IL6	5
HS/REC	BP	GO:0001541	ovarian follicle development	3/56	56/18870	0.000613863	0.024656446	0.018414488	INHBB/KITLG/ESR1	3
HS/REC	BP	GO:1901342	regulation of vasculature development	6/56	354/18870	0.000615471	0.024656446	0.018414488	CYP1B1/VEGFC/IL1A/CAMP/F3/IL6	6
HS/REC	BP	GO:0033079	immature T cell proliferation	2/56	13/18870	0.000660709	0.024656446	0.018414488	TNFRSF9/IL1A	2
HS/REC	BP	GO:0033080	immature T cell proliferation in thymus	2/56	13/18870	0.000660709	0.024656446	0.018414488	TNFRSF9/IL1A	2
HS/REC	BP	GO:0032308	positive regulation of prostaglandin secretion	2/56	13/18870	0.000660709	0.024656446	0.018414488	IL1A/EDN1	2
HS/REC	BP	GO:0032306	regulation of prostaglandin secretion	2/56	13/18870	0.000660709	0.024656446	0.018414488	IL1A/EDN1	2
HS/REC	BP	GO:0032494	response to peptidoglycan	2/56	13/18870	0.000660709	0.024656446	0.018414488	CAMP/IL6	2
HS/REC	BP	GO:0010574	regulation of vascular endothelial growth factor production	3/56	59/18870	0.0007154	0.024776098	0.018503849	CYP1B1/IL1A/IL6	3
HS/REC	BP	GO:0042445	hormone metabolic process	5/56	241/18870	0.000732504	0.024776098	0.018503849	SPP1/SLC16A10/CYP1B1/ESR1/EGR1	5
HS/REC	BP	GO:0097529	myeloid leukocyte migration	5/56	242/18870	0.000746282	0.024776098	0.018503849	KITLG/VEGFC/IL1A/EDN1/IL6	5
HS/REC	BP	GO:0010212	response to ionizing radiation	4/56	138/18870	0.000749033	0.024776098	0.018503849	CRYAB/RAD1/IL1A/EGR1	4
HS/REC	BP	GO:0030238	male sex determination	2/56	14/18870	0.000769359	0.024776098	0.018503849	SF1/PTGDR	2
HS/REC	BP	GO:0097531	mast cell migration	2/56	14/18870	0.000769359	0.024776098	0.018503849	KITLG/VEGFC	2
REC/CON	BP	GO:0009064	glutamine family amino acid metabolic process	7/290	79/18870	0.000212226	0.026691515	0.024822722	ASRGL1/SLC7A7/PHGDH/MTHFS/ASNS/PYCR1/SLC7A11	7
HS/REC	BP	GO:0010573	vascular endothelial growth factor production	3/56	63/18870	0.00086659	0.027364242	0.020436786	CYP1B1/IL1A/IL6	3
HS/REC	BP	GO:0050921	positive regulation of chemotaxis	4/56	145/18870	0.000900991	0.027396811	0.02046111	VEGFC/EDN1/F3/IL6	4
HS/CON	BP	GO:0034198	cellular response to amino acid starvation	5/218	51/18870	0.000299661	0.027917666	0.025016883	ITFG2/ATF3/CDKN1A/NPRL2/SESN2	5
HS/CON	BP	GO:0071456	cellular response to hypoxia	8/218	147/18870	0.000314649	0.027917666	0.025016883	PMAIP1/NOTCH1/PPARG/DDR2/RGCC/EDN1/FOS/MDM2	8
HS/CON	BP	GO:0036293	response to decreased oxygen levels	12/218	315/18870	0.000314684	0.027917666	0.025016883	CRYAB/PLOD1/PMAIP1/PLAT/NOTCH1/PPARG/DDR2/DDIT4/RGCC/EDN1/FOS/MDM2	12
HS/CON	BP	GO:0010639	negative regulation of organelle organization	13/218	363/18870	0.000323228	0.027917666	0.025016883	CLU/GEN1/RAD1/ODF2L/POT1/RNF41/ATXN7/GHITM/MKKS/TJP1/MAP2/PPARG/ARHGEF2	13
HS/CON	BP	GO:1904707	positive regulation of vascular associated smooth muscle cell proliferation	5/218	52/18870	0.000328455	0.027917666	0.025016883	MIR27B/DDR2/CALCRL/EDN1/MDM2	5
HS/CON	BP	GO:0006865	amino acid transport	8/218	150/18870	0.000360533	0.029337223	0.026288941	SYT4/SLC3A2/SLC25A22/SLC6A9/SLC1A4/SLC1A5/SLC7A5/SLC7A11	8
HS/CON	BP	GO:1990928	response to amino acid starvation	5/218	54/18870	0.000392276	0.029337223	0.026288941	ITFG2/ATF3/CDKN1A/NPRL2/SESN2	5
HS/CON	BP	GO:0072348	sulfur compound transport	5/218	54/18870	0.000392276	0.029337223	0.026288941	SLC3A2/SLC26A3/SLC1A4/SLC7A5/SLC7A11	5
HS/CON	BP	GO:0006563	L-serine metabolic process	3/218	13/18870	0.000399303	0.029337223	0.026288941	PHGDH/PSPH/PSAT1	3
HS/CON	BP	GO:0015824	proline transport	3/218	13/18870	0.000399303	0.029337223	0.026288941	SLC3A2/SLC1A4/SLC7A5	3
HS/REC	BP	GO:0031960	response to corticosteroid	4/56	149/18870	0.00099696	0.029634629	0.022132407	CYP1B1/CLDN1/EDN1/IL6	4
HS/REC	BP	GO:0070301	cellular response to hydrogen peroxide	3/56	67/18870	0.001036675	0.029863515	0.022303349	CYP1B1/EDN1/IL6	3
HS/CON	BP	GO:1901653	cellular response to peptide	13/218	374/18870	0.000429253	0.030242837	0.027100458	GKAP1/TRIB3/PPARG/ARHGEF2/CACNB1/ICAM1/DDR2/EDN1/FOS/MDM2/MIR107/GDF15/PCK2	13
REC/CON	BP	GO:0006749	glutathione metabolic process	6/290	58/18870	0.000258917	0.031357696	0.029162203	ETHE1/GGT7/OPLAH/GLRX2/CHAC1/SLC7A11	6
HS/REC	BP	GO:0032310	prostaglandin secretion	2/56	17/18870	0.00114326	0.032366079	0.024172371	IL1A/EDN1	2
HS/REC	BP	GO:0071479	cellular response to ionizing radiation	3/56	70/18870	0.001177164	0.032761061	0.02446736	CRYAB/RAD1/EGR1	3
HS/REC	BP	GO:0042698	ovulation cycle	3/56	71/18870	0.001226525	0.03356591	0.025068456	CYP1B1/ESR1/EGR1	3
REC/CON	BP	GO:0070482	response to oxygen levels	15/290	343/18870	0.000291023	0.033987324	0.031607719	PMAIP1/PLAT/CPEB1/CDKN1A/ATP6V1G1/CPEB4/AGTRAP/KCNA5/LONP1/DDR2/MTHFR/PRKCE/ADAM15/MDM2/CBS	15
HS/REC	BP	GO:0071371	cellular response to gonadotropin stimulus	2/56	18/18870	0.001283721	0.033997892	0.025391079	CYP1B1/EDN1	2
HS/CON	BP	GO:0036294	cellular response to decreased oxygen levels	8/218	158/18870	0.000510083	0.035139051	0.03148793	PMAIP1/NOTCH1/PPARG/DDR2/RGCC/EDN1/FOS/MDM2	8
REC/CON	BP	GO:0006767	water-soluble vitamin metabolic process	6/290	60/18870	0.000312147	0.035197214	0.032732899	TPK1/ALDH1L2/SLC2A3/MTHFS/PSAT1/BTD	6
HS/REC	BP	GO:0032305	positive regulation of icosanoid secretion	2/56	19/18870	0.001432018	0.037323386	0.0278747	IL1A/EDN1	2
HS/CON	BP	GO:0051091	positive regulation of DNA-binding transcription factor activity	10/218	244/18870	0.000564366	0.03803337	0.034081515	CLU/FLOT1/JMY/PPARG/LAMTOR5/ARHGEF2/CEBPG/DDR2/RGCC/EDN1	10
HS/REC	BP	GO:0042060	wound healing	6/56	423/18870	0.001542694	0.039579749	0.029559848	NRG1/CLDN1/IL1A/EDN1/F3/IL6	6
HS/REC	BP	GO:0032303	regulation of icosanoid secretion	2/56	20/18870	0.001588105	0.040117987	0.029961827	IL1A/EDN1	2
HS/REC	BP	GO:1903532	positive regulation of secretion by cell	5/56	288/18870	0.001617778	0.040248359	0.030059195	INHBB/SPP1/VEGFC/IL1A/EDN1	5
HS/CON	BP	GO:0072537	fibroblast activation	3/218	15/18870	0.000624503	0.041163034	0.036885992	DDR2/RGCC/MDM2	3
HS/CON	BP	GO:0006767	water-soluble vitamin metabolic process	5/218	60/18870	0.000640539	0.041163034	0.036885992	TPK1/ALDH1L2/SLC2A3/MTHFD2/PSAT1	5
HS/REC	BP	GO:1903034	regulation of response to wounding	4/56	173/18870	0.001728865	0.041691034	0.031136646	SPP1/CLDN1/EDN1/F3	4
HS/REC	BP	GO:0044321	response to leptin	2/56	21/18870	0.001751937	0.041691034	0.031136646	INHBB/EDN1	2
HS/REC	BP	GO:0050810	regulation of steroid biosynthetic process	3/56	81/18870	0.001793004	0.04205874	0.031411265	SF1/IL1A/EGR1	3
HS/REC	BP	GO:0014032	neural crest cell development	3/56	82/18870	0.001857202	0.042951075	0.032077699	NRG1/KITLG/EDN1	3
HS/REC	BP	GO:0007530	sex determination	2/56	22/18870	0.001923468	0.043865762	0.032760826	SF1/PTGDR	2
HS/REC	BP	GO:0042129	regulation of T cell proliferation	4/56	182/18870	0.002080024	0.045385358	0.033895726	TNFRSF9/KITLG/IL1A/IL6	4
HS/REC	BP	GO:2000193	positive regulation of fatty acid transport	2/56	23/18870	0.002102652	0.045385358	0.033895726	IL1A/EDN1	2
HS/REC	BP	GO:0060252	positive regulation of glial cell proliferation	2/56	23/18870	0.002102652	0.045385358	0.033895726	VEGFC/IL6	2
HS/REC	BP	GO:1905952	regulation of lipid localization	4/56	183/18870	0.002121861	0.045385358	0.033895726	SPP1/IL1A/EDN1/IL6	4
HS/REC	BP	GO:0032370	positive regulation of lipid transport	3/56	86/18870	0.002128302	0.045385358	0.033895726	SPP1/IL1A/EDN1	3
HS/CON	BP	GO:1903432	regulation of TORC1 signaling	6/218	93/18870	0.000733404	0.04547107	0.040746402	NPC1/ITFG2/STAMBPL1/NPRL2/LAMTOR5/SESN2	6
HS/REC	BP	GO:0046879	hormone secretion	5/56	308/18870	0.002168976	0.045659715	0.034100627	INHBB/SPP1/SLC16A10/EDN1/IL6	5
HS/REC	BP	GO:0051047	positive regulation of secretion	5/56	310/18870	0.002230785	0.046366434	0.034628435	INHBB/SPP1/VEGFC/IL1A/EDN1	5
HS/REC	BP	GO:0048864	stem cell development	3/56	88/18870	0.002272588	0.046644877	0.034836388	NRG1/KITLG/EDN1	3
HS/REC	BP	GO:0050927	positive regulation of positive chemotaxis	2/56	25/18870	0.002483799	0.049736569	0.037145396	VEGFC/F3	2
HS/CON	BP	GO:0046942	carboxylic acid transport	12/218	351/18870	0.0008211	0.049910027	0.044724129	SYT4/SLCO2A1/PPARG/SLC3A2/SLC25A22/SLC26A3/SLC6A9/SLC1A4/SLC1A5/EDN1/SLC7A5/SLC7A11	12
HS/REC	BP	GO:0009914	hormone transport	5/56	319/18870	0.00252497	0.049951821	0.037306155	INHBB/SPP1/SLC16A10/EDN1/IL6	5
