# Supplementary material for: Development and characterization of Hanwoo oviduct epithelial cell line for heat stress study
Source: Anim Biosci. 2026 Sep 1;39(11):250962. doi: 10.5713/ab.250962 (PMC13740644; doi:10.5713/ab.250962)
Supplement: Supplementary file 3 [file ab-250962-Supplementary-11.pdf]

Supplement 11. Oviduct epithelial cells Downregulated DEG - GO Enrichment (BP, MF, CC).

Cluster	ONTOLOGY	ID	Description	GeneRatio	BgRatio	pvalue	p.adjust	qvalue	geneID	Count
HS/CON	BP	GO:0060326	cell chemotaxis	12/160	325/18870	2.2013E-05	0.01733758	0.01555571	CXCL2/SEMA5A/CXCL5/PDGFRA/OXSR1/CD74/EDNRB/CCL2/CXCL8/HGF/GREM1/IL6	12
HS/CON	BP	GO:0071347	cellular response to interleukin-1	7/160	111/18870	4.4838E-05	0.01733758	0.01555571	NFKBIA/AKAP12/KMO/CCL2/CXCL8/HAS2/IL6	7
HS/CON	MF	GO:0005125	cytokine activity	9/162	237/18496	0.00024739	0.02591385	0.02467366	CXCL2/BMP3/CXCL5/IL24/CCL2/CXCL8/GREM1/TNFRSF11B/IL6	9
HS/CON	BP	GO:0043410	positive regulation of MAPK cascade	13/160	474/18870	0.00020667	0.03446767	0.03092525	ADCYAP1/AKAP12/IGF1R/NOX4/PDGFRA/TENM1/TLR9/CD74/DNAJC27/CCL2/HGF/ACTA2/IL6	13
HS/CON	BP	GO:0070372	regulation of ERK1 and ERK2 cascade	10/160	313/18870	0.00034715	0.04881785	0.04380059	ADCYAP1/AKAP12/NOX4/PDGFRA/TLR9/CD74/DNAJC27/CCL2/DUSP6/ACTA2	10
REC/CON	MF	GO:0005125	cytokine activity	14/137	237/18496	2.5697E-09	9.6623E-07	9.0347E-07	SPP1/CXCL2/NRG1/TGFB3/KITLG/BMP3/CXCL5/IL1A/CCL2/CXCL8/BMP4/GREM1/TNFRSF11B/IL6	14
REC/CON	BP	GO:0071347	cellular response to interleukin-1	9/136	111/18870	1.0909E-07	0.00019427	0.00015697	NFKBIA/AKAP12/RORA/KMO/CCL2/CXCL8/CAMP/EGR1/IL6	9
REC/CON	BP	GO:0030198	extracellular matrix organization	12/136	321/18870	3.6892E-06	0.00081988	0.00066247	P4HA1/ADAMTS6/CYP1B1/POSTN/ITGA8/OLFML2A/PDGFRA/HAS3/CCDC80/GREM1/TNFRSF11B/IL6	12
REC/CON	BP	GO:0060326	cell chemotaxis	11/136	325/18870	2.4008E-05	0.00255038	0.00206074	CXCL2/KIT/CXCL5/PDGFRA/SFTPD/EDNRB/CCL2/CXCL8/HGF/GREM1/IL6	11
REC/CON	BP	GO:0001819	positive regulation of cytokine production	13/136	499/18870	6.6343E-05	0.00495838	0.00400644	KIT/AKAP12/RORA/IL18R1/CYP1B1/POSTN/PTGS2/C1QTNF3/CD200/IL1A/HGF/EGR1/IL6	13
REC/CON	BP	GO:0050727	regulation of inflammatory response	12/136	425/18870	5.9742E-05	0.00495838	0.00400644	NFKBIA/RORA/PTGER3/CTSC/CD200R1L/PTGS2/C1QTNF3/EDNRB/CD200/HGF/MIR138-1/IL6	12
REC/CON	BP	GO:0001659	temperature homeostasis	8/136	183/18870	5.4941E-05	0.00495838	0.00400644	PTGER3/IL18R1/LAMA4/ALDH1A1/PTGS2/EDNRB/IL1A/EGR1	8
REC/CON	BP	GO:0070372	regulation of ERK1 and ERK2 cascade	10/136	313/18870	9.1933E-05	0.00553848	0.00447517	SPRY2/NRG1/AKAP12/PDGFRA/IL1A/CCL2/BMP4/DUSP6/ACTA2/MIR138-1	10
REC/CON	BP	GO:0002526	acute inflammatory response	6/136	107/18870	0.00012492	0.00677294	0.00547263	PTGER3/REG3G/PTGS2/EDNRB/IL1A/IL6	6
REC/CON	BP	GO:0043410	positive regulation of MAPK cascade	12/136	474/18870	0.00016682	0.00853285	0.00689466	SPRY2/NRG1/KIT/AKAP12/TGFB3/PDGFRA/IL1A/CCL2/HGF/BMP4/ACTA2/IL6	12
REC/CON	BP	GO:0070374	positive regulation of ERK1 and ERK2 cascade	8/136	218/18870	0.00018517	0.00929643	0.00751164	SPRY2/NRG1/AKAP12/PDGFRA/IL1A/CCL2/BMP4/ACTA2	8
REC/CON	BP	GO:0035234	ectopic germ cell programmed cell death	3/136	18/18870	0.00027603	0.01206985	0.00975261	KIT/KITLG/IL1A	3
REC/CON	BP	GO:0042310	vasoconstriction	5/136	83/18870	0.00033424	0.01352413	0.01092769	HTR1D/PTGS2/EDNRB/SLC8A1/ACTA2	5
REC/CON	BP	GO:0042445	hormone metabolic process	8/136	241/18870	0.00036441	0.01411316	0.01140364	HSD3B1/SPP1/CYP1B1/MME/ALDH1A1/PDGFRA/EDNRB/EGR1	8
REC/CON	BP	GO:0006959	humoral immune response	8/136	258/18870	0.00057223	0.01869048	0.01510217	CXCL2/REG3G/CXCL5/ST6GAL1/CCL2/CXCL8/CAMP/IL6	8
REC/CON	MF	GO:0001664	G protein-coupled receptor binding	9/137	291/18496	0.00032274	0.02022515	0.01891154	CXCL2/CCDC88C/PMCH/CXCL5/NPFFR2/PALM/EDNRB/CCL2/CXCL8	9
REC/CON	BP	GO:0030857	negative regulation of epithelial cell differentiation	4/136	56/18870	0.00070919	0.02184785	0.01765337	SPRY2/REG3G/IL1A/FST	4
REC/CON	BP	GO:0010623	programmed cell death involved in cell development	3/136	25/18870	0.00074986	0.02233913	0.01805033	KIT/KITLG/IL1A	3
REC/CON	BP	GO:0002675	positive regulation of acute inflammatory response	3/136	28/18870	0.00105133	0.02714428	0.02193296	PTGER3/PTGS2/IL6	3
REC/CON	BP	GO:0048568	embryonic organ development	10/136	453/18870	0.00166027	0.03782337	0.03056182	SPRY2/KIT/TGFB3/KITLG/MFAP5/ITGA8/PDGFRA/KRT8/CXCL8/BMP4	10
REC/CON	BP	GO:0042698	ovulation cycle	4/136	71/18870	0.00172633	0.03867829	0.0312526	TGFB3/CYP1B1/PDGFRA/EGR1	4
REC/CON	BP	GO:0042447	hormone catabolic process	2/136	11/18870	0.00271773	0.04989387	0.04031496	SPP1/MME	2
REC/CON	BP	GO:0006953	acute-phase response	6/136	48/18870	1.2006E-06	0.00041345	0.00033407	PTGER3/REG3G/PTGS2/EDNRB/IL1A/IL6	6
REC/CON	BP	GO:0043062	extracellular structure organization	12/136	322/18870	3.8087E-06	0.00081988	0.00066247	P4HA1/ADAMTS6/CYP1B1/POSTN/ITGA8/OLFML2A/PDGFRA/HAS3/CCDC80/GREM1/TNFRSF11B/IL6	12
REC/CON	BP	GO:0022612	gland morphogenesis	5/136	122/18870	0.00190847	0.04181378	0.03378612	TGFB3/SOSTDC1/HGF/BMP4/IL6	5
HS/CON	BP	GO:0009101	glycoprotein biosynthetic process	11/160	299/18870	5.0437E-05	0.01733758	0.01555571	TET1/CHPF2/GXYLT2/ST8SIA4/ST3GAL5/ST6GAL1/EXTL2/HS6ST3/B3GALNT1/GLCE/HS3ST3A1	11
REC/CON	BP	GO:0042116	macrophage activation	5/136	110/18870	0.00120687	0.03029456	0.02447844	RORA/CTSC/PLA2G4A/CD200/IL6	5
REC/CON	BP	GO:1905517	macrophage migration	4/136	61/18870	0.00098004	0.02579513	0.02084283	SFTPD/EDNRB/CD200/CCL2	4
REC/CON	BP	GO:0048762	mesenchymal cell differentiation	11/136	262/18870	3.1592E-06	0.00081988	0.00066247	SLC39A10/SPRY2/NRG1/SEMA3F/TGFB3/KITLG/EDNRB/HGF/BMP4/GREM1/IL6	11
REC/CON	BP	GO:0030856	regulation of epithelial cell differentiation	6/136	171/18870	0.00151012	0.03559048	0.02876488	SPRY2/REG3G/PROM1/IL1A/BMP4/FST	6
REC/CON	BP	GO:0034350	regulation of glial cell apoptotic process	2/136	11/18870	0.00271773	0.04989387	0.04031496	AKAP12/CCL2	2
HS/CON	BP	GO:0032496	response to lipopolysaccharide	12/160	348/18870	4.2899E-05	0.01733758	0.01555571	NFKBIA/CXCL2/AKAP12/HPGD/KMO/CXCL5/IL24/TLR9/EDNRB/CCL2/CXCL8/IL6	12
REC/CON	BP	GO:0032496	response to lipopolysaccharide	12/136	348/18870	8.3823E-06	0.00133672	0.00108009	NFKBIA/CXCL2/AKAP12/KMO/CXCL5/PTGS2/EDNRB/IL1A/CCL2/CXCL8/CAMP/IL6	12
REC/CON	BP	GO:0007584	response to nutrient	6/136	155/18870	0.00091113	0.02495031	0.0201602	SPP1/CYP1B1/POSTN/PTGS2/IL1A/TNFRSF11B	6
REC/CON	BP	GO:0002040	sprouting angiogenesis	6/136	190/18870	0.00256655	0.04974248	0.04019263	PIK3R3/PTGS2/ESM1/BMP4/GREM1/MIR138-1	6
REC/CON	BP	GO:0048864	stem cell development	5/136	88/18870	0.00043822	0.01519694	0.01227934	NRG1/SEMA3F/KITLG/EDNRB/BMP4	5
REC/CON	BP	GO:0048863	stem cell differentiation	8/136	246/18870	0.0004178	0.01497527	0.01210023	NRG1/KIT/SEMA3F/KITLG/PDGFRA/EDNRB/BMP4/GREM1	8
REC/CON	BP	GO:0008202	steroid metabolic process	11/136	327/18870	2.54E-05	0.00255038	0.00206074	HSD3B1/SPP1/KIT/RORA/LIMA1/CYP1B1/PDGFRA/EDNRB/BDH1/IL1A/EGR1	11
REC/CON	BP	GO:0014829	vascular associated smooth muscle contraction	3/136	26/18870	0.00084321	0.02458013	0.01986109	EDNRB/SLC8A1/ACTA2	3
REC/CON	BP	GO:0001667	ameboidal-type cell migration	11/136	497/18870	0.000957	0.02568728	0.02075568	PIK3R3/KIT/AKAP12/SEMA3F/KITLG/CYP1B1/PTGS2/EDNRB/BMP4/GREM1/ACTA2	11
HS/CON	MF	GO:0033218	amide binding	12/162	405/18496	0.00024232	0.02591385	0.02467366	NFKBIA/VDAC2/IGF1R/MME/PLA2G4A/NPFFR2/CD74/EDNRB/BCHE/NLGN1/TMEM158/NLN	12
REC/CON	BP	GO:0061844	antimicrobial humoral immune response mediated by antimicrobial peptide	5/136	83/18870	0.00033424	0.01352413	0.01092769	CXCL2/REG3G/CXCL5/CXCL8/CAMP	5
REC/CON	BP	GO:0019730	antimicrobial humoral response	5/136	133/18870	0.00277904	0.04989387	0.04031496	CXCL2/REG3G/CXCL5/CXCL8/CAMP	5
REC/CON	BP	GO:0008207	C21-steroid hormone metabolic process	3/136	37/18870	0.00237841	0.04811852	0.03888044	HSD3B1/EDNRB/EGR1	3
REC/CON	BP	GO:0043010	camera-type eye development	9/136	344/18870	0.00088226	0.02480729	0.02004464	SPRY2/MEIS1/SLC1A1/CYP1B1/HCN1/CRYGA/PDGFRA/PROM1/BMP4	9
REC/CON	BP	GO:0048593	camera-type eye morphogenesis	5/136	133/18870	0.00277904	0.04989387	0.04031496	MEIS1/SLC1A1/HCN1/PROM1/BMP4	5
REC/CON	BP	GO:0055007	cardiac muscle cell differentiation	6/136	131/18870	0.00037524	0.01412892	0.01141637	MEIS1/NRG1/PDGFRA/SLC8A1/BMP4/GREM1	6
REC/CON	BP	GO:0035051	cardiocyte differentiation	6/136	165/18870	0.00125815	0.03100764	0.02505461	MEIS1/NRG1/PDGFRA/SLC8A1/BMP4/GREM1	6
REC/CON	BP	GO:0061005	cell differentiation involved in kidney development	4/136	57/18870	0.00075849	0.02235065	0.01805964	EDNRB/PROM1/GREM1/ACTA2	4
HS/CON	BP	GO:0071216	cellular response to biotic stimulus	10/160	265/18870	9.0414E-05	0.02071978	0.0185903	NFKBIA/CXCL2/KMO/CXCL5/IL24/TLR9/EDNRB/CCL2/CXCL8/IL6	10
REC/CON	BP	GO:0071216	cellular response to biotic stimulus	10/136	265/18870	2.2675E-05	0.00255038	0.00206074	NFKBIA/CXCL2/KMO/CXCL5/EDNRB/IL1A/CCL2/CXCL8/CAMP/IL6	10
REC/CON	BP	GO:1903351	cellular response to dopamine	4/136	73/18870	0.00191254	0.04181378	0.03378612	SLC1A1/HTR1D/ALK/PALM	4
HS/CON	BP	GO:0071222	cellular response to lipopolysaccharide	10/160	226/18870	2.3663E-05	0.01733758	0.01555571	NFKBIA/CXCL2/KMO/CXCL5/IL24/TLR9/EDNRB/CCL2/CXCL8/IL6	10
REC/CON	BP	GO:0071222	cellular response to lipopolysaccharide	10/136	226/18870	5.6763E-06	0.00096786	0.00078204	NFKBIA/CXCL2/KMO/CXCL5/EDNRB/IL1A/CCL2/CXCL8/CAMP/IL6	10
HS/CON	BP	GO:0071219	cellular response to molecule of bacterial origin	10/160	239/18870	3.8109E-05	0.01733758	0.01555571	NFKBIA/CXCL2/KMO/CXCL5/IL24/TLR9/EDNRB/CCL2/CXCL8/IL6	10
REC/CON	BP	GO:0071219	cellular response to molecule of bacterial origin	10/136	239/18870	9.2783E-06	0.00139741	0.00112913	NFKBIA/CXCL2/KMO/CXCL5/EDNRB/IL1A/CCL2/CXCL8/CAMP/IL6	10
REC/CON	BP	GO:0071356	cellular response to tumor necrosis factor	9/136	238/18870	5.7945E-05	0.00495838	0.00400644	NFKBIA/AKAP12/RORA/CYP1B1/POSTN/KRT8/CCL2/CXCL8/CAMP	9
REC/CON	MF	GO:0008009	chemokine activity	4/137	49/18496	0.00047134	0.0221531	0.02071426	CXCL2/CXCL5/CCL2/CXCL8	4
HS/CON	BP	GO:0006935	chemotaxis	14/160	468/18870	4.6618E-05	0.01733758	0.01555571	CXCL2/SEMA5A/CXCL5/ST6GAL1/PDGFRA/OXSR1/CD74/EDNRB/CCL2/CXCL8/PLAU/HGF/GREM1/IL6	14
REC/CON	BP	GO:0006935	chemotaxis	16/136	468/18870	2.7071E-07	0.00019427	0.00015697	CXCL2/NRG1/KIT/SEMA3F/CXCL5/ST6GAL1/PDGFRA/SFTPD/EDNRB/CCL2/CXCL8/PLAU/HGF/BMP4/GREM1/IL6	16
HS/CON	MF	GO:0045236	CXCR chemokine receptor binding	3/162	18/18496	0.00048861	0.04094536	0.03898579	CXCL2/CXCL5/CXCL8	3
REC/CON	MF	GO:0045236	CXCR chemokine receptor binding	3/137	18/18496	0.00029902	0.02022515	0.01891154	CXCL2/CXCL5/CXCL8	3
REC/CON	MF	GO:0005126	cytokine receptor binding	9/137	273/18496	0.00020146	0.01936339	0.01810575	CXCL2/TGFB3/KITLG/CXCL5/IL1A/CCL2/CXCL8/GREM1/IL6	9
REC/CON	BP	GO:0048263	determination of dorsal identity	2/136	10/18870	0.00223413	0.04553922	0.03679633	SOSTDC1/GREM1	2
REC/CON	BP	GO:0048262	determination of dorsal/ventral asymmetry	2/136	11/18870	0.00271773	0.04989387	0.04031496	SOSTDC1/GREM1	2
REC/CON	BP	GO:0007212	dopamine receptor signaling pathway	3/136	38/18870	0.00256877	0.04974248	0.04019263	SLC1A1/ALK/PALM	3
REC/CON	BP	GO:0035850	epithelial cell differentiation involved in kidney development	4/136	47/18870	0.00036207	0.01411316	0.01140364	EDNRB/PROM1/GREM1/ACTA2	4
REC/CON	BP	GO:0001837	epithelial to mesenchymal transition	7/136	178/18870	0.00031025	0.01317597	0.01064637	SLC39A10/SPRY2/TGFB3/HGF/BMP4/GREM1/IL6	7
REC/CON	BP	GO:0070371	ERK1 and ERK2 cascade	10/136	336/18870	0.00016412	0.00853285	0.00689466	SPRY2/NRG1/AKAP12/PDGFRA/IL1A/CCL2/BMP4/DUSP6/ACTA2/MIR138-1	10
REC/CON	BP	GO:0045229	external encapsulating structure organization	12/136	323/18870	3.9316E-06	0.00081988	0.00066247	P4HA1/ADAMTS6/CYP1B1/POSTN/ITGA8/OLFML2A/PDGFRA/HAS3/CCDC80/GREM1/TNFRSF11B/IL6	12
REC/CON	BP	GO:0001654	eye development	10/136	391/18870	0.00054578	0.01849521	0.01494439	SPRY2/MEIS1/SLC1A1/MFAP5/CYP1B1/HCN1/CRYGA/PDGFRA/PROM1/BMP4	10
REC/CON	BP	GO:0048592	eye morphogenesis	6/136	162/18870	0.00114493	0.02900844	0.02343923	MEIS1/SLC1A1/MFAP5/HCN1/PROM1/BMP4	6
REC/CON	BP	GO:0045444	fat cell differentiation	7/136	248/18870	0.00216061	0.04505699	0.03640668	RORA/PDGFRA/PTGS2/C1QTNF3/FBXO9/MIR138-1/IL6	7
REC/CON	BP	GO:0001660	fever generation	4/136	11/18870	8.191E-07	0.0003701	0.00029904	PTGER3/PTGS2/EDNRB/IL1A	4
HS/CON	BP	GO:0001574	ganglioside biosynthetic process	3/160	17/18870	0.00037279	0.04881785	0.04380059	ST8SIA4/ST3GAL5/ST6GALNAC5	3
REC/CON	BP	GO:0014831	gastro-intestinal system smooth muscle contraction	3/136	12/18870	7.6817E-05	0.00495838	0.00400644	KIT/HTR1D/PTGER3	3
REC/CON	BP	GO:0072109	glomerular mesangium development	3/136	17/18870	0.00023125	0.01044848	0.00844252	BMP4/EGR1/ACTA2	3
REC/CON	BP	GO:0032835	glomerulus development	6/136	69/18870	1.0362E-05	0.00147847	0.00119462	PDGFRA/EDNRB/PROM1/BMP4/EGR1/ACTA2	6
REC/CON	BP	GO:0072012	glomerulus vasculature development	4/136	29/18870	5.3304E-05	0.00495838	0.00400644	PDGFRA/BMP4/EGR1/ACTA2	4
HS/CON	BP	GO:0009100	glycoprotein metabolic process	12/160	370/18870	7.7186E-05	0.01938994	0.01739714	CTSL/TET1/CHPF2/GXYLT2/ST8SIA4/ST3GAL5/ST6GAL1/EXTL2/HS6ST3/B3GALNT1/GLCE/HS3ST3A1	12
REC/CON	MF	GO:0005539	glycosaminoglycan binding	8/137	240/18496	0.00042479	0.0221531	0.02071426	NELL2/VCAN/POSTN/REG3G/ALK/CCDC80/CXCL8/BMP4	8
HS/CON	MF	GO:0016757	glycosyltransferase activity	10/162	268/18496	0.00012932	0.02591385	0.02467366	CHPF2/GXYLT2/ST8SIA4/ST3GAL5/ST6GAL1/EXTL2/MFNG/ST6GALNAC5/HAS2/B3GALNT1	10
REC/CON	MF	GO:0008083	growth factor activity	7/137	162/18496	0.00020599	0.01936339	0.01810575	NRG1/TGFB3/KITLG/BMP3/HGF/BMP4/IL6	7
REC/CON	BP	GO:0031649	heat generation	4/136	17/18870	5.7122E-06	0.00096786	0.00078204	PTGER3/PTGS2/EDNRB/IL1A	4
REC/CON	BP	GO:0002244	hematopoietic progenitor cell differentiation	5/136	133/18870	0.00277904	0.04989387	0.04031496	KIT/KITLG/PDGFRA/BMP4/FST	5
HS/CON	BP	GO:0015012	heparan sulfate proteoglycan biosynthetic process	4/160	33/18870	0.00016817	0.03303337	0.02963836	EXTL2/HS6ST3/GLCE/HS3ST3A1	4
REC/CON	BP	GO:0033079	immature T cell proliferation	3/136	13/18870	9.9336E-05	0.00561042	0.0045333	TNFRSF9/IL1A/BMP4	3
REC/CON	BP	GO:0033080	immature T cell proliferation in thymus	3/136	13/18870	9.9336E-05	0.00561042	0.0045333	TNFRSF9/IL1A/BMP4	3
REC/CON	BP	GO:0070498	interleukin-1-mediated signaling pathway	3/136	32/18870	0.00155864	0.03642642	0.02943306	NFKBIA/EGR1/IL6	3
REC/CON	BP	GO:0001822	kidney development	10/136	314/18870	9.4383E-05	0.00556243	0.00449452	ADAMTS6/MME/ITGA8/PDGFRA/EDNRB/PROM1/BMP4/EGR1/GREM1/ACTA2	10
REC/CON	BP	GO:0061440	kidney vasculature development	4/136	31/18870	6.9832E-05	0.00495838	0.00400644	PDGFRA/BMP4/EGR1/ACTA2	4
REC/CON	BP	GO:0071887	leukocyte apoptotic process	5/136	121/18870	0.00184074	0.04090367	0.03305074	SLC39A10/KITLG/ST6GAL1/BMP4/IL6	5
HS/CON	BP	GO:0030595	leukocyte chemotaxis	9/160	240/18870	0.00021378	0.03446767	0.03092525	CXCL2/CXCL5/OXSR1/CD74/EDNRB/CCL2/CXCL8/GREM1/IL6	9
REC/CON	BP	GO:0030595	leukocyte chemotaxis	9/136	240/18870	6.1806E-05	0.00495838	0.00400644	CXCL2/KIT/CXCL5/SFTPD/EDNRB/CCL2/CXCL8/GREM1/IL6	9
REC/CON	BP	GO:0050900	leukocyte migration	12/136	396/18870	3.0183E-05	0.00292232	0.00236127	CXCL2/KIT/KITLG/CXCL5/SFTPD/EDNRB/CD200/IL1A/CCL2/CXCL8/GREM1/IL6	12
REC/CON	BP	GO:0070661	leukocyte proliferation	10/136	350/18870	0.000228	0.01044848	0.00844252	TNFRSF9/SLC39A10/KIT/KITLG/ST6GAL1/SFTPD/IL1A/BMP4/GREM1/IL6	10
REC/CON	BP	GO:0140353	lipid export from cell	4/136	47/18870	0.00036207	0.01411316	0.01140364	SPP1/PLA2G4A/PTGS2/IL1A	4
REC/CON	BP	GO:0030324	lung development	6/136	188/18870	0.00243479	0.04853463	0.03921667	SPRY2/TGFB3/MME/PDGFRA/SFTPD/BMP4	6
REC/CON	BP	GO:0035633	maintenance of blood-brain barrier	3/136	38/18870	0.00256877	0.04974248	0.04019263	SLC1A1/PTGS2/IL6	3
REC/CON	BP	GO:0030318	melanocyte differentiation	3/136	25/18870	0.00074986	0.02233913	0.01805033	KIT/KITLG/EDNRB	3
REC/CON	BP	GO:0090497	mesenchymal cell migration	4/136	61/18870	0.00098004	0.02579513	0.02084283	SEMA3F/KITLG/EDNRB/BMP4	4
REC/CON	BP	GO:0060485	mesenchyme development	12/136	327/18870	4.4588E-06	0.00086342	0.00069765	SLC39A10/SPRY2/NRG1/SEMA3F/TGFB3/KITLG/EDNRB/HGF/BMP4/GREM1/ACTA2/IL6	12
REC/CON	BP	GO:0072210	metanephric nephron development	4/136	41/18870	0.00021256	0.01029016	0.00831459	PDGFRA/BMP4/EGR1/GREM1	4
REC/CON	BP	GO:0001656	metanephros development	5/136	94/18870	0.00059323	0.0189204	0.01528795	ITGA8/PDGFRA/BMP4/EGR1/GREM1	5
REC/CON	BP	GO:0032943	mononuclear cell proliferation	8/136	314/18870	0.00200993	0.04321737	0.03492025	TNFRSF9/SLC39A10/KITLG/ST6GAL1/SFTPD/IL1A/BMP4/IL6	8
REC/CON	BP	GO:0042692	muscle cell differentiation	11/136	419/18870	0.00023027	0.01044848	0.00844252	MEIS1/NRG1/KIT/RORA/ITGA8/PDGFRA/KRT8/EDNRB/SLC8A1/BMP4/GREM1	11
REC/CON	BP	GO:0002274	myeloid leukocyte activation	9/136	241/18870	6.3817E-05	0.00495838	0.00400644	KIT/RORA/CTSC/PLA2G4A/CD200/CXCL8/CAMP/PTGDR/IL6	9
REC/CON	BP	GO:0097529	myeloid leukocyte migration	12/136	242/18870	1.8793E-07	0.00019427	0.00015697	CXCL2/KIT/KITLG/CXCL5/SFTPD/EDNRB/CD200/IL1A/CCL2/CXCL8/GREM1/IL6	12
REC/CON	BP	GO:0046851	negative regulation of bone remodeling	3/136	18/18870	0.00027603	0.01206985	0.00975261	GREM1/TNFRSF11B/IL6	3
REC/CON	BP	GO:0050922	negative regulation of chemotaxis	4/136	70/18870	0.00163812	0.03782337	0.03056182	SEMA3F/ST6GAL1/CCL2/GREM1	4
REC/CON	BP	GO:2000107	negative regulation of leukocyte apoptotic process	4/136	59/18870	0.00086427	0.02466349	0.01992845	SLC39A10/KITLG/ST6GAL1/BMP4	4
REC/CON	BP	GO:0071676	negative regulation of mononuclear cell migration	3/136	25/18870	0.00074986	0.02233913	0.01805033	CD200/CCL2/GREM1	3
REC/CON	BP	GO:0032102	negative regulation of response to external stimulus	12/136	481/18870	0.00019098	0.00941375	0.00760644	SPP1/SEMA3F/RORA/ST6GAL1/PDGFRA/C1QTNF3/CD200/CCL2/PLAU/HGF/GREM1/MIR138-1	12
REC/CON	BP	GO:0034104	negative regulation of tissue remodeling	3/136	21/18870	0.00044285	0.01519694	0.01227934	GREM1/TNFRSF11B/IL6	3
REC/CON	BP	GO:0072006	nephron development	7/136	156/18870	0.00013805	0.00733832	0.00592947	PDGFRA/EDNRB/PROM1/BMP4/EGR1/GREM1/ACTA2	7
REC/CON	BP	GO:0014032	neural crest cell development	5/136	82/18870	0.00031591	0.01317597	0.01064637	NRG1/SEMA3F/KITLG/EDNRB/BMP4	5
REC/CON	BP	GO:0014033	neural crest cell differentiation	5/136	95/18870	0.0006226	0.01962641	0.01585842	NRG1/SEMA3F/KITLG/EDNRB/BMP4	5
REC/CON	BP	GO:0001755	neural crest cell migration	4/136	59/18870	0.00086427	0.02466349	0.01992845	SEMA3F/KITLG/EDNRB/BMP4	4
REC/CON	BP	GO:0150076	neuroinflammatory response	5/136	82/18870	0.00031591	0.01317597	0.01064637	CTSC/CD200R1L/PTGS2/CD200/IL6	5
REC/CON	BP	GO:1990266	neutrophil migration	5/136	129/18870	0.00243477	0.04853463	0.03921667	CXCL2/CXCL5/IL1A/CCL2/CXCL8	5
REC/CON	BP	GO:0042476	odontogenesis	6/136	132/18870	0.00039081	0.01432259	0.01157286	TGFB3/SOSTDC1/PDGFRA/BMP4/TNFRSF11B/FST	6
REC/CON	BP	GO:0042475	odontogenesis of dentin-containing tooth	5/136	93/18870	0.0005649	0.01867621	0.01509064	SOSTDC1/PDGFRA/BMP4/TNFRSF11B/FST	5
REC/CON	BP	GO:0120254	olefinic compound metabolic process	6/136	162/18870	0.00114493	0.02900844	0.02343923	CYP1B1/PLA2G4A/ALDH1A1/PTGS2/EDNRB/EGR1	6
REC/CON	BP	GO:0001503	ossification	10/136	444/18870	0.00143146	0.03434245	0.02774918	SPP1/VCAN/BMP3/PTGS2/SLC8A1/HGF/BMP4/GREM1/MIR138-1/IL6	10
REC/CON	BP	GO:0001649	osteoblast differentiation	8/136	257/18870	0.00055782	0.0186696	0.0150853	SPP1/VCAN/BMP3/HGF/BMP4/GREM1/MIR138-1/IL6	8
HS/CON	MF	GO:0042277	peptide binding	10/162	325/18496	0.00059964	0.04187496	0.03987089	NFKBIA/IGF1R/MME/NPFFR2/CD74/EDNRB/BCHE/NLGN1/TMEM158/NLN	10
REC/CON	BP	GO:0018212	peptidyl-tyrosine modification	8/136	278/18870	0.00092989	0.02520943	0.02036958	KIT/KITLG/ALK/PDGFRA/HGF/GREM1/MIR138-1/IL6	8
REC/CON	BP	GO:0018108	peptidyl-tyrosine phosphorylation	8/136	276/18870	0.00088761	0.02480729	0.02004464	KIT/KITLG/ALK/PDGFRA/HGF/GREM1/MIR138-1/IL6	8
REC/CON	BP	GO:1904238	pericyte cell differentiation	2/136	11/18870	0.00271773	0.04989387	0.04031496	BMP4/ACTA2	2
REC/CON	BP	GO:0014821	phasic smooth muscle contraction	4/136	24/18870	2.4524E-05	0.00255038	0.00206074	KIT/HTR1D/PTGER3/EDNRB	4
REC/CON	BP	GO:0050931	pigment cell differentiation	3/136	36/18870	0.00219706	0.04546747	0.03673836	KIT/KITLG/EDNRB	3
HS/CON	BP	GO:0032722	positive regulation of chemokine production	5/160	72/18870	0.00036439	0.04881785	0.04380059	ADCYAP1/LPL/TLR9/CD74/IL6	5
REC/CON	BP	GO:0070665	positive regulation of leukocyte proliferation	6/136	166/18870	0.00129773	0.03169501	0.02561002	SLC39A10/KIT/KITLG/ST6GAL1/IL1A/IL6	6
REC/CON	BP	GO:0050806	positive regulation of synaptic transmission	6/136	174/18870	0.00164969	0.03782337	0.03056182	CA2/SLC1A1/KMO/MME/PTGS2/CCL2	6
REC/CON	BP	GO:0051968	positive regulation of synaptic transmission, glutamatergic	3/136	35/18870	0.00202457	0.04321737	0.03492025	KMO/PTGS2/CCL2	3
REC/CON	BP	GO:0010575	positive regulation of vascular endothelial growth factor production	5/136	30/18870	2.2268E-06	0.00067077	0.00054199	RORA/CYP1B1/PTGS2/IL1A/IL6	5
REC/CON	BP	GO:0032310	prostaglandin secretion	3/136	17/18870	0.00023125	0.01044848	0.00844252	PLA2G4A/PTGS2/IL1A	3
REC/CON	BP	GO:0015732	prostaglandin transport	3/136	28/18870	0.00105133	0.02714428	0.02193296	PLA2G4A/PTGS2/IL1A	3
HS/CON	BP	GO:0030166	proteoglycan biosynthetic process	5/160	65/18870	0.00022561	0.03446767	0.03092525	CHPF2/EXTL2/HS6ST3/GLCE/HS3ST3A1	5
REC/CON	BP	GO:0002673	regulation of acute inflammatory response	4/136	49/18870	0.00042534	0.01497527	0.01210023	PTGER3/PTGS2/EDNRB/IL6	4
REC/CON	BP	GO:0046850	regulation of bone remodeling	4/136	49/18870	0.00042534	0.01497527	0.01210023	SPP1/GREM1/TNFRSF11B/IL6	4
HS/CON	BP	GO:0050920	regulation of chemotaxis	9/160	232/18870	0.00016598	0.03303337	0.02963836	SEMA5A/ST6GAL1/PDGFRA/OXSR1/CD74/CCL2/CXCL8/GREM1/IL6	9
REC/CON	BP	GO:0050920	regulation of chemotaxis	7/136	232/18870	0.00148024	0.03520125	0.02844311	SEMA3F/ST6GAL1/PDGFRA/CCL2/CXCL8/GREM1/IL6	7
REC/CON	BP	GO:0032965	regulation of collagen biosynthetic process	3/136	38/18870	0.00256877	0.04974248	0.04019263	TGFB3/BMP4/IL6	3
REC/CON	BP	GO:0060159	regulation of dopamine receptor signaling pathway	2/136	11/18870	0.00271773	0.04989387	0.04031496	ALK/PALM	2
REC/CON	BP	GO:0010717	regulation of epithelial to mesenchymal transition	5/136	111/18870	0.00125658	0.03100764	0.02505461	SPRY2/TGFB3/BMP4/GREM1/IL6	5
REC/CON	BP	GO:0072124	regulation of glomerular mesangial cell proliferation	2/136	11/18870	0.00271773	0.04989387	0.04031496	BMP4/EGR1	2
REC/CON	BP	GO:0031650	regulation of heat generation	3/136	12/18870	7.6817E-05	0.00495838	0.00400644	PTGER3/PTGS2/EDNRB	3
REC/CON	BP	GO:0033083	regulation of immature T cell proliferation	3/136	12/18870	7.6817E-05	0.00495838	0.00400644	TNFRSF9/IL1A/BMP4	3
REC/CON	BP	GO:0033084	regulation of immature T cell proliferation in thymus	3/136	12/18870	7.6817E-05	0.00495838	0.00400644	TNFRSF9/IL1A/BMP4	3
REC/CON	BP	GO:0002685	regulation of leukocyte migration	7/136	230/18870	0.00140865	0.03409691	0.02755079	KITLG/CD200/IL1A/CCL2/CXCL8/GREM1/IL6	7
REC/CON	BP	GO:0070663	regulation of leukocyte proliferation	10/136	267/18870	2.4182E-05	0.00255038	0.00206074	TNFRSF9/SLC39A10/KIT/KITLG/ST6GAL1/SFTPD/IL1A/BMP4/GREM1/IL6	10
REC/CON	BP	GO:0043550	regulation of lipid kinase activity	3/136	39/18870	0.00276829	0.04989387	0.04031496	PIK3R3/KIT/PDGFRA	3
REC/CON	BP	GO:0050670	regulation of lymphocyte proliferation	7/136	238/18870	0.00171219	0.03867829	0.0312526	TNFRSF9/SLC39A10/KITLG/SFTPD/IL1A/BMP4/IL6	7
REC/CON	BP	GO:0043030	regulation of macrophage activation	5/136	62/18870	8.4059E-05	0.00529964	0.00428218	RORA/CTSC/PLA2G4A/CD200/IL6	5
REC/CON	BP	GO:0032944	regulation of mononuclear cell proliferation	8/136	242/18870	0.00037462	0.01412892	0.01141637	TNFRSF9/SLC39A10/KITLG/ST6GAL1/SFTPD/IL1A/BMP4/IL6	8
REC/CON	BP	GO:0150077	regulation of neuroinflammatory response	5/136	41/18870	1.0989E-05	0.00148952	0.00120355	CTSC/CD200R1L/PTGS2/CD200/IL6	5
REC/CON	BP	GO:0042129	regulation of T cell proliferation	6/136	182/18870	0.00206994	0.04350082	0.03514927	TNFRSF9/KITLG/SFTPD/IL1A/BMP4/IL6	6
REC/CON	BP	GO:0010574	regulation of vascular endothelial growth factor production	5/136	59/18870	6.6168E-05	0.00495838	0.00400644	RORA/CYP1B1/PTGS2/IL1A/IL6	5
REC/CON	BP	GO:0072001	renal system development	10/136	324/18870	0.00012208	0.00675426	0.00545753	ADAMTS6/MME/ITGA8/PDGFRA/EDNRB/PROM1/BMP4/EGR1/GREM1/ACTA2	10
REC/CON	BP	GO:0061437	renal system vasculature development	4/136	31/18870	6.9832E-05	0.00495838	0.00400644	PDGFRA/BMP4/EGR1/ACTA2	4
REC/CON	BP	GO:0030323	respiratory tube development	6/136	192/18870	0.0027036	0.04989387	0.04031496	SPRY2/TGFB3/MME/PDGFRA/SFTPD/BMP4	6
REC/CON	BP	GO:0036293	response to decreased oxygen levels	8/136	315/18870	0.00205003	0.04341905	0.03508321	SLC1A1/TGFB3/RORA/POSTN/PTGS2/IL1A/PLAU/EGR1	8
REC/CON	BP	GO:1903350	response to dopamine	4/136	74/18870	0.00201067	0.04321737	0.03492025	SLC1A1/HTR1D/ALK/PALM	4
HS/CON	BP	GO:0070555	response to interleukin-1	7/160	140/18870	0.00019321	0.03446767	0.03092525	NFKBIA/AKAP12/KMO/CCL2/CXCL8/HAS2/IL6	7
REC/CON	BP	GO:0070555	response to interleukin-1	9/136	140/18870	7.9292E-07	0.0003701	0.00029904	NFKBIA/AKAP12/RORA/KMO/CCL2/CXCL8/CAMP/EGR1/IL6	9
HS/CON	BP	GO:0002237	response to molecule of bacterial origin	12/160	369/18870	7.5227E-05	0.01938994	0.01739714	NFKBIA/CXCL2/AKAP12/HPGD/KMO/CXCL5/IL24/TLR9/EDNRB/CCL2/CXCL8/IL6	12
REC/CON	BP	GO:0002237	response to molecule of bacterial origin	12/136	369/18870	1.5062E-05	0.00194437	0.00157108	NFKBIA/CXCL2/AKAP12/KMO/CXCL5/PTGS2/EDNRB/IL1A/CCL2/CXCL8/CAMP/IL6	12
REC/CON	BP	GO:0034612	response to tumor necrosis factor	10/136	259/18870	1.8628E-05	0.00229548	0.00185478	NFKBIA/AKAP12/RORA/CYP1B1/POSTN/PTGS2/KRT8/CCL2/CXCL8/CAMP	10
REC/CON	BP	GO:0090596	sensory organ morphogenesis	8/136	277/18870	0.00090855	0.02495031	0.0201602	SPRY2/MEIS1/SLC1A1/MFAP5/HCN1/ITGA8/PROM1/BMP4	8
REC/CON	BP	GO:0048880	sensory system development	10/136	401/18870	0.00066321	0.02066624	0.01669861	SPRY2/MEIS1/SLC1A1/MFAP5/CYP1B1/HCN1/CRYGA/PDGFRA/PROM1/BMP4	10
HS/CON	MF	GO:0008373	sialyltransferase activity	4/162	21/18496	3.0214E-05	0.01265978	0.0120539	ST8SIA4/ST3GAL5/ST6GAL1/ST6GALNAC5	4
REC/CON	MF	GO:0008373	sialyltransferase activity	4/137	21/18496	1.5633E-05	0.0029391	0.0027482	ST3GAL6/ST8SIA4/ST6GAL1/ST6GALNAC5	4
REC/CON	BP	GO:0051145	smooth muscle cell differentiation	4/136	76/18870	0.00221728	0.04553815	0.03679547	KIT/ITGA8/EDNRB/BMP4	4
REC/CON	BP	GO:0006939	smooth muscle contraction	8/136	109/18870	1.2201E-06	0.00041345	0.00033407	CNN1/KIT/HTR1D/PTGER3/PTGS2/EDNRB/SLC8A1/ACTA2	8
REC/CON	MF	GO:1901681	sulfur compound binding	8/137	275/18496	0.00103313	0.04316204	0.04035868	NELL2/POSTN/RRM2/ALK/CCDC80/CXCL8/BMP4/FST	8
REC/CON	BP	GO:0030217	T cell differentiation	9/136	307/18870	0.00039095	0.01432259	0.01157286	TNFRSF9/PIK3R3/KIT/RORA/IL18R1/IL1A/BMP4/EGR1/IL6	9
HS/CON	BP	GO:0042330	taxis	14/160	470/18870	4.8812E-05	0.01733758	0.01555571	CXCL2/SEMA5A/CXCL5/ST6GAL1/PDGFRA/OXSR1/CD74/EDNRB/CCL2/CXCL8/PLAU/HGF/GREM1/IL6	14
REC/CON	BP	GO:0042330	taxis	16/136	470/18870	2.8664E-07	0.00019427	0.00015697	CXCL2/NRG1/KIT/SEMA3F/CXCL5/ST6GAL1/PDGFRA/SFTPD/EDNRB/CCL2/CXCL8/PLAU/HGF/BMP4/GREM1/IL6	16
HS/CON	BP	GO:0006636	unsaturated fatty acid biosynthetic process	5/160	52/18870	7.756E-05	0.01938994	0.01739714	PLA2G4A/FADS2/CD74/ELOVL6/SCD	5
REC/CON	BP	GO:0010573	vascular endothelial growth factor production	5/136	63/18870	9.0781E-05	0.00553848	0.00447517	RORA/CYP1B1/PTGS2/IL1A/IL6	5
REC/CON	BP	GO:0150063	visual system development	10/136	395/18870	0.00059049	0.0189204	0.01528795	SPRY2/MEIS1/SLC1A1/MFAP5/CYP1B1/HCN1/CRYGA/PDGFRA/PROM1/BMP4	10
