# Supplementary material for: Development and characterization of Hanwoo oviduct epithelial cell line for heat stress study
Source: Anim Biosci. 2026 Sep 1;39(11):250962. doi: 10.5713/ab.250962 (PMC13740644; doi:10.5713/ab.250962)
Supplement: Supplementary file 4 [file ab-250962-Supplementary-12.pdf]

Supplement 12. Oviduct epithelial cells Upregulated DEP - GO Enrichment (BP, MF, CC).

Cluster	ONTOLOGY	ID	Description	GeneRatio	BgRatio	pvalue	p.adjust	qvalue	geneID	Count
REC/CON	BP	GO:0006953	acute-phase response	4/123	48/18870	0.000267958	0.047942138	0.045905423	SERPINA1/ORM1/SERPINF2/CEBPB	4
REC/CON	CC	GO:0045178	basal part of cell	7/125	301/19886	0.002961516	0.041461228	0.038187973	KRT14/DSP/SLC3A2/SLC12A6/CNNM4/TACSTD2/DSTYK	7
REC/CON	CC	GO:0062023	collagen-containing extracellular matrix	13/125	429/19886	3.13708E-06	0.000117117	0.000107871	APOA1/SERPINA1/KNG1/ORM1/KRT1/SERPINF2/CRELD1/FBLN2/S100A10/TGFB1/ITIH1/COL10A1/SDC2	13
REC/CON	CC	GO:0060205	cytoplasmic vesicle lumen	10/125	325/19886	3.99302E-05	0.000834451	0.000768573	ALB/APOA1/HSPA6/SERPINA1/KNG1/ORM1/SERPINF2/GCA/S100A11/TGFB1	10
HS/CON	CC	GO:0060205	cytoplasmic vesicle lumen	7/82	325/19886	0.000388828	0.018931432	0.016676002	PKM/HSPA6/COMMD9/GCA/S100A11/CSTB/MIF	7
REC/CON	MF	GO:0004866	endopeptidase inhibitor activity	7/124	168/18496	0.000138983	0.016143451	0.014874852	SERPINA1/KNG1/SERPINF2/FETUB/CST6/ITIH1/RECK	7
REC/CON	CC	GO:0005788	endoplasmic reticulum lumen	8/125	313/19886	0.000824162	0.014200945	0.013079817	ALB/AFP/APOA1/SERPINA1/KNG1/ARSI/COL10A1/SDC2	8
HS/REC	CC	GO:0045111	intermediate filament cytoskeleton	13/92	250/19886	1.32731E-10	9.20269E-09	8.66245E-09	KRT5/KRT14/DSP/KRT3/KRT17/KRT1/KRT10/KRT75/KRT2/KRT19/KRT24/KRT76/KRT73	13
REC/CON	CC	GO:0045111	intermediate filament cytoskeleton	14/125	250/19886	6.07364E-10	4.53498E-08	4.17696E-08	KRT5/KRT14/DSP/KRT3/KRT17/KRT1/KRT10/KRT75/SLC1A4/KRT2/KRT19/KRT24/KRT76/KRT73	14
HS/REC	BP	GO:0045104	intermediate filament cytoskeleton organization	13/90	94/18870	5.9138E-16	4.39778E-13	4.15267E-13	KRT5/KRT14/DSP/KRT3/KRT17/KRT1/KRT10/KRT75/KRT2/KRT19/KRT24/KRT76/KRT73	13
REC/CON	BP	GO:0045104	intermediate filament cytoskeleton organization	13/123	94/18870	3.87207E-14	3.19301E-11	3.05737E-11	KRT5/KRT14/DSP/KRT3/KRT17/KRT1/KRT10/KRT75/KRT2/KRT19/KRT24/KRT76/KRT73	13
HS/REC	BP	GO:0030216	keratinocyte differentiation	11/90	177/18870	7.92643E-10	3.83045E-07	3.61696E-07	KRT5/KRT14/DSP/KRT3/KRT17/KRT1/KRT10/KRT75/KRT2/KRT76/KRT73	11
REC/CON	BP	GO:0030216	keratinocyte differentiation	11/123	177/18870	2.24795E-08	1.20658E-05	1.15533E-05	KRT5/KRT14/DSP/KRT3/KRT17/KRT1/KRT10/KRT75/KRT2/KRT76/KRT73	11
HS/CON	BP	GO:0016239	positive regulation of macroautophagy	4/83	75/18870	0.000333538	0.043999166	0.039965995	BAG3/HSPB8/AMBRA1/BNIP3	4
HS/CON	BP	GO:0010822	positive regulation of mitochondrion organization	4/83	75/18870	0.000333538	0.043999166	0.039965995	AMBRA1/BNIP3/MLLT11/BID	4
HS/CON	BP	GO:0090200	positive regulation of release of cytochrome c from mitochondria	3/83	25/18870	0.000175987	0.034823374	0.031631299	BNIP3/MLLT11/BID	3
HS/CON	BP	GO:0042026	protein refolding	5/83	27/18870	1.09029E-07	8.62963E-05	7.8386E-05	HSPB1/HSPA6/HSPA1L/CRYAB/DNAJB2	5
REC/CON	BP	GO:0042026	protein refolding	4/123	27/18870	2.68609E-05	0.006407828	0.006135606	HSPA6/CRYAB/DNAJB2/BAG5	4
HS/CON	BP	GO:0009408	response to heat	5/83	103/18870	9.10292E-05	0.024016535	0.021815066	BAG3/HSPB1/CHORDC1/HSPA6/CRYAB	5
HS/CON	BP	GO:0006986	response to unfolded protein	7/83	137/18870	2.39042E-06	0.000946009	0.000859294	HSPH1/BAG3/HSPB1/HSPA6/HSPA1L/DNAJB2/HSPB8	7
HS/REC	BP	GO:0043588	skin development	11/90	317/18870	3.23229E-07	7.81003E-05	7.37474E-05	KRT5/KRT14/DSP/KRT3/KRT17/KRT1/KRT10/KRT75/KRT2/KRT76/KRT73	11
REC/CON	BP	GO:0043588	skin development	11/123	317/18870	7.32563E-06	0.002210437	0.002116531	KRT5/KRT14/DSP/KRT3/KRT17/KRT1/KRT10/KRT75/KRT2/KRT76/KRT73	11
REC/CON	CC	GO:0031983	vesicle lumen	10/125	326/19886	4.09775E-05	0.000834451	0.000768573	ALB/APOA1/HSPA6/SERPINA1/KNG1/ORM1/SERPINF2/GCA/S100A11/TGFB1	10
HS/CON	CC	GO:0031983	vesicle lumen	7/82	326/19886	0.000396055	0.018931432	0.016676002	PKM/HSPA6/COMMD9/GCA/S100A11/CSTB/MIF	7
HS/REC	BP	GO:0008544	epidermis development	12/90	385/18870	2.86841E-07	7.81003E-05	7.37474E-05	KRT5/KRT14/DSP/KRT3/KRT17/KRT1/FABP5/KRT10/KRT75/KRT2/KRT76/KRT73	12
REC/CON	BP	GO:0008544	epidermis development	12/123	385/18870	8.23637E-06	0.002210437	0.002116531	KRT5/KRT14/DSP/KRT3/KRT17/KRT1/KRT10/KRT75/KRT2/KRT76/CST6/KRT73	12
HS/CON	MF	GO:0031072	heat shock protein binding	6/82	128/18496	2.22384E-05	0.001890264	0.001607407	CHORDC1/HSPA6/HSPA1L/BAG2/DNAJB2/APOA2	6
HS/REC	BP	GO:0007599	hemostasis	7/90	231/18870	0.000118687	0.01529481	0.014442345	PLG/KRT1/GNA13/JMJD1C/DTNBP1/PRKG1/SLC7A11	7
HS/CON	MF	GO:0000774	adenyl-nucleotide exchange factor activity	4/82	15/18496	4.71887E-07	0.000120331	0.000102325	HSPH1/BAG3/BAG2/BAG4	4
HS/CON	MF	GO:0060590	ATPase regulator activity	5/82	49/18496	2.47703E-06	0.000315822	0.000268563	HSPH1/BAG3/BAG2/DNAJB2/BAG4	5
HS/CON	MF	GO:0140662	ATP-dependent protein folding chaperone	3/82	40/18496	0.000737187	0.020886978	0.017761475	HSPH1/HSPA6/HSPA1L	3
HS/REC	BP	GO:0007596	blood coagulation	7/90	224/18870	9.80105E-05	0.01529481	0.014442345	PLG/KRT1/GNA13/JMJD1C/DTNBP1/PRKG1/SLC7A11	7
REC/CON	CC	GO:0072562	blood microparticle	11/125	144/19886	1.78904E-09	1.00186E-07	9.22767E-08	ALB/APOA1/GC/HSPA6/KNG1/ORM1/KRT1/SERPINF2/TGFB1/ITIH1/CD5L	11
HS/REC	CC	GO:0072562	blood microparticle	9/92	144/19886	2.22023E-08	9.23614E-07	8.69394E-07	ALB/PLG/GC/ORM1/KRT1/AMBP/TGFB1/ITIH1/CD5L	9
HS/CON	MF	GO:0048306	calcium-dependent protein binding	4/82	79/18496	0.000418762	0.015795028	0.013431479	CALM1/S100A11/S100A2/S100A10	4
HS/CON	BP	GO:0051085	chaperone cofactor-dependent protein refolding	3/83	32/18870	0.000371187	0.045199113	0.041055949	HSPH1/HSPA6/HSPA1L	3
HS/CON	BP	GO:0061077	chaperone-mediated protein folding	6/83	75/18870	9.52681E-07	0.000502698	0.000456619	HSPH1/HSPB1/CHORDC1/HSPA6/HSPA1L/DNAJB2	6
HS/REC	BP	GO:0050817	coagulation	7/90	229/18870	0.000112448	0.01529481	0.014442345	PLG/KRT1/GNA13/JMJD1C/DTNBP1/PRKG1/SLC7A11	7
REC/CON	CC	GO:0001533	cornified envelope	8/125	60/19886	3.78296E-09	1.69477E-07	1.56097E-07	KRT14/DSP/KRT17/KRT1/KRT10/KRT75/KRT2/CST6	8
HS/REC	CC	GO:0001533	cornified envelope	7/92	60/19886	1.13806E-08	5.91791E-07	5.5705E-07	KRT14/DSP/KRT17/KRT1/KRT10/KRT75/KRT2	7
REC/CON	MF	GO:0061135	endopeptidase regulator activity	7/124	184/18496	0.000243278	0.017564705	0.01618442	SERPINA1/KNG1/SERPINF2/FETUB/CST6/ITIH1/RECK	7
HS/REC	BP	GO:0009913	epidermal cell differentiation	11/90	245/18870	2.38179E-08	7.67333E-06	7.24565E-06	KRT5/KRT14/DSP/KRT3/KRT17/KRT1/KRT10/KRT75/KRT2/KRT76/KRT73	11
REC/CON	BP	GO:0009913	epidermal cell differentiation	11/123	245/18870	6.05693E-07	0.000216737	0.00020753	KRT5/KRT14/DSP/KRT3/KRT17/KRT1/KRT10/KRT75/KRT2/KRT76/KRT73	11
HS/CON	CC	GO:0101002	ficolin-1-rich granule	6/82	185/19886	0.000116481	0.013919457	0.012261138	PKM/HSPA6/COMMD9/CSTB/COMMD3/MIF	6
HS/CON	CC	GO:1904813	ficolin-1-rich granule lumen	6/82	124/19886	1.23758E-05	0.002957816	0.002605431	PKM/HSPA6/COMMD9/CSTB/COMMD3/MIF	6
HS/REC	CC	GO:0005882	intermediate filament	13/92	214/19886	1.90387E-11	2.55728E-09	2.40715E-09	KRT5/KRT14/DSP/KRT3/KRT17/KRT1/KRT10/KRT75/KRT2/KRT19/KRT24/KRT76/KRT73	13
REC/CON	CC	GO:0005882	intermediate filament	14/125	214/19886	7.80758E-11	1.7489E-08	1.61083E-08	KRT5/KRT14/DSP/KRT3/KRT17/KRT1/KRT10/KRT75/SLC1A4/KRT2/KRT19/KRT24/KRT76/KRT73	14
HS/REC	BP	GO:0045109	intermediate filament organization	13/90	73/18870	1.83471E-17	3.54649E-14	3.34882E-14	KRT5/KRT14/DSP/KRT3/KRT17/KRT1/KRT10/KRT75/KRT2/KRT19/KRT24/KRT76/KRT73	13
REC/CON	BP	GO:0045109	intermediate filament organization	13/123	73/18870	1.24317E-15	2.6691E-12	2.5557E-12	KRT5/KRT14/DSP/KRT3/KRT17/KRT1/KRT10/KRT75/KRT2/KRT19/KRT24/KRT76/KRT73	13
HS/REC	BP	GO:0045103	intermediate filament-based process	13/90	95/18870	6.82532E-16	4.39778E-13	4.15267E-13	KRT5/KRT14/DSP/KRT3/KRT17/KRT1/KRT10/KRT75/KRT2/KRT19/KRT24/KRT76/KRT73	13
REC/CON	BP	GO:0045103	intermediate filament-based process	13/123	95/18870	4.46159E-14	3.19301E-11	3.05737E-11	KRT5/KRT14/DSP/KRT3/KRT17/KRT1/KRT10/KRT75/KRT2/KRT19/KRT24/KRT76/KRT73	13
HS/CON	BP	GO:0016226	iron-sulfur cluster assembly	3/83	30/18870	0.000305767	0.043999166	0.039965995	CIAO2B/FDX2/BOLA2B	3
HS/CON	CC	GO:1990229	iron-sulfur cluster assembly complex	2/82	12/19886	0.001079248	0.036848597	0.032458575	FDX2/BOLA2B	2
HS/REC	CC	GO:0045095	keratin filament	10/92	97/19886	2.45892E-11	2.55728E-09	2.40715E-09	KRT5/KRT14/KRT3/KRT17/KRT1/KRT10/KRT75/KRT2/KRT76/KRT73	10
REC/CON	CC	GO:0045095	keratin filament	10/125	97/19886	5.30193E-10	4.53498E-08	4.17696E-08	KRT5/KRT14/KRT3/KRT17/KRT1/KRT10/KRT75/KRT2/KRT76/KRT73	10
HS/REC	BP	GO:0031424	keratinization	8/90	83/18870	5.74442E-09	2.22079E-06	2.09702E-06	KRT5/KRT3/KRT17/KRT1/KRT75/KRT2/KRT76/KRT73	8
REC/CON	BP	GO:0031424	keratinization	8/123	83/18870	6.79029E-08	2.91575E-05	2.79188E-05	KRT5/KRT3/KRT17/KRT1/KRT75/KRT2/KRT76/KRT73	8
HS/CON	BP	GO:0031163	metallo-sulfur cluster assembly	3/83	30/18870	0.000305767	0.043999166	0.039965995	CIAO2B/FDX2/BOLA2B	3
HS/REC	BP	GO:1904753	negative regulation of vascular associated smooth muscle cell migration	3/90	26/18870	0.000251947	0.030438355	0.028741856	TPM1/GNA13/PRKG1	3
REC/CON	MF	GO:0030414	peptidase inhibitor activity	7/124	175/18496	0.000178875	0.016143451	0.014874852	SERPINA1/KNG1/SERPINF2/FETUB/CST6/ITIH1/RECK	7
REC/CON	MF	GO:0061134	peptidase regulator activity	8/124	228/18496	0.000152257	0.016143451	0.014874852	SERPINA1/KNG1/SERPINF2/FETUB/TGFB1/CST6/ITIH1/RECK	8
HS/REC	BP	GO:0018149	peptide cross-linking	4/90	28/18870	9.07563E-06	0.001949244	0.001840602	DSP/KRT1/KRT10/KRT2	4
REC/CON	BP	GO:0018149	peptide cross-linking	4/123	28/18870	3.11803E-05	0.006694401	0.006410004	DSP/KRT1/KRT10/KRT2	4
REC/CON	CC	GO:0031091	platelet alpha granule	6/125	91/19886	2.35555E-05	0.000659555	0.000607485	ALB/SERPINA1/KNG1/ORM1/SERPINF2/TGFB1	6
HS/REC	CC	GO:0031091	platelet alpha granule	4/92	91/19886	0.000843256	0.025056764	0.02358582	ALB/PLG/ORM1/TGFB1	4
REC/CON	CC	GO:0031093	platelet alpha granule lumen	6/125	67/19886	3.98762E-06	0.000127604	0.00011753	ALB/SERPINA1/KNG1/ORM1/SERPINF2/TGFB1	6
HS/REC	CC	GO:0031093	platelet alpha granule lumen	4/92	67/19886	0.000263136	0.009122041	0.008586537	ALB/PLG/ORM1/TGFB1	4
HS/CON	BP	GO:0006457	protein folding	10/83	223/18870	4.97491E-08	7.87528E-05	7.1534E-05	HSPH1/BAG3/HSPB1/CHORDC1/HSPA6/HSPA1L/CRYAB/BAG2/DNAJB2/BAG4	10
HS/CON	MF	GO:0044183	protein folding chaperone	4/82	66/18496	0.000209832	0.010701441	0.00910009	HSPH1/HSPB1/HSPA6/HSPA1L	4
HS/CON	CC	GO:0101031	protein folding chaperone complex	3/82	42/19886	0.000690814	0.027517419	0.024239083	BAG3/BAG2/HSPB8	3
REC/CON	CC	GO:0033177	proton-transporting two-sector ATPase complex, proton-transporting domain	3/125	26/19886	0.000567149	0.010586783	0.009750984	ATP6V0A2/ATP6V0D1/ATP5PF	3
REC/CON	CC	GO:0033179	proton-transporting V-type ATPase, V0 domain	2/125	12/19886	0.00248253	0.037072454	0.034145681	ATP6V0A2/ATP6V0D1	2
HS/REC	BP	GO:1904752	regulation of vascular associated smooth muscle cell migration	4/90	50/18870	9.42267E-05	0.01529481	0.014442345	TPM1/GNA13/PRKG1/ADAMTS1	4
HS/CON	BP	GO:0009266	response to temperature stimulus	6/83	171/18870	0.000107524	0.024315814	0.022086911	BAG3/HSPB1/CHORDC1/HSPA6/CRYAB/NFKBIA	6
HS/CON	BP	GO:0035966	response to topologically incorrect protein	7/83	159/18870	6.41235E-06	0.002030149	0.001844056	HSPH1/BAG3/HSPB1/HSPA6/HSPA1L/DNAJB2/HSPB8	7
REC/CON	CC	GO:0034774	secretory granule lumen	10/125	322/19886	3.69246E-05	0.000834451	0.000768573	ALB/APOA1/HSPA6/SERPINA1/KNG1/ORM1/SERPINF2/GCA/S100A11/TGFB1	10
HS/CON	CC	GO:0034774	secretory granule lumen	7/82	322/19886	0.000367781	0.018931432	0.016676002	PKM/HSPA6/COMMD9/GCA/S100A11/CSTB/MIF	7
HS/REC	MF	GO:0005200	structural constituent of cytoskeleton	6/94	112/18496	2.27519E-05	0.00327627	0.003005642	TPM1/KRT5/KRT14/DSP/KRT2/KRT19	6
HS/REC	MF	GO:0030280	structural constituent of skin epidermis	8/94	36/18496	8.84303E-12	2.54679E-09	2.33642E-09	KRT5/KRT3/KRT1/KRT10/KRT75/KRT2/KRT76/KRT73	8
REC/CON	MF	GO:0030280	structural constituent of skin epidermis	8/124	36/18496	8.39822E-11	3.03176E-08	2.79351E-08	KRT5/KRT3/KRT1/KRT10/KRT75/KRT2/KRT76/KRT73	8
REC/CON	BP	GO:0000101	sulfur amino acid transport	3/123	12/18870	5.69583E-05	0.011117234	0.010644943	SLC3A2/SLC1A4/SLC7A11	3
HS/CON	MF	GO:0031625	ubiquitin protein ligase binding	7/82	308/18496	0.000433589	0.015795028	0.013431479	HSPA6/HSPA1L/BAG2/AMBRA1/BAG4/NFKBIA/BID	7
HS/CON	MF	GO:0044389	ubiquitin-like protein ligase binding	7/82	327/18496	0.000618882	0.019726867	0.016774962	HSPA6/HSPA1L/BAG2/AMBRA1/BAG4/NFKBIA/BID	7
HS/CON	MF	GO:0051082	unfolded protein binding	5/82	122/18496	0.00020916	0.010701441	0.00910009	HSPB1/HSPA6/HSPA1L/CRYAB/DNAJB2	5
REC/CON	CC	GO:0000220	vacuolar proton-transporting V-type ATPase, V0 domain	2/125	10/19886	0.001706624	0.027305982	0.025150247	ATP6V0A2/ATP6V0D1	2
HS/REC	BP	GO:1904738	vascular associated smooth muscle cell migration	4/90	52/18870	0.000109964	0.01529481	0.014442345	TPM1/GNA13/PRKG1/ADAMTS1	4
HS/REC	BP	GO:0042060	wound healing	10/90	423/18870	3.33233E-05	0.006441401	0.006082385	TPM1/PLG/DSP/KRT1/GNA13/TGFB1/JMJD1C/DTNBP1/PRKG1/SLC7A11	10
