# Supplementary material for: Development and characterization of Hanwoo oviduct epithelial cell line for heat stress study
Source: Anim Biosci. 2026 Sep 1;39(11):250962. doi: 10.5713/ab.250962 (PMC13740644; doi:10.5713/ab.250962)
Supplement: Supplementary file 5 [file ab-250962-Supplementary-13.pdf]

Supplement 13. Oviduct epithelial cells Downregulated DEP - GO Enrichment (BP, MF, CC).

Cluster	ONTOLOGY	ID	Description	GeneRatio	BgRatio	pvalue	p.adjust	qvalue	geneID	Count
REC/CON	BP	GO:0030199	collagen fibril organization	5/103	65/18870	2.79874E-05	0.011873649	0.011482194	COL1A1/COL12A1/COL1A2/COL3A1/COL5A1	5
REC/CON	CC	GO:0005788	endoplasmic reticulum lumen	10/104	313/19886	5.71382E-06	0.000187604	0.000176427	COL1A1/COL12A1/COL1A2/COL3A1/COL6A2/COL5A1/MXRA8/COL15A1/MSLN/COL6A3	10
HS/CON	CC	GO:0005788	endoplasmic reticulum lumen	9/159	313/19886	0.00095666	0.027955731	0.025958494	COL1A1/COL12A1/COL3A1/COL5A1/COL18A1/MXRA8/COL15A1/PRSS23/COL6A3	9
REC/CON	MF	GO:0005201	extracellular matrix structural constituent	13/107	166/18496	1.33263E-11	3.21163E-09	3.01594E-09	COL1A1/COL12A1/COL1A2/COL3A1/COL6A2/COL5A1/TGFBI/CTHRC1/MXRA5/COL15A1/TFPI2/COL6A3/MFAP5	13
HS/CON	MF	GO:0005201	extracellular matrix structural constituent	10/159	166/18496	1.74763E-06	0.000240591	0.00022014	COL1A1/COL12A1/COL3A1/COL5A1/COL18A1/MXRA5/COL15A1/TFPI2/COL6A3/MFAP5	10
REC/CON	MF	GO:0030020	extracellular matrix structural constituent conferring tensile strength	8/107	45/18496	1.73624E-10	2.09216E-08	1.96469E-08	COL1A1/COL12A1/COL1A2/COL3A1/COL6A2/COL5A1/COL15A1/COL6A3	8
HS/CON	MF	GO:0030020	extracellular matrix structural constituent conferring tensile strength	7/159	45/18496	1.04819E-07	4.32903E-05	3.96106E-05	COL1A1/COL12A1/COL3A1/COL5A1/COL18A1/COL15A1/COL6A3	7
HS/CON	CC	GO:0000118	histone deacetylase complex	5/159	82/19886	0.000509912	0.019728722	0.018319246	CHD5/DNTTIP1/JMJD1C/HDAC4/RERE	5
HS/CON	CC	GO:0005759	mitochondrial matrix	11/159	487/19886	0.001901094	0.048766394	0.045282383	CDK1/TYMS/POLRMT/ERAL1/ACAD8/DDX28/MRPL30/MRPL17/COQ3/MRM1/TK2	11
HS/CON	MF	GO:0008171	O-methyltransferase activity	5/159	23/18496	1.30875E-06	0.000240591	0.00022014	COQ3/MRM1/TRMT13/PCMTD2/PCMTD1	5
REC/CON	MF	GO:0008171	O-methyltransferase activity	3/107	23/18496	0.000306412	0.018461326	0.017336471	MRM1/TRMT13/PCMTD1	3
REC/CON	BP	GO:1902275	regulation of chromatin organization	4/103	49/18870	0.000146865	0.031153767	0.030126677	ATF7IP/MSL1/PHF20/RLIM	4
REC/CON	BP	GO:1900095	regulation of dosage compensation by inactivation of X chromosome	3/103	13/18870	4.34107E-05	0.014733606	0.014247862	MSL1/PHF20/RLIM	3
HS/CON	CC	GO:0005657	replication fork	4/159	67/19886	0.002039659	0.048766394	0.045282383	UHRF1/ETAA1/RADX/ZRANB3	4
REC/CON	CC	GO:0098643	banded collagen fibril	4/104	12/19886	3.38327E-07	2.22168E-05	2.08932E-05	COL1A1/COL1A2/COL3A1/COL5A1	4
HS/CON	CC	GO:0098643	banded collagen fibril	3/159	12/19886	0.000104656	0.009174853	0.008519375	COL1A1/COL3A1/COL5A1	3
HS/CON	CC	GO:0045009	chitosome	3/159	21/19886	0.000600113	0.019728722	0.018319246	DTNBP1/TPCN2/MREG	3
REC/CON	CC	GO:0005581	collagen trimer	9/104	86/19886	6.77669E-10	1.33501E-07	1.25547E-07	COL1A1/COL12A1/COL1A2/COL3A1/COL6A2/COL5A1/CTHRC1/COL15A1/COL6A3	9
HS/CON	CC	GO:0005581	collagen trimer	7/159	86/19886	5.7911E-06	0.001523058	0.001414247	COL1A1/COL12A1/COL3A1/COL5A1/COL18A1/COL15A1/COL6A3	7
REC/CON	CC	GO:0062023	collagen-containing extracellular matrix	12/104	429/19886	2.51241E-06	0.000123736	0.000116364	COL1A1/COL12A1/COL1A2/COL3A1/COL6A2/COL5A1/TGFBI/CTHRC1/MXRA5/COL15A1/COL6A3/MFAP5	12
REC/CON	CC	GO:0098644	complex of collagen trimers	4/104	21/19886	3.94512E-06	0.000155438	0.000146177	COL1A1/COL1A2/COL3A1/COL5A1	4
HS/CON	CC	GO:0098644	complex of collagen trimers	3/159	21/19886	0.000600113	0.019728722	0.018319246	COL1A1/COL3A1/COL5A1	3
REC/CON	BP	GO:0045229	external encapsulating structure organization	10/103	323/18870	1.08519E-05	0.006138556	0.005936178	COL1A1/COL12A1/COL1A2/COL3A1/COL5A1/TGFBI/RUNX1/COL15A1/OLFML2A/IER3IP1	10
REC/CON	BP	GO:0030198	extracellular matrix organization	10/103	321/18870	1.02815E-05	0.006138556	0.005936178	COL1A1/COL12A1/COL1A2/COL3A1/COL5A1/TGFBI/RUNX1/COL15A1/OLFML2A/IER3IP1	10
REC/CON	BP	GO:0043062	extracellular structure organization	10/103	322/18870	1.05634E-05	0.006138556	0.005936178	COL1A1/COL12A1/COL1A2/COL3A1/COL5A1/TGFBI/RUNX1/COL15A1/OLFML2A/IER3IP1	10
REC/CON	CC	GO:0005583	fibrillar collagen trimer	4/104	12/19886	3.38327E-07	2.22168E-05	2.08932E-05	COL1A1/COL1A2/COL3A1/COL5A1	4
HS/CON	CC	GO:0005583	fibrillar collagen trimer	3/159	12/19886	0.000104656	0.009174853	0.008519375	COL1A1/COL3A1/COL5A1	3
HS/CON	CC	GO:0033162	melanosome membrane	3/159	21/19886	0.000600113	0.019728722	0.018319246	DTNBP1/TPCN2/MREG	3
REC/CON	BP	GO:0048935	peripheral nervous system neuron development	3/103	15/18870	6.85159E-05	0.0166102	0.016062589	TBCE/RUNX1/NEFH	3
REC/CON	BP	GO:0048934	peripheral nervous system neuron differentiation	3/103	15/18870	6.85159E-05	0.0166102	0.016062589	TBCE/RUNX1/NEFH	3
HS/CON	CC	GO:0090741	pigment granule membrane	3/159	21/19886	0.000600113	0.019728722	0.018319246	DTNBP1/TPCN2/MREG	3
REC/CON	MF	GO:0048407	platelet-derived growth factor binding	4/107	11/18496	3.38598E-07	2.72007E-05	2.55434E-05	COL1A1/COL1A2/COL3A1/COL5A1	4
HS/CON	MF	GO:0048407	platelet-derived growth factor binding	3/159	11/18496	9.77807E-05	0.010095862	0.009237707	COL1A1/COL3A1/COL5A1	3
