# Supplementary material for: Development and characterization of Hanwoo oviduct epithelial cell line for heat stress study
Source: Anim Biosci. 2026 Sep 1;39(11):250962. doi: 10.5713/ab.250962 (PMC13740644; doi:10.5713/ab.250962)
Supplement: Supplementary file 6 [file ab-250962-Supplementary-14.pdf]

Supplement 14. Oviduct epithelial cells Upregulated DEG Pathway Enrichment (KEGG and Reactome).

LogP_HS_CON	LogP_REC_CON	GO	Category	Description	Enrichment	#GeneInGOAndHitList	Hits
-8.891444296	-6.3912668	R-HSA-9648895	Reactome Gene Sets	Response of EIF2AK1 (HRI) to heme deficiency	49.16747967	6	ASNS|ATF3|CEBPG|PPP1R15A|TRIB3|CHAC1
-2.870739459	0	R-HSA-380994	Reactome Gene Sets	ATF4 activates genes in response to endoplasmic reticulum stress	13.65763324	3	ASNS|ATF3|CEBPG
-2.654380488	0	R-HSA-381042	Reactome Gene Sets	PERK regulates gene expression	11.52362805	3	ASNS|ATF3|CEBPG
-2.14921292	0	R-HSA-381119	Reactome Gene Sets	Unfolded Protein Response (UPR)	5.286825771	4	ASNS|ATF3|CEBPG|CREBRF
-2.010710278	0	R-HSA-9633012	Reactome Gene Sets	Response of EIF2AK4 (GCN2) to amino acid deficiency	4.820341145	4	ASNS|ATF3|CEBPG|TRIB3
-7.488463555	-5.275341209	R-HSA-379716	Reactome Gene Sets	Cytosolic tRNA aminoacylation	30.7296748	6	AARS1|GARS1|SARS1|WARS1|YARS1|EEF1E1
-7.33877926	-4.043680603	R-HSA-379724	Reactome Gene Sets	tRNA Aminoacylation	20.48644986	7	AARS1|GARS1|SARS1|WARS1|YARS1|EEF1E1|EARS2
-4.783102026	-2.233597596	hsa00970	KEGG Pathway	Aminoacyl-tRNA biosynthesis	11.1744272	6	AARS1|GARS1|SARS1|WARS1|YARS1|EARS2
-2.383570146	0	R-HSA-72766	Reactome Gene Sets	Translation	3.161895799	8	AARS1|GARS1|SARS1|WARS1|YARS1|EEF1E1|SEC61A2|EARS2
0	-7.077063459	R-HSA-556833	Reactome Gene Sets	Metabolism of lipids	3.278121524	27	ABCA1|ALDH3B1|ARSA|GALC|HMGCL|INPP4A|NEU1|SMPD1|SMPD2|PLPP1|MTMR1|SYNJ1|ECI2|TNFAIP8|CYP39A1|INPP5K|TRIB3|MID1IP1|GLB1L|PIP4K2C|LPCAT1|ACAD10|PLA2G12A|PIP4P1|PTPMT1|PGP|HACD4
0	-4.63769657	R-HSA-1483257	Reactome Gene Sets	Phospholipid metabolism	4.768853587	11	INPP4A|MTMR1|SYNJ1|TNFAIP8|INPP5K|PIP4K2C|LPCAT1|PLA2G12A|PIP4P1|PTPMT1|PGP
0	-4.426676475	R-HSA-1483255	Reactome Gene Sets	PI Metabolism	7.659067882	7	INPP4A|MTMR1|SYNJ1|TNFAIP8|INPP5K|PIP4K2C|PIP4P1
0	-3.558028569	R-HSA-1660499	Reactome Gene Sets	Synthesis of PIPs at the plasma membrane	8.670642886	5	INPP4A|MTMR1|SYNJ1|INPP5K|PIP4K2C
0	-2.916004761	hsa00562	KEGG Pathway	Inositol phosphate metabolism	6.295124287	5	INPP4A|MTMR1|SYNJ1|INPP5K|PIP4K2C
0	-2.356187884	hsa04070	KEGG Pathway	Phosphatidylinositol signaling system	4.689225234	5	INPP4A|MTMR1|SYNJ1|PIP4K2C|PIP4P1
-6.806057875	-5.147681228	R-HSA-2262752	Reactome Gene Sets	Cellular responses to stress	3.578645673	23	ASNS|ATF3|CDKN1A|CEBPG|CRYAB|FOXO3|FOS|ID1|MDM2|NOTCH1|H2BC11|LAMTOR5|TXNIP|NPRL2|SIRT3|PPP1R15A|SLC7A11|POT1|ITFG2|TRIB3|CHAC1|SESN2|CREBRF
-4.335806031	-2.765020604	R-HSA-9711097	Reactome Gene Sets	Cellular response to starvation	6.263373207	8	ASNS|ATF3|CEBPG|LAMTOR5|NPRL2|ITFG2|TRIB3|SESN2
-2.97760513	-2.518101687	R-HSA-9639288	Reactome Gene Sets	Amino acids regulate mTORC1	7.109247649	6	ATP6V1B1|ATP6V1G1|LAMTOR5|NPRL2|ITFG2|SESN2
-6.713585123	-2.798778355	R-HSA-3700989	Reactome Gene Sets	Transcriptional Regulation by TP53	5.313488437	15	CDKN1A|GADD45A|FOS|MDM2|PLAGL1|PMAIP1|RAD1|TAF10|TAF12|LAMTOR5|RGCC|DDIT4|BANP|SESN2|JMY
-2.759800781	-2.144975232	R-HSA-5633007	Reactome Gene Sets	Regulation of TP53 Activity	3.783954394	9	MDM2|RAD1|TAF10|TAF12|NOC2L|BANP|PIP4K2C|PIP4P1|JMY
-2.258929022	0	hsa03022	KEGG Pathway	Basal transcription factors	8.380820399	3	GTF2E1|TAF10|TAF12
-2.218902657	0	R-HSA-3214847	Reactome Gene Sets	HATs acetylate histones	4.35881912	5	ATXN7|TAF10|TAF12|H2BC11|YEATS2
-2.178779047	0	R-HSA-167161	Reactome Gene Sets	HIV Transcription Initiation	7.845874416	3	GTF2E1|TAF10|TAF12
-2.178779047	0	R-HSA-167162	Reactome Gene Sets	RNA Polymerase II HIV Promoter Escape	7.845874416	3	GTF2E1|TAF10|TAF12
-2.178779047	0	R-HSA-73776	Reactome Gene Sets	RNA Polymerase II Promoter Escape	7.845874416	3	GTF2E1|TAF10|TAF12
-2.178779047	0	R-HSA-73779	Reactome Gene Sets	RNA Polymerase II Transcription Pre-Initiation And Promoter Opening	7.845874416	3	GTF2E1|TAF10|TAF12
-2.178779047	0	R-HSA-75953	Reactome Gene Sets	RNA Polymerase II Transcription Initiation	7.845874416	3	GTF2E1|TAF10|TAF12
-2.178779047	0	R-HSA-76042	Reactome Gene Sets	RNA Polymerase II Transcription Initiation And Promoter Clearance	7.845874416	3	GTF2E1|TAF10|TAF12
-2.165579795	0	R-HSA-6804756	Reactome Gene Sets	Regulation of TP53 Activity through Phosphorylation	5.344291269	4	MDM2|RAD1|TAF10|TAF12
-5.146407786	-5.726671625	R-HSA-71291	Reactome Gene Sets	Metabolism of amino acids and derivatives	4.17767339	16	ASNS|CBS|CTH|DBT|PSPH|PYCR1|SAT1|SLC3A2|EEF1E1|ETHE1|PHGDH|PSAT1|ASRGL1|MRI1|HOGA1|PPM1K
-4.463977771	-4.748734905	hsa01230	KEGG Pathway	Biosynthesis of amino acids	8.578156028	7	ASNS|CBS|CTH|PSPH|PYCR1|PHGDH|PSAT1
-3.466316022	-4.094865759	hsa00260	KEGG Pathway	Glycine, serine and threonine metabolism	11.20839202	5	CBS|CTH|PSPH|PHGDH|PSAT1
-4.077619218	-3.70467332	R-HSA-977347	Reactome Gene Sets	Serine metabolism	33.5232816	3	PSPH|PHGDH|PSAT1
0	-3.639736103	R-HSA-1614635	Reactome Gene Sets	Sulfur amino acid metabolism	13.12983066	4	CBS|CTH|ETHE1|MRI1
-3.069634728	-3.597211565	hsa00270	KEGG Pathway	Cysteine and methionine metabolism	8.837386018	5	CBS|CTH|PHGDH|PSAT1|MRI1
-4.116664167	-3.34559347	R-HSA-196849	Reactome Gene Sets	Metabolism of water-soluble vitamins and cofactors	5.734793103	11	BTD|MTHFR|SLC2A3|SLC25A16|MTHFS|MTHFD2|TPK1|PARP6|PPCS|NUDT12|ALDH1L2
-2.407115365	-4.201611341	hsa00670	KEGG Pathway	One carbon pool by folate	10.02586207	6	CBS|CTH|MTHFR|MTHFS|MTHFD2|ALDH1L2
0	-3.110689459	R-HSA-196757	Reactome Gene Sets	Metabolism of folate and pterines	15.3336714	4	MTHFR|MTHFS|MTHFD2|ALDH1L2
-2.907582411	-2.209936214	R-HSA-196854	Reactome Gene Sets	Metabolism of vitamins and cofactors	3.620450192	11	BTD|MTHFR|SLC2A3|SLC25A16|MTHFS|MTHFD2|TPK1|PARP6|PPCS|NUDT12|ALDH1L2
-2.407115365	-5.417768779	R-HSA-9840310	Reactome Gene Sets	Glycosphingolipid catabolism	14.13981763	6	ARSA|GALC|NEU1|SMPD1|SMPD2|GLB1L
0	-4.636369328	R-HSA-428157	Reactome Gene Sets	Sphingolipid metabolism	6.871687072	8	ALDH3B1|ARSA|GALC|NEU1|SMPD1|SMPD2|PLPP1|GLB1L
0	-4.532575392	hsa00600	KEGG Pathway	Sphingolipid metabolism	10.02641614	6	ARSA|GALC|NEU1|SMPD1|SMPD2|PLPP1
0	-4.399510541	R-HSA-1660662	Reactome Gene Sets	Glycosphingolipid metabolism	9.507808406	6	ARSA|GALC|NEU1|SMPD1|SMPD2|GLB1L
0	-2.465398317	hsa04142	KEGG Pathway	Lysosome	4.146262312	6	ARSA|GALC|NEU1|NPC1|SMPD1|CTSF
-5.199620951	-2.749320395	hsa04150	KEGG Pathway	mTOR signaling pathway	7.001698055	9	SLC3A2|FZD3|SLC7A5|ULK1|LAMTOR5|NPRL2|DDIT4|STRADB|SESN2
-5.171212235	-3.35761093	R-HSA-352230	Reactome Gene Sets	Amino acid transport across the plasma membrane	18.62404533	5	SLC1A4|SLC1A5|SLC3A2|SLC7A5|SLC7A11
-4.496414491	-2.162360288	R-HSA-425393	Reactome Gene Sets	Transport of inorganic cations/anions and amino acids/oligopeptides	7.893861416	7	SLC26A3|SLC1A4|SLC1A5|SLC3A2|SLC7A5|SLC7A11|SLC25A22
-4.433960701	0	R-HSA-425407	Reactome Gene Sets	SLC-mediated transmembrane transport	4.996695089	10	SLC26A3|SLC1A4|SLC1A5|SLC2A3|SLC3A2|SLC6A9|SLCO2A1|SLC7A5|SLC7A11|SLC25A22
-4.036765813	-2.611247252	R-HSA-382551	Reactome Gene Sets	Transport of small molecules	2.910331318	17	SLC26A3|TSC22D3|NPC1|SLC1A4|SLC1A5|SLC2A3|SLC3A2|SLC6A9|SLCO2A1|VLDLR|SLC7A5|AKAP1|SLC7A11|MYLIP|SLC25A22|SMDT1|AQP11
-2.969931529	-2.609419968	R-HSA-210991	Reactome Gene Sets	Basigin interactions	10.42689655	4	SLC3A2|SLC7A5|SLC7A7|SLC7A11
-2.315835809	0	hsa04216	KEGG Pathway	Ferroptosis	8.779907085	3	SAT1|SLC3A2|SLC7A11
-4.647845401	0	hsa05418	KEGG Pathway	Fluid shear stress and atherosclerosis	6.924997137	8	ACVR2A|EDN1|FOS|GSTM1|ICAM1|PLAT|MAP2K5|ARHGEF2
-4.497249112	0	R-HSA-6807505	Reactome Gene Sets	RNA polymerase II transcribes snRNA genes	9.966381015	6	GTF2E1|INTS6|PCF11|NABP1|NABP2|ICE2
-4.463977771	-3.769937281	hsa04115	KEGG Pathway	p53 signaling pathway	9.833495935	6	CDKN1A|GADD45A|MDM2|PMAIP1|ZMAT3|SESN2
-3.478285593	-3.110689459	R-HSA-9617828	Reactome Gene Sets	FOXO-mediated transcription of cell cycle genes	21.69153515	3	CDKN1A|GADD45A|FOXO3
-3.202032741	0	R-HSA-6791312	Reactome Gene Sets	TP53 Regulates Transcription of Cell Cycle Genes	10.24322493	4	CDKN1A|GADD45A|PLAGL1|RGCC
-3.101736815	-3.184448596	hsa04068	KEGG Pathway	FoxO signaling pathway	4.837306031	7	CDKN1A|GADD45A|FOXO3|MDM2|PCK2|ATG12|GABARAP
-2.969931529	-2.609419968	R-HSA-5674400	Reactome Gene Sets	Constitutive Signaling by AKT1 E17K in Cancer	14.7502439	3	CDKN1A|FOXO3|MDM2
-2.82047369	0	hsa05200	KEGG Pathway	Pathways in cancer	2.767400357	12	CDKN1A|GADD45A|EDN1|FOS|GSTM1|IFNGR1|MDM2|NOTCH1|PMAIP1|PPARG|FZD3|TRAF4
-2.731659806	0	hsa04218	KEGG Pathway	Cellular senescence	4.697529905	6	CDKN1A|GADD45A|E2F5|FOXO3|MDM2|RAD1
-2.480632104	0	hsa01524	KEGG Pathway	Platinum drug resistance	6.555663957	4	CDKN1A|GSTM1|MDM2|PMAIP1
-2.472412001	0	hsa05216	KEGG Pathway	Thyroid cancer	9.966381015	3	CDKN1A|GADD45A|PPARG
0	-2.305350511	hsa05217	KEGG Pathway	Basal cell carcinoma	5.835480291	4	CDKN1A|GADD45A|WNT7A|FZD3
-2.26839409	0	R-HSA-8878159	Reactome Gene Sets	Transcriptional regulation by RUNX3	5.717148799	4	CDKN1A|FOXO3|MDM2|NOTCH1
-2.250683643	0	hsa05210	KEGG Pathway	Colorectal cancer	5.651434445	4	CDKN1A|GADD45A|FOS|PMAIP1
-2.206082922	0	hsa05202	KEGG Pathway	Transcriptional misregulation in cancer	3.669214901	6	ATF1|CDKN1A|GADD45A|MDM2|PLAT|PPARG
-2.165636754	0	hsa05203	KEGG Pathway	Viral carcinogenesis	3.597620464	6	CDKN1A|GTF2E1|LTBR|MDM2|PMAIP1|H2BC11
0	-2.141492429	hsa05205	KEGG Pathway	Proteoglycans in cancer	3.153733834	7	ANK2|CDKN1A|MDM2|TIMP3|TLR4|WNT7A|FZD3
-2.131945904	0	hsa05224	KEGG Pathway	Breast cancer	4.152658756	5	CDKN1A|GADD45A|FOS|NOTCH1|FZD3
0	-2.080207199	hsa05218	KEGG Pathway	Melanoma	5.03609943	4	CDKN1A|GADD45A|MDM2|RB1
0	-2.080207199	hsa05223	KEGG Pathway	Non-small cell lung cancer	5.03609943	4	CDKN1A|GADD45A|FOXO3|RB1
-2.05520337	0	hsa01522	KEGG Pathway	Endocrine resistance	4.966412088	4	CDKN1A|FOS|MDM2|NOTCH1
0	-2.019757914	hsa05214	KEGG Pathway	Glioma	4.837306031	4	CDKN1A|GADD45A|MDM2|RB1
-2.016229327	0	hsa04110	KEGG Pathway	Cell cycle	3.889832253	5	CDKN1A|GADD45A|E2F5|MDM2|ORC5
0	-2.000243272	hsa05220	KEGG Pathway	Chronic myeloid leukemia	4.774483875	4	CDKN1A|GADD45A|MDM2|RB1
0	-4.427966772	R-HSA-9841251	Reactome Gene Sets	Mitochondrial unfolded protein response (UPRmt)	20.42418102	4	FOXO3|HSPA9|LONP1|SIRT3
-4.213303161	-3.839462646	R-HSA-9617629	Reactome Gene Sets	Regulation of FOXO transcriptional activity by acetylation	36.87560976	3	FOXO3|TXNIP|SIRT3
-3.753441298	-3.175952793	R-HSA-9614085	Reactome Gene Sets	FOXO-mediated transcription	9.603023374	5	CDKN1A|GADD45A|FOXO3|TXNIP|SIRT3
-2.506542724	-2.155680172	R-HSA-156590	Reactome Gene Sets	Glutathione conjugation	9.051125479	5	GGT7|GSTM1|OPLAH|CHAC1|GSTK1
0	-3.583246099	R-HSA-174403	Reactome Gene Sets	Glutathione synthesis and recycling	22.97720365	3	GGT7|OPLAH|CHAC1
0	-2.407518974	hsa00480	KEGG Pathway	Glutathione metabolism	6.627264757	6	GGT7|GPX3|GSTM1|OPLAH|CHAC1|GSTK1
-3.554568936	0	hsa01240	KEGG Pathway	Biosynthesis of cofactors	5.587213599	7	PPOX|GGH|MTHFD2|TPK1|PSAT1|PPCS|EARS2
-3.551125151	0	R-HSA-9031628	Reactome Gene Sets	NGF-stimulated transcription	12.60704607	4	ATF1|FOS|ID1|ID3
-2.809392807	0	R-HSA-198725	Reactome Gene Sets	Nuclear Events (kinase and transcription factor activation)	8.06024257	4	ATF1|FOS|ID1|ID3
-2.679149321	0	hsa04350	KEGG Pathway	TGF-beta signaling pathway	5.587213599	5	ACVR2A|E2F5|ID1|ID3|INHBE
-2.595055549	0	R-HSA-187037	Reactome Gene Sets	Signaling by NTRK1 (TRKA)	5.344291269	5	ATF1|FOS|ID1|ID3|MAP2K5
-2.325034183	0	R-HSA-166520	Reactome Gene Sets	Signaling by NTRKs	4.621003729	5	ATF1|FOS|ID1|ID3|MAP2K5
-2.180995544	0	hsa04550	KEGG Pathway	Signaling pathways regulating pluripotency of stem cells	4.268010388	5	ACVR2A|ID1|ID3|FZD3|INHBE
0	-3.210530323	R-HSA-8956319	Reactome Gene Sets	Nucleotide catabolism	10.21209051	4	DPYD|NT5C3A|NUDT9|ADPRM
0	-2.356187884	R-HSA-15869	Reactome Gene Sets	Metabolism of nucleotides	3.989883885	6	DPYD|NME4|NT5C3A|NUDT9|UCKL1|ADPRM
0	-3.19200288	R-HSA-5668914	Reactome Gene Sets	Diseases of metabolism	3.507970023	10	ABCA3|BTD|DBT|GYG1|NEU1|OMD|OPLAH|PPM1K|THSD7A|ADAMTSL5
0	-3.165143219	hsa04270	KEGG Pathway	Vascular smooth muscle contraction	4.801206732	7	ACTG2|AGTR1|PRKCE|PRKG1|CALCRL|ADM2|PLA2G12A
0	-2.863318583	R-HSA-174824	Reactome Gene Sets	Plasma lipoprotein assembly, remodeling, and clearance	6.127254306	5	ABCA1|NPC1|PLTP|VLDLR|MYLIP
0	-2.793014767	R-HSA-9006931	Reactome Gene Sets	Signaling by Nuclear Receptors	3.10502752	10	ABCA1|FOXO3|FOXA1|MMP7|CITED1|PLTP|H2BC21|H2BC11|KDM4A|MYLIP
0	-2.770100559	R-HSA-9024446	Reactome Gene Sets	NR1H2 and NR1H3-mediated signaling	7.822026774	4	ABCA1|PLTP|KDM4A|MYLIP
0	-2.638412983	hsa04979	KEGG Pathway	Cholesterol metabolism	7.208534478	4	ABCA1|NPC1|PLTP|MYLIP
-2.472412001	-2.122421246	R-HSA-8964043	Reactome Gene Sets	Plasma lipoprotein clearance	9.966381015	3	NPC1|VLDLR|MYLIP
0	-2.122421246	R-HSA-9029569	Reactome Gene Sets	NR1H3 & NR1H2 regulate gene expression linked to cholesterol transport and efflux	7.452066048	3	ABCA1|PLTP|KDM4A
-2.679149321	0	hsa04066	KEGG Pathway	HIF-1 signaling pathway	5.587213599	5	CDKN1A|EDN1|MKNK2|IFNGR1|LTBR
-2.484372488	0	hsa04152	KEGG Pathway	AMPK signaling pathway	5.037651606	5	FOXO3|PCK2|PPARG|ULK1|STRADB
-2.198952625	0	hsa04211	KEGG Pathway	Longevity regulating pathway	5.463053297	4	FOXO3|PPARG|ULK1|SESN2
-2.34543366	0	R-HSA-9772755	Reactome Gene Sets	Formation of WDR5-containing histone-modifying complexes	8.99405116	3	HCFC2|YEATS2|BOD1
-2.322863395	0	R-HSA-2559583	Reactome Gene Sets	Cellular Senescence	3.881643132	6	CDKN1A|FOS|ID1|MDM2|H2BC11|POT1
0	-2.080207199	R-HSA-2559586	Reactome Gene Sets	DNA Damage/Telomere Stress Induced Senescence	4.463568729	5	CDKN1A|RB1|H2BC21|H2BC11|POT1
0	-2.300021719	hsa04136	KEGG Pathway	Autophagy - other	8.616451368	3	ATG12|ATG13|GABARAP
0	-2.240519708	R-HSA-6798695	Reactome Gene Sets	Neutrophil degranulation	2.346608032	12	ALDH3B1|ARSA|CST3|GYG1|NEU1|SLC2A3|MVP|HEBP2|YPEL5|LPCAT1|CYSTM1|MOSPD2
-2.233210199	0	hsa04610	KEGG Pathway	Complement and coagulation cascades	5.587213599	4	CLU|SERPINB2|PLAT|PROCR
0	-2.064720213	R-HSA-109582	Reactome Gene Sets	Hemostasis	1.888930535	18	CLU|EPCAM|SERPINB2|PLAT|PRKCE|PRKG1|SLC3A2|STXBP2|TIMP3|SLC7A5|AKAP1|SLC7A7|PROCR|KIFAP3|SLC7A11|SCCPDH|GTPBP2|PCYOX1L
0	-2.090167621	R-HSA-8950505	Reactome Gene Sets	Gene and protein expression by JAK-STAT signaling after Interleukin-12 stimulation	7.255959047	3	HSPA9|SERPINB2|PSME2
0	-2.042746112	R-HSA-5619115	Reactome Gene Sets	Disorders of transmembrane transporters	3.383146549	6	ABCA1|ABCA3|SLC3A2|SLCO2A1|SLC7A7|SLC9A9
