# Supplementary material for: Development and characterization of Hanwoo oviduct epithelial cell line for heat stress study
Source: Anim Biosci. 2026 Sep 1;39(11):250962. doi: 10.5713/ab.250962 (PMC13740644; doi:10.5713/ab.250962)
Supplement: Supplementary file 7 [file ab-250962-Supplementary-15.pdf]

Supplement 15. Oviduct epithelial cells Downregulated DEG Pathway Enrichment (KEGG and Reactome).

<table>
<tr><th>_LogP_HS_CON</th><th>_LogP_REC_CON</th><th>GO</th><th>Category</th><th>Description</th><th>Enrichment</th><th>#GeneInGOAndHitList</th><th>Hits</th></tr>
<tr><td>-6.958361363</td><td>-8.881923792</td><td>hsa04657</td><td>KEGG Pathway</td><td>IL-17 signaling pathway</td><td>18.84639889</td><td>9</td><td>CXCL1|CXCL2|IL6|CXCL8|NFKBIA|PTGS2|CCL2|CXCL5|SRSF1</td></tr>
<tr><td>-5.135731508</td><td>-8.564037001</td><td>R-HSA-6783783</td><td>Reactome Gene Sets</td><td>Interleukin-10 signaling</td><td>30.94532164</td><td>7</td><td>CXCL1|CXCL2|IL1A|IL6|CXCL8|PTGS2|CCL2</td></tr>
<tr><td>-4.865268948</td><td>-8.386366823</td><td>hsa04060</td><td>KEGG Pathway</td><td>Cytokine-cytokine receptor interaction</td><td>8.678338043</td><td>13</td><td>BMP3|BMP4|CXCL1|CXCL2|IL1A|IL6|CXCL8|TNFRSF9|TNFRSF11B|CCL2|CXCL5|TGFB3|IL18R1</td></tr>
<tr><td>-6.201629462</td><td>-8.010833722</td><td>hsa04668</td><td>KEGG Pathway</td><td>TNF signaling pathway</td><td>15.04544449</td><td>9</td><td>CXCL1|CXCL2|IL6|NFKBIA|PTGS2|CCL2|CXCL5|PIK3R3|IL18R1</td></tr>
<tr><td>-5.759967518</td><td>-7.546019115</td><td>hsa05323</td><td>KEGG Pathway</td><td>Rheumatoid arthritis</td><td>16.75235457</td><td>8</td><td>CXCL1|CXCL2|IL1A|IL6|CXCL8|CCL2|CXCL5|TGFB3</td></tr>
<tr><td>-4.677765775</td><td>-7.084440419</td><td>R-HSA-449147</td><td>Reactome Gene Sets</td><td>Signaling by Interleukins</td><td>6.121052632</td><td>14</td><td>DUSP6|CXCL1|CXCL2|HGF|IL1A|IL6|CXCL8|LMNB1|NFKBIA|PTGS2|RORA|CCL2|PIK3R3|IL18R1</td></tr>
<tr><td>-4.139026609</td><td>-6.382024042</td><td>R-HSA-1280215</td><td>Reactome Gene Sets</td><td>Cytokine Signaling in Immune system</td><td>4.358094818</td><td>17</td><td>DUSP6|EGR1|CXCL1|CXCL2|HGF|IL1A|IL6|CXCL8|TNFRSF9|LMNB1|NFKBIA|TNFRSF11B|PTGS2|RORA|CCL2|PIK3R3|IL18R1</td></tr>
<tr><td>-5.609917533</td><td>-6.116772022</td><td>hsa04061</td><td>KEGG Pathway</td><td>Viral protein interaction with cytokine and cytokine receptor</td><td>13.92539474</td><td>7</td><td>CXCL1|CXCL2|IL6|CXCL8|CCL2|CXCL5|IL18R1</td></tr>
<tr><td>-3.441282327</td><td>-6.087398161</td><td>hsa04933</td><td>KEGG Pathway</td><td>AGE-RAGE signaling pathway in diabetic complications</td><td>13.78751954</td><td>7</td><td>EGR1|IL1A|IL6|CXCL8|CCL2|TGFB3|PIK3R3</td></tr>
<tr><td>-4.426850129</td><td>-6.029594684</td><td>hsa05146</td><td>KEGG Pathway</td><td>Amoebiasis</td><td>13.51980072</td><td>7</td><td>CXCL1|CXCL2|IL6|CXCL8|LAMA4|TGFB3|PIK3R3</td></tr>
<tr><td>-2.415849388</td><td>-5.945157214</td><td>R-HSA-6785807</td><td>Reactome Gene Sets</td><td>Interleukin-4 and Interleukin-13 signaling</td><td>13.13716485</td><td>7</td><td>HGF|IL1A|IL6|CXCL8|PTGS2|RORA|CCL2</td></tr>
<tr><td>-4.907144435</td><td>-5.275371038</td><td>hsa05144</td><td>KEGG Pathway</td><td>Malaria</td><td>13.74454545</td><td>6</td><td>HGF|IL6|CXCL8|CCL2|TGFB3|TLR9</td></tr>
<tr><td>-2.917977199</td><td>-5.146541405</td><td>hsa05167</td><td>KEGG Pathway</td><td>Kaposi sarcoma-associated herpesvirus infection</td><td>8.119763695</td><td>8</td><td>CXCL1|CXCL2|IL6|CXCL8|NFKBIA|PTGS2|PIK3R3|CD200R1L</td></tr>
<tr><td>-4.66378649</td><td>-5.029944446</td><td>hsa05134</td><td>KEGG Pathway</td><td>Legionellosis</td><td>17.76198308</td><td>5</td><td>CXCL1|CXCL2|IL6|CXCL8|NFKBIA</td></tr>
<tr><td>-4.552602575</td><td>-4.917727373</td><td>R-HSA-380108</td><td>Reactome Gene Sets</td><td>Chemokine receptors bind chemokines</td><td>16.8588314</td><td>5</td><td>CXCL1|CXCL2|CXCL8|CCL2|CXCL5</td></tr>
<tr><td>-4.426850129</td><td>-4.854373586</td><td>hsa05142</td><td>KEGG Pathway</td><td>Chagas disease</td><td>11.58840061</td><td>6</td><td>IL6|CXCL8|NFKBIA|CCL2|TGFB3|PIK3R3</td></tr>
<tr><td>-4.827548953</td><td>-2.634645085</td><td>hsa05168</td><td>KEGG Pathway</td><td>Herpes simplex virus 1 infection</td><td>7.343330702</td><td>8</td><td>CD74|IL6|NFKBIA|CCL2|TRAF5|PIK3R3|TLR9|ZC3HAV1</td></tr>
<tr><td>-4.379775129</td><td>-4.806593463</td><td>hsa04064</td><td>KEGG Pathway</td><td>NF-kappa B signaling pathway</td><td>11.36766917</td><td>6</td><td>CXCL1|CXCL2|CXCL8|NFKBIA|PLAU|PTGS2</td></tr>
<tr><td>-4.709276653</td><td>-4.298740709</td><td>hsa04621</td><td>KEGG Pathway</td><td>NOD-like receptor signaling pathway</td><td>7.071355491</td><td>8</td><td>CXCL1|CXCL2|IL6|CXCL8|NFKBIA|CCL2|TRAF5|VDAC2</td></tr>
<tr><td>-4.158592912</td><td>-4.698255091</td><td>hsa05163</td><td>KEGG Pathway</td><td>Human cytomegalovirus infection</td><td>7.041918957</td><td>8</td><td>IL6|CXCL8|NFKBIA|PDGFRA|PTGER3|PTGS2|CCL2|PIK3R3</td></tr>
<tr><td>-4.568307518</td><td>-4.035963989</td><td>hsa04936</td><td>KEGG Pathway</td><td>Alcoholic liver disease</td><td>8.121009822</td><td>7</td><td>CXCL1|CXCL2|IL6|CXCL8|NFKBIA|SCD|NOX4</td></tr>
<tr><td>-3.767869122</td><td>-4.241297</td><td>hsa04062</td><td>KEGG Pathway</td><td>Chemokine signaling pathway</td><td>7.215230434</td><td>7</td><td>CXCL1|CXCL2|CXCL8|NFKBIA|CCL2|CXCL5|PIK3R3</td></tr>
<tr><td>-3.470293338</td><td>-3.93552968</td><td>hsa05417</td><td>KEGG Pathway</td><td>Lipid and atherosclerosis</td><td>6.446942008</td><td>7</td><td>CXCL1|CXCL2|IL6|CXCL8|NFKBIA|CCL2|PIK3R3</td></tr>
<tr><td>-3.28920312</td><td>-3.637226641</td><td>hsa04620</td><td>KEGG Pathway</td><td>Toll-like receptor signaling pathway</td><td>9.125422501</td><td>5</td><td>IL6|CXCL8|NFKBIA|SPP1|PIK3R3</td></tr>
<tr><td>-3.198818587</td><td>-3.601831239</td><td>hsa05164</td><td>KEGG Pathway</td><td>Influenza A</td><td>6.899452388</td><td>6</td><td>IL1A|IL6|CXCL8|NFKBIA|CCL2|PIK3R3</td></tr>
<tr><td>-3.054213621</td><td>-3.338171152</td><td>hsa05120</td><td>KEGG Pathway</td><td>Epithelial cell signaling in Helicobacter pylori infection</td><td>11.20756116</td><td>4</td><td>CXCL1|CXCL2|CXCL8|NFKBIA</td></tr>
<tr><td>-3.219049469</td><td>-2.875724131</td><td>hsa05171</td><td>KEGG Pathway</td><td>Coronavirus disease - COVID-19</td><td>4.913552161</td><td>7</td><td>IL6|CXCL8|MX1|NFKBIA|RPL15|CCL2|PIK3R3</td></tr>
<tr><td>0</td><td>-3.18323529</td><td>hsa05133</td><td>KEGG Pathway</td><td>Pertussis</td><td>10.20175439</td><td>4</td><td>IL1A|IL6|CXCL8|CXCL5</td></tr>
<tr><td>-2.828730396</td><td>-3.166941785</td><td>hsa05135</td><td>KEGG Pathway</td><td>Yersinia infection</td><td>7.207761251</td><td>5</td><td>IL6|CXCL8|NFKBIA|CCL2|PIK3R3</td></tr>
<tr><td>0</td><td>-2.84393923</td><td>hsa05161</td><td>KEGG Pathway</td><td>Hepatitis B</td><td>6.102276397</td><td>5</td><td>IL6|CXCL8|NFKBIA|TGFB3|PIK3R3</td></tr>
<tr><td>0</td><td>-2.615636306</td><td>R-HSA-2559582</td><td>Reactome Gene Sets</td><td>Senescence-Associated Secretory Phenotype (SASP)</td><td>7.168800379</td><td>4</td><td>IL1A|IL6|CXCL8|H2BC21</td></tr>
<tr><td>0</td><td>-2.554068233</td><td>R-HSA-2559583</td><td>Reactome Gene Sets</td><td>Cellular Senescence</td><td>5.235110803</td><td>5</td><td>IL1A|IL6|CXCL8|LMNB1|H2BC21</td></tr>
<tr><td>0</td><td>-2.083219278</td><td>hsa04932</td><td>KEGG Pathway</td><td>Non-alcoholic fatty liver disease</td><td>5.068387529</td><td>4</td><td>IL1A|IL6|CXCL8|PIK3R3</td></tr>
<tr><td>-4.018529117</td><td>-7.881214643</td><td>hsa05200</td><td>KEGG Pathway</td><td>Pathways in cancer</td><td>5.971758665</td><td>16</td><td>ALK|BMP4|EDNRB|GSTA3|HGF|IL6|CXCL8|KIT|LAMA4|KITLG|NFKBIA|PDGFRA|PTGER3|PTGS2|TGFB3|PIK3R3</td></tr>
<tr><td>0</td><td>-5.306960446</td><td>R-HSA-2219530</td><td>Reactome Gene Sets</td><td>Constitutive Signaling by Aberrant PI3K in Cancer</td><td>13.87913097</td><td>6</td><td>HGF|NRG1|KIT|KITLG|PDGFRA|PIK3R3</td></tr>
<tr><td>0</td><td>-4.76888946</td><td>R-HSA-9006934</td><td>Reactome Gene Sets</td><td>Signaling by Receptor Tyrosine Kinases</td><td>4.487237831</td><td>12</td><td>ALK|DUSP6|EGR1|HGF|NRG1|KIT|LAMA4|KITLG|PDGFRA|SPP1|PIK3R3|SPRY2</td></tr>
<tr><td>0</td><td>-4.646937766</td><td>R-HSA-2219528</td><td>Reactome Gene Sets</td><td>PI3K/AKT Signaling in Cancer</td><td>10.65718985</td><td>6</td><td>HGF|NRG1|KIT|KITLG|PDGFRA|PIK3R3</td></tr>
<tr><td>0</td><td>-4.603341105</td><td>R-HSA-6811558</td><td>Reactome Gene Sets</td><td>PI5P, PP2A and IER3 Regulate PI3K/AKT Signaling</td><td>10.47022161</td><td>6</td><td>HGF|NRG1|KIT|KITLG|PDGFRA|PIK3R3</td></tr>
<tr><td>0</td><td>-4.457168213</td><td>R-HSA-199418</td><td>Reactome Gene Sets</td><td>Negative regulation of the PI3K/AKT network</td><td>9.864506307</td><td>6</td><td>HGF|NRG1|KIT|KITLG|PDGFRA|PIK3R3</td></tr>
<tr><td>-3.915213763</td><td>-4.273128549</td><td>hsa01521</td><td>KEGG Pathway</td><td>EGFR tyrosine kinase inhibitor resistance</td><td>12.43338816</td><td>5</td><td>HGF|NRG1|IL6|PDGFRA|PIK3R3</td></tr>
<tr><td>0</td><td>-4.019718346</td><td>hsa04151</td><td>KEGG Pathway</td><td>PI3K-Akt signaling pathway</td><td>4.945878162</td><td>9</td><td>HGF|IL6|KIT|LAMA4|KITLG|PDGFRA|SPP1|PIK3R3|ITGA8</td></tr>
<tr><td>0</td><td>-3.954391386</td><td>hsa04072</td><td>KEGG Pathway</td><td>Phospholipase D signaling pathway</td><td>8.010773578</td><td>6</td><td>CXCL8|KIT|KITLG|PDGFRA|PLA2G4A|PIK3R3</td></tr>
<tr><td>0</td><td>-3.835964754</td><td>hsa04010</td><td>KEGG Pathway</td><td>MAPK signaling pathway</td><td>5.304912281</td><td>8</td><td>DUSP6|HGF|IL1A|KIT|KITLG|PDGFRA|PLA2G4A|TGFB3</td></tr>
<tr><td>0</td><td>-3.424194902</td><td>R-HSA-1257604</td><td>Reactome Gene Sets</td><td>PIP3 activates AKT signaling</td><td>5.315036159</td><td>7</td><td>EGR1|HGF|NRG1|KIT|KITLG|PDGFRA|PIK3R3</td></tr>
<tr><td>-2.114832931</td><td>-3.232858478</td><td>hsa04510</td><td>KEGG Pathway</td><td>Focal adhesion</td><td>4.513808031</td><td>8</td><td>CCND2|HGF|IGF1R|LAMA4|PDGFRA|SPP1|PIK3R3|ITGA8</td></tr>
<tr><td>0</td><td>-3.32706956</td><td>R-HSA-5684996</td><td>Reactome Gene Sets</td><td>MAPK1/MAPK3 signaling</td><td>5.119630418</td><td>7</td><td>DUSP6|HGF|NRG1|IL6|KIT|KITLG|PDGFRA</td></tr>
<tr><td>0</td><td>-3.308531926</td><td>R-HSA-5663202</td><td>Reactome Gene Sets</td><td>Diseases of signal transduction by growth factor receptors and second messengers</td><td>3.961079413</td><td>9</td><td>ALK|DUSP6|HGF|NRG1|KIT|KITLG|PDGFRA|PIK3R3|DKK2</td></tr>
<tr><td>0</td><td>-3.043041832</td><td>R-HSA-9006925</td><td>Reactome Gene Sets</td><td>Intracellular signaling by second messengers</td><td>4.580721953</td><td>7</td><td>EGR1|HGF|NRG1|KIT|KITLG|PDGFRA|PIK3R3</td></tr>
<tr><td>-3.008945254</td><td>-2.222631901</td><td>hsa05218</td><td>KEGG Pathway</td><td>Melanoma</td><td>9.154014985</td><td>4</td><td>HGF|IGF1R|PDGFRA|PIK3R3</td></tr>
<tr><td>0</td><td>-2.985754617</td><td>R-HSA-5683057</td><td>Reactome Gene Sets</td><td>MAPK family signaling cascades</td><td>4.477618886</td><td>7</td><td>DUSP6|HGF|NRG1|IL6|KIT|KITLG|PDGFRA</td></tr>
<tr><td>0</td><td>-2.907212973</td><td>R-HSA-1433557</td><td>Reactome Gene Sets</td><td>Signaling by SCF-KIT</td><td>14.20958647</td><td>3</td><td>KIT|KITLG|PIK3R3</td></tr>
<tr><td>0</td><td>-2.875724131</td><td>hsa04014</td><td>KEGG Pathway</td><td>Ras signaling pathway</td><td>5.015148165</td><td>6</td><td>HGF|KIT|KITLG|PDGFRA|PLA2G4A|PIK3R3</td></tr>
<tr><td>0</td><td>-2.632569455</td><td>R-HSA-5673001</td><td>Reactome Gene Sets</td><td>RAF/MAP kinase cascade</td><td>4.487237831</td><td>6</td><td>DUSP6|HGF|NRG1|KIT|KITLG|PDGFRA</td></tr>
<tr><td>0</td><td>-2.441195</td><td>R-HSA-9856649</td><td>Reactome Gene Sets</td><td>Transcriptional and post-translational regulation of MITF-M expression and activity</td><td>9.783649698</td><td>3</td><td>EDNRB|KIT|KITLG</td></tr>
<tr><td>0</td><td>-2.351786981</td><td>hsa04015</td><td>KEGG Pathway</td><td>Rap1 signaling pathway</td><td>4.691844588</td><td>5</td><td>HGF|KIT|KITLG|PDGFRA|PIK3R3</td></tr>
<tr><td>0</td><td>-2.308500643</td><td>hsa05221</td><td>KEGG Pathway</td><td>Acute myeloid leukemia</td><td>8.776509288</td><td>3</td><td>DUSP6|KIT|PIK3R3</td></tr>
<tr><td>0</td><td>-2.256174345</td><td>hsa05230</td><td>KEGG Pathway</td><td>Central carbon metabolism in cancer</td><td>8.405670867</td><td>3</td><td>KIT|PDGFRA|PIK3R3</td></tr>
<tr><td>-4.728507601</td><td>-2.300233902</td><td>R-HSA-1630316</td><td>Reactome Gene Sets</td><td>Glycosaminoglycan metabolism</td><td>8.421902852</td><td>10</td><td>VCAN|CTSL|EXTL2|HAS2|HAS3|HS3ST3A1|ST3GAL6|GLCE|CHPF2|HS6ST3</td></tr>
<tr><td>-5.339592745</td><td>0</td><td>R-HSA-1638091</td><td>Reactome Gene Sets</td><td>Heparan sulfate/heparin (HS-GAG) metabolism</td><td>20.37326506</td><td>5</td><td>CTSL|EXTL2|HS3ST3A1|GLCE|HS6ST3</td></tr>
<tr><td>-4.920241843</td><td>0</td><td>hsa00534</td><td>KEGG Pathway</td><td>Glycosaminoglycan biosynthesis - heparan sulfate / heparin</td><td>27.84346225</td><td>4</td><td>EXTL2|HS3ST3A1|GLCE|HS6ST3</td></tr>
<tr><td>-2.649010036</td><td>-2.38340118</td><td>R-HSA-71387</td><td>Reactome Gene Sets</td><td>Metabolism of carbohydrates and carbohydrate derivatives</td><td>4.596837945</td><td>12</td><td>ALDH1A1|VCAN|CTSL|EXTL2|HAS2|HAS3|PFKFB3|HS3ST3A1|ST3GAL6|GLCE|CHPF2|HS6ST3</td></tr>
<tr><td>-4.352949527</td><td>0</td><td>R-HSA-2022928</td><td>Reactome Gene Sets</td><td>HS-GAG biosynthesis</td><td>20.24979072</td><td>4</td><td>EXTL2|HS3ST3A1|GLCE|HS6ST3</td></tr>
<tr><td>-5.184991043</td><td>-5.921273239</td><td>R-HSA-373076</td><td>Reactome Gene Sets</td><td>Class A/1 (Rhodopsin-like receptors)</td><td>6.493401531</td><td>11</td><td>EDNRB|CXCL1|CXCL2|HTR1D|CXCL8|PMCH|PTGDR|PTGER3|CCL2|CXCL5|NPFFR2</td></tr>
<tr><td>-5.244880578</td><td>-5.306466376</td><td>R-HSA-500792</td><td>Reactome Gene Sets</td><td>GPCR ligand binding</td><td>5.079171333</td><td>12</td><td>EDNRB|CXCL1|CXCL2|HTR1D|CXCL8|PMCH|PTGDR|PTGER3|CCL2|CXCL5|NPFFR2|TAS2R10</td></tr>
<tr><td>-5.201236806</td><td>-4.829257146</td><td>R-HSA-372790</td><td>Reactome Gene Sets</td><td>Signaling by GPCR</td><td>3.770059768</td><td>16</td><td>ABR|ADCYAP1|EDNRB|CXCL1|CXCL2|HTR1D|CXCL8|PLA2G4A|PTGER3|CCL2|CXCL5|PIK3R3|NPFFR2|TAS2R10|NLN|OXER1</td></tr>
<tr><td>-4.487222639</td><td>-5.035258268</td><td>R-HSA-375276</td><td>Reactome Gene Sets</td><td>Peptide ligand-binding receptors</td><td>7.839771843</td><td>8</td><td>EDNRB|CXCL1|CXCL2|CXCL8|PMCH|CCL2|CXCL5|NPFFR2</td></tr>
<tr><td>-4.525789581</td><td>-4.702790258</td><td>R-HSA-388396</td><td>Reactome Gene Sets</td><td>GPCR downstream signalling</td><td>4.092001166</td><td>13</td><td>EDNRB|CXCL1|CXCL2|HTR1D|CXCL8|PLA2G4A|PMCH|PTGDR|PTGER3|CXCL5|PIK3R3|NPFFR2|TAS2R10</td></tr>
<tr><td>-3.856520168</td><td>-4.440728033</td><td>R-HSA-418594</td><td>Reactome Gene Sets</td><td>G alpha (i) signalling events</td><td>5.612563933</td><td>9</td><td>CXCL1|CXCL2|HTR1D|CXCL8|PLA2G4A|PMCH|PTGER3|CXCL5|TAS2R10</td></tr>
<tr><td>0</td><td>-2.558964113</td><td>hsa04080</td><td>KEGG Pathway</td><td>Neuroactive ligand-receptor interaction</td><td>3.763620199</td><td>7</td><td>EDNRB|HTR1D|PMCH|PTGDR|PTGER3|NPFFR2|NPVF</td></tr>
<tr><td>0</td><td>-5.75014255</td><td>R-HSA-9927432</td><td>Reactome Gene Sets</td><td>Developmental Lineage of Mammary Gland Myoepithelial Cells</td><td>44.20760234</td><td>4</td><td>ACTA2|ALDH1A1|CNN1|MME</td></tr>
<tr><td>-2.026370755</td><td>-5.20140681</td><td>R-HSA-9734767</td><td>Reactome Gene Sets</td><td>Developmental Cell Lineages</td><td>7.524386198</td><td>9</td><td>ACTA2|ALDH1A1|CA2|CD74|CNN1|LAMA4|MME|TFRC|PROM1</td></tr>
<tr><td>0</td><td>-5.297177074</td><td>R-HSA-9924644</td><td>Reactome Gene Sets</td><td>Developmental Lineages of the Mammary Gland</td><td>34.597254</td><td>4</td><td>ACTA2|ALDH1A1|CNN1|MME</td></tr>
<tr><td>-2.312558945</td><td>-3.705409546</td><td>R-HSA-9734779</td><td>Reactome Gene Sets</td><td>Developmental Cell Lineages of the Integumentary System</td><td>10.04718235</td><td>5</td><td>ACTA2|ALDH1A1|CNN1|MME|TFRC</td></tr>
<tr><td>-5.430095815</td><td>-4.130394646</td><td>hsa05202</td><td>KEGG Pathway</td><td>Transcriptional misregulation in cancer</td><td>7.480333141</td><td>9</td><td>CCND2|DUSP6|ETV1|HPGD|IGF1R|IL6|CXCL8|MEIS1|PLAU</td></tr>
<tr><td>-4.352949527</td><td>-4.649415485</td><td>R-HSA-4085001</td><td>Reactome Gene Sets</td><td>Sialic acid metabolism</td><td>17.35422406</td><td>5</td><td>ST6GAL1|ST8SIA4|ST3GAL5|ST3GAL6|ST6GALNAC5</td></tr>
<tr><td>-3.720174991</td><td>0</td><td>R-HSA-9840309</td><td>Reactome Gene Sets</td><td>Glycosphingolipid biosynthesis</td><td>26.37801687</td><td>3</td><td>B3GALNT1|ST3GAL5|ST6GALNAC5</td></tr>
<tr><td>-3.199006493</td><td>-3.484927383</td><td>R-HSA-446219</td><td>Reactome Gene Sets</td><td>Synthesis of substrates in N-glycan biosythesis</td><td>8.810606061</td><td>5</td><td>ST6GAL1|ST8SIA4|ST3GAL5|ST3GAL6|ST6GALNAC5</td></tr>
<tr><td>-2.860744169</td><td>-3.141765061</td><td>R-HSA-446193</td><td>Reactome Gene Sets</td><td>Biosynthesis of the N-glycan precursor (dolichol lipid-linked oligosaccharide, LLO) and transfer to a nascent protein</td><td>7.158617424</td><td>5</td><td>ST6GAL1|ST8SIA4|ST3GAL5|ST3GAL6|ST6GALNAC5</td></tr>
<tr><td>-2.401304077</td><td>0</td><td>R-HSA-428157</td><td>Reactome Gene Sets</td><td>Sphingolipid metabolism</td><td>6.24526256</td><td>4</td><td>B3GALNT1|ST3GAL5|ST6GALNAC5|SGMS2</td></tr>
<tr><td>-2.291386564</td><td>0</td><td>R-HSA-1660662</td><td>Reactome Gene Sets</td><td>Glycosphingolipid metabolism</td><td>8.64107449</td><td>3</td><td>B3GALNT1|ST3GAL5|ST6GALNAC5</td></tr>
<tr><td>0</td><td>-4.678342198</td><td>hsa05321</td><td>KEGG Pathway</td><td>Inflammatory bowel disease</td><td>15.07077352</td><td>5</td><td>IL1A|IL6|RORA|TGFB3|IL18R1</td></tr>
<tr><td>-2.506566439</td><td>-3.812329234</td><td>hsa04640</td><td>KEGG Pathway</td><td>Hematopoietic cell lineage</td><td>8.017651515</td><td>7</td><td>CD59|IL1A|IL6|KIT|KITLG|MME|TFRC</td></tr>
<tr><td>0</td><td>-2.427447652</td><td>hsa05010</td><td>KEGG Pathway</td><td>Alzheimer disease</td><td>2.929357514</td><td>10</td><td>IL1A|IL6|LPL|MME|PTGS2|VDAC2|PIK3R3|DKK2|NOX4|SLC39A10</td></tr>
<tr><td>0</td><td>-4.451314772</td><td>R-HSA-1474244</td><td>Reactome Gene Sets</td><td>Extracellular matrix organization</td><td>5.630213505</td><td>9</td><td>ACTA2|BMP4|VCAN|LAMA4|P4HA1|SPP1|TGFB3|MFAP5|ITGA8</td></tr>
<tr><td>0</td><td>-4.448298639</td><td>R-HSA-2129379</td><td>Reactome Gene Sets</td><td>Molecules associated with elastic fibres</td><td>21.50640114</td><td>4</td><td>BMP4|TGFB3|MFAP5|ITGA8</td></tr>
<tr><td>0</td><td>-4.147264317</td><td>R-HSA-1566948</td><td>Reactome Gene Sets</td><td>Elastic fibre formation</td><td>18.08492823</td><td>4</td><td>BMP4|TGFB3|MFAP5|ITGA8</td></tr>
<tr><td>0</td><td>-3.809744548</td><td>R-HSA-9830674</td><td>Reactome Gene Sets</td><td>Formation of the ureteric bud</td><td>28.41917293</td><td>3</td><td>BMP4|ITGA8|GREM1</td></tr>
<tr><td>0</td><td>-3.225893034</td><td>R-HSA-3000178</td><td>Reactome Gene Sets</td><td>ECM proteoglycans</td><td>10.47022161</td><td>4</td><td>VCAN|LAMA4|TGFB3|ITGA8</td></tr>
<tr><td>0</td><td>-2.797146245</td><td>hsa05410</td><td>KEGG Pathway</td><td>Hypertrophic cardiomyopathy</td><td>8.03774588</td><td>4</td><td>IL6|SLC8A1|TGFB3|ITGA8</td></tr>
<tr><td>0</td><td>-2.79225681</td><td>R-HSA-9830369</td><td>Reactome Gene Sets</td><td>Kidney development</td><td>12.97397025</td><td>3</td><td>BMP4|ITGA8|GREM1</td></tr>
<tr><td>0</td><td>-2.629897632</td><td>hsa04350</td><td>KEGG Pathway</td><td>TGF-beta signaling pathway</td><td>7.233971292</td><td>4</td><td>BMP4|TGFB3|FST|GREM1</td></tr>
<tr><td>-3.28920312</td><td>0</td><td>R-HSA-425393</td><td>Reactome Gene Sets</td><td>Transport of inorganic cations/anions and amino acids/oligopeptides</td><td>7.355643592</td><td>7</td><td>SLC26A4|SLC1A1|SLC6A6|SLC8A1|SLC20A2|SLC36A4|SLC43A2</td></tr>
<tr><td>-2.996364529</td><td>0</td><td>R-HSA-352230</td><td>Reactome Gene Sets</td><td>Amino acid transport across the plasma membrane</td><td>15.18734304</td><td>3</td><td>SLC6A6|SLC36A4|SLC43A2</td></tr>
<tr><td>-3.421521738</td><td>-2.915995491</td><td>hsa04218</td><td>KEGG Pathway</td><td>Cellular senescence</td><td>5.836325034</td><td>8</td><td>CCND2|IL1A|IL6|CXCL8|TGFB3|VDAC2|PIK3R3|CCNB3</td></tr>
<tr><td>-3.705916735</td><td>-2.266921709</td><td>hsa05162</td><td>KEGG Pathway</td><td>Measles</td><td>7.211256409</td><td>6</td><td>CCND2|IL6|MX1|NFKBIA|PIK3R3|TLR9</td></tr>
<tr><td>-2.899684678</td><td>0</td><td>hsa04068</td><td>KEGG Pathway</td><td>FoxO signaling pathway</td><td>5.167122351</td><td>6</td><td>CCND2|IGF1R|IL6|TGFB3|PIK3R3|CCNB3</td></tr>
<tr><td>-2.35859101</td><td>0</td><td>hsa04066</td><td>KEGG Pathway</td><td>HIF-1 signaling pathway</td><td>5.206267218</td><td>5</td><td>IGF1R|IL6|PFKFB3|TFRC|PIK3R3</td></tr>
<tr><td>-2.45805431</td><td>0</td><td>hsa04630</td><td>KEGG Pathway</td><td>JAK-STAT signaling pathway</td><td>4.97204683</td><td>5</td><td>CCND2|IL6|PDGFRA|PIK3R3|IL24</td></tr>
<tr><td>0</td><td>-2.251821094</td><td>hsa05166</td><td>KEGG Pathway</td><td>Human T-cell leukemia virus 1 infection</td><td>3.579308712</td><td>7</td><td>CCND2|EGR1|IL6|NFKBIA|TGFB3|VDAC2|PIK3R3</td></tr>
<tr><td>-2.106114521</td><td>0</td><td>hsa05169</td><td>KEGG Pathway</td><td>Epstein-Barr virus infection</td><td>4.094626801</td><td>5</td><td>CCND2|IL6|NFKBIA|TRAF5|PIK3R3</td></tr>
<tr><td>-2.424968105</td><td>-3.861028689</td><td>hsa04913</td><td>KEGG Pathway</td><td>Ovarian steroidogenesis</td><td>11.01325758</td><td>5</td><td>CYP1B1|HSD3B1|IGF1R|PLA2G4A|PTGS2</td></tr>
<tr><td>-3.170093395</td><td>0</td><td>R-HSA-556833</td><td>Reactome Gene Sets</td><td>Metabolism of lipids</td><td>2.723489852</td><td>18</td><td>BCHE|BDH1|CYP1B1|HPGD|HSD3B1|PLA2G4A|PTGS2|RORA|SCD|PIK3R3|B3GALNT1|ST3GAL5|FADS2|GLIPR1|ELOVL6|PLBD1|ST6GALNAC5|SGMS2</td></tr>
<tr><td>-2.382835834</td><td>0</td><td>R-HSA-8978868</td><td>Reactome Gene Sets</td><td>Fatty acid metabolism</td><td>4.581515152</td><td>7</td><td>CYP1B1|HPGD|PLA2G4A|PTGS2|SCD|FADS2|ELOVL6</td></tr>
<tr><td>0</td><td>-2.482198348</td><td>R-HSA-2142753</td><td>Reactome Gene Sets</td><td>Arachidonate metabolism</td><td>7.765279918</td><td>4</td><td>CYP1B1|HPGD|PLA2G4A|PTGS2</td></tr>
<tr><td>0</td><td>-2.256174345</td><td>hsa05204</td><td>KEGG Pathway</td><td>Chemical carcinogenesis - DNA adducts</td><td>8.405670867</td><td>3</td><td>CYP1B1|GSTA3|PTGS2</td></tr>
<tr><td>0</td><td>-3.655873573</td><td>R-HSA-8957275</td><td>Reactome Gene Sets</td><td>Post-translational protein phosphorylation</td><td>9.209917154</td><td>5</td><td>BMP4|VCAN|IL6|SPP1|CKAP4</td></tr>
<tr><td>0</td><td>-3.362489355</td><td>R-HSA-381426</td><td>Reactome Gene Sets</td><td>Regulation of Insulin-like Growth Factor (IGF) transport and uptake by Insulin-like Growth Factor Binding Proteins (IGFBPs)</td><td>7.957368421</td><td>5</td><td>BMP4|VCAN|IL6|SPP1|CKAP4</td></tr>
<tr><td>-3.344707579</td><td>-2.688444502</td><td>hsa05215</td><td>KEGG Pathway</td><td>Prostate cancer</td><td>7.880225164</td><td>5</td><td>IGF1R|NFKBIA|PDGFRA|PLAU|PIK3R3</td></tr>
<tr><td>0</td><td>-2.930033139</td><td>hsa05206</td><td>KEGG Pathway</td><td>MicroRNAs in cancer</td><td>4.379054949</td><td>7</td><td>CYP1B1|PDGFRA|PLAU|PTGS2|PIK3R3|SPRY2|MIR23B</td></tr>
<tr><td>-2.106114521</td><td>0</td><td>hsa05205</td><td>KEGG Pathway</td><td>Proteoglycans in cancer</td><td>4.094626801</td><td>5</td><td>CTSL|HGF|IGF1R|PLAU|PIK3R3</td></tr>
<tr><td>-3.255665753</td><td>0</td><td>hsa01040</td><td>KEGG Pathway</td><td>Biosynthesis of unsaturated fatty acids</td><td>18.56230816</td><td>3</td><td>SCD|FADS2|ELOVL6</td></tr>
<tr><td>-2.312558945</td><td>0</td><td>hsa01212</td><td>KEGG Pathway</td><td>Fatty acid metabolism</td><td>8.792672288</td><td>3</td><td>SCD|FADS2|ELOVL6</td></tr>
<tr><td>-2.842679818</td><td>-3.1812282</td><td>hsa04210</td><td>KEGG Pathway</td><td>Apoptosis</td><td>7.260372647</td><td>5</td><td>CTSC|LMNB1|NFKBIA|PIK3R3|HRK</td></tr>
<tr><td>0</td><td>-3.162355586</td><td>hsa05140</td><td>KEGG Pathway</td><td>Leishmaniasis</td><td>10.07261825</td><td>4</td><td>IL1A|NFKBIA|PTGS2|TGFB3</td></tr>
<tr><td>0</td><td>-2.897405914</td><td>hsa05222</td><td>KEGG Pathway</td><td>Small cell lung cancer</td><td>8.55631013</td><td>4</td><td>LAMA4|NFKBIA|PTGS2|PIK3R3</td></tr>
<tr><td>0</td><td>-2.703471012</td><td>hsa04625</td><td>KEGG Pathway</td><td>C-type lectin receptor signaling pathway</td><td>7.578446115</td><td>4</td><td>IL6|NFKBIA|PTGS2|PIK3R3</td></tr>
<tr><td>-2.606021666</td><td>0</td><td>hsa04024</td><td>KEGG Pathway</td><td>cAMP signaling pathway</td><td>4.435241774</td><td>6</td><td>ADCYAP1|HTR1D|NFKBIA|PLN|PTGER3|PIK3R3</td></tr>
<tr><td>-2.596506346</td><td>-2.227844612</td><td>hsa05208</td><td>KEGG Pathway</td><td>Chemical carcinogenesis - reactive oxygen species</td><td>4.415703264</td><td>6</td><td>GSTA3|HGF|NFKBIA|VDAC2|PIK3R3|NOX4</td></tr>
<tr><td>0</td><td>-2.482198348</td><td>hsa04923</td><td>KEGG Pathway</td><td>Regulation of lipolysis in adipocytes</td><td>10.11529884</td><td>3</td><td>PTGER3|PTGS2|PIK3R3</td></tr>
<tr><td>0</td><td>-2.461509586</td><td>hsa04370</td><td>KEGG Pathway</td><td>VEGF signaling pathway</td><td>9.946710526</td><td>3</td><td>PLA2G4A|PTGS2|PIK3R3</td></tr>
<tr><td>-2.104588789</td><td>-2.369484881</td><td>hsa04926</td><td>KEGG Pathway</td><td>Relaxin signaling pathway</td><td>6.121052632</td><td>4</td><td>ACTA2|EDNRB|NFKBIA|PIK3R3</td></tr>
<tr><td>0</td><td>-2.273340318</td><td>hsa05211</td><td>KEGG Pathway</td><td>Renal cell carcinoma</td><td>8.52575188</td><td>3</td><td>HGF|TGFB3|PIK3R3</td></tr>
<tr><td>0</td><td>-2.223782487</td><td>hsa04380</td><td>KEGG Pathway</td><td>Osteoclast differentiation</td><td>5.564593301</td><td>4</td><td>IL1A|NFKBIA|TNFRSF11B|PIK3R3</td></tr>
<tr><td>0</td><td>-2.222631901</td><td>hsa05223</td><td>KEGG Pathway</td><td>Non-small cell lung cancer</td><td>8.175378515</td><td>3</td><td>ALK|HGF|PIK3R3</td></tr>
<tr><td>0</td><td>-2.158506998</td><td>hsa05220</td><td>KEGG Pathway</td><td>Chronic myeloid leukemia</td><td>7.750683527</td><td>3</td><td>NFKBIA|TGFB3|PIK3R3</td></tr>
<tr><td>-2.549956811</td><td>0</td><td>hsa00514</td><td>KEGG Pathway</td><td>Other types of O-glycan biosynthesis</td><td>9.747904578</td><td>4</td><td>MFNG|ST6GAL1|GALNT16|GXYLT2</td></tr>
<tr><td>0</td><td>-3.110951831</td><td>hsa05418</td><td>KEGG Pathway</td><td>Fluid shear stress and atherosclerosis</td><td>7.004725723</td><td>5</td><td>BMP4|GSTA3|IL1A|CCL2|PIK3R3</td></tr>
<tr><td>0</td><td>-2.738767535</td><td>R-HSA-9925563</td><td>Reactome Gene Sets</td><td>Developmental Lineage of Pancreatic Ductal Cells</td><td>9.544823232</td><td>4</td><td>CA2|CD74|LAMA4|PROM1</td></tr>
<tr><td>0</td><td>-2.054758765</td><td>R-HSA-9820448</td><td>Reactome Gene Sets</td><td>Developmental Cell Lineages of the Exocrine Pancreas</td><td>5.454184704</td><td>4</td><td>CA2|CD74|LAMA4|PROM1</td></tr>
<tr><td>-3.019889442</td><td>0</td><td>R-HSA-6809371</td><td>Reactome Gene Sets</td><td>Formation of the cornified envelope</td><td>6.682430939</td><td>5</td><td>CDSN|DSC3|KRT6B|KRT8|KRT73</td></tr>
<tr><td>-2.038229324</td><td>0</td><td>R-HSA-6805567</td><td>Reactome Gene Sets</td><td>Keratinization</td><td>3.940112582</td><td>5</td><td>CDSN|DSC3|KRT6B|KRT8|KRT73</td></tr>
<tr><td>-2.291386564</td><td>0</td><td>hsa00240</td><td>KEGG Pathway</td><td>Pyrimidine metabolism</td><td>7.899164054</td><td>4</td><td>DUT|RRM2|NT5C2|HDDC2</td></tr>
<tr><td>-2.604092896</td><td>0</td><td>R-HSA-445355</td><td>Reactome Gene Sets</td><td>Smooth Muscle Contraction</td><td>11.1373849</td><td>3</td><td>ACTA2|ANXA6|PDE5A</td></tr>
<tr><td>-2.097449031</td><td>0</td><td>R-HSA-397014</td><td>Reactome Gene Sets</td><td>Muscle contraction</td><td>4.074653012</td><td>5</td><td>ACTA2|ANXA6|MME|PLN|PDE5A</td></tr>
<tr><td>0</td><td>-2.441195</td><td>hsa04978</td><td>KEGG Pathway</td><td>Mineral absorption</td><td>9.783649698</td><td>3</td><td>SLC8A1|STEAP1|STEAP2</td></tr>
<tr><td>0</td><td>-2.36343946</td><td>hsa04929</td><td>KEGG Pathway</td><td>GnRH secretion</td><td>9.181578947</td><td>3</td><td>SPP1|PIK3R3|HCN1</td></tr>
<tr><td>-2.357669785</td><td>-2.028214775</td><td>hsa04020</td><td>KEGG Pathway</td><td>Calcium signaling pathway</td><td>3.946317484</td><td>6</td><td>EDNRB|HGF|PDGFRA|PLN|PTGER3|VDAC2</td></tr>
<tr><td>0</td><td>-2.256002725</td><td>hsa04371</td><td>KEGG Pathway</td><td>Apelin signaling pathway</td><td>5.683834586</td><td>4</td><td>ACTA2|EGR1|SLC8A1|SPP1</td></tr>
<tr><td>-2.255427328</td><td>0</td><td>R-HSA-9012999</td><td>Reactome Gene Sets</td><td>RHO GTPase cycle</td><td>3.003339748</td><td>8</td><td>ABR|LMNB1|TFRC|PIK3R3|PDE5A|AKAP12|RHOBTB1|CKAP4</td></tr>
</table>
