# Supplementary material for: Development and characterization of Hanwoo oviduct epithelial cell line for heat stress study
Source: Anim Biosci. 2026 Sep 1;39(11):250962. doi: 10.5713/ab.250962 (PMC13740644; doi:10.5713/ab.250962)
Supplement: Supplementary file 8 [file ab-250962-Supplementary-16.pdf]

Supplement 16. Oviduct epithelial cells Upregulated DEP Pathway Enrichment (KEGG and Reactome).

_LogP_HS_REC	_LogP_REC_CON	GO	Category	Description	Enrichment	#GeneInGOAndHitList	Hits
-3.427028579	-14.66903875	hsa04382	KEGG Pathway	Cornified envelope formation	16.8903784	16	DSP|HAL|KRT1|KRT2|KRT3|KRT5|KRT10|KRT14|KRT17|KRT19|S100A10|S100A11|KRT75|KRT76|KRT24|KRT73
0	-12.47690356	R-HSA-6809371	Reactome Gene Sets	Formation of the cornified envelope	21.9912727	12	KRT1|KRT2|KRT3|KRT5|KRT10|KRT14|KRT17|KRT19|KRT75|KRT76|KRT24|KRT73
0	-9.763720117	R-HSA-6805567	Reactome Gene Sets	Keratinization	12.9665523	12	KRT1|KRT2|KRT3|KRT5|KRT10|KRT14|KRT17|KRT19|KRT75|KRT76|KRT24|KRT73
0	-7.74739584	R-HSA-9725554	Reactome Gene Sets	Differentiation of Keratinocytes in Interfollicular Epidermis in Mammalian Skin	35.2424242	6	KRT1|KRT5|KRT10|KRT14|KRT19|KRT24
0	-6.77994743	R-HSA-9734767	Reactome Gene Sets	Developmental Cell Lineages	13.3766866	8	KRT1|KRT5|KRT10|KRT14|KRT17|KRT19|HNF1B|KRT24
0	-6.729118338	R-HSA-9734779	Reactome Gene Sets	Developmental Cell Lineages of the Integumentary System	24.1132376	6	KRT1|KRT5|KRT10|KRT14|KRT19|KRT24
-3.187846269	-4.462503558	hsa04915	KEGG Pathway	Estrogen signaling pathway	9.25700291	8	CALM1|HSPA1L|HSPA6|KRT10|KRT14|KRT17|KRT19|KRT24
0	-4.059627529	hsa05150	KEGG Pathway	Staphylococcus aureus infection	11.2292038	5	KRT10|KRT14|KRT17|KRT19|KRT24
-9.225270288	-5.69810032	R-HSA-2262752	Reactome Gene Sets	Cellular responses to stress	4.88629141	24	ALB|APOA1|ASNS|CDKN2A|CEBPB|CRYAB|HSPA1L|HSPA6|SKP1|ELOC|ZBTB17|ATP6V0D1|ATP6V1F|COX5A|BAG5|BAG3|BAG2|HSPH1|SLC7A11|TXN2|HSPB8|TNRC6C|CRTC3|CDC26
-2.62989938	0	R-HSA-9711123	Reactome Gene Sets	Cellular response to chemical stress	4.89869316	6	ALB|CDKN2A|SKP1|COX5A|SLC7A11|TXN2
-8.244603152	-3.215202463	R-HSA-3371556	Reactome Gene Sets	Cellular response to heat stress	28.2975936	7	CRYAB|HSPA1L|HSPA6|BAG3|BAG2|HSPH1|HSPB8
-6.274355397	0	hsa05134	KEGG Pathway	Legionellosis	31.762605	5	BNIP3|HSPA1L|HSPA6|NFKBIA|BCL2L13
-6.254138832	-2.166904946	hsa04141	KEGG Pathway	Protein processing in endoplasmic reticulum	14.5625043	7	CRYAB|DNAJB2|HSPA1L|HSPA6|SKP1|BAG2|HSPH1
-5.849839427	0	R-HSA-3371453	Reactome Gene Sets	Regulation of HSF1-mediated heat shock response	26.1574394	5	HSPA1L|HSPA6|BAG3|BAG2|HSPH1
-4.381178805	0	R-HSA-3371571	Reactome Gene Sets	HSF1-dependent transactivation	44.4676471	3	CRYAB|HSPA1L|HSPB8
-3.187846269	0	hsa05162	KEGG Pathway	Measles	10.2371562	4	BID|HSPA1L|HSPA6|NFKBIA
-3.143122996	0	hsa04213	KEGG Pathway	Longevity regulating pathway - multiple species	17.2132827	3	CRYAB|HSPA1L|HSPA6
-2.406859488	0	hsa05145	KEGG Pathway	Toxoplasmosis	9.52878151	3	HSPA1L|HSPA6|NFKBIA
-2.101966564	0	hsa05020	KEGG Pathway	Prion disease	5.11857808	4	HSPA1L|HSPA6|PPP3R1|COX5A
0	-6.9820526	R-HSA-114608	Reactome Gene Sets	Platelet degranulation	14.2062485	8	ALB|APOA1|KNG1|ORM1|SERPINA1|SERPINF2|TF|TGFB1
0	-6.854189074	R-HSA-76005	Reactome Gene Sets	Response to elevated platelet cytosolic Ca2+	13.6761646	8	ALB|APOA1|KNG1|ORM1|SERPINA1|SERPINF2|TF|TGFB1
0	-6.303213521	R-HSA-8957275	Reactome Gene Sets	Post-translational protein phosphorylation	11.9141056	8	AFP|ALB|APOA1|APOA2|KNG1|SERPINA1|SDC2|TF
0	-6.21593752	R-HSA-109582	Reactome Gene Sets	Hemostasis	5.1643488	14	ALB|APOA1|KNG1|ORM1|SERPINA1|SERPINF2|S100A10|SDC2|SLC3A2|TF|TGFB1|GNA13|SLC7A11|SH2B1
0	-5.873547116	R-HSA-381426	Reactome Gene Sets	Regulation of Insulin-like Growth Factor (IGF) transport and uptake by Insulin-like Growth Factor Binding Proteins (IGFBPs)	10.2937872	8	AFP|ALB|APOA1|APOA2|KNG1|SERPINA1|SDC2|TF
0	-5.618389667	R-HSA-76002	Reactome Gene Sets	Platelet activation, signaling and aggregation	7.86901457	9	ALB|APOA1|KNG1|ORM1|SERPINA1|SERPINF2|TF|TGFB1|GNA13
0	-2.166835029	hsa04610	KEGG Pathway	Complement and coagulation cascades	7.80940083	3	KNG1|SERPINA1|SERPINF2
-2.754778444	-3.815138057	hsa05152	KEGG Pathway	Tuberculosis	7.06990881	8	BID|CALM1|CEBPB|PPP3CB|PPP3R1|TGFB1|ATP6V0D1|ATP6V0A2
-4.504019671	0	hsa05170	KEGG Pathway	Human immunodeficiency virus 1 infection	10.0208782	6	BID|CALM1|NFKBIA|PPP3R1|SKP1|ELOC
-4.470051618	0	hsa05417	KEGG Pathway	Lipid and atherosclerosis	9.88169935	6	BID|CALM1|HSPA1L|HSPA6|NFKBIA|PPP3R1
-4.409624608	0	R-HSA-1168372	Reactome Gene Sets	Downstream signaling events of B Cell Receptor (BCR)	21.2382792	4	CALM1|NFKBIA|PPP3R1|SKP1
-4.024449439	0	R-HSA-5607764	Reactome Gene Sets	CLEC7A (Dectin-1) signaling	16.940056	4	CALM1|NFKBIA|PPP3R1|SKP1
-3.799620393	0	R-HSA-983705	Reactome Gene Sets	Signaling by the B Cell Receptor (BCR)	14.822549	4	CALM1|NFKBIA|PPP3R1|SKP1
-3.527952345	0	R-HSA-2454202	Reactome Gene Sets	Fc epsilon receptor (FCERI) signaling	12.592608	4	CALM1|NFKBIA|PPP3R1|SKP1
-3.348380093	0	R-HSA-5621481	Reactome Gene Sets	C-type lectin receptors (CLRs)	11.2933707	4	CALM1|NFKBIA|PPP3R1|SKP1
-3.346533193	0	hsa05163	KEGG Pathway	Human cytomegalovirus infection	7.87038001	5	BID|CALM1|CDKN2A|NFKBIA|PPP3R1
-2.990737192	0	hsa04218	KEGG Pathway	Cellular senescence	6.14676785	6	CALM1|CDKN2A|PPP3CB|PPP3R1|TGFB1|TRAF3IP2
-2.429968814	0	hsa05166	KEGG Pathway	Human T-cell leukemia virus 1 infection	5.0262633	7	CDKN2A|NFKBIA|PPP3CB|PPP3R1|TGFB1|CRTC3|CDC26
-2.462546093	0	hsa04922	KEGG Pathway	Glucagon signaling pathway	7.51590774	5	CALM1|PCK2|PKM|PPP3CB|PPP3R1
-2.146721342	0	hsa04114	KEGG Pathway	Oocyte meiosis	5.78562682	5	CALM1|PPP3CB|PPP3R1|SKP1|CDC26
-2.637888497	0	hsa05167	KEGG Pathway	Kaposi sarcoma-associated herpesvirus infection	7.26002401	4	BID|CALM1|NFKBIA|PPP3R1
-2.485617188	0	hsa04625	KEGG Pathway	C-type lectin receptor signaling pathway	10.1640336	3	CALM1|NFKBIA|PPP3R1
-2.304235227	0	hsa05010	KEGG Pathway	Alzheimer disease	4.5491199	5	BID|CALM1|PPP3R1|COX5A|AMBRA1
-3.227990089	-3.9114245	R-HSA-917937	Reactome Gene Sets	Iron uptake and transport	13.8655539	5	SKP1|TF|ATP6V0D1|ATP6V1F|ATP6V0A2
0	-3.475972521	R-HSA-917977	Reactome Gene Sets	Transferrin endocytosis and recycling	20.7536033	4	TF|ATP6V0D1|ATP6V1F|ATP6V0A2
-2.971692694	0	hsa05120	KEGG Pathway	Epithelial cell signaling in Helicobacter pylori infection	9.06143242	4	NFKBIA|ATP6V0D1|ATP6V1F|ATP6V0A2
-2.155326658	0	hsa00190	KEGG Pathway	Oxidative phosphorylation	5.82755165	5	ATP5PF|ATP6V0D1|ATP6V1F|COX5A|ATP6V0A2
0	-2.075576753	hsa05323	KEGG Pathway	Rheumatoid arthritis	6.77222844	4	TGFB1|ATP6V0D1|ATP6V1F|ATP6V0A2
-2.621955654	-2.742728677	R-HSA-196854	Reactome Gene Sets	Metabolism of vitamins and cofactors	6.49860305	8	APOA1|APOA2|CALM1|CYB5A|SDC2|RDH11|PPCDC|ALDH1L2
0	-3.024660848	R-HSA-975634	Reactome Gene Sets	Retinoid metabolism and transport	14.6218569	4	APOA1|APOA2|SDC2|RDH11
0	-2.914256767	R-HSA-6806667	Reactome Gene Sets	Metabolism of fat-soluble vitamins	13.4033688	4	APOA1|APOA2|SDC2|RDH11
-2.521132749	0	R-HSA-2187338	Reactome Gene Sets	Visual phototransduction	7.88433458	5	APOA1|APOA2|CALM1|SDC2|RDH11
0	-2.359899332	R-HSA-174824	Reactome Gene Sets	Plasma lipoprotein assembly, remodeling, and clearance	8.57815603	4	ALB|APOA1|APOA2|NPC1
0	-2.343781071	hsa03320	KEGG Pathway	PPAR signaling pathway	8.46528555	4	PLIN2|APOA1|APOA2|PCK2
-3.028083846	-3.491215604	R-HSA-382551	Reactome Gene Sets	Transport of small molecules	3.36017602	15	ALB|APOA1|APOA2|CALM1|NPC1|SKP1|SLC1A4|SLC3A2|TF|ATP6V0D1|ATP6V1F|SLC12A6|ATP6V0A2|ARL2BP|SLC7A11
0	-3.394596637	R-HSA-352230	Reactome Gene Sets	Amino acid transport across the plasma membrane	20.8250689	3	SLC1A4|SLC3A2|SLC7A11
-2.439938572	-2.868174446	R-HSA-425393	Reactome Gene Sets	Transport of inorganic cations/anions and amino acids/oligopeptides	7.37800117	5	CALM1|SLC1A4|SLC3A2|SLC12A6|SLC7A11
0	-3.083975289	hsa04216	KEGG Pathway	Ferroptosis	16.3625541	3	SLC3A2|TF|SLC7A11
-2.286347205	0	R-HSA-425407	Reactome Gene Sets	SLC-mediated transmembrane transport	3.92293721	6	CALM1|SLC1A4|SLC3A2|SLC12A6|ARL2BP|SLC7A11
-4.265604183	0	R-HSA-6798695	Reactome Gene Sets	Neutrophil degranulation	6.05516896	8	CSTB|HSPA6|MIF|PKM|S100A11|COMMD3|GCA|COMMD9
0	-3.178872204	R-HSA-8853884	Reactome Gene Sets	Transcriptional Regulation by VENTX	16.4964539	4	CDKN2A|CEBPB|TNRC6C|CDC26
0	-3.434605066	R-HSA-9725371	Reactome Gene Sets	Nuclear events stimulated by ALK signaling in cancer	21.4758523	3	CEBPB|SKP1|TNRC6C
0	-2.139957564	R-HSA-9700206	Reactome Gene Sets	Signaling by ALK in cancer	7.63585859	3	CEBPB|SKP1|TNRC6C
0	-2.139957564	R-HSA-9725370	Reactome Gene Sets	Signaling by ALK fusions and activated point mutants	7.63585859	3	CEBPB|SKP1|TNRC6C
0	-2.359899332	hsa01230	KEGG Pathway	Biosynthesis of amino acids	10.722695	5	ASNS|PKM|PSPH|RPE|PSAT1
0	-2.339228156	R-HSA-71291	Reactome Gene Sets	Metabolism of amino acids and derivatives	3.90470041	6	ASNS|HAL|PSPH|SLC3A2|PSAT1|PSTK
-3.286363234	0	R-HSA-8951664	Reactome Gene Sets	Neddylation	5.52241804	8	SKP1|ELOC|UCHL3|DCAF5|COMMD3|COMMD9|COMMD8|KBTBD6
-3.649900374	0	hsa04137	KEGG Pathway	Mitophagy - animal	13.5520448	4	BNIP3|TAX1BP1|BCL2L13|AMBRA1
0	-3.298984235	R-HSA-556833	Reactome Gene Sets	Metabolism of lipids	2.97459175	14	PLIN2|ALB|APOA1|APOA2|CYP3A4|GC|STARD10|MED18|ACOT13|LPIN3|PLEKHA3|FDX2|ARSI|PLPP6
-3.115266236	-2.042330749	hsa05200	KEGG Pathway	Pathways in cancer	3.31940841	11	BID|CALM1|CDKN2A|KNG1|NFKBIA|SKP1|ELOC|TGFB1|TRAF1|ZBTB17|GNA13
0	-2.286759264	hsa04110	KEGG Pathway	Cell cycle	5.08988688	5	CDKN2A|SKP1|TGFB1|ZBTB17|CDC26
-3.140517582	0	hsa05132	KEGG Pathway	Salmonella infection	7.08647762	5	NFKBIA|S100A10|SKP1|TXN2|DYNLRB1
0	-3.124858443	R-HSA-381119	Reactome Gene Sets	Unfolded Protein Response (UPR)	9.85272076	4	ASNS|CEBPB|ZBTB17|ATP6V0D1
0	-2.914256767	R-HSA-9925563	Reactome Gene Sets	Developmental Lineage of Pancreatic Ductal Cells	14.3172348	3	KRT17|KRT19|HNF1B
0	-2.222686089	R-HSA-9820448	Reactome Gene Sets	Developmental Cell Lineages of the Exocrine Pancreas	8.18127706	3	KRT17|KRT19|HNF1B
0	-2.529611935	R-HSA-202733	Reactome Gene Sets	Cell surface interactions at the vascular wall	5.9570528	5	MIF|SDC2|SLC3A2|TGFB1|SLC7A11
0	-2.281563047	R-HSA-3000171	Reactome Gene Sets	Non-integrin membrane-ECM interactions	8.59034091	3	COL10A1|SDC2|TGFB1
-2.570286792	0	R-HSA-9833110	Reactome Gene Sets	RSV-host interactions	10.890036	3	SDC2|ELOC|MED18
-2.313135663	0	R-HSA-9820952	Reactome Gene Sets	Respiratory Syncytial Virus Infection Pathway	8.82002917	3	SDC2|ELOC|MED18
-2.121311391	0	hsa05418	KEGG Pathway	Fluid shear stress and atherosclerosis	7.51565866	3	CALM1|SDC2|TXN2
-2.567582555	0	hsa05203	KEGG Pathway	Viral carcinogenesis	6.94129125	4	CDKN2A|NFKBIA|PKM|ATP6V0D1
-2.5079484	0	R-HSA-162906	Reactome Gene Sets	HIV Infection	6.68058547	4	POLR2F|SKP1|ELOC|BANF1
-2.385338382	0	R-HSA-162909	Reactome Gene Sets	Host Interactions of HIV factors	9.36160991	3	SKP1|ELOC|BANF1
-2.080258372	0	R-HSA-162587	Reactome Gene Sets	HIV Life Cycle	7.26002401	3	POLR2F|ELOC|BANF1
-2.204417115	0	R-HSA-1280215	Reactome Gene Sets	Cytokine Signaling in Immune system	2.48722308	12	EIF4A2|HSPA1L|IFIT1|ISG20|MIF|MT2A|NFKBIA|SKP1|ELOC|TGFB1|SH2B1|BOLA2B
-2.164001295	0	hsa04210	KEGG Pathway	Apoptosis	7.78995277	3	BID|NFKBIA|DIABLO
-2.003870683	0	R-HSA-1852241	Reactome Gene Sets	Organelle biogenesis and maintenance	4.79112696	4	CALM1|SSNA1|CRTC3|DYNLRB1
