# Supplementary material for: Development and characterization of Hanwoo oviduct epithelial cell line for heat stress study
Source: Anim Biosci. 2026 Sep 1;39(11):250962. doi: 10.5713/ab.250962 (PMC13740644; doi:10.5713/ab.250962)
Supplement: Supplementary file 9 [file ab-250962-Supplementary-17.pdf]

**Supplement 17.** Oviduct epithelial cells Downregulated DEP Pathway Enrichment (KEGG and Reactome).

<table>
<tr><th>_LogP_HS_REC</th><th>_LogP_REC_CON</th><th>GO</th><th>Category</th><th>Description</th><th>Enrichment</th><th>#GeneInGOAndHitList</th><th>Hits</th></tr>
<tr><td>-10.30352862</td><td>-10.22250406</td><td>R-HSA-2022090</td><td>Reactome Gene Sets</td><td>Assembly of collagen fibrils and other multimeric structures</td><td>29.3313933</td><td>11</td><td>COL1A1|COL1A2|COL3A1|COL5A1|COL6A2|COL6A3|COL12A1|COL15A1|CTSV|ITGB4|COL18A1</td></tr>
<tr><td>-8.315061512</td><td>-11.36069277</td><td>R-HSA-8948216</td><td>Reactome Gene Sets</td><td>Collagen chain trimerization</td><td>49.08766234</td><td>8</td><td>COL1A1|COL1A2|COL3A1|COL5A1|COL6A2|COL6A3|COL12A1|COL15A1</td></tr>
<tr><td>-8.730155106</td><td>-8.820099692</td><td>R-HSA-1474290</td><td>Reactome Gene Sets</td><td>Collagen formation</td><td>19.77397301</td><td>11</td><td>COL1A1|COL1A2|COL3A1|COL5A1|COL6A2|COL6A3|COL12A1|COL15A1|CTSV|ITGB4|COL18A1</td></tr>
<tr><td>-7.145984175</td><td>-9.989800231</td><td>R-HSA-1442490</td><td>Reactome Gene Sets</td><td>Collagen degradation</td><td>33.74776786</td><td>8</td><td>COL1A1|COL1A2|COL3A1|COL5A1|COL6A2|COL6A3|COL12A1|COL15A1</td></tr>
<tr><td>-7.005989899</td><td>-9.825411262</td><td>R-HSA-1650814</td><td>Reactome Gene Sets</td><td>Collagen biosynthesis and modifying enzymes</td><td>32.23667377</td><td>8</td><td>COL1A1|COL1A2|COL3A1|COL5A1|COL6A2|COL6A3|COL12A1|COL15A1</td></tr>
<tr><td>-5.665416663</td><td>-8.24500138</td><td>hsa04974</td><td>KEGG Pathway</td><td>Protein digestion and absorption</td><td>20.57006803</td><td>8</td><td>COL1A1|COL1A2|COL3A1|COL5A1|COL6A2|COL6A3|COL12A1|COL15A1</td></tr>
<tr><td>-5.937575521</td><td>-7.310414834</td><td>R-HSA-1474228</td><td>Reactome Gene Sets</td><td>Degradation of the extracellular matrix</td><td>11.59343609</td><td>10</td><td>COL1A1|COL1A2|COL3A1|COL5A1|COL6A2|COL6A3|COL12A1|COL15A1|CTSV|COL18A1</td></tr>
<tr><td>-5.102200914</td><td>-6.139715633</td><td>R-HSA-216083</td><td>Reactome Gene Sets</td><td>Integrin cell surface interactions</td><td>15.23708743</td><td>8</td><td>CD47|COL1A1|COL1A2|COL3A1|COL5A1|COL6A2|COL6A3|COL18A1</td></tr>
<tr><td>-5.713116523</td><td>-5.524268065</td><td>R-HSA-1474244</td><td>Reactome Gene Sets</td><td>Extracellular matrix organization</td><td>6.540447905</td><td>13</td><td>CD47|COL1A1|COL1A2|COL3A1|COL5A1|COL6A2|COL6A3|COL12A1|COL15A1|CTSV|ITGB4|MFAP5|COL18A1</td></tr>
<tr><td>-3.053927994</td><td>-6.398638667</td><td>R-HSA-3000178</td><td>Reactome Gene Sets</td><td>ECM proteoglycans</td><td>21.3143797</td><td>6</td><td>COL1A1|COL1A2|COL3A1|COL5A1|COL6A2|COL6A3</td></tr>
<tr><td>-4.825458894</td><td>-5.533045522</td><td>R-HSA-3000170</td><td>Reactome Gene Sets</td><td>Syndecan interactions</td><td>29.62767</td><td>5</td><td>COL1A1|COL1A2|COL3A1|COL5A1|ITGB4</td></tr>
<tr><td>-3.73920167</td><td>-6.083344153</td><td>R-HSA-1566977</td><td>Reactome Gene Sets</td><td>Fibronectin matrix formation</td><td>53.99642857</td><td>4</td><td>COL1A1|COL1A2|COL3A1|COL5A1</td></tr>
<tr><td>-3.820334111</td><td>-6.017349586</td><td>R-HSA-9925563</td><td>Reactome Gene Sets</td><td>Developmental Lineage of Pancreatic Ductal Cells</td><td>28.12313988</td><td>5</td><td>COL1A1|COL1A2|COL3A1|COL5A1|TFPI2</td></tr>
<tr><td>-3.205403163</td><td>-5.343213105</td><td>R-HSA-8874081</td><td>Reactome Gene Sets</td><td>MET activates PTK2 signaling</td><td>35.99761905</td><td>4</td><td>COL1A1|COL1A2|COL3A1|COL5A1</td></tr>
<tr><td>-2.891716201</td><td>-4.807382493</td><td>R-HSA-9820448</td><td>Reactome Gene Sets</td><td>Developmental Cell Lineages of the Exocrine Pancreas</td><td>16.07036565</td><td>5</td><td>COL1A1|COL1A2|COL3A1|COL5A1|TFPI2</td></tr>
<tr><td>-2.805723668</td><td>-4.789194045</td><td>R-HSA-8875878</td><td>Reactome Gene Sets</td><td>MET promotes cell motility</td><td>26.33972125</td><td>4</td><td>COL1A1|COL1A2|COL3A1|COL5A1</td></tr>
<tr><td>-3.463364794</td><td>-4.582501593</td><td>hsa04820</td><td>KEGG Pathway</td><td>Cytoskeleton in muscle cells</td><td>6.206486043</td><td>9</td><td>COL1A1|COL1A2|COL3A1|COL5A1|COL6A2|COL6A3|ITGB4|TPM1|TPM2</td></tr>
<tr><td>-2.77531362</td><td>-4.746966911</td><td>R-HSA-419037</td><td>Reactome Gene Sets</td><td>NCAM1 interactions</td><td>25.71258503</td><td>4</td><td>COL3A1|COL5A1|COL6A2|COL6A3</td></tr>
<tr><td>-2.798855881</td><td>-3.466310996</td><td>hsa04512</td><td>KEGG Pathway</td><td>ECM-receptor interaction</td><td>10.78580346</td><td>6</td><td>CD47|COL1A1|COL1A2|COL6A2|COL6A3|ITGB4</td></tr>
<tr><td>-3.902258917</td><td>-3.792140238</td><td>R-HSA-9734767</td><td>Reactome Gene Sets</td><td>Developmental Cell Lineages</td><td>8.174641795</td><td>7</td><td>COL1A1|COL1A2|COL3A1|COL5A1|ERBB2|ITGB4|TFPI2</td></tr>
<tr><td>0</td><td>-4.338150259</td><td>R-HSA-3000480</td><td>Reactome Gene Sets</td><td>Scavenging by Class A Receptors</td><td>42.6287594</td><td>3</td><td>COL1A1|COL1A2|COL3A1</td></tr>
<tr><td>-2.374246144</td><td>-4.188071858</td><td>R-HSA-186797</td><td>Reactome Gene Sets</td><td>Signaling by PDGF</td><td>18.61945813</td><td>4</td><td>COL3A1|COL5A1|COL6A2|COL6A3</td></tr>
<tr><td>-2.273503285</td><td>-4.046893547</td><td>R-HSA-375165</td><td>Reactome Gene Sets</td><td>NCAM signaling for neurite out-growth</td><td>17.14172336</td><td>4</td><td>COL3A1|COL5A1|COL6A2|COL6A3</td></tr>
<tr><td>-2.970565128</td><td>-3.643798704</td><td>R-HSA-3000171</td><td>Reactome Gene Sets</td><td>Non-integrin membrane-ECM interactions</td><td>9.999338624</td><td>5</td><td>COL1A1|COL1A2|COL3A1|COL5A1|ITGB4</td></tr>
<tr><td>-2.002522965</td><td>-3.664841121</td><td>R-HSA-6806834</td><td>Reactome Gene Sets</td><td>Signaling by MET</td><td>13.66998192</td><td>4</td><td>COL1A1|COL1A2|COL3A1|COL5A1</td></tr>
<tr><td>0</td><td>-3.291462416</td><td>R-HSA-2173782</td><td>Reactome Gene Sets</td><td>Binding and Uptake of Ligands by Scavenger Receptors</td><td>19.28443878</td><td>3</td><td>COL1A1|COL1A2|COL3A1</td></tr>
<tr><td>0</td><td>-3.257803477</td><td>hsa04933</td><td>KEGG Pathway</td><td>AGE-RAGE signaling pathway in diabetic complications</td><td>10.69236209</td><td>4</td><td>COL1A1|COL1A2|COL3A1|CCL2</td></tr>
<tr><td>0</td><td>-2.156834643</td><td>hsa04510</td><td>KEGG Pathway</td><td>Focal adhesion</td><td>4.728751271</td><td>6</td><td>COL1A1|COL1A2|COL6A2|COL6A3|ERBB2|ITGB4</td></tr>
<tr><td>0</td><td>-2.429794581</td><td>R-HSA-9006934</td><td>Reactome Gene Sets</td><td>Signaling by Receptor Tyrosine Kinases</td><td>3.007319887</td><td>10</td><td>COL1A1|COL1A2|COL3A1|COL5A1|COL6A2|COL6A3|ERBB2|RRAD|WWOX|PRR5</td></tr>
<tr><td>0</td><td>-2.092294809</td><td>hsa05165</td><td>KEGG Pathway</td><td>Human papillomavirus infection</td><td>3.363140919</td><td>7</td><td>FAS|COL1A1|COL1A2|COL6A2|COL6A3|ITGB4|MAML2</td></tr>
<tr><td>0</td><td>-2.174734886</td><td>hsa05146</td><td>KEGG Pathway</td><td>Amoebiasis</td><td>7.863557559</td><td>3</td><td>COL1A1|COL1A2|COL3A1</td></tr>
<tr><td>-6.34185705</td><td>-7.232857771</td><td>R-HSA-69205</td><td>Reactome Gene Sets</td><td>G1/S-Specific Transcription</td><td>34.28344671</td><td>6</td><td>CDK1|RRM2|TK1|TYMS|CDC45|LIN54</td></tr>
<tr><td>-4.200447921</td><td>-5.212247233</td><td>R-HSA-69206</td><td>Reactome Gene Sets</td><td>G1/S Transition</td><td>13.38754427</td><td>6</td><td>CDK1|CKS1B|RRM2|TK1|CDC45|LIN54</td></tr>
<tr><td>-3.867774573</td><td>-4.867117207</td><td>R-HSA-453279</td><td>Reactome Gene Sets</td><td>Mitotic G1 phase and G1/S transition</td><td>11.65390545</td><td>6</td><td>CDK1|CKS1B|RRM2|TK1|CDC45|LIN54</td></tr>
<tr><td>0</td><td>-2.399041632</td><td>R-HSA-1640170</td><td>Reactome Gene Sets</td><td>Cell Cycle</td><td>3.157685881</td><td>8</td><td>CDK1|CKS1B|RRM2|TK1|CDC45|PHF20|CEP290|LIN54</td></tr>
<tr><td>0</td><td>-2.334558479</td><td>R-HSA-69278</td><td>Reactome Gene Sets</td><td>Cell Cycle, Mitotic</td><td>3.411326715</td><td>7</td><td>CDK1|CKS1B|RRM2|TK1|CDC45|CEP290|LIN54</td></tr>
<tr><td>-2.064088629</td><td>0</td><td>R-HSA-69656</td><td>Reactome Gene Sets</td><td>Cyclin A:Cdk2-associated events at S phase entry</td><td>7.156923077</td><td>3</td><td>CDC25B|CKS1B|LIN54</td></tr>
<tr><td>-2.87265771</td><td>-2.406859488</td><td>hsa01232</td><td>KEGG Pathway</td><td>Nucleotide metabolism</td><td>11.29337068</td><td>6</td><td>IMPDH1|RRM2|TK1|TK2|TYMS|NUDT16</td></tr>
<tr><td>-2.645605798</td><td>-2.234437129</td><td>R-HSA-15869</td><td>Reactome Gene Sets</td><td>Metabolism of nucleotides</td><td>9.795270489</td><td>6</td><td>IMPDH1|RRM2|TK1|TK2|TYMS|NUDT16</td></tr>
<tr><td>-2.374246144</td><td>0</td><td>hsa00240</td><td>KEGG Pathway</td><td>Pyrimidine metabolism</td><td>11.03375296</td><td>4</td><td>RRM2|TK1|TK2|TYMS</td></tr>
<tr><td>0</td><td>-2.465790032</td><td>hsa00983</td><td>KEGG Pathway</td><td>Drug metabolism - other enzymes</td><td>7.900711999</td><td>4</td><td>IMPDH1|RRM2|TK1|TK2</td></tr>
<tr><td>-3.006913628</td><td>-3.527308902</td><td>R-HSA-6804757</td><td>Reactome Gene Sets</td><td>Regulation of TP53 Degradation</td><td>23.14132653</td><td>3</td><td>CDK1|PHF20|PRR5</td></tr>
<tr><td>-2.970902495</td><td>-3.490690295</td><td>R-HSA-6806003</td><td>Reactome Gene Sets</td><td>Regulation of TP53 Expression and Degradation</td><td>22.4985119</td><td>3</td><td>CDK1|PHF20|PRR5</td></tr>
<tr><td>-3.481010271</td><td>-3.178775392</td><td>R-HSA-8878166</td><td>Reactome Gene Sets</td><td>Transcriptional regulation by RUNX2</td><td>8.439767779</td><td>5</td><td>RUNX2|RUNX1|CDK1|COL1A1|HDAC4</td></tr>
<tr><td>-3.12200654</td><td>0</td><td>R-HSA-8941326</td><td>Reactome Gene Sets</td><td>RUNX2 regulates bone development</td><td>16.77403846</td><td>3</td><td>RUNX2|COL1A1|HDAC4</td></tr>
<tr><td>0</td><td>-2.392599559</td><td>R-HSA-8878159</td><td>Reactome Gene Sets</td><td>Transcriptional regulation by RUNX3</td><td>9.417981728</td><td>3</td><td>RUNX1|HDAC4|MAML2</td></tr>
<tr><td>-3.250486496</td><td>-2.003365558</td><td>hsa04668</td><td>KEGG Pathway</td><td>TNF signaling pathway</td><td>7.517776341</td><td>5</td><td>FAS|CCL2|TRAF2|TRAF5|RPS6KA4</td></tr>
<tr><td>-2.694861785</td><td>-2.271896917</td><td>hsa04657</td><td>KEGG Pathway</td><td>IL-17 signaling pathway</td><td>7.533603239</td><td>4</td><td>CCL2|TRAF2|TRAF5|IL17RA</td></tr>
<tr><td>-2.436910602</td><td>0</td><td>hsa05168</td><td>KEGG Pathway</td><td>Herpes simplex virus 1 infection</td><td>4.915469146</td><td>5</td><td>FAS|CCL2|SRSF5|TRAF2|TRAF5</td></tr>
<tr><td>-2.064088629</td><td>-2.560404985</td><td>hsa04115</td><td>KEGG Pathway</td><td>p53 signaling pathway</td><td>8.532768959</td><td>4</td><td>FAS|CDK1|RRM2|RCHY1</td></tr>
<tr><td>-2.688594212</td><td>0</td><td>hsa02010</td><td>KEGG Pathway</td><td>ABC transporters</td><td>11.92820513</td><td>3</td><td>ABCA1|ABCA2|ABCB8</td></tr>
<tr><td>-2.043806765</td><td>0</td><td>R-HSA-3781865</td><td>Reactome Gene Sets</td><td>Diseases of glycosylation</td><td>5.516876482</td><td>5</td><td>B4GAT1|MAN1B1|ALG12|GALNT12|DHDDS</td></tr>
<tr><td>0</td><td>-2.246781513</td><td>R-HSA-5620912</td><td>Reactome Gene Sets</td><td>Anchoring of the basal body to the plasma membrane</td><td>8.349963181</td><td>3</td><td>CDK1|TCTN1|CEP290</td></tr>
<tr><td>0</td><td>-2.140435278</td><td>R-HSA-6785807</td><td>Reactome Gene Sets</td><td>Interleukin-4 and Interleukin-13 signaling</td><td>7.641004043</td><td>3</td><td>COL1A2|HMOX1|CCL2</td></tr>
<tr><td>-2.112737314</td><td>0</td><td>R-HSA-199992</td><td>Reactome Gene Sets</td><td>trans-Golgi Network Vesicle Budding</td><td>7.455128205</td><td>3</td><td>AP4M1|BLOC1S4|DTNBP1</td></tr>
</table>
