# Supplementary material for: Development and characterization of Hanwoo oviduct epithelial cell line for heat stress study
Source: Anim Biosci. 2026 Sep 1;39(11):250962. doi: 10.5713/ab.250962 (PMC13740644; doi:10.5713/ab.250962)
Supplement: Supplementary file 10 [file ab-250962-Supplementary-2.pdf]

Supplement 2. List of primers used in this study.

qRT-PCR primers for cattle

Gene symbol	Description	Primer sequence (5'-3')	Tm (°C)	Amplicon length (bp)
GADPH	Glyceraldehyde-3-phosphate dehydrogenase	F: GCCGATGCCCCCATGT<br>R: CAGGAGGCATTGCTGACAATC	60	80
SOD1	Superoxide dismutase 1	F: CACGATGGTGGTCCATGAAA<br>R: TTCCAGCGTTGCCAGTCTTT	60	80
CAT	Catalase	F: TCCAAGGCGAAGGTGTTTG<br>R: CCCGATTCTCCAGCAACAGT	60	80
GPX1	Glutathione Peroxidase 1	F: CCCCTGCAACCAGTTTGG<br>R: CGCCTGGTCGGACGTACTT	60	80
BAG3	BCL2-associated athanogene 3	F: ACCAGGAGAAGCTGAAGTGC<br>R: TCCAGCCCTTGCACTTTCTC	60	80
HSPB1	Heat shock protein family B member 1	F: CCCTGGACGTCAACCACTTC<br>R: GTGCTTGCCAGTGATCTCCA	60	80
FOXO3	Forkhead box O3	F: CTCCAGGACCTGCTCACATC<br>R: GCCTGAGACATCAAGGGGTC	60	80
CHAC1	ChaC glutathione-specific gamma-glutamylcyclotransferase 1	F: CACAGATCTTGGCCTGCAGA<br>R: ACAGAGCTGCATGAAGTCCG	60	80

SV40T vector primers

Target location	Primer sequence (5'-3')	Tm (°C)	Amplicon length (bp)
Coding region of SV40-T gene	F: ATGGGAGCAGTGGTGGAATG<br>R: GCAGACACTCTATGCCTGTGT	59<br>59.1	351
