# Supplementary material for: Development and characterization of Hanwoo oviduct epithelial cell line for heat stress study
Source: Anim Biosci. 2026 Sep 1;39(11):250962. doi: 10.5713/ab.250962 (PMC13740644; doi:10.5713/ab.250962)

Supplement 3. Gel electrophoresis of RT-PCR of the oxidative stress marker genes.

Agarose gel (1.5%) electrophoresis (120V, 25 min) of RT-PCR of the oxidative stress marker genes i.e. *GAPDH*, *SOD1*, *CAT*, and *GPX1* were conducted to confirm the specificity of qRT-PCR primers. All samples revealed single bands of targeted specific amplicons size. HS- = heat stress, R- = recovery, -H = hours.

M = marker
1 = Control (0H)
2 = HS4H
3 = R4H
4 = HS12H
5 = R12H
6 = HS24H
7 = R24H

Gene	Target length (bp)
GAPDH	80
SOD1	80
CAT	80
GPX1	80

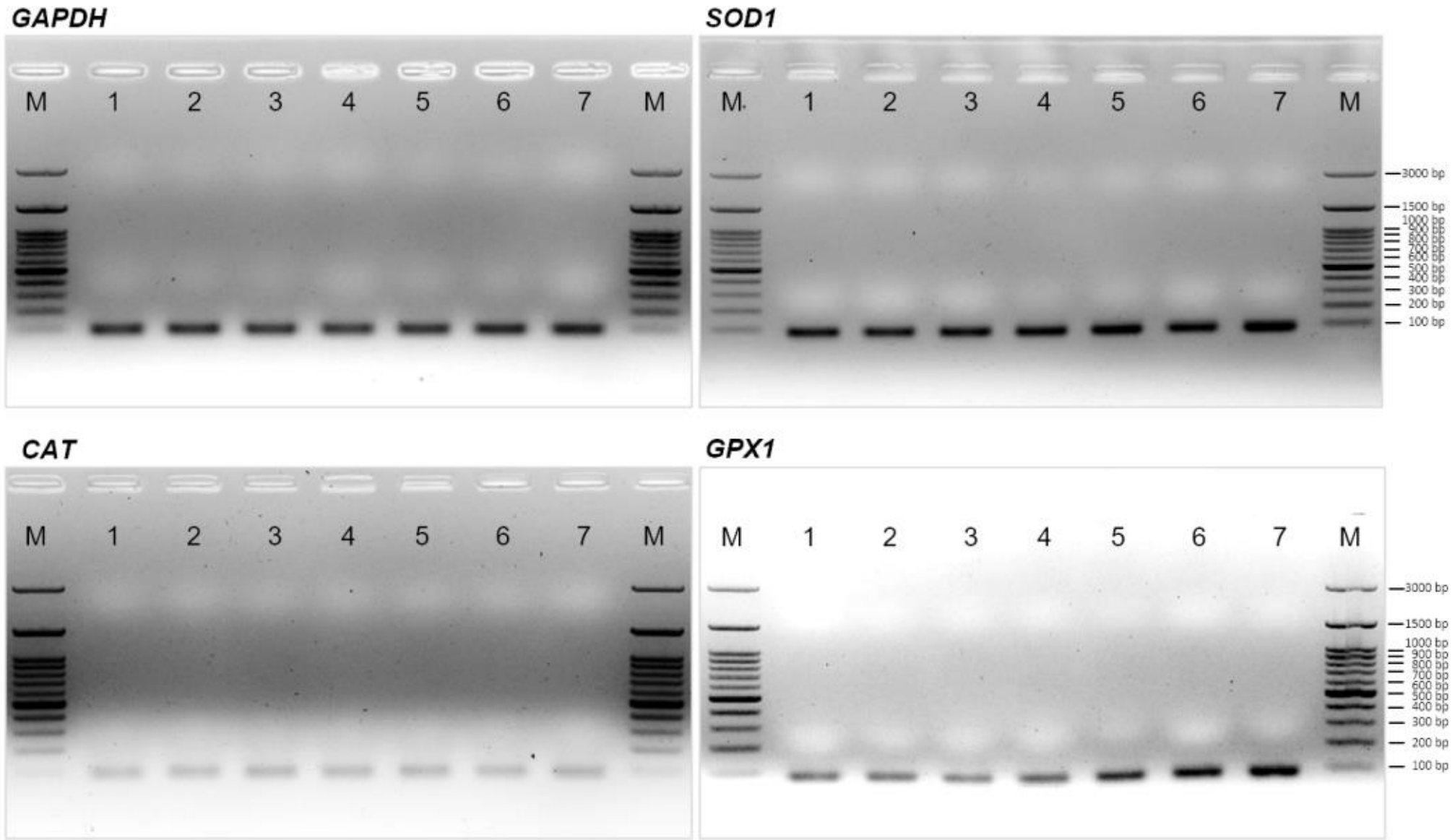

Supplement: Supplementary file 11 [file ab-250962-Supplementary-3.pdf]
