# Supplementary material for: Development and characterization of Hanwoo oviduct epithelial cell line for heat stress study
Source: Anim Biosci. 2026 Sep 1;39(11):250962. doi: 10.5713/ab.250962 (PMC13740644; doi:10.5713/ab.250962)
Supplement: Supplementary file 12 [file ab-250962-Supplementary-4.pdf]

Supplement 4. Microarray normalized fold-change of immortalized oviduct epithelial cells under heat stress.

ProbeID	Gene_Symbol	Gene_ID	mRNA Accession	OV24H/OV0H.fc	OV24R/OV0H.fc	OV24H/OV24R.fc
12730813	CRYAB	281719	NM_174290	3.662732	1.372047	2.669538
12759325	LOC616720	616720	XM_001788477	-1.116358	1.613764	-1.801539
12867245	SMS	615950	NM_001035471	1.037262	1.141009	-1.100020
12889007	CLU	280750	NM_173902	4.096523	3.422855	1.196815
12804546	HSPA1A	281825 // 2822!	NM_174550	-1.361324	-1.718214	1.262165
12726925	LYVE1	404179	NM_205815	1.072056	-1.065573	1.142354
12683494	SLC5A3	282362	NM_174607	1.781620	-1.039378	1.851777
12856372	CLEC2B	100297496	XM_010805551	1.289123	1.014557	1.270626
12840607	LRIF1	539067	XM_003581959	1.389803	1.138181	1.221073
12790461	SERF2	527852	NM_001206348	1.299395	1.539061	-1.184444
12759819	LOC618289	618289	XM_010800228	1.802301	4.737842	-2.628774
12805397	ENPP5	512304	NM_001206724	1.718405	5.078914	-2.955598
12812811	HSPB1	516099	NM_001025569	1.925136	1.231153	1.563685
12726906	TRIM21	359715	NM_182655	1.182516	1.097013	1.077942
12802720	FLOT1	532573	NM_001076887	2.852842	2.210879	1.290366
12717715	LOC100138864	100138864	XR_810477	1.438914	-1.163854	1.674685
12712971	MTUS2	616677	XM_005194136	-1.973984	-1.482375	-1.331636
12703510				1.527011	-1.123796	1.716048
12778599	LOC540098	540098	XR_805091	1.701256	1.305896	1.302750
12751423	BANP	513446	NM_001075620	2.542310	-1.006676	2.559284
12759673	ZNF585A	767967	XM_002694957	1.495326	1.332689	1.122037
12868064	GDF11	540517	XM_002687421	1.227472	-1.042068	1.279110
12781386	SCRN3	511983	NM_001075547	1.632202	-1.055634	1.723009
12708565	GEN1	785690	NM_001205450	2.588668	1.365245	1.896120
12733529	C15H11orf71	615529	NM_001078051	1.530847	1.576669	-1.029933
12903563	LOC508820	508820	NM_001075373	-1.232801	-1.649753	1.338215
12886230	TNFAIP8	529336	NM_001083711	2.113318	2.065141	1.023329
12678907	ST3GAL6	444860	NM_001002883	-1.431602	-2.509222	1.752738
12784117	CLK1	613808	NM_001102271	1.955829	1.481707	1.319984
12847098	CCDC18	540863	XM_005193418	3.054275	3.443343	-1.127385
12785694	RAD1	518502	NM_001192490	2.857414	1.411677	2.024127
12777737	SCN9A	533065	NM_001110787	4.761975	1.267416	3.757231
12839892	ODF2L	533508	NM_001075993	2.569273	1.919832	1.338280
12750919	ZNF529	510501	XM_002694950	1.731629	1.272636	1.360663
12821078	BAG3	782633	NM_001082471	1.254907	1.066970	1.176140
12682890	LOC100139345	100139345	XM_002685011	1.751122	1.403433	1.247742
12805505	SNRNP48	513601	XM_002697597	1.472339	-1.110462	1.634977
12868450	RASSF9	615588	NM_001193083	-2.021828	-1.340118	-1.508694
12862891	THAP2	781850	XM_003582168	2.485593	1.078907	2.303807
12728877	DEPDC7	532586	NM_001192730	1.859545	1.387781	1.339941
12784836	RANBP3L	100296398	XM_002696376	1.000735	-1.055312	1.056088
12892282	MIRLET7F-1	791061	NR_030957	2.149886	2.033253	1.057362
12873712	COMMD8	507292	XM_002688237	1.723224	1.312111	1.313321
12906573	MIR105B	100312990	NR_031127	1.377431	1.180543	1.166778
12718996	NSUN6	511775	NM_001083670	1.228195	1.077793	1.139547
12779266	RSRP1	615263	NM_001103298	2.236434	-1.122153	2.509622
12779974	MIR2351	100313159	NR_031077	-3.039681	-1.423863	-2.134813
12808650	ADCYAP1	615187	NM_001046555	-2.185616	-1.977531	-1.105225
12902018			XM_002683967	1.585766	1.390323	1.140574
12778639	CYTIP	540450	NM_001102243	-1.654849	-1.186326	-1.394937
12786691	LOC783613	783613	XM_010816713	3.110874	-1.056022	3.285152
12910548	LOC784704	784704	XR_816901	1.181721	-1.314314	1.553153
12900274	FUCA2	515729	NM_001205818	1.125995	1.288230	-1.144082
12886251	CLK4	529633	NM_001192988	2.504218	2.047268	1.223200
12851611	TBX20	539419	NM_001192237	1.310839	1.540000	-1.174820
12726617	MDK	280852	NM_173935	1.211286	1.235821	-1.020255

ProbeID	Gene_Symbol	Gene_ID	mRNA Accession	OV24H/OV0H.fc	OV24R/OV0H.fc	OV24H/OV24R.fc
12855975	BET1	616526	NM_001099157	1.644980	1.503870	1.093831
12879520	PCDHGA8	521340	NM_001099705	-1.506959	-1.208427	-1.247042
12791662	HSP90AA1	281832	NM_001012670	-1.044650	-1.190436	1.139555
12901028	SLC35A1	536838	NM_001034637	1.719322	1.112534	1.545410
12772627	TMEM100	613987	NM_001046522	1.036622	-1.100638	1.140946
12807097	ATAT1	786491	NM_001276323	1.470615	1.106643	1.328898
12713136	USPL1	788351	XM_001255437	1.824056	1.738352	1.049302
12755419	ZNF729	768043	XM_005219628	1.134526	1.247621	-1.099685
12892464	MIR2469	100313237	NR_031008	1.018030	1.335611	-1.311957
12825573	UFSP2	617788	NM_001076477	1.254533	1.012969	1.238471
12720706	ZNFX1	539807	NM_001205716	1.200329	1.422916	-1.185438
12906051	ERCC6L	782916	NM_001102530	-1.132044	-1.036694	-1.091975
12855928	RGS2	100848920 // 5:	NM_001075596	1.387906	-1.068028	1.482323
12697010	LOC613512	613512	XR_809342	-1.939104	-1.431969	-1.354152
12710969	MIR15A	100170925	NR_030793	1.247448	-1.206100	1.504548
12866943	FOXRED2	532871	NM_001206011	**2.351971**	1.830452	1.284912
12856114	LOC511712	511712	XR_803175	1.022854	1.380040	-1.349205
12812729	EPHB4	515756	NM_001206268	1.018976	1.109293	-1.088635
12753652	MED29	614626	NM_001080316	-1.086012	1.459951	-1.585524
12908846	GABRE	524115	XM_002699672	1.361871	1.782349	-1.308750
12753128	CNTNAP4	536775	NM_001206511	-1.446885	-1.583218	1.094225
12891820	C8H9orf85	614778	NM_001101235	**4.701594**	**3.072834**	1.530051
12712702	RFXAP	540760	XM_002691750	1.929919	1.489388	1.295779
12881344	TUBB4A	540236	NM_001034697	-1.071016	-1.666833	1.556311
12692579			ENSBTAT00000034439	1.778536	1.391162	1.278454
12753154	ZNF175	537103	XM_001790619	1.261299	1.414449	-1.121423
12772102	ZFP3	538648	NM_001099089	**2.259964**	1.554639	1.453690
12747823	HSPB8	539524	NM_001014955	1.709281	1.930936	-1.129677
12803821	ZSCAN12	768084	NM_001077138	1.308388	-1.039868	1.360550
12829760	CHORDC1	505144	NM_001045912	1.476366	**-2.721440**	**4.017840**
12899712	CASP8AP2	505138	XM_010798926	1.223692	1.081220	1.131769
12754336	ZNF566	767945	NM_001077017	**2.384819**	1.700183	1.402684
12702031	SUPT7L	539152	NM_001076150	1.558524	1.312839	1.187140
12805757	NKAPL	515716	NM_001102498	-1.050612	1.175782	-1.235290
12725482	GGH	525303	NM_001105394	**2.122611**	1.628722	1.303237
12839817	MCOLN2	532671	NM_001192734	1.298081	1.083779	1.197736
12708822	MIR2303	100313126	NR_031116	1.978435	**2.539211**	-1.283444
12816236	ZNF500	510490	NM_001105353	1.999155	1.988361	1.005428
12786853			ENSBTAT00000009630	1.362192	1.032477	1.319343
12732358	MPZL3	521837	NM_001098051	1.853292	1.338753	1.384342
12847867	RNPC3	768060	NM_001077117	1.572827	1.385292	1.135376
12910712	SCML1	786670	XM_001254287	1.345498	1.402198	-1.042141
12753873	ZNF576	616814	NM_001076433	1.155791	-1.048364	1.211689
12895395	LOC616883	616883	XM_002689834	1.283177	1.069006	1.200346
12878480	ZNF354B	510078	XM_005193344	1.328603	1.132476	1.173185
12808949	LOC100295873	100295873	XR_815221	-1.466086	-1.172762	-1.250114
12905870	KANTR	617166	NR_132122	**2.422052**	**2.004302**	1.208427
12790978	PAX9	540196	NM_001192369	-1.005456	1.391808	-1.399402
12871206	PARM1	518368	NM_001075771	1.788611	-1.710360	**3.059169**
12815040	PGP	538173	NM_001038172	**2.163924**	**2.163924**	1.000000
12845158	BTBD8	520456	NM_001105200	1.227353	1.527858	-1.244840
12810708	C24H18orf32	767921	NM_001195062	1.762887	1.469097	1.199980
12886623	LOC100851455	100851455	XM_005193529	1.543986	-1.097066	1.693855
12777779	DNAJB2	533668	NM_001034592	1.723152	1.351386	1.275100
12729666	SLC35C1	540413	NM_001101210	-1.063861	1.018044	-1.083058
12732550	LOC528515	528515	XM_002693239	1.048379	-1.126705	1.181214
12816222	AGFG2	510361	NM_001206722	1.149686	1.476427	-1.284200

ProbeID	Gene_Symbol	Gene_ID	mRNA Accession	OV24H/OV0H.fc	OV24R/OV0H.fc	OV24H/OV24R.fc
12737711	LOC784034	784034	XM_001252463	1.319837	-1.104819	1.458181
12754347	HSBP1	767979	NM_001113316	-1.014191	-1.024557	1.010220
12730651				-1.150595	-1.371087	1.191633
12697892	LOC790312	790312	XM_002690745	1.776170	1.234700	1.438545
12858232	KMT2D	506805	XM_010805028	1.522149	1.356303	1.122278
12737308	MARC2	615506	NM_001076380	1.225168	1.373189	-1.120816
12841195			ENSBTAT00000033541	-1.316968	1.143693	-1.506207
12735978	TNFRSF9	520341	NM_001035336	-2.137968	-4.879252	2.282192
12851621	FAM71F1	539444	NM_001046457	1.716905	-1.081182	1.856287
12825157	SARAF	515461	NM_001076856	1.051333	1.103197	-1.049331
12905810	FANCB	616549	XM_010800601	-1.226434	-1.322548	1.078368
12904277			ENSBTAT00000020218	1.466615	1.346309	1.089359
12680464	DTX3L	515051	NM_001192396	1.542382	2.098720	-1.360701
12795924	CAMK1	520498	NM_001076868	1.297703	1.096283	1.183730
12808216	ANKRD12	534739	XM_002697743	1.205682	1.307989	-1.084853
12746863	LOC101906230	101906230	XR_812325	1.605553	1.596265	1.005819
12740874	TRMT1L	540872	NM_001098121	1.953471	1.240619	1.574594
12851673	UBN2	540792	NM_001193208	1.577675	1.385109	1.139025
12741933	PPID	281420	NM_174153	1.848455	1.223513	1.510776
12772695	CORO6	614661	NM_001102282	1.201578	-1.022166	1.228212
12851464	C4H7orf60	538658	XM_002686799	1.175724	1.395691	-1.187091
12707526	ZBTB26	100848248	XM_003582824	-1.024450	1.281266	-1.312593
12736714	TMEM183A	534797	NM_001034616	1.614570	-1.226035	1.979519
12767392	CALCOCO2	281942	NM_174400	1.547619	1.331996	1.161879
12813124	ZNF394	522836	NM_001077955	1.156239	-1.116026	1.290393
12773248	HOXB4	768240	NM_001078114	1.115082	1.083921	1.028748
12815778	KIAA0430	506615	NM_001271994	1.656318	1.027914	1.611339
12691680	ICE2	541255	NM_001076265	2.469228	1.621715	1.522603
12841430	PPAP2B	617707	NM_001076473	1.145407	1.187864	-1.037068
12831467	PRCP	534927	NM_001038164	1.398267	-1.054523	1.474504
12805356	ZKSCAN8	511794	NM_001100344	1.185364	1.371439	-1.156977
12691075	SV2C	538430	NM_001192019	-1.339449	-1.533087	1.144565
12895239	UHRF2	613759	NM_001102270	1.617438	-1.016577	1.644250
12877262	MFAP3	317721	NM_001166519	1.182426	1.355119	-1.146050
12785577	CREBRF	513587	XM_002696253	2.103439	2.418093	-1.149590
12826652	DDX50	534331	NM_001206053	1.773058	1.464857	1.210397
12699808	RPL7A	513128	NM_001040520	1.039781	1.444710	-1.389437
12750962	TXNL4B	510719	NM_001014897	1.477912	1.216916	1.214473
12781532	XKR8	514365	XM_002685687	-1.092505	-1.025544	-1.065293
12810622	TIMM21	614633	NM_001035453	-1.053544	-1.108862	1.052507
12849348	TMEM176A	404082	NM_205779	1.087850	-1.110093	1.207615
12855706	LSMEM1	613798	NM_001099718	-1.041931	-1.077733	1.034361
12798858	TATDN2	510836	XM_002696881	1.462929	1.609999	-1.100531
12865041	FKBP4	508535	NM_001034322	-1.060467	-1.462655	1.379256
12697644				1.150467	1.150467	1.000000
12843761	ADAMTSL4	507654	NM_001101061	1.355711	1.356012	-1.000222
12857312	CBY1	282859	NM_174739	2.107175	1.901358	1.108247
12836937	OMA1	506223	NM_001035033	1.278303	1.191129	1.073186
12842922			ENSBTAT00000043099	1.772800	1.765969	1.003868
12846170	RABGGTB	533276	NM_001015646	1.318932	1.334575	-1.011860
12738517	PLOD1	281409	BT025353	2.019265	1.447497	1.395005
12753239	NFAT5	538523	XM_002694839	1.341670	1.631546	-1.216056
12747503	SPPL3	535156	XM_002694525	1.340889	1.479962	-1.103717
12887373	ARSI	540390	XM_002689292	-1.306684	-1.187313	-1.100539
12865903	SLC6A12	514339	NM_001101994	-1.101218	-1.324722	1.202961
12881643	MRPL22	614639	NM_001035454	1.267029	1.150491	1.101294
12880946	ARHGAP26	538219	NM_001205522	1.093452	1.357997	-1.241935

ProbeID	Gene_Symbol	Gene_ID	mRNA Accession	OV24H/OV0H.fc	OV24R/OV0H.fc	OV24H/OV24R.fc
12894545	RNF38	532877	NM_001034581	-1.805765	-1.150021	-1.570202
12754730	LOC787397	787397	XM_010814918	1.481122	-1.442389	**2.136353**
12761520	ZNHIT3	504662	NM_001045895	1.997146	1.504454	1.327489
12865887	DDX17	514323	NM_001101993	1.181590	1.636666	-1.385138
12820493	SUFU	535067	NM_001098083	1.190692	-1.059945	1.262068
12745816	HSPA4L	506096	XM_002707745	1.524462	**2.523752**	-1.655503
12753012	DEDD2	534251	NM_001076017	1.412274	1.253090	1.127033
12861727	WNT10B	539337	XM_002687290	1.122068	-1.338985	1.502432
12814558	YPEL3	787498	NM_001205409	1.506311	1.946969	-1.292541
12704139	EIF2AK2	347700	NM_178109	1.248305	-1.005686	1.255403
12891839	TMEM38B	615646	NM_001076387	1.356050	1.753977	-1.293446
12706234	DPM2	523737	NM_001046268	1.788624	1.546396	1.156640
12758837	TOX3	539135	XM_002694777	1.108140	1.074846	1.030975
12890878	DNAJA1	528862	NM_001015637	1.199123	-1.111163	1.332421
12776458	EYA3	514364	NM_001206731	1.591790	1.155911	1.377087
12780621	PGAP1	504677	NM_001205550	1.264888	1.372732	-1.085259
12879363	CYFIP2	518833	NM_001205850	1.201478	1.523215	-1.267785
12844109	INTS3	511052	NM_001193087	1.547458	1.188449	1.302082
12754885	PAPD5	789894	XM_010814610	1.333391	1.457918	-1.093391
12793887	MBIP	774027	NM_001077518	1.178531	1.396611	-1.185043
12872978	MIR2450B	100313224	NR_031191	**2.227306**	1.381667	1.612043
12885399	ERAP1	514617	NM_001102003	1.355993	1.384236	-1.020828
12799813	RBM5	534216	NM_001046374	1.575664	1.496000	1.053252
12852248	ARL4A	767906	NM_001076985	1.318658	1.646988	-1.248988
12835058	B4GAT1	618055	NM_001034808	1.780991	1.298981	1.371068
12808661	RTTN	615378	NM_001206207	1.376238	1.120397	1.228349
12838581	HENMT1	515853	XM_002686160	-1.353533	-1.388156	1.025580
12789359	PRPF39	505547	NM_001109789	-1.001783	-1.408969	1.406461
12854094	SAMD9	514205	NM_001205781	**-5.028820**	-1.599787	**-3.143431**
12709831	PIBF1	524913	XM_002691887	1.368675	1.266906	1.080328
12697609	GCNT4	782825	XM_005200869	-1.151305	-1.057773	-1.088423
12869555	EREG	100295476	XM_010797936	-1.562872	-1.604841	1.026853
12808744	PHLPP1	615982	XM_002707786	1.216191	1.155086	1.052901
12838432	MCOLN3	514345	NM_001192367	-1.153046	-1.225236	1.062608
12906463	TMEM257	101901895	XM_005228587	1.052390	-1.118039	1.176613
12754295	ZNF181	767826	NM_001076549	-1.055795	-1.138410	1.078249
12713011	DCUN1D2	618507	XM_010811009	1.309141	1.365908	-1.043362
12724392	TRPA1	505317	XM_010812107	-1.006572	1.028570	-1.035330
12696838	VCPKMT	540184	NM_001110083	1.629264	1.771744	-1.087451
12722156	TATDN1	509365	NM_001075402	1.274852	1.772616	-1.390448
12759624	ADAT1	618521	NM_001081620	1.835674	1.316986	1.393845
12795463	EIF1B	513481	NM_001037598	**2.204055**	**2.077113**	1.061114
12741155	TATDN3	615965	NM_001080320	1.382481	1.375103	1.005365
12760151	ZNF583	789836	NM_001083534	1.331258	1.228732	1.083441
12753402	ZNF432	539365	NM_001192232	**2.007138**	1.401236	1.432406
12774536	HSPE1	281833	NM_174346	1.679079	-1.010528	1.696757
12799774	MLH1	533652	NM_001075994	1.309658	1.314943	-1.004035
12685693	KLHL24	533510	NM_001206196	1.163814	1.721373	-1.479080
12686285	MYNN	539376	NM_001035424	1.878980	1.459324	1.287569
12773273	SMCR8	781661	XM_003583598	1.211967	1.814409	-1.497079
12875155	PAQR3	534876	NM_001206068	1.184411	1.115677	1.061607
12725871	PLAG1	539210	XM_002692655	-1.361597	1.062601	-1.446835
12833395	CTSF	509715	NM_001075416	1.866027	**2.181303**	-1.168955
12748975	LOC100299712	100299712	XM_005219581	**2.039195**	1.069873	1.906016
12895265	GKAP1	613960	NM_001040573	**3.402818**	**3.423377**	-1.006042
12754148	LOC104974934	104974934	XM_010815399	1.439392	1.298864	1.108194
12701712	TRMT61B	535837	XM_001790235	-1.190535	1.792210	**-2.133689**

ProbeID	Gene_Symbol	Gene_ID	mRNA Accession	OV24H/OV0H.fc	OV24R/OV0H.fc	OV24H/OV24R.fc
12729991	RNF214	616238	NM_001206820	1.648656	1.807982	-1.096640
12691345	ZC2HC1C	540480	XM_005193743	2.417825	1.760835	1.373113
12721001	ECHDC3	617368	NM_001193156	1.151297	2.240235	-1.945836
12771254	SLC25A35	527669	NM_001031763	2.013562	2.107277	-1.046542
12682311	LOC614129	614129	XR_083075	2.100336	1.622535	1.294478
12880985	DNAJB1	538426	NM_001033763	-1.060776	-1.211731	1.142307
12691887	TMEM260	614796	NM_001099139	2.405805	1.166333	2.062709
12908896	UXT	525680	NM_001037471	2.104868	1.710929	1.230249
12772826			ENSBTAT00000006464	2.070243	1.479111	1.399654
12818565	FOPNL	782821	NM_001101286	1.888578	2.227430	-1.179422
12681770	ZMAT3	538512	NM_001076108	2.034861	2.870854	-1.410836
12689708	VPS18	518781	NM_001100380	1.759798	2.099273	-1.192906
12854397	ORC5	519409	NM_001099704	2.036173	1.683052	1.209810
12758399	ZNF416	530973	XM_002695492	1.330926	-1.038039	1.381552
12779684	LOC785710	785710	XR_804983	1.839368	2.450473	-1.332236
12791746	NFKBIA	282291	NM_001045868	-2.165590	-2.246579	1.037398
12745062	USP30	100140210	NM_001191361	1.835572	1.908422	-1.039688
12778449	ID3	538690	NM_001014950	2.137597	1.969733	1.085222
12701187	AHSA2	531017	NM_001102134	1.146741	-1.005393	1.152926
12807542	NPC1	286772	NM_174758	2.137716	2.518858	-1.178294
12790914	LINS	539267	NM_001205649	2.081697	1.405497	1.481111
12774686	HMGCL	317658	NM_001075132	1.730777	2.128858	-1.230001
12754725	ZNF471	787287	XM_001254729	1.864902	1.666533	1.119031
12789116	CYP1A1	282870	XM_002696635	1.474423	-1.241935	1.831137
12888026	GADD45B	618405	NM_001040604	1.641643	-1.078137	1.769915
12872976	MIR2450C	100313223	NR_031190	1.243055	-1.237862	1.538730
12805654	CAP2	515190	NM_001098979	1.473380	-1.000201	1.473677
12847465	BTBD19	615438	NM_001101241	4.862135	1.792471	2.712532
12694538	PARP6	511329	NM_001103241	2.159699	1.605775	1.344957
12874660	HSD17B11	527592	NM_001046286	-1.763180	1.148452	-2.024927
12902050			XM_003583467	1.631071	1.765382	-1.082345
12833481	PCF11	510604	XM_010820857	2.086594	1.985194	1.051078
12795124	TRANK1	509859	XM_002696940	-1.396901	1.025381	-1.432356
12896966	TSPYL4	508104	NM_001101064	-1.001894	2.067375	-2.071291
12828142	P4HA1	518288	NM_001075770	1.686790	-2.726084	4.598330
12806585	MYLIP	541070	NM_001102253	2.053351	2.290496	-1.115492
12748602	ZNF570	100138971	NM_001191353	2.722402	2.493825	1.091657
12714001	ID1	497011	NM_001097568	2.382226	1.365188	1.744979
12860539	SLC38A4	527476	NM_001205943	-1.071528	-1.146320	1.069799
12682021	GTF2E1	540525	NM_001103294	2.257084	1.958433	1.152495
12837074	TXNIP	506790	NM_001101875	3.084144	3.828555	-1.241367
12746819	ZNF280B	517697	NM_001077935	2.257256	1.978792	1.140724
12900168	ZBTB2	513775	NM_001205768	2.210419	1.547404	1.428469
12895667	SVEP1	781282	XM_002705057	2.131295	1.975161	1.079049
12829082	H19	100126192	NR_003958	-1.105623	4.618166	-5.105952
12688722	HAUS2	508698	NM_001076819	2.200208	2.191730	1.003868
12844992	LOC516742	516742	XM_002686058	2.098590	1.470483	1.427143
12724433	NOV	505727	NM_001102382	1.773931	3.178685	-1.791887
12699996	CHCHD5	514560	NM_001037601	1.185241	1.163886	1.018348
12738537	SELE	281484	NM_174181	-1.005979	-1.276435	1.268849
12868298	HELB	614572	NM_001206182	-1.120910	-2.260372	2.016551
12684497	HSPBAP1	513211	NM_001014911	1.441899	1.987796	-1.378596
12874999			ENSBTAT00000003877	1.500694	-1.997686	2.997916
12834357	TMEM86A	532018	NM_001035388	1.580674	2.761955	-1.747327
12853877	POT1	511292	XM_002686916	2.214928	1.669690	1.326551
12860511	CCDC77	527084	NM_001102106	-1.296264	-1.424218	1.098710
12776966	SYF2	522372	XM_002685769	1.304322	2.159190	-1.655412

ProbeID	Gene_Symbol	Gene_ID	mRNA Accession	OV24H/OV0H.fc	OV24R/OV0H.fc	OV24H/OV24R.fc
12765241	CTNS	613527	NM_001102264	-1.031161	1.486480	-1.532801
12708934	LOC520016	520016	XM_005199553	-1.977503	**-2.153256**	1.088876
12726404	LOC100296550	100296550	XM_002693189	**-2.151764**	-1.328861	-1.619255
12798341	CRELD1	504854	NM_001014851	**2.029606**	**2.227368**	-1.097438
12715842	TTPAL	526582	NM_001205931	1.241204	1.333890	-1.074675
12851820	THAP5	614615	NM_001076339	1.485357	**2.328386**	-1.567559
12829222	LOC100301320	100301320	XM_002699128	-1.818312	**-2.066043**	1.136242
12707555	ZER1	540351	NM_001205808	1.231605	1.410102	-1.144930
12724001	LOC100299242	100299242	XR_811002	1.045773	1.076650	-1.029526
12906065	LOC104970028	104970028	XM_010799725	1.562612	**2.009797**	-1.286178
12901444	LRP11	617450	NM_001206831	**2.159534**	**2.170564**	-1.005108
12738690	TNFRSF1B	338033	NM_001040490	-1.492127	-1.560610	1.045896
12905449	KLHL15	540246	NM_001205799	**2.353520**	1.663994	1.414380
12749545	BCAM	282862	NM_174741	1.263670	1.548005	-1.225007
12767655	GABARAP	327715	NM_001034048	1.611719	**2.181575**	-1.353570
12755092	ZNF345	518207	ENSBTAT00000006317	1.261876	1.307744	-1.036349
12710135	SPG20	534027	NM_001077996	1.615365	**2.028270**	-1.255611
12908237	PHF8	513959	XM_002700122	1.855297	1.548595	1.198052
12700376	ATAD2B	518785	NM_001206750	**2.236434**	1.953552	1.144804
12711421	BRCA2	507069	XM_002684277	1.713493	1.641153	1.044078
12842687	HSD3B1	281824	NM_174343	**-2.106970**	**-2.044673**	-1.030468
12785958	GFM2	527467	NM_001102109	1.705412	**-2.314178**	**3.946627**
12869323	LOC786295	786295	XR_806897	-1.495979	**-2.261045**	1.511415
12882982	LOC104972838	104972838	XM_010807286	-1.061298	**2.096380**	**-2.224884**
12859545	LETMD1	514595	NM_001083430	1.777488	1.533885	1.158815
12904467	SCML2	523477	NM_001192580	**2.283014**	**2.046332**	1.115662
12907389	TCEANC	504389	NM_001024472	-1.207656	1.314268	-1.587185
12724157	CA2	280740	NM_178572	-1.220676	**-2.359711**	1.933118
12682510			GENSCAN00000013473	1.722364	1.629772	1.056813
12838206	FUBP1	513562	NM_001076846	-1.048204	**-2.042619**	1.948684
12780807	GCA	507139	XM_002685376	**2.933494**	1.320194	**2.222017**
12846879	PPCS	539294	NM_001102228	**2.120053**	1.900211	1.115693
12791907	FOXA1	503579	NM_001206029	1.865872	**2.207648**	-1.183172
12890318	CTSL	515200	NM_001083686	**-2.317726**	-1.640073	-1.413185
12745498	P2RX7	286814	NM_001206516	-1.015535	1.196997	-1.215593
12815042				-1.144510	-1.112288	-1.028969
12742074	TRPC3	286866	NM_001104960	-1.309422	-1.134967	-1.153709
12874543	ALPK1	524375	XM_002688076	1.355777	**2.329047**	-1.717869
12852612	TMEM196	100140139	NM_001206771	-1.555157	-1.310848	-1.186375
12887990	NUDT12	617720	NM_001046608	**2.005595**	1.436841	1.395837
12764961	KCTD11	539167	NM_001035421	1.778055	1.276789	1.392599
12701968	TMEM178A	538491	NM_001192028	1.760799	-1.038693	1.828930
12867117	NABP2	533842	NM_001101151	**2.131531**	1.551313	1.374017
12793939	LOC783909	783909	XR_813909	**-2.759582**	**-2.388624**	-1.155302
12802769	TBC1D7	532704	ENSBTAT00000022819	1.018743	-1.988389	**2.025657**
12809357	ZNF397	506448	NM_001075262	**2.437395**	**2.588740**	-1.062093
12803451	MBOAT1	541284	NM_001192857	1.330289	-1.530624	**2.036173**
12885292	MYO9B	513493	NM_001193096	-1.139452	-1.046847	-1.088461
12736829	RERE	535394	XM_001790418	1.266617	1.175350	1.077651
12771385	KRBA2	530088	XM_002695875	**2.087954**	1.224846	1.704667
12901165	HIVEP2	540396	XM_005199423	-1.053901	1.372323	-1.446293
12750702	TRAPPC2L	509955	NM_001098920	**2.474557**	**2.533549**	-1.023840
12862258	RNF41	614134	NM_001046525	**2.243856**	1.910341	1.174584
12689068	GNPNAT1	512299	NM_001075559	-1.007451	-1.000506	-1.006942
12775398	STRADB	506029	NM_001192081	**2.124863**	1.886942	1.126088
12876894	GPX3	281210	NM_174077	1.801789	**2.309787**	-1.281941
12799988	ATXN7	536833	XM_002696986	**2.014972**	1.496581	1.346384

ProbeID	Gene_Symbol	Gene_ID	mRNA Accession	OV24H/OV0H.fc	OV24R/OV0H.fc	OV24H/OV24R.fc
12718784	AHCY	508158	NM_001034315	1.787285	-1.226094	**2.191380**
12777446	INHBB	530430	NM_176852	1.095394	-1.886027	**2.065942**
12834765	SF1	541229	NM_001081614	1.382031	-1.514950	**2.093708**
12848182	LOC785516	785516	XR_805351	1.055027	-1.667666	1.759432
12716981	C13H20orf196	615129	NM_001046552	**2.233692**	1.065271	**2.096830**
12906577	MIR224	100313023	NR_031149	1.279092	**2.834983**	**-2.216403**
12840461	C3H1orf43	538459	NM_001015666	**2.088895**	1.500330	1.392290
12825959	GHITM	404143	NM_001034052	**2.402756**	**3.626505**	-1.509311
12872030	AREG	538751	NM_001099092	1.103166	-1.182106	1.304060
12891980	PTPLAD2	618814	NM_001076522	1.727936	**2.327288**	-1.346860
12868668	CEP83	618145	NM_001206525	**2.974774**	1.326284	**2.242938**
12836111	PIK3R3	286865	NM_174796	**-2.576317**	**-2.302386**	-1.118977
12827415	B3GALNT2	100125310	NM_001103343	**2.073862**	1.475823	1.405224
12864136	ITFG2	282275	NM_174562	**2.766074**	1.956113	1.414067
12807296	PMAIP1	100296226	XM_002697779	**4.050640**	**2.400791**	1.687211
12773566	LOC788196	788196	XM_002696017	1.131048	-1.259674	1.424751
12684570	YEATS2	514192	NM_001105367	**2.459014**	1.591900	1.544704
12723396	GDAP1	613472	NM_001101222	**2.492287**	1.865161	1.336232
12720367	RBM38	533516	NM_001206023	**2.133245**	1.208041	1.765871
12899023	IRAK1BP1	782235	NM_001110093	**2.794170**	**2.146625**	1.301658
12771193	ENDOV	526597	NM_001079777	**2.502344**	1.589783	1.574016
12826200	TET1	513640	XM_010820679	**-2.531969**	-1.454396	-1.740908
12735104	DNM3	506315	NM_001243284	1.193385	**2.167617**	-1.816360
12831635	SPTY2D1	539967	NM_001205738	1.861467	**2.893649**	-1.554499
12900979	FOXO3	535530	NM_001206083	**2.158756**	**2.147711**	1.005142
12771754	METRNL	534297	NM_001206050	1.203236	-1.001908	1.205532
12882137	CYSTM1	777789	NM_001078164	1.751025	**2.232747**	-1.275108
12855087	TRA2A	534586	NM_001046380	1.151217	**-2.307771**	**2.656745**
12720825	SEC61A2	614128	NM_001046524	**2.153734**	1.271252	1.694183
12788410	CEP72	617312	XM_005199617	1.307100	1.490390	-1.140226
12717330	PTER	782020	NM_001101282	-1.825700	1.208603	**-2.206546**
12708470				1.321192	**2.383431**	-1.804001
12707896	LOC614219	614219	XR_809762	-1.070741	-1.420472	1.326624
12762156	RNF157	507697	NM_001205618	-1.150459	-1.228459	1.067799
12744793	ANAPC10	783986	NM_001080357	1.289061	1.602084	-1.242831
12891928	LOC616903	616903	XM_005199316	1.140321	1.045034	1.091181
12905351	STK26	539445	NM_001163786	**2.295534**	**2.516782**	-1.096382
12835724	DBT	280759	NM_173905	**2.221586**	**2.123759**	1.046063
12823748	PROSC	509643	NM_001034357	**2.353732**	1.612244	1.459911
12700959	HS1BP3	526092	NM_001046280	1.634897	**2.276630**	-1.392522
12785900	SERF1A	526395	NM_001113301	**2.426135**	**2.612643**	-1.076874
12864864	ATF1	506967	NM_001075289	**2.428255**	1.493203	1.626206
12760968	CHRNB1	282179	NM_174516	1.817418	**2.718668**	-1.495896
12779978	MIR2353	100313161	NR_031070	1.752640	1.357921	1.290679
12792573	ACSBG1	515577	NM_001024548	-1.323960	-1.146368	-1.154918
12821704	ANKRD22	504991	NM_001081579	-1.574354	1.290750	**-2.032098**
12823401	LOC101908780	101908780	XR_232529	1.314797	-1.701256	**2.236806**
12873212	SPP1	281499	NM_174187	-1.009513	**-2.095392**	**2.075645**
12690161	JMY	528498	XM_002690458	**2.486386**	**2.488644**	-1.000908
12827055	ADIRF	613941	NM_001114513	-1.124450	1.333123	-1.499031
12681397	PLOD2	533642	NM_001101149	1.411491	-1.538975	**2.172250**
12729020	LOC101905272	101905272	XR_237504	-1.546793	1.355523	**-2.096714**
12795739			ENSBTAT00000000611	**3.581490**	**2.768837**	1.293500
12851791	GTPBP10	613957	NM_001080314	1.800503	**2.480430**	-1.377631
12862249	BHLHE41	613907	NM_001076297	-1.455899	1.454487	**-2.117585**
12748985	LOC104968473	104968473	XM_010815384	-1.222479	**-2.074969**	1.697345
12702649			ENSBTAT00000011846	1.247751	1.429073	-1.145319

ProbeID	Gene_Symbol	Gene_ID	mRNA Accession	OV24H/OV0H.fc	OV24R/OV0H.fc	OV24H/OV24R.fc
12864483	LTBR	504653	NM_001103228	2.431169	1.887360	1.288132
12861857	PIP4K2C	540005	NM_001076196	1.555221	2.567403	-1.650828
12772311	KRT31	539597 // 6184!	NM_001099099	1.220921	-1.263942	1.543173
12739608	ATF3	515266	NM_001046193	2.437834	1.946146	1.252647
12761115	STAT5A	282375	NM_001012673	1.256665	1.225253	1.025637
12770183	SYNGR2	513812	NM_001100358	1.416067	2.189422	-1.546128
12776899	SLC39A10	521004	NM_001205880	-1.295698	-2.456357	1.895778
12702331	AFTPH	540837	XM_001790625	1.679929	2.013813	-1.198749
12697491	ATG12	767903	NM_001076982	2.024408	2.220324	-1.096777
12846468	LRIG2	535493	NM_001192860	2.316425	1.466432	1.579634
12720838	MKKS	614288	NM_001206171	2.454672	1.339858	1.832039
12791835	TJP1	407102	XM_005196201	2.131974	1.801527	1.183426
12815109	TPT1	326599	NM_001014388	1.843452	2.347508	-1.273430
12900065	OR4A15	511653	XM_002683697	-2.188026	-1.335991	-1.637756
12865876	TMEM106C	514271	NM_001034461	1.922988	2.247576	-1.168793
12717971	CST3	281102	NM_174029	1.863390	2.295137	-1.231699
12752300	SEPW1	523694	NM_001163225	1.711617	2.203153	-1.287177
12848618	CHPF2	100616099	NM_001206923	-2.116837	-1.587449	-1.333484
12696534	ZBTB25	538889	NM_001164023	1.405906	1.452643	-1.033243
12784152	FAM171B	614438	NM_001206755	2.514985	2.765576	-1.099639
12871398	PLA2G12A	527214	NM_001098063	1.886301	2.952731	-1.565355
12756068	CFAP20	445424	NM_001003905	1.477451	-1.386887	2.049057
12806978	OARD1	768056	NM_001077113	1.343690	-1.210304	1.626274
12889316	DDX58	504760	XM_002689480	1.338577	1.724598	-1.288382
12859375	A2M	513856	NM_001109795	1.447717	1.260748	1.148300
12845808	DNALI1	529053	NM_001191299	1.555750	1.237467	1.257205
12790479	DNAJA4	528615	NM_001102120	1.082975	-1.396533	1.512411
12793470	ARRDC4	538517	NM_001192034	1.686217	2.054418	-1.218360
12747034	ULK1	526310	NM_001205927	2.208413	1.739859	1.269306
12727205	ATG13	507340	NM_001076812	1.545368	2.177888	-1.409301
12820687	STAMBPL1	538785	NM_001076129	2.173907	1.438954	1.510755
12889017	LPL	280843	NM_001075120	-2.260936	-1.665459	-1.357545
12734488	FMOD	281168	NM_174058	-1.208150	-1.422422	1.177355
12864197			NM_174719	2.695345	3.379360	-1.253776
12824753	PLAT	281407	NM_174146	2.477749	2.351905	1.053507
12816240	ROGDI	510492	NM_001075452	1.509185	2.229917	-1.477563
12732105	TIMM10B	514937	ENSBTAT00000004639	1.582121	2.319237	-1.465903
12774409	MAP2	281294	NM_001205807	2.069196	1.236644	1.673235
12691905	TMEM55B	615077	NM_001076359	1.785626	2.142804	-1.200030
12834190	TMEM135	523885	NM_001035348	1.643715	2.307803	-1.404017
12890108	ZCCHC7	511821	NM_001046100	2.078280	1.672180	1.242857
12712605	SPRY2	539090	NM_001076147	-1.161517	-2.133615	1.836922
12711245			GENSCAN00000015974	1.766385	2.151526	-1.218039
12687663	MAP2K5	100295732	NM_001206770	2.555455	1.768971	1.444600
12890642	TRIM32	521975	NM_001075824	-1.300053	-1.140787	-1.139610
12753538	KCNJ14	540740	NM_001206021	1.194271	1.044867	1.142988
12786659	ACTBL2	780991	XM_010816767	-1.671195	-1.569386	-1.064872
12890889	NR4A3	528877	XM_005199247	1.283844	-1.335824	1.714990
12828783	RHOBTB1	540513	NM_001082453	-2.052312	-1.653634	-1.241092
12872507	TRMT10A	616544	NM_001035477	3.126070	2.143785	1.458201
12711770	IFT88	514177	NM_001110786	2.164764	2.308187	-1.066253
12766656	LOC100140873	100140873	XM_001789469	-1.146304	-1.403122	1.224039
12792498	CPEB1	514174	XM_010817000	1.038924	2.159908	-2.078986
12830417	RBM14	511512	NM_001014901	-1.028249	-2.415329	2.348973
12864750	LARGE	506466	NM_001205588	-2.187950	-1.453821	-1.504965
12843848	LOC508547	508547	XM_005197743	1.780769	2.001845	-1.124146
12800563	PPARG-TSEN2	724061	NR_003096	2.378447	1.318703	1.803626

ProbeID	Gene_Symbol	Gene_ID	mRNA Accession	OV24H/OV0H.fc	OV24R/OV0H.fc	OV24H/OV24R.fc
12687319			ENSBTAT00000065519	-1.068473	1.081003	-1.155022
12712601	POLR1D	539061 // 6169	NM_001076144	1.486913	-1.697380	**2.523857**
12797808	MIR2370	100313422	NR_030980	**-2.020357**	-1.544329	-1.308243
12764421	ADPRM	534038	NM_001103279	1.845255	**2.864215**	-1.552206
12801900	CDKN1A	513497	NM_001098958	**7.203610**	**4.526262**	1.591514
12836092	PI4KB	286846	NM_174783	-1.027380	-1.073372	1.044766
12740137	RCOR3	532635	NM_001192733	1.200745	**2.031056**	-1.691496
12721420			XM_005215689	**2.001387**	1.140803	1.754366
12900397	TMEM242	517251	NM_001046228	1.818174	**2.245132**	-1.234828
12897474	KIAA1244	522232	XM_002690274	1.088288	**2.071520**	-1.903468
12878068	P4HA2	507327	NM_001034293	1.301964	-1.644273	**2.140785**
12809919	RIOK3	522917	NM_001075836	**2.514654**	**2.381169**	1.056059
12851902	RP9	100336120	XM_002704099	**2.723761**	**2.060965**	1.321595
12784595	LOC783612	783612	XR_082746	-1.073409	1.083275	-1.162798
12857852	HCFC2	505341	NM_001192948	**2.014302**	**2.059052**	-1.022216
12858048	SMDT1	506149	NM_001045937	**2.040567**	1.208092	1.689083
12770107	CTC1	513500	XM_002695868	1.684488	**2.128873**	-1.263810
12808553	SYT4	539867	NM_001098108	**4.423750**	**6.115329**	-1.382386
12864461	MGST1	493719	XM_005206974	-1.557606	1.369339	**-2.132891**
12848033	GSTM1	327709 // 7838	NM_001080354	**2.480808**	1.934579	1.282350
12762513	EIF1	509764	NM_001014884	**2.156871**	**2.373753**	-1.100554
12878199	CALR3	508555	NM_001038514	-1.520610	**-2.145926**	1.411227
12720619	TRIB3	538465	NM_001076103	**4.743625**	**4.921643**	-1.037528
12825680			NM_001206313	1.528694	**2.121802**	-1.387983
12779554	NKAIN1	618218	XM_002685664	**-2.247187**	**-2.566016**	1.141879
12774370	ECE1	281133	NM_181009	1.573318	**3.592056**	**-2.283109**
12725874	E2F5	539427	XM_001789547	**2.129921**	1.802938	1.181361
12827605	LYST	281072	NM_174020	1.760701	**2.121920**	-1.205156
12869915	CXCL2	281214 // 2817	NM_174299	**-5.067415**	**-7.533916**	1.486738
12798602	GXYLT2	507299	XM_002696971	**-2.002997**	-1.809448	-1.106965
12759305	B9D2	616609	NM_001040593	1.416882	1.166018	1.215146
12880668	CNN1	534583	NM_001046379	-1.328161	**-2.050392**	1.543783
12708347	NOTCH1	767866	XM_010810569	**2.035792**	1.101447	1.848289
12710632	N4BP2L1	616069	NM_001046574	**5.346668**	**5.620857**	-1.051282
12693124	LOC100298610	100298610	XM_002690554	**3.319900**	1.550539	**2.141126**
12822938	MGEA5	538561	NM_001206448	**2.195942**	**2.407474**	-1.096328
12855532	FAM126A	540584	XM_002686713	**2.066601**	1.323988	1.560891
12702463	MEIS1	613877	NM_001083507	**-2.112220**	**-2.058452**	-1.026120
12905544	MTMR1	541155	NM_001206067	**2.145256**	**2.401041**	-1.119233
12702977	TMEM182	618298	NM_001083784	**2.554445**	1.419930	1.798994
12718839	RBM39	508722	NM_001206504	**2.022935**	**2.118628**	-1.047304
12826575	ZNF25	101905074	XM_005193104	1.808132	**2.993369**	-1.655503
12852132	LOC101906257	101906257	XR_805947	1.332717	**2.153913**	-1.616182
12686567	TIPARP	540975	NM_001206048	**2.211814**	1.693150	1.306331
12906346	ZNF75D	100125581 // 6	XM_003585582	**2.060851**	1.212984	1.698993
12822991	RAB11FIP2	540709	NM_001192771	-1.446564	1.693796	**-2.450184**
12895145	SMARCA2	540904	NM_001099115	-1.520262	1.365491	**-2.075904**
12879171	FSTL3	515367	NM_001075710	1.587515	1.451928	1.093384
12861583	CALCOCO1	538675	NM_001046435	1.044831	**2.007514**	-1.921376
12678807	SLCO2A1	282468	NM_174829	**2.375119**	**4.566820**	-1.922775
12694122	DUT	507945	XM_005211801	**-2.037429**	1.121158	**-2.284281**
12725603	SPAG1	530104	XM_010812192	**2.053650**	**2.083631**	-1.014599
12731183	SMPD1	505097	NM_001075187	**2.223974**	**2.155108**	1.031955
12809844	METTL4	521222	NM_001205883	-1.045933	-1.643327	1.571160
12865340	TSPAN31	510619	NM_001037595	**2.066057**	1.431989	1.442789
12853718	SNX10	508836	NM_001075375	-1.094415	**2.076955**	**-2.273051**
12823470	NRG1	281361	NM_174128	-1.200221	**-3.354901**	**2.795236**

ProbeID	Gene_Symbol	Gene_ID	mRNA Accession	OV24H/OV0H.fc	OV24R/OV0H.fc	OV24H/OV24R.fc
12869844	KIT	280832	NM_001166484	-1.649776	**-2.030071**	1.230513
12849921	NUB1	511298	NM_001080906	1.505622	**2.135021**	-1.418032
12879860	RASGRF2	524957	XM_002689373	1.978120	**2.481375**	-1.254411
12881001	MKNK2	538519	NM_001205275	**2.388790**	1.770995	1.348841
12686922	GBE1	615745	NM_001122729	-1.121259	1.808495	**-2.027792**
12725842	OXR1	539092 // 7829:	NM_001076148	1.379045	**2.864374**	**-2.077070**
12708063	NFU1	615964	NM_001046566	1.816914	**2.326272**	-1.280343
12811007				1.654792	**2.323710**	-1.404231
12905959	HMGN5	767875	XM_002699936	**-2.108504**	-1.173160	-1.797286
12901148	PLAGL1	539761	NM_001103289	**2.990507**	**2.505398**	1.193625
12708452	LOC781337	781337	XR_809708	1.534416	**3.158720**	**-2.058581**
12888606	MIR23A	791041	NR_031347	1.571312	1.460822	1.075636
12806780	LOC616868	616868	XM_002702885	1.262655	1.031340	1.224285
12813341	TBC1D24	529002	NM_001046296	1.906478	**2.475414**	-1.298423
12679157	LRRC3	506054	ENSBTAT00000023769	1.310402	**2.215036**	-1.690348
12746135	TMEM120B	508790	NM_001101908	1.775924	**2.087896**	-1.175667
12757357	LOC515089	515089	XM_005192763	**3.255705**	1.890293	1.722328
12899003	SDHAF4	768071	NM_001077128	-1.030597	1.945202	**-2.004719**
12802761	ZFAND3	532641	NM_001102145	**2.375268**	1.871039	1.269491
12690032	GALNT16	526331	NM_001101127	1.510085	**-2.075300**	**3.133880**
12892785	SLC1A1	282353	NM_174599	-1.526206	**-2.053963**	1.345796
12813407	HS3ST2	532099	NM_001205994	**2.806788**	1.438355	1.951387
12849788	GPNMB	509600	NM_001038065	-1.265871	**2.770737**	**-3.507395**
12768215	UTP18	505846	NM_001098879	1.981812	**2.287117**	-1.154053
12714880	KIAA1217	513129	XM_010811217	-1.006293	1.370042	-1.378663
12713397	LOC618696	618696	XM_010811733	**-2.207877**	**-2.207311**	-1.000256
12797985	PPARG	281993	NM_181024	**2.283299**	1.533917	1.488542
12897211	AKAP12	513774	XM_010799018	**-2.659933**	**-2.174178**	-1.223420
12873825	BOD1L1	508527	NM_001192190	-1.155871	-1.089941	-1.060489
12792817	TRAPPC6B	521470	NM_001038134	1.385244	**2.084903**	-1.505080
12892865	FZD3	445419	NM_001192964	**2.606041**	**2.201673**	1.183664
12872713	LOC782855	782855	XR_800937	1.218284	**2.045169**	-1.678730
12703595	LOC100300896	100300896	XM_002691338	**-2.336696**	**-2.026472**	-1.153086
12750311	SPINT2	507484	NM_001045971	-1.167045	**2.420424**	**-2.824744**
12822057	MKI67	513220	XM_002698582	-1.830274	**-2.081105**	1.137046
12782756	GLB1L	532551	NM_001206002	1.299062	**2.288941**	-1.761995
12887334	PWWP2A	540114	XM_010798064	1.816007	**2.391573**	-1.316940
12779057	NABP1	613474	NM_001098124	**2.447790**	1.409321	1.736858
12889602	FAM206A	507081	NM_001045956	1.483639	**2.090314**	-1.408911
12697687	LOC785050	785050	XM_001253192	**-2.144201**	-1.846918	-1.160961
12711493	FRY	507622	NM_001205616	1.542029	**2.427801**	-1.574420
12836813	IQGAP3	505886	NM_001098880	-1.002804	-1.069050	1.066061
12902320	LOC783993	783993	XM_010802316	-1.361163	**-2.177179**	1.599499
12902073			XR_804038	1.595756	-1.258330	**2.007987**
12866764	LOC782591	782591	XM_001790444	**2.543244**	1.775998	1.432008
12910785	LOC788379	788379	XR_803524	-1.466554	-1.226596	-1.195629
12739170	PDPN	509732	NM_001033120	-1.380777	1.466910	**-2.025475**
12804650	BOLA-DQA2	282535	XM_005196597	-1.325549	-1.413361	1.066246
12851723	GSTK1	613498	NM_001079616	**2.066086**	1.566115	1.319243
12766875	LOC100336815	100336815	XR_238798	-1.291627	**-2.096714**	1.623312
12780223	ACVR2A	281598	NM_174227	**2.166716**	1.634909	1.325283
12904255	ARMCX3	516747	NM_001192453	1.166075	**2.399477**	**-2.057739**
12860348	NELL2	524622	NM_001102084	1.112627	**-2.014875**	**2.241804**
12868324	DESI1	614655	XM_002687946	1.613429	**2.321714**	-1.438993
12799597	SEMA3F	531141	XM_002697033	-1.851250	**-2.161825**	1.167765
12718246	PFKFB3	407183	NM_001077837	-1.727685	**-2.460105**	1.423932
12691926	CDKN3	615282	NM_001040582	1.898474	1.557249	1.219120

ProbeID	Gene_Symbol	Gene_ID	mRNA Accession	OV24H/OV0H.fc	OV24R/OV0H.fc	OV24H/OV24R.fc
12844440	KDM4A	512622	NM_001206316	1.760909	2.492459	-1.415439
12805748	BOLA	507917 // 5330	NM_001038518	1.680767	2.367903	-1.408823
12849526	STEAP2	504616	NM_001077847	-3.001326	-2.622204	-1.144581
12755342	LOC101907883	101907883	XM_010814968	-2.059737	-2.642732	1.283043
12902223	TSC22D3	100125309	BC148966	2.971147	2.735890	1.085989
12894704	ABCA1	535379	NM_001024693	-1.518693	2.164029	-3.286495
12718222	LOC404103	404103	NM_205786	-2.225223	-2.323259	1.044057
12892568	OMD	280885	ENSBTAT00000015704	-1.272592	2.350439	-2.991149
12825460	THAP1	538615	NM_001034648	2.164419	1.147028	1.886981
12898939	LYRM2	615821	NM_001113310	1.214414	2.163324	-1.781373
12903973	TCEAL4	513987	XM_002699844	1.202336	2.344548	-1.949994
12794992	NPRL2	508487	NM_001015547	2.200467	2.201245	-1.000354
12737971			GENSCAN00000023254	-2.405455	-2.338203	-1.028763
12899908	IFNGR1	508619	NM_001035063	2.053265	1.306204	1.571933
12813668	CALN1	538163	NM_001206115	-1.617292	-2.774331	1.715418
12895040	LZTS1	539634	NM_001192287	2.018173	2.152853	-1.066734
12762966	GLOD4	512334	NM_001101953	1.571040	2.092257	-1.331765
12696574	TGFB3	538957	NM_001101183	-1.652190	-2.473185	1.496913
12762696	IFI35	510697	NM_001075462	1.413106	2.158666	-1.527603
12906223	MXRA5	786844	XM_001254409	-2.122331	-2.647499	1.247448
12838632	LAMTOR5	516090	NM_001034517	2.025053	1.864967	1.085839
12827782	SLC25A16	282524	NM_174672	1.454366	2.429400	-1.670419
12910441	LOC781489	781489	XM_002700317	1.506405	-1.577358	2.376140
12727890	TAF10	514443	NM_001098968	2.025124	2.711009	-1.338688
12769776	SLC43A2	511955	NM_001075546	-2.142670	-1.059784	-2.021800
12834028	LOC784113	784113	XM_001252523	1.547994	-1.341252	2.076250
12864062	KITLG	281885	NM_174375	-1.170553	-3.003594	2.565962
12820005	ZRANB1	523338	NM_001101114	1.283346	2.108519	-1.642986
12825048	RWDD4	509865	NM_001076824	1.618806	2.895836	-1.788872
12766526	LOC100850190	100850190	XM_003585325	-2.084339	-1.875532	-1.111332
12683252	GYG1	280812	NM_001045867	2.237938	2.869979	-1.282421
12889619	FAM160B2	507256	XM_002689765	1.961313	2.454825	-1.251623
12899918	SMPD2	509018	NM_001075383	2.113728	2.468321	-1.167757
12825915	VDAC2	282120	NM_174486	-2.187298	-1.097834	-1.992376
12694383	ABHD4	509896	NM_001034368	-1.053507	1.907787	-2.009867
12852485			GENSCAN00000019568	-1.681781	-2.980822	1.772419
12901720			GENSCAN00000014068	-2.141854	-2.261328	1.055781
12802401	HIST1H2BJ	522960	XM_002697500	2.107671	3.940039	-1.869380
12855202	TAX1BP1	535589	NM_001046409	1.262646	2.009003	-1.591106
12777335	DLX2	528490	NM_001192662	2.091126	1.941740	1.076934
12682652	TMSB4	781334	ENSBTAT00000007124	-1.679777	-2.414509	1.437398
12792596	CKB	516210	NM_001015613	-1.942615	-2.359482	1.214591
12846597	LRRC52	536513	NM_001206099	-2.024282	-1.889730	-1.071202
12787220	DNAJC21	509302	NM_001192218	2.055658	1.413439	1.454366
12678716	SYNJ1	282087	NM_174468	1.144542	2.005511	-1.752240
12849736	MPLKIP	509064	NM_001075389	1.415783	2.478642	-1.750722
12712472	TGDS	534594	NM_001101159	1.898855	2.290671	-1.206343
12886461	UPF1	532933	XM_001789911	1.691238	2.010410	-1.188721
12765844	TRAF4	781568 // 7833(	NM_001101280	2.091865	1.599388	1.307916
12829973	SLC3A2	507107	NM_001024488	3.538799	2.436837	1.452210
12769692	MED11	511672	NM_001038084	-1.137850	-1.401518	1.231725
12723793	SLC10A5	104968462	XM_010812265	1.417118	2.310668	-1.630540
12780541	IGFBP5	404185	NM_001105327	-2.967442	-1.905184	-1.557562
12825130	VPS37A	513985	NM_001046161	1.899290	2.106430	-1.109062
12854729	LOC524050	524050	XM_602368	2.468218	1.992390	1.238823
12834738	ZBTB44	540532	XM_002699251	2.184238	1.669366	1.308424
12885658	PRDM6	519857	NM_001103255	2.195196	1.580751	1.388705

ProbeID	Gene_Symbol	Gene_ID	mRNA Accession	OV24H/OV0H.fc	OV24R/OV0H.fc	OV24H/OV24R.fc
12779112	TAF12	614227	NM_001034754	**2.019797**	1.555275	1.298675
12770228	INPP5K	514123	NM_001101986	1.351930	**2.270783**	-1.679661
12809364	MPPE1	506830	NM_001305022	1.748709	-1.339570	**2.342518**
12862594			ENSBTAT00000037671	1.582275	**2.008112**	-1.269130
12896090	C9H6orf120	100125289	NM_001103331	1.266064	**2.067275**	-1.632836
12897014	FRK	509227	XM_002690084	-1.849416	1.158927	**-2.143339**
12795056	PDZRN3	509083	XM_002696956	**-2.024829**	-1.751438	-1.156095
12871228	AGPAT9	519739	NM_001192514	**3.084700**	**2.418144**	1.275648
12751361	APLP1	513154	NM_001038095	1.458636	**2.085596**	-1.429826
12792605	STRN3	516375	NM_001098042	**2.449437**	**2.034988**	1.203662
12788177	BOD1	540063	NM_001076200	**2.003941**	1.679905	1.192889
12779277	PTMA	615626 // 78633	NM_001039953	-1.242986	**-2.030141**	1.633278
12719526	ATP9A	516579	XM_003583005	-1.225610	1.645470	**-2.016705**
12871178	NUDT9	517589	NM_001101096	1.643419	**2.054005**	-1.249837
12891530	FAM122A	539712	NM_001101196	1.421969	**2.607650**	-1.833830
12711664	INTS6	509695	XM_010799168	**2.186904**	1.608270	1.359786
12812425	SNRNP25	513526	NM_001166612	**2.003163**	**2.019055**	-1.007933
12862676	TAS2R10	664636	NM_001046626	**-3.815336**	**-4.406826**	1.155030
12678842	ADAMTS5	286805	NM_001166515	-1.783449	1.283933	**-2.289829**
12910377	BEX2	767968 // 76802	NM_001077087	1.009345	**2.582897**	**-2.558982**
12738390	LZIC	767886	BC151733	-1.341958	-1.080763	-1.241677
12708443	YPEL5	780856	NM_001079793	**2.608409**	**2.585154**	1.008996
12775752	CHN1	508266	NM_001075349	-1.954108	1.125066	**-2.198501**
12768248	PRPSAP2	506111	NM_001081513	1.598867	**2.550730**	-1.595336
12909762	CAPN6	539360	NM_001192231	**2.752438**	1.904497	1.445231
12754903	LOC789960	789960	XM_005219828	-1.896015	1.177960	**-2.233428**
12869909	GRO1	281212	NM_175700	**-3.583750**	**-2.906534**	-1.232998
12868425	C5H12orf5	615392	NM_001076370	**2.010940**	**2.267669**	-1.127666
12843090	NIT1	504199	NM_001038023	**2.034282**	1.923348	1.057678
12692985			GENSCAN00000032237	**-2.525642**	-1.823159	-1.385311
12690836	RORA	535597	NM_001192861	-1.618458	**-2.063466**	1.274958
12833412	AQP11	510038	NM_001110069	**3.311305**	**3.570881**	-1.078391
12786378	MSX2	540230	NM_001079614	1.222437	**2.105102**	-1.722054
12812568	CTF1	514803	NM_001192384	1.130766	**3.524650**	**-3.117047**
12907694	PIM2	508424	NM_001206378	1.806679	**2.216433**	-1.226800
12727811	LOC514057	514057	XM_001787715	-1.959696	**-2.031591**	1.036687
12772798	SRSF1	615796	NM_001076394	-1.446113	**-2.122229**	1.467540
12904823			ENSBTAT00000064281	**-2.152406**	-1.608861	-1.337844
12904630	HTATSF1	526910	NM_001206414	-1.767353	1.230163	**-2.174133**
12870826	C6H4orf19	511424	NM_001104973	-1.519946	**-2.033789**	1.338067
12844912	PPOX	515770	NM_001192426	**3.137793**	**3.221271**	-1.026604
12787557	SH3PXD2B	518356	XM_010816700	**-2.048162**	-1.394164	-1.469097
12710196	RB1	534712	NM_001076907	1.811846	**2.071032**	-1.143051
12717355	LOC782950	782950	XR_083823	**2.246501**	1.587614	1.415018
12808543	ABHD3	539795	NM_001076187	**2.203703**	1.934177	1.139349
12804265	MIR877	100313087	NR_031221	**-2.809863**	**-2.391640**	-1.174869
12752457	PVR	526865	XM_005195348	1.633447	-1.250106	**2.041982**
12722742	KLF10	522795	NM_001168462	**2.300871**	**2.396502**	-1.041563
12828057	ERO1LB	514361	NM_001206730	**-2.876371**	-1.323924	**-2.172611**
12847968	LOC782233	782233	XR_805429	1.714550	**2.407991**	-1.404445
12771139	LOC524719	524719	XM_010824247	**2.504374**	1.789058	1.399828
12889102	ATP6V1G1	281641	NM_174245	1.274967	**2.067719**	-1.621782
12789065	IGF1R	281848	NM_001244612	**-2.124098**	-1.874272	-1.133292
12842040	MIR186	791032	NR_031376	-1.480229	**-2.102608**	1.420462
12854683	SOSTDC1	523184	NM_001046265	-1.247396	**-2.173063**	1.742079
12896338	CNR1	100299449	NM_001242341	1.030718	1.344510	-1.304439
12727546	HBG	511735	NM_001014902	1.339338	-1.820658	**2.438476**

ProbeID	Gene_Symbol	Gene_ID	mRNA Accession	OV24H/OV0H.fc	OV24R/OV0H.fc	OV24H/OV24R.fc
12738945	TRAF5	507234	NM_001105340	-3.114304	-1.717774	-1.812989
12859746	CCDC134	516012	NM_001205826	1.612892	1.530179	1.054055
12846895	TCEANC2	539385	NM_001104992	2.371566	1.398703	1.695546
12875352	ANK2	539238	XM_010805941	1.544061	2.410914	-1.561411
12848065				-2.046786	-1.904867	-1.074503
12891999	LOC104968438	104968438	XM_003586368	-2.406506	-2.399028	-1.003117
12728123	SESN3	515914	XM_002692957	-2.165800	-1.407076	-1.539221
12885908	ZNF354A	524106	NM_001256565	2.447163	2.400126	1.019598
12901612	LOC100850276	100850276	XM_005211029	-1.112234	1.925389	-2.141483
12832994	LOC504490	504490	XM_002699110	-2.108753	-2.496246	1.183755
12877198	VCAN	282662	NM_181035	-1.467591	-3.125550	2.129714
12893495	HPGD	512259	NM_001034419	-2.260654	-1.530030	-1.477522
12831768	LOC613739	613739	XM_002699259	-2.067389	-1.892758	-1.092263
12902364	LOC100295153	100295153	XR_816739	-2.055943	-1.729134	-1.189001
12758513	RHPN2	533687	NM_001083470	1.297073	2.042520	-1.574714
12781058	SESN2	509863	NM_001024507	2.434136	3.013918	-1.238188
12718301	BEND7	504404	XM_005214313	2.445450	4.395600	-1.797460
12842414	LOC531090	531090	XM_010803765	-2.023650	-1.734644	-1.166608
12883575	ST8SIA4	407768	NM_001001163	-3.566577	-2.625896	-1.358232
12901609	LOC788043	788043	XR_802924	-1.720836	-2.002178	1.163491
12842026	SCLY	790815	NM_001083804	1.931471	-1.106520	2.137212
12805318	CYP39A1	511195	NM_001098938	-1.002234	2.278430	-2.283521
12887830	ADAMTSL5	100337439	XM_010800165	1.028349	2.151242	-2.091938
12792547	CCDC88C	515039	NM_001102017	-1.479634	-2.042775	1.380595
12725230	CA13	513850	XM_002692829	1.587042	2.416820	-1.522846
12818608	DNAH3	786654	NM_001099199	-1.778647	-2.223496	1.250106
12856483	MIR2284H	100313467	NR_031257	2.908187	1.651870	1.760542
12780953	ZNF593	508477	NM_001192186	1.946537	2.532022	-1.300783
12698356	ASS1	280726	NM_173892	-1.104689	1.833639	-2.025601
12836852	ARHGEF2	505940	NM_001098881	2.217094	1.793863	1.235933
12693774	TRD@	407199	BC113229	-1.435795	-2.097979	1.461196
12910177	MID1IP1	615572	NM_001076383	1.884576	2.229052	-1.182787
12734027	LOC790683	790683	XM_002693137	-2.017544	-1.915260	-1.053405
12690804	ATL1	535424	NM_001034631	-2.254566	-1.423192	-1.584162
12775056	HTR1D	407136	XM_005197607	-2.854801	-2.049867	-1.392676
12840828	PYGO2	540401	NM_001102242	1.722436	2.152555	-1.249716
12842868	DNAJC6	317659	NM_174836	2.088692	1.657766	1.259944
12681285	MECOM	532209	XM_003581740	-1.780843	1.180543	-2.102360
12836019	PTGER3	282330	NM_181032	-2.188557	-3.182168	1.454003
12825725			ENSBTAT00000025237	-1.449465	-2.780357	1.918196
12699109	MRPL53	505728	NM_001045926	-1.893388	1.335861	-2.529303
12885154	USE1	512890	NM_001075584	1.544083	2.412301	-1.562288
12836290	CRABP2	493998	NM_001008670	-1.090009	1.085590	-1.183303
12879073	PCYOX1L	514598	NM_001075673	2.257851	1.712887	1.318155
12829596	CTSC	352958	NM_001033617	-1.781028	-2.267056	1.272892
12862774	GLIPR1	767905	NM_001076984	-2.291766	-1.636042	-1.400799
12872081	BMP3	539527	NM_001192268	-3.176967	-2.214191	-1.434820
12703398			GENSCAN00000012309	-2.423799	-2.209760	-1.096860
12684439	NUDT16	512320	NM_001075560	-1.233434	1.625924	-2.005469
12756629	ETHE1	509150	NM_001034344	1.287355	2.266160	-1.760323
12720915	GGT7	615929	NM_001076401	1.546332	2.362198	-1.527614
12712593	KBTBD6	538461	NM_001205540	1.885922	2.058937	-1.091741
12774650	PRKRA	282875	NM_001045870	2.082173	1.168850	1.781386
12707871	SIRT3	614027	NM_001206669	2.659767	2.311982	1.150427
12892272	MIR27B	791000	NR_030910	3.012059	1.512222	1.991810
12786416	ADAMTS6	540722	NM_001193016	-1.473146	-2.535218	1.720956
12704122	ATP6V1B1	338059	NM_176654	1.122776	2.667004	-2.375366

ProbeID	Gene_Symbol	Gene_ID	mRNA Accession	OV24H/OV0H.fc	OV24R/OV0H.fc	OV24H/OV24R.fc
12811207				-1.861609	**-2.576424**	1.383977
12739704	KMO	515996	NM_001243298	**-4.359915**	**-6.045451**	1.386598
12856205	TPK1	788066	NM_001080370	**2.289179**	**2.067346**	1.107303
12782544	ARHGEF10L	529043	NM_001046297	**2.474025**	1.297892	1.906187
12785993	LOC530348	530348	XM_002696335	-1.140795	1.776380	**-2.026486**
12760429	ABR	515556	XM_005220104	**-2.013604**	-1.726847	-1.166058
12682788	LOC783884	783884	XM_001252341	**-2.200605**	**-2.745350**	1.247543
12698826	IL18R1	407221	XM_005193845	-1.486377	**-2.090561**	1.406481
12906798			GENSCAN00000043039	1.219661	**2.007987**	-1.646349
12691723	ISCA2	613290	NM_001038683	**2.070573**	**2.078208**	-1.003687
12901286	SLC16A10	541240	NM_001192847	1.459243	-1.673131	**2.441504**
12787924	FAM105A	534389	NM_001102171	**-2.457361**	-1.635226	-1.502766
12760005	LOC100848201	100848201	XM_010827389	**-2.033733**	**-2.290734**	1.126369
12786245	CPEB4	538794	XM_003583671	1.562439	**2.605445**	-1.667550
12901705			GENSCAN00000022835	**-2.214698**	-1.912368	-1.158092
12835544	LOC101903478	101903478	XM_010803745	**2.934165**	**3.891024**	-1.326110
12692693	TYRO3	788224	NM_001191228	1.257676	**2.410563**	-1.916681
12807083	LOC785639	785639	XM_001790501	**-2.109396**	-1.500933	-1.405389
12825721	MIR2403	100313193	NR_031302	1.346851	-1.495989	**2.014875**
12703615	MIR2295	100313460	NR_031092	1.587570	-1.331129	**2.113260**
12910512	ZXDB	783265	XM_005228277	1.928862	**4.156099**	**-2.154690**
12907723	SAT1	508861	NM_001034333	**2.184601**	**3.191556**	-1.460933
12809579	FAM210A	511554	NM_001080259	**2.021085**	1.438385	1.405107
12694950	LOC515823	515823	XR_082839	**-3.048015**	**-2.663826**	-1.144224
12684414	DPH3	511579	NM_001113299	**2.050904**	1.511373	1.356981
12762788	MBTD1	511415	NM_001206222	1.634523	**2.004080**	-1.226094
12746496	C17H4orf33	513251	XM_003587131	-1.919074	1.629648	**-3.127414**
12867326	XPOT	535264	NM_001206076	**2.195105**	**2.252317**	-1.026063
12899085	LOC782807	782807	XM_001253329	-1.810088	**-2.051416**	1.133324
12689424	TMEM62	515078	NM_001205497	**-2.042789**	-1.061703	-1.924068
12712812	SPRYD7	615298	NM_001038206	1.798657	**2.232918**	-1.241436
12678550	SST	280932	NM_173960	1.040603	**5.323850**	**-5.116120**
12896371	LAMA4	529670	NM_001205965	-1.137495	**-2.481375**	**2.181439**
12850876	SLC26A4	530241	XM_002686803	**-2.127472**	**-3.024591**	1.421683
12861991	LIMA1	540637	NM_001192754	**-2.586731**	**-2.159130**	-1.198043
12683292	ECE2	281134	NM_001314064	-1.196284	1.809737	**-2.164959**
12852487			GENSCAN00000032320	1.012365	**-2.334413**	**2.363279**
12793718	TM6SF1	616003	NM_001102295	1.722447	**2.567030**	-1.490338
12907669	MOSPD2	508293	NM_001101896	1.652798	**2.258899**	-1.366713
12892238	MAMDC2	788176	XM_002689639	**-2.128563**	**-2.064955**	-1.030804
12726242	PLEKHF2	782597	NM_001098150	1.767218	**2.004830**	-1.134455
12857056	MFAP5	281908	NM_174386	**-2.481341**	**-2.647902**	1.067126
12697937			NM_001105489	1.132507	**2.431304**	**-2.146833**
12739104	AGTRAP	508521	NM_001075363	1.804401	**2.002386**	-1.109723
12705362	CYP1B1	511470	NM_001192294	-1.108532	**-2.695158**	**2.431287**
12741511	SYT14	787880	XM_002694296	**-4.193631**	**-3.433405**	-1.221421
12787307	GPX8	511575	NM_001046088	**-2.047552**	-1.054128	-1.942413
12710821	SCEL	784362	NM_001102332	1.216124	**5.030773**	**-4.136728**
12730248	LOC783497	783497	XM_002692947	-1.585502	**-2.074624**	1.308496
12857869	FAM118A	505415	NM_001038035	**2.907925**	1.879580	1.547115
12690776	C10H15orf61	535251	XM_002690478	1.471594	**2.002455**	-1.360739
12697035			GENSCAN00000022865	**-2.330792**	**-2.455778**	1.053624
12906380	F8A1	101902099	XM_005227653	1.154109	**3.258640**	**-2.823511**
12801086			ENSBTAT00000007026	**2.497180**	1.771253	1.409839
12852253	TMEM60	767911	NM_001076988	-1.851956	1.255472	**-2.325079**
12841663	LOC782190	782190	XM_001250777	1.853831	-1.424406	**2.640608**
12684477	U2AF1	512680	NM_001080268	**-2.017935**	-1.812474	-1.113360

ProbeID	Gene_Symbol	Gene_ID	mRNA Accession	OV24H/OV0H.fc	OV24R/OV0H.fc	OV24H/OV24R.fc
12864664	ARSA	505514	NM_001075205	**2.023201**	**2.514288**	-1.242728
12721874	OPLAH	408006	NM_001001173	1.463041	**2.207939**	-1.509144
12679281	C1H3orf33	507040	XM_002684988	**-2.699253**	-1.180183	**-2.287148**
12799059	IRAK2	515034	NM_001075696	1.923202	**3.169642**	-1.648107
12767646	CYB561	317663	NM_174837	1.316886	**2.150065**	-1.632689
12685960	MME	536741	NM_001192884	**-2.194968**	**-2.936566**	1.337863
12816951	CYP3A24	517246	XM_005225269	-1.785886	**-2.200025**	1.231896
12729981	LOC616011	616011	XR_811737	**-2.331066**	**-2.773197**	1.189669
12814986	EARS2	100300732	XM_002698024	**2.300791**	1.810942	1.270494
12812085	GNPTG	508713	NM_001017428	-1.519830	1.320945	**-2.007611**
12896970	TNFAIP3	508105	NM_001192170	1.168891	-1.649639	1.928247
12738131	MIR2284N	100313140	NR_030870	-1.896737	-1.616126	-1.173632
12698798	ST3GAL5	404164	NM_205807	**-2.168955**	-1.431840	-1.514803
12892284	MIR23B	791065	NR_030945	**-2.534586**	**-2.513347**	-1.008450
12712820	PARP4	615359	XM_002691868	-1.476550	1.793751	**-2.648563**
12681492	CCDC39	534432	NM_001206056	1.416912	**3.648289**	**-2.574817**
12753926	CEBPG	617530	NM_001034801	**2.752953**	**2.197708**	1.252647
12872147	THAP6	539787	NM_001103290	**2.061751**	1.526450	1.350684
12739733	KIFAP3	518402	NM_001035329	1.959615	**2.522038**	-1.287007
12821440			ENSBTAT00000027838	**2.745807**	**3.556209**	-1.295142
12791646	WARS	281576	NM_174218	**3.618395**	**4.959965**	-1.370764
12755423				-1.589243	**-2.788154**	1.754391
12805924	LRFN2	524447	NM_001192595	-1.999653	**-2.063495**	1.031926
12716894			NM_001076302	1.439642	**2.469587**	-1.715418
12864984	PMCH	508013	NM_001080240	-1.544286	**-2.013130**	1.303599
12844669	LOC513884	513884	XM_002685947	**-2.513469**	-1.963149	-1.280325
12835571	MIR2887-1	100498812	NR_036352	-1.598235	**-2.523997**	1.579240
12814684	NME4	789324	NM_001077906	1.725698	**2.042392**	-1.183517
12732436	NCR3LG1	523303	NM_001206792	**2.793822**	**2.300743**	1.214313
12744054	PCDH18	539464	NM_001102229	-1.049855	**-2.834315**	**2.699720**
12812393	PHKG2	512670	NM_001046128	-1.748891	1.265037	**-2.212412**
12879187	STXBP2	515618	NM_001046208	1.273536	**2.102171**	-1.650657
12848001	ST6GALNAC3	782689	XM_003581999	**2.379107**	1.320697	1.801402
12786203	HCN1	538255	NM_001206580	-1.190667	**-2.315061**	1.944339
12678456	VEPH1	100337421	XM_005201776	-1.140954	**2.042166**	**-2.330016**
12683046	CD200R1L	100335828 // 5:	XM_002684750	-1.594186	**-2.709375**	1.699535
12784678	LOC785151	785151	XR_802935	**-2.150169**	-1.461703	-1.471003
12727383	FAM111B	509351	NM_001205654	**-2.036752**	-1.026625	-1.983928
12736315	PLA2G4A	525072	NM_001075864	**-2.275605**	**-4.131770**	1.815680
12815026	MIR1225	100313098	NR_031237	-1.727661	**-5.161821**	**2.987751**
12803613	EEF1E1	617105	NM_001040599	**2.998165**	**2.205935**	1.359136
12832191	E2F8	786629	NM_001191234	**-2.095581**	-1.518840	-1.379724
12811895	IQCK	506290	NM_001192088	-1.406656	1.431076	**-2.013032**
12768342	ITGB4	506995	NM_001193257	-1.318941	1.731269	**-2.283442**
12682849			GENSCAN00000007056	-1.438764	**-2.593913**	1.802876
12761288	CACNB1	327703	NM_175819	**2.669630**	1.762508	1.514677
12908490	RENBP	516214	NM_001046223	-1.069332	1.892732	**-2.023959**
12812034	LYRM1	508417	NM_001205637	1.597006	**2.510875**	-1.572238
12709043	POSTN	281960	NM_001040479	-1.518945	**-2.814191**	1.852727
12777810	RHBDD1	533985	NM_001077995	**2.066616**	**2.132905**	-1.032077
12699877	REG3G	513652	NM_001024534	-1.757519	**-2.015587**	1.146837
12870968	PYURF	100996942	NM_001038102	-1.059578	**2.244867**	**-2.378612**
12904911	HNRNPH2	534001	NM_001076008	-1.872025	1.085726	**-2.032507**
12871379	OTOP1	525643	NM_001206784	1.340304	1.742695	-1.300224
12750986	AARS	510933	NM_001101075	**2.199369**	1.901529	1.156632
12837225	SLAMF9	507627	NM_001302662	1.527095	-1.829437	**2.793725**
12724014	MIR2311	100313132	NR_031164	-1.454658	**-2.177209**	1.496715

ProbeID	Gene_Symbol	Gene_ID	mRNA Accession	OV24H/OV0H.fc	OV24R/OV0H.fc	OV24H/OV24R.fc
12899131	PLN	100125240	NM_001103319	**-3.371428**	-1.354866	**-2.488385**
12896057	TMEM246	786832	NM_001102342	**-2.064955**	-1.167652	-1.768468
12688778	PSME2	509857	NM_001014889	1.262322	**2.018957**	-1.599399
12857834	APOL3	505078 // 5129(	NM_001100297	**-2.176259**	**-3.004427**	1.380547
12895699	SLC25A51	781425	NM_001099394	-1.487459	1.359833	**-2.022697**
12853428	CYP51A1	505060	NM_001025319	**2.486558**	1.438385	1.728715
12744806	ZNF140	785125	XM_002694443	**3.189499**	**2.129050**	1.498086
12776426			ENSBTAT00000038024	1.306403	1.452270	-1.111656
12871600	UNC5C	533256	XM_002688134	**3.295963**	**2.573122**	1.280920
12902925	CITED1	282182	NM_174518	1.554758	**2.161226**	-1.390072
12687438	PPP2R3A	787607	XM_005195983	-1.629354	1.248010	**-2.033451**
12718453	PLTP	505640	NM_001035027	1.164088	**2.410496**	**-2.070716**
12857324	PLBD1	317710	NM_001101044	-1.454466	**-2.528199**	1.738231
12801369	NEU1	505554	NM_001083642	1.287935	**2.152674**	-1.671415
12842719	TUFT1	282104	NM_174479	1.705991	1.423261	1.198650
12729417	C15H11orf1	538766	NM_001038177	-1.770676	1.162886	**-2.059094**
12841009	FAM69A	541171	NM_001083500	**-2.215466**	-1.727529	-1.282448
12897007	HEBP2	509223	NM_001192213	1.543462	**2.540989**	-1.646292
12727554	LOC511823	511823	XM_002693233	**-2.083083**	-1.447085	-1.439502
12743278	ANKRD50	527956	NM_001205949	**-2.169391**	-1.582823	-1.370583
12718157	EIF6	286811	NM_174830	1.863907	**2.073230**	-1.112303
12700517	IL1RL1	520709	NM_001206302	-1.317333	-1.892706	1.436771
12865053	KCNA5	508960	NM_001015552	1.299935	**2.006429**	-1.543483
12688871	LOC510673	510673	XR_802492	1.521190	-1.042784	1.586272
12682512	FAM162A	617104	ENSBTAT00000014796	**-2.166190**	-1.390101	-1.558297
12878366	TSPAN17	509386	NM_001014880	1.315079	**2.052298**	-1.560588
12831992			ENSBTAT00000030370	1.157875	**2.231571**	-1.927298
12869954	CENPE	281681	XM_010805938	-1.093589	1.139989	-1.246679
12687084	PIGP	767951	NM_001077022	-1.202278	1.683414	**-2.023931**
12743116	LOC101909508	101909508	XM_005193460	-1.173128	1.812825	**-2.126676**
12842647	SARS	281476	NM_174175	**2.604976**	1.973423	1.320029
12825103	BRF2	512789	NM_001015582	**2.081365**	1.378195	1.510211
12740247	FLVCR1	533317	NM_001206019	**-2.276441**	-1.656468	-1.374274
12726994	LOC504551	504551	XM_001787386	1.339514	1.145820	1.169045
12764250	AKAP1	532072	XM_002695587	**2.242192**	1.771781	1.265502
12875211	SGCB	535372	NM_001102188	1.543259	**2.605897**	-1.688568
12829623	NOX4	378474	NM_001304775	**-2.147205**	-1.709139	-1.256308
12876963	ICAM1	281839	ENSBTAT00000013608	**2.311293**	1.604941	1.440111
12894613	ECM2	533916	NM_001034597	-1.393681	1.878251	**-2.617682**
12785247	SEMA5A	506636	XM_002696441	**-2.138976**	-1.285295	-1.664190
12832069	ASRGL1	767970	NM_001077035	1.432128	**2.013869**	-1.406208
12835631	LOC101904240	101904240	XR_234021	-1.764611	-1.276692	-1.382175
12699294	KRCC1	507166	NM_001075303	**2.172732**	1.594098	1.362985
12845896	OR10J1	530601	XM_002685875	**-2.336890**	**-2.645939**	1.132248
12681463	NCEH1	534212	NM_001123034	-1.415341	1.459253	**-2.065341**
12699923	EPCAM	514039	NM_001035290	1.056461	**2.350683**	**-2.225053**
12767419	VAMP2	282116	NM_174483	1.489017	**2.045013**	-1.373398
12726511	LOC100336916	100336916	XM_002693681	**-2.013827**	-1.767120	-1.139610
12696080	GALC	533428	NM_001206020	1.005805	**2.117849**	**-2.105627**
12905202	RAB39B	537560	NM_001076095	1.083643	**2.500125**	**-2.307147**
12848486	LOC100295719	100295719	XM_002686921	1.407417	-1.171681	1.649044
12759367	ETFB	617210	NM_001038582	1.329469	**2.133319**	-1.604640
12681995	FBXO25	540328	NM_001076211	-1.420521	1.418120	**-2.014470**
12848622	MIR196B	100313452	NR_030829	1.340174	**2.201993**	-1.643066
12866165	CKAP4	515784	XM_002687658	**-2.284059**	**-2.021477**	-1.129896
12752612	WDR62	530449	XM_002694947	1.011278	1.028705	-1.017233
12870010	CXCL5	281735	NM_174300	**-8.266691**	**-7.284702**	-1.134802

ProbeID	Gene_Symbol	Gene_ID	mRNA Accession	OV24H/OV0H.fc	OV24R/OV0H.fc	OV24H/OV24R.fc
12693035			ENSBTAT00000064250	1.790820	**2.127177**	-1.187823
12900957	TPD52L1	534629	NM_001076033	**2.035679**	1.854513	1.097689
12731429	LOC507428	507428	XM_002693191	**-2.130748**	**-2.708211**	1.271014
12855561	ETV1	540846	NM_001046492	**-2.212381**	**-3.892292**	1.759323
12734319			ENSBTAT00000034028	-1.266994	**2.096481**	**-2.656230**
12783701	TYW5	539498	XM_002685484	**2.104577**	1.595490	1.319078
12883735	SPRY4	504593	NM_001081512	-1.455082	-1.585766	1.089813
12806109	CYP39A1	511195	NM_001098938	-1.513638	1.547501	**-2.342355**
12791559	LOC100301305	100301305	XM_005221785	1.119504	-1.804851	**2.020539**
12875732	LOC615303	615303	XM_002688321	**-2.083935**	**-2.067661**	-1.007870
12832525	CLCF1	100336481	XM_005193325	**2.206302**	1.773894	1.243762
12719021	ITGA8	511976	XM_002692081	-1.785874	**-2.548468**	1.427015
12877798	MCOLN1	505738	NM_001075222	-1.234332	1.460052	-1.802189
12890470	RECK	517232	NM_001192465	-1.010704	**2.378480**	**-2.403939**
12817119	TEKT5	521940	NM_001046259	**-2.026598**	**-2.211262**	1.091120
12709775	LOC522140	522140	XR_810126	-1.943639	**-2.042746**	1.050991
12909398	SMARCA1	535439	NM_001191542	-1.031690	1.467723	-1.514236
12844355	LOC512286	512286	XM_003581921	-1.725339	1.171015	**-2.020399**
12829688	SLC25A22	504371	NM_001075159	**2.606728**	1.926537	1.353064
12681445	NSUN3	533791	NM_001076001	1.150228	**2.054789**	-1.786418
12797711	LOC100138407	100138407	XM_010799506	**-2.184253**	**-2.160372**	-1.011054
12855735	ZC3HAV1	614589	XM_003582128	**-2.278477**	1.118178	**-2.547743**
12688364	SLC7A7	504220	NM_001075151	1.825549	**2.689131**	-1.473054
12840420	HIST2H2BE	537985	NM_001099384	1.092346	**2.427632**	**-2.222402**
12910691	LOC104968697	104968697	XM_010799727	**2.314451**	1.708014	1.355054
12875026	AP1AR	533664	NM_001081532	1.419478	**2.340262**	-1.648678
12683322	AGTR1	281607	NM_174233	-1.207774	**2.450541**	**-2.959698**
12745970	GATSL3	506974	NM_001081514	-1.686754	1.410230	**-2.378711**
12853998	SLC26A3	512856	NM_001083676	**2.167347**	1.574507	1.376524
12875228	LOC536367	536367	XM_002688145	-1.888852	**-2.030352**	1.074913
12700406	ADD2	518896	NM_001099009	-1.182188	1.878824	**-2.221124**
12882838	MIR2454	100313227	NR_030812	**2.305724**	**2.346499**	-1.017684
12872629	LOC786899	786899	XM_010799338	-1.490876	1.342358	**-2.001290**
12816697	C25H16orf13	514636	NM_001038106	1.696981	**2.241244**	-1.320725
12773826	CD302	100126272	NM_001110191	-1.375895	1.915047	**-2.634903**
12736229	MXRA8	522392	NM_001075830	1.124793	-1.902716	**2.140162**
12872974	MIR2447	100313222	NR_031260	1.702837	1.456313	1.169280
12698660	EPAS1	282711	NM_174725	-1.565008	1.508568	**-2.360921**
12820938	RPP30	615098	NM_001035461	-1.352754	1.491992	**-2.018299**
12878593	LONP1	510796	NM_001015569	1.995057	**2.786531**	-1.396717
12846227	CGN	533725	NM_001192786	1.638391	**2.297874**	-1.402519
12871619	NSUN7	533295	XM_002688230	1.065854	**3.261329**	**-3.059827**
12779988	CRYGA	100335856	NM_001243570	-1.734560	**-2.102360**	1.212042
12692366	VPS13C	783566	XM_001251843	-1.897329	1.078256	**-2.045807**
12808614	TMEM241	614734	XM_002697719	1.448872	**2.030352**	-1.401333
12805255	BOLA-NC1	510417	NM_001105616	1.383910	**3.967169**	**-2.866639**
12880685	WIZ	535200	XM_003582372	**2.049213**	1.839648	1.113916
12802930	TMEM63B	534891	NM_001163939	-1.167498	1.348514	-1.574387
12760792	SERPINF1	281386	NM_174140	1.530932	**4.847766**	**-3.166546**
12803670	LOC617875	617875	NM_001099724	**2.909780**	**2.306571**	1.261517
12837217	LOC104968660	104968660	XM_005197920	1.431363	**2.039817**	-1.425087
12742553	ACAD10	511425	NM_001192292	1.171527	**2.719064**	**-2.320957**
12873015	LOC100336621	100336621	XM_002688163	**-2.317967**	-1.500798	-1.544489
12846183	DDR2	533523	NM_001083720	**3.268095**	**3.201016**	1.020956
12829936	SLC37A2	506687	NM_001024486	1.350600	1.054537	1.280751
12758642	FAM192A	534394	NM_001014944	-1.911281	**-2.091764**	1.094430
12887049	NWD1	537999	NM_001102210	1.167312	1.012793	1.152567

ProbeID	Gene_Symbol	Gene_ID	mRNA Accession	OV24H/OV0H.fc	OV24R/OV0H.fc	OV24H/OV24R.fc
12718608	PPDPF	506990	NM_001075294	-1.039882	**2.343557**	**-2.437023**
12734888	MTHFR	497032	NM_001011685	1.615958	**2.130246**	-1.318255
12866308	ALDH1L2	516864	NM_001191391	**5.735543**	**9.667347**	-1.685516
12819767	DPCD	516908	NM_001075745	-1.062410	1.998767	**-2.123509**
12797682	LOC789829	789829	XM_005222618	-1.098748	**-2.082895**	1.895699
12716231	UCKL1	534046	XM_002692400	**2.355413**	1.925763	1.223106
12826182	ZNF32	512392	NM_001046119	-1.081685	1.213042	-1.312129
12826569	DDIT4	529235	NM_001075922	**2.037006**	1.122449	1.814787
12848889	NPVF	281451	NM_174168	**-10.138413**	**-3.609027**	**-2.809182**
12680157	PLCH1	513565	XM_010801458	**-2.694486**	**-2.596053**	-1.037916
12828047	OR5L2	512400	XM_002698777	-1.652385	**-2.654941**	1.606733
12735280	NOC2L	508638	NM_001034326	1.403015	**2.650124**	-1.888879
12907860	LOC509805	509805	XM_002699648	**2.169135**	1.655859	1.309976
12848331	BMP8A	788948	NM_001206869	1.772211	**2.046857**	-1.154974
12871075	BDH2	515321	NM_001034488	-1.451234	1.926564	**-2.795895**
12828451	ANXA7	533360	NM_001075991	-1.592463	1.340991	**-2.135479**
12816081	ARPC1A	508402	NM_001075359	-1.541345	1.376686	**-2.121949**
12704737	RRM2	508167	NM_001244181	-1.848263	**-2.089098**	1.130303
12779857	LOC100137987	100137987	XR_804899	-1.234477	**-2.072325**	1.678706
12892676	ALDH1A1	281615	NM_174239	-1.751863	**-3.286427**	1.875961
12700208	OLFML2A	516730	XM_002691603	**-3.376995**	**-2.353928**	-1.434621
12739991	IL24	526285	XM_010813062	**-2.071262**	-1.736100	-1.193055
12892762	VLDLR	282123	NM_174489	**2.421633**	**2.707648**	-1.118108
12835617	MIR2414	100313201	NR_031310	**-2.294739**	**-2.063509**	-1.112056
12742473	TMEM154	510523	XM_002694322	1.588450	**3.003095**	-1.890581
12877266	NDUFA11	326346	NM_175718	-1.220540	-1.354218	1.109523
12862666	ADM2	618896	NM_001193200	**2.031098**	**2.467055**	-1.214641
12870423	SEC24D	504422	XM_002688058	-1.622423	1.281586	**-2.079274**
12804072			GENSCAN00000042013	-1.953810	**-2.281780**	1.167862
12726834	MMP7	286794	NM_001075130	1.166592	**2.020119**	-1.731641
12874466	SGMS2	520954	NM_001205877	**-2.404822**	-1.642098	-1.464482
12701582	INPP4A	535487	NM_001206848	1.879072	**2.090459**	-1.112496
12702319	PHYHD1	540828	NM_001076243	1.503839	**2.121640**	-1.410816
12692930	LOC100296102	100296102	XM_002690566	**-2.035679**	-1.873323	-1.086667
12808208	SERPINB5	534323	NM_001098080	**-3.422641**	-1.465802	**-2.334996**
12789434	TRAF3	506182	NM_001205586	-1.015401	-1.099220	1.082547
12853822	NFE2L3	510807	NM_001077899	**-3.329995**	**-2.781417**	-1.197230
12864745	C1QTNF6	506413	NM_001101872	**-2.121170**	**-2.772293**	1.306965
12683407	ST6GAL1	282073	XM_005201456	**-2.949090**	**-2.000707**	-1.474024
12701879	ALK	536642	XM_002691492	-1.976955	**-2.207311**	1.116521
12735094	MR1	506206	NM_001190298	-1.575795	1.769314	**-2.788076**
12801335	ECI2	505355	NM_001034242	1.090772	**2.130792**	-1.953471
12871498	NPFFR2	530560	XM_002688346	**-8.026884**	**-10.102007**	1.258522
12842441	MIR197	100313015	NR_030830	**-2.794732**	-1.208092	**-2.313344**
12857400	STAT6	353105	NM_001205501	-1.094407	1.629207	-1.783016
12819230	HOGA1	506001	NM_001075237	1.779004	**2.077286**	-1.167668
12872195	PPM1K	540329	NM_001046474	1.772186	**2.741908**	-1.547190
12730503	LOC100140748	100140748	XM_001787493	**-2.502084**	**-2.707329**	1.082030
12803467	LOC574091	574091	NM_001025347	-1.504089	1.346991	**-2.025994**
12795198	THOC7	510250	ENSBTAT00000046670	**-2.186359**	-1.996371	-1.095166
12739457	LOC514121	514121	XM_010800116	-1.999750	**-2.410295**	1.205298
12855679	AGMO	613658	NM_001192973	-1.372589	1.534693	**-2.106503**
12824834	VEGFC	282122	NM_174488	1.276152	-1.686135	**2.151764**
12699276	PRKCE	507041	NM_001111120	1.485388	**2.394708**	-1.612177
12779933	HOXD3	100295744	XM_010801922	**2.272358**	1.560037	1.456605
12848620	MIR2422	100313434	NR_030879	-1.481922	-1.403666	-1.055751
12771467	ASGR2	531519	NM_001075952	**2.084252**	**2.328192**	-1.117039

ProbeID	Gene_Symbol	Gene_ID	mRNA Accession	OV24H/OV0H.fc	OV24R/OV0H.fc	OV24H/OV24R.fc
12881959	LOC618071	618071	NM_001206718	1.128957	**2.226843**	-1.972479
12866697	LOC524576	524576	XM_002687704	**-3.580001**	-1.735186	**-2.063180**
12764948	NAGLU	789125	NM_001102226	-1.385734	-1.314606	-1.054106
12830405	ALDH3B1	511469	NM_001075518	1.299863	**2.128829**	-1.637733
12894591	C8H9orf40	533577	XM_002689652	1.866027	**2.524294**	-1.352764
12824974	RPL15	507151	ENSBTAT00000020729	**-2.758225**	**-2.056285**	-1.341363
12882286	PALM	786096	XM_010800088	-1.143844	**-2.088316**	1.825700
12688861	SRSF5	510474	NM_001098929	-1.984795	1.235864	**-2.452937**
12786629	PPAP2A	617172	NM_001080329	1.315727	**2.192049**	-1.666036
12829250	ANO9	512371	XM_003588068	-1.498906	1.388002	**-2.080485**
12741058	FAM129A	614787	NM_001191282	1.291260	**2.517008**	-1.949265
12726622			ENSBTAT00000010545	-1.377059	1.511719	**-2.081726**
12870149	PDGFRA	282301	NM_001192345	**-2.739267**	**-2.159040**	-1.268743
12801040				**-2.270956**	**-2.409126**	1.060842
12850240	STEAP1	515156	NM_001205806	-1.724514	**-2.437378**	1.413371
12712871	SAP18	615692	NM_001035467	1.089760	**2.189528**	**-2.009184**
12713867	PLCB1	287026	NM_174817	-1.174877	-1.452502	1.236301
12738576	PTGS2	282023	NM_174445	-1.335074	**-2.036187**	1.525149
12880368	LOC789086	789086	XM_001255941	-1.397356	**-2.434423**	1.742163
12880677	MRI1	534734	NM_001046387	**2.404905**	**2.795875**	-1.162572
12713409	SLC32A1	100301361	XM_002692459	-1.245720	-1.352745	1.085914
12735535	GLRX2	513762	NM_001040523	1.616619	**2.028566**	-1.254820
12779348	STPG1	616738	NM_001102304	1.141911	**2.025152**	-1.773476
12801306	RNF39	504845	XM_003583890	1.255986	**2.017460**	-1.606276
12829890	RPS6KB2	506083	NM_001205582	-1.385340	1.473186	**-2.040864**
12681617	SLC9A9	535743	NM_001076068	1.656077	**2.988373**	-1.804489
12869291	NCAPD2	786159	NM_001206711	1.270706	1.153070	1.102020
12825905	SFTPD	282072	NM_181026	-1.505132	**-2.266129**	1.505602
12852647			GENSCAN00000001951	1.584360	1.045577	1.515296
12841059	LOC613418	613418	XM_002686600	-1.367281	-1.747764	1.278277
12713857	BPIFA2A	286882	NM_174803	-1.721922	**-4.129966**	**2.398463**
12853311	GARS	408010	NM_001097566	**2.190986**	**2.608807**	-1.190700
12908754	LOC522586	522586	XM_010799369	1.129512	-1.304421	1.473360
12786020	C1QTNF3	531659	NM_001101138	-1.519672	**-2.448724**	1.611350
12811798	TAF6	505638	NM_001015525	1.237759	1.749097	-1.413116
12889086	TLR4	281536	NM_174198	1.021154	**2.802433**	**-2.744380**
12857816	TOM1	504912	NM_001035015	1.259813	1.445872	-1.147688
12682147	XRN1	540834	NM_001206035	**-2.536589**	-1.142584	**-2.220047**
12827996	ANK3	511203	NM_001105620	-1.364895	1.349860	-1.842417
12751650	PPP1R15A	514688	NM_001046178	**2.359907**	**2.274927**	1.037355
12815049				1.333169	**3.182411**	**-2.387102**
12836043	SLC6A9	515755 // 2823(	NM_001242342	**2.056969**	1.530964	1.343578
12725675	IMPAD1	532797	NM_001046335	**-2.202207**	-1.673734	-1.315745
12864124	CYP2D14	282211	NM_174529	-1.817481	**-2.086912**	1.148245
12892163	LOC785403	785403	XM_010798665	**-2.702978**	-1.695229	-1.594462
12710595	RGCC	614348	NM_001102276	**2.532215**	1.340991	1.888316
12807934	POLI	515909	NM_001024692	-1.304195	1.322438	-1.724717
12796146	OXSR1	526949	NM_001075892	**-2.045296**	-1.268066	-1.612926
12883748	POLRMT	504757	NM_001205551	1.087315	1.038902	1.046600
12884486	SPC24	509117	NM_001075391	**-2.323082**	-1.687468	-1.376667
12710773	SLC46A3	781117	NM_001103303	**-3.239340**	-1.430312	**-2.264779**
12707523	OXER1	786807	XM_005193887	**-2.012139**	-1.939373	-1.037521
12909086	LOC531038	531038	XM_005200747	-1.796925	1.162588	**-2.089083**
12708898	LOC100337053	100337053	XM_010810913	**-2.082491**	**-2.312110**	1.110262
12804243	LOC100298767	100298767	XM_002702931	**-2.040638**	**-2.300664**	1.127424
12892432				-1.754670	**-2.390745**	1.362504
12678900	CLDN1	414922	NM_001001854	1.332190	-1.864282	**2.483578**

ProbeID	Gene_Symbol	Gene_ID	mRNA Accession	OV24H/OV0H.fc	OV24R/OV0H.fc	OV24H/OV24R.fc
12831035	FADS2	521822	NM_001083444	-2.780530	-1.706606	-1.629275
12709067	TRPC4	282102	NM_174478	1.360974	-1.534299	2.088142
12851128	KLHL7	534697	NM_001076037	1.881300	2.492926	-1.325108
12827718	ANXA8L1	281627	NM_174241	-2.743352	-1.263933	-2.170489
12789104	IDH3A	282446	NM_174644	-3.034860	-2.163729	-1.402606
12909652	LRCH2	100336559	XM_003588142	1.127791	-1.015852	1.145669
12908989	LOC527430	527430	XR_802762	-1.346935	-1.152359	-1.168850
12774061	FBXO42	100848477 // 52	XM_005197658	-2.209929	-1.350384	-1.636519
12723779			GENSCAN00000029163	-2.401058	-1.648850	-1.456201
12698714	SLC1A4	326577	NM_001081577	3.292698	2.031760	1.620613
12720944	MRGBP	616297	NM_001206561	1.322447	2.023075	-1.529797
12848893	SCIN	281478	NM_174177	-1.094582	1.835445	-2.009045
12840641	MYCBP	539291	NM_001038565	1.435029	2.183209	-1.521369
12824734	MIR2398	100313428	NR_031172	-1.224922	-1.503349	1.227302
12906724			GENSCAN00000004138	1.722376	2.145583	-1.245712
12906742	TENM1	100336965	NM_001256555	-2.585870	1.058330	-2.736705
12749523	CDH1	282637	NM_001002763	-1.208318	1.669667	-2.017488
12834822	MACROD1	613568	NM_001046509	-1.802276	1.131369	-2.039040
12885558	HSPA9	517535	NM_001034524	1.460052	2.302801	-1.577205
12794421	TLR9	282602	NM_183081	-2.075271	-1.679638	-1.235547
12763389	CYB5D2	515356	XM_002695757	1.045846	1.823702	-1.743758
12842734	VCAM1	282118	NM_174484	1.018390	1.023698	-1.005212
12887608	CD74	613384	NM_001034735	-2.045807	-1.474484	-1.387473
12894487	CNTLN	532280	NM_001192721	1.278303	2.541975	-1.988554
12729535	RNF26	539611	XM_002693026	-1.219382	1.241996	-1.514467
12848511			ENSBTAT00000023762	-1.919380	-2.225285	1.159377
12837750	CC2D1B	511350	XM_010803562	1.504506	1.548230	-1.029062
12897765	LAMA4	529670	NM_001205965	-1.190403	-2.596521	2.181212
12841065	LINGO4	613529	XM_002684068	-2.233428	-2.004052	-1.114456
12708750	MIR19A	100170926	NR_030794	1.217456	-1.128996	1.374503
12906107	PABPC1L2A	784438 // 5210	XM_010821834	-1.921896	-2.288750	1.190882
12824697	LOC100336146	100336146	XM_010799201	-1.362645	-2.238326	1.642633
12901037	LCA5	537291	XM_005210689	1.740667	1.275612	1.364573
12787132	CENPH	505284	NM_001034236	-1.037463	1.844117	-1.913203
12907898	DDX3X	510093	NM_001192962	-1.484565	1.354706	-2.011149
12763074			ENSBTAT00000014061	1.488367	2.587107	-1.738219
12905875	TSPYL2	617179	XM_002700120	-1.164645	1.755242	-2.044234
12806486	BLA-DQB	539241	NM_001034668	-1.121112	-2.267590	2.022627
12774447	YARS	281581	NM_174220	2.671759	2.921663	-1.093535
12906911	LOC782803	782803	XM_001787351	-1.020864	1.980288	-2.021603
12847429	PIFO	615099 // 6174	XM_002686144	-2.041005	-2.989698	1.464817
12796360	WNT7A	533782	NM_001192788	1.373779	2.150467	-1.565366
12864151	SLC2A3	282358	NM_174603	2.015866	2.078107	-1.030875
12819189	TLX1	505304	NM_001192057	1.179079	-1.165089	1.373731
12735234				-1.890175	-2.369233	1.253446
12887709	HAVCR1	613999 // 6148	XM_010798531	1.819977	1.509457	1.205716
12777248	CALCRL	527140	NM_001102107	3.554385	3.048353	1.166002
12805515	GTPBP2	513605	NM_001083421	2.499830	2.695420	-1.078241
12770993	PLD2	522159	NM_001075827	-1.120754	1.146813	-1.285295
12871633	ELOVL6	533333	NM_001102155	-2.247529	-1.749460	-1.284699
12765895	ULBP27	785947	NM_001168616	1.451083	2.486747	-1.713718
12703851	ACTG2	281595	NM_001013592	1.645356	2.866539	-1.742200
12749590	HAS3	286824	NM_001192867	-1.552195	-2.298623	1.480885
12713731	PROCR	282005	NM_174437	2.011818	1.984341	1.013847
12723461	LOC101903806	101903806	XM_010812106	-1.872713	-2.339370	1.249187
12713107	RCBTB1	785222	NM_001191212	2.044474	1.827270	1.118868
12815225	TNP2	281538	NM_174200	-1.897119	-1.882918	-1.007542

ProbeID	Gene_Symbol	Gene_ID	mRNA Accession	OV24H/OV0H.fc	OV24R/OV0H.fc	OV24H/OV24R.fc
12909019	LOC528342	528342	XM_002699647	-1.536843	**-2.132314**	1.387464
12856851	KRT8	281269	NM_001033610	**-4.493314**	**-3.036964**	-1.479541
12731267	CD59	505574	NM_001037446	**-2.783345**	-1.911029	-1.456464
12785148	SEPP1	282066	NM_174459	-1.107756	-1.149702	1.037866
12908119	CXHXorf57	511907	XM_001789923	**-2.133970**	**-2.238776**	1.049113
12896865	ABRACL	505914	NM_001166561	**-3.717570**	**-2.693944**	-1.379973
12807507	DSC3	281129	NM_001178103	**-2.317854**	-1.436920	-1.613071
12908260	CA5B	514494	NM_001080908	**-2.128534**	-1.689469	-1.259883
12895307	C8H9orf152	614478	NM_001206177	1.676706	1.857780	-1.107994
12878948	ERAP2	513572	NM_001075628	-1.874272	1.627728	**-3.050805**
12711148	EDNRB	281750	NM_174309	**-2.482322**	**-3.580845**	1.442539
12715490	MKX	521239	NM_001192534	1.342852	**2.217494**	-1.651332
12887402	LMNB1	540643	NM_001103295	**-2.037274**	**-2.484645**	1.219593
12832203	LOC787455	787455	XM_001254846	**-2.923649**	-1.877171	-1.557476
12755837	SLC1A5	282355	NM_174601	**2.178205**	1.299791	1.675812
12875869	PROM1	618054	NM_001245952	-1.541570	**-2.112220**	1.370175
12843247	PHGDH	505103	NM_001035017	**3.305664**	**3.883291**	-1.174739
12882175			ENSBTAT00000054971	**-3.268390**	**-2.664269**	-1.226749
12905311	TAF9B	538983	NM_001192149	-1.384908	1.547737	**-2.143473**
12859619	USP18	515202	NM_001017940	**2.165200**	1.785836	1.212429
12896617			ENSBTAT00000064900	-1.088861	1.003075	-1.092210
12848599				1.783091	**2.230010**	-1.250643
12848438	LOC101904529	101904529	XR_225878	1.934539	**2.363181**	-1.221573
12681567	CD200	534910	NM_001034620	-1.563501	**-2.474025**	1.582362
12737673	C16H1orf21	781721	NM_001081547	-1.514383	-1.485090	-1.019725
12690936	KLHDC1	537136	NM_001192897	1.665436	**2.409560**	-1.446805
12894605	CD274	533834	NM_001163412	-1.365595	-1.263005	-1.081227
12903493	GPRASP1	100124524 // 5(	XM_010800428	-1.637790	1.313103	**-2.150586**
12780852	MARCH7	507865	NM_001100317	-1.365699	1.698569	**-2.319735**
12684718	CCDC80	515235	NM_001098982	-1.573012	**-2.603658**	1.655205
12685704	BDH1	534090	NM_001034600	-1.885399	**-2.211845**	1.173144
12876521	MIR2456	100313300	NR_030809	1.272010	1.138031	1.117729
12806652			GENSCAN00000024182	1.198052	**2.342469**	-1.955232
12802848	UHRF1BP1	534225	XM_002697198	1.826359	**2.170489**	-1.188424
12852542	LOC789101	789101	XM_010804711	-1.536215	**-2.292021**	1.491992
12764323	LRRC59	532659	NM_001034578	**-2.336307**	**-2.224730**	-1.050153
12682962	LOC100295890	100295890	XR_804437	**-3.878396**	**-2.275100**	-1.704714
12811259				1.410640	-1.363647	1.923615
12703833	IL1A	281250	NM_174092	-1.598180	**-6.196356**	**3.877132**
12832473	LOC100301071	100301071	XM_002699098	**-2.003163**	-1.547769	-1.294226
12730068	LOC618213	618213	XR_811567	-1.703391	-1.902149	1.116683
12873374			GENSCAN00000028999	-1.391162	**-2.171633**	1.561021
12856116	THSD7A	782187	NM_001206743	1.160044	**2.164989**	-1.866299
12825182	SLC20A2	518905	NM_001080280	**-2.018411**	-1.364469	-1.479264
12702138	DNAJC27	540033	NM_001098109	**-2.032718**	-1.030554	-1.972452
12895413	AAED1	616897	NM_001076436	1.377775	**2.096263**	-1.521485
12896505	LAMA4	529670	NM_001205965	-1.222039	**-2.579712**	**2.110990**
12866832	METTL20	530461	XM_005198364	**-2.527340**	1.070786	**-2.706240**
12721560	MIR2310	100313355	NR_031046	-1.253889	1.094999	-1.373008
12763498	LOC517569	517569	XM_002695737	**2.016719**	1.748927	1.153118
12760480	MIR2348	100313158	NR_031289	1.009688	-1.499644	1.514173
12823815	DLC1	511433	NM_001102493	1.336445	1.395556	-1.044230
12846912	MLLT11	539519	NM_001076169	**-3.027422**	**-2.533866**	-1.194784
12763615	FAM20A	521099	XM_002707768	-1.184584	**2.132033**	**-2.525572**
12859097	METTL1	511197	NM_001046077	1.767083	**2.324741**	-1.315581
12882858	LOC100335642	100335642	XM_002683636	-1.345740	**2.468629**	**-3.322133**
12725851	WISP1	539163	XM_005215320	**-2.444704**	-1.999044	-1.222937

ProbeID	Gene_Symbol	Gene_ID	mRNA Accession	OV24H/OV0H.fc	OV24R/OV0H.fc	OV24H/OV24R.fc
12803716	HIST1H2AM	618824	XM_002697505	1.433429	1.129786	1.268761
12819166	PTPRE	505285	NM_001205531	1.021373	-1.080531	1.103625
12681523	BCHE	534616	NM_001076906	**-2.940945**	-1.302705	**-2.257569**
12898542	RWDD2A	539733	NM_001205704	-1.444530	1.669065	**-2.411014**
12786321	ESM1	539571	NM_001098101	**-5.487085**	**-15.015077**	**2.736440**
12885042	TYK2	512484	NM_001113764	1.003437	1.275108	-1.270741
12745292	GLRB	281198	NM_174071	1.228102	**2.822552**	**-2.298305**
12783551	KHDRBS1	538775	NM_001046442	-1.122869	1.469637	-1.650210
12888848	MIR2466	100313235	NR_031006	**2.330226**	1.856841	1.254941
12737340	SMYD3	616050	NM_001076406	1.335954	**2.067002**	-1.547211
12759917	GYS1	511047 // 7863	NM_001024515	1.245150	-1.132068	1.409594
12818411	MSRB1	618441	NM_001034810	-1.523437	1.075494	-1.638448
12788683				1.388782	-1.089246	1.512725
12728837	LOC531237	531237	XM_002692896	**-2.033747**	-1.337084	-1.521031
12770563	COG1	515788	NM_001192427	1.263005	1.631252	-1.291565
12769400	CCR7	510668	NM_001024930	-1.103854	-1.148826	1.040740
12685403	NLGN1	530042	NM_001205973	**-3.858339**	-1.846598	**-2.089431**
12848091	ADAM15	784605	NM_001205442	1.014016	**2.355380**	**-2.322824**
12906591	MIR2486	100313248	NR_031275	1.031076	-1.163588	1.199747
12727485	LOC510901	510901	XM_005216558	**2.153614**	1.356548	1.587570
12848523			ENSBTAT00000053230	-1.515086	-1.621602	1.070304
12843331	GADD45A	505463	NM_001034247	**2.465380**	**2.951892**	-1.197338
12787721	IL31RA	522636	NM_001192563	1.135549	1.084846	1.046738
12704110	SLC8A1	337925	NM_176632	-1.643327	**-2.072526**	1.261176
12762177	COPS3	507932	NM_001098904	-1.274508	1.486140	-1.894097
12825497	SORBS2	614391	NM_001079787	1.525107	**2.115517**	-1.387127
12857108	TIMP3	282094	NM_174473	1.789182	**2.220508**	-1.241075
12804444	EDN1	281137	NM_181010	**2.089344**	-1.634501	**3.415034**
12826173	LOC510670	510670	NM_001105618	**-2.188952**	**2.464099**	**-5.393792**
12870553	TACC3	506194	NM_001100305	-1.070296	-1.117039	1.043673
12687887	FOS	280795	NM_182786	**2.232732**	1.803088	1.238282
12897508	PDE7B	522509	NM_001102068	1.711202	1.682178	1.017254
12821369	SCD	280924	NM_173959	**-2.918385**	-1.207949	**-2.415982**
12856557	KRT77	100336698	XM_002687245	1.624145	1.449615	1.120397
12678851	NDUFB4	327706	NM_175822	1.148356	1.333160	-1.160929
12870067	PDE5A	281972	NM_174417	**-2.342388**	-1.443939	-1.622221
12685825	ERG	535231	NM_001102183	-1.190411	-1.066105	-1.116598
12732587	ZNF215	529157	NM_001076882	**-2.063452**	-1.795680	-1.149120
12734323			ENSBTAT00000034028	1.153606	**3.521182**	**-3.052328**
12783122	FAM76A	535402	NM_001015660	-1.378300	1.454204	**-2.004330**
12891947	LOC616977	616977	XM_005199128	1.182369	**2.030057**	-1.716940
12678517	MX1	280872	NM_173940	**-2.121655**	-1.319316	-1.608148
12902566	MIR502B	100313383	NR_031326	-1.038406	-1.635351	1.574867
12873740	SDAD1	507675	NM_001102489	1.012492	1.420422	-1.402898
12840882	EXTL2	540527	NM_001076224	**-2.128991**	-1.096351	-1.941888
12857450	KRT6B	404115	NM_001257404	**-2.194268**	-1.984437	-1.105738
12835816	DPYD	281124	NM_174041	1.661608	**2.644033**	-1.591249
12893429	TDH	511957	NM_001046104	-1.043847	-1.186918	1.137061
12754736	CMTM3	787512	NM_001099399	-1.671288	1.392213	**-2.326788**
12883402	ANXA6	327685	NM_001103224	**-2.098197**	-1.661447	-1.262873
12834862	LOC614402	614402	XM_010821155	1.064473	-1.270125	1.352014
12879927	DAPK3	525506	NM_001101124	-1.634444	-1.258173	-1.299062
12908337	DNASE1L1	515176	NM_001038634	1.223301	1.693526	-1.384390
12832208	LOC100336475	100336475	XM_001255100	1.015310	-1.075248	1.091710
12803409	TFAP2B	540867	NM_001076247	-1.407037	-1.324034	-1.062689
12746511	LOC100852110	100852110	XM_005195105	-1.977270	**-2.299611**	1.163023
12767023	CCL2	281043	NM_174006	**-4.833204**	**-3.523062**	-1.371876

ProbeID	Gene_Symbol	Gene_ID	mRNA Accession	OV24H/OV0H.fc	OV24R/OV0H.fc	OV24H/OV24R.fc
12759042	MT1E	613358 // 7683:	ENSBTAT00000002088	-1.432644	-1.502724	1.048917
12870804	KIAA1211	782369	XM_005198628	**-2.094506**	**-2.373424**	1.133167
12803653			ENSBTAT00000015992	-1.827549	1.545335	**-2.824176**
12833745				-1.034784	-1.165541	1.126362
12697060	C10H14orf105	614197	XM_005192421	**-2.207984**	**-2.297938**	1.040740
12689010	DCAF4	511629	NM_001024520	-1.027430	-1.005498	-1.021812
12744061	HIC2	539541	NM_001192270	1.329239	**2.036766**	-1.532280
12868043	MDM2	540378	NM_001099107	**2.055458**	**2.388591**	-1.162072
12755848	SLC7A5	282369	NM_174613	**2.648068**	1.494414	1.771977
12751621	LPCAT2	514646	NM_001205797	-1.944636	-1.367385	-1.422156
12827408	TMEM26	100125281	NM_001103163	**-2.824861**	**-2.824861**	1.000000
12792414	MTHFS	513372	NM_001075616	1.893283	**3.038649**	-1.604963
12729909	PTPMT1	614890	NM_001076353	1.159594	**2.305101**	-1.987852
12812019	NDE1	508088	NM_001100321	**-2.007208**	-1.797921	-1.116405
12726238	LOC782385	782385	XM_002692707	-1.207681	1.774718	**-2.143294**
12904529	TMEM164	524183	NM_001031762	**-2.020679**	-1.621254	-1.246368
12813989	BAIAP2L1	615412	XM_003584061	1.485337	**2.223604**	-1.497037
12822595	LOC532031	532031	XM_002698595	-1.521886	-1.723367	1.132389
12684618	CBS	514525	NM_001102000	1.972588	**2.066458**	-1.047587
12854099	ASNS	514209	NM_001075653	**3.186273**	**2.751465**	1.158028
12824044	SMIM19	515895	NM_001098040	1.046078	1.212992	-1.159562
12733912	LOC785144	785144	NM_001206667	-1.585151	**-2.113406**	1.333252
12864496	NAB2	504755	NM_001045897	-1.188622	-1.353298	1.138544
12706002	MTHFD2	517539	NM_001075755	**2.327966**	1.840809	1.264643
12692926			ENSBTAT00000064523	**-2.125069**	**-2.602954**	1.224880
12802397	CDSN	522351	NM_001077950	**-2.228511**	-1.876286	-1.187724
12712739	TEX30	613895	NM_001076295	**2.819110**	1.961273	1.437388
12901187	BCLAF1	540648	NM_001098117	-1.420147	1.701515	**-2.416401**
12728735	LOC528807	528807	XM_002693269	1.015359	**-2.080456**	**2.112410**
12893297	NMRK1	510456	XM_002689653	-1.270644	1.174641	-1.492551
12869838	CXCL8	280828	NM_173925	**-4.279345**	**-3.464267**	-1.235282
12806992	GSTA3	768055 // 7776	ENSBTAT00000005618	**-2.043313**	**-2.304111**	1.127635
12756919	MGC138914	512219	NM_001083673	-1.529510	-1.194701	-1.280245
12905630	CDK16	613810	NM_001101226	-1.218056	1.236190	-1.505748
12811225				1.817619	-1.553896	**2.824391**
12864595	MFNG	505267	NM_001101051	**-2.094796**	1.021444	**-2.139717**
12825327	LOC533821	533821	XM_005226034	1.290768	1.187362	1.087089
12899272	PTPRK	509657	NM_001191537	1.959601	**2.073244**	-1.057993
12791431	MIR345	791018	NR_031357	1.617393	1.274534	1.269007
12789500	ISG20	506604	XM_002696514	-1.060916	-1.357018	1.279101
12739381	FBXO6	513023	NM_001034433	1.512799	**2.408692**	-1.592209
12806178	MIC1	533051	NM_001127317	1.478352	-1.031140	1.524388
12762208	TMEM106A	508269	NM_001014870	-1.318831	1.494590	-1.971112
12862571	MAFF	617914	NM_001103300	-1.321073	-1.192997	-1.107357
12896514	ESR1	407238	NM_001001443	1.304087	-1.967195	**2.565393**
12738141	LOC101905630	101905630	XM_005200961	-1.720598	-1.291216	-1.332541
12688416	CHAC1	505991	NM_001098882	**3.428957**	**2.179821**	1.573045
12893194	AADAT	508929	NM_001015551	-1.195256	1.835878	**-2.194344**
12836500	CITED4	504742	NM_001080726	-1.097347	1.120451	-1.229524
12819467				-1.369292	**-2.240219**	1.636042
12732109	LOC515027	515027	XM_002693199	1.311329	-1.536524	**2.014888**
12814286	PRR35	101902553	XM_005196865	-1.544286	**-2.029817**	1.314405
12825859	PLAU	281408	NM_174147	**-3.375521**	**-2.267905**	-1.488387
12786624	ENC1	617091	NM_001078067	1.486016	-1.078608	1.602828
12829803	LOC505546	505546	XM_002699202	1.026440	-1.102952	1.132115
12905482	ARAF	540421	NM_001014964	1.035559	1.207631	-1.166163
12849229	HGF	282879	NM_001031751	**-2.167212**	**-2.387615**	1.101699

ProbeID	Gene_Symbol	Gene_ID	mRNA Accession	OV24H/OV0H.fc	OV24R/OV0H.fc	OV24H/OV24R.fc
12791245	LOC617694	617694	XR_813961	-1.643567	1.341010	**-2.204039**
12826638	FAM213A	534049	NM_001034599	**2.082678**	1.525773	1.364999
12843892	CYR61	508941	NM_001034340	1.365747	-1.101615	1.504527
12807392	OSBPL1A	100336844	XM_002697723	-1.842137	1.587746	**-2.924845**
12897798			GENSCAN00000029329	**-2.701742**	**-2.047467**	-1.319554
12769340	C19H17orf85	510317	XM_002695755	-1.718965	1.158092	-1.990720
12787973	CDH6	537946	NM_001034640	**-4.020960**	**-4.604869**	1.145216
12803691	LOC618034	618034	XM_002697413	-1.671137	-1.518124	-1.100791
12761426	MYL4	504201	NM_001075149	-1.294612	1.008485	-1.305597
12813690	PLK1	538238	NM_001038173	1.321201	1.163523	1.135518
12873299	EVC	282874	NM_174747	**-2.161541**	-1.603284	-1.348196
12710489	THSD1	541228	NM_001014967	-1.334565	-1.464786	1.097575
12752602	LOC530319	530319	XR_803009	**-2.517881**	**-3.003615**	1.192914
12863649	MIR2437	100313217	NR_031054	**-4.890019**	**-4.441910**	-1.100882
12815621	ABCA3	505787	NM_001113746	1.142814	**2.694729**	**-2.357978**
12738172			NM_174771	-1.345488	-1.463386	1.087624
12868434	CCND2	615414	NM_001076372	**-2.799094**	-1.555168	-1.799867
12878534	DOT1L	510442	XM_003586273	-1.089359	-1.302010	1.195207
12889824	TTC39B	508428	XM_002689579	**-2.893890**	-1.108731	**-2.610091**
12693777	BMP4	407216	NM_001045877	-1.514803	**-3.010723**	1.987535
12847127	ST6GALNAC5	540995	NM_001192804	**-2.401191**	**-2.480498**	1.033028
12815104	LOC101904761	101904761	XR_240129	-1.458919	-1.428331	-1.021416
12750559			GENSCAN00000014380	-1.042899	1.071179	-1.117132
12823293	MIR107	791005	NR_031010	**2.479433**	**2.384737**	1.039709
12878044	CIRBP	507120	NM_001034278	**-2.268864**	1.021741	**-2.318192**
12852779	PODXL	616366	XM_002686960	1.209634	-1.045280	1.264406
12693130	TCRA	785621	BC146053	**2.015573**	1.140100	1.767892
12860825	KRT73	531981	NM_001111104	**-2.004233**	-1.587702	-1.262348
12884646	LOC509641	509641	XM_002688615	1.161976	-1.813655	**2.107423**
12890767	SLC31A2	526609	NM_001034556	1.177143	1.631038	-1.385590
12889817	ERCC6L2	508357	NM_001082606	-1.821213	1.227251	**-2.235085**
12887236	MAST1	539825	NM_001191528	-1.102180	-1.241918	1.126783
12895371	LURAP1L	616371	NM_001101256	-1.452321	**-2.212182**	1.523205
12797637	TMEM158	788085	NM_001081626	**-2.990756**	**-2.402589**	-1.244805
12719955	GTPBP4	524743	NM_001192601	-1.955530	1.120824	**-2.191806**
12862854	LOC781146	781146	NM_001080336	-1.343932	-1.331202	-1.009562
12908168	MAGED1	512562	NM_001046125	**-2.660800**	-1.320048	**-2.015685**
12683750	TFRC	504698	NM_001206577	**-2.999787**	-1.150260	**-2.607921**
12775831	TFPI	508763	NM_001244204	**-6.022740**	-1.678218	**-3.588771**
12798062	CAMP	317650	NM_174831	-1.297370	**-2.785024**	**2.146669**
12816921	MVP	516456	NM_001035317	-1.246290	**2.234141**	**-2.784387**
12703161	GTF3C5	783869	NM_001191410	1.049149	-1.071796	1.124474
12832085	C29H11orf86	768031	NM_001077090	-1.343438	-1.493958	1.112041
12890045	CDK20	510920	NM_001098933	1.941565	1.720598	1.128425
12703348	ACTR1B	100125305	NM_001103338	-1.341419	1.144137	-1.534767
12829160	LOC100296082	100296082	XR_816485	-1.412245	**-2.041586**	1.445632
12811261				-1.246670	-1.626871	1.304973
12842453	MIR760	100313318	NR_031382	-1.927365	**-2.695084**	1.398325
12877406	EGR1	407125	NM_001045875	-1.576789	**-3.276033**	**2.077661**
12784953				-1.447798	-1.139602	-1.270442
12735710	ERRFI1	516303	NM_001077930	-1.199265	-1.318594	1.099502
12888051	GDF15	618677	NM_001206298	**5.319977**	**3.770537**	1.410934
12756723	C18H19orf54	509803	NM_001164025	1.409741	1.703462	-1.208351
12894099	LOC520638	520638	XM_002689448	-1.173575	-1.562580	1.331470
12693229			ENSBTAT00000050159	1.440540	1.196044	1.204421
12694411	LOC510112	510112	XM_002690711	-1.126838	-1.453449	1.289847
12808491	ZNF521	538792	NM_001105419	1.026896	-1.114850	1.144835

ProbeID	Gene_Symbol	Gene_ID	mRNA Accession	OV24H/OV0H.fc	OV24R/OV0H.fc	OV24H/OV24R.fc
12892466	MIR2474	100313239	NR_030917	1.449897	1.352033	1.072383
12820727	HHEX	539542	NM_001105424	1.373770	1.715953	-1.249084
12826008	TBCE	505066	NM_001038032	-1.701444	1.103801	-1.878056
12790204	CEMIP	519047	XM_002696612	**2.250507**	1.428687	1.575227
12819589	SFXN2	513450	NM_001034446	-1.316429	1.130930	-1.488790
12906399	LOC100851595	100851595	XM_010805270	-1.126729	1.578167	-1.778166
12903893			ENSBTAT00000063298	-1.682038	1.452693	**-2.443485**
12790698	PPP4R4	537521	XM_001790303	-1.118232	-1.002756	-1.115159
12902527	CCNB3	100301478	XM_003584903	**-2.003066**	1.350375	**-2.704890**
12804310	LOC104975716	104975716	XR_815042	-1.190750	**2.010243**	**-2.393696**
12703617			ENSBTAT00000013286	**-2.136886**	**-2.232887**	1.044925
12730328	LOC784741	784741	NM_001206638	**-2.676374**	**-2.081437**	-1.285830
12902169			XM_005201066	**-2.297731**	-1.516137	-1.515517
12746326	ANKRD13A	511883	NM_001205709	-1.913641	1.067133	**-2.042109**
12878509	ATP6AP1L	510390	NM_001207027	-1.436124	**-2.068708**	1.440480
12892664	TSPY	281554	NM_001244608	1.402752	-1.017966	1.427954
12815216	PRM1	281423	NM_174156	-1.357225	-1.249326	-1.086366
12821481	PRKG1	282004	NM_174436	1.044201	**2.173846**	**-2.081827**
12807830	SERPINB10	510205	NM_001098925	**-2.094854**	-1.857317	-1.127893
12816143	HAGH	509274	NM_001035274	1.095303	1.276771	-1.165679
12824540	DEFB7	768320	NM_001102362	-1.461561	-1.941740	1.328538
12730088			GENSCAN00000016365	-1.227897	-1.361465	1.108778
12898821	LOC614138	614138	XR_802042	-1.343001	-1.785292	1.329331
12867247	ACVRL1	534536	NM_001083479	-1.134833	-1.663613	1.465954
12871728	LOC534181	534181	XM_005198640	-1.760079	**-2.013339**	1.143891
12804010	H2B	519934 // 7875	NM_001105643	-1.251849	**-2.212366**	1.767279
12736341	LOC525101	525101	XM_002694075	-1.032391	-1.011587	-1.020566
12848526	LOC100295614	100295614	XR_806392	-1.183057	-1.359051	1.148762
12852910	CD36	281052	NM_001278621	-1.871545	-1.451797	-1.289123
12685337	ANKRD28	529062	NM_001205960	1.059784	1.413694	-1.333946
12788050	NLN	538650	NM_001033989	**-2.255379**	1.040279	**-2.346223**
12878231	DDX46	508660	XM_005199046	-1.263250	**2.004122**	**-2.531706**
12706587	MAPRE3	528839	NM_001075917	1.204997	1.217769	-1.010599
12721596	HAS2	281220	NM_174079	**-2.396851**	-1.761202	-1.360918
12731091	LOC504200	504200	XM_002693829	1.128096	-1.150356	1.297712
12817732	PSPH	533630	NM_001046355	**3.030887**	**3.552316**	-1.172039
12688328	ATP5S	493709	NM_001007812	1.330935	1.823083	-1.369776
12906561	LOC100300684	100300684	XM_002699503	1.028271	-1.105616	1.136872
12882714	CTH	539159	NM_001024567	**3.133554**	**2.663845**	1.176328
12801726	FBXO9	511798	NM_001034412	**-2.528129**	**-2.055202**	-1.230112
12843293	THRAP3	505228	NM_001205565	-1.289516	1.380136	-1.779707
12884126	OR1I1	507573	XM_002688604	1.449254	1.139894	1.271393
12850461	LHFPL3	520147	NM_001104984	1.073737	1.110801	-1.034519
12833840	FADS3	515925	NM_001083691	-1.014030	-1.070830	1.056015
12891041	PSAT1	533044	NM_001102150	**2.711178**	**2.337976**	1.159626
12772434	ENO3	540303	NM_001034702	-1.705423	1.399984	**-2.387565**
12868116	C3AR1	540702	NM_001083752	-1.675335	-1.435188	-1.167328
12710343	HS6ST3	538566	NM_001205484	**-2.475552**	**-2.168173**	-1.141768
12893731	IFT74	514024	NM_001205775	**-2.156348**	1.573743	**-3.393538**
12864606	USP5	505280	NM_001192056	-1.436104	1.015120	-1.457817
12811263	FUS	280796	NM_173912	-1.019668	1.594098	-1.625451
12781280	LDLRAP1	511199	NM_001083668	1.002429	1.249560	-1.246532
12790928	LRFN5	539366	NM_001081610	1.172542	-1.167741	1.369225
12821842	SFXN4	508473	NM_001035060	-1.026078	1.389976	-1.426223
12712708	LCP1	540990	NM_001034720	**-2.498600**	-1.905580	-1.311202
12878696	OR2V1	511586	XM_002689049	-1.843542	**-2.064654**	1.119939
12692273	LOC781758	781758	XM_002690701	-1.395682	-1.800179	1.289820

ProbeID	Gene_Symbol	Gene_ID	mRNA Accession	OV24H/OV0H.fc	OV24R/OV0H.fc	OV24H/OV24R.fc
12698604			ENSBTAT00000025313	-1.896396	**-2.518125**	1.327848
12865769	NCKAP1L	513641	NM_001143876	-1.074906	1.056959	-1.136132
12719918	FERMT1	524427	NM_001206179	-1.093028	-1.202644	1.100287
12809470	ATP9B	510301	NM_001080255	-1.140432	1.419468	-1.618806
12786260	PLK2	539449	NM_001192245	-1.140463	-1.395730	1.223827
12713287	WFDC10A	100296236	XM_005192497	-1.497172	-1.645835	1.099296
12835715	F3	280686	NM_173878	1.153749	-1.814736	**2.093751**
12901838			NM_001034793	-1.629749	-1.084831	-1.502307
12761717	MSI2	505542	NM_001206656	-1.224820	1.434880	-1.757470
12800454	MAPKAPK3	615215	NM_001034779	-1.668267	1.192294	-1.989064
12896174			GENSCAN00000000783	**-2.430512**	**-2.013813**	-1.206920
12800430	NICN1	614730	NM_001034767	1.067081	1.523363	-1.427598
12866349	INHBE	517587	NM_001205842	**13.596374**	**10.959272**	1.240627
12880562	MAPK9	534125	NM_001046369	-1.449284	1.029297	-1.491744
12903971	LOC513925	513925	XR_803719	-1.179790	-1.270028	1.076486
12859705	LOC515697	515697	NM_001102030	-1.903362	**-2.021043**	1.061828
12682595	B3GALNT1	767844	NM_001076963	**-2.739875**	-1.213354	**-2.258101**
12866101	DUSP6	515310	NM_001046195	**-2.067676**	**-4.314550**	**2.086666**
12681726	BTD	537669	NM_001102206	1.189619	**2.072799**	-1.742405
12760464	MIR744	100313080	NR_030988	1.514509	1.227982	1.233331
12817714	IGFALS	532494	NM_001075963	1.006028	1.575085	-1.565648
12822177	FBXW4	513977	NM_001101985	1.396998	1.452401	-1.039659
12888738	TMEFF1	100139803	NM_001205476	1.114047	1.266459	-1.136809
12806778	HIST1H2BI	616776	XM_002697461	-1.014438	-1.158469	1.141982
12872256	DKK2	541161	NM_001082615	1.921017	**-3.322847**	**6.383245**
12821871	ANKRD1	510376	NM_001034378	1.232417	1.174201	1.049578
12903830	KIAA2022	512493	XM_002699975	1.103671	1.131048	-1.024805
12744947	HRK	787139	NM_001191241	**-4.173796**	**-7.228719**	1.731929
12778525	SLC25A12	539494	NM_001101194	-1.931110	-1.172591	-1.646874
12850012	NT5C3A	511858	NM_001037597	1.913389	**2.795681**	-1.461115
12881383	SH3RF2	540718	NM_001083753	1.059409	1.149359	-1.084906
12679507	LOC508623	508623	XR_804310	-1.617774	-1.672829	1.034031
12688601	OR10G2	507759	XM_002690730	-1.148436	-1.147974	-1.000402
12858096	ISX	506388	XM_010805321	1.034748	-1.281079	1.325595
12794639	MGLL	505290	NM_001206681	-1.312748	1.165695	-1.530263
12777117	LOC524236	524236	XM_005197411	-1.135148	-1.650943	1.454386
12855153	ICA1	535346	NM_001038168	**-2.297062**	1.410601	**-3.240238**
12901412	FAM26E	101903708	XM_005199378	-1.410669	-1.078847	-1.307572
12682999	LOC100300014	100300014	XM_010801720	1.387416	1.313768	1.056059
12830844	CARS	515715	NM_001103249	1.716583	**2.021856**	-1.177837
12812007	LOC790390	790390	XM_010800284	1.182516	1.324530	-1.120094
12717216	BPIFB2	618574	NM_001193194	-1.142172	-1.530603	1.340081
12736694	TGFB2	534069	NM_001113252	-1.048488	1.255463	-1.316338
12836423	DIRAS3	504559	NM_001034215	-1.117682	-1.093354	-1.022251
12782572	LOC101908056	101908056	XM_010802213	1.371467	1.638414	-1.194643
12726287			GENSCAN00000020389	1.656628	1.498491	1.105531
12683570	MYLK	338037	NM_176636	-1.199630	-1.386272	1.155582
12809196	SERPINB2	505184 // 28137	NM_001192079	**2.487385**	**2.625077**	-1.055356
12901337	MARCKS	613548	NM_001076276	-1.084387	1.501246	-1.627932
12788836	MIR431	100313313	NR_031063	-1.332061	-1.418897	1.065189
12716781	BMP7	540595	NM_001206015	-1.361380	-1.556235	1.143130
12897460	PHACTR2	522158	XM_002690297	1.092172	1.478588	-1.353805
12810552	ATP8B1	541187	NM_001192839	-1.249759	-1.264687	1.011944
12724971	CYC1	512500	NM_001038090	-1.008324	1.276992	-1.287623
12700001	SEPT10	514603	NM_001046176	**-2.245614**	-1.131056	-1.985414
12755369	FAM83E	100851343	XM_003584863	-1.040885	1.163418	-1.210984
12879301	LOC101904981	101904981	XM_010798262	-1.245418	-1.440291	1.156472

ProbeID	Gene_Symbol	Gene_ID	mRNA Accession	OV24H/OV0H.fc	OV24R/OV0H.fc	OV24H/OV24R.fc
12813389	MAPK3	531391	NM_001110018	-1.887452	1.227693	**-2.317212**
12769620	SPATA20	511363	NM_001083407	-1.501766	1.156608	-1.736955
12872721				-1.617617	-1.528769	-1.058118
12869377	LOC787600	787600	XM_001254950	-1.598524	-1.574703	-1.015127
12902629	LOC517820	517820	XM_010821425	1.139483	1.054588	1.080501
12788826	MIR2284F	100313169	NR_030981	-1.023740	-1.495067	1.460396
12708824	MIR2300B	100313278	NR_031038	1.196856	-1.112180	1.331119
12738095	LOC100299783	100299783	XM_002694090	1.962088	1.883949	1.041476
12884966			GENSCAN00000034208	1.148452	1.012267	1.134534
12819502	SFXN3	511755	NM_001101946	-1.880401	-1.077912	-1.744484
12835548	GBP2	514143	XM_010803354	-1.875493	-1.415096	-1.325347
12772679	GAS7	614517	NM_001102280	1.035581	-1.239536	1.283639
12802627	LOC528373	528373	XM_002697495	-1.093103	-1.412245	1.291959
12902249	TCEAL7	100138330 // 1(	XM_001790056	1.395933	1.029305	1.356191
12749115	LOC617141	617141	XM_010815466	-1.292129	-1.707564	1.321512
12807288				1.042184	1.101600	-1.057011
12719139	CASS4	512832	XM_005194419	1.345367	-1.042256	1.402217
12719676	SLC17A9	518062	NM_001100378	-1.014501	1.521569	-1.543633
12845601			ENSBTAT00000065314	-1.089307	1.395208	-1.519809
12870781	DDIT4L	510906	NM_001081519	-1.780781	-1.993081	1.119217
12703609	MIR2296	100313121	NR_031090	1.138804	-1.428004	1.626217
12731528	LOC508808	508808	XM_002693215	-1.979848	-1.513564	-1.308070
12688814	IPO4	510372	NM_001083661	-1.016098	1.152606	-1.171161
12786958	ITGA2	281872	NM_001166499	-1.466899	-1.434940	-1.022273
12910823	MIR92A-2	791023	NR_031359	-1.337186	-1.479900	1.106727
12784009	DAPL1	574085	NM_001025346	-1.265818	-1.040473	-1.216579
12687068	LOC618840	618840	XR_804063	**-2.150243**	**-2.200772**	1.023499
12803265	LOC616819	616819	XM_010818465	1.939077	1.267117	1.530306
12851861	GSAP	615147	NM_001193061	-1.191897	-1.027708	-1.159763
12875191	SCFD2	535130	NM_001078005	1.537962	1.328097	1.158020
12713997	LOC407163	407163	XM_005200225	**-2.085842**	-1.763107	-1.183049
12771970	KRT35	536311	NM_001076073	-1.425018	1.299764	-1.852187
12750214	LOC506634	506634	XM_003587272	1.487727	1.296282	1.147688
12754716	BOSTAUV1R414	787174	XM_010815328	-1.043963	-1.251094	1.198409
12889336	GRHPR	504764	NM_001192037	-1.337204	-1.006586	-1.328456
12800654	FAM19A1	782935	NM_001099727	-1.483505	-1.449485	-1.023471
12796196	WNT5A	530005	NM_001205971	-1.255603	-1.328631	1.058162
12732069	LOC513948	513948	XM_002693766	1.066172	-1.162250	1.239158
12696603	GREM1	539079	NM_001082450	**-2.953345**	**-2.721836**	-1.085056
12772448	FAM64A	540455	NM_001099109	-1.321485	-1.363373	1.031697
12896979	MICAL1	508306	NM_001081582	-1.932810	-1.109685	-1.741765
12806768	HIST1H2AH	616634	XM_010818503	-1.216259	-1.599843	1.315380
12902242	LOC101906965	101906965	XM_010821798	1.035100	1.042126	-1.006788
12717610	CENPB	788381	XM_001255460	-1.254950	1.176344	-1.476253
12753328	FOXL1	538955	XM_005192656	-1.314861	-1.172648	-1.121275
12845168	SLC50A1	520463	XM_003581932	1.393082	1.678148	-1.204630
12828476	TSNAX	533927	NM_001076006	**-2.475706**	-1.288185	-1.921856
12773167	RAC3	619066	ENSBTAT00000031159	-1.225355	-1.179087	-1.039241
12846849	LOC786781	786781	XM_001254364	**2.405555**	**2.273729**	1.057978
12723720	LOC782052	782052	XR_811321	1.067533	-1.121539	1.197280
12874623	HNRNPD	527471	NM_001098064	**-2.370761**	-1.367234	-1.733983
12806766	LOC616611	616611	XR_814900	-1.487717	-1.678439	1.128198
12781514	SFT2D3	514114	XM_005197407	-1.343494	-1.329589	-1.010458
12793839	BRF1	618161	XM_005196352	-1.011776	-1.123204	1.110131
12735269	ELF3	508503	NM_001098909	1.509321	1.702530	-1.128010
12863077	PRICKLE1	785152	NM_001102534	-1.590257	-1.528271	-1.040560
12791021	MAN2C1	541086	NM_001110085	1.083756	-1.140700	1.236241

ProbeID	Gene_Symbol	Gene_ID	mRNA Accession	OV24H/OV0H.fc	OV24R/OV0H.fc	OV24H/OV24R.fc
12703346	MIR181B-2	791012	NR_031367	**-4.061098**	**-3.846403**	-1.055817
12875311	MAPK10	537631	NM_001083728	-1.034031	-1.070942	1.035696
12886108	CHSY3	528149	NM_001192652	-1.668695	-1.340359	-1.244961
12798047	SLC6A6	282366	NM_174610	**-2.530390**	-1.410220	-1.794323
12715724	LOC524176	524176	XM_005192491	1.072078	-1.239089	1.328400
12736336	LOC525100	525100	XM_002694074	-1.357997	-1.249326	-1.086983
12869667	SLC39A8	508193	NM_001205630	-1.476253	-1.460872	-1.010528
12848566	LOC100299757	100299757	XM_005205556	-1.569690	1.129880	-1.773562
12693052			GENSCAN00000040356	1.541847	1.389408	1.109716
12876554	C7H5orf63	100335582	XM_002688977	-1.059945	1.388791	-1.472043
12719715	LOC519069	519069	XM_002692063	1.061416	-1.392580	1.478106
12873877			GENSCAN00000015031	1.009136	**2.035213**	**-2.016789**
12831879	SLC36A4	616864	NM_001282523	**-3.659915**	-1.421585	**-2.574532**
12682761	KRTAP10-8	783679	NM_001101290	-1.558599	-1.227557	-1.269676
12732213	SLC43A3	516840	NM_001038126	-1.790708	-1.198832	-1.493710
12730326	LOC784603	784603	XM_005194618	-1.440780	-1.504016	1.043890
12807081	LOC785277	785277	XM_001253348	-1.377574	-1.175284	-1.172120
12865056	SLC35E3	509009	NM_001083654	-1.908911	-1.166762	-1.636076
12841606	NEGR1	781106	NM_001205414	**-2.778738**	-1.467530	-1.893480
12734210			GENSCAN00000001426	-1.193849	-1.015253	-1.175912
12765029	PYCR1	539606	NM_001014957	**2.236465**	**2.369627**	-1.059541
12802519	HIST1H2BB	525512 // 6149!	XM_002697525	1.085508	-1.948306	**2.114901**
12753547	LONP2	541085	NM_001034723	-1.185808	1.174763	-1.393043
12811184	MIR940	100313089	NR_031219	-1.243615	-1.900383	1.528112
12683071	LSAMP	100336456	NM_001205368	-1.717667	-1.381035	-1.243753
12692745	GPR65	788507	NM_001101303	-1.440191	-1.463822	1.016408
12687982	GLCE	281195	NM_174070	**-3.225024**	-1.985841	-1.624009
12757460	DDX19B	517438	NM_001046230	-1.261133	1.057282	-1.333373
12745282	LIF	280840	NM_173931	1.039983	-1.650153	1.716131
12726440	LOC100300167	100300167	XM_010812313	-1.023201	1.002922	-1.026192
12686720	TBC1D5	614554	NM_001205489	1.194635	1.727888	-1.446373
12720693	GDF5	539559	NM_001192273	-1.066586	-1.354885	1.270301
12689502	PTGDR	515331	NM_001098034	-1.082960	**-2.497405**	**2.306092**
12852736	MIR320B	100313207	NR_030877	**2.840156**	**2.776313**	1.022996
12801577	HIST1H1D	509275	NM_001101066	1.347215	1.266239	1.063950
12809644	SERPINB8	513825	NM_001035287	-1.658950	-1.108232	-1.496933
12786562	LOC614206	614206	XM_005196157	-1.209390	**-2.011275**	1.663048
12817585	MYH11	530050	NM_001102127	-1.445141	-1.585337	1.097013
12869024				-1.084274	-1.194453	1.101615
12726408	FJX1	100297910	XM_003584883	-1.299134	-1.196259	-1.085997
12893945	LOC100335490	100335490	XM_002704930	**-3.042864**	**-2.806049**	-1.084395
12901874			XM_010800841	1.020842	1.404688	-1.376009
12709498	VPS36	511223	NM_001098939	**-2.115619**	-1.125027	-1.880505
12765398	PRR11	615857	NM_001098137	-1.609798	-1.371543	-1.173713
12730037	IL18BP	617470	XM_002693432	1.877171	1.043970	1.798108
12756039	MT1A	404071	NM_001040492	-1.028064	1.680791	-1.727960
12844465	MFSD2A	512633	NM_001101959	-1.770111	-1.909242	1.078600
12884704	LOC510252	510252	XR_801371	-1.596674	-1.245263	-1.282199
12809133	BCL2	281020	NM_001166486	1.089322	1.080441	1.008220
12694003	EID1	506056	NM_001098377	-1.783808	-1.012400	-1.761959
12853855	GIMAP7	100125415 // 6:	NM_001080257	-1.166713	-1.544843	1.324098
12775434	ATIC	506343	NM_001075254	-1.263215	1.267917	-1.601651
12687143			ENSBTAT00000015298	-1.533056	-1.850917	1.207338
12892548	TRPM3	100337068 // 5ʌ	XM_010807877	-1.172022	-1.513113	1.291028
12756033	MT2A	404070	NM_001075140	-1.455757	1.278977	-1.861880
12878157	PTTG1	100848911 // 5(	XM_003582470	1.449565	1.020043	1.421082
12780762	DNPEP	506882	NM_001045952	-1.144788	1.352154	-1.547930

ProbeID	Gene_Symbol	Gene_ID	mRNA Accession	OV24H/OV0H.fc	OV24R/OV0H.fc	OV24H/OV24R.fc
12778346	CCNYL1	538167	XM_002685539	-1.284574	-1.245349	-1.031497
12693599	PCK2	282856	NM_001205594	**3.666821**	**3.876111**	-1.057077
12738957	SCCPDH	507289	NM_001034288	-1.078510	**2.361052**	**-2.546419**
12730578	MIR2284D	100313461	NR_031104	1.373884	-1.736449	**2.385679**
12713419	MIR296	100313259	NR_031141	1.210883	1.000000	1.210883
12870641	SLC39A8	508193	NM_001205630	-1.261307	-1.358270	1.076874
12718209	TRDMT1	353353	NM_181812	**-2.071161**	-1.635577	-1.266318
12678443	LOC100337296	100337296	ENSBTAT00000065610	1.095121	1.182401	-1.079700
12721303	DEFB122A	785417	NM_001102339	-1.537749	-1.578856	1.026732
12825663	LOC789175	789175	NM_001114862	-1.777587	**-2.133689**	1.200329
12734691	PFKFB2	287019	NM_174812	-1.183418	1.914968	**-2.266208**
12822288	ACTA2	515610	NM_001034502	**-4.685458**	**-3.187820**	-1.469800
12748263				**-2.192535**	**-2.490042**	1.135691
12807161				**-4.780959**	**-2.477457**	-1.929785
12724247	FABP4	281759	NM_174314	1.052142	-1.047042	1.101638
12770990	HS3ST3A1	521959	NM_001192548	**-2.068005**	-1.410777	-1.465863
12722803	TNFRSF11B	523822	NM_001098056	**-5.946662**	**-11.639603**	1.957334
12692919			ENSBTAT00000065378	1.006272	-1.225585	1.233271
12803578			ENSBTAT00000033365	1.160181	-1.285180	1.491041
12763571	CACNG4	519331	XM_002696227	-1.003987	-1.116660	1.112226
12753770	SELV	615288	NM_001163244	1.016796	1.375685	-1.352961
12872534	CLRN2	617290	NM_001193151	-1.485512	-1.475087	-1.007067
12876045	NMU	782109	NM_001191153	1.004119	1.056644	-1.052310
12805927	HIST1H2AC	506900 // 5248(	XM_005196641	-1.304937	-1.524863	1.168534
12770208	ALDH3A2	513967	NM_001101984	-1.266415	1.207280	-1.528917
12910560	LOC784970	784970	XR_803515	1.554133	1.475097	1.053580
12820507	CYP2C18	535243	NM_001076051	1.543505	1.135620	1.359174
12897025	HDDC2	509282	NM_001075401	**-2.003038**	-1.300359	-1.540373
12733686	C15H11orf70	617430	NM_001046598	-1.388936	**2.050165**	**-2.847548**
12807157			GENSCAN00000024173	1.581891	**2.160073**	-1.365501
12726701	MMP1	281308	NM_174112	1.127658	1.080179	1.043955
12886016	MFSD12	526668	XM_605041	-1.609196	-1.127377	-1.427381
12697717	LOC785623	785623	XM_001253583	-1.349860	-1.063154	-1.269676
12829117	LOC100139585	100139585	XM_002699010	1.234083	1.195198	1.032535
12711036	FAM155A	616912	NM_001083776	-1.308433	-1.038125	-1.260381
12752079	PRMT1	520388	NM_001015624	1.169888	1.585008	-1.354838
12824843	ANGPT2	282141	NM_001098855	-1.234255	-1.375713	1.114611
12713105	LOC784887	784887	XM_001253067	**-2.004900**	**-2.193219**	1.093930
12842694	PSMD4	282016	NM_001013598	-1.264354	1.430967	-1.809248
12882777			GENSCAN00000031768	-1.043420	-1.100340	1.054552
12901355	GRIK2	615226	NM_001193063	-1.570496	-1.579546	1.005763
12848966	INHBA	281867	NM_174363	-1.745742	-1.286838	-1.356614
12793912	LOC101903487	101903487	XM_010816914	1.439692	-1.204697	1.734392
12700766	IL36A	523429	XM_003582787	-1.219585	-1.129442	-1.079812
12848624			GENSCAN00000029973	1.244038	-1.030025	1.281390
12728368	LOC521645	521645	XM_002693606	-1.136061	-1.200945	1.057113
12783344	UBXN4	536181	NM_001035414	-1.503078	1.293464	-1.944178
12804914	LOC505183	505183	XM_002697515	-1.065248	-1.620299	1.521053
12842602	S1PR1	281135	NM_001013585	-1.082277	-1.291905	1.193691
12903604	RBM3	509771	NM_001303463	**-7.201962**	-1.076240	**-6.691780**
12777867	KCNH7	534542	XM_005202503	1.415969	1.703391	-1.202986
12726322	LOC101905705	101905705	XM_005216146	-1.054486	-1.125604	1.067444
12863669	LOC101905711	101905711	XM_010805440	1.751620	**2.239288**	-1.278409
12689969	MYEF2	524920	NM_001102085	-1.958325	1.347794	**-2.639418**
12697362	CLN6	617615	NM_001109984	-1.189924	1.747194	**-2.079029**
12787048	FST	327681	NM_175801	1.154934	**-2.071721**	**2.392701**
12824802	DEFB1	281743	ENSBTAT00000009756	-1.818980	-1.853626	1.019047

ProbeID	Gene_Symbol	Gene_ID	mRNA Accession	OV24H/OV0H.fc	OV24R/OV0H.fc	OV24H/OV24R.fc
12821454	NT5C2	281951	NM_174405	**-2.833372**	-1.481327	-1.912726
12831187	LOC525938	525938	XM_002699398	-1.247786	-1.228297	-1.015866
12692472			GENSCAN00000022855	1.134833	-1.937478	**2.198714**
12794223	MIR138-1	100313000	NR_030863	-1.186778	**-2.278272**	1.919712
12723468	C14H8orf37	614744	XM_002692813	1.053230	**2.965736**	**-2.815849**
12803695	LOC104975684	104975684	XM_010818515	-1.347495	-1.891984	1.404075
12899445	CTGF	281103	ENSBTAT00000008357	1.124965	1.004836	1.119551
12806854	HIST1H1E	515957 // 6178	XM_002697518	1.259647	1.092823	1.152654
12892357			GENSCAN00000021974	-1.494715	-1.275754	-1.171632
12879015	LOC514002	514002	XR_801241	-1.080650	1.156199	-1.249447
12788824	MIR2366	100313168	NR_030949	**2.270468**	**3.825902**	-1.685072
12732697	SLC35F2	533008	XM_002692963	**-2.223712**	1.309086	**-2.911031**
12718026	THBD	281529	NM_001166522	1.436572	1.144986	1.254663
12765414	GDPD1	615890	NM_001076400	-1.016775	1.819106	-1.849621
12850085	SEMA3C	512660	NM_001101082	-1.833970	1.040459	-1.908170
12786382	LOC787920	787920	XM_001255135	-1.104099	1.037585	-1.145597
12825711	LOC100298658	100298658	XM_002698957	-1.126237	-1.361031	1.208477
12862577	H1F0	617975	NM_001076487	1.940018	**3.779774**	-1.948319
12873900	HERC3	510924	NM_001083663	**-2.588955**	1.314514	**-3.403219**
12814450	GBAS	767938	NM_001077011	-1.603362	1.034906	-1.659329
12794248	ZBTB47	100335506	XM_002702725	**2.151183**	**2.123200**	1.013180
12743103	SLC7A11	524078	XM_010813777	**3.718601**	**3.777783**	-1.015915
12893440	LOC101903205	101903205	XR_801688	**-2.825077**	**-4.823733**	1.707470
12802001	HIST1H1C	513971	ENSBTAT00000015499	1.216014	1.388897	-1.142172
12848557			ENSBTAT00000065831	**-11.628153**	**-4.941092**	**-2.353357**
12712983	FAM155A	616912	NM_001083776	-1.343587	1.194643	-1.605108
12848835	IL6	280826 // 5170	NM_173923	**-3.900692**	**-10.533186**	**2.700338**
12792419	SLC25A21	513423	NM_001015587	1.355956	1.424504	-1.050554
12732324			ENSBTAT00000065837	-1.008940	-1.312593	1.300963
12755458	MIR2330	100313147	NR_030854	**12.694485**	**11.388217**	1.114703
