# Supplementary material for: Development and characterization of Hanwoo oviduct epithelial cell line for heat stress study
Source: Anim Biosci. 2026 Sep 1;39(11):250962. doi: 10.5713/ab.250962 (PMC13740644; doi:10.5713/ab.250962)
Supplement: Supplementary file 13 [file ab-250962-Supplementary-5.pdf]

Supplement 5. Proteomics normalized log2 of immortalized oviduct epithelial cells under heat stress.

Accession	Gene_Symbol	Gene_ID	Ensembl_Gene ID	log2(ratio(OV24H/OV0H))	log2(ratio(OV24R/OV0H))	log2(ratio(OV24H/OV24R))
A0AAF6Z0U5	EIF4A2	A0AAF6Z0U5	ENSBTAG00000014724	0.813763027	0.745928992	0.067834035
F1MWU9	HSPA6	F1MWU9	ENSBTAG00000039035	0.987876441	0.782127776	0.205748665
F1MMU4	H1-10	F1MMU4	ENSBTAG00000040116	0.63487493	0.590594369	0.044280562
A0AAF7AH20	CRYAB	A0AAF7AH20	ENSBTAG00000000434	1.537965021	1.174401949	0.363563073
Q3ZCF3	SKP1	bta:615427; Q3ZCF3	ENSBTAG00000025191	1.248318188	0.675285284	0.573032903
A0AAA9SSY0	CRELD1	A0AAA9SSY0	ENSBTAG00000010835	0.629920283	0.601219971	0.028700312
P61420	ATP6V0D1	bta:282148; P12953; P61420; Q02547; Q3T0P9		0.749474006	0.647094564	0.102379442
A0AAA9TVG8	DNAJB2	A0AAA9TVG8	ENSBTAG00000013782	0.656458092	0.653051491	0.003406601
Q0VCY8	PEA15	bta:510586; Q0VCY8	ENSBTAG00000005793	1.179536942	0.695559332	0.483977611
Q5EAC9	HSPB8	bta:539524; Q3T0Q5; Q5EAC9	ENSBTAG00000001303	1.37137728	0.893529523	0.477847757
A0AAF6YRV5	SLC1A4	A0AAF6YRV5	ENSBTAG00000007763	0.652746549	0.797582854	-0.144836305
F1N7I6	GCA	bta:507139; F1N7I6	ENSBTAG00000018446	0.87803353	0.74772108	0.13031245
A0A3Q1LVU6	SLC12A6	A0A3Q1LVU6	ENSBTAG00000016236	1.145283496	1.097209756	0.04807374
A0AAA9TWM3	S100A11	A0AAA9TWM3	ENSBTAG00000015145	0.593653461	0.750846111	-0.15719265
F1MFW9	KRT24	bta:788424; F1MFW9	ENSBTAG00000040279	0.656045599	2.303596272	-1.647550673
A0AAF6Z117	S100A10	A0AAF6Z117	ENSBTAG00000015147	0.610558817	0.717571973	-0.107013157
Q32KN2	BNIP3	bta:615342; Q32KN2		0.653901738	0.727437982	-0.073536244
A0AAA9TCX1	GPN3	A0AAA9TCX1	ENSBTAG00000005347	0.71122278	0.872591831	-0.161369051
A0AAA9TEE5	DENND1A	A0AAA9TEE5	ENSBTAG00000003610	0.906314554	0.899384061	0.006930494
F1MSS9	BAG4	bta:516353; F1MSS9	ENSBTAG00000013143	0.88658702	0.882559018	0.004028002
Q5E9C3	SSNA1	bta:507840; Q2NL12; Q5E9C3	ENSBTAG00000046415	0.640824862	0.646363045	-0.005538184
Q17QH5	BID	bta:510373; Q17QH5		1.550280661	1.229229851	0.32105081
E1BEQ6	C8H9orf40	bta:533577; E1BEQ6	ENSBTAG00000013231	0.587692293	0.914521037	-0.326828743
F6QNS8	ZC3H8	F6QNS8	ENSBTAG00000015965	0.843102855	0.626922263	0.216180592
F1MZ30	COL10A1	F1MZ30	ENSBTAG00000003078	1.20579694	1.38169324	-0.1758963
A0A3Q1MQ12	SDC2	A0A3Q1MQ12	ENSBTAG00000014357	0.64143527	0.870143193	-0.228707923
F1N1Z3	CRTC3	F1N1Z3	ENSBTAG00000010915	0.829483609	0.737438531	0.092045077
F1MX90	RUNDC1	bta:539525; F1MX90	ENSBTAG00000015140	0.95661729	0.877056912	0.079560378
A0AAA9SJM1	ZBTB17	A0AAA9SJM1	ENSBTAG00000018730	0.701510818	0.705177135	-0.003666317
F1MAV6	RFWD3	bta:540072; F1MAV6	ENSBTAG00000004460	0.699971387	0.586990182	0.112981205
A0AAA9TP99	MACF1	A0AAA9TP99	ENSBTAG00000021024	-0.838937567	-0.768636206	-0.070301361
P02453	COL1A1	bta:282187; P02453; Q3MHM2	ENSBTAG00000013103	-0.882798789	-0.839797636	-0.043001153
F1N401	COL12A1	F1N401	ENSBTAG00000020056	-0.939558781	-0.774770713	-0.164788068
A0AAA9RWG3	TPM2	A0AAA9RWG3	ENSBTAG00000011424	-1.066373022	-1.036792031	-0.029580991
Q08E14	COL3A1	bta:510833; F1MX58; Q08E14	ENSBTAG00000021466	-0.673549763	-0.688500105	0.01495034
G3MZI7	COL5A1	G3MZI7	ENSBTAG00000047998	-0.778741327	-0.838283273	0.059541946
A0AAA9T749	UHRF1	A0AAA9T749	ENSBTAG00000002224	-0.984158484	-0.877886478	-0.106272005
P48734	CDK1	bta:281061; P48734; Q2YDM6	ENSBTAG00000010109	-0.673159458	-0.601836319	-0.071323139
A0AAA9TZT4	RRM2	A0AAA9TZT4	ENSBTAG00000008216	-0.78576806	-1.068940111	0.283172051
F1MFF9	CHD5	F1MFF9	ENSBTAG00000040477	-0.78393646	-0.739647184	-0.044289276
A0A3Q1LVL2	HMOX1	A0A3Q1LVL2	ENSBTAG00000015582	-0.869792631	-1.006829338	0.137036708
A0A3Q1LP21	TBCE	A0A3Q1LP21	ENSBTAG00000013735	-0.762997449	-0.64106239	-0.121935059
A0AAA9TC35	MVD	A0AAA9TC35	ENSBTAG00000012059	-0.709185455	-0.623107515	-0.086077939
A0AAA9SJQ0	RPS6KA4	A0AAA9SJQ0; bta:523746	ENSBTAG00000039153	-0.740494406	-0.742458593	0.001964187
E1BAD4	RUNX1	bta:529631; E1BAD4	ENSBTAG00000004742	-0.890170433	-0.740172475	-0.149997958
A6H7B7	SFRS5	A6H7B7; bta:510474		-1.260139503	-0.753271327	-0.506868176
Q2HJI6	GRN	Q2HJI6		-0.608715014	-0.71762121	0.108906196
A0A3Q1MQB3	LIN54	A0A3Q1MQB3	ENSBTAG00000010546	-0.765203638	-0.814825025	0.049621387
A0A452DII7	MXRA8	A0A452DII7	ENSBTAG00000012247	-0.674514086	-0.651016904	-0.023497181
A0A3Q1M7L9	ATF7IP	A0A3Q1M7L9	ENSBTAG00000003221	-0.649738328	-0.600885843	-0.048852486
A0AAA9S4A3	MXRA5	A0AAA9S4A3	ENSBTAG00000022150	-0.953913087	-1.018242962	0.064329876
A1L552	INTS5	A1L552; bta:514150	ENSBTAG00000010455	-0.643180648	-0.597025239	-0.046155409
A5PJW3	TCF25	A5PJW3; bta:100848970		-0.871158918	-0.827377225	-0.043781692
E1BIU4	ZC3H7B	E1BIU4	ENSBTAG00000002782	-0.791324812	-0.905308299	0.113983486
A6H7A8	DNTTIP1	A6H7A8; bta:505524		-0.606085792	-0.729793939	0.123708146
E1BCZ0	POLRMT	bta:504757; E1BCZ0	ENSBTAG00000015051	-0.996948829	-1.087081092	0.090132263
Q5EA01	B4GAT1	bta:618055; Q3MHK2; Q58DG2; Q5EA01	ENSBTAG00000022238	-0.608971596	-0.734802633	0.125831037
A0A3Q1MUA3	COL15A1	A0A3Q1MUA3	ENSBTAG00000010082	-0.87924132	-0.936219261	0.056977941
A0A3Q1MFU3	TRAF5	A0A3Q1MFU3	ENSBTAG00000012020	-0.609081065	-0.722900964	0.1138199
A0A3Q1M5N9	OLFML2A	A0A3Q1M5N9; bta:516730	ENSBTAG00000004848	-1.225232373	-1.040569031	-0.184663342
Q2KIJ2	METTL18	bta:783955; Q2KIJ2		-1.147109597	-1.014148806	-0.132960791
A0AAA9SMD9	TRIM59	A0AAA9SMD9	ENSBTAG00000058033	-0.753708858	-0.740669563	-0.013039295
B0JYP6	IGK	B0JYP6		-0.983783699	-0.659707138	-0.324076561
A5D7F6	MAML2	A5D7F6; bta:521194		-0.908386251	-0.876100818	-0.032285432
A5PKL7	LZTS2	A5PKL7; bta:504411		-0.69228058	-0.592180913	-0.100099667
A0A3Q1LWE9	HDAC4	A0A3Q1LWE9	ENSBTAG00000017764	-0.926845202	-0.733446824	-0.193398378
A0A140T852	MRPL30	A0A140T852	ENSBTAG00000038949	-0.852016546	-0.725598016	-0.126418531
G3X7E2	PPP6R2	G3X7E2	ENSBTAG00000018660	-0.937093248	-0.858483414	-0.078609835
E1B8E9	MSL1	bta:787839; E1B8E9	ENSBTAG00000010375	-0.768097359	-0.894835157	0.126737798
Q3T0L3	MRPL17	bta:506988; Q3T0L3	ENSBTAG00000024781	-0.906890596	-0.801609145	-0.10528145
F1MSQ6	NEFH	bta:528842; F1MSQ6	ENSBTAG00000013147	-0.806767625	-0.770241749	-0.036525876
E1BJZ4	GCC1	E1BJZ4	ENSBTAG00000003102	-1.227338244	-1.126878062	-0.100460182
A0AAF6Z1X8	TFPI2	A0AAF6Z1X8	ENSBTAG00000015844	-1.198494154	-1.369862572	0.171368418
A0AAA9TLF7	C8orf33	A0AAA9TLF7	ENSBTAG00000000879	-0.967640857	-0.816735287	-0.15090557
A0A3Q1MT65	PAM	A0A3Q1MT65	ENSBTAG00000012387	-1.202455364	-1.150888221	-0.051567142
A0AAA9TCI2	HMGXB4	A0AAA9TCI2	ENSBTAG00000010533	-1.238502087	-1.287628944	0.049126857
F6R642	MRM1	F6R642	ENSBTAG00000010311	-0.849934711	-0.853818145	0.003883434
A0A3Q1LMJ2	MFSD10	A0A3Q1LMJ2	ENSBTAG00000021130	-0.666629897	-0.652398511	-0.014231386
A0AAA9SG13	CCDC167	A0AAA9SG13	ENSBTAG00000012069	-0.653124152	-0.804913813	0.151789661
A0A452DHX6	AP4M1	A0A452DHX6	ENSBTAG00000002881	-0.681070233	-0.699192252	0.018122018
Q0II32	TIMMDC1	bta:507206; F6PYZ8; Q0II32	ENSBTAG00000012581	-1.758783704	-1.225101709	-0.533681996
A0A3Q1ME72	DOP1B	A0A3Q1ME72	ENSBTAG00000014306	-1.719846724	-1.652847985	-0.066998739
F1MZB7	ZBTB10	F1MZB7	ENSBTAG00000003339	-1.016119665	-1.272684576	0.256564911
Q0P5A5	CKS1B	bta:615827; Q0P5A5	ENSBTAG00000024476	-0.917785237	-0.751135367	-0.166649869
E1B7S1	PARP12	E1B7S1	ENSBTAG00000016546	-0.77606721	-0.756052623	-0.020014587
A0A3Q1M937	KCTD9	A0A3Q1M937	ENSBTAG00000012382	-1.08851305	-0.868106445	-0.220406605
F6R1G6	TRMT13	F6R1G6	ENSBTAG00000015781	-0.671330911	-0.75966581	0.088334899
Q08DR4	UGCG	bta:514357; Q08DR4	ENSBTAG00000016073	-0.771247414	-0.681049605	-0.090197809
A0AAA9SB29	NHEJ1	A0AAA9SB29	ENSBTAG00000031041	-1.248197706	-1.195271953	-0.052925753
A3KN50	MYST1	A3KN50; bta:100125883		-0.751568977	-0.707500339	-0.044068638
A0A3Q1N2Q7	GALNT12	A0A3Q1N2Q7	ENSBTAG00000009037	-1.414194557	-1.452911665	0.038717108
A0A3Q1LT78	DCAF6	A0A3Q1LT78	ENSBTAG00000020969	-0.825837048	-0.952789072	0.126952023
A0A3Q1MFU2	RADX	A0A3Q1MFU2; bta:511907	ENSBTAG00000003668	-0.690907956	-0.911943823	0.221035867
A0AAA9TBU0	TSEN34	A0AAA9TBU0	ENSBTAG00000006486	-2.984149532	-3.189824559	0.205675026
A6QR31	FAM32A	A6QR31; bta:508634	ENSBTAG00000046846	-0.694871194	-0.806275464	0.11140427
E1BER4	CRACR2A	bta:525377; E1BER4; G3MY43	ENSBTAG00000018940	-1.386117887	-1.571941699	0.185823812
Q0VCC3	BLOC1S4	bta:518544; Q0VCC3	ENSBTAG00000003766	-1.100485753	-1.269698136	0.169212383
Q1JQC2	IER3IP1	bta:101906131; bta:768079; Q1JQC2	ENSBTAG00000017330	-0.711180217	-0.729849428	0.018669211
A0JNM0	ATXN7L3B	A0JNM0; bta:777786	ENSBTAG00000035083	-1.52667265	-1.830173648	0.303500997
F1N2C5	IL17RA	F1N2C5	ENSBTAG00000011067	-0.854245054	-0.779568368	-0.074676686
F1MQQ0	CDC45	F1MQQ0	ENSBTAG00000004286	-1.387442491	-1.185911971	-0.20153052
A0AAA9SM30	TSTD2	A0AAA9SM30	ENSBTAG00000014722	-0.799581906	-0.902328922	0.102747016
F1MFC9	TCTN1	F1MFC9	ENSBTAG00000009208	-1.173584344	-1.047366628	-0.126217716
A0AAA9SLP7	ZFAND5	A0AAA9SLP7	ENSBTAG00000009417	-1.091280507	-0.90841645	-0.182864057
Q2KJ69	RLIG1	bta:767986; Q2KJ69		-1.261575419	-1.43271614	0.171140722
Q0P5J1	FAR2	bta:534380; Q0P5J1	ENSBTAG00000011095	-1.836706254	-1.786345293	-0.050360961
A0AAA9TWJ8	TMTC2	A0AAA9TWJ8	ENSBTAG00000002888	-0.687992131	-0.640626676	-0.047365455
E1BB91	COL6A3	E1BB91	ENSBTAG00000030190	-1.697054192	-1.88684753	0.189793337
A0AAA9TEI4	MREG	A0AAA9TEI4; bta:613445	ENSBTAG00000013215	-0.846867595	-0.75439332	-0.092474276
A0AAA9SE34	PRR5	A0AAA9SE34	ENSBTAG00000039784	-0.807102017	-0.885301229	0.078199212
A0AAA9SDI3	ABCA2	A0AAA9SDI3	ENSBTAG00000047254	-3.208717484	-4.088641032	0.879923548
A0AAA9TJZ8	MFAP5	A0AAA9TJZ8	ENSBTAG00000000310	-0.754443391	-0.819845866	0.065402475
A2VDP2	PCMTD1	A2VDP2; bta:521261	ENSBTAG00000017492	-0.996869377	-0.822318866	-0.174550511
A0AAA9TJC7	CEP290	A0AAA9TJC7	ENSBTAG00000018745	-0.654750831	-0.909397451	0.25464662
Q3SZ23	DNAJC30	bta:617118; F1MW37; Q3SZ23	ENSBTAG00000009022	-1.014808686	-1.021695071	0.006886386
E1B7S6	NR4A3	bta:528877; E1B7S6	ENSBTAG00000001864	-2.744517948	-3.349546072	0.605028124
A0AAA9S206	PHF20	A0AAA9S206; bta:529591	ENSBTAG00000005571	-2.121644986	-2.489965987	0.368321
A5PKH9	WDR51B	A5PKH9; bta:525136		-2.242926832	-1.954328142	-0.288598691
Q58D25	KLHDC3	Q58D25		-3.206156019	-4.403864177	1.197708158
F1MTW9	TRIM35	bta:518310; F1MTW9	ENSBTAG00000009958	-1.200051669	-1.267050408	0.066998739
A5PJF7	CCL2	A5PJF7		-0.62063277	-0.783447383	0.162814612
A0A3Q1NJF2	RLIM	A0A3Q1NJF2	ENSBTAG00000015630	-1.014738859	-0.618627864	-0.396110996
A0AAA9TCQ9	AHNAK	A0AAA9TCQ9	ENSBTAG00000013468	-0.098085	0.051886742	-0.149971741
E1BF59	PLEC	E1BF59	ENSBTAG00000011922	-0.044669049	-0.073691951	0.029022901
A0A3Q1NIP5	PLEC	A0A3Q1NIP5	ENSBTAG00000011922	-0.094592537	-0.058730202	-0.035862335
A0A3Q1M2E4	PLEC	A0A3Q1M2E4	ENSBTAG00000011922	-0.039801008	-0.207745645	0.167944637
A0A3Q1M6Q7	PLEC	A0A3Q1M6Q7	ENSBTAG00000011922	0.11649968	0.104182038	0.012317642
E1BDX8	DYNC1H1	E1BDX8	ENSBTAG00000016598	-0.06994412	-0.098264016	0.028319896
A0AAA9S484	MACF1	A0AAA9S484	ENSBTAG00000021024	-0.110877022	0.00897281	-0.119849832
A0AAA9RW41	TLN1	A0AAA9RW41	ENSBTAG00000025868	-0.100460182	-0.112999377	0.012539196
F1MQ37	MYH9	F1MQ37	ENSBTAG00000010402	0.299743726	-0.151513961	0.451257687
F1N169	FLNA	bta:281165; F1N169	ENSBTAG00000011190	-0.028238705	-0.029535509	0.001296805
E1BKX7	FLNB	bta:613533; E1BKX7	ENSBTAG00000022004	-0.029016074	-0.01282404	-0.016192034
E1BFB0	SPTAN1	E1BFB0	ENSBTAG00000015327	-0.141542265	-0.155592527	0.014050262
A0A3Q1NKL1	SPTAN1	A0A3Q1NKL1	ENSBTAG00000015327	-0.081367551	-0.132741191	0.05137364
A0A3Q1LV98	FLNC	A0A3Q1LV98	ENSBTAG00000006253	0.097726032	0.058005603	0.039720429
A0A3Q1MXU7	SPTBN1	A0A3Q1MXU7	ENSBTAG00000006995	-0.106466702	-0.169341977	0.062875274
Q27991	MYH10	bta:317655; Q27991		0.341277187	0.037552746	0.303724441
A0A3Q1N191	GCN1	A0A3Q1N191	ENSBTAG00000017379	-0.09238643	-0.056692006	-0.035694424
A0A3Q1MTY6	MAP1B	A0A3Q1MTY6	ENSBTAG00000001961	0.002691596	0.047696742	-0.045005146
F1N7I5	NUMA1	F1N7I5	ENSBTAG00000018449	-0.10255584	-0.066171689	-0.03638415
A0AAA9T5L8	GOLGB1	A0AAA9T5L8	ENSBTAG00000008862	0.114089343	0.041476636	0.072612707
A0A3Q1M9Z4	MAP1A	A0A3Q1M9Z4	ENSBTAG00000014001	-0.124429168	-0.074638801	-0.049790367
A0A3Q1LMV9	EPPK1	A0A3Q1LMV9	ENSBTAG00000045727	-0.295035089	-0.443943376	0.148908287
A0A3Q1LLS4	RANBP2	A0A3Q1LLS4	ENSBTAG00000000152	-0.048223947	-0.043486028	-0.004737919
A0AAA9SF14	CLTC	A0AAA9SF14	ENSBTAG00000016708	0.054881351	0.01681331	0.038068041
E1BGJ0	LRP1	E1BGJ0	ENSBTAG00000010830	-0.220953324	-0.065645744	-0.15530758
A0A3Q1MIB0	IQGAP1	A0A3Q1MIB0	ENSBTAG00000013060	-0.015466801	0.005118971	-0.020585772
A0AAA9RXD6	RRBP1	A0AAA9RXD6	ENSBTAG00000015438	0.083867611	-0.10496956	0.188837171
F1MQ21	NES	F1MQ21	ENSBTAG00000015794	0.098563834	-0.040641984	0.139205819
A0AAA9S236	MKI67	A0AAA9S236	ENSBTAG00000002444	-0.114486015	-0.290663881	0.176177866
A0AAA9TU63	UBR4	A0AAA9TU63	ENSBTAG00000015240	-0.219532767	-0.140327884	-0.079204883
A0A3Q1LIB4	VCL	A0A3Q1LIB4	ENSBTAG00000021879	0.021238796	0.027250034	-0.006011238
A0AAA9T4R9	TPR	A0AAA9T4R9	ENSBTAG00000060237	0.013646193	0.080005643	-0.066359451
E1BNR0	APOB	E1BNR0	ENSBTAG00000008505	-0.210271745	0.001377275	-0.21164902
A0AAA9TDR0	HSPG2	A0AAA9TDR0	ENSBTAG00000017122	-0.412371272	-0.236067358	-0.176303914
F1N647	FASN	F1N647	ENSBTAG00000015980	-0.26399015	-0.378869953	0.114879803
A0AAA9SFW6	KTN1	A0AAA9SFW6	ENSBTAG00000017713	0.081816948	0.186281172	-0.104464225
A0AAA9TMZ2	EPRS1	A0AAA9TMZ2	ENSBTAG00000006898	0.131177445	0.07075666	0.060420786
A0AAA9SKJ1	A2M	A0AAA9SKJ1	ENSBTAG00000018137	-0.107217076	0.347082218	-0.454299294
G3X757	VCP	G3X757	ENSBTAG00000015000	0	-0.109018985	0.109018985
F1N3I4	MYOF	F1N3I4	ENSBTAG00000016918	-0.20029865	-0.206157319	0.005858668
A0A3Q1MSB2	AKAP12	A0A3Q1MSB2	ENSBTAG00000014788	-0.093420297	-0.185866545	0.092446249
B8Y9S9				-0.435709114	-0.57834679	0.142637675
A0A3Q1MPS4	ACTN1	A0A3Q1MPS4	ENSBTAG00000018255	0.041375928	0.106359873	-0.064983945
P48616	VIM	bta:280955; P48616; Q17QM7	ENSBTAG00000018463	-0.013546532	-0.112110366	0.098563834
A0A3Q1M820	SACS	A0A3Q1M820; bta:539378	ENSBTAG00000001867	-0.627823715	-0.502880848	-0.124942867
F1MRT9	UTRN	F1MRT9	ENSBTAG00000009665	-0.041356088	0.136666381	-0.178022469
A0A3Q1MYL9	MAP4	A0A3Q1MYL9	ENSBTAG00000015839	0.126671803	0.272388634	-0.145716831
A0AAA9SGA6	HUWE1	A0AAA9SGA6	ENSBTAG00000006122	-0.002777085	-0.090153061	0.087375976
Q3SYU2	EEF2	bta:281138; Q3SYU2	ENSBTAG00000004258	-0.217591435	-0.296393003	0.078801568
Q76LV1	HSP90AB1	bta:767874; Q76LV1	ENSBTAG00000000778	0.331514144	0.126532406	0.204981738
Q95M18	HSP90B1	bta:282646; Q3MHX8; Q95M18	ENSBTAG00000003362	0.426566209	0.310399861	0.116166348
Q76LV2	HSP90AA1	bta:281832; Q76LV2		0.528042534	0.382563893	0.145478641
A0A140T856	COPA	A0A140T856	ENSBTAG00000004333	0.04858018	-0.064964729	0.113544909
E1BPX9	LRPPRC	E1BPX9	ENSBTAG00000016368	-0.403393408	-0.442578192	0.039184784
E1B7R4	EIF3A	E1B7R4	ENSBTAG00000002528	-0.068887303	-0.174763558	0.105876256
A7Z025	PRPF8	A7Z025; bta:507371	ENSBTAG00000011488	-0.139562066	-0.061639066	-0.077923001
E1BBY7	HSPA4	bta:536558; E1BBY7	ENSBTAG00000015683	0.381249186	0.296774618	0.084474568
E1BH78	SNRNP200	bta:510529; E1BH78	ENSBTAG00000000848	-0.165621904	-0.04580369	-0.119818215
Q2HJ49	MSN	bta:540426; Q2HJ49	ENSBTAG00000003418	0.017124178	-0.042242449	0.059366626
P08169	IGF2R	bta:281849; P08169		0.233353921	0.275570041	-0.04221612
Q0IIM3	HSPH1	bta:507165; Q0IIM3	ENSBTAG00000005012	0.876210978	0.517924382	0.358286595
A0A3Q1NND0	HDLBP	A0A3Q1NND0	ENSBTAG00000004315	-0.108657064	-0.199937571	0.091280507
E3W9A2	CLIP1	bta:534624; E3W9A2	ENSBTAG00000016779	0.213026578	0.111217479	0.101809099
A0A3Q1LQZ3	CLIP1	A0A3Q1LQZ3	ENSBTAG00000016779	0.250737047	0.17732725	0.073409797
A0A140T897	ALB	A0A140T897; bta:280717	ENSBTAG00000017121	0.024746662	0.719892081	-0.695145418
A0AAA9U1R4	TPM1	A0AAA9U1R4	ENSBTAG00000005373	-0.186878135	-0.152411731	-0.034466404

Accession	Gene_Symbol	Gene_ID	Ensembl_Gene ID	log2(ratio(OV24H/OV0H))	log2(ratio(OV24R/OV0H))	log2(ratio(OV24H/OV24R))
Q3ZC87	PKM2	Q3ZC87		0.064784947	-0.04279494	0.107579887
F1N3F6	CHD4	F1N3F6	ENSBTAG00000014734	-0.087292078	-0.181756762	0.094464684
A0A3Q1MH82	LMO7	A0A3Q1MH82	ENSBTAG00000010693	0.18074922	0.265789793	-0.085040573
A0AAA9SJJ8	CAD	A0AAA9SJJ8	ENSBTAG00000017894	-0.039231818	-0.135636302	0.096404484
F1MX04	EIF4G1	F1MX04	ENSBTAG00000012881	-0.138757375	-0.099436208	-0.039321167
F1MPE5	IPO5	F1MPE5	ENSBTAG00000039374	0.058587026	-0.032560862	0.091147888
F1MLW0	CALD1	F1MLW0	ENSBTAG00000013953	-0.003267714	0.040233925	-0.043501639
A5D7D1	ACTN4	A5D7D1; bta:522269	ENSBTAG00000011215	0.054812885	0.145250356	-0.090437471
A0AAA9SIS5	LMNA	A0AAA9SIS5	ENSBTAG00000017574	-0.109451142	-0.182330022	0.07287888
A0AAA9TE87	UGGT1	A0AAA9TE87	ENSBTAG00000005444	0.097487845	0.129909323	-0.032421478
A5PKJ3	DCTN1	A5PKJ3; bta:511045		0.027046876	0.017141133	0.009905743
A0JN39	COPB1	A0JN39; bta:535533	ENSBTAG00000006556	0.0799607	-0.00155212	0.08151282
A0AAA9RXQ2	PKM	A0AAA9RXQ2	ENSBTAG00000001601	0.589433205	0.51806151	0.071371695
P56701	PSMD2	A7E3R5; bta:539784; P56701; Q2HJE3; Q9TRA7	ENSBTAG00000005660	-0.096785063	-0.152309041	0.055523978
A0A3Q1MVZ1	PARP1	A0A3Q1MVZ1	ENSBTAG00000000837	0.141438405	0.156793794	-0.01535539
A0A3Q1MJM1	HYOU1	A0A3Q1MJM1	ENSBTAG00000010322	0.282362667	0.256068216	0.02629445
A0A3Q1LJA4	DCTN1	A0A3Q1LJA4	ENSBTAG00000004887	-0.166705266	-0.091029876	-0.07567539
A0AAF7A102	ANXA6	A0AAF7A102	ENSBTAG00000014809	-0.247927513	-0.209792385	-0.038135129
A0A3Q1MHX1	ECPAS	A0A3Q1MHX1	ENSBTAG00000006712	0.053617516	0.001399995	0.05221752
E1BKE7	ITPR3	E1BKE7	ENSBTAG00000013245	-0.087273821	0.07513433	-0.162408151
A0AAF6Z0Y8	ANXA6	A0AAF6Z0Y8	ENSBTAG00000014809	-0.083226907	-0.139382127	0.05615522
A0A3Q1M9W3	AFDN	A0A3Q1M9W3	ENSBTAG00000005557	0.072058148	-0.012578066	0.084636214
A0AAA9SCC2	DST	A0AAA9SCC2	ENSBTAG00000021237	-0.117194099	-0.004123952	-0.113070146
F2Z4J5	DNMT1	F2Z4J5	ENSBTAG00000002736	-0.154931799	-0.29413361	0.139201811
F1MDU7	TOP2A	bta:787696; F1MDU7	ENSBTAG00000019262	0.006829338	-0.274373975	0.281203313
E1BKX3	MYBBP1A	bta:513045; E1BKX3	ENSBTAG00000007430	-0.001548787	-0.023408221	0.021859434
A7MBI5	DPYSL3	A7MBI5; bta:509796	ENSBTAG00000010661	0.046194823	-0.026848958	0.073043781
A0AAA9S4P4	DST	A0AAA9S4P4	ENSBTAG00000021237	-0.277453466	-0.199112014	-0.078341451
A0AAA9T5V4	AARS1	A0AAA9T5V4	ENSBTAG00000019810	0.013776503	0.177764828	-0.163988325
A0AAA9TRW0	CALD1	A0AAA9TRW0	ENSBTAG00000013953	0.013674937	0.37056287	-0.356887933
F1MM34	CTNNA1	F1MM34	ENSBTAG00000014182	0.051412573	-0.111568229	0.162980802
F1N1G7	KIF5B	F1N1G7	ENSBTAG00000002280	-0.075238728	-0.059297184	-0.015941544
G3N0R5	HSPD1	G3N0R5	ENSBTAG00000012586	0.238275463	0.209405404	0.028870058
A0AAA9T7M1	EEA1	A0AAA9T7M1	ENSBTAG00000000421	0.089655187	0.195067427	-0.10541224
A0A140T886	COPG1	A0A140T886; bta:338055	ENSBTAG00000013212	0.067853092	-0.02987053	0.097723622
A0AAA9SJL9	PARP1	A0AAA9SJL9	ENSBTAG00000000837	0.140007479	0.135831803	0.004175676
V9GW27				-0.051124465	-0.078855421	0.027730956
A0AAA9SHQ7	UBA1	A0AAA9SHQ7	ENSBTAG00000016764	0.078749674	0.041982449	0.036767225
A0AAA9SYD6	CENPF	A0AAA9SYD6	ENSBTAG00000024449	0.108338682	-0.079031454	0.187370136
Q28141	DHX9	bta:281115; Q28141		-0.062894791	-0.064390587	0.001495796
Q29443	TF	bta:280705; Q0IIK2; Q29443		-0.044077615	0.035729787	-0.079807401
A7MBA2	PSMD1	A7MBA2; bta:511803	ENSBTAG00000005119	0.002896979	0.005788152	-0.002891173
G3X6N3	TF	G3X6N3	ENSBTAG00000007273	-0.027760693	0.53225545	-0.560016144
A0A3Q1MW77	ROCK2	A0A3Q1MW77	ENSBTAG00000005847	0.223294106	0.209709306	0.013584799
A0A3Q1LW78	STIP1	A0A3Q1LW78	ENSBTAG00000015663	0.360824532	0.256623712	0.104200819
F1MD77	LAMC1	F1MD77	ENSBTAG00000011966	-0.160682849	-0.099800607	-0.060882242
A0A3Q1N4H7	TNKS1BP1	A0A3Q1N4H7; bta:783548	ENSBTAG00000016469	-0.025506875	0.048171999	-0.073678874
A0A3Q1LMS5	HSPA8	A0A3Q1LMS5	ENSBTAG00000013162	0.400814599	0.208762207	0.192052393
A0A3Q1MJD5	HNRNPM	A0A3Q1MJD5; bta:515290	ENSBTAG00000021039	0.013003849	-0.059256586	0.072260435
F1MYX5	LCP1	F1MYX5	ENSBTAG00000007079	0.047947483	-0.073477012	0.121424495
A0AAA9S1P6	MCM4	A0AAA9S1P6	ENSBTAG00000017021	-0.106267626	-0.149199021	0.042931394
Q9XSX1				-0.077598995	-0.02491257	-0.052686425
B0JYQ0	ALB	B0JYQ0		-0.051817528	0.450147629	-0.501965157
A0AAA9RTW0	SMCHD1	A0AAA9RTW0	ENSBTAG00000003354	0.035069667	0.065241195	-0.030171528
F1MHU9	CNOT1	F1MHU9	ENSBTAG00000020782	0.045667751	0.062427223	-0.016759473
A0AAF6ZGM3	TPM2	A0AAF6ZGM3	ENSBTAG00000011424	-0.118548862	-0.058057102	-0.06049176
A0AAA9TTT0	TPM1	A0AAA9TTT0	ENSBTAG00000005373	-0.994596641	-0.336892534	-0.657704107
Q0VCX2	HSPA5	A7E3V5; bta:415113; Q0VCX2	ENSBTAG00000007662	0.366356683	0.356025319	0.010331364
E1BMZ9	SMC4	E1BMZ9	ENSBTAG00000005862	-0.283108846	-0.304006187	0.020897341
Q3ZBH0	CCT2	bta:505313; Q3ZBH0	ENSBTAG00000019156	0.13095377	0.023216283	0.107737487
A0A3Q1MQV3	THBS1	A0A3Q1MQV3	ENSBTAG00000002006	-0.122600701	-0.06481141	-0.057789291
A0A3Q1LZN8	COL1A2	A0A3Q1LZN8	ENSBTAG00000013472	-0.570048524	-0.60449467	0.034446146
F2Z4C6	EIF4G2	F2Z4C6	ENSBTAG00000020308	0.135953072	0.091558953	0.044394119
A0A3Q1M168	PYGL	A0A3Q1M168	ENSBTAG00000011494	-0.004253649	-0.017090368	0.012836719
A0AAA9TXI1	NUP153	A0AAA9TXI1; bta:513270	ENSBTAG00000017128	0.199208937	0.072451794	0.126757142
E1BLD1	HECTD1	bta:536240; E1BLD1	ENSBTAG00000032477	-0.036567961	-0.143094735	0.106526774
A4FV23	DDX21	A4FV23; bta:781917		-0.22216057	-0.390666294	0.168505724
A0AAA9T089	UPF1	A0AAA9T089	ENSBTAG00000020922	0.102441405	0.119945152	-0.017503748
A8E4L8	LMO7	A8E4L8; F1N6J9		0.188830104	0.478568219	-0.289738115
E1BKT0	LUZP1	bta:516426; E1BKT0	ENSBTAG00000009502	0.013151368	0.005274942	0.007876426
A0AAA9RYK8	EIF5B	A0AAA9RYK8	ENSBTAG00000034255	0.055853235	0.030343228	0.025510007
Q863B3	SND1	bta:404098; Q3MHZ9; Q863B3	ENSBTAG00000010692	-0.036394237	-0.131611305	0.095217067
A0AAA9TE63	MATR3	A0AAA9TE63	ENSBTAG00000006756	0.041306974	0.020801319	0.020505655
A0AAA9TK25	IARS1	A0AAA9TK25	ENSBTAG00000000974	0.074578121	0.15719265	-0.082614528
Q0VCK0	ATIC	bta:506343; Q0VCK0		-0.056684596	-0.033225624	-0.023458973
A7Z065	SMC3	A7Z065	ENSBTAG00000013905	-0.083760862	-0.125280131	0.041519269
Q2UVX4	C3	bta:280677; Q2KIZ4; Q2UVX4	ENSBTAG00000017280	-0.024609467	0.459708873	-0.484318341
A0A3Q1LJT3	GLG1	A0A3Q1LJT3	ENSBTAG00000002303	-0.188825615	-0.209313937	0.020488321
A4FUD0	MTHFD1	A4FUD0; bta:534382	ENSBTAG00000009641	-0.09483134	-0.170283569	0.075452228
F1MEN8	PDIA4	F1MEN8	ENSBTAG00000017143	0.375433425	0.269379917	0.106053508
Q3ZCH0	HSPA9	A5D9A6; bta:517535; Q3ZCH0	ENSBTAG00000011419	0.012383724	0.119298928	-0.106915204
G3X6B2	PRRC2C	G3X6B2	ENSBTAG00000001821	-0.031445558	-0.063591817	0.032146259
A0AAA9S4Y0	TJP1	A0AAA9S4Y0	ENSBTAG00000015398	0.281199329	0.318211113	-0.037011784
Q3ZBH9	MCM7	bta:539924; Q3ZBH9	ENSBTAG00000030965	-0.051060489	0.197198564	-0.248259053
F1MD74	CAST	F1MD74	ENSBTAG00000000874	-0.217005875	0.019653116	-0.23665899
O97593	SMC1A	bta:282370; O97593	ENSBTAG00000017761	0.007152692	-0.132064246	0.139216938
A0AAF6ZVV1	PC	A0AAF6ZVV1	ENSBTAG00000019700	0.005986295	-0.01356068	0.019546975
A0A3Q1LUJ8	ATP1A1	A0A3Q1LUJ8	ENSBTAG00000001246	0.063846482	0.044987455	0.018859027
A5D7A2	GARS1	A5D7A2; bta:408010	ENSBTAG00000018972	0.206552975	0.512734173	-0.306181198
F1MJK3	LOC506828	F1MJK3	ENSBTAG00000038461	-0.294362208	0.008998127	-0.303360335
A0A3Q1MPF1	GDI2	A0A3Q1MPF1	ENSBTAG00000005316	0.044896538	0.046235466	-0.001338928
A7MBJ5	CAND1	A7MBJ5; bta:538086	ENSBTAG00000009939	0.037776651	0.039210029	-0.001433378
A0A3Q1MLL6	PPP1R12A	A0A3Q1MLL6	ENSBTAG00000014609	0.019686203	0.083877261	-0.064191058
A0A3Q1MXS2	ITGAV	A0A3Q1MXS2	ENSBTAG00000019929	-0.247848002	-0.174225139	-0.073622863
A0A3Q1LVC7	EZR	A0A3Q1LVC7	ENSBTAG00000010347	0.101814813	0.147576988	-0.045762175
A6QR15	TPM4	A6QR15; bta:535277	ENSBTAG00000004553	0.195292064	0.186627203	0.008664861
F1MX61	SF3B1	bta:510177; F1MX61	ENSBTAG00000008915	-0.01944564	0.029413762	-0.048859401
Q3T0K2	CCT3	bta:504735; Q3T0K2	ENSBTAG00000006370	0.080949108	-0.012866931	0.093816039
A0A3Q1LTK7	ERC1	A0A3Q1LTK7	ENSBTAG00000013658	0.083416008	0.225066556	-0.141650547
Q3ZCI9	CCT8	bta:281047; Q3ZCI9	ENSBTAG00000014233	0.148926811	0.039878024	0.109048787
A0A3Q1N4A7	CIT	A0A3Q1N4A7	ENSBTAG00000008963	-0.310082979	-0.49523234	0.185149361
F1MF78	SYNE2	F1MF78	ENSBTAG00000025450	-0.198683994	-0.219582888	0.020898893
F1ME65	CKAP4	bta:515784; F1ME65	ENSBTAG00000011913	0.016640266	-0.066795439	0.083435704
F1MY41	SMC2	bta:539217; F1MY41	ENSBTAG00000008772	-0.275199787	-0.255345872	-0.019853915
F1MY84	CSPG4	F1MY84	ENSBTAG00000012219	-0.230663683	0.02078575	-0.251449433
A0A3Q1M0D6	SRRM2	A0A3Q1M0D6; bta:539515	ENSBTAG00000012683	-0.037313158	-0.051558206	0.014245048
E1BE98	XPO1	bta:510568; E1BE98	ENSBTAG00000013254	0.183745496	0.109887904	0.073857592
Q3SZ54	EIF4A1	bta:504958; Q3SZ54	ENSBTAG00000000132	-0.120294234	-0.198215707	0.077921473
F1N6Y1	GANAB	bta:540155; F1N6Y1	ENSBTAG00000016956	0.053231681	0.055979672	-0.002747991
F1MNT4	LAMB1	F1MNT4	ENSBTAG00000011412	-0.053695008	-0.094917671	0.041222663
E1BDU8	LIMA1	bta:540637; E1BDU8	ENSBTAG00000012342	0.028391601	-0.02588546	0.054277061
A0AAA9SFD3	GOLGA3	A0AAA9SFD3	ENSBTAG00000009755	0.075057667	0.029901486	0.045156181
A0AAA9SMJ2	ACTB	A0AAA9SMJ2	ENSBTAG00000026199	0.084639813	0.033034779	0.051605034
A0A3Q1M1Q5	CTNND1	A0A3Q1M1Q5	ENSBTAG00000002411	-0.022678059	-0.135916109	0.11323805
A0A3Q1M5Q7	NASP	A0A3Q1M5Q7	ENSBTAG00000015346	0.173994666	0.195507758	-0.021513093
F1N365	FLII	F1N365	ENSBTAG00000016161	-0.067869336	-0.224074865	0.156205529
A0AAA9RY84	NAA15	A0AAA9RY84	ENSBTAG00000004745	0.048549525	0.009228358	0.039321167
A0JN43	FAM62A	A0JN43; bta:520669; F1N785		-0.125647261	-0.129843178	0.004195917
F6QQ60	TPM4	F6QQ60	ENSBTAG00000004553	-0.039943512	0.059871456	-0.099814968
A0AAA9TWX8	DDX3X	A0AAA9TWX8	ENSBTAG00000035907	-0.281164733	-0.211008832	-0.070155901
A4FUD9	MCM3	A4FUD9; bta:281302		0.022832446	0.028484539	-0.005652094
Q08DS7	AP2B1	bta:282183; Q08DS7	ENSBTAG00000020316	0.012661471	0.025212788	-0.012551317
A0A3Q1LK06	LRRFIP1	A0A3Q1LK06	ENSBTAG00000005354	-0.165976408	-0.253439249	0.087462841
A0A3Q1MVC6	RAI14	A0A3Q1MVC6	ENSBTAG00000007071	0.139841209	0.168410361	-0.028569152
Q9TRY0	FKBP4	A5D9B2; bta:508535; Q3T0C4; Q9TRX9; Q9TRY0		0.557758143	0.343682159	0.214075984
A7E3Q8	PLS3	A7E3Q8	ENSBTAG00000011613	-0.007463519	-0.056166711	0.048703192
A7E3W7	DDX1	A7E3W7	ENSBTAG00000009906	0.101671418	0.09453994	0.007131478
F1MXF3	SYNE1	F1MXF3	ENSBTAG00000009362	0.292228317	0.192109854	0.100118463
A0AAA9TC88	ACO2	A0AAA9TC88	ENSBTAG00000006429	-0.012990839	0.067375435	-0.080366274
A2VDN7	HNRNPU	A2VDN7; bta:539950; F1MFT4	ENSBTAG00000007606	0.112110366	-0.035405336	0.147515702
K4JDT2	A2M	K4JDT2		-0.12407435	0.442820656	-0.566895006
Q2KJD0	TUBB5	A5D9A3; bta:615087; Q2KJD0	ENSBTAG00000006969	0.075001037	-0.001540518	0.076541555
E1BMW2	AP2A1	E1BMW2	ENSBTAG00000019180	-0.079757082	-0.056320527	-0.023436555
Q3T0P6	PGK1	bta:507476; Q3T0P6	ENSBTAG00000000894	0.026022791	-0.006191834	0.032214625
Q2NKZ1	CCT7	bta:514355; Q2NKZ1	ENSBTAG00000012756	0.170286806	0.082281224	0.088005582
A0A3Q1M1I5	EIF4G3	A0A3Q1M1I5	ENSBTAG00000040215	0.275995722	0.303191532	-0.027195811
A0AAA9SZH0	DNAJC13	A0AAA9SZH0	ENSBTAG00000002966	-0.151860104	-0.101283336	-0.050576768
A0AAA9SDW8	GART	A0AAA9SDW8	ENSBTAG00000009188	-0.039075379	-0.09024564	0.05117026
A0AAA9SZZ0	SEC31A	A0AAA9SZZ0	ENSBTAG00000020525	0.279096179	0.535675563	-0.256579384
A0AAF6YPM1	OGDH	A0AAF6YPM1	ENSBTAG00000006029	-0.142293882	-0.117582129	-0.024711753
A0A3Q1M0F0	HK1	A0A3Q1M0F0	ENSBTAG00000012380	-0.00168835	-0.056786026	0.055097676
A0AAA9T8D8	PDS5A	A0AAA9T8D8	ENSBTAG00000017809	-0.051981255	-0.064130337	0.012149083
A0A3Q1LHC9	MCM2	A0A3Q1LHC9	ENSBTAG00000014380	-0.025535092	-0.019486047	-0.006049045
A0AAA9RXY5	SEC31A	A0AAA9RXY5	ENSBTAG00000020525	0.158922507	0.16855427	-0.009631762
Q3SYU9	MVP	bta:516456; Q3SYU9		-0.334900392	-0.240337391	-0.094563002
A7E3V3	IMMT	A7E3V3		-0.025943267	-0.055327074	0.029383807
F1MIF2	AP1B1	F1MIF2	ENSBTAG00000001189	0.015266757	0.019402531	-0.004135775
Q32LP2	RDX	bta:517111; Q32LP2	ENSBTAG00000004672	0.108731914	-0.014387106	0.12311902
E1BE13	BIRC6	E1BE13	ENSBTAG00000027932	-0.322250449	-0.230148865	-0.092101584
F1MWN1	CSE1L	F1MWN1	ENSBTAG00000005957	0.074828613	0.061902439	0.012926174
A0A452DII8	ATP5F1B	A0A452DII8	ENSBTAG00000013315	-0.04341436	-0.033166864	-0.010247496
A5D7D9	DHX15	A5D7D9; bta:512327	ENSBTAG00000008896	0.051994638	0.040601133	0.011393505
A0AAA9TCS0	ENO1	A0AAA9TCS0	ENSBTAG00000013411	0.086546448	-0.015658413	0.10220486
A0A3Q1LYI6	IMMT	A0A3Q1LYI6	ENSBTAG00000003307	-0.057523701	0.380558597	-0.438082298
A0AAA9T403	SUPT16H	A0AAA9T403	ENSBTAG00000020421	-0.04467859	-0.024478976	-0.020199614
A0AAA9S717	DNM1L	A0AAA9S717	ENSBTAG00000011395	0.136624992	0.110017157	0.026607835
A0AAA9T689	ANLN	A0AAA9T689	ENSBTAG00000009218	-0.268519945	-0.36923381	0.100713865
A0AAA9T8P4	AP1B1	A0AAA9T8P4	ENSBTAG00000001189	0.002709287	0.002709287	0
A0A3Q1NE05	KHSRP	A0A3Q1NE05	ENSBTAG00000021018	-0.005730673	0.029718163	-0.035448836
A0AAA9TT10	DDB1	A0AAA9TT10	ENSBTAG00000021569	-0.245830221	-0.262776113	0.016945892
F1MNF8	TUBA1A	bta:404053; F1MNF8	ENSBTAG00000001489	0.008347343	-0.009800004	0.018147347
E1B8K6				0.100511237	0.100511237	0
A0AAA9TV68	TOP1	A0AAA9TV68; bta:534799	ENSBTAG00000007960	0.207256949	0.040881934	0.166375015
A0A3Q1LJL3	OPA1	A0A3Q1LJL3; bta:524142	ENSBTAG00000019514	-0.146278106	-0.222870531	0.076592425
A0A3Q1MEP1	CEP170	A0A3Q1MEP1	ENSBTAG00000007566	0.145273893	0.108648661	0.036625232
A0A3Q1LVF3	SUPT6H	A0A3Q1LVF3	ENSBTAG00000009036	0.107675518	-0.003553438	0.111228955
Q3SZ00	HADHA	Q3SZ00	ENSBTAG00000015038	-0.044999985	-0.053596008	0.008596024
Q27966-3	MYO1C	Q27966-3		-0.092132841	0.085345555	-0.177478396
Q2KJH6	SERPINH1	bta:510850; Q2KJH6	ENSBTAG00000001027	0.18950537	0.138317916	0.051187455
A0A3Q1NB13	ABCF1	A0A3Q1NB13	ENSBTAG00000006927	0.061587798	0.011935498	0.049652301
O62654	DES	A2VDU8; bta:280765; O62654; O62655	ENSBTAG00000005353	-0.05431112	0.116023796	-0.170334916
A0AAA9S7W6	USP7	A0AAA9S7W6	ENSBTAG00000005455	-0.025865743	-0.007138535	-0.018727208
F1MWD3	CCT5	F1MWD3	ENSBTAG00000017889	0.156288493	0.066138464	0.090150029
A0A3Q1NGQ6	ALDOA	A0A3Q1NGQ6	ENSBTAG00000012927	-0.07513433	-0.181816898	0.106682567
A0AAF6YZT3	MOV10	A0AAF6YZT3	ENSBTAG00000014297	-0.196426197	-0.150921439	-0.045504758
A0A3Q1MWL3	NCKAP1	A0A3Q1MWL3	ENSBTAG00000033662	0.044947406	0.074978569	-0.030031163
F1MH31	NUP214	F1MH31	ENSBTAG00000021896	0.115361733	0.163069742	-0.047708009
E1BHU1	TCOF1	bta:513591; E1BHU1	ENSBTAG00000015224	0.043721377	0.238159737	-0.19443836
G3X755	PLXNB2	G3X755	ENSBTAG00000014966	0.033105974	0.13176861	-0.098662636
E1BCV4	NUP98	E1BCV4	ENSBTAG00000008432	-0.064732701	0.013188772	-0.077921473
A0A3Q1LZ02	HK2	A0A3Q1LZ02; bta:788926	ENSBTAG00000013108	0.070591968	0.060424942	0.010167026
E1BMD6	LPP	bta:524132; E1BMD6	ENSBTAG00000044001	0.100603941	0.133125083	-0.032521142
A0A3Q1MMV9	AHCTF1	A0A3Q1MMV9	ENSBTAG00000013949	0.045658717	0.111546653	-0.065887936
A7Z014	TKT	A7Z014; F1MUX4	ENSBTAG00000003758	0.068552669	0.103568111	-0.035015441
A0AAA9TM36	NCAPD2	A0AAA9TM36	ENSBTAG00000014730	-0.218345588	-0.272793372	0.054447784
F1MJI7	LMNB2	bta:516326; F1MJI7	ENSBTAG00000013624	-0.007502331	-0.11522781	0.107725479
A0AAA9SH52	TPM3	A0AAA9SH52	ENSBTAG00000033217	0.080064389	0.0726278	0.007436589
A0A3Q1LUN6	GMPS	A0A3Q1LUN6	ENSBTAG00000013013	-0.21988517	-0.218291912	-0.001593258
A0AAA9S813	MYO18A	A0AAA9S813	ENSBTAG00000017380	0.091423028	0.231991226	-0.140568198

Accession	Gene_Symbol	Gene_ID	Ensembl_Gene ID	log2(ratio(OV24H/OV0H))	log2(ratio(OV24R/OV0H))	log2(ratio(OV24H/OV24R))
P41541	USO1	bta:317724; P41541; Q32PH6	ENSBTAG00000017028	0.230574534	0.281498469	-0.050923935
E1BLV6	VARS1	E1BLV6	ENSBTAG00000005631	-0.006847165	-0.075727326	0.068880161
Q2HJ81	TUBB6	bta:534206; Q2HJ81		-0.166681059	-0.164966626	-0.001714433
A0A3Q1ML77	CSDE1	A0A3Q1ML77	ENSBTAG00000000678	-0.147249563	-0.272780446	0.125530882
A0AAA9S5Z7	TPM3	A0AAA9S5Z7	ENSBTAG00000033217	0.211308922	0.195147585	0.016161337
A0AAA9S0P0	LOC100141266	A0AAA9S0P0	ENSBTAG00000056851	-0.056583528	0.037652541	-0.09423607
E1BKM4	PDCD6IP	E1BKM4	ENSBTAG00000017426	0.083015902	0.11886935	-0.035853448
A0AAA9SNY0	RARS1	A0AAA9SNY0	ENSBTAG00000014626	0.061046205	0	0.061046205
A0A3Q1LXE1	PPFIBP1	A0A3Q1LXE1; bta:407130	ENSBTAG00000004283	-0.111832337	-0.165552248	0.053719912
E1BN47	CYFIP1	bta:100141021; E1BN47	ENSBTAG00000016894	0.037442284	0.053370289	-0.015928005
A0AAF6DM79	SAFB	A0AAF6DM79	ENSBTAG00000007875	-0.008445068	-0.080812046	0.072366977
F1N7G8	PDS5B	F1N7G8	ENSBTAG00000011597	-0.031148699	-0.100036001	0.068887303
A0AAA9T7D8	DPP3	A0AAA9T7D8	ENSBTAG00000022242	-0.032421478	-0.098042973	0.065621495
A0A3Q1MY58	PALM2	A0A3Q1MY58	ENSBTAG00000014471	0.10742088	0.277710982	-0.170290102
Q3ZBV8	TARS1	bta:510075; Q3ZBV8	ENSBTAG00000014261	0.351598962	0.398549376	-0.046950415
A0A3Q1N365	GOLGA4	A0A3Q1N365	ENSBTAG00000016563	0.019069963	0.094308691	-0.075238728
A0A3Q1MQ84	TRIP11	A0A3Q1MQ84	ENSBTAG00000026953	0.005018075	0.087758506	-0.082740431
F1N6D5	EFTUD2	F1N6D5	ENSBTAG00000013526	-0.142586585	-0.108181652	-0.034404934
E1BFV0	KPNB1	bta:507804; E1BFV0	ENSBTAG00000006586	0.142471789	0.098032083	0.044439706
A0AAA9U0Z2	MSH2	A0AAA9U0Z2	ENSBTAG00000002742	-0.140041482	-0.103736218	-0.036305264
A0A3Q1N1M4	ACACA	A0A3Q1N1M4	ENSBTAG00000017567	-0.171716947	-0.090151595	-0.081565352
A0A3Q1MR43	DDX46	A0A3Q1MR43	ENSBTAG00000021520	-0.219901697	-0.171625691	-0.048276006
A0A3Q1LIW3	AP2A2	A0A3Q1LIW3	ENSBTAG00000009915	0.091742569	0.144112308	-0.052369739
E1BGC5	YLPM1	bta:540849; E1BGC5	ENSBTAG00000008287	0.056131875	-0.010310269	0.066442143
A0A3Q1LWK1	EPB41L2	A0A3Q1LWK1	ENSBTAG00000005960	0.183752526	0.289845915	-0.10609339
A0AAA9SAT9	ITGA6	A0AAA9SAT9; bta:535043	ENSBTAG00000017266	-0.38545232	-0.361921604	-0.023530716
A0AAA9SHL0	DOCK7	A0AAA9SHL0	ENSBTAG00000019877	-0.018360537	-0.018360537	0
A0A3Q1M2R2	MIA3	A0A3Q1M2R2	ENSBTAG00000018824	0.133169981	-0.038252369	0.17142235
Q2KIZ8	MCM6	bta:517812; Q2KIZ8		-0.104480649	-0.046825856	-0.057654792
A0A3Q1M1Z2	LOC100141266	A0A3Q1M1Z2	ENSBTAG00000056851	0.041333392	-0.013163367	0.054496759
A0AAA9TNY2	LMNB1	A0AAA9TNY2	ENSBTAG00000002882	-0.156429159	-0.3063442	0.149915041
F1MC31	SF3B2	F1MC31	ENSBTAG00000012778	0.071805891	0.064334642	0.007471249
F1ME38	UBA6	F1ME38	ENSBTAG00000006837	-0.049421599	-0.049421599	0
P35605	COPB2	A4IFE1; bta:281706; P35605	ENSBTAG00000014843	0.02644667	-0.054393756	0.080840425
F1MCZ3	SCIN	F1MCZ3	ENSBTAG00000009786	-0.007699525	0.118796048	-0.126495573
E1BGE5	IPO7	bta:513707; E1BGE5	ENSBTAG00000027722	0.011997533	-0.031976608	0.043974141
E1BGH0	ABCF1	E1BGH0	ENSBTAG00000006927	-0.155950278	-0.169186105	0.013235827
F1MMA0	ILF3	bta:614936; F1MMA0	ENSBTAG00000040076	-0.134301092	-0.059501012	-0.07480008
A0A3Q1N716	KIAA1217	A0A3Q1N716	ENSBTAG00000018395	-0.077199632	0.101846759	-0.179046391
P11024	NNT	A4FUB4; bta:280878; P11024	ENSBTAG00000011885	0.012469352	0.019899557	-0.007430205
E1BMC4	GBF1	E1BMC4	ENSBTAG00000006014	0.063062268	0.001533966	0.061528302
A1A4J1	PFKL	A1A4J1; bta:508683	ENSBTAG00000010658	-0.091485163	-0.175336936	0.083851772
A0AAA9TW48	TRIP12	A0AAA9TW48	ENSBTAG00000021653	-0.179634212	-0.174859707	-0.004775505
A0AAA9SJ11	ACADVL	A0AAA9SJ11	ENSBTAG00000003072	-0.193210878	-0.161862934	-0.031347944
A0AAA9T4D7	CAPN1	A0AAA9T4D7	ENSBTAG00000010230	-0.186245773	-0.194553061	0.008307288
E1BNQ9	HNRNPUL2	bta:616476; E1BNQ9	ENSBTAG00000002510	0.056143078	0.073929017	-0.017785939
P48644	ALDH1A1	A7E3P6; bta:281615; P48644; Q3MHL5	ENSBTAG00000008103	-0.013151368	-0.172217854	0.159066486
P61286	PABPC1	bta:282296; P61286; Q3T0J7	ENSBTAG00000046358	0.03360353	-0.078988296	0.112591826
G3X6W2	RAD50	bta:788127; G3X6W2	ENSBTAG00000011252	-0.086269659	-0.040222291	-0.046047368
Q2KJ47	EHD2	bta:538348; Q2KJ47	ENSBTAG00000021191	0.112474729	0.012050423	0.100424306
Q3SZ57	AFP	bta:506011; Q3SZ57	ENSBTAG00000017131	0.034410031	0.618832702	-0.584422671
Q27971	CAPN2	A4IFD3; bta:281662; Q27971	ENSBTAG00000012778	0.091586393	0.035379287	0.056207105
A0A3Q1MFI5	DPYSL2	A0A3Q1MFI5	ENSBTAG00000018373	0.113433257	0.043589269	0.069843989
F1N3P2	USP5	F1N3P2	ENSBTAG00000019780	-0.205860697	-0.197036847	-0.00882385
A0A3Q1NAH5	SPAG9	A0A3Q1NAH5	ENSBTAG00000012293	-0.106812356	0.052515171	-0.159327528
A4FUC8	CARS1	A0A3Q1MBW4; A4FUC8; bta:515715; F1N5G7	ENSBTAG00000010777	-0.143280229	0.037474705	-0.180754935
E1BPX1	VPS13C	E1BPX1	ENSBTAG00000038920	-0.226199893	-0.164733939	-0.061465954
A0AAF6DLX9	HSD17B4	A0AAF6DLX9	ENSBTAG00000006978	0.029119604	-0.152151664	0.181271268
E1BJH1	EDC4	bta:513171; E1BJH1	ENSBTAG00000006417	0.091870052	-0.024271543	0.116141595
A0AAA9SJ67	LARP1	A0AAA9SJ67	ENSBTAG00000008443	0.005799787	-0.029353913	0.0351537
F1MF76	LOC515570	bta:515570; F1MF76	ENSBTAG00000017759	-0.051087104	-0.052573653	0.001486548
A0AAA9T6M2	ANXA2	A0AAA9T6M2	ENSBTAG00000009615	0.103237266	0.051871634	0.051365632
E1BAF6	PRRC2A	bta:538553; E1BAF6	ENSBTAG00000009682	0.001506731	0.076335345	-0.074828613
A0AAA9SZP1	MSH6	A0AAA9SZP1	ENSBTAG00000001424	-0.129813998	-0.132676489	0.002862491
A0A3Q1M1V8	TPM1	A0A3Q1M1V8	ENSBTAG00000005373	0.346650524	0.366881461	-0.020230937
E1BQ37	SFPQ	E1BQ37	ENSBTAG00000016328	0.049652301	0.007409852	0.042242449
E1BP91	NPEPPS	bta:534122; E1BP91	ENSBTAG00000011435	0.153167261	0.118721115	0.034446146
P81948	TUBA4A	bta:777775; P81948; Q17QT1; Q5E986		0.05195208	-0.08798892	0.139940999
A0A3Q1MFI2	FAM120A	A0A3Q1MFI2	ENSBTAG00000008696	-0.159154917	-0.200716908	0.041561991
A0A3Q1M384	SMARCA5	A0A3Q1M384	ENSBTAG00000003399	-0.170967788	-0.202607105	0.031639317
A0A3Q1MME4	HNRNPA2B1	A0A3Q1MME4	ENSBTAG00000005726	0.061400545	0.01218757	0.049212974
A0AAA9S896	LONP1	A0AAA9S896	ENSBTAG00000001795	0.111378992	0.34490242	-0.233523428
F1N693	CUX1	F1N693	ENSBTAG00000010659	-0.122029633	-0.199308808	0.077279175
F1N0E5	CCT4	F1N0E5	ENSBTAG00000004531	0.235907228	0.118867807	0.117039421
A0A3Q1LIF3	MCM5	A0A3Q1LIF3	ENSBTAG00000015595	-0.080016981	-0.063093678	-0.016923303
A0A3Q1N4S4	NUP205	A0A3Q1N4S4	ENSBTAG00000011127	0.060177996	0.049421599	0.010756397
F1MTG3	WASHC2A	bta:513740; F1MTG3	ENSBTAG00000018915	0.098042973	0.189844996	-0.091802023
F1N2K8	PPL	F1N2K8	ENSBTAG00000002178	-0.237440224	0.054170543	-0.291610767
G5E531	TCP1	G5E531	ENSBTAG00000002829	0.058198247	-0.041138695	0.099336942
E1B6X6	BAZ1B	bta:508442; E1B6X6	ENSBTAG00000005197	-0.206880667	-0.21658493	0.009704263
F1N4K1	PFAS	F1N4K1	ENSBTAG00000031509	-0.012060217	0.061335275	-0.049275058
Q3SZN2	SEC23B	bta:535071; Q3SZN2	ENSBTAG00000018295	-0.149576356	-0.248534836	0.09895848
A7MB16	EIF3B	A7MB16; bta:789999	ENSBTAG00000007474	-0.004308699	0.002865334	-0.007174033
A0A3Q1LUD9	DIAPH1	A0A3Q1LUD9	ENSBTAG00000030499	0.087628907	0.144927576	-0.057298669
Q0VCX4	CTNNB1	A7E3R2; bta:539003; Q0VCX4		-0.161308959	-0.233084758	0.071775798
Q2T9S0	TUBB3	bta:768070; Q2T9S0		0.063642495	-0.051387527	0.115030023
P62261	YWHAE	bta:282125; P29360; P42655; P62261; Q3ZC40; Q63631	ENSBTAG00000005664	0.342345485	0.225232373	0.117113112
A4FV12	UBA2	A4FV12; bta:507005	ENSBTAG00000012573	0.109151363	0.154090283	-0.04493892
F1N3R4	LETM1	F1N3R4	ENSBTAG00000020108	0.001495796	0.036937375	-0.035441579
A0AAF7AFD7	EHD1	A0AAF7AFD7	ENSBTAG00000050712	-0.034466404	-0.068347296	0.033880893
A0A3Q1M5W5	FAT1	A0A3Q1M5W5	ENSBTAG00000020657	-0.149234424	-0.029173136	-0.120061287
P21856	GDI1	A4FUY4; bta:281188; P21856	ENSBTAG00000012125	0.243017599	0.309127395	-0.066109796
A0AAA9TK27	MDN1	A0AAA9TK27	ENSBTAG00000016444	-0.074216107	-0.094474946	0.020258839
A0AAA9S9R4	PSMC3	A0AAA9S9R4	ENSBTAG00000021744	-0.037700709	-0.102298576	0.064597867
A0A3Q1MR81	MYO1B	A0A3Q1MR81	ENSBTAG00000011256	-0.221640038	0.162127311	-0.383767349
F1MHM5	YARS1	F1MHM5	ENSBTAG00000018065	0.309567948	0.618750814	-0.309182867
A0AAA9T3T4	UNC45A	A0AAA9T3T4	ENSBTAG00000018639	-0.041771738	-0.08653675	0.044765013
A4FUZ3	PSMC1	A4FUZ3; F1N550	ENSBTAG00000005426	-0.081498114	-0.122553182	0.041055068
A0A452DIS2	ITGB1	A0A452DIS2	ENSBTAG00000015910	-0.20142025	-0.258200299	0.05678005
P51176	TGM2	bta:281528; P51176; Q3ZBH7		0.031026896	0.128142096	-0.0971152
P68103	EEF1A1	A5PK01; bta:282220; P04719; P04720; P68103; Q2KJJ3	ENSBTAG00000014534	0.033326488	-0.077924472	0.111250959
A0AAA9T5M5	BOD1L1	A0AAA9T5M5	ENSBTAG00000004316	-0.05821196	-0.118845967	0.060024771
Q3MHH4	QARS1	bta:514586; Q3MHH4	ENSBTAG00000018928	-0.23570842	-0.268262921	0.0325545
A0A3S5ZPM3	PGD	A0A3S5ZPM3	ENSBTAG00000013527	0.024922415	-0.041080985	0.0660034
F1MM55	NUP133	F1MM55	ENSBTAG00000004965	-0.114787757	-0.101408728	-0.01337903
O02751	CFDP2	bta:286876; O02751; O02752; O18732; Q7JFY0; Q8I032		-0.102525716	-0.110028047	0.007502331
A0AAF6YIC2	HNRNPA1	A0AAF6YIC2	ENSBTAG00000001553	-0.017364643	-0.110571104	0.093206461
Q3SZ62	PGAM1	bta:404148; Q3SZ62	ENSBTAG00000012697	0.001547126	-0.073023256	0.074570382
A5PJZ5	NUP93	A5PJZ5; bta:510004	ENSBTAG00000006611	0.085196804	0.045466842	0.039729962
A0JN67	GLS	A0JN67; bta:525335		0.02075856	-0.224001674	0.244760234
A0A3Q1LG90	PALLD	A0A3Q1LG90	ENSBTAG00000001081	0.127659274	0.206072168	-0.078412893
A0AAA9RXX2	RBBP6	A0AAA9RXX2; bta:282034	ENSBTAG00000009441	0.10258577	0.057715498	0.044870272
A0AAA9SE56	PTK7	A0AAA9SE56	ENSBTAG00000012761	0.059518548	0.118278956	-0.058760409
A6H7I5	DNM2	A6H7I5; bta:511691		0.06877342	0.0142778	0.05449562
Q2KJC1	CDC5L	bta:767817; Q2KJC1	ENSBTAG00000020012	-0.017629894	-0.047500424	0.02987053
E1BNE5	RAB11FIP5	E1BNE5	ENSBTAG00000006162	0.109358756	0.051967092	0.057391664
A0A3Q1MA79	LAMA5	A0A3Q1MA79	ENSBTAG00000003061	-0.20268475	-0.031148699	-0.171536051
F1MJ56	MYO1D	F1MJ56	ENSBTAG00000015527	-0.385113863	0.139857664	-0.524971526
A0A3Q1LLF1	TNC	A0A3Q1LLF1	ENSBTAG00000000575	0.034080995	0.015246589	0.018834406
P10096	GAPDH	bta:281181; P10096; P79130; Q3ZCB1	ENSBTAG00000014731	-0.022414149	-0.059017255	0.036603106
A0A3S5ZP34	POR	A0A3S5ZP34	ENSBTAG00000017082	0.141019125	0.145591855	-0.00457273
F1MQ01	ATP2B4	F1MQ01	ENSBTAG00000014059	-0.025237291	-0.008363473	-0.016873819
A0A3Q1NBH6	GLS	A0A3Q1NBH6	ENSBTAG00000007863	0.101879614	0.096404484	0.00547513
A0A3Q1ME65	STAT1	A0A3Q1ME65	ENSBTAG00000007867	-0.068749139	-0.036258119	-0.03249102
A0A3Q1MDH5	ACAA2	A0A3Q1MDH5	ENSBTAG00000002863	0.068603814	0.040034662	0.028569152
A0A3Q1M6W0	NCOR2	A0A3Q1M6W0	ENSBTAG00000024603	-0.369573467	-0.311101064	-0.058472403
F1N7H0	DNAJC7	F1N7H0	ENSBTAG00000002654	0.257258667	0.076961982	0.180296686
A0A3Q1LQ12	EPB41L2	A0A3Q1LQ12	ENSBTAG00000005960	0.072265151	0.328535149	-0.256269999
E1BJ31	EPHA2	bta:512798; E1BJ31	ENSBTAG00000000815	0.266626392	0.3427395	-0.076113108
Q3MHK9	FSCN1	bta:507342; F1N089; Q3MHK9	ENSBTAG00000003191	-0.032453188	-0.093667615	0.061214426
Q0VCU1	ACO1	bta:512995; Q0VCU1		0.114458725	0.080090853	0.034367872
A0AAA9TEU1				-0.069670122	-0.14432107	0.074650949
P62739	ACTA2	bta:515610; P03996; P04108; P62739; Q3ZCA0; Q862W5	ENSBTAG00000014614	-0.001535599	0.039362432	-0.040898031
A7Z066	CANX	A7Z066; bta:407129	ENSBTAG00000048107	0.144095347	0.032112185	0.111983161
E1BIA7	CLUH	E1BIA7	ENSBTAG00000011786	-0.280211283	-0.235231535	-0.044979748
A0A3Q1M7S1	FBN1	A0A3Q1M7S1	ENSBTAG00000002278	-0.268851732	-0.082120694	-0.186731039
E1BCK1	RIF1	E1BCK1	ENSBTAG00000021020	-0.063036662	-0.088403382	0.02536672
F1MCT8	SYNCRIP	F1MCT8	ENSBTAG00000006672	0.286100016	0.33682231	-0.050722294
A0AAA9T461				0.114406307	0.254504606	-0.1400983
A0A452DJG1	ATP6V1A	A0A452DJG1; bta:282147	ENSBTAG00000002703	-0.047771449	-0.173899151	0.126127703
F6Q9Q9	P4HB	F6Q9Q9	ENSBTAG00000006045	0.029534195	-0.039321167	0.068855362
Q28006				-0.194973132	-0.344079662	0.14910653
F1MF50	ROCK1	F1MF50	ENSBTAG00000008403	-0.132537271	-0.127588055	-0.004949216
F1MUF1	XPO5	F1MUF1	ENSBTAG00000018970	0.24364468	0.355127788	-0.111483108
A0JN52	SF3B3	A0JN52; bta:504962	ENSBTAG00000010627	-0.083226907	-0.111031312	0.027804405
A0AAA9TFB6				0.008869055	-0.042125476	0.05099453
Q1RMU3	P4HA1	bta:518288; Q1RMU3	ENSBTAG00000032996	-0.175187117	-0.278996701	0.103809584
A0AAA9SJ37	SHMT2	A0AAA9SJ37	ENSBTAG00000031500	-0.189503547	-0.141765985	-0.047737562
A0A3Q1MK49	AKAP9	A0A3Q1MK49	ENSBTAG00000007442	-0.098239407	-0.054071823	-0.044167583
E1BJ59	ATXN2L	E1BJ59	ENSBTAG00000032087	0.143859019	0.192047812	-0.048188794
A0AAA9SQC6	ARHGEF2	A0AAA9SQC6	ENSBTAG00000000113	0.069286771	0.013820495	0.055466277
A0A140T871	GLUD1	A0A140T871; bta:281785	ENSBTAG00000007540	-0.040347286	-0.0555818	0.015234514
P62194	PSMC5	A6H7C5; bta:282015; O35051; P47210; P52915; P52916; P62194	ENSBTAG00000021061	-0.061400545	-0.076062206	0.014661661
F1N533	DDX18	bta:519254; F1N533	ENSBTAG00000015077	0.065588342	0.075753321	-0.010164979
A0A3Q1MJ74	PDLIM5	A0A3Q1MJ74	ENSBTAG00000012818	0.031364171	-0.071777202	0.103141373
A0A3Q1N9S5	HNRNPK	A0A3Q1N9S5	ENSBTAG00000021131	-0.032191286	-0.138082454	0.105891169
A0AAA9S3H4				0.200508294	0.370276898	-0.169768603
P15690	NDUFS1	bta:288380; P15690; Q0VCP7	ENSBTAG00000021976	-0.207064138	-0.093853527	-0.11321061
A2VDN6	SF3A1	A2VDN6; bta:504381	ENSBTAG00000003390	-0.050626073	0	-0.050626073
A0A3Q1MH83	GAPVD1	A0A3Q1MH83	ENSBTAG00000011544	0.043403124	0.001420675	0.041982449
A7E307	DDX17	A7E307; bta:514323		0.265550004	0.240194429	0.025355574
F1MSF1	RRM1	F1MSF1	ENSBTAG00000013111	-0.272538529	-0.489015623	0.216477093
Q08E34	TOMM70	bta:507707; Q08E34	ENSBTAG00000014302	0.017665874	-0.022390957	0.040056831
Q8MJG1	PSIP1	A7E383; bta:282011; Q0VCL7; Q8MJG1	ENSBTAG00000007872	0.12998149	0.103198888	0.026782602
A0A3Q1MJD6	TPX2	A0A3Q1MJD6	ENSBTAG00000018775	-0.087273821	-0.279459954	0.192186132
E1BCW3	PFKP	E1BCW3	ENSBTAG00000002683	0.01129317	-0.113470933	0.124764103
A0A3Q1M5A0	FXR1	A0A3Q1M5A0	ENSBTAG00000016457	0.096312673	-0.036680663	0.132993336
Q0V888	COPG	Q0V888		-0.332091424	-0.31410859	-0.017982834
A4IFM8	ACTA1	A4IFM8		0.078822272	0.018575729	0.060246542
A0A3Q1LNN6	KIF4A	A0A3Q1LNN6	ENSBTAG00000012861	-0.404934859	-0.210321426	-0.194613433
A0AAA9SNZ2	USP14	A0AAA9SNZ2	ENSBTAG00000019214	-0.051805388	-0.027771696	-0.024033692
A5D7C6	PREP	A5D7C6	ENSBTAG00000016281	-0.034661007	-0.039052777	0.004391769
A0AAA9SAU9	UBAP2L	A0AAA9SAU9	ENSBTAG00000004271	-0.062306151	-0.153656612	0.091350462
F6RN21	PABPC4	F6RN21	ENSBTAG00000017125	0.029659974	0.022653184	0.007006789
A0A3Q1M8Y4	SKIC3	A0A3Q1M8Y4	ENSBTAG00000009131	-0.049421599	-0.027247175	-0.022174424
Q2HJG5	VPS35	bta:521864; Q2HJG5	ENSBTAG00000002493	0.128200069	0.090374437	0.037825632
P12378	UGDH	A6QR10; bta:281564; O77806; P12378	ENSBTAG00000014521	0.053018382	-0.047048573	0.100066955
A0A3Q1MEH9	TJP2	A0A3Q1MEH9	ENSBTAG00000011770	-0.121034751	-0.110969395	-0.010065355
A7YWL9	GSPT1	A7YWL9		-0.005657635	-0.069401519	0.063743884
F1MVW5	SEC23A	F1MVW5	ENSBTAG00000003708	-0.009317761	-0.003099239	-0.006218523
P10881	SSB	bta:338071; P10881; Q3ZBL2	ENSBTAG00000008716	-0.199840579	-0.136929431	-0.062911148
A0AAF6Z672	TUFM	A0AAF6Z672	ENSBTAG00000019216	-0.070943252	-0.053596008	-0.017347244
E1BN24	ZNFX1	E1BN24	ENSBTAG00000020166	-0.108694436	-0.150342133	0.041647697
A7E3Q2	HSPA2	A7E3Q2	ENSBTAG00000007807	0.041080985	-0.067661708	0.108742693
Q3SYW6	EIF3C	bta:534882; Q3SYW6	ENSBTAG00000006543	-0.053519637	-0.160548203	0.107028565
A0AAA9U1R6	SWAP70	A0AAA9U1R6	ENSBTAG00000001987	0.129871192	0.209193645	-0.079322453
A7MBB7	NFKB2	A7MBB7; bta:526392; F6Q9X9		-0.169925001	-0.072595131	-0.097329871
Q5E9T9	RTCB	bta:525106; Q5E9T9	ENSBTAG00000011070	0.073421069	0.045622617	0.027798452
Q9GMB8	SARS1	bta:281476; Q9GMB8	ENSBTAG00000012962	0.260389685	0.286621226	-0.026231542

Accession	Gene_Symbol	Gene_ID	Ensembl_Gene ID	log2(ratio(OV24H/OV0H))	log2(ratio(OV24R/OV0H))	log2(ratio(OV24H/OV24R))
E1BDU0	NUP107	bta:504823; E1BDU0	ENSBTAG00000006911	0.036562906	0.023506753	0.013056153
E1B801	ARHGAP35	bta:540310; E1B801	ENSBTAG00000015543	-0.203248216	-0.178691112	-0.024557104
A6QL77	P4HA1	A6QL77; E1BLJ7	ENSBTAG00000032996	0.526068812	0.251379564	0.274689247
A0AAF6DLQ5	MTREX	A0AAF6DLQ5	ENSBTAG00000010518	-0.168256181	-0.068386975	-0.099869206
A0A3Q1MB56	SEPTIN9	A0A3Q1MB56; bta:100140583	ENSBTAG00000002633	0.009020901	0.047055486	-0.038034585
F1MFA3	PITRM1	F1MFA3	ENSBTAG00000002685	-0.219117289	-0.175509846	-0.043607443
A7MB83	PLOD2	A7MB83; bta:533642	ENSBTAG00000007331	0.096326117	0.042310805	0.054015312
E1BJG3	GEMIN5	E1BJG3	ENSBTAG00000012095	-0.0399585	-0.066243145	0.026284645
A0A3Q1M5H7	MRC2	A0A3Q1M5H7	ENSBTAG00000015739	-0.075655932	-0.156707735	0.081051803
A0A3Q1MWC7	ME1	A0A3Q1MWC7	ENSBTAG00000005681	-0.043253198	-0.015133304	0.058386501
A0A3Q1M326	SYNCRIP	A0A3Q1M326	ENSBTAG00000006672	0.057200556	-0.030963473	0.08816403
A0A3Q1NI32	THRAP3	A0A3Q1NI32; bta:505228	ENSBTAG00000024095	-0.229989099	-0.156615912	-0.073373186
A0A3Q1NNE9	COBLL1	A0A3Q1NNE9	ENSBTAG00000005501	-0.239391815	-0.061787316	-0.1776045
A7Z055	PLAA	A7Z055; bta:506073; F1MF89	ENSBTAG00000013011	-0.088894797	-0.028569152	-0.060325645
A0A3Q1MH62	PAPSS1	A0A3Q1MH62	ENSBTAG00000012961	-0.112190146	-0.287460448	0.175270302
A0A3Q1M2H6	CUL1	A0A3Q1M2H6; bta:407228	ENSBTAG00000008736	-0.057387482	-0.12882262	0.071435138
A0A3Q1M7L5	HSPA4L	A0A3Q1M7L5	ENSBTAG00000015692	0.367600942	0.517709846	-0.150108905
Q3SYZ5	DDX5	Q3SYZ5		-0.527356181	-0.627019239	0.099663058
A4IF88	IPO4	A4IF88; bta:510372; F1MGQ4		-0.237594828	-0.199885022	-0.037709806
A0AAF6YY96	ACAT1	A0AAF6YY96	ENSBTAG00000012885	-0.130035313	-0.226856209	0.096820896
A6H7B6	DDX24	A6H7B6; bta:529613	ENSBTAG00000003423	0.312719798	0.253692222	0.059027576
A0A3Q1LYQ6	NEXN	A0A3Q1LYQ6	ENSBTAG00000008921	0.137691143	0.156330873	-0.01863973
F1MTP5	WDR1	F1MTP5	ENSBTAG00000013607	0.017884878	-0.004506079	0.022390957
Q2KJG3	NARS	bta:616033; Q2KJG3		0.098722085	0.151850581	-0.053128495
A0AAA9RW37	NIBAN2	A0AAA9RW37	ENSBTAG00000012847	-0.216146512	-0.293397033	0.077250521
A0A3Q1ME79	XPO5	A0A3Q1ME79	ENSBTAG00000018970	0.07094008	0.089696943	-0.018756863
A0AAA9SZ98	NSUN2	A0AAA9SZ98; bta:516044	ENSBTAG00000015466	-0.32976052	-0.334891628	0.005131108
Q0VCR7	MTHFD1L	A6QNK9; bta:534296; Q0VCR7		-0.266335769	-0.258096483	-0.008239286
Q3T122	EIF3D	bta:515226; Q3T122	ENSBTAG00000001988	0.001471387	-0.102166889	0.103638276
A0A3Q1M8V2	UBR5	A0A3Q1M8V2	ENSBTAG00000021209	-0.520181003	-0.449240923	-0.07094008
A1L5B6	ANXA5	A1L5B6; bta:281626		0.142717043	0.070085315	0.072631728
F1MBW3	ACSL4	F1MBW3	ENSBTAG00000018986	-0.370299185	-0.373974843	0.003675658
A4IFB1	SRRT	A4IFB1; bta:527938		-0.023129875	-0.105592035	0.08246216
A0A3Q1LPZ9	CLASP1	A0A3Q1LPZ9	ENSBTAG00000019781	0.021651658	-0.093109404	0.114761062
F1MJB0	FILIP1L	F1MJB0	ENSBTAG00000013664	0.340787122	-0.101995368	0.44278249
Q2HJI1	SDHA	Q2HJI1		-0.051755154	0.006853671	-0.058608825
A0AAF6DLK2	IQGAP3	A0AAF6DLK2	ENSBTAG00000006882	-0.465876611	-0.406859356	-0.059017255
A0A3Q1MRG1	CUL3	A0A3Q1MRG1	ENSBTAG00000021769	0.09483134	0.078969466	0.015861874
A0AAA9SL11	ITGB3	A0AAA9SL11	ENSBTAG00000009987	-0.004898799	0.038602839	-0.043501639
Q3ZBU7	TUBB4A	bta:540236; Q3ZBU7		0.03280769	0.085881805	-0.053074116
Q2TBU9	RUVBL2	bta:511048; Q2TBU9		0.050867314	0.002795921	0.048071393
E1BDS6	TP53BP1	E1BDS6	ENSBTAG00000021304	0.015522272	0.052777031	-0.037254759
Q2KJH7	ALDH18A1	bta:514759; Q2KJH7	ENSBTAG00000011832	0.024453044	0.061850032	-0.037396989
E1B7F5	WDHD1	bta:540681; E1B7F5	ENSBTAG00000019120	-0.134697729	-0.165865747	0.031168018
A0A3Q1MDF7	GTF2I	A0A3Q1MDF7	ENSBTAG00000009780	-0.093109404	-0.224353938	0.131244533
F6QI68	PPP2R1A	F6QI68	ENSBTAG00000019851	0.115708883	0.037165761	0.078543122
A0A3Q1M4Y9	USP15	A0A3Q1M4Y9	ENSBTAG00000010428	-0.040034662	-0.016802356	-0.023232306
A1L505	UBAP2L	A1L505; bta:100125221		-0.041404686	-0.251464014	0.210059328
A0A3Q1M7L2	ANKHD1	A0A3Q1M7L2	ENSBTAG00000010871	-0.016022018	-0.125530882	0.109508864
A0AAA9S1Z4	SAFB2	A0AAA9S1Z4	ENSBTAG00000001790	-0.086177742	-0.057351707	-0.028826035
Q5E9D0	EIF2S2	bta:520094; Q0IIJ6; Q5E9D0		0.11872392	0.062029197	0.056694723
Q08DJ3	FUBP1	bta:513562; F1MX51; Q08DJ3		0.327361981	0.220446777	0.106915204
Q32LP6	STAT3	bta:508541; Q32LP6		0.109198771	0.031645627	0.077553144
F1MI56	ANKRD17	F1MI56	ENSBTAG00000004912	0.125030381	-0.031875674	0.156906055
A0A3Q1LQF0	PSMC6	A0A3Q1LQF0	ENSBTAG00000015732	-0.064325327	-0.070317838	0.005992511
A0AAF6DMH3	FUBP1	A0AAF6DMH3	ENSBTAG00000008926	0.116644919	0.006061753	0.110583166
A0AAA9STY1	GFPT1	A0AAA9STY1	ENSBTAG00000017626	0.148553731	0.091762979	0.056790753
E1BNG3	ascc3	bta:538416; E1BNG3	ENSBTAG00000020482	-0.130376094	-0.0716821	-0.058693994
A0AAA9SFG0	MYO1E	A0AAA9SFG0; bta:523485	ENSBTAG00000021538	0.00757322	-0.088728919	0.096302138
A0AAA9SUI7	PALLD	A0AAA9SUI7	ENSBTAG00000001081	0.100724846	0.176772167	-0.076047321
A0A3Q1NCG2	COBLL1	A0A3Q1NCG2	ENSBTAG00000005501	-0.002086327	0.112266232	-0.114352559
A0AAA9RWB8	TRIO	A0AAA9RWB8	ENSBTAG00000005514	-0.13983442	-0.203964758	0.064130337
A5PKI0	APPL1	A5PKI0; bta:517850	ENSBTAG00000006824	0.001698287	0.056648884	-0.054950597
A0AAA9U0H2	IDE	A0AAA9U0H2	ENSBTAG00000019759	-0.04493892	0.004016787	-0.048955707
A0AAA9ST00	PTPN11	A0AAA9ST00	ENSBTAG00000002048	0.084716959	0.136272147	-0.051555188
P00727	LAP3	bta:781648; P00727; Q2HJH5; Q2PC24	ENSBTAG00000005989	0.024714866	0.033874866	-0.009159999
G3MXU7	PHLDB1	G3MXU7	ENSBTAG00000016197	0.110012668	-0.103468699	0.213481367
E1BMZ5	EXOSC10	E1BMZ5	ENSBTAG00000002590	-0.224515593	-0.119372089	-0.105143504
F1MV07	SEC24D	F1MV07	ENSBTAG00000003619	-0.130113295	-0.139322018	0.009208723
A0AAA9TZ00	HNRNPA3	A0AAA9TZ00; bta:782669	ENSBTAG00000021580	0.061331843	0.030991814	0.030340029
Q3MHE8	SRPRA	bta:281504; Q3MHE8	ENSBTAG00000014105	0.072658609	-0.02859896	0.101257569
A0AAA9SB13	LDHA	A0AAA9SB13	ENSBTAG00000008683	0.038665109	-0.096015136	0.134680245
Q17R04	CCAR1	bta:767587; F1MMZ9; Q17R04	ENSBTAG00000033457	0.18781516	0.194572527	-0.006757366
A0AAA9SGU6	GOT2	A0AAA9SGU6	ENSBTAG00000007172	-0.051479219	0.025066761	-0.07654598
Q1JPJ8	THOP1	A6QQT3; bta:510889; Q1JPJ8		-0.090908496	-0.067638717	-0.023269779
A0AAA9S386	HADHB	A0AAA9S386	ENSBTAG00000010083	-0.268035087	-0.220729372	-0.047305715
O77588	PLOD1	bta:281409; O77588; Q1JPK0		0.234823376	0.124814371	0.110009005
F1N6K7	SEC16A	bta:519689; F1N6K7	ENSBTAG00000023933	-0.001413714	-0.028540892	0.027127178
A5PJZ1	SLC25A24	A5PJZ1; bta:534742	ENSBTAG00000003752	0.006472398	-0.024863387	0.031335785
Q08E58	TTLL12	bta:511319; F1N5K2; Q08E58	ENSBTAG00000001708	0.111568239	-0.01603821	0.127606439
F1MHN8	ALCAM	F1MHN8	ENSBTAG00000000888	0.004833154	-0.042926325	0.038093171
F1N405	RTN4	F1N405	ENSBTAG00000011104	0.064320981	0.32105081	-0.256729829
E1BF29	KIF11	bta:508467; E1BF29	ENSBTAG00000009383	-0.025036551	-0.044103283	0.019066732
A0A3Q1M070	RRP12	A0A3Q1M070	ENSBTAG00000002191	-0.35032369	-0.203996316	-0.146327374
A0A452DHZ5	NUCB1	A0A452DHZ5	ENSBTAG00000002378	-0.31709089	-0.096424181	-0.220666709
E1BIU0	SEC24C	E1BIU0	ENSBTAG00000002791	0.139055643	0.103361219	0.035694424
A0A3Q1ND98	ATP2B1	A0A3Q1ND98	ENSBTAG00000009552	0.077582941	0.005304032	0.072278909
Q6Q137	SEPTIN7	bta:408000; Q6Q137	ENSBTAG00000001182	-0.01763542	-0.005403359	-0.012232061
A0A3Q1MP70	GPI	A0A3Q1MP70	ENSBTAG00000006396	-0.028071757	-0.092134281	0.064062524
Q3SWY3	IMPDH2	bta:511969; Q3SWY3		-0.089565385	-0.054232312	-0.035333073
A0AAF7AMW6	ARCN1	A0AAF7AMW6	ENSBTAG00000006690	0.008951601	-0.094307159	0.10325876
Q3T030	PSMC4	bta:510029; Q3T030	ENSBTAG00000009414	-0.09172153	-0.106594807	0.014873276
A6QP73	DDX31	A6QP73; bta:507640		-0.064196712	-0.019108823	-0.04508789
A5D7J6	CALR	A5D7J6	ENSBTAG00000015114	0.179466096	0.080720616	0.09874548
E1BB38	SRP72	bta:509430; E1BB38	ENSBTAG00000010593	0.134195743	0.105108164	0.029087579
Q5E9F9	PSMC2	bta:530186; Q3T0H8; Q5E9F9	ENSBTAG00000046712	-0.031740571	-0.106585934	0.074845363
A0AAA9SIE1	INF2	A0AAA9SIE1	ENSBTAG00000007187	-0.196763798	-0.111351236	-0.085412562
A0A3Q1MCX8	SHTN1	A0A3Q1MCX8; bta:532603	ENSBTAG00000007578	0.045415458	0.146669649	-0.101254191
Q2TA49	VASP	bta:514902; Q2TA49	ENSBTAG00000019604	-0.03656016	-0.009389959	-0.027170201
Q3MHF7	MTAP	bta:782907; F1N1J3; Q3MHF7	ENSBTAG00000025929	0.039374238	-0.030252328	0.069626566
E1BCR2	DIDO1	E1BCR2	ENSBTAG00000006002	0.057374263	0.15721395	-0.099839687
E1BGF3	HCFC1	E1BGF3	ENSBTAG00000011904	-0.045043894	-0.013925736	-0.031118158
Q9TS87	TAGLN	bta:513463; Q2KJH8; Q9TS87	ENSBTAG00000007196	-0.01232495	-0.122311409	0.109986459
A0A3Q1LUN1	MPRIP	A0A3Q1LUN1	ENSBTAG00000010534	0.01232495	0.194019728	-0.181694778
A0A3Q1M334	STK10	A0A3Q1M334; bta:526376	ENSBTAG00000017457	-0.03170886	-0.028797264	-0.002911595
F1MPN0	FNDC3B	bta:615534; F1MPN0	ENSBTAG00000009376	0.172763803	0.129411958	0.043351845
A0AAA9RXM7	HLTF	A0AAA9RXM7	ENSBTAG00000019704	-0.349243848	-0.294796064	-0.054447784
F1MMK8	KARS1	F1MMK8	ENSBTAG00000015677	0.097652453	0.045339308	0.052313146
F1MKJ5	WASHC5	bta:508040; F1MKJ5	ENSBTAG00000010275	-0.116588778	-0.026916733	-0.089672045
A0A452DJ96	PGM1	A0A452DJ96	ENSBTAG00000019011	0.008395921	-0.042728544	0.051124465
E1BIU7	PTPN23	E1BIU7	ENSBTAG00000002774	-0.187446171	0.012962307	-0.200408478
Q3T102	EIF3E	bta:534165; Q1JP76; Q3T102		-0.038703529	-0.145730496	0.107026967
Q3MHL7	CCT6A	A5D9B9; bta:521540; Q3MHL7	ENSBTAG00000008184	0.161850879	0.04858018	0.113270699
F1MMJ2	NCAM1	F1MMJ2	ENSBTAG00000005710	-0.067878295	0.151147276	-0.21902557
Q3SYU7	TNPO1	bta:767620; Q3SYU7	ENSBTAG00000007979	0.321232653	0.190253089	0.130979563
P17248	WARS1	bta:281576; P17248; Q3SZK9	ENSBTAG00000004679	0.151582421	0.496798471	-0.34521605
F1MET0	PLOD3	bta:514996; F1MET0	ENSBTAG00000000342	0.201000127	0.132194612	0.068805515
Q17R07	ARFGAP3	bta:532778; Q17R07	ENSBTAG00000001057	0.096164397	0.023484431	0.072679966
Q3SYZ4	DARS1	bta:510162; Q3SYZ4		-0.08448787	0.028828653	-0.113316524
Q08DQ9	DLGAP5	bta:509161; Q08DQ9	ENSBTAG00000002331	0.085890421	-0.202218405	0.288108826
A0AAA9TSD9	AP1G1	A0AAA9TSD9	ENSBTAG00000002769	0.005782353	-0.018954326	0.024736678
A0AAF6YT68	AP3D1	A0AAF6YT68	ENSBTAG00000009034	-0.031650886	-0.010473331	-0.021177555
E1B9R8	SLFN11	bta:521795; E1B9R8	ENSBTAG00000019437	-0.14151072	-0.299722301	0.158211581
A0A3Q1NB02	SLTM	A0A3Q1NB02	ENSBTAG00000011319	-0.084670324	-0.190997225	0.106326901
A0A3Q1LRB7	KIF1B	A0A3Q1LRB7	ENSBTAG00000054239	0.202360233	-0.228905728	0.431265961
A0A3Q1MN33	GCLC	A0A3Q1MN33	ENSBTAG00000015571	0.041180636	-0.001492701	0.042673337
A0A3Q1LT42	AAK1	A0A3Q1LT42	ENSBTAG00000009343	0.078674314	-0.007110389	0.085784703
A0AAA9T569	LOC515333	A0AAA9T569	ENSBTAG00000032603	0.09661819	0.027834208	0.068783982
A6QQ11	PGM2	A6QQ11; bta:506980		0.150980579	0.212764651	-0.061784073
F1MYS7	EIF4B	F1MYS7	ENSBTAG00000006883	0.1313088	-0.001478929	0.132787729
Q17QG2	NUDC	bta:513277; Q17QG2		0.19592021	-0.064064099	0.259984309
A0AAA9SXS5	FERMT2	A0AAA9SXS5	ENSBTAG00000014501	-0.101573667	-0.113862455	0.012288788
A0AAA9S1S4	HNRNPR	A0AAA9S1S4	ENSBTAG00000016578	0.205800135	0.17875326	0.027046876
F1N4N6	SMARCC2	F1N4N6	ENSBTAG00000014697	0.19002913	0.200464582	-0.010435453
A0A3Q1MGY6	NCAM1	A0A3Q1MGY6	ENSBTAG00000005710	-0.30746816	0.075162725	-0.382630885
A4IFJ5	PLA2G4A	A4IFJ5; bta:525072; Q1XD55	ENSBTAG00000013298	-0.127185568	-0.270048008	0.14286244
O62768	TXNRD1	bta:282388; O62768	ENSBTAG00000013912	0.02486206	0.080329815	-0.055467755
E1BLB6	PRKDC	E1BLB6	ENSBTAG00000017019	-0.431103312	-0.298454627	-0.132648684
Q2KJ46	PSMD3	bta:504937; Q2KJ46	ENSBTAG00000021461	-0.11353612	-0.144389909	0.03085379
A3KN39	NCAPG	A3KN39; bta:531234		-0.182397166	-0.339310173	0.156913008
F1N6Z0	PSMD5	F1N6Z0	ENSBTAG00000016952	-0.074078144	-0.139125441	0.065047297
G3N2F2	P4HA2	G3N2F2	ENSBTAG00000001116	-0.226856209	-0.455259324	0.228403115
A0AAA9SB04	EIF2A	A0AAA9SB04	ENSBTAG00000009106	-0.157488633	-0.196169101	0.038680468
A0AAF7AIK6	ASNS	A0AAF7AIK6	ENSBTAG00000003222	0.377637527	0.700814169	-0.323176641
E1B9N7	DHX29	bta:512470; E1B9N7	ENSBTAG00000010510	-0.17412499	-0.32161747	0.147492481
F1MTY9	HMOX2	A0A8J8Y7Y8; bta:510243; F1MTY9; M5FHP2	ENSBTAG00000018937	0.29161247	0.10135267	0.1902598
E1BNV7	MYO9B	E1BNV7	ENSBTAG00000011125	-0.054133437	-0.088484942	0.034351505
A0AAF6YJJ1	PSMD12	A0AAF6YJJ1	ENSBTAG00000002423	0.005657635	0.030845967	-0.025188332
A0AAA9S255	ADAR	A0AAA9S255	ENSBTAG00000007519	0.016005858	0.024662054	-0.008656196
A0A3Q1MNQ1	TUT1	A0A3Q1MNQ1	ENSBTAG00000011846	-0.085960816	-0.131716079	0.045755263
Q3ZC41	ACAA1	bta:508324; Q3ZC41	ENSBTAG00000018283	0.013223695	-0.143760781	0.156984477
Q1RMR9	PACSIN2	bta:540107; Q1RMR9	ENSBTAG00000019290	0.174497731	0.180572246	-0.006074514
A5D7P3	PAFAH1B1	A5D7P3	ENSBTAG00000016806	0.09617254	0.078700241	0.017472299
Q5E954	DNAJA1	bta:528862; Q3SZU2; Q5E954	ENSBTAG00000016265	0.487027747	-0.106627729	0.593655475
A0AAA9TNL3	SLK	A0AAA9TNL3	ENSBTAG00000001017	-0.114828086	-0.074615818	-0.040212268
A0A3Q1NC75	CALU	A0A3Q1NC75	ENSBTAG00000016481	0.240280996	0.210644761	0.029636234
F1MAY4	CCDC88A	F1MAY4	ENSBTAG00000017469	-0.128997401	-0.193733658	0.064736257
A7E306	NID2	A7E306; bta:521854		0.154926837	-0.017319476	0.172246313
F1N3I3	UBE2O	bta:789422; F1N3I3	ENSBTAG00000020115	-0.093714435	-0.147976029	0.054261594
E1BGE2	UTP20	bta:510487; E1BGE2	ENSBTAG00000003239	-0.251104808	-0.135031707	-0.116073102
A0AAA9SJ92	RPS6KA1	A0AAA9SJ92	ENSBTAG00000014447	0.146963106	0.10196537	0.044997735
F1MJQ4	P4HA2	F1MJQ4	ENSBTAG00000001116	0.039075379	-0.218335631	0.257411011
F1MTX7	DARS1	F1MTX7	ENSBTAG00000009949	-0.085259088	-0.016378928	-0.068880161
A5PJD6	ATL3	A5PJD6; bta:515124		-0.068499937	0.139881375	-0.208381312
Q2YDF3	DDX19B	bta:517438; Q2YDF3		-0.064057314	-0.059027576	-0.005029738
Q148D3	FH	bta:520260; Q148D3	ENSBTAG00000021688	-0.049826685	0.011151322	-0.060978007
Q2TBI4	TRAP1	bta:514472; Q2TBI4	ENSBTAG00000020109	0.009993969	-0.067455347	0.077449316
F6PUM3	ZYX	F6PUM3	ENSBTAG00000017970	-0.018967505	-0.021356073	0.002388568
F1N3H1	CALU	F1N3H1	ENSBTAG00000016481	0.088398855	-0.089544457	0.177943312
F1MF97	NID2	F1MF97	ENSBTAG00000021945	-0.198050461	-0.39586242	0.197811959
Q2YDE9	TES	A4D7R3; bta:534965; Q2YDE9	ENSBTAG00000021204	0.259512351	0.192645078	0.066867273
A2VE14	SAE1	A2VE14; bta:505512	ENSBTAG00000002676	0.129684019	0.246038255	-0.116354236
Q3ZBH5	PA2G4	bta:540272; F1MLI9; Q3ZBH5	ENSBTAG00000010451	-0.123815867	-0.210085526	0.086269659
E1BCZ9	ADNP	bta:533757; E1BCZ9	ENSBTAG00000000340	-0.27677102	-0.428289906	0.151518886
P15497	APOA1	A5D7M9; bta:281631; P15497; Q3SZD9		0.538840455	0.651179331	-0.112338876
F6RAC6	SRP68	bta:538777; F6RAC6	ENSBTAG00000009228	0.079659449	0.073685548	0.005973901
E1BHM5	RBM25	E1BHM5	ENSBTAG00000017177	-0.069594474	-0.186462973	0.116868498
E1BKT3	ERBIN	E1BKT3	ENSBTAG00000000415	-0.186970754	-0.320374304	0.13340355
A0AAA9S692	AP3B1	A0AAA9S692	ENSBTAG00000005016	0.075634438	0.034992453	0.040641984
G3MWG4	IARS2	G3MWG4	ENSBTAG00000012582	-0.040721103	-0.078233288	0.037512184
Q2KJI7	AFG3L2	bta:515757; Q2KJI7		0.01052518	0.050417907	-0.039892727
A0AAA9T2V7	KIF15	A0AAA9T2V7	ENSBTAG00000016726	-0.12945693	-0.198021216	0.068564286
A0A3Q1M302	KNL1	A0A3Q1M302	ENSBTAG00000053398	-0.081683798	-0.198328716	0.116644919
A0A3Q1M5R4	LDHB	A0A3Q1M5R4	ENSBTAG00000019603	0.065304039	0.011326418	0.053977621
A7MB01	SART3	A7MB01; bta:505922		0.035477895	0.120533665	-0.08505577
F1MKX7	XRN2	bta:517417; F1MKX7	ENSBTAG00000011304	-0.69234882	-0.457289986	-0.235058833
Q2NL22	EIF4A3	bta:515145; Q2NL22	ENSBTAG00000016023	0.043845959	0.030286695	0.013559264
A0A3Q1MF35	PRPF40A	A0A3Q1MF35	ENSBTAG00000048151	-0.005961558	-0.042257687	0.036296129
A0A3Q1MM65				-0.041113881	-0.125394412	0.084280531

Accession	Gene_Symbol	Gene_ID	Ensembl_Gene ID	log2(ratio(OV24H/OV0H))	log2(ratio(OV24R/OV0H))	log2(ratio(OV24H/OV24R))
P61157	ACTR3	A5PJ90; bta:281597; P32391; P61157; Q08DS8		0.018505469	-0.027480736	0.045986205
A7Z089	NUP160	A7Z089; bta:540111		0.029029049	0.148920881	-0.119891832
P68102	EIF2S1	bta:327694; P05199; P68102; Q3SWZ1	ENSBTAG00000016311	0.033705937	-0.016402911	0.050108848
P33097	GOT1	bta:281206; P33097; Q2KJF2; Q5E9R4	ENSBTAG00000011960	0.086414752	0.074162491	0.012252261
F6RPT3	RAP1GDS1	F6RPT3	ENSBTAG00000007522	0.047064562	-0.065762192	0.112826754
A0AAA9RSD3	PRKCSH	A0AAA9RSD3	ENSBTAG00000008202	0.052669069	0.037619482	0.015049587
A0AAF6DLW2	FARSB	A0AAF6DLW2	ENSBTAG00000011649	-0.026800059	-0.055639742	0.028839683
A0AAA9SL80	RPAP3	A0AAA9SL80	ENSBTAG00000018690	0.113520864	0.143606591	-0.030085726
F2Z4E7	ILF2	F2Z4E7	ENSBTAG00000012471	-0.032061209	-0.066359451	0.034298242
A0A3Q1M2B9	ARID1A	A0A3Q1M2B9	ENSBTAG00000001024	-0.102795569	-0.164571767	0.061776198
A0A3Q1M0G8	PAK2	A0A3Q1M0G8; bta:513673	ENSBTAG00000047856	0.017364643	-0.019050592	0.036415236
Q0IIM6	USP8	bta:538743; F1N3U9; Q0IIM6	ENSBTAG00000011916	-0.227068909	-0.183469882	-0.043599027
F1MCA8	TMPO	F1MCA8	ENSBTAG00000004240	-0.045638082	-0.101003319	0.055365237
A0A3Q1MMR9	PPP6R3	A0A3Q1MMR9	ENSBTAG00000009584	-0.048859401	-0.124604763	0.075745362
Q08D98	PPP2R5D	bta:100134966; Q08D98		0.067114196	0.034661007	0.032453188
Q2KI84	HARS1	bta:510937; Q2KI84		0.075090917	-0.029383807	0.104474723
A0AAF6YW85	CNN1	A0AAF6YW85	ENSBTAG00000011207	0.134895457	0.113859434	0.021036023
F1N032	DENND4C	bta:536760; F1N032	ENSBTAG00000003097	-0.162075902	-0.103549825	-0.058526077
A0AAA9SWC2	TBCD	A0AAA9SWC2	ENSBTAG00000015414	-0.103193185	-0.084521909	-0.018671277
Q3MHR7	ARPC2	bta:540838; Q3MHR7	ENSBTAG00000010386	-0.040438544	-0.031677212	-0.008761333
A0A3Q1M9F3	NUP155	A0A3Q1M9F3	ENSBTAG00000002458	0.01439827	0.002891173	0.0115071
A0AAF6YQK3	COPS4	A0AAF6YQK3	ENSBTAG00000006950	0.094804366	0.022437389	0.072366977
E1BP72	CDC42BPB	E1BP72	ENSBTAG00000016456	-0.104441612	-0.051275902	-0.05316571
A0JBZ9	p97bcnt2	A0JBZ9; bta:514131	ENSBTAG00000030600	0.120785803	0.148773006	-0.027987202
A0AAA9SSX1	RNH1	A0AAA9SSX1	ENSBTAG00000046869	0.317134615	0.007020428	0.310114188
E1BH06				0.029975059	0.306220339	-0.27624528
G3N3P0	BICD2	bta:282447; G3N3P0	ENSBTAG00000046549	0.134531957	0.033740212	0.100791745
F1MQU4	BCLAF1	F1MQU4	ENSBTAG00000007802	-0.079012503	-0.131644006	0.052631502
A0AAA9TYH2	MRE11	A0AAA9TYH2	ENSBTAG00000008925	-0.086633309	-0.032823847	-0.053809462
A0AAA9T7E3	RAB3GAP1	A0AAA9T7E3	ENSBTAG00000013449	-0.038434723	-0.017611959	-0.020822764
A0A3Q1NC33	TCERG1	A0A3Q1NC33	ENSBTAG00000003259	-0.084509524	-0.094872693	0.01036317
A0AAF6YHR8	ANXA4	A0AAF6YHR8	ENSBTAG00000011105	-0.108047173	-0.09469441	-0.013352763
A0A3Q1MJD9	ANXA3	A0A3Q1MJD9	ENSBTAG00000010153	-0.004249473	-0.027847985	0.023598512
F1MUP9	VAT1	F1MUP9	ENSBTAG00000007390	-0.066314254	0.00359625	-0.069910504
Q8SPJ1	JUP	bta:445543; Q17QY6; Q8SPJ1	ENSBTAG00000017685	-0.282594216	-0.015675069	-0.266919147
A2VE51	BAG3	A2VE51; bta:782633		0.738751108	0.406076632	0.332674476
A0AAA9TGB2	NF1	A0AAA9TGB2	ENSBTAG00000050242	-0.284501599	-0.306034761	0.021533162
E1BMM0	NCBP1	bta:100124428; E1BMM0	ENSBTAG00000009732	0.121379239	0.070604672	0.050774567
A0A3Q1LTM9	GOLGA2	A0A3Q1LTM9; bta:508805	ENSBTAG00000011317	-0.107929559	-0.07979377	-0.02813579
Q9XSK7	HDGF	bta:327953; Q9XSK7	ENSBTAG00000039793	0.071462363	0.06608919	0.005373172
A0A3Q1MT69	XPO7	A0A3Q1MT69	ENSBTAG00000047628	0.170248802	0.19592021	-0.025671408
Q2KI42	PSMD11	A1L5A1; bta:513461; F1MXM5; Q2KI42		-0.055438986	-0.011495639	-0.043943348
A0AAA9TG38	SEC63	A0AAA9TG38	ENSBTAG00000016562	0.059572445	0.036313699	0.023258745
A0AAA9SW45	U2SURP	A0AAA9SW45; bta:505019	ENSBTAG00000009154	0.001449217	-0.065251185	0.066700401
F1N7U2	NPLOC4	F1N7U2	ENSBTAG00000019105	-0.080789747	-0.052417249	-0.028372498
A0AAA9TZH9	KIF1B	A0AAA9TZH9	ENSBTAG00000054239	-0.017802823	-0.130985312	0.11318249
A0A3Q1MD15	ANKFY1	A0A3Q1MD15	ENSBTAG00000021091	-0.108148804	0.002669187	-0.110817991
Q28156	PDE6A	bta:281972; Q28156	ENSBTAG00000024888	-0.190556334	-0.281885732	0.091329398
Q0P5L6	ZW10	A0A8J8YS76; bta:511084; F1N3Z5; Q0P5L6; V6F7Q3		-0.167686529	-0.141092319	-0.02659421
A0AAF6YKW6	RPL3	A0AAF6YKW6	ENSBTAG00000003228	-0.102381904	-0.153376435	0.05099453
Q95L54	ANXA8	bta:281627; Q95L54	ENSBTAG00000018499	-0.156211834	-0.184178985	0.027967151
Q148J8	IDH3A	F1MN74; Q148J8	ENSBTAG00000006227	-0.083571368	-0.149588541	0.066017173
A6QL85	RCC2	A6QL85; bta:509120; F1MZE9	ENSBTAG00000008579	0.085916064	0.287493261	-0.201577197
A0AAA9TW40	CDC37	A0AAA9TW40	ENSBTAG00000011699	0.12936035	0.042670616	0.086689734
Q2HJ89	FKBP10	bta:535310; Q2HJ89	ENSBTAG00000011454	-0.045442971	-0.128584206	0.083141235
A6H742	PLS1	A6H742; bta:616560	ENSBTAG00000023429	0.117155391	0.062144521	0.055010869
A0A3Q1MY28	DYNC1I2	A0A3Q1MY28	ENSBTAG00000002646	-0.056583528	-0.084888898	0.028305369
A0A3S5ZPK0	OGT	A0A3S5ZPK0	ENSBTAG00000000902	0.101356472	-0.024662054	0.126018526
E1BP90	ARFGEF1	E1BP90	ENSBTAG00000014691	-0.001420675	-0.041778412	0.040357738
A0AAA9TIX2	SMARCA4	A0AAA9TIX2	ENSBTAG00000019220	-0.132647251	-0.1382718	0.005624549
E1B726	PLG	E1B726	ENSBTAG00000001271	-0.292436798	0.295084127	-0.587520925
E1BKY9	CPOX	bta:540283; E1BKY9	ENSBTAG00000004124	0.029918711	0.029918711	0
A0AAF6YL22	DDX19A	A0AAF6YL22	ENSBTAG00000003570	-0.011416044	-0.050626073	0.039210029
F1MUT3	XDH	F1MKA4; F1MUT3	ENSBTAG00000012519	-0.27462238	-0.216575095	-0.058047285
E1BKW5	SEC23IP	E1BKW5	ENSBTAG00000007441	0.080087911	0.071405668	0.008682243
E1BMN2	DIAPH3	E1BMN2	ENSBTAG00000012443	-0.074926002	0.017471136	-0.092397138
E1BI26	MDC1	E1BI26	ENSBTAG00000025526	0.183471533	0.137812816	0.045658717
A0AAF6YZK2	EIF3H	A0AAF6YZK2	ENSBTAG00000014032	-0.093642847	-0.215298639	0.121655792
O77834	PRDX6	bta:282438; O77834; Q5E9F3	ENSBTAG00000004855	0.165016678	0.123692217	0.041324461
Q5E9A3	PCBP1	bta:509917; Q5E9A3	ENSBTAG00000008985	-0.038556411	-0.068564286	0.030007875
A0A3Q1LKM4	ANKLE2	A0A3Q1LKM4	ENSBTAG00000033339	0.056754778	-0.057533764	0.114288542
P00432	CAT	bta:531682; P00432; Q3SZ80	ENSBTAG00000020980	0.051050402	-0.037402734	0.088453136
Q07130	UGP2	bta:281565; Q07130; Q17QU0	ENSBTAG00000000111	0.130060541	0.03167405	0.09838649
P80724	BASP1	bta:286842; O02692; P80724; Q08E19	ENSBTAG00000048565	0.175299713	0.245112498	-0.069812784
A0AAA9TRF6	RAB11FIP5	A0AAA9TRF6	ENSBTAG00000006162	-0.158539046	-0.277575337	0.119036291
A7MBG8	RUVBL1	A7MBG8; bta:511475; F1MBF3	ENSBTAG00000020998	0.039183263	0.023916506	0.015266757
E1BP36	MMS19	A6QPX1; bta:533499; E1BP36; F1MUW1	ENSBTAG00000012704	-0.014312568	0.058608825	-0.072921393
E1BNH2	CTH	E1BNH2	ENSBTAG00000014791	0.174404792	0.537697574	-0.363292782
A0AAA9SX99	PAPSS2	A0AAA9SX99	ENSBTAG00000003196	-0.005047335	-0.005047335	0
G3MXG4	ARHGEF40	bta:527362; G3MXG4	ENSBTAG00000013401	-0.092589608	-0.154894267	0.062304659
P31408	ATP6V1B2	A4FUX5; bta:338082; P31408; Q28058	ENSBTAG00000018646	-0.002793215	-0.060120992	0.062914207
P37980	PPA1	bta:280701; P37980; Q1RMH6	ENSBTAG00000007836	0.135795518	0.074704545	0.061090973
F1N3G2	NOP2	F1N3G2	ENSBTAG00000014733	-0.013838907	-0.097937936	0.084099029
E1BI98	COL6A1	bta:511422; E1BI98	ENSBTAG00000011802	-0.574595785	-0.374010231	-0.200585554
Q3MHL4	AHCY	A5D988; bta:508158; Q3MHL4	ENSBTAG00000018101	-0.01137479	0.037281016	-0.048655806
A6H768	GALK1	A6H768; bta:530855; Q1JP66		-0.142482283	-0.123334442	-0.019147842
F1N719	PPP5C	bta:510245; F1N719	ENSBTAG00000008014	-0.034500873	0.006185197	-0.040686071
A0AAA9SCC8	SLC25A12	A0AAA9SCC8	ENSBTAG00000016762	-0.215816668	-0.189142917	-0.026673751
A6QQR4	ERCC6L	A6QQR4; bta:782916; Q0V8L7	ENSBTAG00000005607	0.091003199	-0.016040011	0.10704321
A0A3Q1M6R3	ELP1	A0A3Q1M6R3	ENSBTAG00000004991	-0.119255557	-0.107011961	-0.012243596
F1N650	ANXA1	F1N650	ENSBTAG00000015978	-0.014855605	-0.140804236	0.125948631
Q3MHN5	GC	bta:530076; Q3MHN5		-0.359431738	0.198315265	-0.557747003
Q24JZ4	MTDH	bta:509788; Q24JZ4	ENSBTAG00000003098	0.001398638	0.074663568	-0.07326493
A5PK07	WDR44	A5PK07		0.008536682	-0.091303005	0.099839687
A5D7C4	TNPO3	A5D7C4; bta:537183	ENSBTAG00000004992	0.134604466	0.037435807	0.097168659
F1MW01	ESPL1	bta:506740; F1MW01	ENSBTAG00000008934	-0.047965325	0.009102207	-0.057067532
Q2NKY7	SEPTIN2	bta:511612; Q2NKY7	ENSBTAG00000002608	-0.024112456	-0.00132906	-0.022783396
E1BGC3	WAPL	bta:534322; E1BGC3	ENSBTAG00000012213	0.145180254	0.190983944	-0.04580369
F1N5M2	GC	F1N5M2	ENSBTAG00000013718	0.008046284	0.63153924	-0.623492955
E1BFB4	MTOR	E1BFB4	ENSBTAG00000015325	-0.101913416	0	-0.101913416
E1BCF8	ZC3HAV1	bta:614589; E1BCF8	ENSBTAG00000021617	-0.145197916	-0.240881264	0.095683348
A0A3Q1MF39	NBAS	A0A3Q1MF39	ENSBTAG00000014081	-0.024295587	0.054237815	-0.078533403
A0AAA9T8S7	NFKB1	A0AAA9T8S7	ENSBTAG00000020270	0.203275602	0.023594384	0.179681218
A0A3Q1MR04	NSF	A0A3Q1MR04; bta:504457	ENSBTAG00000008442	0.044142555	0.102520904	-0.058378349
F1N1Z2	USP47	F1N1Z2	ENSBTAG00000010919	-0.245997252	-0.2098046	-0.036192652
Q5E947	PRDX1	A6QLL7; Q5E947	ENSBTAG00000003642	-0.065972032	-0.112231558	0.046259525
A7MBC2	PDXDC1	A7MBC2; bta:505868		-0.038122209	-0.117528643	0.079406434
A0AAF6ZF08	MAPRE1	A0AAF6ZF08	ENSBTAG00000003908	0.112724927	-0.005730673	0.1184556
A0AAA9SKH3	GIGYF2	A0AAA9SKH3	ENSBTAG00000014079	-0.174066282	-0.267933205	0.093866923
A0A3Q1MJF5	SLC25A13	A0A3Q1MJF5	ENSBTAG00000003773	-0.007557351	0.047451596	-0.055008947
G5E567	IPO9	G5E567	ENSBTAG00000004452	-0.236402624	-0.247756101	0.011353477
A0AAA9T365	HNRNPL	A0AAA9T365	ENSBTAG00000001055	0.03391106	0.061075577	-0.027164517
A6QQS8	NOP2	A6QQS8; bta:541169		-0.015786486	0.003484772	-0.019271259
F1N1M7	CPT2	F1N1M7	ENSBTAG00000014649	-0.130060541	-0.091888038	-0.038172502
E1BLR9	CPD	E1BLR9	ENSBTAG00000012371	0.138731291	0.022344717	0.116386575
A0AAA9THK8	POLA1	A0AAA9THK8	ENSBTAG00000008170	-0.225389631	-0.299985542	0.074595911
Q3ZBX5	AIMP1	bta:505126; F6PWM7; Q3ZBX5	ENSBTAG00000004230	0.089580593	0.077755158	0.011825435
E1BBH1	PHLDB2	E1BBH1	ENSBTAG00000001209	0.353936331	0.153379711	0.20055662
A1L5B7	SERBP1	A1L5B7; F1MFK6		-0.203616196	-0.520832163	0.317215968
A0A3Q1MG97	LNPEP	A0A3Q1MG97	ENSBTAG00000019900	-0.085148119	0.168022964	-0.253171083
A5D9H5	HNRNPD	A5D9H5; bta:527471	ENSBTAG00000013952	-0.22881869	-0.277728291	0.0489096
A0AAA9T5D6	SMARCA1	A0AAA9T5D6	ENSBTAG00000002292	-0.486722467	-0.314178595	-0.172543872
A0AAF6DM04	SERBP1	A0AAF6DM04	ENSBTAG00000012957	-0.09172153	-0.242902809	0.151181279
A0AAA9T5N1	EEF1D	A0AAA9T5N1	ENSBTAG00000014643	0.069239083	0.037242518	0.031996564
A0AAA9S435	PRPF6	A0AAA9S435	ENSBTAG00000011656	-0.262764516	-0.089066352	-0.173698165
Q2KJA2	ABCF2	bta:513061; Q1JPK5; Q2KJA2		0.039052777	-0.13355033	0.172603107
E1BJQ1	GLDC	bta:507688; E1BJQ1	ENSBTAG00000025903	-0.100017294	-0.031808738	-0.068208556
E1B7Q0	HOOK3	bta:524648; E1B7Q0	ENSBTAG00000007634	0.064560886	0.069288454	-0.004727569
A0AAA9SA75	SYTL2	A0AAA9SA75	ENSBTAG00000000460	-0.063193826	0.139064038	-0.202257864
F1N7T2	MAN2A1	F1N7T2	ENSBTAG00000025029	0.054447784	-0.011427347	0.065875131
F1MXE4	PSMD6	F1MXE4	ENSBTAG00000015112	-0.082071133	-0.07365071	-0.008420423
A1L5A7	DBN1	A1L5A7	ENSBTAG00000011400; ENSBTAG00000069499	-0.111031312	-0.099242115	-0.011789198
A0A3Q1MMI6	EPS15L1	A0A3Q1MMI6	ENSBTAG00000009990	0.048273111	0.083446651	-0.03517354
A0A3Q1MTV4	GPD2	A0A3Q1MTV4	ENSBTAG00000009770	0.093880811	0.217344498	-0.123463687
F1N0F7	EIF5	bta:504752; F1N0F7	ENSBTAG00000016282	0.122233292	-0.018079122	0.140312414
E1BJV0	EHD4	bta:505206; E1BJV0	ENSBTAG00000006792	-0.061298222	-0.151218807	0.089920585
Q3ZC50	CCDC47	A5D9E2; bta:540167; Q3ZC50	ENSBTAG00000011834	0.046518379	0.053882841	-0.007364462
A0AAA9T9F6	CUL4B	A0AAA9T9F6	ENSBTAG00000018569	0.04476987	-0.027825261	0.072595131
A6QLD1	EXOC4	A6QLD1; bta:537690; F1MNJ7	ENSBTAG00000014112	0.155701365	0.128859753	0.026841612
F1MMD7	ITIH4	F1MMD7	ENSBTAG00000007850	0.073581132	0.3016557	-0.228074567
A5PJY9	CPNE3	A5PJY9; bta:615304; E1B819	ENSBTAG00000015536	0.029429477	-0.031488642	0.060918119
F1MYA8	ATP13A1	bta:534369; F1MYA8	ENSBTAG00000007128	-0.054393756	-0.104863543	0.050469788
Q3SZH7	LTA4H	bta:507130; Q3SZH7	ENSBTAG00000016415	0.278888357	0.310082979	-0.031194622
A0A3Q1LY87	ENAH	A0A3Q1LY87	ENSBTAG00000016185	0.1627295	0.102499363	0.060230137
A0A3Q1MN56	PHLDB2	A0A3Q1MN56	ENSBTAG00000001209	0.100994661	-0.159320244	0.260314904
A0AAA9U1C1	TFRC	A0AAA9U1C1	ENSBTAG00000032719	-0.462884259	-0.476367488	0.013483229
A0A3Q1MR38	PRMT1	A0A3Q1MR38	ENSBTAG00000006646	-0.074115808	-0.093839329	0.01972352
A0AAA9RVW3	UGGT2	A0AAA9RVW3	ENSBTAG00000004401	-0.226068079	-0.084221601	-0.141846478
A0AAA9SSF0	SCFD1	A0AAA9SSF0; bta:100140328	ENSBTAG00000017565	0.106915204	0.155092572	-0.048177368
F1N331	HMMR	F1N331	ENSBTAG00000014773	0.423452322	0.288860393	0.134591928
A0A3Q1MJ08	NEDD4L	A0A3Q1MJ08	ENSBTAG00000005412	0.109439605	0.083444397	0.025995209
E1B864	ITGB4	E1B864	ENSBTAG00000018169	-0.59286542	-0.321098721	-0.271766698
A0AAA9TH40	AIFM1	A0AAA9TH40	ENSBTAG00000006838	0.085560348	0.088326782	-0.002766434
A5PKK6	AHCYL1	A5PKK6; bta:505511		-0.225174862	-0.137384396	-0.087790466
A0AAA9TRR7	HIP1R	A0AAA9TRR7	ENSBTAG00000021595	-0.022089542	0.01091922	-0.033008762
Q2HJ33	OLA1	bta:509966; Q2HJ33	ENSBTAG00000006970	0.110456648	0.177832083	-0.067375435
Q9XSA7	CLIC4	bta:286823; Q3ZBJ4; Q9XSA7	ENSBTAG00000009470	0.113310195	0.127874823	-0.014564628
F1MZ21	SART1	bta:615704; F1MZ21	ENSBTAG00000003094	0.022672468	-0.052749565	0.075422034
G3N2L2	RCN1	G3N2L2	ENSBTAG00000047362	0.265922685	0.302719041	-0.036796356
A0A3Q1MUN6	SEPTIN11	A0A3Q1MUN6	ENSBTAG00000021372	-0.08210108	-0.065302829	-0.016798251
A0A3Q1MNL1	CUL4A	A0A3Q1MNL1	ENSBTAG00000020093	-0.162176978	-0.187553541	0.025376563
E1B8H0	PEAK1	E1B8H0	ENSBTAG00000005145	0.045118546	0.416033492	-0.370914946
Q0VCV0	RPA1	A0A3Q1MBP4; bta:504844; Q0VCV0	ENSBTAG00000009711	0.127629887	0.128986441	-0.001356554
A0AAA9SYH6	CACYBP	A0AAA9SYH6	ENSBTAG00000001107	0.514765263	0.295953843	0.21881142
F1N153				-0.161681007	-0.195995372	0.034314365
A0A140T8A5	IDH1	A0A140T8A5; bta:281235	ENSBTAG00000020527	0.097635003	-0.006489867	0.10412487
A0A3Q1LW48	EMC1	A0A3Q1LW48	ENSBTAG00000011950	-0.263747024	-0.268030139	0.004283116
A5PKM3	SPTLC2	A5PKM3; bta:537972	ENSBTAG00000020480	-0.121066634	-0.157173504	0.03610687
A0A3Q1LVN5	DDX42	A0A3Q1LVN5	ENSBTAG00000021058	-0.019496176	-0.052108853	0.032612677
Q0VCX5	ETF1	bta:541077; Q0VCX5	ENSBTAG00000011415	0.052142306	0.101051906	-0.0489096
F1MD37	PPFIA1	F1MD37	ENSBTAG00000022188	0.079538765	0.212764651	-0.133225886
A0A3Q1NN09	RBM10	A0A3Q1NN09	ENSBTAG00000002890	-0.191879113	-0.196481015	0.004601902
Q3T0G3	AHSA1	bta:539220; Q3T0G3	ENSBTAG00000020477	0.428236997	0.225420114	0.202816883
A0AAA9SY77	ZC3H4	A0AAA9SY77	ENSBTAG00000009286	0.129495694	0.058357604	0.07113809
A0A3Q1LMN6	PXDN	A0A3Q1LMN6; bta:515860	ENSBTAG00000050986	0.108490787	0.205366319	-0.096875532
A0A3Q1M4B7	ESYT2	A0A3Q1M4B7	ENSBTAG00000005483	-0.140105319	-0.190452404	0.050347085
Q29RL9	TCEA1	bta:505722; Q29RL9		0.074079761	0.14597268	-0.071892918
A0AAA9RW91	KIF23	A0AAA9RW91	ENSBTAG00000009983	-0.077696921	-0.205076227	0.127379306
A0A3Q1MRT9	LRRC40	A0A3Q1MRT9; bta:526961	ENSBTAG00000008243	-0.056244111	-0.019938847	-0.036305264
Q8WN55	PTBP1	A0A3Q1M994; bta:282018; Q8WN55	ENSBTAG00000045828	0.105825473	0.09158355	0.014241923
Q24JZ7	OXCT1	bta:535804; F1MTP3; Q24JZ7	ENSBTAG00000033186	-0.034518941	-0.005062093	-0.029456847
A0AAA9S704	ACOX1	A0AAA9S704	ENSBTAG00000030174	-0.246535025	-0.285247432	0.038712406
A0A3Q1M7N2	DHX30	A0A3Q1M7N2	ENSBTAG00000015833	-0.371458076	-0.33971433	-0.031743746
A0A3Q1N6T1	CYB5R3	A0A3Q1N6T1	ENSBTAG00000016516	0.119082648	0.117688066	0.001394582
A0AAA9TSW3	CTPS1	A0AAA9TSW3	ENSBTAG00000001626	0.24346396	0.109195785	0.134268176
A6H7H6	CDH17	A6H7H6; bta:507526	ENSBTAG00000021964	-0.376922127	-0.401719833	0.024797706
A0A3Q1M507	CPT1A	A0A3Q1M507; bta:506812	ENSBTAG00000021999	-0.025066761	0.023280824	-0.048347584
Q5E9E2	MYL9	bta:531505; Q5E9E2	ENSBTAG00000016024	0.005418579	-0.079511292	0.084929871
A0AAA9SRV2	ATXN2	A0AAA9SRV2	ENSBTAG00000022069	-0.128424955	-0.001446311	-0.126978644

Accession	Gene_Symbol	Gene_ID	Ensembl_Gene ID	log2(ratio(OV24H/OV0H))	log2(ratio(OV24R/OV0H))	log2(ratio(OV24H/OV24R))
Q2T9U1	SRP54	bta:614017; Q2T9U1	ENSBTAG00000019085	0.039057476	-0.019129938	0.058187414
F1MSJ6	TBC1D8B	F1MSJ6	ENSBTAG00000046314	-0.140428791	-0.173004394	0.032575604
A0A3Q1M2P8	EPS8	A0A3Q1M2P8	ENSBTAG00000000369	-0.08305574	-0.03058832	-0.05246742
A0A3Q1M7H7	DNPEP	A0A3Q1M7H7	ENSBTAG00000021514	-0.112001518	-0.027195811	-0.084805707
P28339	POLD1	bta:281990; P28339		-0.206236653	-0.240375255	0.034138602
A0A3Q1MMW5				-0.131441771	-0.204328486	0.072886715
A4FUY3	LOC539976	A4FUY3; bta:539976		-0.099535674	-0.13262646	0.033090786
E1BJG5	TRIM28	E1BJG5	ENSBTAG00000006422	-0.032358242	-0.009771557	-0.022586685
P07857	SCP2	bta:508918; P07857; Q3SYV0		-0.082757885	-0.273123223	0.190365338
A0AAA9TCP2	DCTN2	A0AAA9TCP2	ENSBTAG00000010624	0.019590728	-0.088608012	0.108198741
F1MHP6	ADSL	F1MHP6	ENSBTAG00000000077	-0.02181222	-0.007752276	-0.014059944
F1N2Y2	COL5A2	F1N2Y2	ENSBTAG00000027020	-0.408730095	-0.401004802	-0.007725293
E1BMC6	SLMAP	E1BMC6	ENSBTAG00000021164	-0.218740027	-0.061639066	-0.157100961
F1MG56	PPP2R2A	F1MG56	ENSBTAG00000018317	0.126944596	0.135397912	-0.008453315
A0AAA9TXG2	COL6A2	A0AAA9TXG2	ENSBTAG00000019269	-0.518369287	-0.60745283	0.089083544
A0AAF7AGM7	TAGLN2	A0AAF7AGM7	ENSBTAG00000002068	0.033819998	0.066865305	-0.033045307
A0AAA9T8H1	SUGP2	A0AAA9T8H1	ENSBTAG00000013288	0.681157377	0.448852212	0.232305165
F1MS08	SLC12A2	F1MS08	ENSBTAG00000009637	-0.458493755	-0.485093295	0.02659954
A0AAA9T5C9	MAPK1	A0AAA9T5C9	ENSBTAG00000010312	0.034899985	0.073011127	-0.038111142
A0AAA9SUI2	APEX1	A0AAA9SUI2	ENSBTAG00000002745	0.090765464	0.13606155	-0.045296086
Q3ZC44	HNRNPAB	bta:513410; Q3ZC44	ENSBTAG00000013627	0.010514222	-0.075826627	0.086340849
A0AAA9TGR1	GFM1	A0AAA9TGR1	ENSBTAG00000027795	-0.445850621	-0.416021267	-0.029829354
A0AAA9SQ91	PSIP1	A0AAA9SQ91	ENSBTAG00000007872	-0.032214625	0.272861552	-0.305076177
P63103	YWHAZ	bta:287022; P29213; P29312; P63103; Q3ZCF9	ENSBTAG00000000236	0.15038778	0.08685839	0.06352939
A5D7S0	NAPA	A5D7S0; bta:518407		0.132607495	0.01337903	0.119228465
A0AAA9RSP8	ASPH	A0AAA9RSP8	ENSBTAG00000026283	0.041042233	-0.008028374	0.049070607
A0A3Q1N7W0	PRPF3	A0A3Q1N7W0	ENSBTAG00000013946	-0.13664418	-0.127225028	-0.009419152
A0AAA9T6Y7	MON2	A0AAA9T6Y7	ENSBTAG00000011068	-0.104652152	-0.070389328	-0.034262825
Q04467	IDH2	bta:327669; Q04467; Q3ZCD4	ENSBTAG00000014093	-0.041469539	-0.052025891	0.010556352
Q59A30	son	bta:516462; Q59A30		-0.116554257	-0.129058735	0.012504477
F1MU12				-0.698638975	0.106486124	-0.805125099
E1BKC4	NIPBL	bta:539666; E1BKC4	ENSBTAG00000000579	-0.102988313	-0.096900984	-0.00608733
F6QZ79				-0.028428408	-0.053040963	0.024612554
A0AAA9SV11	RPN2	A0AAA9SV11	ENSBTAG00000014648	-0.394725929	-0.34191957	-0.052806359
A0AAA9STQ6	G3BP1	A0AAA9STQ6	ENSBTAG00000020309	-0.216200588	-0.239558276	0.023357688
F1ME46	LMAN1	F1ME46	ENSBTAG00000006833	-0.093793077	-0.040098069	-0.053695008
A0AAA9TIZ3	NAV1	A0AAA9TIZ3	ENSBTAG00000021919	-0.093596719	-0.215587243	0.121990524
A0A3Q1M166	HSPA12A	A0A3Q1M166; bta:540050	ENSBTAG00000013779	0.142138693	0.132853415	0.009285278
F1MJP7	UAP1	F1MJP7	ENSBTAG00000010206	0.100447906	-0.134768556	0.235216462
Q1RMR3	CTTN	bta:506939; Q1RMR3	ENSBTAG00000006071	0.007061664	-0.077058957	0.084120621
A0AAA9TPR5	DPP9	A0AAA9TPR5	ENSBTAG00000011134	-0.174476955	-0.151679945	-0.02279701
E1BNS3	BRD4	E1BNS3	ENSBTAG00000002495	0.02795723	-0.028509722	0.056466952
F6PTU3	SUN2	F6PTU3	ENSBTAG00000003693	-0.309893995	-0.210460753	-0.099433242
F6R6W2	DDX23	F6R6W2	ENSBTAG00000021810	0.001463922	-0.013242577	0.014706499
Q08DZ4	SMTN	bta:528013; F1N031; Q08DZ4		-0.45132832	-0.384524949	-0.066803371
F1MSZ6	SERPINC1	F1MSZ6	ENSBTAG00000004362	-0.070320695	0.119799981	-0.190120677
A0AAA9T691	PPP4R1	A0AAA9T691	ENSBTAG00000009822	-0.217189845	-0.220899721	0.002980776
A1L5C2	ADFP	A1L5C2		0.291025815	0.731701151	-0.440675336
A0AAA9SEC9	SPECC1	A0AAA9SEC9	ENSBTAG00000000928	0.009151698	0.253164162	-0.244012463
A0A3Q1N369	CD2AP	A0A3Q1N369	ENSBTAG00000000322	0.150535652	0.027732292	0.12280336
Q2HJ94	DNAJA2	bta:360006; Q2HJ94	ENSBTAG00000003757	0.17807124	-0.0364002	0.21447144
A5PK45	COLGALT1	A5PK45; bta:513167	ENSBTAG00000012678	0.21818017	0.060541542	0.157638628
A7Z035	CLINT1	A7Z035; bta:538540	ENSBTAG00000016199	0.12388129	0.085671341	0.038209949
A0AAA9S063	MAP4K4	A0AAA9S063	ENSBTAG00000013023	-0.082944425	-0.094567409	0.011622984
Q3SYU6	CNN2	bta:539019; Q3SYU6	ENSBTAG00000020764	0.011266222	-0.00882385	0.020090073
P68509	YWHAH	bta:282126; P11576; P68509; P70198; Q3ZC14	ENSBTAG00000031134	0.016945892	0.039737757	-0.022791865
F6Q8A8	NUCB2	F6Q8A8	ENSBTAG00000017468	-0.230204625	-0.243501448	0.013296823
A7YWC4	ATAD3	A7YWC4; bta:784353	ENSBTAG00000002219	-0.042030251	-0.06127634	0.019246089
F1MLI5	OSBPL3	F1MLI5	ENSBTAG00000019342	-0.060058413	-0.09105198	0.030993567
A0A3Q1MXM6	NEDD4	A0A3Q1MXM6; bta:507781	ENSBTAG00000013793	0.05714137	0.006119606	0.05099453
Q2KJ42	NONO	bta:615175; Q2KJ42	ENSBTAG00000012152	-0.157593478	-0.148538877	-0.009054601
A0AAF6Z3R0	YBX1	A0AAF6Z3R0	ENSBTAG00000017368	0.021000299	-0.139055643	0.160055942
Q2KIK0	SUGT1	bta:515509; Q2KIK0		0.247175522	0.188845863	0.058329659
G3MYQ1	FHOD1	bta:787862; G3MYQ1	ENSBTAG00000032427	-0.25307002	-0.268759394	0.015689374
A4IFE6	ABCE1	A4IFE6; bta:514991	ENSBTAG00000019854	-0.086364675	-0.131566499	0.045201824
A0JN97	PRKCD	A0JN97; bta:505708		-0.019504962	-0.037950629	0.018445667
A0AAF6ZQF2	TUBG1	A0AAF6ZQF2	ENSBTAG00000048152	-0.082989365	0.031026896	-0.11401626
E1BIX2	POLR1A	bta:536316; E1BIX2	ENSBTAG00000006481	0.094728288	0.066132169	0.028596119
A6H7G2	DBNL	A6H7G2; bta:514706		-0.07474058	-0.036231017	-0.038509563
A0A3Q1LKM6	TFG	A0A3Q1LKM6	ENSBTAG00000016363	0.138944058	0.11279177	0.026152288
A0AAA9TYA8	ITSN1	A0AAA9TYA8	ENSBTAG00000021997	0.05564106	0.079019999	-0.023378939
A6QLA8	TRIM25	A6QLA8; bta:510923; F1MTX8	ENSBTAG00000009948	0.190589664	0.174664118	0.015925546
A0A3Q1MUZ8	SH3KBP1	A0A3Q1MUZ8	ENSBTAG00000003727	0.04301735	-0.019341722	0.062359073
A0AAA9TV55	GNE	A0AAA9TV55	ENSBTAG00000001142	0.084008937	0.014587536	0.069421401
A0AAF6ZAI1	CNDP2	A0AAF6ZAI1	ENSBTAG00000009841	0.003520201	0.045105964	-0.041585763
Q29RT6	KIF20A	bta:527854; Q29RT6		-0.285541805	-0.354298817	0.068757012
Q0V7M0	KPNA6	bta:531602; Q0V7M0	ENSBTAG00000002896	0.08590905	0.023108263	0.062800787
F1MG91	LOC281376	bta:281376; F1MG91	ENSBTAG00000023026	-0.136178126	0.023672829	-0.159850955
Q3SYV6	KPNA2	bta:513592; Q3SYV6	ENSBTAG00000012225	-0.001530711	-0.357552005	0.356021294
A6QNL5	PDIA6	A6QNL5; bta:613927		0.306846911	0.186307394	0.120539517
P63243	RACK1	bta:327682; P25388; P63243; P99049; Q3T0R8		-0.069751108	-0.115122616	0.045371508
F1MDS3	PCK2	bta:282856; F1MDS3	ENSBTAG00000011934	0.034047481	0.613860002	-0.579812521
E1BBQ0	KNTC1	bta:506353; E1BBQ0	ENSBTAG00000019278	-0.579642169	-0.412670485	-0.166971684
A0AAA9S5L9	BAG6	A0AAA9S5L9	ENSBTAG00000019685	-0.364065681	-0.374201754	0.010136072
A0AAF6Z7A1	UBE4A	A0AAF6Z7A1	ENSBTAG00000019725	-0.001495796	-0.025641883	0.024146087
F1MBP8	COPS5	bta:507179; F1MBP8	ENSBTAG00000002784	-0.039335894	-0.033650389	-0.005685505
Q1RMW3	MTA2	bta:515389; Q1RMW3		-0.112400743	-0.130267164	0.017866421
A0A3Q1N814	FAM114A2	A0A3Q1N814	ENSBTAG00000017078	0.25276607	0.232282597	0.020483473
A0A3Q1NF03	RANGAP1	A0A3Q1NF03	ENSBTAG00000012481	-0.111031312	-0.129370903	0.018339591
E1B8Q8	XPOT	E1B8Q8	ENSBTAG00000026962	0.11594148	0.331554119	-0.215612639
A0A3Q1M0B4	PTPN12	A0A3Q1M0B4; bta:531877	ENSBTAG00000003825	-0.119052672	-0.119052672	0
A0A3Q1LVH9	SH3PXD2B	A0A3Q1LVH9	ENSBTAG00000019019	-0.147933429	-0.191946605	0.044013176
Q2PQT6				0.075597619	0.052304357	0.023293261
Q28104	COPE	A1L5B2; bta:338085; Q28104; Q2TBL7; Q9N286		0.010536161	-0.183394933	0.193931094
A0AAA9T7E7	PNPT1	A0AAA9T7E7	ENSBTAG00000008909	-0.036144564	-0.054562197	0.018417634
Q2KHZ2	HBS1L	bta:541083; Q2KHZ2		0.130909778	0.192837527	-0.061927749
A6QNS2	UBE4B	A0A3Q1LPU1; A6QNS2; bta:520833	ENSBTAG00000010401	0.054133437	-0.043068722	0.097202159
F1MYU1	SMARCC1	F1MYU1	ENSBTAG00000031567	-0.245486205	-0.517819644	0.272333439
A0AAF6YZS2	PRKAR2A	A0AAF6YZS2	ENSBTAG00000014205	0.153222616	0.158697746	-0.00547513
A0A3Q1LGY1	PUF60	A0A3Q1LGY1	ENSBTAG00000017493	0.125277221	-0.007629288	0.13290651
Q1LZF9	MYL9	bta:505964; F1MLA6; Q1LZF9	ENSBTAG00000011473	-0.292397791	-0.194503024	-0.097894766
F1MMD5	XRCC6	F1MMD5	ENSBTAG00000006103	0.002911595	0.069695224	-0.066783629
G1K1X0	UQCRC1	G1K1X0	ENSBTAG00000019096	-0.047557253	-0.032044253	-0.015512999
A0A3Q1MTX3	ADD1	A0A3Q1MTX3	ENSBTAG00000021128	-0.060695932	0.025440293	-0.086136225
E1BGB2	PRRC2B	E1BGB2	ENSBTAG00000004400	-0.283624207	-0.206948616	-0.076675591
A6QLU8	NXN	A6QLU8; bta:531367	ENSBTAG00000000855	0.156342029	0.051225323	0.105116706
E1BB16	GCC2	bta:539484; E1BB16	ENSBTAG00000016038	-0.160063288	-0.098853387	-0.061209901
A0AAA9TK37	ACTR1A	A0AAA9TK37	ENSBTAG00000021067	-0.009886348	-0.024128271	0.014241923
A6H7E3	PDLIM1	A6H7E3	ENSBTAG00000011182	-0.007819504	0.023207391	-0.031026896
E1BEI0	API5	E1BEI0	ENSBTAG00000014474	0.247298359	0.179567583	0.067730776
A0A3Q1MN26	FOCAD	A0A3Q1MN26	ENSBTAG00000038181	-0.091029876	-0.180481769	0.089451893
A0A3Q1M1M5	TMEM43	A0A3Q1M1M5	ENSBTAG00000007360	0.011216344	0.053874241	-0.042657897
Q3SZI4	YWHAQ	A7E3X8; bta:768311; Q3SZI4	ENSBTAG00000002108	0.224303909	0.168808796	0.055495113
A6H7H2	KLC1	A6H7H2; bta:508999	ENSBTAG00000017299	-0.080335734	-0.142867517	0.062531783
F1MH07	MICAL1	A2VDL7; bta:508306; F1MH07		-0.203395706	-0.196870616	-0.00652509
F1MBA3	ITGA2	bta:281872; F1MBA3	ENSBTAG00000019289	-0.322855575	-0.273830007	-0.049025568
F1MJM0	ZNF326	bta:533643; F1MJM0; Q0VCF6		0.144315396	-0.036445635	0.180761031
F1N3S4	CUL4A	F1N3S4	ENSBTAG00000020093	-0.076395819	-0.03769229	-0.038703529
Q08DU9	HP1BP3	bta:510194; Q08DU9	ENSBTAG00000018656	-0.186717293	-0.061784073	-0.124933221
G5E5X0	LIMD1	bta:617525; G5E5X0	ENSBTAG00000026097	0.16949052	0.160190278	0.009300242
A0AAF6YVA6	EIF3L	A0AAF6YVA6	ENSBTAG00000010790	-0.037684476	-0.203694427	0.166009951
Q2HJD8	RBM39	bta:508722; Q2HJD8		0.289203339	0.098312061	0.190891278
A0AAA9RXM0	UFL1	A0AAA9RXM0	ENSBTAG00000020410	0.156725504	0.294229027	-0.137503524
A0A3Q1MQ76	SUPT5H	A0A3Q1MQ76	ENSBTAG00000010467	0.030936167	-0.065423075	0.096359242
Q3B7N3	IPO5	Q3B7N3	ENSBTAG00000039374	0.201633861	0.121736208	0.079897653
F1MTS8	LTN1	F1MTS8	ENSBTAG00000000201	0.02200391	0.011777168	0.010226742
A0A3Q1N7P8	ITPR2	A0A3Q1N7P8	ENSBTAG00000002313	0.14577114	0.274732927	-0.128961787
A5D7E1	EHD3	A5D7E1; bta:510183	ENSBTAG00000001662	0.104990104	0.143664811	-0.038674707
Q56JV9	RPS3A	A2V9E4; B0JYM2; bta:282053; Q56JV9	ENSBTAG00000009908	-0.127513423	-0.154750008	0.027236584
A0A3Q1LPP0	KIF1C	A0A3Q1LPP0	ENSBTAG00000001349	-0.002714385	0.054548219	-0.057262603
A0A452DJI0	MICAL3	A0A452DJI0	ENSBTAG00000048271	-0.008007579	0.028986629	-0.036994207
G3MZY8	POLR2A	bta:282312; G3MZY8	ENSBTAG00000046765	-0.169050694	0.031830534	-0.200881228
F1MHC2	STXBP2	F1MHC2	ENSBTAG00000009178	0.04096032	0.225050637	-0.184090317
Q3T149	HSPB1	Q3T149	ENSBTAG00000011969	0.609646764	0.508269744	0.10137702
A0A452DIX3	TPI1	A0A452DIX3	ENSBTAG00000019782	0.138458213	0.141635957	-0.003177744
A0A3Q1M7R3	ATXN2	A0A3Q1M7R3	ENSBTAG00000022069	-0.352631842	-0.093454176	-0.259177667
A0AAA9TDA2	NUFIP2	A0AAA9TDA2; bta:781847	ENSBTAG00000033077	0.204630982	0.134728969	0.069902013
A0A3Q1M1T4	CKAP2	A0A3Q1M1T4	ENSBTAG00000021162	0.285908872	-0.01626098	0.302169852
Q58DN4	PPME1	bta:535390; Q58DN4	ENSBTAG00000027612	-0.023132966	-0.093211401	0.070078436
E1BMF9	LLGL1	E1BMF9	ENSBTAG00000004635	-0.193325434	-0.264463524	0.07113809
A4IFC8	LIG1	A4IFC8; bta:100124507; F1MSC6		0.059501012	-0.118644496	0.178145508
A0AAA9TKS4	NSFL1C	A0AAA9TKS4	ENSBTAG00000006533	0.03099791	0.004081177	0.026916733
E1B7Q6	MED1	bta:520734; E1B7Q6	ENSBTAG00000013236	-0.174100677	-0.10371135	-0.070389328
Q5E964	PSMD13	bta:513525; Q3T0Z1; Q5E964		-0.034404934	-0.061914783	0.027509849
A0A3Q1MT26	PARD3	A0A3Q1MT26	ENSBTAG00000014991	-0.10562785	-0.157594942	0.051967092
A0AAA9RX06	LYAR	A0AAA9RX06	ENSBTAG00000003653	0.243056704	0.205028802	0.038027901
P08166	AK2	bta:280716; P08166; P08167; Q2KIJ7	ENSBTAG00000017605	0.085729874	0.040902187	0.044827687
A0A3Q1LPW5	KIF22	A0A3Q1LPW5	ENSBTAG00000013669	-0.086898222	-0.172562815	0.085664592
F1MPE9	NOTCH2	F1MPE9	ENSBTAG00000007909	0.272843929	0.278419569	-0.00557564
F1MRE5	ACBD3	bta:783375; F1MRE5	ENSBTAG00000013128	0.095617266	0.054562197	0.041055068
A0A3Q1MNI4	SSRP1	A0A3Q1MNI4	ENSBTAG00000000375	-0.043501639	-0.030474958	-0.01302668
A0A452DJ13	NDUFV1	A0A452DJ13	ENSBTAG00000021776	-0.127132987	-0.082367974	-0.044765013
A0A3Q1MH34	DCTN4	A0A3Q1MH34	ENSBTAG00000015625	0.011205455	-0.069124361	0.080329815
F6RQC9	GNAI2	F6RQC9	ENSBTAG00000020645	0.028515371	-0.001372035	0.029887407
O02691	HSD17B10	bta:281809; O02691; Q2TBG6		0.074704545	0.009358055	0.06534649
A0AAA9SQ31	TXNL1	A0AAA9SQ31	ENSBTAG00000015731	-0.031154523	0.134431543	-0.165586066
F1MI47	RBM14	F1MI47	ENSBTAG00000001225	-0.010185484	-0.119348927	0.109163443
Q3T147	DDX39B	bta:540191; Q3T147		-0.043515308	-0.043515308	0
A7MBD4	FARSA	A7MBD4; bta:522024	ENSBTAG00000021374	-0.035697369	-0.056936165	0.021238796
A0AAA9RW75	ITIH2	A0AAA9RW75	ENSBTAG00000014075	-0.078855421	0.430683863	-0.509539283
G3MXV0	FKBP5	bta:535704; G3MXV0	ENSBTAG00000047502	0.295573833	0.351558442	-0.05598461
F1MPG7	NAT10	F1MPG7	ENSBTAG00000016747	0.024146087	0.139162748	-0.11501666
F6RV75	TRIOBP	F6RV75	ENSBTAG00000002539	0.110070778	0.181737987	-0.07166721
A0AAA9TT27	ATRX	A0AAA9TT27	ENSBTAG00000038434	-0.277419834	-0.230998033	-0.046421801
E1BHK2	ADD1	E1BHK2	ENSBTAG00000021128	-0.227773301	-0.044045572	-0.183727729
F6Q5B6	EIF2AK2	F6Q5B6	ENSBTAG00000008703	0.0957112	0.025809186	0.069902013
F1MYQ3	RIPOR1	F1MYQ3	ENSBTAG00000008860	-0.117379697	-0.097141158	-0.020238539
Q8SQH5	SLC25A5	bta:282479; Q3SZD0; Q8SQH5		-0.052520483	-0.157265791	0.104745308
P11180	DLAT	bta:512723; F1N690; P11180; V6F7T1	ENSBTAG00000010709	0.00434329	0.010114078	-0.005770788
A0A3Q1MTE4	ERO1A	A0A3Q1MTE4	ENSBTAG00000015716	0.010159866	0.006781188	0.003378678
F1MZN7	SNX2	F1MZN7	ENSBTAG00000025358	-0.01926586	0.04778052	-0.067046379
A0AAF6ZJJ6	ITGA3	A0AAF6ZJJ6	ENSBTAG00000019498	-0.19891561	-0.173246889	-0.025668721
Q2KJC8	FKBP9	bta:534182; Q2KJC8	ENSBTAG00000016707	-0.063325935	-0.143458537	0.080132602
A0A3Q1LY89	MYEF2	A0A3Q1LY89	ENSBTAG00000008771	-0.229621726	-0.076350886	-0.15327084
A0AAA9S2J2	AGO2	A0AAA9S2J2	ENSBTAG00000001579	-0.057030945	-0.049777561	-0.007253384
A0A3Q1LW57	FNDC3A	A0A3Q1LW57	ENSBTAG00000020681	0.197856987	0.14071308	0.057143907
Q2HJ58	PRPS1	A5PKB5; bta:781227; Q2HJ58		-0.014047774	-0.0775366	0.063488826
A6QR01	LRRC47	A6QR01; bta:616209; F1MKE3		-0.025995209	0.037396989	-0.063392197
A0AAA9SJ86	CUX1	A0AAA9SJ86	ENSBTAG00000010659	-0.147487612	-0.162594505	0.015106892
A0AAA9TGU4	FMNL3	A0AAA9TGU4	ENSBTAG00000016593	-0.008127881	0.043116398	-0.051244279
F1N7G0	HNRNPLL	F1N7G0	ENSBTAG00000020567	0.018634174	0.034215715	-0.015581541
A0A452DI31	ENO3	A0A452DI31	ENSBTAG00000005534	-0.047545584	0.140444133	-0.187989717
A0AAA9S8L6	STAU1	A0AAA9S8L6	ENSBTAG00000000241	0.065712841	-0.024010163	0.089723004
Q2KHU8	EIF2S3	A5D972; bta:512350; Q2KHU8	ENSBTAG00000014337	-0.04717312	-0.052663879	0.005490758
A0A3Q1MS21	VDAC1	A0A3Q1MS21	ENSBTAG00000013113	0.056918429	0.048522508	0.008395921
A0AAA9SVE9	TMX3	A0AAA9SVE9	ENSBTAG00000007567	0.208416402	0.208416402	0
Q08DH4	KLC2	bta:518136; Q08DH4		0	-0.008788017	0.008788017
A0AAA9S1F3	GPS1	A0AAA9S1F3	ENSBTAG00000019317	-0.27822081	-0.257004324	-0.021216486
A0AAA9TR62	AGRN	A0AAA9TR62	ENSBTAG00000013191	-0.193925766	-0.281068329	0.087142563

Accession	Gene_Symbol	Gene_ID	Ensembl_Gene ID	log2(ratio(OV24H/OV0H))	log2(ratio(OV24R/OV0H))	log2(ratio(OV24H/OV24R))
A0A3Q1MJM3	TOR1AIP1	A0A3Q1MJM3	ENSBTAG00000035226	0.135731924	0.149303944	-0.01357202
F1MYG0	OAT	F1MYG0	ENSBTAG00000006928	0.001718517	-0.131326419	0.133044936
F1MK73	YAP1	F1MK73	ENSBTAG00000039307	0.43171624	0.257686841	0.1740294
A0AAA9T8F7	ALDH1L2	A0AAA9T8F7	ENSBTAG00000006326	0.220390068	1.008976355	-0.788586286
P79136	CAPZB	bta:338052; P79136; Q3T012	ENSBTAG00000004554	0.158730415	0.167134488	-0.008404073
A0AAF6YU44	USP10	A0AAF6YU44	ENSBTAG00000009446	-0.127527001	-0.141073533	0.013546532
F1N5F2	BCKDHA	F1N5F2	ENSBTAG00000016037	-0.187251819	-0.084847964	-0.102403855
A5PJW8	POLR2B	A5PJW8; bta:538184; F1MPP6	ENSBTAG00000019366	-0.134301092	0.037152998	-0.17145409
A0A3Q1MEC9	TNPO2	A0A3Q1MEC9	ENSBTAG00000011811	-0.087914658	0.052519047	-0.140433705
Q3T0E7	PPP1CA	bta:516175; Q3T0E7	ENSBTAG00000012146	-0.124057993	-0.05226731	-0.071790683
B0JYL8	CFL1	B0JYL8; bta:534553		0.017718726	-0.045980653	0.063699379
Q2HJJ0	KLC4	Q2HJJ0		-0.055358593	0.062676496	-0.118035089
A0AAA9T205	RNF20	A0AAA9T205	ENSBTAG00000009516	-0.033535604	-0.172810392	0.139274789
E1BDM8	DDX6	bta:513906; E1BDM8	ENSBTAG00000009067	0.052830957	0.037325536	0.015505421
Q29RL2	CHORDC1	bta:505144; Q29RL2	ENSBTAG00000013615	0.629853958	0.386486586	0.243367372
P28801	GSTP1	bta:281806; P28801; Q3SZU6		0.242914745	0.102380593	0.140534152
P21282	ATP6V1C1	bta:338089; P21282; Q0VCX0	ENSBTAG00000013513	-0.039943512	-0.10274134	0.062797828
A7YY57	ARHGAP29	A7YY57; bta:504657	ENSBTAG00000004190	-0.333811086	-0.448045916	0.114234829
Q32PJ8	HDAC1	bta:404126; Q32PJ8	ENSBTAG00000012698	-0.249089716	-0.136360116	-0.1127296
A0A3Q1M4F5	ANXA7	A0A3Q1M4F5	ENSBTAG00000020218	-0.085359905	-0.020871178	-0.064488726
A4IFN7	UBE3A	A4IFN7; bta:533136	ENSBTAG00000002487	-0.030057234	-0.045323991	0.015266757
F1N036	DNAJC3	F1N036	ENSBTAG00000012065	0.119052672	0.172030148	-0.052977476
F1MR50	SLC4A1AP	F1MR50	ENSBTAG00000018680	0.146785384	-0.060268753	0.207054137
A0AAA9S381	COPS2	A0AAA9S381	ENSBTAG00000013425	0.036069255	0.037552746	-0.001483491
P10103	HMGB1	A5D9G8; bta:282691; P10103; Q3ZC39	ENSBTAG00000018103	-0.020631333	-0.016185428	-0.004445905
F6R9I4	BIN1	F6R9I4	ENSBTAG00000019177	-0.063078683	-0.051610458	-0.011468224
A0AAA9TL16	DOCK6	A0AAA9TL16	ENSBTAG00000009569	-0.289895746	-0.218894977	-0.071000769
A0AAA9S1Z8	BLMH	A0AAA9S1Z8	ENSBTAG00000021370	0.287536447	0.339808868	-0.052272422
A0A3Q1N8A3	CAPZB	A0A3Q1MG13; A0A3Q1N8A3	ENSBTAG00000004554	-0.116023796	-0.116023796	0
A0A452DIW6	MICALL1	A0A452DIW6	ENSBTAG00000037703	-0.336218752	-0.284917524	-0.051301229
A0AAA9TZG3	PRMT5	A0AAA9TZG3	ENSBTAG00000010890	0.016074763	-0.023083613	0.039158376
E1BN58	OTUD4	E1BN58	ENSBTAG00000019857	0.031510973	0.019587805	0.011923167
F1N4V2	CTR9	bta:534265; F1N4V2	ENSBTAG00000015566	0.087649344	0.072652379	0.014996965
P26882	PPID	bta:281420; P26882; Q28077; Q2HJ45	ENSBTAG00000016680	0.551726171	0.469983022	0.081743149
F6QQ96	NUB1	F6QQ96	ENSBTAG00000004540	0.001593258	0.255041615	-0.253448357
F1MZK0	NMT1	F1MZK0	ENSBTAG00000006051	0.21649182	0.093109404	0.123382416
A2VE21	COPG2	A2VE21; bta:536616	ENSBTAG00000017245	-0.100198829	-0.114483055	0.014284226
A7MAZ5	H1-3	A7MAZ5; bta:509275		-0.224966365	0.045562577	-0.270528942
Q32L92	CNN3	bta:538975; Q32L92	ENSBTAG00000020345	0.13876407	0.030200238	0.108563832
F1MH70	NCAPH	F1MH70	ENSBTAG00000012925	-0.319596601	-0.450128164	0.130531563
F1MDU9	HEATR1	F1MDU9	ENSBTAG00000044100	-0.027127178	0.026626502	-0.05375368
A0A3Q1LUY1	SCRIB	A0A3Q1LUY1	ENSBTAG00000008421	-0.40949506	-0.243837905	-0.165657155
P0CB32	HSPA1L	bta:540190; P0CB32	ENSBTAG00000025442	0.75510798	0.510996734	0.244111246
F1MZK4	BZW1	F1MZK4	ENSBTAG00000006049	-0.256794751	-0.327895818	0.071101067
A0AAA9RZ48	ZFR	A0AAA9RZ48	ENSBTAG00000008414	0.094346666	0.01816639	0.076180277
A0AAA9T2D6	SMURF2	A0AAA9T2D6	ENSBTAG00000019853	-0.282604965	-0.278915206	-0.003689759
A0AAA9TMV7	SMG1	A0AAA9TMV7; bta:525143	ENSBTAG00000020735	0.004253649	0.077404504	-0.073150854
Q0VCU8	EIF3J	A6QPP4; bta:539052; Q0VCU8		0.212779635	0.181489407	0.031290228
A0AAA9SQ71	PXN	A0AAA9SQ71	ENSBTAG00000018108	0.055541563	0.079038745	-0.023497181
A0A3Q1LW88	DCAF1	A0A3Q1LW88; bta:785711	ENSBTAG00000006501	-0.105972369	-0.021963431	-0.084008937
A7Z057	YWHAG	A7Z057; bta:286862; F2Z4H9	ENSBTAG00000004077	0.235896686	0.122764448	0.113132238
Q2HJH2	RAB1B	bta:540685; Q2HJH2		0.022195746	-0.071648041	0.093843787
A0A3Q1M4Z7	BCAR1	A0A3Q1M4Z7	ENSBTAG00000012002	-0.339941715	-0.391363963	0.051422248
A0A3Q1LY70	NRDC	A0A3Q1LY70; bta:511254	ENSBTAG00000021174	-0.54221758	-0.555479479	0.013261899
A0AAA9TE99	EFL1	A0AAA9TE99; bta:527607	ENSBTAG00000003410	-0.005125032	0.001704306	-0.006829338
F1N152	HTRA1	bta:282326; F1N152; O97658		0.209638149	0.283402204	-0.073764055
A0A3Q1N0G2	ABHD16A	A0A3Q1N0G2	ENSBTAG00000000578	-0.037631112	-0.039156966	0.001525854
A0AAA9TG77	FKBP15	A0AAA9TG77	ENSBTAG00000005116	0.180473906	0.170110736	0.01036317
A0AAA9SU45	ARFGEF2	A0AAA9SU45	ENSBTAG00000007120	-0.007319625	0.087806381	-0.095126006
A0AAA9T1Q6	TOP2B	A0AAA9T1Q6	ENSBTAG00000004593	-0.235522842	-0.308511361	0.072988519
A0A3Q1MC76	DLG1	A0A3Q1MC76	ENSBTAG00000020378	0.116829955	0.163394003	-0.046564048
F1N4E5	TOR1AIP1	F1N4E5	ENSBTAG00000035226	0.097062128	0.073768867	0.023293261
G3N251	CENPE	G3N251	ENSBTAG00000009035	0.011456515	0.118255274	-0.106798759
A0A3Q1M7Q2	TNIK	A0A3Q1M7Q2	ENSBTAG00000011742	0.092604171	0.054198308	0.038405863
E1BPR4	SEC24B	E1BPR4	ENSBTAG00000010945	0.075872024	0.06445598	0.011416044
Q3SWW9	PPP1CB	bta:538829; Q3SWW9	ENSBTAG00000012447	0.078395071	0.12154691	-0.043151838
Q3ZC32	OPTN	bta:534150; Q3ZC32	ENSBTAG00000016401	-0.143500096	-0.235460401	0.091960304
Q32LG3	MDH2	Q32LG3		0.008878151	-0.016419883	0.025298034
A0AAA9TFJ4	KIF3A	A0AAA9TFJ4	ENSBTAG00000025477	-0.148306425	-0.239145656	0.090839231
A0A3Q1N147	RAB1A	A0A3Q1N147	ENSBTAG00000016720	0.156042373	0.048609944	0.107432429
F1MIX6	DOCK1	F1MIX6	ENSBTAG00000031890	-0.213123123	-0.207852999	-0.005270125
A0A3Q1MIM4	HNRNPH1	A0A3Q1MIM4; bta:541202	ENSBTAG00000009389	-0.027619187	-0.087552833	0.059933646
P32007	SLC25A6	A1L5C3; A6QQX7; bta:282480; P32007		-0.177986046	-0.277423674	0.099437628
A0AAA9TG74	PPP2R1B	A0AAA9TG74	ENSBTAG00000020277	-0.446862945	-0.377069649	-0.069793296
A0A3Q1LHD8	RPS6KA3	A0A3Q1LHD8	ENSBTAG00000017639	0.045293387	0.016235103	0.029058284
A5D7B4	DENND2B	A5D7B4; bta:537907; F6QME3	ENSBTAG00000005356	-0.114511881	0.041406262	-0.155918143
P11966	PDHB	A6QQW6; bta:613610; P11966; Q58CN6	ENSBTAG00000021724	0.009618003	-0.057420116	0.067038118
A0A3Q1LYB8	WNK1	A0A3Q1LYB8	ENSBTAG00000005221	-0.070033327	-0.151528445	0.081495118
A0A452DJA8				-0.361832882	-0.193013818	-0.168819064
A0A3Q1NMR2	SPATS2L	A0A3Q1NMR2	ENSBTAG00000016092	0.09439237	-0.016783219	0.111175589
A0AAF6Z5Y5	FBLIM1	A0AAF6Z5Y5	ENSBTAG00000018725	-0.033337184	-0.052196211	0.018859027
Q08E27	STRBP	bta:516282; Q08E27		0.255257055	0.117266052	0.137991003
E1B844	TIMM44	E1B844	ENSBTAG00000015567	-0.124533857	-0.207611745	0.083077888
F1MVK2	SCYL1	F1MVK2	ENSBTAG00000006740	0.014167332	-0.069677647	0.083844979
Q2KI14	NAA10	bta:613636; Q2KI14		0.01772012	0.013310454	0.004409667
A0A3Q1LX10	RCC1	A0A3Q1LX10	ENSBTAG00000003788	0.004441343	0.002962414	0.001478929
F1MH45	CDC42BPA	F1MH45	ENSBTAG00000022777	-0.11417102	-0.071601587	-0.042569433
O77784	IDH3B	bta:613338; O77784; O77785; Q3MI01	ENSBTAG00000018813	-0.03678667	-0.059317504	0.022530833
F1MSR6	TSR1	F1MSR6	ENSBTAG00000004225	-0.170248802	-0.24655217	0.076303367
F1MBS5	KDM1A	F1MBS5	ENSBTAG00000009500	-0.1935501	-0.254195109	0.060645009
A0A3Q1MWG7	ME2	A0A3Q1MWG7	ENSBTAG00000016269	0.096167435	0.069540933	0.026626502
A7MB62	ACTR2	A7MB62; bta:538486	ENSBTAG00000009761	0.023083613	0.051428239	-0.028344626
A0AAA9T768	RAB3GAP2	A0AAA9T768	ENSBTAG00000012585	0.045293387	0.075187496	-0.029894109
Q2KJ39	RCN3	bta:522073; Q2KJ39		0.199079253	-0.005346621	0.204425875
Q5EAC7	CIAPIN1	bta:535119; Q0VCL8; Q5EAC7		-0.169010804	-0.169010804	0
A6H7B5	COPS3	A6H7B5; bta:507932	ENSBTAG00000018973	-0.022968764	0.005685505	-0.028654269
E1BCH9	VCPIP1	E1BCH9	ENSBTAG00000006695	-0.046656625	0.100740894	-0.147397519
Q0P5M8	PMPCA	bta:767847; Q0P5M8	ENSBTAG00000001353	-0.336070665	-0.342696142	0.006625477
A0AAA9SY31	CORO1C	A0AAA9SY31	ENSBTAG00000007993	0.100111485	0.201258208	-0.101146723
A0AAA9T271	TMEM214	A0AAA9T271	ENSBTAG00000017423	0.066021969	-0.113976754	0.179998723
A0AAA9RZQ0	TTF2	A0AAA9RZQ0	ENSBTAG00000015392	-0.20332816	-0.20332816	0
A0AAA9RUL8	MFGE8	A0AAA9RUL8	ENSBTAG00000003300	-0.289506617	-0.273404943	-0.016101674
Q95115	STAT5A	bta:282375; O46628; O97606; Q95115; Q9N0U8; Q9TUM1	ENSBTAG00000009496	-0.145605322	-0.053560245	-0.092045077
Q17QP9	PSMD14	bta:535683; Q17QP9	ENSBTAG00000021691	0.01451457	-0.05652518	0.071039337
P61763	STXBP1	bta:282378; P61763; Q28208; Q62759; Q64320; Q96TG8		-0.057800106	0.125646035	-0.183446141
E1BPW1	OSBP	E1BPW1	ENSBTAG00000020332	0.076647382	0.083807174	-0.007159792
A0AAF6YIQ2	CIAPIN1	A0AAF6YIQ2	ENSBTAG00000001854	0.042213525	0.134301092	-0.092087567
A0AAA9TQX8	RPS3	A0AAA9TQX8	ENSBTAG00000005620	-0.157261766	-0.230693686	0.07343192
Q2LGB8	CASP8	bta:507481; Q2LGB8	ENSBTAG00000015718	0.038219322	0.102759574	-0.064540252
A0AAA9TCE5	LEMD3	A0AAA9TCE5	ENSBTAG00000039435	0	-0.00877021	0.00877021
F1MTR3	SIN3A	F1MTR3	ENSBTAG00000009985	-0.051735645	-0.093297635	0.041561991
G3N3B7	PTK2	G3N3B7	ENSBTAG00000009578	-0.027100358	0.09169569	-0.118796048
A4IFE0	CCDC93	A4IFE0; bta:616839; F1MDJ4	ENSBTAG00000010221	0.050783329	0.10408916	-0.053305831
Q5E966	EIF3I	bta:521747; Q3SZ38; Q5E966	ENSBTAG00000014388	-0.138476768	-0.129326451	-0.009150316
A0A3Q1LPY4	PCBP2	A0A3Q1LPY4	ENSBTAG00000020757	0.050040682	0.095518313	-0.045477631
A0AAA9SIU0	FRYL	A0AAA9SIU0	ENSBTAG00000000137	-0.02120609	-0.101742703	0.080536613
F1N7C1	HERC6	F1N7C1	ENSBTAG00000020536	-0.428093652	-0.229123346	-0.198970306
E1BPA1	TBC1D4	bta:534649; E1BPA1	ENSBTAG00000005760	-0.068315	0.13034172	-0.19865672
A0JN78	HEATR3	A0JN78; bta:539353; F1MLE2	ENSBTAG00000016640	-0.115800728	-0.271078953	0.155278225
Q1RMX7	NANS	bta:540602; Q1RMX7	ENSBTAG00000019023	0.159152087	0.146539811	0.012612276
A0AAA9TIF9	GLRX3	A0AAA9TIF9	ENSBTAG00000005027	0.148134877	0.114219469	0.033915409
Q3SWY2	ILK	bta:540207; Q3SWY2	ENSBTAG00000000696	-0.128539381	-0.175556007	0.047016627
Q58DT4	PYCR1	bta:539606; Q58DT4	ENSBTAG00000000042	-0.066797057	0.257918855	-0.324715912
Q2NL05	KIF2A	bta:768014; Q2NL05		-0.024095676	-0.037828466	0.01373279
Q5E9F1	BCAP31	bta:533949; Q5E9F1	ENSBTAG00000020547	0.228881483	0.175669677	0.053211806
E1BBJ7	INPPL1	bta:783833; E1BBJ7	ENSBTAG00000019167	-0.129196236	-0.106594807	-0.022601429
P62935	PPIA	P04374; P10110; P62935; Q29580; Q56JX4	ENSBTAG00000012003	-0.043589269	-0.049501961	0.005912693
A0A493UA87	AKR1B1	A0A493UA87; bta:317748	ENSBTAG00000009902	0.285235126	0.265855349	0.019379777
Q3SX26	TRIP6	bta:615869; Q3SX26	ENSBTAG00000024772	0.008158523	0.120294234	-0.11213571
F1N155	CLPX	F1N155	ENSBTAG00000008386	-0.499084666	-0.550746785	0.05166212
A0AAA9T4H8	SUCLG2	A0AAA9T4H8	ENSBTAG00000009541	0.073047783	0.076665073	-0.00361729
G3X6R3	STAG2	G3X6R3	ENSBTAG00000009121	-0.092397138	-0.209536857	0.117139719
A5PJA6	STOML2	A5PJA6	ENSBTAG00000011388	-0.267811546	-0.184153617	-0.083657929
D3JUI8	RBMX	bta:509591; D3JUI8	ENSBTAG00000013308	-0.062531783	-0.109624491	0.047092708
A0AAA9S0E1	NPM1	A0AAA9S0E1	ENSBTAG00000015316	0.014781841	0.059666089	-0.044884248
Q3MHZ4	TGFB1I1	bta:515834; Q3MHZ4		-0.172616597	-0.225084017	0.05246742
Q3SYS9	KIFBP	bta:527433; Q3SYS9	ENSBTAG00000014561	0.028014376	0	0.028014376
Q3T005	PDLIM4	bta:515410; Q3T005	ENSBTAG00000015426	0.22757266	0.330207573	-0.102634912
Q0VD16	MAP2K1	bta:533199; F2Z4G4; Q0VD16	ENSBTAG00000033983	0.010908899	-0.026244099	0.037152998
Q5XQN5	KRT5	bta:281268; Q5XQN5		-0.295718216	1.445020365	-1.740738581
A0A3Q1NBU3	AGPS	A0A3Q1NBU3	ENSBTAG00000009897	0.053199567	-0.043113106	0.096312673
P56658	ADA	bta:280712; P56658; Q9MYY1		0.177187936	0.134659509	0.042528427
F1MF74	MICAL2	A0A3Q1LSU4; F1MF74		-0.1586348	-0.193805578	0.035170778
A5D7A5	GOLGA5	A5D7A5; bta:510412		0.001780006	0.040394588	-0.038614581
A0A3Q1MCE5	AACS	A0A3Q1MCE5; bta:505842	ENSBTAG00000005678	-0.03496766	-0.069353281	0.034385621
Q1RMT6	DBN1	bta:505406; F1N3U4; Q1RMT6		0.172628614	0.197829168	-0.025200554
A0A3Q1M8R6	PML	A0A3Q1M8R6	ENSBTAG00000015779	-0.120235227	-0.073786304	-0.046448923
A1L555	PSAP	A1L555		0.046685067	0.190720201	-0.144035134
A0AAA9T9H6	XRCC5	A0AAA9T9H6	ENSBTAG00000010426	0.052622607	0.069285295	-0.016662688
A4FV36	SPATA20	A4FV36; bta:511363	ENSBTAG00000004155	-0.267198031	-0.189283303	-0.077914727
F1MMN6	ITGA1	F1MMN6	ENSBTAG00000016525	0.031351549	-0.073444641	0.10479619
E1BKQ2	RAB11FIP1	E1BKQ2	ENSBTAG00000011684	-0.200519954	-0.170110736	-0.030409218
A0AAA9TAD8	ADK	A0AAA9TAD8	ENSBTAG00000011072	0.039702424	0.102538162	-0.062835738
A0AAA9TTF2	DYNC1LI2	A0AAA9TTF2	ENSBTAG00000000458	-0.129164223	-0.184251783	0.05508756
A4FUZ6	HSDL2	A4FUZ6; A6QPZ9; bta:404131	ENSBTAG00000031295	-0.116863758	-0.091539601	-0.025324156
E1BB48	GALNT2	E1BB48	ENSBTAG00000004870	-0.096970088	-0.15669988	0.059729792
A0AAA9SDW9	BTAF1	A0AAA9SDW9	ENSBTAG00000016104	-0.574908836	-0.468709432	-0.106199404
A0A3Q1LN63	SERPINB1	A0A3Q1LN63	ENSBTAG00000011975	-0.14419115	-0.060415658	-0.083775492
Q17QI3	ACAT2	Q17QI3	ENSBTAG00000002827	0.157390365	0.11760765	0.039782715
A0A3Q1N1J5	PLBD2	A0A3Q1N1J5	ENSBTAG00000019040	-0.336031839	-0.186303551	-0.149728287
Q3ZBW4	PCNA	bta:515499; Q3ZBW4	ENSBTAG00000006065	0.050073768	-0.148610226	0.198683994
F1MBM9	ARHGEF7	F1MBM9	ENSBTAG00000020726	0.150533205	0.155817808	-0.005284603
A0A3Q1LN88	UACA	A0A3Q1LN88	ENSBTAG00000013982	-0.284003471	-0.214948054	-0.069055417
A6QNT8	SEC24A	A6QNT8; bta:505089	ENSBTAG00000018322	-0.046025108	-0.108288545	0.062263437
Q2KJE7	PSME1	bta:510041; Q2KJE7	ENSBTAG00000021395	-0.121254643	-0.022252807	-0.099001836
A0AAA9S0H3	EML4	A0AAA9S0H3	ENSBTAG00000010935	0.104164593	0.230612928	-0.126448335
A0A3Q1MH45	GSPT2	A0A3Q1MH45; bta:538724	ENSBTAG00000013311	0.153805336	0.081794091	0.072011245
A0A3Q1LUV2	PGM3	A0A3Q1LUV2	ENSBTAG00000000679	0.159059153	0.125197195	0.033861958
O02811	PI4KA	bta:282309; F1MLV0; O02811		-0.291022053	-0.08902566	-0.201996393
G5E5C4	PDCD11	G5E5C4	ENSBTAG00000009715	-0.244355853	-0.006827722	-0.23752813
F1MGC0	SUCLA2	F1MGC0	ENSBTAG00000006759	-0.059084131	-0.083089594	0.024005463
A0AAA9RZ74	SF1	A0AAA9RZ74	ENSBTAG00000001035	0.026284645	0.013202182	0.013082463
A0AAA9RVE8	RBM15	A0AAA9RVE8	ENSBTAG00000060532	0.048461489	0.099535674	-0.051074185
A0A3Q1MB23				-0.211504105	-0.082903082	-0.128601024
Q5E970	DDX39A	bta:614215; Q5E970	ENSBTAG00000021820	-0.054294085	-0.12807628	0.073782195
O46563	ATP6V1H	A0A3Q1M3I3; bta:282657; F1MZL6; O46562; O46563; Q3ZBD5	ENSBTAG00000003450	0.101908528	-0.025671408	0.127579935
E1BM12	SRM	bta:505916; E1BM12	ENSBTAG00000005304	-0.073184347	-0.079727192	0.006542846
A0AAA9U092	SEPTIN6	A0AAA9U092	ENSBTAG00000004291	-0.121799687	-0.015220737	-0.106578951
E1BJF6	SYMPK	E1BJF6	ENSBTAG00000012101	0.016591553	-0.032214625	0.048806178
B0JYK4	PRKAR2B	B0JYK4		0.114902095	0.185620092	-0.070717997
F6PWU2	BTF3	F6PWU2	ENSBTAG00000010012	-0.05246742	-0.283792966	0.231325546
A0A3Q1LHG7	MARK2	A0A3Q1LHG7	ENSBTAG00000007825	-0.075387122	-0.145703441	0.070316319
Q3ZCJ2	AKR1A1	bta:618607; Q3ZCJ2	ENSBTAG00000000497	0.024711753	0.040479069	-0.015767316
P23004	UQCRC2	P23004; Q3ZCG7; Q5E9C7	ENSBTAG00000021651	-0.034548227	-0.034548227	0
E1BB46	SH3BP4	bta:520462; E1BB46	ENSBTAG00000013869	-0.219953376	-0.150957283	-0.068996093
A0AAA9SN47	SF1	A0AAA9SN47	ENSBTAG00000001035	0.219918008	0.037272989	0.182645019
A0A3Q1N3E6	GRIPAP1	A0A3Q1N3E6	ENSBTAG00000007788	-0.209407221	0.066375678	-0.275782899
F1MGJ5	SEL1L	F1MGJ5	ENSBTAG00000008083	0.058830481	0.105108164	-0.046277683
A0AAA9S5S3	MTMR6	A0AAA9S5S3	ENSBTAG00000003116	0.073045325	0.016070693	0.056974631

Accession	Gene_Symbol	Gene_ID	Ensembl_Gene ID	log2(ratio(OV24H/OV0H))	log2(ratio(OV24R/OV0H))	log2(ratio(OV24H/OV24R))
Q2KI95	FHL2	bta:510008; Q2KI95	ENSBTAG00000001086	0.207139116	0.248703698	-0.041564583
A0A3Q1N4H1	FANCI	A0A3Q1N4H1	ENSBTAG00000009097	-0.21488889	-0.102514911	-0.112373979
Q3ZC13	AP2M1	bta:517446; Q3ZC13	ENSBTAG00000020106	-0.19863843	-0.218249449	0.019611018
A0AAA9SH40	TRRAP	A0AAA9SH40	ENSBTAG00000007113	-0.15929345	0.190331212	-0.349624662
A0AAA9SQZ6	GPATCH8	A0AAA9SQZ6	ENSBTAG00000038316	-0.024274746	-0.011277406	-0.012997341
P20000	ALDH2	bta:508629; P20000; Q1LZC6	ENSBTAG00000008743	0.031049923	0.06765524	-0.036605317
E1BJ19	TAOK1	bta:536532; E1BJ19	ENSBTAG00000000827	0.089498151	0.116364757	-0.026866606
A0AAA9TYT8	ALDH4A1	A0AAA9TYT8	ENSBTAG00000030335	-0.108807524	-0.108807524	0
A0AAA9TMW1	EIF3M	A0AAA9TMW1	ENSBTAG00000005338	-0.048859401	-0.161578359	0.112718957
A0AAA9TJ26	ACAD10	A0AAA9TJ26	ENSBTAG00000001164	-0.010715026	0.498007919	-0.508722945
F1MUW6	TMF1	F1MUW6	ENSBTAG00000015554	0.23430423	0.159871337	0.074432893
A0A3Q1LMM5	SRGAP2	A0A3Q1LMM5; bta:519843	ENSBTAG00000001483	0.008478154	0.03638415	-0.027905997
A0A3Q1MNY8	PRP4K	A0A3Q1MNY8	ENSBTAG00000002086	0.131316547	0.011548221	0.119768326
F1MKF8	SQOR	F1MKF8	ENSBTAG00000010365	0.178046187	0.201556132	-0.023509945
A0AAA9TLS4	SPTBN2	A0AAA9TLS4	ENSBTAG00000005929	-0.360251332	-0.285338766	-0.074912566
A0AAF7AS54	VRK1	A0AAF7AS54	ENSBTAG00000014230	-0.185089637	-0.187169948	0.00208031
G3MWV5	H1-4	G3MWV5	ENSBTAG00000047206	-0.294253136	-0.050888711	-0.243364426
F6Q807	HNRNPUL1	F6Q807	ENSBTAG00000003168	0.078644494	0.261508552	-0.182864057
F1MW06	NOP56	F1MW06	ENSBTAG00000018812	-0.142352923	0.059612783	-0.201965706
A0AAA9TGS6	BZW2	A0AAA9TGS6	ENSBTAG00000014262	-0.077702357	-0.215205881	0.137503524
E1BL45	EHBP1L1	E1BL45	ENSBTAG00000039554	-0.014303538	-0.06619028	0.051886742
A6QP83	FUBP3	A6QP83; bta:541080	ENSBTAG00000013072	0.190023279	0.238650247	-0.048626968
A0A3Q1LHD2	PBRM1	A0A3Q1LHD2; bta:506557	ENSBTAG00000014786	-0.393284701	-0.245351272	-0.147933429
F1N6E9	RTF1	bta:518260; F1N6E9	ENSBTAG00000001671	0.13003388	-0.050349975	0.180383855
A0AAA9SME9	POGLUT2	A0AAA9SME9	ENSBTAG00000008880	-0.542479169	-0.553794483	0.011315313
P48452-2	PPP3CA	P48452-2		0.081260615	0.031364171	0.049896444
A0A3Q1LQ03	SON	A0A3Q1LQ03	ENSBTAG00000008482	0.061209901	0.100145564	-0.038935663
A0AAA9SLN8	KIF14	A0AAA9SLN8	ENSBTAG00000021874	0.183540373	0.155471491	0.028068882
A0A3Q1N6B7	LRCH3	A0A3Q1N6B7	ENSBTAG00000012839	-0.345511442	-0.321612026	-0.023899415
A0A3Q1M3I7	PPP1R9B	A0A3Q1M3I7	ENSBTAG00000049864	0.092296847	0.086868073	0.005428774
A0AAA9TKA2	CLIC1	A0AAA9TKA2	ENSBTAG00000013533	0.033983681	-0.107035444	0.141019125
Q3SWX9	RAD21	bta:540966; Q3SWX9	ENSBTAG00000007303	-0.031209959	-0.132180437	0.100970477
A0AAA9SLV9	HSPA14	A0AAA9SLV9	ENSBTAG00000016451	0.063103922	0.072111413	-0.00900749
Q2TBG8	UCHL3	bta:520170; Q2TBG8		0.592760871	0.328948523	0.263812349
A0A3Q1LWN6	MYH11	A0A3Q1LWN6	ENSBTAG00000015988	0.187550672	0.132944341	0.054606331
A0A3Q1NL10	SORBS2	A0A3Q1NL10	ENSBTAG00000016486	0.019172309	0.198063092	-0.178890783
A0AAF6YIJ5	NDUFS2	A0AAF6YIJ5	ENSBTAG00000022203	0.003102571	0.042840328	-0.039737757
A0A3Q1LZH0	PKN2	A0A3Q1LZH0	ENSBTAG00000021900	0.017124178	0.075507088	-0.05838291
A0AAA9RYK0	TWF2	A0AAA9RYK0	ENSBTAG00000054131	0.105749732	0.122190879	-0.016441147
F1MFS4	SRGAP1	bta:539452; F1MFS4	ENSBTAG00000011892	-0.064899775	-0.069332021	0.004432246
P02253	H1-2	A3KN02; bta:513971; P02253	ENSBTAG00000011677	-0.321928095	0.112474729	-0.434402824
P34955	SERPINA1	bta:280699; P34955; Q3SZS3	ENSBTAG00000018843	0.108813487	0.636123401	-0.527309913
F1MUH9	CTTNBP2NL	bta:538981; F1MUH9	ENSBTAG00000021602	-0.06788631	-0.102275035	0.034388726
F1MWP1	BUB1B	F1MWP1	ENSBTAG00000007237	0.185142747	-0.076838981	0.261981728
F1MC11	KRT14	F1MC11	ENSBTAG00000007583	-0.063826303	2.005563813	-2.069390116
A0AAF7AML4	RPAP1	A0AAF7AML4	ENSBTAG00000001693	0.04940224	0.042242449	0.007159792
A0A3Q1LLS1	CTPS2	A0A3Q1LLS1	ENSBTAG00000017017	0.176141237	0.065497173	0.110644064
A0A3Q1MIY3	CAPNS1	A0A3Q1MIY3	ENSBTAG00000014872	0.160698049	0.010310269	0.15038778
F1MK70	SPATS2	bta:617860; F1MK70	ENSBTAG00000004660	0.187627003	0.028992203	0.1586348
P67774	PPP2CA	A6QLI7; bta:282320; P05323; P13197; P67774	ENSBTAG00000000469	0.177304532	0.12156198	0.055742552
A0AAA9T615	PCMT1	A0AAA9T615	ENSBTAG00000010204	0.21788955	0.184755806	0.033133744
Q2KIT4	DNAJB4	bta:541274; Q2KIT4	ENSBTAG00000021752	0.493888406	0.145197916	0.34869049
A0A3Q1M986	GALNT2	A0A3Q1M986	ENSBTAG00000004870	-0.061325962	-0.038242349	-0.023083613
A4FV40	EIF2B3	A4FV40; bta:534063; E1BB97	ENSBTAG00000019010	0.035414028	0.031209959	0.004204068
Q27977	ITGA5	F1MK44; Q27977	ENSBTAG00000013745	-0.140089772	-0.052626931	-0.087462841
A0AAA9T986	STIM1	A0AAA9T986	ENSBTAG00000013109	0.057312653	0.099219605	-0.041906952
F1MQI1	TLN2	F1MQI1	ENSBTAG00000003667	-0.031218731	-0.116335344	0.085116614
A0AAA9RRS3	TMOD3	A0AAA9RRS3	ENSBTAG00000048622	0.061400545	0.007448107	0.053952438
A0A3Q1LYB3	COPS6	A0A3Q1LYB3	ENSBTAG00000002871	-0.001491158	-0.053129966	0.051638809
A5D9B4	HNRNPH2	A5D9B4	ENSBTAG00000007499	-0.025014207	-0.053494307	0.0284801
A0AAA9SHR8	CAPG	A0AAA9SHR8	ENSBTAG00000005432	-0.104546776	-0.113610859	0.009064082
A0AAF6DLX8	KIF2C	A0AAF6DLX8	ENSBTAG00000015280	0.1944541	-0.06668327	0.261137371
G5E5C8	TALDO1	bta:513453; G5E5C8	ENSBTAG00000010336	-0.013056153	0.005189556	-0.018245709
F1MV75	WDR48	F1MV75	ENSBTAG00000003745	0.175538608	0.099324738	0.07621387
P00514	PRKAR1A	A5D9F4; bta:615074; P00514; Q17QP8	ENSBTAG00000008621	-0.125303597	-0.544893624	0.419590028
A0A3Q1LUG9	TXNDC5	A0A3Q1LUG9; bta:615027	ENSBTAG00000019237	0.040167432	-0.034573149	0.07474058
Q3T0C7	STMN1	bta:616317; Q3T0C7	ENSBTAG00000013761	0.018147347	-0.10007289	0.118220237
Q0P594	PPP2CB	bta:524361; Q0P594	ENSBTAG00000009245	0.329092069	0.327409626	0.001682443
Q29RK1	CS	bta:280682; Q29RK1	ENSBTAG00000004371	-0.029686131	-0.044761451	0.01507532
Q3SWY7	SF3A3	bta:523250; Q3SWY7	ENSBTAG00000007299	-0.066551193	-0.026855492	-0.039695701
G3N2U8	CTNNA2	G3N2U8	ENSBTAG00000031669	-0.306771949	-0.10006115	-0.206710799
A2T1U6	AP-B	A2T1U6		-0.290335812	-0.128671144	-0.161664668
F1MI41	SYNRG	F1MI41	ENSBTAG00000010292	0.247664144	0.256068216	-0.008404073
F1MEX9	ACSL3	bta:100138312; F1MEX9	ENSBTAG00000017258	0.086433247	0.386929554	-0.300496307
Q0V850	HGS	bta:511582; Q0V850; Q2NKZ6		0.062984786	0.205572703	-0.142587917
Q08DQ2	GFPT2	bta:530101; Q08DQ2		-0.121621137	-0.143257051	0.021635914
A4FV97	RSL1D1	A4FV97	ENSBTAG00000014588	-0.080342108	-0.074000581	-0.006341527
Q1LZB6	CAPRIN1	bta:535571; Q1LZB6	ENSBTAG00000016744	-0.205414086	-0.445009579	0.239595493
A0AAA9TU20	NUP50	A0AAA9TU20	ENSBTAG00000017652	0.00443679	-0.159593637	0.164030427
A0A3Q1LP50	LARP7	A0A3Q1LP50	ENSBTAG00000009632	0.065050549	0.174957079	-0.109906529
F1N746	RABGAP1	bta:519260; F1N746	ENSBTAG00000039172	0.128514741	0.02075856	0.10775618
A0AAF7AMR3	NT5C2	A0AAF7AMR3	ENSBTAG00000012858	-0.314475791	-0.389009947	0.074534155
F1N1R4	DIS3	F1N1R4	ENSBTAG00000019889	-0.134957341	-0.124115781	-0.01084156
A0AAA9TZA6	FTO	A0AAA9TZA6	ENSBTAG00000012501	0.028166308	-0.082926991	0.111093298
F1MLG3	TRAPPC8	F1MLG3	ENSBTAG00000007696	-0.07350867	-0.041087192	-0.032421478
Q5E959	STRAP	bta:510201; Q5E959	ENSBTAG00000014175	0.003092595	-0.134565401	0.137657996
A0AAA9S5I1	ACTR10	A0AAA9S5I1	ENSBTAG00000014392	-0.368165316	-0.439264341	0.071099025
Q17QQ2	TPMT	bta:511644; Q17QQ2	ENSBTAG00000019300	0.189981577	0.126894875	0.063086702
A0AAA9RXW5	PCBP2	A0AAA9RXW5	ENSBTAG00000020757	0.036296129	0.025803755	0.010492374
Q0VCY1	VAPA	bta:516024; Q0VCY1	ENSBTAG00000017279	0.078229155	0.060443215	0.017785939
P56652	ITIH3	A7MB92; bta:508355; P56652; Q0V8M9	ENSBTAG00000007846	0.056710644	0.546443688	-0.489733044
A7MBG0	ADSS2	A7MBG0; bta:522529	ENSBTAG00000013885	0.077385052	0.01684351	0.060541542
Q1LZD9	PSPC1	bta:506696; Q1LZD9		-0.24161374	-0.16942554	-0.0721882
A0A3Q1LMX5	ABCC1	A0A3Q1LMX5	ENSBTAG00000021090	0.004543925	0.298991284	-0.294447358
E1BMU6	AKAP1	bta:532072; E1BMU6	ENSBTAG00000025320	-0.028261335	0.148781071	-0.177042406
A0A3Q1LN12	SEPTIN8	A0A3Q1LN12	ENSBTAG00000008609	0.042609349	0.12312639	-0.080517041
G3X6E6	TANC1	bta:507983; G3X6E6	ENSBTAG00000004177	-0.018908463	-0.060094347	0.041185885
A0A3S5ZPF7	PSAT1	A0A3S5ZPF7	ENSBTAG00000013960	0.53158915	0.877958104	-0.346368954
Q10741	ADAM10	A8E663; bta:282132; Q10741	ENSBTAG00000005481	-0.051119432	-0.125873362	0.074753929
Q3SX08	EMD	Q3SX08	ENSBTAG00000022314	-0.076156271	-0.117183539	0.041027268
A0AAF6YQ83	DRG2	A0AAF6YQ83	ENSBTAG00000006517	-0.135992321	-0.163952402	0.027960081
A0A3Q1MKT3	GPSM1	A0A3Q1MKT3	ENSBTAG00000018799	-0.125900378	0.045337956	-0.171238333
A0A3Q1M3Z5	CTSD	A0A3Q1M3Z5	ENSBTAG00000007622	0.028014376	0.03197239	-0.003958014
A4IFH7	MAP2K3	A4IFH7; bta:516039; F6S1D0	ENSBTAG00000010576	0.042095736	0.006873262	0.035222473
E1BPP1	TTK	E1BPP1	ENSBTAG00000005456	0.221531625	-0.041244488	0.262776113
A0A3Q1MLW0	PPP1CC	A0A3Q1MLW0	ENSBTAG00000011198	-0.018033923	-0.105950513	0.08791659
F1MKX4	PSME4	F1MKX4		-0.349536756	-0.136321471	-0.213215284
A6QR55	USP4	A6QR55; bta:508042	ENSBTAG00000011899	-0.075065815	-0.063635234	-0.011430581
A0A3Q1MMU8	ABR	A0A3Q1MMU8	ENSBTAG00000008424	-0.101849254	-0.116618713	0.014769459
Q2KJ77	HSPBP1	bta:512757; Q2KJ77	ENSBTAG00000003644	0.28920015	0.19958062	0.08961953
A0A5H1ZRH6	CDH2	A0A5H1ZRH6	ENSBTAG00000021190	0.346568731	0.311358853	0.035209878
Q58DM8	ECHS1	bta:281748; Q2TBV2; Q4PS76; Q58DM8		-0.048634775	-0.073581132	0.024946357
A0AAA9TS26	CNP	A0AAA9TS26	ENSBTAG00000025762	0.145561562	0.129400225	0.016161337
A0AAA9SUZ5	CKB	A0AAA9SUZ5	ENSBTAG00000035998	0.006239655	-0.150515012	0.156754666
E1B7G3	STUB1	E1B7G3	ENSBTAG00000019136	-0.130243651	0.003231121	-0.133474772
A0A452DI66	F2	A0A452DI66	ENSBTAG00000007148	-0.104382431	-0.020281031	-0.0841014
Q3T165	PHB1	bta:530409; Q3T165	ENSBTAG00000017120	-0.158950032	-0.205095598	0.046145566
F1N1A3	UBQLN1	F1N1A3	ENSBTAG00000014827	0.098862932	0.028540892	0.07032204
E1BNG7	TUBGCP3	E1BNG7	ENSBTAG00000044184	-0.097847323	-0.049623377	-0.048223947
Q2KII9	DYNC1LI1	bta:510330; F1N4N4; Q2KII9	ENSBTAG00000014700	0.029175818	0.001472889	0.027702929
Q5E9J1	HNRNPF	bta:506917; Q5E9J1	ENSBTAG00000008853	0.211796178	0.087273821	0.124522357
F1MUD4	USP19	F1MUD4	ENSBTAG00000018921	0.226068079	0.17990909	0.046158989
E1BA93	SYNPO	E1BA93	ENSBTAG00000013744	0.091630475	0.115022324	-0.023391848
E1BE82	PUM1	E1BE82	ENSBTAG00000018979	-0.009896036	0.049848549	-0.059744586
Q0VC01	HDAC2	bta:407223; Q0VC01		-0.139702853	-0.313549677	0.173846824
A0A3Q1LYL5	PCCA	A0A3Q1LYL5	ENSBTAG00000010672	0.034080995	0.072850565	-0.03876957
A0AAA9TES1	RAB5C	A0AAA9TES1	ENSBTAG00000006982	0.044825199	-0.007131478	0.051956677
A0A3Q1LPE3	PCCB	A0A3Q1LPE3	ENSBTAG00000015221	-0.12265125	-0.042908591	-0.079742659
Q9BGI3	PRDX2	bta:286793; Q3T0L0; Q9BGI3		0.07356746	-0.110536476	0.184103937
A0AAF7AHP7	EIF5A	A0AAF7AHP7	ENSBTAG00000002018	-0.105645039	-0.144679753	0.039034713
A0AAA9RWC4	VPS26A	A0AAA9RWC4	ENSBTAG00000016882	0.065015191	-0.006801969	0.071817161
E1BK29	COG1	E1BK29	ENSBTAG00000006087	-0.056523605	0.005980092	-0.062503696
F6R2J7	SETD3	F6R2J7	ENSBTAG00000018310	-0.192865522	-0.235827358	0.042961836
Q1JQE0	SNW1	bta:326578; Q1JQE0	ENSBTAG00000008136	-0.066975695	-0.163033422	0.096057727
A0A3Q1MGW7	STRN	A0A3Q1MGW7	ENSBTAG00000018590	-0.02726208	0.127779638	-0.155041718
A5PJR5	CSTF3	A5PJR5; bta:508491; E1BGY7		-0.1015806	-0.042862325	-0.058718275
E1BC55	KIAA1671	E1BC55	ENSBTAG00000044022	0.007037551	0.082266182	-0.075228631
F1N206	DLD	bta:533910; F1N206	ENSBTAG00000001908	0.054171474	0.147370685	-0.093199211
Q3MHG1	SPTLC1	bta:614165; Q3MHG1	ENSBTAG00000002220	-0.051138849	0.128995885	-0.180134734
A0AAF6YS25	GNPDA1	A0AAF6YS25	ENSBTAG00000007865	0.090386236	0.117007117	-0.02662088
Q08DE9	CUL2	bta:535219; F1MWA4; Q08DE9		0.213638586	0.184988381	0.028650205
P10895	PLCD1	A2VDM2; bta:281986; P10895	ENSBTAG00000037726	-0.059150602	-0.073351742	0.01420114
A0A3Q1M3Y2	CHD3	A0A3Q1M3Y2	ENSBTAG00000014377	-0.250657463	-0.078810847	-0.171846615
F1MFC6	FARP1	F1MFC6	ENSBTAG00000000146	-0.228792661	-0.010030365	-0.218762295
F1MJ80	NAMPT	F1MJ80	ENSBTAG00000015509	0.111591691	0.160553168	-0.048961476
F1N565	ZC3H14	F1N565	ENSBTAG00000030453	0.09843663	0.013420516	0.085016114
E1BPP6	MCCC2	E1BPP6	ENSBTAG00000044160	-0.059027576	0.117206327	-0.176233903
F1N7X3	NAP1L4	F1N7X3	ENSBTAG00000022160	-0.004459648	-0.03911613	0.034656482
Q05B79	DHX36	bta:509583; Q05B79		0.01493627	-0.022005308	0.036941578
A0AAA9T043	THADA	A0AAA9T043; bta:514187	ENSBTAG00000003555	-0.234990593	-0.23349013	-0.001500463
A0AAA9SUD1	ACADS	A0AAA9SUD1	ENSBTAG00000007484	-0.128642151	-0.041619089	-0.087023062
F6RFP6	BSG	F6RFP6	ENSBTAG00000016648	0.206342928	0.089461627	0.1168813
E1BME9	NDUFV3	E1BME9	ENSBTAG00000021491	-0.094137931	-0.061583431	-0.0325545
Q2HJ54	PITPNA	bta:616550; Q2HJ54; Q9TR37	ENSBTAG00000011480	0.023481433	-0.068441056	0.091922489
A0AAA9TU53	DCBLD2	A0AAA9TU53	ENSBTAG00000014889	0.021542619	0.099876092	-0.078333474
A0AAA9T5N2	PHB2	A0AAA9T5N2	ENSBTAG00000013123	-0.100791745	-0.16604293	0.065251185
F1N6W6	SNX9	F1N6W6	ENSBTAG00000006323	0.085641725	0.162393329	-0.076751604
A0AAA9SHB3	TP53BP2	A0AAA9SHB3	ENSBTAG00000012781	-0.063866204	-0.091463763	0.027597559
A0AAA9SWE4	ANXA11	A0AAA9SWE4	ENSBTAG00000020940	0.028828653	0.118121939	-0.089293286
E1BF68	DHX16	bta:506405; E1BF68	ENSBTAG00000006960	0.16549003	0.015853951	0.149636079
A0A3Q1NM85	KIF13B	A0A3Q1NM85	ENSBTAG00000017232	0.10450696	0.109945967	-0.005439008
Q148I9	AMACR	Q148I9		-0.082344461	-0.034423831	-0.047920631
A0A3Q1N4F3	GPHN	A0A3Q1N4F3	ENSBTAG00000004171	-0.134542123	-0.108436232	-0.026105891
A0A3Q1MKB1	CPSF1	A0A3Q1MKB1	ENSBTAG00000008355	-0.196021198	-0.13223669	-0.063784508
Q1LZH7	KANK2	bta:767586; Q1LZH7	ENSBTAG00000009568	0.073184347	0.093211401	-0.020027055
Q5E9D4	BCAS2	bta:507944; Q5E9D4	ENSBTAG00000016705	0.232334556	0.147225953	0.085108603
A0AAA9TN35	OSBPL9	A0AAA9TN35	ENSBTAG00000021170	-0.108165012	-0.076424441	-0.031740571
Q3SWX8	RBBP7	bta:537402; Q3SWX8	ENSBTAG00000002820	0.019108823	-0.097951165	0.117059988
A7MB23	PICALM	A7MB23; bta:513579	ENSBTAG00000001657	0.106019707	0.17917901	-0.073159303
A0AAF7A2L6	CYRIB	A0AAF7A2L6	ENSBTAG00000020801	-0.011183738	-0.008379666	-0.002804073
A0A3Q1MNT5	EXOC7	A0A3Q1MNT5	ENSBTAG00000007910	-0.002740162	-0.004112198	0.001372035
Q5E9X4	LRRC59	bta:532659; Q17QQ7; Q5E9X4	ENSBTAG00000006072	-0.015580011	-0.172639386	0.157059375
E1BND0	USP24	bta:532257; E1BND0	ENSBTAG00000016990	-0.31152032	-0.266479356	-0.045040964
Q3SWZ6	SBDS	bta:513237; Q3SWZ6	ENSBTAG00000004051	0.059572445	0.18679882	-0.127226375
A0AAA9S7S4	WDR26	A0AAA9S7S4	ENSBTAG00000011785	0.072342266	0.186317458	-0.113975192
G5E589	PSMB1	G5E589	ENSBTAG00000007685	0.073835881	0.076961982	-0.003126101
A0AAF6YQA7	PSMA1	A0AAF6YQA7	ENSBTAG00000006564	0.040984099	0.004611709	0.03637239
A0A3Q1MH18	PIEZO1	A0A3Q1MH18	ENSBTAG00000020944	-0.192645078	-0.2257802	0.033135123
A0AAF6ZDQ7	PRPSAP1	A0AAF6ZDQ7	ENSBTAG00000030172	-0.021927839	0.016546309	-0.038474148
A6QR19	ENO2	A6QR19; bta:526006; F1N123		0.076510232	0.085036104	-0.008525871
A0A3Q1LXA8	PPA2	A0A3Q1LXA8	ENSBTAG00000002428	0.100258648	0.158342581	-0.058083934
E1BF96	PPP1R10	bta:510825; E1BF96	ENSBTAG00000006933	-0.089580593	0.08156224	-0.171142833
A0A3Q1LXV4	DNAJC10	A0A3Q1LXV4	ENSBTAG00000008390	0.01887801	0.008743633	0.010134377
A0AAA9SEE8	MYCBP2	A0AAA9SEE8	ENSBTAG00000018851	0.034333732	0.118843256	-0.084509524
E1B8E7	HTT	E1B8E7	ENSBTAG00000001506	-0.28308097	-0.280017922	-0.003063048
F6RWK1	ARHGAP1	bta:512817; F6RWK1	ENSBTAG00000016501	-0.072731884	-0.049056358	-0.023675527
A5PKI2	ZC3H11A	A5PKI2; bta:529699; F6QB05		-0.066418865	-0.090407221	0.023988356
F1MJ55	CSNK2A2	F1MJ55	ENSBTAG00000013784	0.05166212	0.109917692	-0.058255572
A0AAA9T5P2	FAM98B	A0AAA9T5P2	ENSBTAG00000021870	0.008604568	0.049484838	-0.040880269

Accession	Gene_Symbol	Gene_ID	Ensembl_Gene ID	log2(ratio(OV24H/OV0H))	log2(ratio(OV24R/OV0H))	log2(ratio(OV24R/OV24H))
A0A3Q1MNU7	RBM26	A0A3Q1MNU7	ENSBTAG00000017573	0.17856392	0.092804749	0.085759171
A0AAF6Z704	RPL10A	A0AAF6Z704	ENSBTAG00000019494	-0.093581413	-0.152160413	0.058578999
A0A3Q1N9E9	LAMA4	A0A3Q1N9E9; bta:529670	ENSBTAG00000008817	-0.059278723	-0.261187672	0.201908948
A0AAA9SBK0	PAWR	A0AAA9SBK0	ENSBTAG00000043949	0.210172591	0.305868244	-0.095695653
A0A3Q1LN78	ARFIP1	A0A3Q1LN78	ENSBTAG00000008438	0.04620379	0.077553144	-0.031349354
A0A140T832	RPS17	A0A140T832	ENSBTAG00000000622	-0.070535802	-0.152817026	0.082281224
Q3SX47	HNRPC	bta:510852; Q3SX47		0.102978464	0.098312061	0.004666403
A0A3Q1MVT2	SRSF1	A0A3Q1MVT2	ENSBTAG00000014766	0.185002175	0.157928585	0.02707359
E1BE97	KDM3B	E1BE97	ENSBTAG00000010063	-0.242533954	-0.259425733	0.016891779
A0AAF6Z7G1				0.034948508	-0.045307628	0.080256137
G3N314	DNAAF5	G3N314	ENSBTAG00000048140	-0.102014958	-0.063815098	-0.03819986
F1MRK2	SPEN	F1MRK2	ENSBTAG00000018729	-0.091630475	-0.139724764	0.048094288
A7YW33	POLDIP3	A7YW33; bta:512693; F1N375	ENSBTAG00000015511	-0.02341418	-0.096026888	0.072612707
A0AAF6ZHS4	DDAH1	A0AAF6ZHS4	ENSBTAG00000034776	0.017277991	0.00808882	0.009189171
P79126	PPM1G	bta:286880; P79126; Q3ZBB4	ENSBTAG00000019522	-0.44519947	-0.188387244	-0.256812226
A0AAA9SB79	TP53	A0AAA9SB79	ENSBTAG00000001069	0.052372177	-0.058504845	0.110877022
A0A140T894	YWHAB	A0A140T894; bta:286863	ENSBTAG00000015846	0.20069735	0.094454575	0.106242776
A0AAF6YYL2	SLC25A4	A0AAF6YYL2	ENSBTAG00000013208	-0.197669992	-0.089754653	-0.107915339
E1BC78	ATAD2	E1BC78	ENSBTAG00000002002	-0.334671225	-0.25638003	-0.078291195
E1BG87	HAUS6	E1BG87	ENSBTAG00000019843	0.022815895	-0.04042298	0.063238875
P68399	CSNK2A1	bta:282419; P19138; P20426; P68399; Q14013; Q2KI05	ENSBTAG00000012341	0.001513052	-0.046145566	0.047658618
F1MHQ5	RNMT	F1MHQ5	ENSBTAG00000009142	0.082729896	0.055161636	0.02756826
P02584	PFN1	bta:513895; P02584; Q3ZCH4; Q5E942	ENSBTAG00000004915	0.06874759	-0.002999367	0.071746957
A4FUB9	ACIN1	A4FUB9		0.03678667	-0.124447371	0.161234041
Q2KIL3	ALAD	Q2KIL3		-0.018845341	-0.146581373	0.127736032
G3MX37	ZC3H18	G3MX37	ENSBTAG00000019520	0.050888711	-0.015315378	0.066204089
F6RZ38	CHERP	F6RZ38	ENSBTAG00000005289	0.026073512	-0.056641997	0.082715509
Q3T0S6	RPL8	B0JYM1; bta:535056; Q3T0S6	ENSBTAG00000026327	-0.158079576	-0.217038323	0.058958747
Q17QI1	ACSF2	bta:768237; Q17QI1	ENSBTAG00000021301	-0.287520215	-0.042797688	-0.244722528
F1MLE1	DIP2B	F1MLE1	ENSBTAG00000005246	0.074845363	0.152924649	-0.078079286
A0A3Q1LSQ0	TACC1	A0A3Q1LSQ0	ENSBTAG00000004238	0.020353062	-0.156389521	0.176742583
A0AAA9SDZ0	STK24	A0AAA9SDZ0	ENSBTAG00000032234	0.05226731	0.048209093	0.004058217
A0AAA9TU27	VDAC2	A0AAA9TU27	ENSBTAG00000012975	0.007402248	0.005924834	0.001477414
F1MDN4	RPLP0	F1MDN4	ENSBTAG00000017389	-0.014721506	-0.098489863	0.083768358
Q3MHP5	DRG1	bta:540161; Q3MHP5		0.102569734	0.207564107	-0.104994374
F1MBB4	GRHPR	bta:504764; F1MBB4	ENSBTAG00000019299	-0.247184354	-0.228948783	-0.018235571
A0A3Q1LK04	UCHL1	A0A3Q1LK04	ENSBTAG00000005078	0.284891571	0.068923218	0.215968353
E1BEP4	ASH2L	E1BEP4	ENSBTAG00000000978	0.089334361	0.114316717	-0.024982357
A0A452DKI9	XPNPEP1	A0A452DKI9	ENSBTAG00000004438	-0.152874549	-0.105709441	-0.047165108
A0A452DII9	SERPINE1	A0A452DII9	ENSBTAG00000014465	-0.164188632	-0.324085127	0.159896496
A0A3Q1LPF0	APOE	A0A3Q1LPF0	ENSBTAG00000010123	-0.069002788	-0.066925473	-0.002077315
A0AAA9SIE7				0.019380628	-0.105944376	0.125325003
F1MV22	PPAT	F1MV22	ENSBTAG00000010571	-0.057820654	-0.200024608	0.142203954
A0AAA9TMF7	SAMHD1	A0AAA9TMF7	ENSBTAG00000022007	0.241424853	0.167363959	0.074060894
Q3ZBG0	PSMA7	bta:505050; Q3ZBG0	ENSBTAG00000015127	0.035059291	-0.03909982	0.074159111
A0AAA9TFV5	PTPN1	A0AAA9TFV5	ENSBTAG00000006616	-0.025995209	0.024128271	-0.050123479
A0AAA9TMK4	APMAP	A0AAA9TMK4	ENSBTAG00000004278	0.012140563	0.038759308	-0.026618745
Q2HJ88	RTCA	bta:510469; Q2HJ88	ENSBTAG00000006321	0.073026471	0.075872024	-0.002845553
A0AAA9SRL1	PCDH7	A0AAA9SRL1	ENSBTAG00000000061	-0.358706499	-0.456664595	0.097958095
Q2HJ55	SAMM50	bta:618777; Q2HJ55	ENSBTAG00000045957	-0.12041383	-0.082057715	-0.038356115
A0A3Q1MCL9	SPECC1L	A0A3Q1MCL9	ENSBTAG00000021656	0.107622234	0.166778446	-0.059156212
Q0VD48	VPS4B	bta:539357; Q0VD48	ENSBTAG00000010492	0.143973907	-0.007297411	0.151271318
A0AAA9SHG7	ARFGAP2	A0AAA9SHG7	ENSBTAG00000009574	-0.018818028	-0.068598756	0.049780727
A0AAA9S251	ACTL6A	A0AAA9S251	ENSBTAG00000002460	-0.028196892	-0.024134867	-0.004062025
A0A452DJ82	ARHGDIA	A0A452DJ82	ENSBTAG00000030209	-0.003005616	0.058833638	-0.061839254
A0A3Q1NCA0	MAP4K4	A0A3Q1NCA0	ENSBTAG00000013023	0.103263853	0.129094786	-0.025830933
E1B7R0	DIS3L2	bta:519222; E1B7R0	ENSBTAG00000011964	-0.081030205	-0.07498116	-0.006049045
P07688	CTSB	bta:281105; P07688; Q3ZC03	ENSBTAG00000012442	0.079111729	0.229260367	-0.150148638
A0AAA9TFI9	NCLN	A0AAA9TFI9	ENSBTAG00000020759	-0.02987053	0.016168937	-0.046039466
A6H767	NAP1L1	A6H767; bta:790872	ENSBTAG00000061413	-0.030816017	-0.033650389	0.002834372
A0AAA9SKD1	CDK2	A0AAA9SKD1	ENSBTAG00000004021	0.038672492	-0.053752174	0.092424666
F1N1H2	MAP1S	F1N1H2	ENSBTAG00000020709	0.092012715	0.172034798	-0.080022083
Q2KJH9	ALDH9A1	bta:537539; Q2KJH9		0.099441653	0.252891981	-0.153450328
A0AAA9SIZ2	XRN1	A0AAA9SIZ2	ENSBTAG00000011524	-0.371968777	-0.269979994	-0.101988784
A0A3Q1MBT8	BPTF	A0A3Q1MBT8	ENSBTAG00000014598	0.085337357	0.006265786	0.079071571
Q2KJ80	VPS33A	bta:510197; Q2KJ80		-0.002891173	0.037067327	-0.0399585
Q3ZBG1	RAB14	bta:539180; F1MY39; Q3ZBG1		0.071240727	-0.013476232	0.084716959
A4FUA8	CAPZA1	A4FUA8; bta:534656	ENSBTAG00000014295	0.138531241	0.10437423	0.034157011
F1MTI2	CDKN2AIP	F1MTI2	ENSBTAG00000000957	0.192169177	0.308927309	-0.116758133
G3N3U9	MRPS22	G3N3U9	ENSBTAG00000047608	-0.428020723	-0.354379961	-0.073640762
E1BLG8	AMPD2	bta:514185; E1BLG8	ENSBTAG00000013018	-0.214769452	-0.263034406	0.048264954
A7MB69	DAPK3	A7MB69; bta:525506	ENSBTAG00000020417	0.122120491	-0.095676478	0.217796969
A0A3Q1M1S1	NCOA5	A0A3Q1M1S1	ENSBTAG00000014186	0.12799293	-0.057143907	0.185136837
F1MYV9	OXSR1	bta:526949; F1MYV9	ENSBTAG00000014927	0.061819177	0.095798492	-0.033979315
A0AAA9SI49	KPNA4	A0AAA9SI49	ENSBTAG00000017416	0.203007822	0.099646603	0.103361219
A0AAA9SVG0	CDR2L	A0AAA9SVG0	ENSBTAG00000039466	0.266373979	0.044444777	0.221929203
P84080	ARF1	A5D7F1; bta:338058; P10947; P32889; P84080; Q3SZG1	ENSBTAG00000007725	0.082622291	-0.048363022	0.130985312
Q0VCQ9	RCN2	bta:512717; F1MQB4; Q0VCQ9	ENSBTAG00000015780	0.474857585	0.351529292	0.123328294
F1MNY2	CEMIP2	bta:515491; F1MNY2	ENSBTAG00000005090	-0.364960627	-0.478516556	0.113555929
A0AAA9RXF3	IKBIP	A0AAA9RXF3	ENSBTAG00000021660	-0.074282882	-0.068647347	-0.005635535
A0A3Q1LRF2	UPF2	A0A3Q1LRF2	ENSBTAG00000000288	0.159131928	0.058357604	0.100774325
F2Z4H8	TIAL1	F2Z4H8	ENSBTAG00000004080	0.16855427	0.025967684	0.142586585
E1BDV7	NAA25	E1BDV7	ENSBTAG00000006666	-0.095157233	-0.150298787	0.055141554
Q2KIW9	CMPK1	bta:509965; Q2KIW9		0.144679753	0.12945693	0.015222823
A4IF71	PKP2	A4IF71; bta:537784; F1N7H1	ENSBTAG00000002651	0.010341944	-0.110201223	0.120543167
A0A3Q1MBN8	EXOC1	A0A3Q1MBN8	ENSBTAG00000032637	0.042686399	0.058026945	-0.015340546
A0AAA9U0F6	SH3GL1	A0AAA9U0F6	ENSBTAG00000006007	-0.208872615	-0.205571252	-0.003301363
A0AAA9TRI5	IGF2BP2	A0AAA9TRI5	ENSBTAG00000007666	0.084205298	0.082794349	0.001410949
A0AAA9TE19	NUP54	A0AAA9TE19	ENSBTAG00000010955	0.12592244	0.120168913	0.005753527
Q0VBZ0	CSK	bta:509246; Q0VBZ0	ENSBTAG00000021424	0.00965017	-0.035946696	0.045596866
Q58DQ3	RPL6	A3KMZ3; bta:511051; Q58DQ3	ENSBTAG00000031723	-0.131493919	-0.163394003	0.031900084
G5E5P2	CASK	G5E5P2	ENSBTAG00000020136	0.192645078	0.151606694	0.041038384
A0A3Q1M2A3	PPIP5K2	A0A3Q1M2A3	ENSBTAG00000001485	0.029515492	0.055709433	-0.026193941
A0A3Q1LPM7	MAP3K7	A0A3Q1LPM7	ENSBTAG00000002625	0.136837613	0.090819048	0.046018565
F1N4Q2	METAP2	F1N4Q2	ENSBTAG00000019944	-0.048533482	-0.220575425	0.172041942
F1MQ96	PRKCI	bta:528478; F1MQ96	ENSBTAG00000001403	-0.045142156	-0.139898607	0.094756451
A0A3Q1MNX0	RASA1	A0A3Q1MNX0	ENSBTAG00000009565	-0.002905731	0.055522497	-0.058428228
A0A3Q1MHS1	PRPS2	A0A3Q1MHS1	ENSBTAG00000047997	-0.044822198	-0.033874866	-0.010947332
Q5E946	PARK7	bta:511268; Q3ZC96; Q5E946	ENSBTAG00000020518	0.069656166	0.002795921	0.066860245
O18789	RPS2	A2V9C7; bta:286867; O18789; Q3T0W5		-0.06529146	-0.11358701	0.048295557
A0AAA9S628	CC2D1A	A0AAA9S628	ENSBTAG00000006068	0.026421178	0.016743264	0.009677914
A0AAA9S2X0	CMTR1	A0AAA9S2X0	ENSBTAG00000017091	-0.261697958	-0.042056148	-0.21964181
Q29S22	DDX47	bta:534721; Q29S22	ENSBTAG00000019608	-0.154509949	-0.212097441	0.057587492
Q0VC68	SEPTIN5	bta:615408; Q0VC68	ENSBTAG00000008320	-0.175305556	-0.181703328	0.006397771
A0A3Q1MHE5	DLST	A0A3Q1MHE5	ENSBTAG00000006463	-0.058952394	0.012624539	-0.071576933
Q2T9V5	TRMT6	bta:505816; Q2T9V5		-0.231718865	-0.07423863	-0.157480235
F1MVW4	XPO4	bta:506392; F1MVW4	ENSBTAG00000003710	-0.028044631	-0.053439259	0.025394628
Q5W9D7	QKI	bta:493722; Q5W9D7	ENSBTAG00000011593	-0.031650886	-0.010473331	-0.021177555
F1N2K1	PCYOX1	A6QPR1; bta:100125835; F1N2K1	ENSBTAG00000002783	-0.042668363	0.135208414	-0.177876776
A0A3Q1MLQ2				0.006191834	-0.023458973	0.029650807
F6QNE3	KIFC1	F6QNE3	ENSBTAG00000001631	-0.151686331	-0.307036542	0.155350211
A0A3Q1NC60	ZMYND8	A0A3Q1NC60	ENSBTAG00000013114	0.052034828	0.195102982	-0.143068155
A7YWH5	DKC1	A7YWH5; bta:525619		-0.16567803	0.110022787	-0.275700818
A0AAA9T915	GTPBP4	A0AAA9T915	ENSBTAG00000014917	-0.395724024	-0.239123917	-0.156600108
A0AAA9TSF9	RNF214	A0AAA9TSF9	ENSBTAG00000019358	0.093213162	0.17783524	-0.084622078
A0AAA9U1D0	PRPF4	A0AAA9U1D0	ENSBTAG00000004571	-0.146592498	-0.160546669	0.013954172
A5PJU0	NOL6	A5PJU0; bta:782674	ENSBTAG00000003323	-0.019567369	0.035441579	-0.055008947
A0A3Q1M4P9	FAM83H	A0A3Q1M4P9	ENSBTAG00000038682	-0.13594275	-0.131988353	-0.003954397
A0A3Q1MJU0	RAB2A	A0A3Q1MJU0	ENSBTAG00000000948	0.012084068	-0.0662156	0.078299669
A6H769	RPS7	A6H769; bta:505507	ENSBTAG00000016224	-0.169027189	-0.252154733	0.083127544
Q08E38	PRPF19	bta:513868; Q08E38	ENSBTAG00000014366	0.09567521	0.049366077	0.046309133
Q3SX41	LUC7L3	bta:539955; Q3SX41	ENSBTAG00000002279	0.153403089	-0.039441905	0.192844994
Q1RMM1	ZC3H15	bta:535102; Q1RMM1	ENSBTAG00000021762	0.101671418	0.115829481	-0.014158063
Q29466	ATP6V0A1	bta:286768; Q29466		-0.015846036	0.143093105	-0.158939141
A0AAF6Z0I1	SPARC	A0AAF6Z0I1	ENSBTAG00000014835	-0.244925025	-0.416725849	0.171800824
Q08DT0	DDX10	bta:528483; F1MQL8; Q08DT0	ENSBTAG00000002382	-0.031954685	0.087369887	-0.119324572
A0A3Q1M6A2	CD109	A0A3Q1M6A2	ENSBTAG00000013222	-0.442546561	-0.497629626	0.055083065
E1BDK6	LAMB2	E1BDK6	ENSBTAG00000022635	-0.22264906	-0.097118178	-0.125530882
Q0P5I7	UBA3	bta:512647; F1MIY7; Q0P5I7		-0.226612646	-0.033097688	-0.193514957
A6QLN5	PI4K2A	A6QLN5; bta:507860	ENSBTAG00000002010	0.192437541	0.137654441	0.0547831
F1N703	UBE3C	F1N703	ENSBTAG00000008025	0.38466385	0.288139781	0.096524069
A0AAA9S105	DNAJC2	A0AAA9S105	ENSBTAG00000003784	0.032010395	-0.009277814	0.041288208
A0A3Q1M133	FYCO1	A0A3Q1M133	ENSBTAG00000015698	-0.018505469	0.121990524	-0.140495993
F6REA4	ACY1	F6REA4	ENSBTAG00000032304	-0.247437551	-0.028277954	-0.219159596
P40673	HMGB2	bta:540444; P40673; Q32L56	ENSBTAG00000015101	0.018129804	0.142852033	-0.124722229
Q3ZBD0	PSMD7	bta:512456; Q3ZBD0	ENSBTAG00000018425	-0.023773062	-0.06480033	0.041027268
Q28009	FUS	bta:280796; Q0P5J2; Q28009		-0.150357633	-0.042374952	-0.107982681
Q3SZ56	NUP88	bta:504786; Q3SZ56		-0.001398638	-0.019705409	0.018306771
A0A7S6SNS8				-0.024641995	0.178174888	-0.202816883
F1MND5	RBM12	bta:100138139; F1MND5	ENSBTAG00000044015	-0.080454932	-0.040398101	-0.040056831
A5D9C3	ZC3HC1	A5D9C3		0.181570305	0.230130663	-0.048560358
A0AAA9RYV2	ZPR1	A0AAA9RYV2	ENSBTAG00000002650	0.260062839	0.16477039	0.09529245
A6QR33	VPS18	A6QR33; bta:518781	ENSBTAG00000011567	0.009867029	0.025237291	-0.015370262
A0A3Q1MAU7	ESD	A0A3Q1MAU7	ENSBTAG00000003415	0.059847222	0.212993723	-0.153146502
A0A140T8B2	THOC5	A0A140T8B2	ENSBTAG00000013150	0.073556607	-0.031412953	0.10496956
A0AAA9T703	ASL	A0AAA9T703	ENSBTAG00000015314	-0.274439169	-0.14662011	-0.127819059
Q3T0S7	RTRAF	bta:513083; Q3T0S7	ENSBTAG00000021944	0.406656126	0.409308139	-0.002652014
P00517	PRKACA	bta:282322; P00517	ENSBTAG00000006642	-0.020425472	0.021473067	-0.041898538
A6QQP4	SCARB2	A6QQP4; bta:533177; F1N1Q4		0.239620225	0.409699501	-0.170079276
F1MS45	CPNE2	bta:782388; F1MS45	ENSBTAG00000002233	-0.01093992	-0.084146006	0.073206086
A0AAA9T1Y3	PTBP3	A0AAA9T1Y3	ENSBTAG00000016750	-0.076379766	-0.017374027	-0.059005739
F1ML88	PUS7	F1ML88	ENSBTAG00000007743	-0.55802759	-0.334419039	-0.223608551
A0A3Q1ML81	HTATSF1	A0A3Q1ML81; bta:526910	ENSBTAG00000054942; ENSBTAG00000055212	0.16639331	0.107041176	0.059352134
A0AAF6Z657	PDIA5	A0AAF6Z657	ENSBTAG00000018877	-0.021533162	-0.056034035	0.034500873
A6QL88	SACM1L	A6QL88; bta:530577	ENSBTAG00000003501	0.08638189	0.121875951	-0.03549406
F1N2F1	NIBAN1	F1N2F1	ENSBTAG00000014548	0.020359088	0.260235772	-0.239876685
A0AAA9TUL1	RABGGTA	A0AAA9TUL1	ENSBTAG00000018796	-0.101097877	-0.108934372	0.007836494
Q3B7M5	LASP1	bta:532851; Q3B7M5	ENSBTAG00000030587	0.039285296	0.025467476	0.01381782
A0A3Q1MFG3	RPSA	A0A3Q1MFG3	ENSBTAG00000009757	0.017738277	-0.204164514	0.221902791
A0AAA9SCD4	MTHFD2	A0AAA9SCD4	ENSBTAG00000004881	-0.127727601	-0.111333163	-0.016394438
A0AAA9T3X0	PRKAA1	A0AAA9T3X0	ENSBTAG00000000013	-0.020319984	0.03839829	-0.058718275
E1BM23	ARHGAP5	E1BM23	ENSBTAG00000027024	-0.013888868	0.034148222	-0.048037091
E1BDU9	GATAD2B	E1BDU9	ENSBTAG00000006678	-0.074904809	-0.106915204	0.032010395
A0AAA9S219	H1-5	A0AAA9S219; bta:527304	ENSBTAG00000067558	-0.061067289	-0.16037449	0.0993072
A0AAA9TQ63	FXR2	A0AAA9TQ63	ENSBTAG00000005462	-0.032741401	-0.121824945	0.089083544
E1BDK7	ELAC2	bta:509256; E1BDK7	ENSBTAG00000021939	0.024134867	0.037370694	-0.013235827
Q5BIP4	TOM1	bta:504912; F1MNE8; Q5BIP4	ENSBTAG00000045785	-0.149131241	-0.060383148	-0.088748093
A0AAA9TBQ5	PRMT3	A0AAA9TBQ5	ENSBTAG00000013421	0.103198888	0.149402678	-0.04620379
Q29RZ2	PPWD1	bta:517560; Q29RZ2	ENSBTAG00000009514	0.419069253	0.357376931	0.061692322
Q3SZ71	PMPCB	bta:534546; Q3SZ71		-0.270409744	-0.26712716	-0.003282584
A3KN51	TSG101	A3KN51; bta:507659	ENSBTAG00000013563	-0.057170869	-0.017454876	-0.039715993
A0A3Q1M3K7	RAB7A	A0A3Q1M3K7	ENSBTAG00000010193	-0.035808127	-0.110206309	0.074398182
A0AAA9TSW0	ERP44	A0AAA9TSW0	ENSBTAG00000009804	0.09350526	0.177121378	-0.083616118
A0A3Q1LG54	PPP1R21	A0A3Q1LG54	ENSBTAG00000002570	0.021501069	0.082783044	-0.061281975
A5D7A0	EFHD2	A5D7A0; bta:514259	ENSBTAG00000009048	0.168224712	0.18027234	-0.012047628
A0AAF6Z1S7	SMU1	A0AAF6Z1S7	ENSBTAG00000019564	-0.078959621	-0.174403107	0.095443485
F1N0S9	FAM98A	F1N0S9	ENSBTAG00000008548	0.022520575	-0.031551248	0.054071823
G5E5T1	EPS8L2	G5E5T1	ENSBTAG00000022147	-0.034200265	0.020137646	-0.05433791
A0A3Q1LV44	ALDH3A2	A0A3Q1LV44	ENSBTAG00000039161	-0.154145367	-0.098483704	-0.055661663
A0AAA9S6D6	AFAP1	A0AAA9S6D6	ENSBTAG00000012464	-0.109549462	-0.065621495	-0.043927967
P04409	PRKCA	bta:282001; P04409		0	0.102830946	-0.102830946
F6PZZ7	VPS4A	F6PZZ7	ENSBTAG00000001659	0.10584025	-0.021216486	0.127056736
Q5EA53	GTF2F1	A6H7C0; bta:505702; Q5EA53	ENSBTAG00000021016	-0.006042711	0.013504264	-0.019546975
A0AAA9S6L2	ATP5PB	A0AAA9S6L2	ENSBTAG00000006441	-0.028428408	-0.007054758	-0.021373651
Q3SWX5	CDH6	bta:537946; Q3SWX5	ENSBTAG00000012992	-0.474800496	-0.962798796	0.4879983
A5PJR9	GNL1	A5PJR9; bta:532528; F1MWB9	ENSBTAG00000025516	0.012478939	0.121463512	-0.108984573
F1N399	SNX1	F1N399	ENSBTAG00000002014	-0.007312205	0.044526726	-0.051838932
F1N091	CCDC6	F1N091	ENSBTAG00000003189	0.170092144	0.136272147	0.033819998
A2VE06	RPS4Y1	A2VE06; bta:286873	ENSBTAG00000025617	-0.129370903	-0.146389487	0.017018584

Accession	Gene_Symbol	Gene_ID	Ensembl_Gene ID	log2(ratio(OV24H/OV0H))	log2(ratio(OV24R/OV0H))	log2(ratio(OV24H/OV24R))
Q3SZK8	NHERF1	bta:505242; Q3SZK8	ENSBTAG00000011382	0.36536234	0.443606651	-0.078244311
A2VDZ9	VAPB	A2VDZ9; bta:326580	ENSBTAG00000017424	0.171987468	0.215298639	-0.043311171
A0AAA9TE78	ADRM1	A0AAA9TE78	ENSBTAG00000003058	0.065029813	0.069262662	-0.004232849
E1BJ75	CRTAP	bta:540924; E1BJ75	ENSBTAG00000021442	-0.182060327	-0.207595419	0.025535092
Q3MI00	DNAJB1	bta:538426; Q3MI00	ENSBTAG00000016874	0.394019718	0.168599604	0.225420114
A0A3Q1MRJ9	NECTIN2	A0A3Q1MRJ9	ENSBTAG00000015318	-0.095032449	-0.013792601	-0.081239848
A0A3Q1MXH0	CUL5	A0A3Q1MXH0	ENSBTAG00000012882	0.059907844	0.136553802	-0.076645958
Q3T145	MDH1	bta:535182; Q3T145	ENSBTAG00000019295	0.01938391	-0.005586435	0.024970345
E1BPL8	MME	bta:536741; E1BPL8	ENSBTAG00000002075	-0.017961283	0.175746553	-0.193707837
P02694	RBP1	bta:537379; P02694; Q53J07; Q9TS50		-0.05264148	0.015889902	-0.068531382
A0AAF6Z2Q2	METAP1	A0AAF6Z2Q2	ENSBTAG00000016004	-0.032722321	-0.049366077	0.016643756
Q3MHX3	RBM4	A5D970; bta:767937; Q3MHX3	ENSBTAG00000031688	0.28361986	0.06166978	0.22195008
E1BH11	SH3BP1	bta:100297734; E1BH11	ENSBTAG00000030257	0.085620426	0.13569158	-0.050071155
A0AAA9SI20	ATXN10	A0AAA9SI20	ENSBTAG00000009351	0.273018494	0.073615195	0.1994033
Q17QH2	MAP2K2	bta:510434; F1MYR6; Q17QH2	ENSBTAG00000024450	-0.199720595	-0.08916222	-0.110558375
Q0IIG7	RAB5A	bta:539764; Q0IIG7		0.023083613	0.01590958	0.007174033
E1BF82	NDC80	bta:538789; E1BF82	ENSBTAG00000021673	-0.09903308	-0.40708359	0.308050511
Q3SYT5	PNN	bta:100296970; F1N3M1; Q3SYT5	ENSBTAG00000026995	0.01451578	-0.052827688	0.067343468
P12763	AHSG	A6QLF7; bta:280988; P12763		0.078891364	0.558823947	-0.479932584
A4FUG8	RABEP2	A4FUG8; bta:511736	ENSBTAG00000006542	-0.014307837	-0.095642671	0.081334834
A4IF81	HNRPDL	A4IF81; bta:534770		-0.438823752	-0.349408831	-0.089414921
A5D7P5	TUBGCP2	A5D7P5; bta:781376		-0.363244867	-0.187594209	-0.175650658
A0A3S5ZPD8	GATAD2A	A0A3S5ZPD8	ENSBTAG00000003034	-0.040971781	-0.084670324	0.043698543
A4FUY8	IK	A4FUY8; bta:100125318; F1MWA0		-0.030246287	-0.158001834	0.127755547
A0AAA9SDI5	ALDH6A1	A0AAA9SDI5	ENSBTAG00000018469	0.092397138	0.125412195	-0.033015057
Q3T0V9	DERA	bta:534558; Q3T0V9	ENSBTAG00000022167	0.065156286	0.02341418	0.041742106
A0A3Q1LPP5	EIF2B4	A0A3Q1LPP5	ENSBTAG00000019510	0.004365193	0.023129875	-0.018764682
F1MZC0	AKR7A2	F1MZC0	ENSBTAG00000012289	0.086700841	0.067055328	0.019645513
A0AAA9TUA9	USP48	A0AAA9TUA9	ENSBTAG00000006940	-0.284519272	-0.276716684	-0.007802588
E1BE68	PIK3C2A	E1BE68	ENSBTAG00000004901	0.004135775	0.055521161	-0.051385386
Q17QL4	MAGED2	bta:514372; Q17QL4	ENSBTAG00000039888	-0.285889459	-0.164973359	-0.1209161
A0A3Q1NKI3	KPNA3	A0A3Q1NKI3; bta:540719	ENSBTAG00000004850	0.027984186	-0.077392143	0.105376329
A0A3Q1LV49	RNF213	A0A3Q1LV49	ENSBTAG00000022489	-0.122360262	-0.065718264	-0.056641997
A0AAA9U1A2	NUSAP1	A0AAA9U1A2	ENSBTAG00000010774	0.109437111	-0.204325099	0.31376221
F1MIB2	GAK	F1MIB2	ENSBTAG00000019188	-0.095611912	-0.0691397	-0.026472211
A0AAA9S909	RFC2	A0AAA9S909	ENSBTAG00000018589	-0.025803755	0.05316571	-0.078969466
E1BMX5	NRP1	E1BMX5	ENSBTAG00000019866	-0.051498583	0.076440806	-0.127939389
Q3SZ99	AIP	bta:360190; F1MPM7; Q3SZ99		-0.081149067	0.035297052	-0.116446119
F1MYN2	CGN	F1MYN2	ENSBTAG00000017564	0.180421502	0.235873948	-0.055452446
A6QLS8	WDR11	A6QLS8; bta:525169	ENSBTAG00000021540	-0.029915578	-0.008097468	-0.02181811
P80311	PPIB	bta:281419; P80311; Q5E9F4	ENSBTAG00000016822	0.111963324	0.063103922	0.048859401
A0A3Q1NNK5	RBM27	A0A3Q1NNK5	ENSBTAG00000011642	0.049065559	0.023207391	0.025858168
Q58D72	ATL1	bta:535424; Q3ZBC1; Q58D72	ENSBTAG00000014312	-0.221026879	-0.281513826	0.060486947
A0AAF6ZDE8	SNX5	A0AAF6ZDE8	ENSBTAG00000003422	-0.126085317	-0.108886548	-0.017198769
A6H7A9	LOC790875	A6H7A9; bta:790875		0.11297471	-0.031597738	0.144572448
Q58CQ2	ARPC1B	bta:326600; Q0V8Q8; Q58CQ2; Q58DS1	ENSBTAG00000046248	-0.177225453	-0.208561238	0.031335785
A5D7U5	GLB1	A5D7U5		0.009805951	0.168147357	-0.158341406
A0A3Q1M2Z8	RAPH1	A0A3Q1M2Z8	ENSBTAG00000027201	0.011852422	-0.051145665	0.062998087
P12234-2	SLC25A3	P12234-2		-0.212333149	-0.223604261	0.011271112
P01017	AGT	A0A3Q1LGY9; P01017	ENSBTAG00000012393	0.00625899	0.402437462	-0.396178472
G3N1K4	MED12	G3N1K4	ENSBTAG00000021351	0.099242115	0.020402088	0.078840027
A0AAA9TFP3	PDZRN3	A0AAA9TFP3	ENSBTAG00000018644	0.04510803	-0.564811893	0.609919924
E1BMF1	TM9SF3	bta:614147; E1BMF1	ENSBTAG00000016298	0.136941727	0.099947519	0.036994207
A0A3Q1MRX3	NVL	A0A3Q1MRX3	ENSBTAG00000006718	0.011495639	0.022900402	-0.011404763
A0AAF6YIM3	LGALS3BP	A0AAF6YIM3	ENSBTAG00000001368	-0.035816179	-0.032666189	-0.00314999
F1MHD9	LARP4	F1MHD9	ENSBTAG00000017164	-0.150889227	-0.247927513	0.097038286
F1MY62	GBP1	F1MY62	ENSBTAG00000001143	-0.028396126	0.040521815	-0.068917941
F1MBI1	PSMB7	F1MBI1	ENSBTAG00000003067	-0.076706173	-0.034651547	-0.042054627
A0AAA9TVM3	SNX6	A0AAA9TVM3	ENSBTAG00000012873	0.036438011	0.100121302	-0.06368329
A6QLX2	FBL	A6QLX2; bta:538548		-0.175720023	0.006993203	-0.182713227
A0A3Q1LQH8	GNPAT	A0A3Q1LQH8	ENSBTAG00000010773	-0.047662972	-0.174957079	0.127294106
A0A3Q1LM83	PPP4R3B	A0A3Q1LM83	ENSBTAG00000000545	-0.227488867	-0.161315457	-0.06617341
F1N371	DAB2	bta:509221; F1N371	ENSBTAG00000016152	0.060387936	-0.074787649	0.135175585
A7MB35	PDHA1	A7MB35; bta:407109	ENSBTAG00000019852	0.020177882	0.05067997	-0.030502088
A7E3S8	ST13	A7E3S8; bta:510494	ENSBTAG00000006202	0.223050735	0.10086662	0.122184115
A5PKF6	SMARCE1	A5PKF6; bta:540910		-0.00532852	-0.068064275	0.062735755
A0A452DJ17	TWF1	A0A452DJ17	ENSBTAG00000020938	0.032612677	0.128873219	-0.096260542
P08760	AK3	bta:281613; P08760; Q29RI4	ENSBTAG00000017147	0.083886008	0.032031548	0.05185446
A0A3Q1MC72	CELF1	A0A3Q1MC72	ENSBTAG00000002520	0.163083628	0.130089164	0.032994464
E1BM26	PSME3	E1BM26	ENSBTAG00000019918	-0.046244185	-0.236876668	0.190632483
E1BKZ1	GSR	bta:506406; E1BKZ1	ENSBTAG00000007115	0.106128351	0.088480485	0.017647866
E1B768	LARS2	E1B768	ENSBTAG00000007315	-0.292345246	-0.275987984	-0.016357262
Q1RMW4	APPL2	bta:515605; Q1RMW4		-0.041619089	-0.004108294	-0.037510795
F1MLP7	SNRNP40	F1MLP7	ENSBTAG00000016817	-0.021673343	0.028400426	-0.050073768
E1BNE7	CAVIN1	E1BNE7	ENSBTAG00000039684	-0.007183559	-0.079795051	0.072611492
A7E3T7	MAT2A	A7E3T7; bta:527403	ENSBTAG00000020873	-0.273018494	-0.27484123	0.001822736
A5D7G6	STT3B	A5D7G6; bta:504474	ENSBTAG00000007438	0.024662054	-0.014059944	0.038721998
A0AAA9S166	TASOR	A0AAA9S166	ENSBTAG00000019748	-0.139902518	-0.131672633	-0.008229886
A0AAA9SZ05	AP1M1	A0AAA9SZ05	ENSBTAG00000013329	-0.060561246	-0.041982449	-0.018578796
A0AAA9RVE1	CRKL	A0AAA9RVE1	ENSBTAG00000065402	0.15984023	0.090512293	0.069327937
A0A3Q1LUI6	MSH3	A0A3Q1LUI6	ENSBTAG00000007684	0.219781208	0.305565911	-0.085784703
Q17QZ9	IFIT5	bta:515091; Q17QZ9	ENSBTAG00000017367	-0.059933646	-0.228520606	0.16858696
F6REL8	MICALL2	F6REL8	ENSBTAG00000003049	0.057070185	0.158064846	-0.100994661
A0AAA9RYE2	BUB3	A0AAA9RYE2	ENSBTAG00000017913	0.121655792	-0.068146536	0.189802328
A6H737	LOXL2	A6H737; bta:532684; F6RUG1		-0.344139374	-0.290219235	-0.053920139
A0A3S5ZPM2	HAT1	A0A3S5ZPM2	ENSBTAG00000010118	0.094387126	0.029586796	0.06480033
G3MWR4	ATP1B1	G3MWR4	ENSBTAG00000002688	-0.010297653	-0.014437383	0.00413973
P11017	GNB2	A5D7A9; bta:281202; P11017	ENSBTAG00000006495	0.18683478	0.192258452	-0.005423672
F1MTK4	LPCAT1	F1MTK4	ENSBTAG00000000944	-0.055186921	-0.069778146	0.014591225
F6R5L6	ELOA	F6R5L6	ENSBTAG00000026585	-0.064816453	-0.080687846	0.015871393
F6RM04	RBM15B	bta:508868; F6RM04	ENSBTAG00000006500	-0.058158731	-0.115816555	0.057657825
A0AAA9TX78	GSS	A0AAA9TX78	ENSBTAG00000003504	0.054290156	0.015520585	0.03876957
Q2TBX2	VBP1	bta:508727; Q2TBX2		0.062584537	0.023963243	0.038621294
A0AAF6DLT1	SUPV3L1	A0AAF6DLT1	ENSBTAG00000001044	0.13606155	0.138944058	-0.002882509
E1BCI2	RNF40	E1BCI2	ENSBTAG00000003421	-0.100762977	-0.153912683	0.053149707
Q3SWY5	NOSIP	bta:511234; Q3SWY5	ENSBTAG00000014450	-0.388467779	-0.447580873	0.059113094
Q3T0Q8	UTP14A	bta:505324; Q3T0Q8	ENSBTAG00000013671	-0.359939823	-0.289067908	-0.070871915
A0AAA9T1J2	P3H3	A0AAA9T1J2	ENSBTAG00000003598	-0.115477217	-0.132103536	0.016626319
Q5E995	RPS6	bta:787914; Q5E995		-0.028912692	-0.097696674	0.068783982
A0A3Q1MAH9	IRGQ	A0A3Q1MAH9	ENSBTAG00000052481	0.172180975	-0.347553903	0.519734878
P62894	CYCS	bta:100847700; bta:510767; P00006; P62894; Q2KJD4	ENSBTAG00000022613; ENSBTAG00000063185	-0.014470483	0.011472784	-0.025943267
Q9N2I2	SERPINA5	A7E3W0; bta:338050; Q9N2I2		0.188035155	0.369989458	-0.181954303
Q2KID6	PLRG1	bta:533951; Q2KID6		-0.090886796	-0.066923552	-0.023963243
A0AAF6ZNJ0	UQCRFS1	A0AAF6ZNJ0	ENSBTAG00000046786	-0.337614732	-0.274089594	-0.063525138
F1ML98	BICD1	F1ML98	ENSBTAG00000006734	-0.058893689	-0.245162204	0.186268515
E1BN25	BAZ1A	E1BN25	ENSBTAG00000020164	-0.171271427	-0.123574685	-0.047696742
Q2TBQ5	RPL7A	A5D9D0; bta:513128; Q2TBQ5	ENSBTAG00000011559	-0.031528082	-0.115703221	0.084175139
Q1RMS5	NXF1	bta:512136; Q1RMS5	ENSBTAG00000009488	0.138679314	0.046157446	0.092521868
A0AAA9S5T9	ELAVL1	A0AAA9S5T9	ENSBTAG00000000613	0.072652379	0.048328178	0.024324202
A0AAF7AP66	LRRC8C	A0AAF7AP66	ENSBTAG00000000900	-0.142985758	-0.095157233	-0.047828525
E1BIX3	CAPN7	E1BIX3	ENSBTAG00000006479	0.224533236	0.181542303	0.042990934
A0AAA9TTC5	PRC1	A0AAA9TTC5	ENSBTAG00000018643	0.022252807	-0.124851487	0.147104294
E1BKM0	FIP1L1	E1BKM0	ENSBTAG00000020653	-0.322597404	-0.450259973	0.127662569
A6QR56	ALDH16A1	A6QR56; bta:506329		-0.035382878	-0.163161299	0.127778421
A0AAA9TCR0	STK3	A0AAA9TCR0	ENSBTAG00000026234	0.023363993	0.008805897	0.014558096
A6QP36	LMAN2	A6QP36; bta:790870	ENSBTAG00000008034	0.248297863	0.261567507	-0.013269644
A0A3Q1M3W4	RPS15	A0A3Q1M3W4	ENSBTAG00000019718	-0.041733758	-0.127401909	0.08566815
F1MY06	TNRC6B	bta:528167; F1MY06	ENSBTAG00000021076	0.057407376	0.001427704	0.055979672
A7MB49	DDX20	A7MB49; bta:528433		-0.066204089	-0.072376055	0.006171967
F1MU62	CPSF7	F1MU62	ENSBTAG00000015263	-0.086618639	-0.018128344	-0.068490295
A0A3Q1MC04	TRIM47	A0A3Q1MC04	ENSBTAG00000018920	-0.179447776	-0.183274555	0.00382678
A0AAA9SMQ6	PLCG1	A0AAA9SMQ6	ENSBTAG00000017584	0.111299123	0.093335373	0.01796375
A0A3Q1LR68	ITCH	A0A3Q1LR68	ENSBTAG00000000308	-0.181762237	-0.177431984	-0.004330253
A0AAA9RWR1	UROD	A0AAA9RWR1; bta:504914	ENSBTAG00000012644	-0.336786054	-0.376148486	0.039362432
F1N5K1	CDK11	F1N5K1	ENSBTAG00000010737	0.064266396	-0.018900205	0.083166601
Q769I5	MET	A4D7R6; bta:280855; Q769I5		0.141586477	0.115211062	0.026375415
A0AAF6Z3T2	PYCR3	A0AAF6Z3T2	ENSBTAG00000016810	-0.002929331	0.090729776	-0.093659107
A0AAA9S9T4	ITPR1	A0AAA9S9T4	ENSBTAG00000020455	0.232894546	0.323497095	-0.090602549
E1BC65	RBM6	bta:100296443; E1BC65	ENSBTAG00000006328	-0.362387702	-0.256410684	-0.105977018
F1MGJ4	STAT6	bta:353105; F1MGJ4	ENSBTAG00000006335	-0.198702315	0.025702701	-0.224405016
A0A452DIC9	GNPDA2	A0A452DIC9	ENSBTAG00000000287	-0.001395931	0.035828079	-0.037224011
F2Z4E9	CPSF6	F2Z4E9	ENSBTAG00000007323	0.081239848	0.023269779	0.057970069
P61223	RAP1B	bta:327708; P09526; P61223; Q3T0M4	ENSBTAG00000008967	0.183190385	0.17714134	0.006049045
A0A3Q1MWV1	MAD1L1	A0A3Q1MWV1	ENSBTAG00000002474	-0.352753221	-0.067872113	-0.284881108
A6QQS0	DPF2	A6QQS0; bta:513235	ENSBTAG00000003077	0.003932838	-0.10479619	0.108729028
A0A3Q1MRR8	DIAPH2	A0A3Q1MRR8	ENSBTAG00000050709	-0.053558305	0.132524086	-0.18608239
A0AAF6YQN7	STT3A	A0AAF6YQN7	ENSBTAG00000007203	-0.089974513	-0.106276325	0.016301812
A0A452DID1	TPT1	A0A452DID1	ENSBTAG00000010156	-0.535992889	-0.419043875	-0.116949014
G3MZK6	MAP7D3	G3MZK6	ENSBTAG00000014170	0.230215781	0.249940434	-0.019724654
F1N4Q3	GALNT7	F1N4Q3	ENSBTAG00000016845	-0.143909411	-0.274493521	0.13058411
A0A3Q1LUG2	APP	A0A3Q1LUG2	ENSBTAG00000017753	-0.407436981	-0.133538258	-0.273898723
A0JN38	SCFD2	A0JN38; bta:535130		-0.037549738	-0.198358729	0.160808991
A5PKL2	RRAS2	A5PKL2; bta:519269	ENSBTAG00000011905	0.159931989	0.101846759	0.05808523
Q24JZ8	U2AF2	bta:507895; G5E532; Q24JZ8	ENSBTAG00000002830	-0.014781841	-0.004418671	-0.01036317
A0AAA9SZI0	AFF4	A0AAA9SZI0	ENSBTAG00000009482	-0.074000581	0.157762363	-0.231762944
A0A3Q1M245	KIF13A	A0A3Q1M245	ENSBTAG00000019217	-0.028405086	-0.034393866	0.00598878
Q24K07	VPS11	bta:540381; Q24K07		0.050860936	0.049545209	0.001315727
E1B928	ZNF598	E1B928	ENSBTAG00000001023	-0.06853908	0.016989736	-0.085528816
Q32KL2	PSMB5	bta:534640; Q32KL2	ENSBTAG00000012726	0.031026896	0.061400545	-0.030373649
F1MLE8	SEC22B	F1MLE8	ENSBTAG00000007712	0.203045007	0.166312456	0.036732551
Q0V8A7	ARFGAP1	Q0V8A7		-0.121501005	-0.265811491	0.144310485
A0AAA9SIX4	VPS39	A0AAA9SIX4	ENSBTAG00000032055	-0.033947332	-0.249941982	0.21599465
A0A3Q1LHQ2	MMUT	A0A3Q1LHQ2	ENSBTAG00000014272	-0.453500375	-0.29927156	-0.154228815
F1MUT0	SETD7	F1MUT0	ENSBTAG00000003581	0.052100649	0.102385161	-0.050284512
A6QLY3	P3H2	A6QLY3; bta:511799	ENSBTAG00000021025	0.051901102	-0.055667822	0.107568924
Q3T0D7	SAR1A	bta:517171; Q3T0D7	ENSBTAG00000016858	0.005967723	-0.156161917	0.162129641
F1MBU2	SMPD4	F1MBU2	ENSBTAG00000002130	-0.177538186	0.035455538	-0.212993723
A0AAA9TC56	MEMO1	A0AAA9TC56	ENSBTAG00000007734	0	-0.052008839	0.052008839
Q2TBX4	HSPA13	bta:505907; Q2TBX4	ENSBTAG00000012908	-0.012531632	-0.310277735	0.297746103
F1MHM7	CEBPZ	F1MHM7	ENSBTAG00000039770	-0.078418286	0.11501666	-0.193434947
F1N2H2	SCAF11	bta:538632; F1N2H2	ENSBTAG00000001810	0.13792338	0.021396293	0.116527087
Q5E987	PSMA5	bta:510155; Q5E987		0.227618722	0.182824392	0.044794331
F1MQH8	RABEP1	bta:504785; F1MQH8	ENSBTAG00000011505	0.443654543	0.432838786	0.010815757
F1MPK4	HIBCH	F1MPK4	ENSBTAG00000007787	0.026269251	0.041261965	-0.014992714
E1BMP8	SBNO1	bta:540582; E1BMP8	ENSBTAG00000020505	-0.164884385	-0.265344567	0.100460182
A0A3S5ZPB0	ALDOC	A0A3S5ZPB0	ENSBTAG00000013099	0.05976968	0.026358407	0.033411273
A0AAA9T9J6	ARF4	A0AAA9T9J6	ENSBTAG00000016069	0.040733791	0.048744334	-0.008010543
A0AAF6DM82	POLR1B	A0AAF6DM82	ENSBTAG00000014166	-0.015106892	-0.020639766	0.005532874
A7Z038	ABCD3	A7Z038; bta:526059		-0.123932328	-0.207804885	0.083872557
E1BLZ8	EIF3F	bta:513793; E1BLZ8	ENSBTAG00000004861	0.156158723	-0.051782542	0.207941265
A0AAF6YUC8	PGP	A0AAF6YUC8	ENSBTAG00000009951	0.166078279	0.132064246	0.034014032
A0AAA9TH74	PSPH	A0AAA9TH74	ENSBTAG00000013081	0.388328139	0.82890073	-0.440572591
F1MR60	TFIP11	F1MR60	ENSBTAG00000012288	0.021883118	0.13664418	-0.114761062
P13696	PEBP1	bta:431786; P13696; Q3T0H9		0.105528664	0.038291784	0.067236879
A4FUD8	XAB2	A4FUD8; F6RY07		-0.070025791	0.052830957	-0.122856748
F1MUY2				-0.323269513	2.571491861	-2.894761374
A0A3Q1LNI4	ITPRID2	A0A3Q1LNI4	ENSBTAG00000000937	-0.03863266	-0.031122517	-0.007510142
A0A3Q1M9D9	MOGS	A0A3Q1M9D9	ENSBTAG00000001844	0.018564347	-0.002671658	0.021236005
A0A140T827	IMPDH1	A0A140T827; bta:504305	ENSBTAG00000000296	-0.574126229	-0.709864268	0.135738039
A7Z023	CCDC132	A7Z023; bta:535248		0.114804475	0.157239742	-0.042435266
A0AAA9S0J8	NOP58	A0AAA9S0J8	ENSBTAG00000005517	-0.247410881	-0.001378591	-0.246032291
A0A3Q1M190	RFC4	A0A3Q1M190	ENSBTAG00000014727	-0.001471387	-0.094188275	0.092716888
A5D7H2	STRN3	A5D7H2; bta:516375	ENSBTAG00000021845	0.135799218	0.177814273	-0.042015055
F1MHA1	KMT2A	F1MHA1	ENSBTAG00000018093	-0.074382196	-0.042673337	-0.03170886
E1BJP2	RACGAP1	E1BJP2	ENSBTAG00000012784	-0.030072152	-0.208523637	0.178451485
A0AAA9SAB4	LUC7L2	A0AAA9SAB4; bta:516355	ENSBTAG00000017770	0.05189073	-0.078410249	0.130300979
F1MFJ6	TBL1XR1	bta:614346; F1MFJ6	ENSBTAG00000021910	-0.095275288	-0.089737105	-0.005538184
G3MY03	TSNAX	G3MY03	ENSBTAG00000047434	-0.214538329	-0.141051863	-0.073486465
A0A3Q1LS42	HERC4	A0A3Q1LS42	ENSBTAG00000014029	-0.121513349	0.04930292	-0.170816269

Accession	Gene_Symbol	Gene_ID	Ensembl_Gene ID	log2(ratio(OV24H/OV0H))	log2(ratio(OV24R/OV0H))	log2(ratio(OV24H/OV24R))
A0A3Q1MDE2	AFG2A	A0A3Q1MDE2	ENSBTAG00000000125	0.036232317	0.029058284	0.007174033
F1MMS4	TERF2	F1MMS4	ENSBTAG00000007617	0.087843349	0.129837438	-0.041994089
E1B8Q9	IPO8	bta:523408; E1B8Q9	ENSBTAG00000014106	0.179995942	0.107814484	0.072181457
A0AAA9TLY4	PPIL2	A0AAA9TLY4	ENSBTAG00000021121	0.020844254	-0.025722565	0.046566818
Q32PD5	RPS19	bta:516063; Q32PD5	ENSBTAG00000011963	-0.112241265	-0.161311871	0.049070607
A0A3Q1MD85	LRPAP1	A0A3Q1MD85	ENSBTAG00000000073	-0.027730956	-0.092531286	0.06480033
A0AAA9TJK9	CAB39	A0AAA9TJK9	ENSBTAG00000040055	0.155495384	-0.015282929	0.170778314
A0AAA9RSV1	PTCD3	A0AAA9RSV1	ENSBTAG00000006482	-0.407466617	-0.302964691	-0.104501926
Q9BGI2	PRDX4	bta:281999; Q32L06; Q9BGI2	ENSBTAG00000006165	-0.033097688	-0.084213004	0.051115316
E1BPB3	EIF2B5	E1BPB3	ENSBTAG00000011414	0.153049229	0.191523376	-0.038474148
Q1JPH6	EIF4H	A5D959; bta:517409; Q1JPH6		-0.038044873	-0.060459022	0.022414149
F1MM78	ZCCHC8	bta:539598; F1MM78	ENSBTAG00000006114	-0.130161548	0.149860211	-0.28002176
F1MGU0	DAXX	F1MGU0	ENSBTAG00000016830	0.062388692	0.063807969	-0.001419277
Q07537	GALNT1	bta:282241; Q07537	ENSBTAG00000011206	0.183561162	0.055931275	0.127629887
Q3SZ73	ABHD11	bta:510109; Q3SZ73	ENSBTAG00000010339	-0.077634224	-0.097358877	0.019724654
A0AAA9S5H4	NUP85	A0AAA9S5H4	ENSBTAG00000016126	0.021760519	0.028941694	-0.007181175
A0AAF6ZAT9	NIFK	A0AAF6ZAT9	ENSBTAG00000019387	-0.156039475	-0.160710915	0.00467144
E1BK00	CUL7	bta:504558; E1BK00	ENSBTAG00000012749	-0.244859761	-0.178480922	-0.06637884
E1BLF0	PRRC1	E1BLF0	ENSBTAG00000021977	0.02521999	0.004482743	0.020737247
A0A3Q1LWU4	TNS3	A0A3Q1LWU4	ENSBTAG00000009655	0.142719853	0.048255169	0.094464684
Q08DF7	PDE12	bta:538860; Q08DF7	ENSBTAG00000015563	0.084268917	0.087675518	-0.003406601
F6RDI0	RAD18	F6RDI0	ENSBTAG00000012142	-0.025065337	-0.08272013	0.057654792
F1N6W9	COL18A1	F1N6W9	ENSBTAG00000023907	-1.056528094	-0.553095479	-0.503432615
F1MZT2	CRNKL1	F1MZT2	ENSBTAG00000008472	0.004432246	-0.1462602	0.150692446
E1BKT9	DSP	E1BKT9	ENSBTAG00000015106	-0.136502	1.29018955	-1.42669155
F1N338	HADH	F1N338	ENSBTAG00000002049	0.217170354	0.14919356	0.067976794
A0A3Q1NNP6	ATP6V1E1	A0A3Q1NNP6	ENSBTAG00000014238	-0.013584799	-0.064991024	0.051406225
P13621	ATP5PO	bta:281640; P13621; Q3T0Y7	ENSBTAG00000018278	-0.037474705	-0.047732611	0.010257905
G5E543	NDUFA10	G5E543	ENSBTAG00000003279	-0.213403638	-0.178970141	-0.034433497
A0AAA9RVW5	STK4	A0AAA9RVW5	ENSBTAG00000003257	0.058949661	-0.111983161	0.170932822
A0AAA9TNW6	LBR	A0AAA9TNW6	ENSBTAG00000008453	-0.043404684	-0.069756011	0.026351327
A0AAA9TV32	INTS1	A0AAA9TV32	ENSBTAG00000000642	-0.227375829	-0.056526675	-0.170849154
A5PJN8	SF3A2	A5PJN8; bta:789082		0.005765023	0.048283671	-0.042518648
A0AAA9T5S5	EPB41L3	A0AAA9T5S5	ENSBTAG00000019251	0.058279646	0.131911676	-0.07363203
G3N131	H1-1	bta:618164; G3N131	ENSBTAG00000059303	0.371221267	0.13837513	0.232846137
A0A3Q1LXP4	APOH	A0A3Q1LXP4	ENSBTAG00000001915	-0.321285326	0.086100737	-0.407386063
A6QQX8	USP39	A6QQX8; bta:532985	ENSBTAG00000010691	-0.19079618	-0.251168798	0.060372618
A0AAA9TDE9	MLEC	A0AAA9TDE9	ENSBTAG00000020050	0.114499119	0.04843286	0.066066259
Q3MHW6	SLC16A1	bta:505775; Q3MHW6	ENSBTAG00000015107	0.288396058	0.298360445	-0.009964386
A0AAF6DMI1	RBM45	A0AAF6DMI1	ENSBTAG00000006168	-0.210784178	-0.012213364	-0.198570813
A0A3Q1LZ07	SDHB	A0A3Q1LZ07	ENSBTAG00000008314	0.10713585	0.094170955	0.012964895
A0A3Q1MSI2	NEMF	A0A3Q1MSI2	ENSBTAG00000004934	-0.072077678	-0.052988305	-0.019089373
A0AAA9T405	RUFY1	A0AAA9T405; bta:528153	ENSBTAG00000019868	-0.293731203	-0.131063756	-0.162667447
A0A3Q1LMC7	APC	A0A3Q1LMC7	ENSBTAG00000018852	-0.08265118	-0.177576438	0.094925257
A0A3Q1LYY4	CRAT	A0A3Q1LYY4	ENSBTAG00000001932	-0.426837786	-0.324398162	-0.102439625
F1MM88	NEK9	F1MM88	ENSBTAG00000016612	0.075407403	0.07139434	0.004013063
A0AAA9TMJ0	WDR62	A0AAA9TMJ0	ENSBTAG00000058132	-0.301274179	-0.336356638	0.035082459
F2Z4I5	ARL8B	F2Z4I5	ENSBTAG00000012902	0.054819168	0.078077914	-0.023258745
Q2TBV1	RFC3	bta:515602; Q2TBV1	ENSBTAG00000010787	0.071456805	-0.010503286	0.081960091
Q3T0B6	C1QBP	bta:518321; Q3T0B6	ENSBTAG00000012739	0.277654195	0.196047426	0.081606769
A0AAA9TTA6	SFXN1	A0AAA9TTA6	ENSBTAG00000014536	0.130556448	0.085176545	0.045379904
Q1RMR2	SNRNP70	bta:535113; Q1RMR2	ENSBTAG00000048800	-0.007589155	-0.033695046	0.026105891
F1MYW2	CAMSAP1	F1MYW2	ENSBTAG00000006008	0.061061526	0.013495842	0.047565683
Q58DH8	FEN1	bta:616242; Q3MHF6; Q58DH8; Q58DS0	ENSBTAG00000000064	-0.528850621	-0.509328063	-0.019522558
Q3SZM5	CMAS	bta:100336710; Q3SZM5	ENSBTAG00000010136	0.057387482	0.122856748	-0.065469266
A0AAA9T1Q1	BLVRA	A0AAA9T1Q1	ENSBTAG00000013414	-0.026099718	-0.119794513	0.093694795
F1MS25	NDUFS3	F1MS25	ENSBTAG00000018483	-0.180261353	-0.154077422	-0.026183932
Q2HJ56	PWP1	bta:514147; Q2HJ56	ENSBTAG00000004654	0.175509846	-0.023896116	0.199405963
Q3SYR7	RPL9	bta:282884; Q3SYR7; Q56K06	ENSBTAG00000014518	0.005943139	-0.0843025	0.09024564
A0AAA9S2E8	CALM1	A0AAA9S2E8	ENSBTAG00000025644	0.754754908	-0.109671596	0.864426503
E1B7Z4	LOC511531	E1B7Z4	ENSBTAG00000017670	-0.461652859	-0.375629115	-0.086023744
A0AAA9S913	SUCLG1	A0AAA9S913	ENSBTAG00000006075	-0.041027268	-0.052167826	0.011140558
A0AAF7AAJ5	BDH1	A0AAF7AAJ5	ENSBTAG00000000448	-0.126114946	-0.223249129	0.097134183
A0A3Q1MFL7	DSTN	A0A3Q1MFL7	ENSBTAG00000015434	-0.132450296	0.023781226	-0.156231522
A0AAA9TUR4	ZZEF1	A0AAA9TUR4	ENSBTAG00000016316	-0.098535538	-0.13498451	0.036448971
A0AAA9TW00	CIP2A	A0AAA9TW00	ENSBTAG00000017140	-0.080892981	-0.008453315	-0.072439665
A0AAA9TPD6	ACSL1	A0AAA9TPD6	ENSBTAG00000004344	-0.07745614	0.265764199	-0.343220339
Q08DN8	FLOT1	bta:532573; Q08DN8	ENSBTAG00000009960	0.523252929	0.504589084	0.018663844
Q3T0F4	RPS10	bta:540965; Q3T0F4	ENSBTAG00000015598	-0.105905659	-0.175039651	0.069133992
A0A3Q1MPE4	TRNT1	A0A3Q1MPE4	ENSBTAG00000019534	0.121208007	0.161266259	-0.040058252
A0A3Q1MUH0	MIA2	A0A3Q1MUH0	ENSBTAG00000006819	0.131322162	0.176259567	-0.044937405
Q3ZC35	CCN1	bta:508941; Q3ZC35	ENSBTAG00000009844	-0.227177484	-0.345103208	0.117925725
A0AAA9SSZ3	ERLEC1	A0AAA9SSZ3	ENSBTAG00000008068	-0.095994475	0.073359953	-0.169354428
A0AAA9SV13	BRD2	A0AAA9SV13	ENSBTAG00000011074	0.054664976	-0.08453351	0.139198486
A0A0K1L4Y9				-0.27270479	-0.09170454	-0.18100025
A0AAA9S956	PPP1R8	A0AAA9S956	ENSBTAG00000003866	-0.053965358	-0.046748259	-0.007217099
A0A3Q1N9R6	RFC5	A0A3Q1N9R6	ENSBTAG00000007137	-0.05166212	-0.060707259	0.00904514
Q9N0F3	SARS2	A5D7G7; bta:282060; Q58CP7; Q58DN2; Q9N0F3		-0.019628807	0.028216242	-0.047845049
A0AAA9SZA4	DNAJC8	A0AAA9SZA4	ENSBTAG00000018385	-0.04935253	0.019840912	-0.069193442
F1N2P6	FKBP8	F1N2P6	ENSBTAG00000016264	-0.112474729	-0.060007309	-0.05246742
P38408	GNA14	bta:281789; P38408		-0.063078683	0.015347964	-0.078426647
Q3ZCK9	PSMA4	bta:510423; Q3ZCK9	ENSBTAG00000014440	0.062951906	0.020177882	0.042774024
A0A3Q1MPB6	RANBP9	A0A3Q1MPB6	ENSBTAG00000021556	-0.026445252	0.007260685	-0.033705937
F1MSB5	GORASP2	bta:513771; F1MSB5	ENSBTAG00000013218	0.025788681	0.085590869	-0.059802187
A0AAA9TTJ9	YME1L1	A0AAA9TTJ9	ENSBTAG00000016445	-0.225049319	-0.25008587	0.025036551
Q08E52	PAK1	bta:533729; Q08E52	ENSBTAG00000010191	0.051178724	-0.08813091	0.139309634
E1BCI8	SLC12A4	E1BCI8	ENSBTAG00000018045	-0.183877186	-0.19609055	0.012213364
A0AAF6ZWM0	AASS	A0AAF6ZWM0	ENSBTAG00000012128	0.031057257	0.136075819	-0.105018563
A0A3Q1M6K7	PFKM	A0A3Q1M6K7	ENSBTAG00000000286	-0.024779722	-0.064192379	0.039412657
A0AAA9TM22	MASTL	A0AAA9TM22	ENSBTAG00000017271	-0.035425096	0.017391603	-0.052816698
E1BM34	ANAPC7	bta:535072; E1BM34	ENSBTAG00000016675	-0.07863251	-0.147600795	0.068968285
A0AAF6YQE1	ASRGL1	A0AAF6YQE1	ENSBTAG00000006910	0.232017933	0.251481231	-0.019463297
Q3SZC1	GRPEL1	bta:515580; Q3SZC1; Q9T2U7	ENSBTAG00000010181	0.152851488	0.084064265	0.068787223
Q5EAD0	BAIAP2	bta:507837; Q5EAD0	ENSBTAG00000019044	-0.174669381	-0.244120167	0.069450786
Q3T0W4	PPP1R7	bta:505297; Q3T0W4	ENSBTAG00000000789	0.040167432	0.00524617	0.034921262
A0A3Q1LXS9	WDR70	A0A3Q1LXS9	ENSBTAG00000033095	-0.001441974	0.056528094	-0.057970069
Q3MI06	DNM2	Q3MI06		-0.048809305	0.10514815	-0.153957456
A0AAA9SKW7	GOLM1	A0AAA9SKW7	ENSBTAG00000031569	-0.293966265	-0.350132976	0.056166711
A0AAA9TKY6	THOC1	A0AAA9TKY6	ENSBTAG00000019215	-0.002962414	0.035082459	-0.038044873
F1N4H9	FUT8	F1N4H9	ENSBTAG00000019819	0.031194622	-0.061194901	0.092389522
P80513	MANF	A6QPB9; bta:540432; P80513		0.103590888	0	0.103590888
F1MJ58	ECT2	F1MJ58	ENSBTAG00000023814	-0.147047296	-0.17606337	0.029016074
E1BHR5	HEATR6	E1BHR5	ENSBTAG00000009635	-0.039158376	-0.01877139	-0.020386986
E1BNM6	WBP11	bta:534416; E1BNM6	ENSBTAG00000014759	-0.005811468	-0.021914776	0.016103308
E1BLI8	MTCL2	E1BLI8	ENSBTAG00000021669	-0.136189926	0.265951889	-0.402141815
E1BDV5	PELP1	bta:787413; E1BDV5	ENSBTAG00000018315	-0.22881869	-0.106915204	-0.121903487
A0A3Q1MW62				0.220898175	0.096046687	0.124851487
P62833	RAP1A	A4IFG1; bta:282031; P10113; P62833	ENSBTAG00000050904	0.337813617	0.329723717	0.0080899
F1N1K2	TRIP4	F1N1K2	ENSBTAG00000014493	-0.049615035	0.00157242	-0.051187455
E1BC36	CDK6	bta:511754; E1BC36	ENSBTAG00000044023	0.047085136	0.135637567	-0.088552431
F1MT69	VPS36	F1MT69	ENSBTAG00000044307	-0.298799625	-0.117716238	-0.181083386
A0A3Q1N590	MAP3K20	A0A3Q1N590	ENSBTAG00000001083	0.232446086	0.200609999	0.031836087
F6QK60	SIRT2	F6QK60	ENSBTAG00000011776	0.006403451	0.064018721	-0.057615271
F1MNI4	RAB5B	F1MNI4	ENSBTAG00000014129	0.261813335	0.147455472	0.114357863
A0AAA9TXP4	MCM3AP	A0AAA9TXP4	ENSBTAG00000003148	0.010703669	0.287132729	-0.276429061
A0AAA9S5Q9	FHL1	A0AAA9S5Q9	ENSBTAG00000018513	0.019856312	0.017778997	0.002077315
E1BAD8	MID1	E1BAD8	ENSBTAG00000010152	0.089472446	0.097482989	-0.008010543
F1MXS4	POLA2	F1MXS4	ENSBTAG00000011455	-0.187547571	-0.04354541	-0.144002161
A0AAA9S6M2	CHCHD3	A0AAA9S6M2	ENSBTAG00000024723	0.013162033	-0.026689878	0.039851911
A0AAF7AP21	GNAS	A0AAF7AP21	ENSBTAG00000052413	-0.192280347	0.052649797	-0.244930144
A0AAF6YHC2	CYP20A1	A0AAF6YHC2	ENSBTAG00000000851	-0.121405947	0.008494794	-0.129900741
A0AAF6ZIP1	DDX27	A0AAF6ZIP1	ENSBTAG00000006671	0.00293828	-0.058526077	0.061464357
O18739	CCN2	bta:281103; O18739; Q2HJ71; Q9GL71	ENSBTAG00000006367	-0.523682346	-0.546006893	0.022324547
Q3T0T7	SAR1B	bta:515999; Q3T0T7	ENSBTAG00000021226	0.037051216	-0.099428446	0.136479661
F2Z4E0	VGF	F2Z4E0	ENSBTAG00000009868	-0.244772199	-0.253874407	0.009102207
A0A452DIQ6	ETFDH	A0A452DIQ6	ENSBTAG00000016679	0.10514815	0.109198771	-0.00405062
F1MC71	EXOC5	F1MC71	ENSBTAG00000013042	-0.099822748	-0.10701825	0.007195501
Q2KJ14	PAF1	bta:528744; Q2KJ14	ENSBTAG00000020428	0.176308215	0.131959588	0.044348626
A0AAA9TRE2	VPS35L	A0AAA9TRE2	ENSBTAG00000019412	-0.147994014	0.00643774	-0.154431753
Q58DV0	PELO	bta:528728; Q1JPI6; Q58DV0		-0.018015157	0.160778728	-0.178793885
E1BE83	FANCD2	E1BE83	ENSBTAG00000010077	-0.193391107	-0.049569859	-0.143821248
A0A3Q1M8V3	RASAL2	A0A3Q1M8V3	ENSBTAG00000015537	-0.147557188	-0.196172451	0.048615263
A0AAF6YU11	PSMA6	A0AAF6YU11	ENSBTAG00000009683	0.078094324	0.125240104	-0.04714578
A2VDS0	PRPSAP2	A2VDS0; bta:506111	ENSBTAG00000014858	0.024637279	0.152441536	-0.127804256
P62871	GNB1	bta:281201; P04697; P04901; P62871; Q3MHF1	ENSBTAG00000000215	0.239095641	0.311621899	-0.072526259
Q9MZ08	BCAM	bta:282862; Q9MZ08	ENSBTAG00000009495	-0.202492864	0.138544054	-0.341036918
Q3MHY1	CSRP1	bta:615329; Q3MHY1	ENSBTAG00000016057	-0.062304659	-0.049339763	-0.012964895
A0A3Q1MUY5	IFI16	A0A3Q1MUY5; bta:506759	ENSBTAG00000011511	-0.227098028	-0.231604107	0.004506079
E1BF14	POP1	bta:511306; E1BF14	ENSBTAG00000012596	-0.160545191	-0.182124365	0.021579174
A3KMY8	GUSB	A3KMY8; bta:515687	ENSBTAG00000000704	0.032525904	0.273180531	-0.240654628
A0A3Q1M6Y9	ARHGAP12	A0A3Q1M6Y9	ENSBTAG00000012677	0.03542949	0.031535573	0.003893916
E1BEW3	IPO11	E1BEW3	ENSBTAG00000018616	0.129785054	-0.009618003	0.139403057
A0AAA9S5G9	RECQL	A0AAA9S5G9	ENSBTAG00000021083	0.147794938	0.075789223	0.072005714
A0A3Q1LVU1	FTH1	A0A3Q1LVU1	ENSBTAG00000011184	0.076732198	0.1379421	-0.061209901
E1B738	SHCBP1	bta:523367; E1B738	ENSBTAG00000033441	-0.325826794	-0.308189716	-0.017637078
A5PJM5	MCMBP	A5PJM5; bta:514863		0.08638189	-0.097921695	0.184303585
F1N2B8	PUM2	F1N2B8	ENSBTAG00000011155	-0.10120377	-0.116936697	0.015732926
F1MMH3	LUC7L	F1MMH3	ENSBTAG00000016568	0.275130243	0.02463398	0.250496263
E1BEA2	EDC3	E1BEA2	ENSBTAG00000007308	-0.027711681	0.01874763	-0.046459311
O62830	PPM1B	bta:281995; O62830; Q2T9W5	ENSBTAG00000000223	0.1901409	0.128094762	0.062046138
Q1JP79	ARPC1A	A6QLF5; bta:508402; Q1JP79	ENSBTAG00000004242	-0.045682815	-0.071701465	0.02601865
Q0II26	AIMP2	bta:520979; Q0II26	ENSBTAG00000007367	0.137786156	0.08453351	0.053252646
Q32LN7	SKIC8	bta:513208; Q32LN7		-0.077755158	0.002965458	-0.080720616
Q3ZBS7	VTN	bta:507525; Q3ZBS7	ENSBTAG00000016151	0.026904184	0.132867114	-0.10596293
E1BJE7	MCM3AP	E1BJE7	ENSBTAG00000003148	0.062996694	0.134573626	-0.071576933
E1B8X4	NAE1	E1B8X4	ENSBTAG00000010132	-0.117436603	0.107138964	-0.224575567
A0AAA9SEL9	TRANK1	A0AAA9SEL9	ENSBTAG00000021452	-0.001524242	0.072781317	-0.074305559
F1MJQ1	RAB7A	F1MJQ1	ENSBTAG00000010193	-0.001441974	-0.166326359	0.164884385
A7MBI6	GLOD4	A7MBI6; bta:512334; E1BBM1		0.174573505	0.185142747	-0.010569242
A0A3Q1MKW0	VIRMA	A0A3Q1MKW0	ENSBTAG00000003789	-0.111989436	-0.019587805	-0.092401631
A0AAA9SWL8	SERPINB8	A0AAA9SWL8	ENSBTAG00000001207	-0.29619545	-0.19103722	-0.10515823
A7MB32	STK38L	A7MB32; bta:514787	ENSBTAG00000011798	-0.043647885	0.008335859	-0.051983744
A0A3Q1LKW6	MPDZ	A0A3Q1LKW6	ENSBTAG00000043961	-0.25931132	-0.189630411	-0.069680909
Q0P5H6	TBL2	bta:511758; F1MET7; Q0P5H6	ENSBTAG00000000321	0.039057476	0.046743669	-0.007686193
A0A3Q1MLS9	L1CAM	A0A3Q1MLS9	ENSBTAG00000013462	0.015409439	0.071771882	-0.056362443
Q32LE9	CSRP2	bta:539381; Q32LE9		0.115477217	0.063796651	0.051680567
A6QP40	LRRFIP1	A6QP40; bta:616994		-0.130634675	-0.256962583	0.126327907
E1BAX1	GOPC	bta:541186; E1BAX1	ENSBTAG00000016046	0.221916363	0.16807278	0.053843583
A0AAA9SWP3	ACOT7	A0AAA9SWP3	ENSBTAG00000021202	-0.177061183	-0.335703474	0.158642291
Q32KY4	CDK4	bta:510618; Q32KY4	ENSBTAG00000007160	-0.094454575	-0.124919855	0.030465281
A0AAA9SHQ2	NAA35	A0AAA9SHQ2	ENSBTAG00000001016	0.068781011	0.115590109	-0.046809098
E1BIW0	UBR1	bta:522464; E1BIW0	ENSBTAG00000021087	-0.133099136	-0.078651352	-0.054447784
F1MLD0	PANK4	F1MLD0	ENSBTAG00000002479	-0.155541083	-0.14751271	-0.008028374
Q3SYS6	CHP1	bta:512662; Q3SYS6	ENSBTAG00000027173	0.19018384	0.05626822	0.133915621
F1MWF0	HIP1	F1MWF0	ENSBTAG00000000781	-0.025671408	-0.008933125	-0.016738283
F1N1A2	DHX57	F1N1A2	ENSBTAG00000014679	-0.344537191	-0.447247112	0.102709921
A0A3Q1NHI6	FAM114A1	A0A3Q1NHI6	ENSBTAG00000033160	-0.233275262	-0.240088474	0.006813212
A0A3Q1MVR5	EHMT1	A0A3Q1MVR5	ENSBTAG00000012148	-0.39849119	-0.267907081	-0.13058411
Q29RK4	RAD23B	bta:530189; Q29RK4	ENSBTAG00000001926	0.021593594	-0.092656225	0.114249819
E1BEG4	ZFYVE16	E1BEG4	ENSBTAG00000021577	-0.019973024	0.007912424	-0.027885448
A0AAF6DM61	VASN	A0AAF6DM61	ENSBTAG00000046339	0.03353399	0.150224882	-0.116690892
Q3SZ18	HPRT1	bta:281229; Q3SZ18	ENSBTAG00000014685	-0.046402232	-0.02446524	-0.021936992
A0AAA9S9P9	EP300	A0AAA9S9P9	ENSBTAG00000016198	-0.117476469	-0.205800135	0.088323666
F1MXZ0	GNS	F1MXZ0	ENSBTAG00000017465	0.04467859	0.220155338	-0.175476747
Q9N181	NMT2	bta:282049; Q9N181		-0.128779371	-0.116245017	-0.012534354
A0AAA9TM77	MRI1	A0AAA9TM77	ENSBTAG00000002524	0.031961764	0.001292159	0.030669606
F1MNI5	PTGS2	F1MNI5	ENSBTAG00000014127	-0.129230102	-0.185887712	0.05665761
A0AAF7APW4	ILVBL	A0AAF7APW4	ENSBTAG00000015186	-0.048094288	-0.019609749	-0.028484539
A5PKD6	GNB4	A5PKD6; bta:525962		0.173104035	0.212668536	-0.039564501

Accession	Gene_Symbol	Gene_ID	Ensembl_Gene ID	log2(ratio(OV24H/OV0H))	log2(ratio(OV24R/OV0H))	log2(ratio(OV24H/OV24R))
A4FV84	MRTO4	A4FV84; bta:509932	ENSBTAG00000011951	-0.234205753	-0.237669608	0.003463855
A0AAA9TWE2	CDK13	A0AAA9TWE2	ENSBTAG00000001528	-0.014781841	-0.127537691	0.112755851
A0A3Q1M9W0	NDRG1	A0A3Q1M9W0	ENSBTAG00000000711	0.003072834	-0.159198595	0.162271429
Q3ZCA7	GNAI3	bta:539521; Q3ZCA7		0.263875873	0.348283992	-0.084408119
G8JKZ0	DHODH	G8JKZ0	ENSBTAG00000019887	-0.012407392	-0.012407392	0
G3N093	CDCP1	bta:540622; G3N093	ENSBTAG00000012482	-0.361838768	-0.088423251	-0.273415517
Q2TBV3	ETFB	bta:617210; Q2TBV3		-0.067824242	0.063193826	-0.131018068
A0AAF7ANF9	ANP32B	A0AAF7ANF9	ENSBTAG00000021367	0.075288127	0.076917371	-0.001629244
A0A3Q1LXG3	CAPZA2	A0A3Q1LXG3	ENSBTAG00000004072	0.254348063	0.225881407	0.028466655
A0AAA9TAD0	MRPS9	A0AAA9TAD0	ENSBTAG00000006150	-0.228682901	-0.162340406	-0.066342495
A0AAF7A1X1	HSD3B1	A0AAF7A1X1	ENSBTAG00000006769	-0.383462962	-0.3594841	-0.023978863
Q0VCW8	PDCL3	bta:514033; Q0VCW8		0.220084277	-0.023675527	0.243759804
A0A3Q1LVA2	BCAT1	A0A3Q1LVA2	ENSBTAG00000013825	-0.241957657	-0.11402771	-0.127929948
Q5E951	TBCB	bta:530447; Q3ZCC1; Q5E951		0.15292088	0.112245191	0.040675689
A0A3Q1LX22	PCM1	A0A3Q1LX22; bta:525337	ENSBTAG00000007236	-0.439363337	-0.183654929	-0.255708409
A0A3Q1N0U6	ANAPC1	A0A3Q1N0U6; bta:535565	ENSBTAG00000027991	-0.378511623	-0.366538982	-0.011972642
A0AAF6YHD2	ERAP2	A0AAF6YHD2	ENSBTAG00000039275	-0.307409818	-0.107145464	-0.200264354
A2VE08	KPNA1	A2VE08; bta:539679	ENSBTAG00000011143	0.13081921	0.01298272	0.11783649
A0A452DI44	PSMD4	A0A452DI44	ENSBTAG00000006101	0.007455805	-0.012010018	0.019465823
A0A3Q1MMQ9	SUB1	A0A3Q1MMQ9	ENSBTAG00000021965	-0.001892059	-0.007583171	0.005691112
A0AAA9TWW9	SFXN3	A0AAA9TWW9	ENSBTAG00000005015	-0.099439007	-0.160146267	0.060707259
Q1RMM9	NAGA	Q1RMM9		0.102031686	0.243271151	-0.141239465
E1BNI8	NT5DC2	bta:514296; E1BNI8	ENSBTAG00000014784	-0.224399078	-0.242400854	0.018001776
F1MZS1	WWC2	F1MZS1	ENSBTAG00000012191	0.184616704	0.209756266	-0.025139562
E1BDW9	EXOC2	bta:513431; E1BDW9	ENSBTAG00000020215	0.052517687	0.126586127	-0.074068439
A0A3Q1NF64	SERPINB5	A0A3Q1NF64	ENSBTAG00000002019	-0.333752403	-0.292918686	-0.040833718
F1N226	APLP2	bta:785005; F1N226	ENSBTAG00000016168	-0.167509259	-0.112351732	-0.055157527
A0AAA9T2N9	EPM2AIP1	A0AAA9T2N9; bta:540067	ENSBTAG00000055342	-0.269749474	-0.269749474	0
A6QLP2	AHCYL2	A6QLP2; bta:532836		0.419781083	0.270215581	0.149565502
F1N1B8	KANK1	bta:534869; F1N1B8	ENSBTAG00000010976	-0.051698188	-0.073393259	0.021695071
F6RNH4	PPP1R13L	F6RNH4	ENSBTAG00000020689	-0.273696102	-0.243868536	-0.029827567
A0AAA9SSL4	NAA50	A0AAA9SSL4	ENSBTAG00000007784	0.067259754	-0.043893826	0.11115358
A0AAF6YVY1	CTSH	A0AAF6YVY1	ENSBTAG00000010992	-0.22981705	-0.085814889	-0.144002161
Q17QE2	LMCD1	bta:540474; Q17QE2	ENSBTAG00000005431	-0.037519007	0.005041455	-0.042560462
E1BMF2	ZMPSTE24	E1BMF2	ENSBTAG00000044178	0.13719196	0.204164514	-0.066972554
A4IFN6	SNX18	A4IFN6; bta:538366	ENSBTAG00000027924	0.013999307	0.041596866	-0.027597559
G5E5A1	CAMSAP2	G5E5A1	ENSBTAG00000008153	0.032263849	-0.021437171	0.05370102
Q3SWX2	ACOT9	bta:527845; Q3SWX2	ENSBTAG00000011258	-0.328805102	-0.144183589	-0.184621513
A0A452DIL6	GPC1	A0A452DIL6	ENSBTAG00000015996	-0.443878627	-0.531572688	0.087694061
A4IFK3	LMOD1	A4IFK3; bta:532090	ENSBTAG00000021576	-0.324726728	-0.306170909	-0.018555819
P05131	PRKACB	bta:282323; P05131; P24256		0.219708337	0.26838764	-0.048679304
A0A3Q1LPH8	TAOK3	A0A3Q1LPH8; bta:534620	ENSBTAG00000012550	0.012371925	0.321651955	-0.30928003
F2Z4J7	CBX5	bta:538885; F2Z4J7	ENSBTAG00000006246	0.247401334	0.122890746	0.124510589
Q3T0Y1	OTUB1	bta:540984; Q3T0Y1	ENSBTAG00000003967	-0.008638918	0.03124068	-0.039879599
F1N2W0	PTGR1	F1N2W0	ENSBTAG00000011027	0.153791928	0.170852822	-0.017060894
Q3T108	PSMB4	bta:506203; Q3T108	ENSBTAG00000021288	0	0.064266396	-0.064266396
A6H772	PPP4C	A6H772; bta:540398	ENSBTAG00000012928	-0.119323008	-0.12998149	0.010658481
A0AAA9TD90	COA7	A0AAA9TD90; bta:520518	ENSBTAG00000062331	0.190822342	0.175132968	0.015689374
F1MWR3	ETFA	F1MWR3	ENSBTAG00000012490	0.135842389	0.102211611	0.033630778
A0AAF6YJV2	GNAI1	A0AAF6YJV2	ENSBTAG00000002714	0.052179864	0.133692684	-0.08151282
E1BG63	TMTC3	bta:522132; E1BG63	ENSBTAG00000009023	0.02398668	0.092308871	-0.06832219
A0A452DIP2	GALE	A0A452DIP2	ENSBTAG00000005002	0.049486353	0.106128351	-0.056641997
A0AAA9RS28	DOCK9	A0AAA9RS28	ENSBTAG00000010395	-0.38144338	-0.222392421	-0.159050958
G3X6U1	ALDH1A2	bta:535075; G3X6U1	ENSBTAG00000010119	-0.17838658	-0.119153473	-0.059233107
A0AAF6Z178	RPS8	A0AAF6Z178	ENSBTAG00000015285	-0.073462162	-0.076541555	0.003079393
P13620	ATP5PD	bta:282710; P13620; Q3SX10	ENSBTAG00000021227	0.018897031	-0.043068722	0.061965753
F1N3Z8	PPP4R2	bta:504900; F1N3Z8	ENSBTAG00000002147	-0.178378509	-0.072350844	-0.106027665
F1MMK0	COMT	F1MMK0	ENSBTAG00000019516	0.387023123	0.430091845	-0.043068722
E1BPQ8	CDC73	bta:100139547; E1BPQ8	ENSBTAG00000016703	0.101948208	0.092071528	0.009876679
A0AAA9T466	CORO7	A0AAA9T466	ENSBTAG00000009201	-0.144816553	-0.017668648	-0.127147905
F6RNL0	NOP14	F6RNL0	ENSBTAG00000001504	-0.102615066	-0.180189492	0.077574425
E1BCS1	CNOT3	E1BCS1	ENSBTAG00000014900	0.08094752	0.10507579	-0.024128271
Q08E42	LRRC8A	bta:505605; Q08E42	ENSBTAG00000013030	-0.153030079	-0.251727342	0.098697263
A0AAF6DMG5	SCYL2	A0AAF6DMG5	ENSBTAG00000013859	0.227512493	0.320621897	-0.093109404
P61585	RHOA	bta:338049; P06749; P61585; Q3ZC72; Q9UEJ4	ENSBTAG00000004279	-0.143073782	-0.185119678	0.042045897
A0AAA9SA64	MESD	A0AAA9SA64	ENSBTAG00000001565	0.08323916	0.098019319	-0.014780158
Q08DY0	ERICH5	bta:504339; Q08DY0	ENSBTAG00000018493	0.476813697	0.157403081	0.319410616
A0AAA9TBT5	ACADM	A0AAA9TBT5	ENSBTAG00000024240	-0.141596278	-0.107439268	-0.034157011
Q3MHL3	RBBP4	A5D9B1; bta:767940; Q3MHL3		0.066045335	-0.052358599	0.118403934
Q29RQ2	KHDRBS1	bta:538775; Q29RQ2	ENSBTAG00000012497	-0.25420738	-0.240298054	-0.013909326
Q32KW2	UBXN1	bta:506676; Q32KW2	ENSBTAG00000010481	-0.196166425	-0.111277527	-0.084888898
P81623	ERP29	bta:613357; P81623; Q17QC3	ENSBTAG00000006665	0.280924565	0.250118518	0.030806047
Q32LF9	CAMLG	bta:615658; F1MX41; Q32LF9		0.117173109	0.063298868	0.053874241
A0A3Q1LR80	LARP4B	A0A3Q1LR80	ENSBTAG00000013899	-0.140230614	-0.316424708	0.176194094
A0AAF6YR53				0.504042505	0.473931188	0.030111317
A0AAA9THW9	PIK3C3	A0AAA9THW9	ENSBTAG00000026290	-0.087545795	-0.126134293	0.038588498
A5PKA3	CCDC80	A5PKA3; bta:515235		-0.10416634	-0.177396251	0.073229911
A6QPN5	MMP2	A6QPN5		-0.119949153	-0.079546799	-0.040402354
A0AAF6DMH7	RIPK1	A0AAF6DMH7	ENSBTAG00000006378	0.182698426	0.090324278	0.092374148
A0A3Q1MK17	MTA1	A0A3Q1MK17	ENSBTAG00000047606	-0.141820819	-0.146584813	0.004763995
A4IF89	EXOC8	A4IF89; bta:540237	ENSBTAG00000006111	-0.13628486	0.014824647	-0.151109507
Q5E9A4	RAE1	bta:513080; Q5E9A4	ENSBTAG00000010934	-0.015658413	-0.012798759	-0.002859654
A0A3Q1LU41	OSBPL8	A0A3Q1LU41	ENSBTAG00000004427	0.036525876	-0.035956878	0.072482754
O62830-2	PPM1B	O62830-2		-0.017158121	-0.071384633	0.054226512
A0AAA9U062	PEPD	A0AAA9U062	ENSBTAG00000031941	-0.370441556	-0.195502467	-0.17493909
A0A3Q1M4Q5	PCYT1A	A0A3Q1M4Q5	ENSBTAG00000016979	-0.214481805	-0.129050643	-0.085431162
Q0IIF2	EIF2B1	bta:516670; Q0IIF2		0.177473007	0.148438394	0.029034613
A0AAF6YZF4	RPS11	A0AAF6YZF4	ENSBTAG00000013924	-0.157183333	-0.245851394	0.088668062
A7YY75	SEH1L	A7YY75; bta:506509	ENSBTAG00000010792	-0.184141939	-0.150621863	-0.033520076
A0AAA9SQQ3	LAS1L	A0AAA9SQQ3	ENSBTAG00000002210	0.019752847	0.065792314	-0.046039466
A0A3Q1MQL4	TUT7	A0A3Q1MQL4	ENSBTAG00000001737	-0.203813409	-0.283506051	0.079692642
A0AAA9SZ86	SNX27	A0AAA9SZ86	ENSBTAG00000001942	0.224764389	0.251659616	-0.026895227
A0AAA9TD15	ENSA	A0AAA9TD15	ENSBTAG00000007123	0.319190532	0.43371892	-0.114528388
A0AAA9SWA2	GNAQ	A0AAA9SWA2; bta:536654	ENSBTAG00000009789	0.133932155	0.148981741	-0.015049587
F6R1L1	PPP4R3A	F6R1L1	ENSBTAG00000005040	0.007222518	0.009022512	-0.001799994
P52556	BLVRB	bta:281650; P52556; Q3T0T4		0.170105711	0.111091551	0.05901416
A0AAA9TRU0	DEK	A0AAA9TRU0	ENSBTAG00000012865	0.017774702	-0.037749277	0.055523978
Q3MHE9	JSP.1	bta:407173; Q3MHE9		-0.029810933	0.129341154	-0.159152087
A0AAA9TAT3	UBFD1	A0AAA9TAT3	ENSBTAG00000046033	0.234003271	0.219530149	0.014473122
F1MU05	PALS2	F1MU05	ENSBTAG00000015303	0.031967729	0.199857508	-0.167889779
A0AAA9TQV8	THUMPD3	A0AAA9TQV8	ENSBTAG00000003112	0.134573626	0.080501803	0.054071823
A3KMZ0	STXBP3	A3KMZ0; bta:512490		-0.017647866	0.102942275	-0.120590141
Q3SX44	DDAH2	bta:540386; Q3SX44	ENSBTAG00000013530	0.150140349	0.14313356	0.007006789
A0A3Q1LY00	PALS1	A0A3Q1LY00; bta:528109	ENSBTAG00000011767	0.10018393	0.253547415	-0.153363485
A5PKJ4	MAPK7	A5PKJ4; bta:537703		-0.006966189	0.024803391	-0.03176958
A0AAA9TUK9	DDOST	A0AAA9TUK9	ENSBTAG00000006085	-0.076621282	-0.111555072	0.03493379
Q2KIS4	DHRS1	bta:528832; Q2KIS4	ENSBTAG00000010234	-0.451593232	-0.338322533	-0.113270699
A0AAA9ST05	TBK1	A0AAA9ST05	ENSBTAG00000017401	-0.14303463	-0.128209982	-0.014824647
F1MQD1	CAPN5	F1MQD1	ENSBTAG00000005034	-0.247647297	0.058220947	-0.305868244
Q9BGI1	PRDX5	bta:282885; Q3SZ78; Q9BGI1	ENSBTAG00000008648	0.354108501	0.394554026	-0.040445526
E1BNQ2	DHX37	E1BNQ2	ENSBTAG00000020414	-0.201695781	-0.133346189	-0.068149592
A2VE47	ADPGK	A2VE47; bta:518158	ENSBTAG00000001578	0.084653874	0.017982834	0.06667104
E1BGG6	RFX5	E1BGG6	ENSBTAG00000017580	-0.100587584	0.125408199	-0.225995783
A0AAA9SH86	TAF15	A0AAA9SH86	ENSBTAG00000006916	-0.031283204	0.113321824	-0.144605028
Q3T025	RPL17	bta:514046; Q3T025	ENSBTAG00000022902; ENSBTAG00000060385	-0.05652518	-0.064130337	0.007605158
A0A3Q1MF00	PRIM1	A0A3Q1MF00; bta:506240	ENSBTAG00000044000	-0.284753087	-0.121875951	-0.162877137
A0AAF6YKI3	DNAJB11	A0AAF6YKI3	ENSBTAG00000003151	-0.004208156	-0.054232312	0.050024156
A0A3Q1MHB0	EGFR	A0A3Q1MHB0	ENSBTAG00000011628	-0.106695465	0.129691077	-0.236386542
A0AAA9T950	MAP2K4	A0AAA9T950	ENSBTAG00000012103	0.02187048	0.071648041	-0.049777561
A5PJI5	GET3	A5PJI5; bta:504586		0.102037699	0.08246216	0.019575538
Q5E9R9	ASCC2	bta:505503; Q5E9R9		-0.353413714	-0.254220357	-0.099193357
P02081				0.331993959	0.282302133	0.049691826
E1B9S7	OTUD7B	E1B9S7	ENSBTAG00000001586	-0.001305016	-0.225439297	0.224134281
A0AAA9SFU7	PPP1R18	A0AAA9SFU7	ENSBTAG00000039695	-0.064521506	-0.079596826	0.01507532
Q3T0C8	PDLIM2	bta:512907; Q3T0C8	ENSBTAG00000007369	0	0.273807931	-0.273807931
F1MYN1	PLEKHA5	F1MYN1	ENSBTAG00000017571	0.106999599	0.0804258	0.026573799
A0A3Q1LT51	TAB1	A0A3Q1LT51	ENSBTAG00000019828	0.04842669	0	0.04842669
A0A452DKB6	COG7	A0A452DKB6	ENSBTAG00000032223	0.091181007	0.020944014	0.070236992
A0A452DIL1	PCNP	A0A452DIL1	ENSBTAG00000015069	0.372672894	0.171836616	0.200836278
A0AAA9TPL7	PRPF31	A0AAA9TPL7	ENSBTAG00000007757	-0.088536675	-0.050983929	-0.037552746
O97902	ASAP1	bta:327705; O97902		0.212504425	0.134431543	0.078072882
F1MQ89	COG2	bta:512213; F1MQ89	ENSBTAG00000020314	-0.078322187	0.046702397	-0.125024584
E1BI41	GIT2	bta:516048; E1BI41	ENSBTAG00000006506	0.052883302	0.109304891	-0.056421589
A0AAA9S835	ERAP1	A0AAA9S835	ENSBTAG00000013557	-0.342887714	-0.195819326	-0.147068388
A0AAA9RYG1	DSG2	A0AAA9RYG1	ENSBTAG00000021923	0.097333085	0.019673419	0.077659666
E1BK49	RPRD2	E1BK49	ENSBTAG00000014984	0.062698905	0.066011639	-0.003312734
A0AAA9TTI7	MCU	A0AAA9TTI7	ENSBTAG00000003876	0.076923977	0.092103118	-0.015179141
A0A173FDL2	BoLA	A0A173FDL2		-0.322235345	-0.146007752	-0.176227593
E1BJQ7	GIPC1	bta:519617; E1BJQ7	ENSBTAG00000000764	0.001343917	-0.013508479	0.014852396
G3X6L5	ARL8A	G3X6L5	ENSBTAG00000006574	0	-0.008189398	0.008189398
A0AAF6ZNP5	THUMPD1	A0AAF6ZNP5	ENSBTAG00000047673	0.1908079	0.31275978	-0.12195188
E1BGA6	COG3	E1BGA6	ENSBTAG00000017907	-0.100327059	-0.042145988	-0.058181071
A0A3Q1LWP2	PPP1R12C	A0A3Q1LWP2	ENSBTAG00000017847	-0.004123952	0.051241285	-0.055365237
A0AAA9TUH8	WDR12	A0AAA9TUH8	ENSBTAG00000015424	0.164630702	0.266992419	-0.102361718
A0A3Q1MA84	WIZ	A0A3Q1MA84	ENSBTAG00000010450	-0.278216604	-0.314001179	0.035784575
A6H7C4	CBX3	A6H7C4; bta:539788	ENSBTAG00000049437	-0.04157084	-0.167630667	0.126059826
Q76I82	RPS15A	bta:337888; Q76I82	ENSBTAG00000020733	-0.128424955	-0.134759521	0.006334566
F1N194	ZNF622	F1N194	ENSBTAG00000016441	0.274270192	0.20722099	0.067049202
A0AAF6ZFR0	CSNK2B	A0AAF6ZFR0	ENSBTAG00000008837	0.189779744	0.166649869	0.023129875
A0A3Q1MQJ9				0.07364414	-0.151686331	0.225330471
F1N053	CTBP2	F1N053	ENSBTAG00000003397	-0.032644763	-0.187042372	0.154397609
F1MLB2	CHD8	F1MLB2	ENSBTAG00000020422	0.023170848	-0.176638826	0.199809674
A0AAA9S9H0	RAN	A0AAA9S9H0	ENSBTAG00000055033	-0.015520585	-0.1168813	0.101360715
E1BBG4	RIMOC1	E1BBG4	ENSBTAG00000004929	0.22527493	0.142740172	0.082534758
Q5E971	TMED10	A4FV04; bta:529761; Q5E971	ENSBTAG00000005694	0.099430094	0.069559564	0.02987053
A5D7G0	YARS2	A5D7G0; bta:536956	ENSBTAG00000011399	-0.001397283	-0.065718264	0.064320981
E1BG26	ATP13A3	A0A3Q1LMJ5; E1BG26	ENSBTAG00000013204	-0.100043397	-0.062451508	-0.037591888
Q2TBH2	RRAS	bta:616503; Q2TBH2	ENSBTAG00000020983	0.152449679	0.160464672	-0.008014993
E1BG07	CWF19L1	E1BG07	ENSBTAG00000007594	0.089674999	0.24197279	-0.152297792
Q3ZBR5	TTC1	bta:504602; Q3ZBR5	ENSBTAG00000010321	0.310961883	0.165276333	0.145685549
Q2TBV6	CCDC124	bta:510778; Q2TBV6	ENSBTAG00000004604	0.298015475	0.233641562	0.064373912
A0A3Q1MYR8	KRT3	A0A3Q1MYR8	ENSBTAG00000045662	-0.153978036	1.450613272	-1.604591307
Q17QJ7	PYCR2	bta:504987; Q17QJ7	ENSBTAG00000005835	0.032926005	0.079515564	-0.046589559
Q2KIV3	RBM4B	bta:505756; Q2KIV3	ENSBTAG00000000423	-0.136600509	-0.179798198	0.043197689
Q1JP69	GNL2	Q1JP69		-0.112366523	0.010637431	-0.123003954
Q5E9N0	BYSL	B0JYL5; bta:514128; Q5E9N0	ENSBTAG00000010101	-0.350230353	-0.437905872	0.087675518
Q3T0Q4	NME2	bta:615447; Q3T0Q4	ENSBTAG00000047186	0.103667596	0.032926005	0.070741591
E1BA82	UBAP2	bta:506577; E1BA82	ENSBTAG00000000695	-0.042125476	-0.19837554	0.156250065
A0AAA9SLR3	RELA	A0AAA9SLR3	ENSBTAG00000013895	-0.021061616	-0.042435266	0.021373651
A0A3Q1LKF0	CCDC91	A0A3Q1LKF0	ENSBTAG00000012448	0.08890565	0.053251381	0.03565427
A0AAA9S1I7	ECD	A0AAA9S1I7	ENSBTAG00000000315	0.088293701	-0.210925854	0.299219555
A0A3Q1N8Z4	HNRNPH3	A0A3Q1N8Z4	ENSBTAG00000014316	0.325770161	0.358873603	-0.033103442
G3MXD0	IRF2BP2	G3MXD0	ENSBTAG00000046841	-0.08973862	-0.20535613	0.11561751
F1MEP1	QNG1	F1MEP1	ENSBTAG00000006478	0.01880985	0.025025693	-0.006215843
A0JN47	PLXDC2	A0A3Q1LUJ4; A0JN47; bta:515731	ENSBTAG00000009475	-0.147445659	-0.15819204	0.010746381
F1MNV5	KNG1	F1MNV5	ENSBTAG00000005122	0.08607095	0.632776744	-0.546705794
F6QGZ0	LIMS1	F6QGZ0	ENSBTAG00000010971	-0.029775251	-0.055994247	0.026218996
F1MPT7	RRS1	F1MPT7	ENSBTAG00000016716	0.107334044	0.049007341	0.058326703
E1BIM8	SRC	E1BIM8	ENSBTAG00000008938	-0.006152227	-0.01480966	0.008657433
F6PSE2	NBN	F6PSE2	ENSBTAG00000013225	0.099324738	0.070991832	0.028332906
E1BA27	GLMN	E1BA27	ENSBTAG00000018737	-0.051504017	0.066391222	-0.117895239
P34943	NDUFA9	bta:404188; P34943; Q3T0K3	ENSBTAG00000005465	-0.116151279	0.047723041	-0.16387432
F1N3B9	TRIP13	bta:506746; F1N3B9	ENSBTAG00000006972	0.046134458	-0.003027693	0.049162151
A0A3Q1MG38	CAP2	A0A3Q1MG38	ENSBTAG00000016924	0.288356945	0.205818671	0.082538274
A0AAA9TD87	NDRG3	A0AAA9TD87	ENSBTAG00000019621	0.105545773	-0.047305715	0.152851488
Q0VC97	CBR3	bta:516036; Q0VC97	ENSBTAG00000018688	0.168744607	0.216529123	-0.047784517
G3N3X2	SRBD1	G3N3X2	ENSBTAG00000007508	-0.318228444	-0.225538977	-0.092689467
F1MYK6	SCAF8	F1MYK6	ENSBTAG00000031917	-0.140969043	-0.24870653	0.107737487
Q1LZF8	CLCC1	bta:519085; Q1LZF8	ENSBTAG00000017129	0.244591801	0.171035194	0.073556607

Accession	Gene_Symbol	Gene_ID	Ensembl_Gene ID	log2(ratio(OV24H/OV0H))	log2(ratio(OV24R/OV0H))	log2(ratio(OV24H/OV24R))
F1MT41	PTGFRN	bta:538209; F1MT41	ENSBTAG00000018022	-0.035536157	-0.08866998	0.053133823
E1BDX9	FAM50A	bta:515539; E1BDX9	ENSBTAG00000033032	0.022742462	-0.166883793	0.189626255
Q0VD29	RRAGC	bta:617367; E1BFI0; Q0VD29	ENSBTAG00000009368	0.010746381	0.123956992	-0.11321061
A5D7U9	DCP5	A5D7U9; bta:282640		-0.081654154	0.087373035	-0.169027189
A6H788	SNRPA1	A6H788; bta:512584	ENSBTAG00000013593	-0.149034404	-0.039878024	-0.109156381
Q5E9G3	PSME2	bta:509857; Q3T038; Q5E9G3; Q9TR87; Q9TR88; Q9TR89; Q9TR90	ENSBTAG00000005814	0.023773062	0.078711976	-0.054938914
A0AAA9T2P6	CTBP1	A0AAA9T2P6	ENSBTAG00000008480	-0.153788607	-0.170259617	0.01647101
A0A3Q1MR25	PDK3	A0A3Q1MR25	ENSBTAG00000017832	-0.237516911	-0.020201427	-0.217315483
Q0V898	NELFE	bta:540158; Q0V898		-0.239977787	-0.086134841	-0.153842946
Q29RH4	THOC3	bta:529231; Q29RH4	ENSBTAG00000035174	0.042673337	-0.078251446	0.120924782
A0AAA9ST33	EXOC6B	A0AAA9ST33	ENSBTAG00000020799	-0.15552878	-0.015237439	-0.140291341
Q3SZJ9	PMM2	bta:510978; Q3SZJ9	ENSBTAG00000001440	0.069650432	0.020027055	0.049623377
F1MNN1	HAUS3	F1MNN1	ENSBTAG00000024787	-0.101074548	-0.023153075	-0.077921473
A0A3Q1LL63	AASDHPPT	A0A3Q1LL63; bta:519816	ENSBTAG00000012279	-0.031093769	-0.154055292	0.122961522
A0AAA9SSM4	CEP55	A0AAA9SSM4	ENSBTAG00000005129	0.127358698	-0.239811449	0.367170147
E1BQ32	CRK	E1BQ32	ENSBTAG00000005665	0.029923637	0.066132169	-0.036208533
A0AAA9SMA6	SRPK1	A0AAA9SMA6	ENSBTAG00000018838	0.030597722	-0.121990524	0.152588247
A0AAA9T3P5	RSF1	A0AAA9T3P5	ENSBTAG00000020268	-0.130537191	-0.153489642	0.022952451
Q32LM0	SH3GLB1	bta:533918; Q32LM0		-0.058560079	-0.115254802	0.056694723
A0AAA9TZL3	NDUFV2	A0AAA9TZL3	ENSBTAG00000004871	0.047628863	0.070071238	-0.022442375
A0A3Q1LX83				0.102433234	0.081204741	0.021228493
A0AAA9SIH1	TAPBP	A0AAA9SIH1	ENSBTAG00000016828	-0.360973294	-0.245987207	-0.114986087
A6QLW1	PIK3R4	A6QLW1; bta:507414	ENSBTAG00000020787	-0.027170201	-0.024430039	-0.002740162
A0A3Q1MVQ2	PCID2	A0A3Q1MVQ2	ENSBTAG00000021725	0.084115335	-0.03562391	0.119739244
Q17Q89	PFDN6	bta:509914; Q17Q89	ENSBTAG00000010723	0.122109276	0.157995757	-0.03588648
F1N0C7	NTN1	F1N0C7	ENSBTAG00000012137	-0.230083984	-0.115609484	-0.1144745
A0A3Q1MP16	AQR	A0A3Q1MP16	ENSBTAG00000012712	-0.002923395	0.104200819	-0.107124214
E1BPG0	MCAT	E1BPG0	ENSBTAG00000002413	0.136736337	0.108320655	0.028415682
A0A3Q1MV30	LSG1	A0A3Q1MV30	ENSBTAG00000008461	0.028912692	-0.098180394	0.127093086
A6QLZ0	LGALS3	A6QLZ0; bta:786492	ENSBTAG00000002326	0.001841347	-0.012955655	0.014797002
A0AAF6Z9V5	IKBIP	A0AAF6Z9V5	ENSBTAG00000021660	-0.061776198	-0.120043853	0.058267655
E1BGS4	TXLNA	bta:511331; E1BGS4	ENSBTAG00000012867	0.049563101	-0.092549243	0.142112344
A0A3Q1MDA4	DAG1	A0A3Q1MDA4	ENSBTAG00000011580	-0.086955796	0.035267635	-0.122223431
F1MI35	HHIPL1	bta:100299504; F1MI35	ENSBTAG00000026913	-0.08808397	-0.071283838	-0.016800132
E1BH39	KATNA1	bta:506715; E1BH39	ENSBTAG00000032024	0.081079606	0.05877663	0.022302976
Q2HJD7	HIBADH	bta:512002; Q2HJD7	ENSBTAG00000011036	0.156281975	0.317795671	-0.161513687
A0A140T867	KRT17	A0A140T867; bta:281889	ENSBTAG00000006806	-0.045323991	2.320440013	-2.365764004
E1BC58	RAB2B	bta:528574; E1BC58	ENSBTAG00000044207	0.073011127	-0.008503139	0.081514266
Q24K01	NUDCD3	bta:533678; Q24K01	ENSBTAG00000006325	0.437963896	0.338341185	0.099622711
A0AAF6YHP1	TM9SF4	A0AAF6YHP1	ENSBTAG00000001015	-0.094563002	-0.051768062	-0.04279494
A0AAA9T7H2	GEMIN4	A0AAA9T7H2	ENSBTAG00000013393	0.123932328	0.304245817	-0.180313489
A0A654IE56	CXNK1	A0A654IE56; bta:281193		-0.113134038	-0.043807968	-0.069326071
A0A3Q1MP25	ACAD9	A0A3Q1MP25	ENSBTAG00000003242	-0.035422054	-0.012163309	-0.023258745
Q02399	CDK5	bta:281066; Q02399; Q0VCN5; Q6LBE2		-0.11783649	-0.073752461	-0.044084029
A0A452DHU5	DAP3	A0A452DHU5	ENSBTAG00000001199	-0.385891154	-0.29770843	-0.088182723
A7MB33	GTPBP1	A7MB33		-0.019743935	0.012551317	-0.032295252
Q3T0X6	RPS16	bta:506297; Q3T0X6	ENSBTAG00000021093	-0.109121722	-0.173854424	0.064732701
A7MAZ2	STX12	A7MAZ2; bta:505538; F1MIE7	ENSBTAG00000011192	-0.002642299	0.014446771	-0.01708907
A0AAA9SKK8	MAP3K4	A0AAA9SKK8	ENSBTAG00000020079	-0.058125908	0.054261594	-0.112387502
A0AAF7ABH7	ITPK1	A0AAF7ABH7	ENSBTAG00000009845	0.014597376	0.096925051	-0.082327675
P50291	FST	bta:327681; P50291; Q32XW5		-0.008082346	-0.119243345	0.111160999
O18973	RABGEF1	bta:282335; O18973		0.051337262	0.072418113	-0.021080851
Q28165	PABPN1	A3KN38; bta:282298; Q28165	ENSBTAG00000006884	-0.015325731	-0.020939338	0.005613606
F2Z4I2	AP3M1	F2Z4I2	ENSBTAG00000021883	0.178873958	0.121678557	0.057195402
Q148C9	HEBP1	bta:533227; Q148C9		-0.062766087	-0.001365542	-0.061400545
Q17QG3	RILPL1	bta:505840; Q17QG3		0.108566357	-0.017452169	0.126018526
A5PKI3	FAM3C	A5PKI3; bta:615690	ENSBTAG00000007976	0.095522611	0.049243519	0.046279092
F1MQY1	LIG3	F1MQY1	ENSBTAG00000018689	-0.298388788	-0.23356932	-0.064819468
G5E5S2	NELFB	G5E5S2	ENSBTAG00000021861	0.00557564	0.009743274	-0.004167634
Q2HJ18	VPS33B	A1L527; bta:526538; Q2HJ18		-0.063117017	0.329797671	-0.392914688
A0AAA9SD81	TM9SF2	A0AAA9SD81	ENSBTAG00000001589	0.033008762	-0.03378172	0.066790482
Q2KHZ8	GBA1	bta:537087; Q2KHZ8		-0.012539196	0.168691402	-0.181230597
A0A3Q1MZJ3	THOC6	A0A3Q1MZJ3	ENSBTAG00000001415	0.022764891	0.004295869	0.018469022
A2VE52	REXO2	A2VE52; bta:540139	ENSBTAG00000005843	0.046617789	0.055763272	-0.009145483
A0AAA9T8U5	CSAD	A0AAA9T8U5	ENSBTAG00000007829	0.043863814	0.066824963	-0.022961148
A0A3Q1MCY3	MYO10	A0A3Q1MCY3	ENSBTAG00000020126	-0.26712882	-0.220446777	-0.046682043
A0AAF6YUU7	NMRAL1	A0AAF6YUU7	ENSBTAG00000010015	0.001090061	0.095969059	-0.094878998
A0AAA9SPH3	ARMC10	A0AAA9SPH3	ENSBTAG00000014161	0.001319337	-0.157439747	0.158759084
P38409	GNA11	bta:281788; P38409; Q2TA47	ENSBTAG00000012181	0.031891722	0.137630976	-0.105739254
P48427	TBCA	bta:327683; P48427; Q32KV5	ENSBTAG00000003263	0.051530301	0.043068722	0.008461579
E1BDN8	SREK1	bta:507801; E1BDN8	ENSBTAG00000007321	0.274891534	0.154485684	0.12040585
A0AAA9TXU6	HELLS	A0AAA9TXU6	ENSBTAG00000005979	-0.278893483	-0.247347769	-0.031545714
F1N458	ARHGAP17	F1N458	ENSBTAG00000020043	0.068057442	0.15984023	-0.091782788
F1N020	KIF3B	bta:508404; F1N020	ENSBTAG00000003215	-0.037288429	-0.065160374	0.027871945
F1N2H6	CD276	bta:508656; F1N2H6	ENSBTAG00000019734	0	0.018487227	-0.018487227
F1MND3	TEX10	F1MND3	ENSBTAG00000044003	-0.058416686	0.01282404	-0.071240727
Q3ZBU9	UBXN4	bta:536181; Q3ZBU9	ENSBTAG00000015169	-0.33288995	-0.207104378	-0.125785573
F1N1X8	UBE2Z	F1N1X8	ENSBTAG00000018802	-0.174774401	-0.196800707	0.022026306
F1MBS4	SLC25A10	F1MBS4	ENSBTAG00000055145	-0.33913228	-0.413336809	0.07420453
A0A3Q1N3E8	UFD1	A0A3Q1N3E8	ENSBTAG00000004284	-0.018147347	-0.029429477	0.01128213
P00125	CYC1	bta:512500; P00125; Q2TBM2	ENSBTAG00000012232	0.018437248	0.317902111	-0.299464862
A0A3Q1NNG9	FCHO2	A0A3Q1NNG9	ENSBTAG00000017545	-0.042821283	0.1559447	-0.198765983
A0AAA9U0A2	CCPG1	A0AAA9U0A2; bta:538508	ENSBTAG00000012741	-0.099410922	0.039622772	-0.139033694
G5E5M5	PGRMC1	G5E5M5	ENSBTAG00000019552	0.528233396	0.24076813	0.287465266
A0AAF6Z0W3	LANCL1	A0AAF6Z0W3	ENSBTAG00000015066	0.257971845	0.330837576	-0.072865731
A0A3Q1LNZ3	NUCKS1	A0A3Q1LNZ3	ENSBTAG00000008001	-0.214606676	0.022573692	-0.237180368
A6QLW0	BUB1	A6QLW0; bta:514777		0.176549392	0.006813212	0.169736179
A0AAA9TJD5	STK39	A0AAA9TJD5	ENSBTAG00000017162	0.135283566	0.02419671	0.111086855
A0A3Q1MIT1	YTHDF3	A0A3Q1MIT1	ENSBTAG00000018557	0.039933627	-0.064403033	0.10433666
A0A452DIS6	ERP27	A0A452DIS6	ENSBTAG00000006036	-0.203112077	-0.20812318	0.005011103
A0A3Q1MFQ7	EWSR1	A0A3Q1MFQ7	ENSBTAG00000017755	-0.195871948	-0.12035194	-0.075520008
A4IFE3	ACTR1B	A4IFE3; bta:100125305	ENSBTAG00000048041	0.002823279	0.045892001	-0.043068722
G5E547	SYAP1	G5E547	ENSBTAG00000003316	-0.017399548	-0.103040114	0.085640566
Q9MZ13	VDAC3	bta:282716; Q9MZ13		0.040077439	0.14640434	-0.106326901
F6RA54	OGFR	F6RA54	ENSBTAG00000031718	-0.356647457	-0.216947492	-0.139699964
A0AAA9SQN4	ARIH1	A0AAA9SQN4	ENSBTAG00000006708	0.155143161	0.164030427	-0.008887267
Q3ZC42	ADH5	bta:505515; Q3ZC42	ENSBTAG00000016007	-0.095185524	-0.011246705	-0.083938818
A0AAA9RUF4	PPIE	A0AAA9RUF4	ENSBTAG00000047314	0	0.035059291	-0.035059291
G5E518	CDK12	G5E518	ENSBTAG00000013238	-0.102218649	-0.178812805	0.076594156
A0AAA9SBQ7	ATM	A0AAA9SBQ7	ENSBTAG00000003111	-0.179303703	-0.129368004	-0.049935699
Q0V8C2	EXOC3	bta:513138; Q0V8C2	ENSBTAG00000008606	-0.076442504	-0.079407963	0.002965458
A0A3Q1MNZ0	ATPAF1	A0A3Q1MNZ0; bta:505430	ENSBTAG00000000094	-0.145638456	-0.228019408	0.082380953
A0A3Q1N5G3	MSRA	A0A3Q1N5G3	ENSBTAG00000021632	0.011693641	0.107439268	-0.095745627
A0A3Q1M3H2	LTBP2	A0A3Q1M3H2	ENSBTAG00000021957	-0.100556328	0.077852145	-0.178408473
F6QJJ8	PGRMC2	F6QJJ8	ENSBTAG00000010843	0.104108403	0.118386204	-0.0142778
A0A3Q1MJT2	A1BG	A0A3Q1MJT2	ENSBTAG00000009735	0.022672468	0.49324045	-0.470567981
E1B7G6	ANKRD50	E1B7G6	ENSBTAG00000001117	-0.04608923	-0.38294387	0.336854639
E1BNY8	GTF3C1	E1BNY8	ENSBTAG00000006126	-0.069262662	-0.007978057	-0.061284605
F1MZV2	CHMP5	F1MZV2	ENSBTAG00000012383	0.209328253	0.152157384	0.057170869
P22292	SLC25A11	A5PJY2; bta:282523; P22292; Q5E9W4	ENSBTAG00000004910	-0.139289867	-0.053418207	-0.08587166
F1MQ09	VEZT	F1MQ09	ENSBTAG00000014044	-0.01772177	-0.012866931	-0.004854839
Q58CU6				0.143005625	0.094135475	0.04887015
E1BBS6	QSOX2	E1BBS6	ENSBTAG00000011129	-0.016873819	0.313853288	-0.330727107
F1MMA2	TTL	bta:514559; F1MMA2	ENSBTAG00000014159	0.148136313	0.13272126	0.015415052
F1MDA6	VPS45	F1MDA6	ENSBTAG00000020635	-0.053912282	-0.1054163	0.051504017
E1BI11	MIDEAS	bta:505848; E1BI11	ENSBTAG00000009272	-0.363581716	-0.334726853	-0.028854863
Q3SWX4	NIPSNAP2	bta:767938; E1BL91; Q3SWX4	ENSBTAG00000031598	-0.156489488	-0.002882509	-0.153606979
A0AAA9RZZ1	GALM	A0AAA9RZZ1	ENSBTAG00000021102	0.188239899	0.147976029	0.04026387
Q3SZR3	ORM1	bta:497200; Q3SZR3		0.006765288	0.676375614	-0.669610326
F1MI29	XPO6	bta:510797; F1MI29	ENSBTAG00000010309	-0.251723125	-0.340434845	0.08871172
Q1RMW9	GOLPH3	bta:531359; Q1RMW9	ENSBTAG00000032848	-0.119321624	-0.353198245	0.233876622
E1BGC1	MCCC1	E1BGC1	ENSBTAG00000006548	-0.040197089	0.012686213	-0.052883302
A0AAA9RUI1	PPP6R1	A0AAA9RUI1	ENSBTAG00000021157	0.03562391	0.071762669	-0.03613876
A0A3Q1LIJ0	YBX3	A0A3Q1LIJ0	ENSBTAG00000009663	-0.204130215	-0.181622816	-0.022507399
Q08DA0	RABL6	bta:508218; Q08DA0		-0.249978253	-0.236009069	-0.013969184
A1A4P5	PFDN2	A1A4P5; bta:404138		0.190891278	0.164722345	0.026168933
A0AAF6YJR2	FKBP3	A0AAF6YJR2	ENSBTAG00000002610	0.027318119	0.101487822	-0.074169703
F6QMQ4	RPS14	F6QMQ4	ENSBTAG00000008570	-0.180664287	-0.154305879	-0.026358407
A7MAZ9	DCP1A	A7MAZ9; bta:783258		-0.072828373	-0.078698961	0.005870588
A0AAA9SAW2	LPCAT4	A0AAA9SAW2	ENSBTAG00000020040	0.018242962	-0.098115752	0.116358714
A0A3Q1MTL1	SCAF4	A0A3Q1MTL1	ENSBTAG00000018855	0.149602317	-0.017236705	0.166839022
A0A452DIZ8	STRIP1	A0A452DIZ8	ENSBTAG00000020964	-0.014873276	0.127379306	-0.142252583
G5E6N7	CBX1	G5E6N7	ENSBTAG00000002949	0.395259833	0.193689525	0.201570308
E1BEV7	BMP1	E1BEV7	ENSBTAG00000000917	-0.372564208	-0.418414859	0.045850652
A0A452DI40	IVD	A0A452DI40	ENSBTAG00000004409	-0.024058841	0.004763995	-0.028822835
A0AAA9TAZ9	PTPN2	A0AAA9TAZ9	ENSBTAG00000010563	0.060120992	0.012502972	0.04761802
A0A3Q1M0U3	SORBS3	A0A3Q1M0U3	ENSBTAG00000014401	-0.187416283	-0.062005391	-0.125410892
A0AAF6DM45	ABCF3	A0AAF6DM45	ENSBTAG00000006920	-0.375542605	-0.317291685	-0.05825092
Q5E9C0	RSU1	bta:617534; Q5E9C0		0.129482518	-0.00699999	0.136482508
A0AAA9TQT8	ATE1	A0AAA9TQT8	ENSBTAG00000003178	0.301992474	0.360043467	-0.058050993
F1MEG3	LAMA1	F1MEG3	ENSBTAG00000018160	0.42202393	-0.046584335	0.468608265
F1N455	CTSC	F1N455	ENSBTAG00000011100	-0.01603821	-0.004356405	-0.011681805
Q08DL0	SLC3A2	bta:507107; Q08DL0	ENSBTAG00000013240	0.513441203	0.683953263	-0.170512059
E1B725	GLA	bta:532742; E1B725	ENSBTAG00000019256	-0.084837945	0.078533403	-0.163371348
Q17QZ7	TTC27	bta:524908; Q17QZ7		-0.350156386	-0.22694495	-0.123211436
Q5E9N5	CLPB	bta:520639; Q5E9N5		0.287746159	0.216852304	0.070893855
A0AAA9TTF1	NOL10	A0AAA9TTF1	ENSBTAG00000002716	0.018324658	-0.063807969	0.082132627
F1MLG1	SRPRB	F1MLG1	ENSBTAG00000044075	0.136479661	0.053673183	0.082806479
Q3MHI4	PHAX	bta:507478; Q3MHI4	ENSBTAG00000009647	0.276300012	0.269259026	0.007040985
A0AAA9SCC4	SEPTIN10	A0AAA9SCC4	ENSBTAG00000022461	-0.326206976	-0.236225609	-0.089981367
A0AAA9T4C9	SLC44A1	A0AAA9T4C9	ENSBTAG00000007163	0.136107592	0.318239908	-0.182132316
A0AAA9TAH2	PUM3	A0AAA9TAH2	ENSBTAG00000013357	-0.144924736	-0.161062433	0.016137697
A0A3Q1M182	VAV2	A0A3Q1M182	ENSBTAG00000004511	-0.086761312	0.008395921	-0.095157233
A0A3Q1MQ45	AKT2	A0A3Q1MQ45	ENSBTAG00000001400	-0.112622995	-0.246128695	0.1335057
A6QQX0	PBK	A6QQX0; bta:534781	ENSBTAG00000021069	-0.152748865	0.012028097	-0.164776962
A0A3Q1MGZ4	TKFC	A0A3Q1MGZ4	ENSBTAG00000018201	-0.072734504	-0.034610959	-0.038123545
A5PJ81	LACTB	A5PJ81; bta:614196	ENSBTAG00000017373	-0.095394115	-0.13571801	0.040323895
A0AAA9TAG5	SELENBP1	A0AAA9TAG5	ENSBTAG00000008091	0.219636151	0.280718325	-0.061082174
F6RN75	SYNPO2	F6RN75	ENSBTAG00000006434	0.186962932	0.136953507	0.050009425
Q2HJD0	FAF2	bta:767948; Q2HJD0	ENSBTAG00000017744	0.030088578	-0.051060489	0.081149067
E1BB26	HEATR5B	bta:540503; E1BB26	ENSBTAG00000014310	0.005724988	0.076794351	-0.071069363
A0AAA9T3F8	TACC3	A0AAA9T3F8	ENSBTAG00000011044	-0.183518559	-0.170637268	-0.012881291
A0AAA9SP28	PHF2	A0AAA9SP28	ENSBTAG00000012922	0.125649794	0.113829741	0.011820053
E1BDD9	MRTFA	bta:509011; E1BDD9	ENSBTAG00000002630	0.072350844	0.11712507	-0.044774226
G1K192	ARL3	G1K192	ENSBTAG00000004318	0.120475884	0.114894852	0.005581032
A0AAA9SCV4	UAP1L1	A0AAA9SCV4	ENSBTAG00000048054	-0.911953862	-0.521894018	-0.390059844
F1MFX7	UHRF2	F1MFX7	ENSBTAG00000020815	-0.124010175	-0.125835216	0.001825041
F1MNM6	TANGO6	bta:534015; F1MNM6	ENSBTAG00000013941	0.24688036	0.086112298	0.160768062
A0AAA9U1B4	SRSF10	A0AAA9U1B4	ENSBTAG00000008072	0.08246216	-0.029113668	0.111575828
A0A3Q1M100	ITSN2	A0A3Q1M100	ENSBTAG00000023172	-0.102778597	0.128716031	-0.231494628
G3MX91	TARDBP	bta:540632; G3MX91	ENSBTAG00000003697	0.051024003	-0.017417053	0.068441056
A0A3Q1N6R4	CCDC22	A0A3Q1N6R4	ENSBTAG00000013277	0.229040797	0.226195244	0.002845553
A0AAA9TQ21	ESF1	A0AAA9TQ21	ENSBTAG00000007130	-0.200575839	-0.309363457	0.108787618
F6QY62	INTS4	F6QY62	ENSBTAG00000010505	-0.299778327	-0.051942632	-0.247835695
A0AAF7ABD1	ERLIN2	A0AAF7ABD1	ENSBTAG00000017831	0.015485247	0.193941883	-0.178456635
A0A452DI32	BCCIP	A0A452DI32	ENSBTAG00000005792	-0.039933627	-0.030963473	-0.008970154
A0AAA9S584	CPSF3	A0AAA9S584	ENSBTAG00000019735	0	-0.04580369	0.04580369
Q2KIR1	SNRPA	bta:509802; Q2KIR1	ENSBTAG00000009077	-0.056292985	0.051377359	-0.107670344
A0AAA9TI02	CERT1	A0AAA9TI02	ENSBTAG00000000081	0.086724701	0.160134498	-0.073409797
E1BLF7	NUDCD1	E1BLF7	ENSBTAG00000007386	0.134357985	0.071181565	0.063176419
A0AAF6ZJM9	QDPR	A0AAF6ZJM9	ENSBTAG00000040333	0.213751513	0.237554538	-0.023803025
Q3T133	TMED9	bta:618580; Q3T133; Q5E957	ENSBTAG00000001394	0.205368586	0.219375444	-0.014006858
A0A3Q1NKB1	RANBP1	A0A3Q1NKB1	ENSBTAG00000006656	0.146254459	0.141187922	0.005066537
A0A3Q1MRI8	IDH3G	A0A3Q1MRI8	ENSBTAG00000001059	-0.113849077	-0.184661545	0.070812468
A4FUC6	SRSF9	A4FUC6; bta:509905; F1MWF4	ENSBTAG00000012792	0.024368007	0.152147645	-0.127779638
A4FV64	SUGP1	A4FV64; bta:533971; E1BMW4	ENSBTAG00000006116	-0.10809378	0.059995965	-0.168089745
F1N081	FNTA	bta:281169; F1N081	ENSBTAG00000003201	-0.119271892	-0.189065188	0.069793296
A0A3Q1MXQ4	NHSL3	A0A3Q1MXQ4	ENSBTAG00000018062	-0.051555188	-0.017904798	-0.033650389
A5D9H9	POLDIP2	A5D9H9; bta:539673; F1MFH2	ENSBTAG00000008116	-0.538722322	-0.551694619	0.012972297

Accession	Gene_Symbol	Gene_ID	Ensembl_Gene ID	log2(ratio(OV24H/OV0H))	log2(ratio(OV24R/OV0H))	log2(ratio(OV24H/OV24R))
A8E644	DCLK1	A8E644; bta:613449; F1ME50		-0.028782901	0.233039666	-0.261822566
A0AAF6YXA0	MAT2B	A0AAF6YXA0	ENSBTAG00000012350	-0.117641611	-0.003056558	-0.114585052
Q0VCQ6	PDCD10	bta:506411; Q0VCQ6	ENSBTAG00000031709	-0.206450877	-0.166277224	-0.040173653
A6H704	CHMP7	A6H704; bta:541288	ENSBTAG00000013528	0.12041675	0.034801683	0.085614868
F1MIZ7	RO60	F1MIZ7	ENSBTAG00000013812	0.012798759	0.16196748	-0.149168721
Q3SZG7	RPL13	Q3SZG7		-0.020865788	-0.069723622	0.048857834
Q32KN6	PGK2	bta:538592; Q32KN6		0.179567583	-0.238991754	0.418559337
P30274	CCNA2	bta:281667; P30274; Q17QS7		-0.123842076	-0.261034036	0.13719196
E1BAT8	KIF20B	E1BAT8	ENSBTAG00000005708	-0.087270392	0.001538875	-0.088809267
A0AAA9SBU8	GTSE1	A0AAA9SBU8	ENSBTAG00000007102	-0.281742933	-0.451233453	0.16949052
A0A3Q1LQN8	GPKOW	A0A3Q1LQN8	ENSBTAG00000002568	-0.313951374	-0.376767482	0.062816108
A0AAA9U021	HACD3	A0AAA9U021	ENSBTAG00000015155	0.267229366	0.150519602	0.116709763
A0AAA9SEF0	NBEAL1	A0AAA9SEF0	ENSBTAG00000014469	-0.015325731	0.057063174	-0.072388906
A0AAA9SWJ0	ARAP1	A0AAA9SWJ0	ENSBTAG00000012051	-0.295016941	-0.131359517	-0.163657424
A0AAF6YNA5	ZWILCH	A0AAF6YNA5	ENSBTAG00000005213	-0.425454115	-0.364053571	-0.061400545
A0A3Q1LTK2	VPS41	A0A3Q1LTK2	ENSBTAG00000007305	0.042536173	0.046586794	-0.00405062
Q0P565	HDDC2	bta:509282; Q0P565		0.019257617	0.070927747	-0.051670131
A0AAA9SHG0	FAM120C	A0AAA9SHG0	ENSBTAG00000003855	0.07113809	0.107425766	-0.036287676
A0A3Q1M5Q9	PTPRF	A0A3Q1M5Q9	ENSBTAG00000000253	-0.191060569	0.169925001	-0.360985571
A0A3Q1LWF1	MARK3	A0A3Q1LWF1	ENSBTAG00000013410	-0.181018831	-0.227312484	0.046293652
A0A3Q1MDQ6	KLHDC4	A0A3Q1MDQ6	ENSBTAG00000006905	-0.179930579	-0.172161605	-0.007768974
A0A3Q1N4D8	POLD2	A0A3Q1N4D8	ENSBTAG00000012241	-0.20511443	-0.191817607	-0.013296823
F1MBJ4	PAXBP1	bta:514918; F1MBJ4	ENSBTAG00000003064	0.077358883	0.136947679	-0.059588796
E1BMM8	CHPF	E1BMM8	ENSBTAG00000020539	-0.249272505	-0.162454347	-0.086818157
Q08DP5	KAT7	bta:508246; F1MQP0; Q08DP5	ENSBTAG00000018699	0.13197065	0.125024584	0.006946066
Q0VC82	SEPHS1	bta:507470; Q0VC82		0.128104826	0.181005162	-0.052900337
Q3SZZ0	BRIX1	bta:518501; Q3SZZ0	ENSBTAG00000019211	-0.008267616	-0.030549063	0.022281447
A0AAF6YL13	CSTF2	A0AAF6YL13	ENSBTAG00000003547	0.242926832	0.128855066	0.114071767
A0A3Q1LTT9	MAPK14	A0A3Q1LTT9	ENSBTAG00000000783	-0.333956192	-0.216415771	-0.117540421
A0A3Q1MAC0	FNBP1L	A0A3Q1MAC0	ENSBTAG00000004383	0.077167861	0.176549392	-0.099381531
Q2TA25	PLK1	bta:538238; F1N225; Q2TA25	ENSBTAG00000014453	-0.141133708	0.12301089	-0.264144598
A0AAF6YQG5	EPB41	A0AAF6YQG5	ENSBTAG00000006667	0.01243115	0.119739244	-0.107308095
E1B8R6	ARIH2	E1B8R6	ENSBTAG00000005488	0.038832646	0.08094752	-0.042114874
E1BFI8	SPAG1	E1BFI8	ENSBTAG00000050156	0.084175139	0.471931774	-0.387756635
P05689	CTSZ	bta:404187; P05689; Q0IIK1	ENSBTAG00000018784	-0.401781403	-0.307206589	-0.094574813
F6QN89	SNTB2	F6QN89	ENSBTAG00000001654	0.047828525	0.124179411	-0.076350886
A6QLG5	RPS9	A6QLG5; bta:533892	ENSBTAG00000006487	-0.108718242	-0.166312456	0.057594215
A4IFP7	ARF5	A4IFP7; bta:511918		0.084277837	-0.013902557	0.098180394
E1B731	TMEM165	bta:532600; E1B731	ENSBTAG00000001269	0.18136981	0.054887238	0.126482573
Q3SZK4	TBRG4	bta:767855; Q3SZK4		-0.266574095	0.101075901	-0.367649997
F6RSR1	KLHL3	F6RSR1	ENSBTAG00000002796	-0.184424571	-0.083141235	-0.101283336
A0AAA9SZ85	ADPRHL1	A0AAA9SZ85	ENSBTAG00000020928	-0.041998349	-0.182727633	0.140729285
Q27U16	TRIM5	bta:505265; Q27U16		-0.308219469	-0.062554381	-0.245665088
A6QPQ5	MRPL1	A6QPQ5; bta:504835	ENSBTAG00000018360	-0.123204579	-0.03310069	-0.09010389
A0AAA9SLW2	VPS53	A0AAA9SLW2	ENSBTAG00000018126	0.089603461	0.122141941	-0.03253848
A0A3Q1M8V6	DAZAP1	A0A3Q1M8V6	ENSBTAG00000019714	0.118331666	0.110095516	0.00823615
A0AAA9RZX1	PIGS	A0AAA9RZX1	ENSBTAG00000013098	0.048461489	0.10510056	-0.056639071
A0AAA9SE16	STON1	A0AAA9SE16	ENSBTAG00000014168	-0.310021328	0.155301463	-0.465322792
A0AAF6ZIV2	GFM2	A0AAF6ZIV2	ENSBTAG00000015519	-0.111645356	-0.101729887	-0.009915469
F1MCX5	MAPKAPK2	F1MCX5	ENSBTAG00000038107	-0.291810782	-0.210141529	-0.081669253
E1BQ16	OGA	bta:538561; E1BQ16	ENSBTAG00000016336	0.354855114	0.645767525	-0.29091241
Q2KIY3	WDFY1	bta:614729; Q2KIY3	ENSBTAG00000016951	0.083523356	0.183059029	-0.099535674
A0AAF6Z3X9	PTGIS	A0AAF6Z3X9	ENSBTAG00000017537	0.068110188	0.101924901	-0.033814713
Q5BIS9	PRKAB1	A6QQW7; bta:534107; Q5BIS9	ENSBTAG00000005940	-0.03656148	-0.016759473	-0.019802008
Q3T057	RPL23	A6QLM2; bta:504876; Q3T057	ENSBTAG00000003229	-0.175264176	-0.235517341	0.060253165
Q3SZD7	CBR1	bta:515946; Q3SZD7	ENSBTAG00000023384	0.15480915	0.092710374	0.062098776
A6H7F3	BCL2L14	A6H7F3		0.005198907	0.050159048	-0.044960141
A0AAA9TIA3	CFH	A0AAA9TIA3	ENSBTAG00000023177	-0.031813749	0.298773429	-0.330587177
E1BEI2	SPCS2	E1BEI2	ENSBTAG00000006644	-0.115070653	-0.099303337	-0.015767316
Q1RMI2	RHOG	bta:538559; Q1RMI2	ENSBTAG00000000347	0.03876957	0.011183738	0.027585832
A6QLU9	LOC508963	A6QLU9; bta:508963		0.104301231	0.056759299	0.047541932
A0AAF6ZBL8	ZNF512	A0AAF6ZBL8	ENSBTAG00000003902	-0.228564652	-0.190956728	-0.037607924
E1BIE7	PGAM5	bta:508608; E1BIE7	ENSBTAG00000007513	-0.109278341	-0.084268917	-0.025009424
A0AAA9SDC5	LSM14A	A0AAA9SDC5	ENSBTAG00000000630	0.13492958	0.010959212	0.123970368
Q2KIC7	PPP6C	bta:511058; F1N6Z5; Q2KIC7	ENSBTAG00000008033	-0.005713651	-0.082126767	0.076413116
A0AAA9T3G0	RPL19	A0AAA9T3G0	ENSBTAG00000002060	-0.11883623	-0.213695416	0.094859186
E1BM72	PES1	E1BM72	ENSBTAG00000007728	-0.41861739	-0.19341145	-0.22520594
E1B8G1	NUMB	E1B8G1	ENSBTAG00000011683	0.004418671	-0.110323415	0.114742086
F1MW73	CRACD	F1MW73	ENSBTAG00000040398	0.397573594	0.270089163	0.12748443
E1BN70	INTS3	bta:511052; E1BN70	ENSBTAG00000006235	-0.291320758	-0.168565892	-0.122754866
A0AAF7AEM3	SGTA	A0AAF7AEM3	ENSBTAG00000015090	0.244678931	0.060372618	0.184306313
A0A3Q1MU57	DDX54	A0A3Q1MU57	ENSBTAG00000011930	-0.336266798	-0.245358301	-0.090908496
A0A3Q1MPG8	GBE1	A0A3Q1MPG8	ENSBTAG00000036262	-0.379971102	-0.153787102	-0.226184
Q58DG1	MYG1	Q58DG1		-0.056163896	-0.107520658	0.051356761
A0AAF6ZVE8	EEF1B2	A0AAF6ZVE8	ENSBTAG00000021979	0.031676706	-0.12580819	0.157483896
Q3B7M7	URI1	A7E3X6; bta:515119; Q3B7M7		0.193603998	0.196285588	-0.00268159
Q7YRD0	PNO1	A6QLI9; bta:360193; Q7YRD0	ENSBTAG00000010604	-0.468469203	-0.178811495	-0.289657708
H7BWW2				0.02448967	0.130486317	-0.105996647
A0A452DIE1	BTF3L4	A0A452DIE1	ENSBTAG00000011025	0.082544957	0.025116677	0.05742828
Q2HJ57	COTL1	bta:617165; Q2HJ57	ENSBTAG00000016315	0.095431004	0.06568366	0.029747343
Q24K16	PTGR3	bta:532505; Q24K16	ENSBTAG00000001724	-0.074141463	-0.069920996	-0.004220466
A0AAA9SNR2	LOC515358	A0AAA9SNR2	ENSBTAG00000062846	0.065986356	0.152829616	-0.08684326
P12624	MARCKS	bta:613548; P12624; Q1LZI0	ENSBTAG00000002082	-0.081031422	0.012690722	-0.093722144
Q08E32	CHMP4B	bta:616164; F1MAW4; Q08E32	ENSBTAG00000013387	0.234312902	0.188258534	0.046054368
Q2KID9	MRPS5	bta:512469; P82674; Q2KID9		-0.302381822	-0.253025982	-0.049355841
A0AAA9SRD9	TIA1	A0AAA9SRD9	ENSBTAG00000019182	0.262526504	0.031642622	0.230883882
E1BAJ4	STBD1	bta:513376; E1BAJ4	ENSBTAG00000010389	0.022697735	0.015642978	0.007054758
A0A3Q1MYN1	CNOT10	A0A3Q1MYN1	ENSBTAG00000004834	0.149989963	0.102597909	0.047392055
Q3MHP2	RAB11B	bta:532723; Q3MHP2	ENSBTAG00000002475	-0.008478154	-0.218640286	0.210162133
A5PJS3	DBR1	A5PJS3	ENSBTAG00000019949	-0.067673977	-0.020715977	-0.046958
A0AAF6YGY8	GRWD1	A0AAF6YGY8	ENSBTAG00000000080	0.108836492	0.059754743	0.04908175
A0AAA9TJF9	PDPK1	A0AAA9TJF9	ENSBTAG00000034436	0.070741591	-0.011886329	0.082627921
Q1LZB5	TOMM40	bta:510219; Q1LZB5	ENSBTAG00000001781	0.043284098	-0.014721506	0.058005603
Q3T094	ETHE1	bta:509150; Q3T094	ENSBTAG00000004379	-0.002947284	0.150824902	-0.153772186
A0AAF6YUZ1	NUDT21	A0AAF6YUZ1	ENSBTAG00000010207	-0.038913221	-0.172326569	0.133413348
Q08DB4	CPNE1	bta:615677; Q08DB4		0.136881806	0.202525423	-0.065643616
A3KMX6	UBR7	A3KMX6; bta:505130	ENSBTAG00000004310	0.09168873	-0.003791579	0.095480309
F1MI95	ERMP1	F1MI95	ENSBTAG00000015607	0.062240579	0.106139353	-0.043898773
A0AAF6YNF5	CSNK1A1	A0AAF6YNF5	ENSBTAG00000005326	-0.269151064	-0.355256271	0.086105207
A0AAA9RYF3	CENPC	A0AAA9RYF3	ENSBTAG00000001323	0.02363835	0.083344596	-0.059706246
P01131	LDLR	bta:281276; F1MZ58; P01131	ENSBTAG00000012314	-0.477853607	-0.512250615	0.034397008
A0A3Q1MCW7	CRYBG1	A0A3Q1MCW7; bta:526200	ENSBTAG00000017527	0.033947332	0.010268335	0.023678996
F1MM83	PGLS	F1MM83	ENSBTAG00000016783	0.40587244	-0.110586497	0.516458937
G3MZH3	MYO5A	G3MZH3	ENSBTAG00000006489	0.090686742	0.296531318	-0.205844576
Q2NL21	DNAJC11	bta:508137; Q2NL21	ENSBTAG00000011217	0.113321824	0.037814736	0.075507088
A0AAF6YPQ0	FECH	A0AAF6YPQ0	ENSBTAG00000006393	-0.234238159	-0.176374252	-0.057863906
A0AAF6YKI7	RMDN3	A0AAF6YKI7	ENSBTAG00000002921	-0.098293495	0.02389062	-0.122184115
A5PKG4	NFS1	A5PKG4; bta:517656; F1MZY8		-0.004204068	-0.015475181	0.011271112
A0AAA9TXM1	ALDH5A1	A0AAA9TXM1; bta:532724	ENSBTAG00000021902	0.050545442	0.060669659	-0.010124217
A0AAA9T8I6	RDH11	A0AAA9T8I6	ENSBTAG00000001950	0.271166721	0.695331105	-0.424164385
A0A3Q1LP37	UBE2S	A0A3Q1LP37	ENSBTAG00000009211	-0.006813212	-0.064284826	0.057471614
A0AAA9RWG1	ELMO2	A0AAA9RWG1	ENSBTAG00000003639	-0.061339824	-0.049728534	-0.01161129
F1MIM5	VWA8	F1MIM5	ENSBTAG00000001133	-0.119435231	-0.038723432	-0.080711799
F1N0D3	AXL	F1N0D3	ENSBTAG00000003166	-0.054447784	0.007613184	-0.062060968
Q3T0I4	ALYREF	bta:537706; Q3T0I4	ENSBTAG00000008498	-0.007494537	-0.081495118	0.074000581
G3N0V2	KRT1	G3N0V2	ENSBTAG00000046089	-0.220356154	0.786748233	-1.007104387
A0AAF7AEY9	AK1	A0AAF7AEY9	ENSBTAG00000006305	0.052118226	0.158552259	-0.106434033
F1MM32	QSOX1	F1MM32	ENSBTAG00000014191	0.17994081	0.169072999	0.010867811
A0A3Q1NBY0	NTAN1	A0A3Q1NBY0; bta:505869	ENSBTAG00000004803	0.026967048	0.132676489	-0.105709441
A8E4P3	STOM	A8E4P3; bta:100125834; F1N2R1	ENSBTAG00000038375	-0.229987505	-0.064463593	-0.165523912
E1BDG5	WNT5A	bta:530005; E1BDG5	ENSBTAG00000020221	-0.217977131	-0.195015982	-0.022961148
A0AAA9T6K1				-0.060968017	-0.109213099	0.048245082
Q5E9J8	RIC8A	bta:521037; Q17QK2; Q5E9J8		-0.110037595	0.029774036	-0.139811631
C3V9V7	TMED2	bta:100140040; C3V9V7	ENSBTAG00000039630	0	0.028071757	-0.028071757
B0JYN8	PSMB3	B0JYN8; bta:533874		0.046362057	0.049505185	-0.003143128
A0AAA9TKZ5	CFL2	A0AAA9TKZ5	ENSBTAG00000015053	0.177254543	0.279675608	-0.102421065
Q5EA75	PCYT2	bta:510274; Q5EA75		-0.088446182	-0.136360116	0.047913934
Q2T9L9	GTF2F2	bta:509259; Q2T9L9	ENSBTAG00000034495; ENSBTAG00000049772	0.02739833	0.023106721	0.00429161
Q0VC21	MRPL15	bta:510347; Q0VC21	ENSBTAG00000001174	-0.126645937	-0.10890766	-0.017738277
A0A3Q1LVW7	TBL3	A0A3Q1LVW7	ENSBTAG00000020366	-0.216480146	-0.156504486	-0.059975661
A0AAA9S0B0	DDI2	A0AAA9S0B0	ENSBTAG00000039662	0.143172274	0.220768679	-0.077596405
F1MB04	CASP3	F1MB04	ENSBTAG00000015874	0.030373649	-0.01788152	0.048255169
Q17R06	RAB21	bta:507488; Q17R06	ENSBTAG00000053598	0.246128695	0.211430172	0.034698523
A0A3Q1MK97	AHNAK2	A0A3Q1MK97	ENSBTAG00000048906	-0.049070607	0.480081031	-0.529151638
A0AAA9SEF4	NSRP1	A0AAA9SEF4	ENSBTAG00000019341	0.054966459	-0.125106498	0.180072957
A0A3Q1LRS4	SMARCA2	A0A3Q1LRS4	ENSBTAG00000007494	0.013445531	-0.165878168	0.179323699
P51122	ANP32A	bta:538427; P51122; P55930	ENSBTAG00000012365	-0.016791984	-0.058198247	0.041406262
A8E658	PPIG	A8E658; bta:783720; F1MD69	ENSBTAG00000027789	-0.029839637	-0.037396989	0.007557351
A0AAF6YW73	NAXD	A0AAF6YW73	ENSBTAG00000011466	-0.073017986	0.051591732	-0.124609719
A0AAA9TPL0	SUN1	A0AAA9TPL0	ENSBTAG00000046498	0.070389328	0.142098098	-0.07170877
A0A3Q1M273	PBXIP1	A0A3Q1M273	ENSBTAG00000004552	-0.030684706	0.042109296	-0.072794002
F6RSB3	ZRANB2	F6RSB3	ENSBTAG00000002627	0.081743149	0.06946482	0.01227833
Q08DH9	CTCF	bta:517097; F1MK31; Q08DH9	ENSBTAG00000013757	-0.014797002	-0.034262825	0.019465823
F1N318	VTA1	bta:506430; F1N318	ENSBTAG00000005851	0.081376552	0.049374611	0.032001942
Q75WB5	OPLAH	bta:408006; F1ME97; Q75WB5		-0.281045814	0.056170405	-0.337216219
Q0VCL3	ATG3	bta:508571; Q0VCL3	ENSBTAG00000009084	0.22490364	0.111411376	0.113492264
F1MZ61	SMG8	F1MZ61	ENSBTAG00000003365	-0.127935902	0.009705818	-0.13764172
E1BN60	SLC30A1	bta:522265; E1BN60	ENSBTAG00000010909	-0.434539404	-0.524351738	0.089812334
A4IFT6	TMED7	A4IFT6; bta:100125926	ENSBTAG00000035030	0.069695224	0.075238728	-0.005543504
A4FUC0	MRPL37	A4FUC0; bta:510127	ENSBTAG00000010715	-0.194473718	-0.109213099	-0.085260618
A0AAF6ZJH4	VPS28	A0AAF6ZJH4	ENSBTAG00000026320	0.146294941	0.220851903	-0.074556962
G5E6P3	GNA13	G5E6P3	ENSBTAG00000046174	0.035522695	0.154628814	-0.119106119
A0A452DJ27	AGFG1	A0A452DJ27	ENSBTAG00000021323	0.055763272	0.127779638	-0.072016367
A0A3Q1NI16	HS1BP3	A0A3Q1NI16	ENSBTAG00000015490	0.290886526	0.339704174	-0.048817648
E1BAB5	GOLIM4	E1BAB5	ENSBTAG00000018727	-0.044807795	-0.328930588	0.284122793
A0AAA9TFK3	COG5	A0AAA9TFK3	ENSBTAG00000005526	-0.072574602	-0.09170454	0.019129938
A0A3S5ZPR2	MELK	A0A3S5ZPR2	ENSBTAG00000021686	-0.442485557	-0.500802053	0.058316496
A0AAA9SUW0	OSBPL11	A0AAA9SUW0	ENSBTAG00000006471	0.148729819	0.105065158	0.043664661
A0AAF6Z126	PHF6	A0AAF6Z126	ENSBTAG00000015196	0.200800976	0.155669006	0.045131971
F6R277	TRMT1	F6R277	ENSBTAG00000019325	-0.156045067	-0.009110418	-0.146934649
A0A3S5ZP65	MEPCE	A0A3S5ZP65; bta:519969	ENSBTAG00000016763	0.208014775	0.161205677	0.046809098
E1B777	WIPF2	bta:616849; E1B777	ENSBTAG00000038126	-0.041080985	-0.090783602	0.049702617
Q0P5H2	DNTTIP2	bta:511258; Q0P5H2	ENSBTAG00000047679	0.1155944	0.078563669	0.037030731
A7YWC9	UTP25	A7YWC9; bta:510385; F1MDA2	ENSBTAG00000020886	0.050730802	0.216740754	-0.166009951
Q58DS5	RAB13	bta:514541; Q32KZ4; Q58DS5		-0.116209205	0.115670207	-0.231879412
A5PJV9	DTYMK	A5PJV9; bta:506946		-0.150690956	-0.071308535	-0.079382421
Q58D84	FSTL1	bta:534482; Q58D84	ENSBTAG00000022155	-0.430634354	-0.512912038	0.082277684
A0A3Q1MRE3	AKAP13	A0A3Q1MRE3	ENSBTAG00000037383	0.00805527	0.072464983	-0.064409713
A0A3Q1M1V1	TOPBP1	A0A3Q1M1V1	ENSBTAG00000000526	-0.110295287	-0.024271543	-0.086023744
Q5E988	RPS5	bta:506229; Q3T0I8; Q5E988	ENSBTAG00000015989	0.01918702	-0.073140648	0.092327668
F1N735	KDM2A	F1N735	ENSBTAG00000044032	-0.013894442	-0.12511192	0.111217479
Q9N285	MTCH2	A6QR21; bta:337927; F1MR38; Q5E9I9; Q9N285	ENSBTAG00000018742	-0.03493379	-0.097072475	0.062138685
Q0V8M0	KRI1	bta:511427; Q0V8M0	ENSBTAG00000010729	-0.088475188	0.086414752	-0.17488994
F1MMU3	BRD3	F1MMU3	ENSBTAG00000010582	-0.234396091	-0.381336284	0.146940193
A0A3Q1NDF8	NPC1	A0A3Q1NDF8	ENSBTAG00000015195	0.219009782	0.669175505	-0.450165723
F1N6W2	PPIL4	F1N6W2	ENSBTAG00000016980	-0.257700962	-0.005949266	-0.251751695
A0A3Q1MFA8	GNL3L	A0A3Q1MFA8	ENSBTAG00000031564	0.067462968	-0.021648563	0.089111531
A0A3Q1MT88	BAX	A0A3Q1MT88	ENSBTAG00000013340	0.172550471	0.290849784	-0.118299313
A0AAA9S4Z4	AKT1	A0AAA9S4Z4	ENSBTAG00000017636	-0.109832196	-0.135610637	0.025778441
A0A140T872	DHFR	A0A140T872; bta:508809	ENSBTAG00000007681	-0.155843114	0.034107854	-0.189950969
Q2HJD4	CSTF2	G3MZ78; Q2HJD4		0.136372442	0.024662054	0.111710387
F1MMV6	CAB39L	F1MMV6	ENSBTAG00000034222	0.201139872	0.146474897	0.054664976
P01045	KNG2	bta:280833; P01045; P01047		0.110042778	0.385677221	-0.275634443
A0A452DIR5	TSFM	A0A452DIR5	ENSBTAG00000016912	0.098200653	0.075001037	0.023199616
A0A3Q1M9H9	NUP62	A0A3Q1M9H9; bta:100138517	ENSBTAG00000050948	0.097730583	0.091060691	0.006669892
Q2TBT5	RNASEH2A	bta:512830; Q2TBT5	ENSBTAG00000009661	-0.036525876	0.029982866	-0.066508742
A0A3Q1M647	CAMK2D	A0A3Q1M647	ENSBTAG00000014463	-0.002804073	0.053617516	-0.056421589
A0A3Q1MN47	ARHGEF12	A0A3Q1MN47	ENSBTAG00000021343	0.030712357	0.02200391	0.008708447
F2Z4J6	GTF2B	F2Z4J6	ENSBTAG00000000504	0.22562856	0.117220656	0.108407905

Accession	Gene_Symbol	Gene_ID	Ensembl_Gene ID	log2(ratio(OV24H/OV0H))	log2(ratio(OV24R/OV0H))	log2(ratio(OV24H/OV24R))
F1MEM4	DHX8	F1MEM4	ENSBTAG00000008339	0.010540285	-0.06352939	0.074069674
Q76I81	RPS12	A1XED7; bta:326582; Q76I81; Q862U6	ENSBTAG00000001360	0.03608887	0.028319896	0.007768974
A0A3Q1NAY5	DNAJC9	A0A3Q1NAY5; bta:510898	ENSBTAG00000000949	0.293563204	0.264629425	0.028933778
A6QLS6	FRMD6	A6QLS6; bta:530910	ENSBTAG00000031707	0.00138654	-0.001387874	0.002774414
E1BF95	PPM1F	E1BF95	ENSBTAG00000006938	-0.196857329	-0.184257292	-0.012600037
F6RT22	HABP4	F6RT22	ENSBTAG00000011586	0.131395309	0.016093976	0.115301333
A0AAA9SG84	TBC1D23	A0AAA9SG84	ENSBTAG00000015010	0.64455806	0.389807951	0.25475011
E1BCX3	ZSWIM8	E1BCX3	ENSBTAG00000012657	-0.126482573	0.077136714	-0.203619287
Q2KIT5	CRELD2	bta:515222; Q2KIT5		0.309266624	0.290779396	0.018487227
A0AAF6Z482	METTL13	A0AAF6Z482	ENSBTAG00000017721	-0.073026471	-0.151528445	0.078501974
F1MCX1	GPC4	bta:540179; F1MCX1	ENSBTAG00000020644	-0.157451638	-0.144512582	-0.012939056
F1MNS0	HERC1	F1MNS0	ENSBTAG00000019474	-0.086156644	-0.069971216	-0.016185428
A0A3Q1M6N5	BMP2K	A0A3Q1M6N5	ENSBTAG00000001126	-0.037929456	0.071622928	-0.109552383
F1MSE5	ARMH3	F1MSE5	ENSBTAG00000013120	0.065053387	0.044608618	0.020444769
A0AAA9SWH3	PATL1	A0AAA9SWH3	ENSBTAG00000005913	-0.014323621	-0.053230114	0.038906494
A0A3Q1M011	STAU2	A0A3Q1M011	ENSBTAG00000002076	-0.224468541	-0.031459926	-0.193008615
A7YWD2	IPO13	A7YWD2; bta:523399		0.065555222	0.147225953	-0.081670732
A4FUF0	GLYR1	A4FUF0; bta:539636; Q58DC3	ENSBTAG00000001731	-0.047965325	-0.082999377	0.035034051
F1MJQ5	NDUFAF7	F1MJQ5	ENSBTAG00000001113	0.196481015	0.106585934	0.089895081
Q0VCY3	SURF6	bta:511610; Q0VCY3	ENSBTAG00000011554	0.080252956	0.131377421	-0.051124465
A0AAA9SDU1	CWC22	A0AAA9SDU1; bta:781016	ENSBTAG00000021726	-0.158219501	-0.14678892	-0.011430581
A0AAF6Z059	RHOC	A0AAF6Z059	ENSBTAG00000014299	0.047305715	-0.021373651	0.068679366
A0AAA9U0Y4	OCRL	A0AAA9U0Y4	ENSBTAG00000018247	-0.10864126	-0.047106392	-0.061534868
P55906	TGFBI	A7YWB6; bta:539596; P55906		-0.364007027	-0.684318656	0.32031163
A0AAA9SZH9	TRIM56	A0AAA9SZH9; bta:514896	ENSBTAG00000012038	0.541198058	0.327627142	0.213570917
Q17QK8	SPR	A7YKV0; bta:533836; Q17QK8		0.147024599	0.17118665	-0.024162051
Q1LZB2	NCK1	bta:536154; F1MBQ7; Q1LZB2	ENSBTAG00000018427	0.104546776	0.218946149	-0.114399373
Q3ZBL4	LZTFL1	bta:512570; Q3ZBL4	ENSBTAG00000001484	-0.180652531	-0.064020864	-0.116631667
Q3MI03	OGFOD1	bta:514141; Q3MI03		-0.246289018	-0.215506036	-0.030782982
A0AAA9T5H0	MPP1	A0AAA9T5H0	ENSBTAG00000013046	-0.189383448	-0.124919855	-0.064463593
A5D7C2	SDAD1	A5D7C2; bta:507675		-0.308710598	-0.179995942	-0.128714657
Q2NKU1	DUT	bta:507945; Q2NKU1	ENSBTAG00000011998	-0.218231198	-0.453038688	0.234807489
A0A3Q1M3K3	SMAD5	A0A3Q1M3K3; bta:768050	ENSBTAG00000005062	-0.479775656	-0.64702995	0.167254294
A0A452DIH1	MRPL44	A0A452DIH1	ENSBTAG00000011514	-0.238759354	-0.164434243	-0.074325111
P17697	CLU	A5D983; bta:280750; P17697; Q148K1	ENSBTAG00000005574	0.489751227	0.471861068	0.017890159
A0A3Q1LLL1	SH3GLB2	A0A3Q1LLL1	ENSBTAG00000010626	0.099439007	0.139479368	-0.04004036
P11456	M6PR	A1L5A0; bta:281291; P11456; Q0VCN7	ENSBTAG00000018207	-0.040015679	-0.019128312	-0.020887366
A0AAA9SXY1	CHAF1A	A0AAA9SXY1	ENSBTAG00000008181	-0.340571006	-0.370982319	0.030411313
F6RDU5	SRSF11	F6RDU5	ENSBTAG00000015474	-0.063062268	-0.113030727	0.049968459
A0A3Q1MW87	JUND	A0A3Q1MW87	ENSBTAG00000052791	0.080301628	0.134409033	-0.054107405
A2VE70	VAC14	A2VE70; bta:533202		-0.158807037	-0.040507724	-0.118299313
Q58DT8	WDR55	A1L559; bta:508709; Q58DT8	ENSBTAG00000015042	-0.094996514	-0.147296091	0.052299577
A0A3Q1M489	SNRPB	A0A3Q1M489	ENSBTAG00000001038	-0.010003869	0.085702486	-0.095706355
A0A3Q1LXW0	TRIM33	A0A3Q1LXW0; bta:533296	ENSBTAG00000001499	0.03889678	0.048812249	-0.009915469
P61284	RPL12	A5PJB1; bta:404133; P61284; Q3SZV1	ENSBTAG00000006963	-0.046908743	-0.059265949	0.012357207
Q2YDJ9	GMPPB	bta:514161; Q2YDJ9		0.139562066	0.060645009	0.078917057
F6RBQ9	RBM3	F6RBQ9	ENSBTAG00000025848	-0.241606554	-0.287460448	0.045853894
A0AAA9U1E9	GALNS	A0AAA9U1E9	ENSBTAG00000004568	-0.027808659	-0.041241671	0.013433012
F1MWJ3	ADPRS	F1MWJ3	ENSBTAG00000021715	-0.315126736	-0.175128596	-0.13999814
A4FV55	PPP2R5C	A4FV55; bta:505757	ENSBTAG00000020192	-0.24448934	-0.184146434	-0.060342906
F1MD32	CREBBP	A0A8J8YPW6; F1MD32; M5FJS5	ENSBTAG00000026403	-0.376633987	-0.074353794	-0.302280193
G3N3E5	WDR5	G3N3E5	ENSBTAG00000047031	0.070175294	0.137094886	-0.066919592
E1B782	HERC2	bta:535440; E1B782	ENSBTAG00000013916	-0.201952231	-0.129123858	-0.072828373
E1BA60	RAB32	E1BA60	ENSBTAG00000009784	0.155367896	0.053199567	0.102168329
Q3SZI2	RIPK2	bta:534407; Q3SZI2	ENSBTAG00000015271	0.074919735	-0.03211756	0.107037295
A0AAA9TCA1	PUDP	A0AAA9TCA1	ENSBTAG00000000207	0.007195501	-0.008682243	0.015877745
A0AAA9TL09	CTNNBL1	A0AAA9TL09	ENSBTAG00000007658	-0.121105034	-0.132064246	0.010959212
P62992	RPS27A	bta:286839; O97577; P02248; P02249; P02250; P0CG52; P62990; P62992; P80169; Q01235; Q24K23; Q28169; Q28170; Q29120; Q3T0V5; Q3ZCE3; Q862C1; Q862F4; Q862M4; Q862T5; Q862X8; Q91887; Q91888	ENSBTAG00000015473	-0.131789873	-0.102298576	-0.029491297
A0AAF6YM07	BPNT1	A0AAF6YM07	ENSBTAG00000004064	0.114603122	0.115914067	-0.001310945
F1MBE0	CWC27	F1MBE0	ENSBTAG00000000037	-0.061054035	-0.07497977	0.013925736
Q32KL5	EMC8	bta:510727; Q32KL5		0.068598756	0.071711367	-0.003112612
A0AAA9RT28	STAG1	A0AAA9RT28; bta:539234	ENSBTAG00000035437	0.017364643	-0.036861868	0.054226512
Q1LZ90	CLNS1A	bta:613968; F1N1B1; Q1LZ90	ENSBTAG00000019930	0.173037613	0.166111556	0.006926058
Q58DU7	SH3BGRL	bta:614007; Q58DU7		0.373272862	-0.00757322	0.380846082
A0AAA9SW30	CADM1	A0AAA9SW30	ENSBTAG00000000977	-0.169403891	-0.11872392	-0.05067997
Q05B85	JPT2	A0A8J8XE70; bta:618512; M5FK16; Q05B85	ENSBTAG00000002209	0.253157726	0.352976531	-0.099818805
A0AAA9S8T0	UBE2K	A0AAA9S8T0	ENSBTAG00000020175	0.116568377	0.167996616	-0.051428239
A0AAA9SWA1	STK26	A0AAA9SWA1	ENSBTAG00000019652	0.081628762	0.019365325	0.062263437
F1N444	LTBP1	F1N444	ENSBTAG00000019839	-0.146206802	-0.249300295	0.103093493
F1MV85	PBDC1	bta:511011; F1MV85	ENSBTAG00000003609	0.299457814	0.220048481	0.079409333
A0AAA9S5H7	CELF2	A0AAA9S5H7	ENSBTAG00000008937	-0.065357201	0.01553578	-0.080892981
Q32KP9	NUTF2	bta:614921; Q32KP9		0.582073064	0.149576356	0.432496708
E1BJC0	CARNMT1	bta:538441; E1BJC0	ENSBTAG00000008204	0.285971217	0.279585582	0.006385635
A0AAF7A0P8	CHID1	A0AAF7A0P8	ENSBTAG00000009899	0.063578796	0.241739627	-0.178160832
A0A3Q1M902	HPF1	A0A3Q1M902	ENSBTAG00000020132	-0.034626343	0.156557778	-0.191184121
F2Z4G3	RBM22	F2Z4G3	ENSBTAG00000010366	0.255119915	0.102801857	0.152318058
A0AAA9S0Y0	IGF2BP3	A0AAA9S0Y0	ENSBTAG00000019406	-0.01280507	0.094569421	-0.10737449
E1BMW9	PURA	bta:782746; E1BMW9	ENSBTAG00000008881	0.03656148	0.113613624	-0.077052144
A0AAA9T3W1	NIF3L1	A0AAA9T3W1	ENSBTAG00000018282	0.105133549	0.146539811	-0.041406262
A0A3Q1MX61	UEVLD	A0A3Q1MX61	ENSBTAG00000013568	0.069540933	0.085016114	-0.015475181
P35705	PRDX3	bta:281998; P35705; Q3SZA8	ENSBTAG00000008731	0.295455884	0.136561919	0.158893964
E1BD67	SAAL1	E1BD67	ENSBTAG00000008804	0.09965225	0.539365634	-0.439713384
A0AAA9TSJ7	SEC13	A0AAA9TSJ7	ENSBTAG00000017825	0.11880743	0.142841122	-0.024033692
F1MDT8	SETD2	F1MDT8	ENSBTAG00000002948	-0.117333371	-0.084509524	-0.032823847
A6QQV4	METTL3	A6QQV4; bta:540339		-0.006610298	0.124706249	-0.131316547
A0AAA9TPZ1	NUDT5	A0AAA9TPZ1	ENSBTAG00000019509	0.118970812	0.121506305	-0.002535493
A0A3Q1LVP1	PTK2B	A0A3Q1LVP1	ENSBTAG00000005958	-0.214801492	-0.083556958	-0.131244533
A4FUE3	RBBP5	A4FUE3; bta:510455		0.059533323	0.03788476	0.021648563
F1MQB8	SIRT1	F1MQB8	ENSBTAG00000014023	0.078822272	0.311294699	-0.232472428
A0AAA9SNN6	SARNP	A0AAA9SNN6	ENSBTAG00000020662	0.087023062	0.098239407	-0.011216344
A0AAA9SGI8	COPS7A	A0AAA9SGI8	ENSBTAG00000011111	-0.040898031	0.041222663	-0.082120694
G3MYD5	IVNS1ABP	bta:514940; G3MYD5	ENSBTAG00000014249	-0.37658748	-0.600170735	0.223583255
A0AAA9S4Z0	RALY	A0AAA9S4Z0	ENSBTAG00000016524	-0.115714835	-0.062640719	-0.053074116
A6QLS9	RAB10	A6QLS9; bta:783373	ENSBTAG00000000985	0.179424973	0.078412893	0.10101208
Q0P5G8	ACAD11	bta:526956; F1MNX2; Q0P5G8	ENSBTAG00000031010	0.138162439	0.027289222	0.110873217
A0AAA9SYT0	UPF3B	A0AAA9SYT0	ENSBTAG00000035615	0.179821038	0.110558375	0.069262662
A0AAF6YWB5	IMPA1	A0AAF6YWB5	ENSBTAG00000011709	-0.063743884	-0.031519914	-0.03222397
A0A3Q1M792	SUZ12	A0A3Q1M792; bta:538774	ENSBTAG00000018752	-0.196116342	-0.32699315	0.130876808
A0A3Q1LJH2	PRUNE1	A0A3Q1LJH2	ENSBTAG00000015336	0.072252072	0.101212251	-0.028960179
A0AAA9S140	MADD	A0AAA9S140	ENSBTAG00000021700	0.100338621	0.063344413	0.036994207
A0AAA9U0N9	ABI1	A0AAA9U0N9	ENSBTAG00000005136	0.11189288	0.163446791	-0.051553911
F1MR28	OXR1	F1MR28	ENSBTAG00000008285	0.120019146	0.209564873	-0.089545726
F1MUP3	SEC62	bta:538938; F1MUP3	ENSBTAG00000039442	0.004232849	-0.054719545	0.058952394
A0AAA9U078	C15H11orf58	A0AAA9U078	ENSBTAG00000006755	0.102923694	0.145093148	-0.042169454
F1MFW8	NOC4L	F1MFW8	ENSBTAG00000011889	-0.152170256	-0.080428264	-0.071741991
Q17QW1	CDC42EP1	bta:511099; Q17QW1	ENSBTAG00000030258	0.199308808	0.294796064	-0.095487256
F6RJG0	HMGCS1	F6RJG0	ENSBTAG00000011839	0.08246216	0.079047496	0.003414664
A0A3Q1M809	MED24	A0A3Q1M809	ENSBTAG00000021468	-0.141043881	0.015475181	-0.156519062
A5PKH0	TOR1AIP2	A5PKH0; bta:782450; F1N6U6	ENSBTAG00000035230	0.197323992	0.010459772	0.18686422
F1MXY9	SRSF6	F1MXY9	ENSBTAG00000008527	-0.036600011	-0.149114814	0.112514804
A0A452DJR2	JMJD6	A0A452DJR2	ENSBTAG00000019153	-0.012397026	-0.235827358	0.223430331
E1BNH9	OBSL1	E1BNH9	ENSBTAG00000040338	-0.36923381	-0.250913842	-0.118319967
Q0P5F2	PSMG1	bta:614817; Q0P5F2	ENSBTAG00000013600	0.005418579	-0.017752269	0.023170848
Q3T0P5	CASP6	bta:538409; Q3T0P5		-0.0100849	-0.063927327	0.053842427
A0AAA9T728	ANK2	A0AAA9T728	ENSBTAG00000002392	0.089411456	0.503432615	-0.414021159
A0AAA9TQU6	CORO2A	A0AAA9TQU6	ENSBTAG00000007659	-0.105031216	0.05293416	-0.157965376
A0A3Q1MNS5	UBE2V2	A0A3Q1MNS5	ENSBTAG00000023218	0.097998438	0.074845363	0.023153075
A0AAA9RRZ7	WDR77	A0AAA9RRZ7	ENSBTAG00000014102	0.061902439	0.137777306	-0.075874867
A0A3Q1LJG6	AOX1	A0A3Q1LJG6	ENSBTAG00000009725	-0.9763358	-0.518897051	-0.457438749
A6QLZ3	ARSB	A6QLZ3; bta:538401	ENSBTAG00000008341	-0.043863814	0.135397912	-0.179261726
A0AAA9TJZ3	NFX1	A0AAA9TJZ3	ENSBTAG00000012385	-0.114742086	-0.092619275	-0.022122811
A0A3Q1LLQ5	RBM47	A0A3Q1LLQ5; bta:540831	ENSBTAG00000002356	-0.201785732	0.271446646	-0.473232377
E1BE36	ARMCX3	bta:516747; E1BE36	ENSBTAG00000037996	0.224526586	0.140558284	0.083968302
Q0VCJ2	APIP	bta:508345; Q0VCJ2	ENSBTAG00000018257	0.044602087	-0.022131902	0.066733989
A0AAA9T3B8	CD2BP2	A0AAA9T3B8	ENSBTAG00000015486	-0.178264302	-0.090063698	-0.088200604
G1K1U4	POLD3	G1K1U4	ENSBTAG00000016869	-0.340075442	-0.351074441	0.010998999
A0JNF4	PRIM2	A0JNF4; bta:514682; F1MP18	ENSBTAG00000007937	-0.077591124	0.00443679	-0.082027914
Q08DA3	USP16	bta:519992; F1N2B1; Q08DA3		-0.42462909	-0.441463529	0.016834439
Q08DJ7	AAR2	Q08DJ7	ENSBTAG00000001645	0.184988381	0.096922568	0.088065813
F1MZA7	URB1	bta:530483; F1MZA7	ENSBTAG00000012412	-0.269115904	-0.025042587	-0.244073317
Q0P5J0	QRICH1	A5D7N1; bta:511970; Q0P5J0		-0.034765418	-0.113363014	0.078597596
Q2KI46	CDC42EP3	bta:538967; Q2KI46	ENSBTAG00000023736	0.334117504	0.177998302	0.156119202
E1BCU7	METTL16	E1BCU7	ENSBTAG00000017440	-0.158417402	-0.12151782	-0.036899583
A8E4R4	EXOSC6	A8E4R4; bta:100126247	ENSBTAG00000053923	0.064059137	0.019518156	0.044540981
A0A3Q1MXF1	THYN1	A0A3Q1MXF1	ENSBTAG00000012936	-0.175042761	0.026992275	-0.202035037
A7MB43	MTMR9	A7MB43; bta:539174		0.006158793	0.018398061	-0.012239268
Q29RM7	FAF1	bta:531770; Q29RM7	ENSBTAG00000014482	-0.095860015	-0.118055762	0.022195746
F1MHR4	PIP4K2A	bta:533289; F1MHR4	ENSBTAG00000018033	-0.0061002	0.034584237	-0.040684436
A5PK76	ASPSCR1	A5PK76; bta:515670		-0.219951312	-0.237126456	0.017175144
A8PUI7	SKA3	A8PUI7; bta:509921	ENSBTAG00000003314	0.012149083	0.052412953	-0.04026387
A0AAA9TVR1	MICU1	A0AAA9TVR1	ENSBTAG00000005807	-0.113119365	-0.097089255	-0.01603011
A0AAA9TQM8	PUS1	A0AAA9TQM8	ENSBTAG00000012990	0.039270118	0.139528765	-0.100258648
A0A3Q1N5S1	RFC1	A0A3Q1N5S1	ENSBTAG00000014515	0.011898583	-0.187958924	0.199857508
G3MZ11	CRYBG3	G3MZ11	ENSBTAG00000047147	0.030246287	0.090336737	-0.06009045
A0A452DIE3	APRT	A0A452DIE3	ENSBTAG00000000639	0.034656482	0.042081589	-0.007425107
F1MKC7	HELZ	F1MKC7	ENSBTAG00000004746	0.315475697	0.369705726	-0.054230029
F1N270	ATP6V1D	bta:404152; F1N270	ENSBTAG00000016309	-0.003950788	0.076825339	-0.080776126
A0A3Q1MKV8	PLEKHO2	A0A3Q1MKV8	ENSBTAG00000008411	-0.1168813	-0.011271112	-0.105610188
A0AAA9SZH8	GALK2	A0AAA9SZH8	ENSBTAG00000004011	0.037509505	0.022889716	0.014619789
F1MSA1	PPT1	F1MSA1	ENSBTAG00000013367	-0.461599021	-0.205740234	-0.255858787
Q2KJE0	TAX1BP1	bta:535589; Q2KJE0	ENSBTAG00000019020	0.662776585	0.296648686	0.366127899
A0AAA9SJP4	DHRS7	A0AAA9SJP4	ENSBTAG00000020729	0.187553541	0.140512274	0.047041267
P00921	CA2	bta:280740; P00921; Q3ZBJ5; Q865Y7		0.053439259	0.043869108	0.009570151
A0A3Q1M8C6	VIPAS39	A0A3Q1M8C6	ENSBTAG00000020475	0.105224876	0.111974338	-0.006749463
F6Q4T4	REPS1	F6Q4T4	ENSBTAG00000012299	0.030996594	0.080324306	-0.049327712
F1MC10	P3H4	F1MC10	ENSBTAG00000025752	-0.144540724	-0.186314323	0.041773599
A0AAA9S957	NRBP1	A0AAA9S957	ENSBTAG00000018153	0.058718275	0.053093726	0.005624549
Q3T0V3	EIF3K	bta:515326; Q3T0V3	ENSBTAG00000011212	0.259606636	-0.12194405	0.381550685
A0AAA9S3E1	FBXO3	A0AAA9S3E1	ENSBTAG00000010071	-0.295397229	-0.274955431	-0.020441798
A0AAA9U053	EP400	A0AAA9U053	ENSBTAG00000020423	-0.065979661	-0.026887689	-0.039091971
A0AAA9S711	PSMA3	A0AAA9S711	ENSBTAG00000002808	0.030040763	-0.016065812	0.046106575
F1MUZ0	EBNA1BP2	F1MUZ0	ENSBTAG00000021544	0.160633399	0.100603941	0.060029458
A0AAA9T3V8	PLPBP	A0AAA9T3V8	ENSBTAG00000011075	0.157420524	0.247190128	-0.089769604
E1BCT7	WDR33	bta:541270; E1BCT7	ENSBTAG00000005987	-0.286127373	-0.362362959	0.076235586
E1BFK4	SMG6	E1BFK4	ENSBTAG00000000267	-0.385906054	-0.132149461	-0.253756592
F1MUX7	GTF2E2	F1MUX7	ENSBTAG00000012691	-0.006640725	-0.018336059	0.011695334
Q3MHR5	SRSF2	bta:508312; Q3MHR5	ENSBTAG00000018258	-0.023602899	0.069971216	-0.093574115
Q0X0C4	HEXIM1	bta:539696; Q0X0C4	ENSBTAG00000006056	0.111393	-0.101131134	0.212524134
P02316	HMGN1	bta:614915; P02316; Q3T0D1		0.117865503	0.115135711	0.002729793
A0AAA9SIT5	IKBKB	A0AAA9SIT5	ENSBTAG00000007599	0.06529146	0.103093493	-0.037802033
E1BGU0	HEATR5A	E1BGU0	ENSBTAG00000022847	-0.109694865	-0.071258683	-0.038436182
Q3SX42	CHMP2B	bta:615954; Q3SX42		0.306238113	0.17145409	0.134784022
A0A3Q1MJG1	TTC4	A0A3Q1MJG1	ENSBTAG00000015931	0.267283879	0.256636635	0.010647244
F1MUZ3	SMAD3	bta:515125; F1MUZ3	ENSBTAG00000012599	0.04548277	0.185245801	-0.139763031
A6QPZ6	TAP1	A6QPZ6	ENSBTAG00000008953	0.020218279	0.028797264	-0.008578985
F1MMW4	AKAP8L	bta:539700; F1MMW4	ENSBTAG00000010439	0.61685031	0.467082221	0.149768089
A0AAA9TKV8	PDAP1	A0AAA9TKV8	ENSBTAG00000044040	0.100676513	-0.007802588	0.108479101
Q5E9L7	VPS16	bta:505361; Q5E9L7		-0.113742166	-0.070714882	-0.043027284
A0AAA9TVL3	RNF31	A0AAA9TVL3	ENSBTAG00000005815	-0.066972554	0.361247416	-0.42821997
A0AAF7AI76	ALDH3B1	A0AAF7AI76	ENSBTAG00000013093	-0.494022851	-0.011267111	-0.48275574
A0AAA9TCR7	RAB18	A0AAA9TCR7	ENSBTAG00000009871	0.003855757	-0.267933205	0.271788963
A0AAA9TDL0	C11H9orf78	A0AAA9TDL0	ENSBTAG00000020307	0.026717338	-0.244280446	0.270997784
Q5E948	Ociad1	bta:533520; Q3ZBB3; Q5E948		0.208523637	0.147739129	0.060784509
E1BJS0				-0.037393757	0.02591281	-0.063306568
A0A3Q1MIC9	DNM3	A0A3Q1MIC9	ENSBTAG00000014319	0.082317348	-0.068045387	0.150362735
A0A3Q1LLT2	EXOSC8	A0A3Q1LLT2	ENSBTAG00000011855	0.040597994	-0.006348503	0.046946497
G3N315	STAM2	G3N315	ENSBTAG00000001887	0.085462507	0.245160767	-0.15969826
Q0V8R6	HEXA	bta:504468; Q0V8R6	ENSBTAG00000012981	-0.019285671	0.126881088	-0.146166759
A0JN63	CSTF1	A0JN63; bta:507051	ENSBTAG00000006639	0.007802588	0.010911846	-0.003109258

Accession	Gene_Symbol	Gene_ID	Ensembl_Gene ID	log2(ratio(OV24H/OV0H))	log2(ratio(OV24R/OV0H))	log2(ratio(OV24H/OV24R))
A5PKK7	GORAB	A5PKK7		0.050626073	0.067626426	-0.017000353
A0AAA9TRT3	MORC2	A0AAA9TRT3	ENSBTAG00000009963	0.262221849	0.161234041	0.100987808
E1BMI6	GNL3	E1BMI6	ENSBTAG00000014549	-0.136866713	0.253448357	-0.39031507
A0AAA9RYW7	SPART	A0AAA9RYW7	ENSBTAG00000008040	0.54417886	0.314970544	0.229208316
A0AAA9SJA2	STK38	A0AAA9SJA2	ENSBTAG00000011126	0.284088038	0.223100493	0.060987545
P11116	LGALS1	bta:326598; P11116; P11945; Q54A27	ENSBTAG00000015089	0.098684275	-0.144613913	0.243298188
P29105	HPCAL1	A5PKL3; bta:513870; P29105; P29106	ENSBTAG00000004259	0.202661788	0.122373999	0.080287789
A0A3Q1LT47	ANKRD44	A0A3Q1LT47	ENSBTAG00000031696	-0.171870646	-0.032775036	-0.13909561
A0A140T8D6	BCAR3	A0A140T8D6	ENSBTAG00000003245	-0.183339645	-0.180488468	-0.002851177
A0A3Q1LQW5	SLC38A10	A0A3Q1LQW5	ENSBTAG00000018271	0.044727806	-0.122031014	0.16675882
Q1JQE6	NCEH1	bta:534212; Q1JQE6	ENSBTAG00000020073	-0.226254027	-0.001157397	-0.22509663
A0A452DI54	CCDC25	A0A452DI54	ENSBTAG00000006547	-0.122920614	-0.087373035	-0.035547579
E1BGR7				-0.227114832	-0.225538977	-0.001575855
Q3ZBK3	SPC25	bta:505011; Q3ZBK3		-0.126029653	-0.340214323	0.214184669
E1BHF6	SIPA1L3	E1BHF6	ENSBTAG00000018225	-0.199459899	-0.078229155	-0.121230745
A4FV54	RAB8A	A4FV54; bta:100125881	ENSBTAG00000038696	0.086222118	0.00300875	0.083213368
Q05588	PLAUR	A5PJ83; bta:281983; Q05588	ENSBTAG00000013125	0.010627636	0.085422143	-0.074794507
Q8MJ50	OSTF1	bta:281961; Q8MJ50	ENSBTAG00000002746	0.271058577	0.152145084	0.118913493
O97764	CRYZ	bta:281093; O97764; Q0VCY2	ENSBTAG00000003162	0.169925001	0.194548788	-0.024623787
A0AAA9TAV4	ANO6	A0AAA9TAV4	ENSBTAG00000002902	-0.006566671	0.067828755	-0.074395426
F6RBN9	MTX2	F6RBN9	ENSBTAG00000006693	0.142160476	0	0.142160476
F1N063	CAMK1D	F1N063	ENSBTAG00000008650	0.069488587	-0.096598929	0.166087516
Q0VCZ3	YTHDF2	bta:541050; Q0VCZ3	ENSBTAG00000015771	0.020887366	-0.039611856	0.060499222
A0AAA9T0P5	PNPLA6	A0AAA9T0P5	ENSBTAG00000032137	0.353495354	0.352220325	0.001275029
A0AAA9TIR9	SOS1	A0AAA9TIR9; bta:537682	ENSBTAG00000011643	-0.024711753	-0.040930264	0.016218511
A0A3Q1MS93	CDC27	A0A3Q1MS93	ENSBTAG00000002726	-0.079932333	-0.234710311	0.154777979
A4FV10	TMED4	A4FV10; bta:508729		0.071474469	0.12471649	-0.053242021
A0AAA9SHQ6	GPALPP1	A0AAA9SHQ6	ENSBTAG00000009416	0.172139423	0.001661134	0.170478288
A1A4J9	DNAJA3	A0A8J8Y340; A1A4J9; bta:513397; M5FJZ6	ENSBTAG00000010013	0.083892696	0.063734952	0.020157744
E1BP85	UCKL1	E1BP85	ENSBTAG00000015798	0.12081112	0.193250786	-0.072439665
A0AAA9TMM9	NEK7	A0AAA9TMM9	ENSBTAG00000016943	-0.001431955	0.064379228	-0.065811184
P21146	GRK2	bta:282682; P21146	ENSBTAG00000005832	0.072252072	-0.089386318	0.16163839
A0A3Q1MDB6	HDAC7	A0A3Q1MDB6	ENSBTAG00000026819	-0.120401096	-0.108428454	-0.011972642
Q0VC53	DOHH	A5H2K6; A5H2K8; bta:526521; Q0VC53	ENSBTAG00000005272	0.505310252	0.072350844	0.432959407
A0AAF6Z2G3	GAA	A0AAF6Z2G3	ENSBTAG00000016021	0.121079924	0.331997353	-0.210917429
A0AAA9S6V6	HSPE1	A0AAA9S6V6	ENSBTAG00000012589	0.298643412	0.166049802	0.13259361
F1MF02	SLAIN2	F1MF02	ENSBTAG00000021963	0.0044007	0.076941857	-0.081342557
A7E3Q1	TARSL1	A7E3Q1; bta:511740		0.026445252	0.070883486	-0.044438234
F6Q0E0				-0.217565487	-0.174115231	-0.043450256
A0A3Q1LPQ8	ARVCF	A0A3Q1LPQ8	ENSBTAG00000001473	0.104720991	-0.010664735	0.115385725
Q2HJ47	DENR	bta:614238; Q2HJ47	ENSBTAG00000008852	0.238394521	0.175571565	0.062822956
E1BAZ9	EXOG	bta:524343; E1BAZ9	ENSBTAG00000019305	0.130167292	0.156793794	-0.026626502
A0A3Q1M9L7	MAP4K5	A0A3Q1M9L7	ENSBTAG00000014792	0.010547165	0.109181811	-0.098634646
A0A3Q1M337	NDC1	A0A3Q1M337	ENSBTAG00000006625	-0.070054168	-0.057441892	-0.012612276
A0AAF6DM24	GRSF1	A0AAF6DM24	ENSBTAG00000008577	-0.23502003	-0.293253295	0.058233266
F1MPI4	POFUT2	F1MPI4	ENSBTAG00000007818	-0.548250722	-0.235048632	-0.31320209
Q3SZ21	RPP30	bta:615098; Q3SZ21	ENSBTAG00000002973	-0.201070907	-0.022700215	-0.178370692
F6QVL1	POLR1G	F6QVL1	ENSBTAG00000023601	0.085841404	0.228799358	-0.142957954
Q5BIR6	MED17	bta:541303; Q3SYX5; Q5BIR6	ENSBTAG00000003552	-0.080465711	0.046495707	-0.126961418
A0A3Q1NA18	AVEN	A0A3Q1NA18	ENSBTAG00000044029	0.132282667	0.129374007	0.00290866
A0A3Q1LN00	CBL	A0A3Q1LN00	ENSBTAG00000006817	0.162445627	0.143297786	0.019147842
E1BK63	LOC101903301	E1BK63	ENSBTAG00000040367	-0.025090981	-0.109393481	0.0843025
F1MSM5	MTPAP	bta:511412; F1MSM5	ENSBTAG00000004387	0.094366571	0.184300729	-0.089934159
G5E683	MBD2	G5E683	ENSBTAG00000037457	-0.073615195	-0.100058863	0.026443668
A0A3Q1M7Q3	GIT1	A0A3Q1M7Q3	ENSBTAG00000012040	-0.038364522	-0.082064537	0.043700015
F6PRJ0	LRRC8E	bta:507824; F6PRJ0	ENSBTAG00000004300	0.052418534	0.032482311	0.019936223
E1BEM3	CDV3	E1BEM3	ENSBTAG00000035556	0.329659138	0.185629902	0.144029236
A0AAF6YXD6	CNBP	A0AAF6YXD6	ENSBTAG00000012159	-0.134868747	-0.052122731	-0.082746016
A0AAA9S3N0	SERPINE2	A0AAA9S3N0	ENSBTAG00000008717	-0.10897179	0.03208218	-0.14105397
A0A3Q1M9B3	RPL18	A0A3Q1M9B3	ENSBTAG00000015388	-0.076303367	-0.129510733	0.053207366
A0AAA9SVT1	RPL32	A0AAA9SVT1	ENSBTAG00000015283	-0.080782655	-0.106695465	0.02591281
F1MKA6	AARS2	F1MKA6	ENSBTAG00000013722	-0.53236755	-0.324310746	-0.208056804
A0AAF6DLE4	HAUS5	A0AAF6DLE4	ENSBTAG00000013436	-0.004325925	0.045353677	-0.049679603
Q2KJ61	ELP3	bta:784720; Q2KJ61		-0.028249477	-0.055677965	0.027428489
P62248	MYDGF	bta:407769; P62248; Q3T039	ENSBTAG00000018655	0.202760233	0.065857082	0.136903151
A0AAF6Z8G6	CTSK	A0AAF6Z8G6; bta:513038	ENSBTAG00000021035	-0.511500339	-0.331900681	-0.179599658
Q0VCH0	ANP32E	bta:507203; F1MPJ7; Q0VCH0	ENSBTAG00000016730	-0.252442995	-0.138009821	-0.114433173
Q3T083	SDF2L1	bta:517962; Q3T083		0.053726752	-0.034468757	0.088195508
Q2KHT8	DIMT1	bta:509725; Q2KHT8		-0.245112498	-0.304066361	0.058953863
A0A3Q1MH41	SRPK2	A0A3Q1MH41	ENSBTAG00000002175	0.122109276	0.200678328	-0.078569052
A0AAA9SH20	GMPR2	A0AAA9SH20	ENSBTAG00000002715	-0.003122718	-0.047565683	0.044442965
A0A3Q1N340	TRMT1L	A0A3Q1N340	ENSBTAG00000011285	-0.012226302	0	-0.012226302
A5D7R9	NRAS	A5D7R9; bta:506322	ENSBTAG00000046797	0.212348755	0.119321624	0.093027132
A5PK21	CKAP2L	A5PK21; bta:507498; Q0V8I3		0.026472211	-0.107429076	0.133901287
A0AAA9S5X5	HM13	A0AAA9S5X5	ENSBTAG00000008840	-0.136321471	-0.274113852	0.137792381
A2VDK6	WASF2	A2VDK6; bta:504482	ENSBTAG00000018374	-0.007905198	-0.018513298	0.0106081
A0AAF6YYF2	COG4	A0AAF6YYF2	ENSBTAG00000012988	0.01887801	-0.071924927	0.090802937
A0AAA9S0R6	GLIPR2	A0AAA9S0R6	ENSBTAG00000000078	0.224519977	0.342056296	-0.11753632
Q08DV2	GNPNAT1	A0A3Q1MLH0; bta:512299; F1N5F5; Q08DV2	ENSBTAG00000005344	0.148287199	0.04265935	0.10562785
A0AAA9SJN2	PARVA	A0AAA9SJN2	ENSBTAG00000000700	-0.116562685	-0.17563284	0.059070155
A0A3Q1MIQ1	RNGTT	A0A3Q1MIQ1	ENSBTAG00000002725	-0.670361595	-0.463646149	-0.206715447
Q32PB8	RPS21	bta:615178; Q32PB8		-0.131573593	-0.073709686	-0.057863906
A0AAA9STD7	POLR3A	A0AAA9STD7	ENSBTAG00000013259	-0.006126103	0.238890468	-0.245016571
A0A3Q1M4E6	TJAP1	A0A3Q1M4E6	ENSBTAG00000011189	0.029901486	0.215686727	-0.185785242
F1MNU7	YTHDC2	bta:541024; F1MNU7	ENSBTAG00000014099	-0.174330181	-0.026303282	-0.148026899
F1MT65	HDGFL2	F1MT65	ENSBTAG00000013251	-0.194503024	-0.20795134	0.013448316
F1MM80	ASPM	F1MM80	ENSBTAG00000007860	-0.141315857	-0.227841701	0.086525844
Q58DR5	SCAMP3	bta:539670; Q3ZCL2; Q58DR5		0.092585074	0.128324097	-0.035739023
A0AAA9SM15	ADAM17	A0AAA9SM15; bta:517541	ENSBTAG00000001141	-0.016173881	0.331865596	-0.348039476
A0A3Q1N363	NIPSNAP1	A0A3Q1N363	ENSBTAG00000013152	-0.018736563	0.03675855	-0.055495113
A0AAA9SD78	EXOSC7	A0AAA9SD78; bta:100847343	ENSBTAG00000018329	0.060803279	0.116211974	-0.055408695
A0AAF6ZJK8	MRPL38	A0AAF6ZJK8	ENSBTAG00000022009	-0.099127209	-0.005730673	-0.093396536
A0AAA9SUN6	SVIL	A0AAA9SUN6	ENSBTAG00000027444	0.06168595	0.095419565	-0.033733615
A0AAF6DMH4	LANCL2	A0AAF6DMH4	ENSBTAG00000018026	0.207579546	0.198215707	0.009363839
E1BP14	ARHGEF17	E1BP14	ENSBTAG00000012505	0.030246287	0.013689355	0.016556932
Q32LC3	EIF1AX	Q32LC3	ENSBTAG00000017633	0.079434467	-0.024220407	0.103654875
A3KN48	EEFSEC	A3KN48; bta:532824	ENSBTAG00000030962	-0.198376889	-0.055988725	-0.142388164
A0AAA9RUC6	U2AF1	A0AAA9RUC6	ENSBTAG00000011645	0.124258103	0.036182336	0.088075767
A0AAA9S7F8	GNA13	A0AAA9S7F8	ENSBTAG00000046174	-0.090251058	0.615450776	-0.705701834
A0AAA9RSE0	MAN1A1	A0AAA9RSE0	ENSBTAG00000024381	-0.203484657	-0.248631341	0.045146684
F1N3H6	DPY19L1	F1N3H6	ENSBTAG00000005785	-0.03769229	0	-0.03769229
E1BNM2	ITGA8	bta:511976; E1BNM2	ENSBTAG00000007602	-0.131193494	-0.410044236	0.278850742
Q0VD18	LHPP	bta:534183; Q0VD18	ENSBTAG00000010957	-0.093218828	-0.01153503	-0.081683798
F1N1J5	CEP43	F1N1J5	ENSBTAG00000014495	-0.032385087	-0.048854387	0.0164693
Q2KJ44	PTPA	bta:514460; Q2KJ44		0.05718983	0.151056754	-0.093866923
A0A3Q1LSG0				-0.075050705	2.22571278	-2.300763485
A0A3Q1M127	KIDINS220	A0A3Q1M127	ENSBTAG00000007963	0.047534951	-0.127729226	0.175264176
A0AAA9T6L5	AGO1	A0AAA9T6L5	ENSBTAG00000012253	0.190477241	0.214323983	-0.023846742
Q3ZBA3	PLEKHA2	bta:530879; Q3ZBA3	ENSBTAG00000003509	-0.16620141	-0.11260202	-0.053599389
F1N7Q7	COL4A2	F1N7Q7	ENSBTAG00000025210	-0.172610664	-0.131190737	-0.041419927
A0AAA9SCI1	PDXK	A0AAA9SCI1	ENSBTAG00000018186	-0.011994762	0.048290095	-0.060284857
Q9N2J2	GPX4	bta:286809; Q9N2J2	ENSBTAG00000053003	0.005619073	0.04707868	-0.041459607
E1BER5	EPHA4	E1BER5	ENSBTAG00000010030	0.211132305	0.101061528	0.110070778
B0JYR3	WASL	B0JYR3		0.231815675	0.178234635	0.05358104
A0AAF6Z7G4	RNASEH2B	A0AAF6Z7G4	ENSBTAG00000020149	-0.056344592	-0.076675591	0.020330998
A7MAZ3	UBA5	A7MAZ3; bta:509292		-0.034114576	0.013979712	-0.048094288
A0A3Q1LSY0	PAK3	A0A3Q1LSY0; bta:534526	ENSBTAG00000015670	0.180757933	0.238091108	-0.057333175
F6R4P6	SERPIND1	F6R4P6	ENSBTAG00000013973	0.001634782	0.387792663	-0.386157881
A7E3V0	SQSTM1	A7E3V0		0.004464248	0.233421605	-0.228957357
A0AAA9SXN7	TLK2	A0AAA9SXN7	ENSBTAG00000015713	0.106695465	0.061720937	0.044974528
A0AAA9T1A8	LUM	A0AAA9T1A8	ENSBTAG00000001745	0.129800328	0.27655273	-0.146752402
Q3SX39	MNAT1	bta:534176; F1MPA0; Q3SX39	ENSBTAG00000030801	0.076505986	0.198067966	-0.12156198
A3KN01	ZNF830	A3KN01; bta:539497	ENSBTAG00000020340	0.10514815	-0.016086984	0.121235135
G3X7E1	SAP18	bta:615692; G3X7E1	ENSBTAG00000018631	-0.067041653	-0.043299564	-0.023742089
E1BPL3	ABCB7	E1BPL3	ENSBTAG00000011028	-0.145506013	-0.248395187	0.102889174
F1MYF5	HS2ST1	F1MYF5	ENSBTAG00000017595	0.14637541	0.115600657	0.030774753
A0AAA9TWT4	DYNC2H1	A0AAA9TWT4	ENSBTAG00000019309	-0.15215447	-0.07694332	-0.07521115
G3N0U8	ERLIN1	bta:617074; G3N0U8	ENSBTAG00000007588	-0.017391603	-0.02536672	0.007975117
A0A3Q1MEL1	RHOT1	A0A3Q1MEL1	ENSBTAG00000010001	-0.137126233	-0.033079113	-0.10404712
Q0VD11	KIAA1715	bta:533298; Q0VD11		0.218808084	0.176671215	0.042136869
A0AAF6DM91	TRIM3	A0AAF6DM91	ENSBTAG00000003565	0.08435693	0.321307447	-0.236950517
Q5J801				0.015580011	0.537726404	-0.522146393
A2VDQ5	NLN	A2VDQ5; bta:538650		-0.54300331	-0.206263745	-0.336739565
A0A3Q1MUB3	ARHGAP31	A0A3Q1MUB3; bta:515490	ENSBTAG00000000210	-0.130087519	-0.086926756	-0.043160763
Q2KIN5	HMBS	bta:515614; Q2KIN5	ENSBTAG00000005364	0.190983944	0.134633089	0.056350854
A0A3Q1LXT2	CFAP36	A0A3Q1LXT2	ENSBTAG00000000542	-0.257445408	0.273069309	-0.530514717
A0AAA9TAG9	TXLNG	A0AAA9TAG9; bta:515927	ENSBTAG00000001768	0.032322422	-0.079406434	0.111728856
A0AAF6YUX3	GGCT	A0AAF6YUX3	ENSBTAG00000010597	0.092164923	0.107670344	-0.015505421
A0A3Q1N3F3	PPIH	A0A3Q1N3F3	ENSBTAG00000004590	-0.06871275	-0.04806844	-0.02064431
F1MCK7	WDR43	bta:538915; F1MCK7	ENSBTAG00000018196	-0.064130337	-0.007669848	-0.056460489
A7MB77	RBM17	A7MB77; bta:512694	ENSBTAG00000021951	-0.171625691	-0.253547415	0.081921724
F6QCN2	PSME3IP1	F6QCN2	ENSBTAG00000002242	-0.0588321	-0.274312474	0.215480374
P82925	MRPS31	bta:534185; P82925; Q148G3	ENSBTAG00000015522	-0.271736358	-0.217038323	-0.054698035
Q5E9K3	PNPO	bta:512573; Q5E9K3	ENSBTAG00000012259	-0.077188005	0.131324234	-0.208512239
F6RBM0	HSD17B12	F6RBM0	ENSBTAG00000000087	0.124223501	0.021226892	0.102996609
Q2YDN6	RPF2	bta:511294; Q2YDN6		0.08190656	0.035275475	0.046631085
A0AAA9SRY0	ATAD1	A0AAA9SRY0	ENSBTAG00000000806	0.050273206	-0.090480746	0.140753952
E1BMX2	TRMT2A	E1BMX2	ENSBTAG00000019871	-0.064742765	0.004035513	-0.068778278
F1MG80	NMI	F1MG80	ENSBTAG00000032369	-0.531535165	-0.236009069	-0.295526096
A6QLS4	NDE1	A6QLS4; bta:508088	ENSBTAG00000015986	-0.171025036	-0.259519922	0.088494886
A0AAF6DLS1	ARHGAP18	A0AAF6DLS1	ENSBTAG00000015381	0.032618843	-0.041185885	0.073804727
A0AAA9U0G2	ARPC4	A0AAA9U0G2	ENSBTAG00000007964	0.049172842	-0.06323312	0.112405962
A0AAA9TBR3	PAOX	A0AAA9TBR3	ENSBTAG00000018321	-0.304598216	-0.237545194	-0.067053022
F1MWB8	DMXL2	F1MWB8	ENSBTAG00000000737	-0.154159713	-0.162484577	0.008324865
A7Z062	ZC3H7A	A0A8J8XWW7; A7Z062; bta:540922; M5FKF8	ENSBTAG00000014826	-0.271939959	-0.12217187	-0.149768089
A5PJZ4	HECTD3	A5PJZ4; bta:513646		-0.122118082	-0.072631728	-0.049486353
A0A3Q1M861	EHBP1	A0A3Q1M861	ENSBTAG00000044173	-0.115010854	-0.07909128	-0.035919574
Q3T0V7	EDF1	bta:515380; Q3T0V7	ENSBTAG00000030302	0.300812078	0.095419565	0.205392513
A4FUE7	ZC2HC1A	A4FUE7; bta:516900		-0.005538184	0.00413973	-0.009677914
F6QEG6	RABGAP1L	F6QEG6	ENSBTAG00000049490	-0.25090065	-0.219349402	-0.031551248
Q2KIM0	FUCA1	bta:509522; Q2KIM0	ENSBTAG00000030434	0.240221614	0.474748679	-0.234527064
Q0V8A9	TIMM50	Q0V8A9		-0.064192379	-0.04858018	-0.015612199
G5E628	UBQLN2	G5E628	ENSBTAG00000021843	-0.001423478	0.219951312	-0.221374791
O97681	ATP6V0A2	bta:338038; O97681	ENSBTAG00000016523	0.106318037	0.776213805	-0.669895768
E1BEM1	EEF2K	bta:521730; E1BEM1	ENSBTAG00000017662	0.157921709	0.054141252	0.103780458
A0AAA9T257	ALG11	A0AAA9T257; bta:540845	ENSBTAG00000010337	0.1379421	0.130615039	0.00732706
P55052	FABP5	bta:281760; O62808; P55052; Q5E9D9	ENSBTAG00000047330	-0.142688417	0.509712882	-0.652401299
A0AAF6YH77	YKT6	A0AAF6YH77	ENSBTAG00000000274	-0.0088964	0.023458973	-0.032355373
A0AAA9SNV6	ZNF281	A0AAA9SNV6	ENSBTAG00000047428	-0.090551758	-0.17164025	0.081088492
A0AAA9S2F0	TXN	A0AAA9S2F0	ENSBTAG00000002953	0.109243847	0.054290156	0.054953691
F1N1L4	DPP8	bta:536604; F1N1L4	ENSBTAG00000014807	-0.416280666	-0.278777142	-0.137503524
A0A3Q1N960	SYTL4	A0A3Q1N960; bta:515978	ENSBTAG00000005724	-0.258262671	-0.402098444	0.143835773
A0AAA9RS96	UBXN7	A0AAA9RS96	ENSBTAG00000007577	0.144913669	0.05700658	0.087907089
F1MXY8	SLC25A1	F1MXY8	ENSBTAG00000008528	-0.009294892	0.031421098	-0.04071599
F6K7I9	CD46	F6K7I9		0.346845727	0.275270135	0.071575592
A0AAA9SD62	STAM	A0AAA9SD62	ENSBTAG00000002658	0.198481002	0.285247432	-0.086766429
A4FV68	PPP2R5E	A4FV68; bta:533617		0.170290102	0.155613525	0.014676577
A0AAA9TSI6	PACSIN3	A0AAA9TSI6	ENSBTAG00000009576	-0.067421748	-0.016889014	-0.050532734
A0AAA9S0T2	SERPINF2	A0AAA9S0T2	ENSBTAG00000020859	0.249135217	0.610053482	-0.360918264
A0A3Q1N325	PPP3CB	A0A3Q1N325	ENSBTAG00000019525	-0.117381137	0.619274832	-0.73665597
A5PK18	AGK	A5PK18; bta:514693		-0.24234589	-0.301541682	0.059195793
A0A3Q1MRT1	PRKAG1	A0A3Q1MRT1	ENSBTAG00000014426	-0.070845949	-0.01178786	-0.059058089
A0AAA9SLG2	CLTA	A0AAA9SLG2	ENSBTAG00000001137	0.123231766	0.058526077	0.064705689
Q9XT56	F11R	bta:281258; Q5E9V8; Q9XT56	ENSBTAG00000017846	0.15603056	0.08066856	0.075362
F1MM52	NTPCR	F1MM52	ENSBTAG00000013935	0.187022796	0.076861462	0.110161335
A0AAA9T259	PLEK2	A0AAA9T259	ENSBTAG00000016255	-0.296119772	-0.40275917	0.106639398
A0A3Q1MIS5	NBEA	A0A3Q1MIS5	ENSBTAG00000022991	-0.195757295	-0.037609461	-0.158147834
A0AAA9S2L1	XRCC1	A0AAA9S2L1; bta:616905	ENSBTAG00000016268	-0.505196688	-0.364452712	-0.140743976
A0A3Q1MPI8	ZNF800	A0A3Q1MPI8	ENSBTAG00000020004	0.382486927	0.18568622	0.196800707

Accession	Gene_Symbol	Gene_ID	Ensembl_Gene ID	log2(ratio(OV24H/OV0H))	log2(ratio(OV24R/OV0H))	log2(ratio(OV24H/OV24R))
A0A3Q1MWX6	RPRD1B	A0A3Q1MWX6	ENSBTAG00000005086	0.16567803	0.27822081	-0.112542779
Q3T112	PSMB8	bta:282013; Q32S32; Q3T112		0.086083874	0.162841073	-0.076757199
Q3SZ19	PSMD9	bta:513315; Q3SZ19	ENSBTAG00000004179	-0.057173708	-0.07570966	0.018535951
Q1W668	SMAD2	bta:516010; Q1W668		-0.049361927	-0.042591877	-0.00677005
A0A3Q1M6S1	RELCH	A0A3Q1M6S1; bta:504761	ENSBTAG00000016666	0.085528816	0.164938149	-0.079409333
F1MKD1	NTMT1	F1MKD1	ENSBTAG00000015437	-0.110764788	-0.080503573	-0.030261215
Q2KI45	TRMT10C	bta:532418; Q2KI45	ENSBTAG00000024027	-0.117356951	-0.099133192	-0.018223759
A0AAF6YUI9	CPSF2	A0AAF6YUI9	ENSBTAG00000010227	-0.147213649	-0.173838534	0.026624886
Q862I1	RPL24	bta:282048; P38663; Q3T157; Q862I1; Q862I5; Q862V7	ENSBTAG00000013461	-0.109336629	-0.107862234	-0.001474395
Q9TU47	EIF6	bta:286811; Q2TBM1; Q9TU47	ENSBTAG00000011263	0.255600554	0.408268944	-0.15266839
Q3ZBT6	TRA2B	bta:615156; Q3ZBT6	ENSBTAG00000001697	0.049170085	0.007794157	0.041375928
A0A3Q1MK32	UBE2L3	A0A3Q1MK32	ENSBTAG00000013038	-0.097870064	-0.13439594	0.036525876
Q59A28	CRYZL1	bta:506426; Q59A28		-0.555358788	-0.197008239	-0.358350548
Q0IIl9	ACTN3	bta:539375; Q0IIl9	ENSBTAG00000022244	-0.138916892	0.058823054	-0.197739945
A0A3Q1MG73	FBLN1	A0A3Q1MG73	ENSBTAG00000012088	0.231104503	0.580479757	-0.349375255
A0AAA9TJZ6	NUP35	A0AAA9TJZ6	ENSBTAG00000009777	-0.507165223	-0.474292268	-0.032872955
A0AAF6ZHP5	C4BPA	A0AAF6ZHP5	ENSBTAG00000009876	-0.222138269	-0.088034847	-0.134103422
Q0VBZ5	JUNB	bta:514246; Q0VBZ5	ENSBTAG00000012046	-0.230483834	-0.536675482	0.306191648
A0AAF6Z7M5	CSNK1D	A0AAF6Z7M5	ENSBTAG00000019986	-0.242360838	-0.394008544	0.151647706
A0AAF6Z8P4	SNAP29	A0AAF6Z8P4	ENSBTAG00000022760	0.190084838	0.068508778	0.12157606
F1MD13				-0.20614092	-0.080093591	-0.126047329
F6QIZ6	TUBGCP4	F6QIZ6	ENSBTAG00000014479	0.036606471	0.124993465	-0.088386994
A0AAA9S8Y0	TPD52L2	A0AAA9S8Y0	ENSBTAG00000021232	0.023594384	0.01945861	0.004135775
A0AAA9T383	CWF19L2	A0AAA9T383	ENSBTAG00000018629	0.176233903	0.046117467	0.130116436
A0AAA9SUC5	KYAT3	A0AAA9SUC5	ENSBTAG00000000505	-0.338839233	-0.26684602	-0.071993214
A7Z085	WDR82	A7Z085; bta:783336	ENSBTAG00000002960	-0.043677252	-0.122665548	0.078988296
A0AAA9T2E9	PHF3	A0AAA9T2E9	ENSBTAG00000019139	-0.179239678	-0.126830022	-0.052409655
A0A3Q1LRL0	NACC1	A0A3Q1LRL0	ENSBTAG00000016350	-0.223774975	-0.185555653	-0.038219322
F1MYE0	IRS1	bta:538598; F1MYE0	ENSBTAG00000021308	-0.115731146	-0.213028347	0.097297201
A0AAA9RVY9	DBT	A0AAA9RVY9	ENSBTAG00000006320	0.210012947	0.376120195	-0.166107248
F1ML90	PARP14	F1ML90	ENSBTAG00000016656	-0.219180116	-0.135278859	-0.083901258
A0AAF6Z8C2	ACP1	A0AAF6Z8C2	ENSBTAG00000020498	0.103776818	0.058270605	0.045506214
Q32PJ6	CIAO1	bta:540069; Q32PJ6	ENSBTAG00000015659	-0.147341716	-0.112096218	-0.035245498
F1MM82	SLC39A10	A0A3Q1N2V0; F1MM82	ENSBTAG00000016782	0.122484007	-0.195256291	0.317740298
Q0P592	FN3KRP	bta:615868; F1MII4; Q0P592	ENSBTAG00000015390	0.070596033	0.2504386	-0.179842568
Q3SZA5	SMS	A1L596; bta:615950; Q3SZA5	ENSBTAG00000018382	0.064428717	0.04193783	0.022490887
A0A3Q1MEN6	CHTOP	A0A3Q1MEN6	ENSBTAG00000005166	0.090291924	-0.009347949	0.099639873
G3MX46	CIC	bta:538483; G3MX46	ENSBTAG00000019785	-0.015170435	-0.060088884	0.04491845
F1MPG0	MLH1	bta:533652; F1MPG0	ENSBTAG00000016758	-0.284557401	-0.134531957	-0.150025444
F1N099	BLTP3B	F1N099	ENSBTAG00000006040	-0.307137689	-0.230819781	-0.076317908
Q3ZBY2	TAGLN3	bta:515562; Q3ZBY2		0.19295597	0.233718839	-0.040762869
E1BK69	UFSP2	bta:617788; E1BK69	ENSBTAG00000018454	0.010947332	-0.031743746	0.042691078
A0AAA9RZX6	SMARCD2	A0AAA9RZX6	ENSBTAG00000021062	-0.156531835	-0.133308878	-0.023222957
F1N3H0	DCAF8	bta:510316; F1N3H0	ENSBTAG00000005796	-0.174915304	-0.306731258	0.131815954
E1BML6	SLC2A13	bta:613556; E1BML6	ENSBTAG00000043962	-0.042114874	0.092841021	-0.134955895
A0AAA9S547	CD58	A0AAA9S547	ENSBTAG00000040131	-0.196084162	-0.196084162	0
A0A3Q1N5D7	GOLM2	A0A3Q1N5D7	ENSBTAG00000012545	0.14292574	-0.102587706	0.245513445
F1MM35	VPS13A	F1MM35	ENSBTAG00000017734	-0.099431692	0.025969212	-0.125400904
Q2KI74	TRADD	bta:504707; Q2KI74	ENSBTAG00000012642	-0.048216069	0.027046876	-0.075262944
Q5BIN2	SMARCB1	A2VE50; bta:537412; Q5BIN2	ENSBTAG00000009988	-0.019515013	-0.107767483	0.08825247
A0A3Q1M2B6	EGLN1	A0A3Q1M2B6; bta:534075	ENSBTAG00000053296	0.031554734	-0.00532688	0.036881614
A0A3Q1MIB8	CASP2	A0A3Q1MIB8; bta:531419	ENSBTAG00000018159	-0.231930551	-0.115825064	-0.116105487
Q08DC2	RAB31	bta:616979; Q08DC2	ENSBTAG00000046533	-0.076142855	-0.043721377	-0.032421478
Q17QI5	VAMP7	bta:613984; Q17QI5		0.107271322	0.267735995	-0.160464672
A7YY77	ENDOD1	A7YY77; bta:538618; F1MN88	ENSBTAG00000024803	0.031922405	0.121079924	-0.089157519
Q1JQA2	SMAD1	bta:540488; Q06AL6; Q1JQA2	ENSBTAG00000002835	-0.143222208	-0.152003093	0.008780886
Q3SZV7	HPX	bta:534509; Q3SZV7		-0.132022209	0.31836148	-0.450383689
Q3T0Q3	POLR2C	A7E3Q9; bta:504452; Q3T0Q3	ENSBTAG00000001856	-0.149769572	0.086448169	-0.236217741
A0AAA9RS89	RRP8	A0AAA9RS89	ENSBTAG00000000697	0.010935478	-0.001569	0.012504477
A0AAA9T4S4	CHD2	A0AAA9T4S4	ENSBTAG00000017776	-0.012161885	0	-0.012161885
E1BGX4	WBP2	bta:507060; E1BGX4	ENSBTAG00000008634	0.12619276	0.201357509	-0.075164749
A0A3Q1N178	TPP1	A0A3Q1N178	ENSBTAG00000015403	0.132395629	0.34847962	-0.216083991
F1N532	EMG1	F1N532	ENSBTAG00000013126	-0.106117032	-0.007432758	-0.098684275
A0A3Q1MSA9	MTCL1	A0A3Q1MSA9	ENSBTAG00000009459	-0.02200391	-0.093206461	0.071202551
A0A3Q1LMP2	KRAS	A0A3Q1LMP2; bta:541140	ENSBTAG00000009778	0.225991064	0.074946094	0.15104497
A0AAA9SVU4	SIRT5	A0AAA9SVU4	ENSBTAG00000014904	-0.079647145	0.045759661	-0.125406806
A0AAF6ZPG9	GUK1	A0AAF6ZPG9	ENSBTAG00000014775	0.08082248	0.171711621	-0.090889141
A0A452DIR7	BPNT2	A0A452DIR7	ENSBTAG00000015637	-0.376398472	-0.575594318	0.199195846
A0AAA9TSN5	NHLRC2	A0AAA9TSN5	ENSBTAG00000003690	-0.040601133	0.076571976	-0.117173109
Q0VD30	CCZ1	bta:511088; Q0VD30	ENSBTAG00000003288	-0.125412195	-0.006542846	-0.11886935
F1MPL1	MFN1	bta:515180; F1MPL1	ENSBTAG00000010485	-0.218566537	-0.158477653	-0.060088884
Q0P5N1	CNPY3	bta:510220; Q0P5N1	ENSBTAG00000015900	0.287783996	0.200585554	0.087198442
A0AAA9S8X1	RCOR1	A0AAA9S8X1	ENSBTAG00000002336	-0.185581919	-0.233598746	0.048016827
A0A3Q1MAB7	DCUN1D1	A0A3Q1MAB7	ENSBTAG00000012797	0.037067327	0.017226414	0.019840912
A0AAA9TL37	NUF2	A0AAA9TL37	ENSBTAG00000007247	-0.007463519	-0.309328058	0.301864539
F1MSS2	DDHD2	bta:513116; F1MSS2	ENSBTAG00000013145	0.108198741	-0.054966459	0.1631652
A0AAA9SQY1	MIPEP	A0AAA9SQY1	ENSBTAG00000008888	-0.081339627	-0.084227907	0.002888279
F1MM20	CCDC86	F1MM20	ENSBTAG00000014215	0.180202015	-0.038261558	0.218463574
A6QLC7	TRAPPC11	A6QLC7; bta:537211		-0.107528464	-0.040414268	-0.067114196
P79132	CAV1	A4D7R5; bta:281040; P79132; Q5PX19		0.103835811	0.040232678	0.063603133
F1MXV4	SIPA1L1	bta:787248; F1MXV4	ENSBTAG00000015805	-0.04338271	0.045884628	-0.089267338
Q3ZCK2	RALA	bta:538477; Q3ZCK2	ENSBTAG00000006661	0.010726407	-0.020134557	0.030860964
F1N388	AKAP11	F1N388	ENSBTAG00000014752	0.242897244	-0.100258648	0.343155892
A0AAA9SB14	NUP58	A0AAA9SB14	ENSBTAG00000015225	0.281606512	0.244793846	0.036812666
A0A3Q1N213	NLE1	A0A3Q1N213	ENSBTAG00000019094	-0.113150586	-0.138827705	0.025677119
A0AAF6YR37	MAPRE2	A0AAF6YR37	ENSBTAG00000007520	-0.107432429	-0.192453013	0.085020584
A5D7A3	MKLN1	A5D7A3; bta:508844; F1MMV9		0.067263227	-0.009870244	0.077133471
E1BAI0	RAB23	bta:618588; E1BAI0	ENSBTAG00000005362	-0.154849681	0.106434033	-0.261283714
A0AAF6ZBG6				0.068947354	0.176156955	-0.107209601
A0A3Q1MJQ1				-0.209368782	-0.037011784	-0.172356998
F1MJI8	PHKA2	F1MJI8	ENSBTAG00000015355	-0.247715201	-0.238333965	-0.009381236
A0AAA9T1Y2	IGSF23	A0AAA9T1Y2	ENSBTAG00000048075	0.029264595	0.044632996	-0.015368401
F1MEP0	MRPL45	F1MEP0	ENSBTAG00000025001	-0.204183623	-0.218152807	0.013969184
Q2KIE4	MCTS1	bta:508412; Q2KIE4		0.021673343	0.147921092	-0.126247749
G1K130	GPATCH1	G1K130	ENSBTAG00000000721	-0.234070589	0.125866354	-0.359936943
F6R3N4	KRT10	F6R3N4	ENSBTAG00000020824	-0.127718441	1.413533909	-1.54125235
B0JYL0	SMAD4	B0JYL0	ENSBTAG00000006919	0	-0.100453321	0.100453321
A2VE85	PRRX1	A2VE85; bta:540901	ENSBTAG00000004570	-0.255545427	0.096791886	-0.352337313
A0AAA9S9F0	BROX	A0AAA9S9F0	ENSBTAG00000007595	0.02173593	0.060316519	-0.03858059
A0AAF6YMX3	CDK9	A0AAF6YMX3	ENSBTAG00000004695	-0.115240574	-0.095383156	-0.019857418
A0AAA9SH73	SNX4	A0AAA9SH73	ENSBTAG00000011383	0.153039566	0.183357708	-0.030318143
Q3ZBG5	BAG2	bta:506107; Q3ZBG5		0.587426541	0.543686225	0.043740316
A0AAA9S9E2	PHACTR4	A0AAA9S9E2	ENSBTAG00000002727	0.132806737	0.241248549	-0.108441812
F6QYT8	AP1G2	F6QYT8	ENSBTAG00000007099	-0.285328674	-0.144699614	-0.14062906
Q08D87	FARS2	bta:505390; Q08D87	ENSBTAG00000018072	-0.322875885	-0.29936594	-0.023509945
F1MNQ8	STX2	F1MNQ8	ENSBTAG00000007988	-0.127379306	-0.14974712	0.022367813
P21839	BCKDHB	bta:282150; P21839; Q148F4; Q28047	ENSBTAG00000012096	-0.387992676	-0.228917131	-0.159075545
A0AAA9RYD0	QRSL1	A0AAA9RYD0	ENSBTAG00000017557	-0.102454065	0.041775322	-0.144229387
Q5EA68	SEC61A1	A9JSM4; bta:505064; Q5EA68	ENSBTAG00000004937	-0.074149767	-0.189005481	0.114855714
F6RN30	GGA1	F6RN30	ENSBTAG00000006464	-0.048680365	-0.010676793	-0.038003572
A0AAA9U2M1	ATE1	A0AAA9U2M1	ENSBTAG00000003178	0.073026471	0.122031014	-0.049004543
A0A3Q1MC43	ARID4B	A0A3Q1MC43; bta:534612	ENSBTAG00000000222	-0.105310843	-0.22703729	0.121726446
F1N4M3	PDPR	F1N4M3	ENSBTAG00000016471	-0.135514971	0.029544276	-0.165059246
E1BEY9	LSM14B	E1BEY9	ENSBTAG00000021558	0.196169101	0.179649511	0.016519589
A0AAA9S307	STIM2	A0AAA9S307	ENSBTAG00000019353	0.038665109	0.10629304	-0.06762793
H7BWW0	CORO1B	H7BWW0	ENSBTAG00000004783	0.088762149	0.10646415	-0.017702002
A0AAA9T947	ATP11A	A0AAA9T947	ENSBTAG00000000446	-0.138220035	-0.042157313	-0.096062722
A0AAA9TM73	ALG9	A0AAA9TM73	ENSBTAG00000020279	0.071083098	0.129532813	-0.058449715
F6RD06	FKBP7	F6RD06	ENSBTAG00000001097	-0.131322162	-0.1859073	0.054585138
E1BA21	TFAM	E1BA21	ENSBTAG00000003546	0.162967451	0.177506873	-0.014539423
E1BC89	OSBPL5	E1BC89	ENSBTAG00000010777	-0.207904658	-0.21063445	-0.002729793
Q3SYZ2	MAPKAPK3	bta:615215; Q0V8M1; Q3SYZ2	ENSBTAG00000016532	-0.242147039	-0.030373649	-0.21177339
Q05589	PLAU	A7E3W6; bta:281408; Q05589; Q0IIG3; Q28209	ENSBTAG00000005947	-0.510484285	-0.403407062	-0.107077223
A6QLY8	IGFBP7	A6QLY8; bta:616368		-0.108357178	-0.304303619	0.195946441
A0AAF6YT43	MX1	A0AAF6YT43	ENSBTAG00000030913	-0.255823491	-0.44530129	0.189477799
A0AAA9SEM5	MRPL40	A0AAA9SEM5	ENSBTAG00000019891	0.091046938	0.173767068	-0.08272013
Q3SZF8	SNRPD2	bta:514932; Q3SZF8	ENSBTAG00000012177	-0.091960304	-0.039328802	-0.052631502
A0A3Q1MJR9	VPS52	A0A3Q1MJR9	ENSBTAG00000002643	-0.159027009	0.409557476	-0.568584485
Q09430	PFN2	A4FUW7; bta:539034; Q09430		0.482352838	0.362570079	0.119782758
E1BPP2	BMS1	E1BPP2	ENSBTAG00000012878	-0.070309535	0.117930364	-0.188239899
Q2KID0	EXOSC2	bta:615712; Q2KID0	ENSBTAG00000048071	0.098593454	0.124745742	-0.026152288
F1MBS9	CDC40	F1MBS9	ENSBTAG00000000409	0.053439259	0.126947929	-0.07350867
Q861S4	RPL21	bta:326584; Q861S4	ENSBTAG00000001648	-0.062092771	-0.130142451	0.068049679
A0AAA9T6Q2	UBE2I	A0AAA9T6Q2	ENSBTAG00000038866	-0.042789273	-0.088286532	0.045497259
A0AAA9SQL2	TBC1D10B	A0AAA9SQL2	ENSBTAG00000031852	0.151213657	0.099114695	0.052098962
A0AAA9TZ45	PLAT	A0AAA9TZ45	ENSBTAG00000011244	0.032697038	0.09723729	-0.064540252
E1B8G9	H2BC26	bta:520120; E1B8G9	ENSBTAG00000058723	-0.111143917	-0.007054758	-0.10408916
F2Z4I1	RHEB	F2Z4I1	ENSBTAG00000031861	-0.124837112	-0.216467587	0.091630475
A0AAF6Z191	GLE1	A0AAF6Z191	ENSBTAG00000030566	-0.296617006	-0.275858446	-0.02075856
E1BH45	RB1CC1	bta:539858; E1BH45	ENSBTAG00000000878	-0.09676869	-0.029719488	-0.067049202
Q7SIB2	COL4A1	G1K238; Q7SIB2	ENSBTAG00000012849	-0.305298095	-0.479596228	0.174298133
F1N1E3	MMP14	F1N1E3	ENSBTAG00000014824	-0.354052298	-0.200139614	-0.153912683
E1BK11	ANAPC5	bta:540537; E1BK11	ENSBTAG00000003092	-0.190382026	-0.091348947	-0.09903308
A0AAA9TT40	ZC3H13	A0AAA9TT40	ENSBTAG00000007067	0.16550633	0.221919639	-0.056413309
A3KMX8	ARMT1	A3KMX8; bta:540698		0.29476178	0.293372569	0.001389211
F1N0R7	FLRT3	bta:529416; F1N0R7	ENSBTAG00000003319	-0.077823418	-0.136858888	0.05903547
A0A3Q1MR39	FLOT2	A0A3Q1MR39	ENSBTAG00000010073	0.139485054	0.36792652	-0.228441466
A0AAA9TWP9	NT5C3A	A0AAA9TWP9	ENSBTAG00000016709	0.287802311	0.309382842	-0.021580531
E1BAL6	EIF2AK4	bta:513829; E1BAL6	ENSBTAG00000014351	0.027647045	-0.025194082	0.052841127
G3MWN1	ARG2	G3MWN1	ENSBTAG00000001945	-0.088791515	-0.241719964	0.152928449
A0AAA9TZ73	DBI	A0AAA9TZ73	ENSBTAG00000009517	0.26124335	0.483635772	-0.222392421
A0AAA9SJL3	SPG7	A0AAA9SJL3	ENSBTAG00000012041	-0.116863758	-0.019533887	-0.097329871
A0AAA9S2Z7	FABP3	A0AAA9S2Z7	ENSBTAG00000016819	0.105460508	0.133565845	-0.028105338
E1BGD1	TOMM34	E1BGD1	ENSBTAG00000003256	-0.026883294	0.121950532	-0.148833826
F6RU89	RPA2	F6RU89	ENSBTAG00000006225	0.132944341	0.128656983	0.004287358
F1N795	RASA2	F1N795	ENSBTAG00000015413	0.130990817	0.124369865	0.012707833
F1MY63	TYMS	F1MY63	ENSBTAG00000007003	-0.788787162	-0.514115586	-0.274671576
A0A3Q1M488	MRPL39	A0A3Q1M488	ENSBTAG00000019542	-0.274876018	-0.274876018	0
Q3B7M6	NEDD1	bta:519463; Q3B7M6	ENSBTAG00000001894	0.026704324	0.055655698	-0.028951374
A5D7E0	RAB35	A5D7E0; bta:614521; F1MSJ9		0.15759209	0.254659509	-0.097067418
G3X687	TUBGCP6	bta:618908; G3X687	ENSBTAG00000000650	-0.066482902	0.101126004	-0.167608907
F6QYF9	DDX41	F6QYF9	ENSBTAG00000015187	-0.133819149	-0.265407911	0.131588762
Q2YDM1	ARL1	bta:517345; Q2YDM1	ENSBTAG00000011883	-0.082251594	-0.196571246	0.114319652
A0AAA9TQT5	WDR47	A0AAA9TQT5	ENSBTAG00000003384	0.065140605	0.03822945	0.026911154
F1N3R6	EPHB2	F1N3R6	ENSBTAG00000045902	-0.199897318	0.079203953	-0.279101271
A0A3Q1LM49	PPM1A	A0A3Q1LM49	ENSBTAG00000018127	-0.070320695	-0.03613876	-0.034181936
A5D7C1	DDX52	A5D7C1; bta:510826	ENSBTAG00000010313	0.15417093	0.204797003	-0.050626073
A0A3Q1MXM3	ARID1B	A0A3Q1MXM3	ENSBTAG00000002728	-0.222817306	-0.168810275	-0.054007031
A0A3Q1M1E6	DHRS7B	A0A3Q1M1E6	ENSBTAG00000010559	0.016488123	0.119992699	-0.103504576
A0A3Q1M3Q2	PARN	A0A3Q1M3Q2	ENSBTAG00000018097	-0.250774223	-0.125774231	-0.124999992
A0A173FDG1	BoLA	A0A173FDG1		-0.248253362	-0.204046886	-0.044206476
A0A3Q1MEJ9	MBNL1	A0A3Q1MEJ9	ENSBTAG00000004564	0.077346369	0.189525736	-0.112179367
Q3SZ32	TCEB2	A0A8J8XQC3; bta:617277; F1N668; M5FK31; Q3SZ32		0.13936707	-0.33912371	0.47849078
E1BDC9	VPS52	E1BDC9	ENSBTAG00000002643	0.057278971	0.113656782	-0.056377811
F1MM23	CDK5RAP3	F1MM23	ENSBTAG00000014202	0.232349268	0.299966847	-0.067617579
A5PK88	PITHD1	A5PK88; bta:506494	ENSBTAG00000021620	0.347305367	0.296096427	0.051208941
F6Q3P6	MINK1	F6Q3P6	ENSBTAG00000004907	-0.172384002	-0.053389813	-0.118994189
F1MXJ5	IST1	F1MXJ5	ENSBTAG00000019883	0.211437325	0.029087579	0.182349746
E1BJ09	MIB1	bta:533735; E1BJ09	ENSBTAG00000007467	0.029503994	-0.004478105	0.033982099
Q1LZE1	KCMF1	bta:613522; Q1LZE1		0.130517093	0.134332067	-0.003814974
E1BEN4	GMPPA	bta:504889; E1BEN4	ENSBTAG00000002995	-0.303972518	-0.24138004	-0.062592478
Q1RMT1	ERCC3	bta:507984; Q1RMT1	ENSBTAG00000020777	-0.028105338	0.070904668	-0.099010006
Q3SZ29	POLR1E	bta:511587; F1MXX3; Q3SZ29	ENSBTAG00000012243	-0.026652392	0.1428006	-0.169452993
A0A3Q1NE17	DLGAP4	A0A3Q1NE17	ENSBTAG00000001741	0.004195917	-0.057143907	0.061339824
A0A3Q1MSC8	SRRM1	A0A3Q1MSC8	ENSBTAG00000013772	-0.009337865	-0.096215315	0.086877451
F6RWP3	PAIP1	F6RWP3	ENSBTAG00000020376	0.084603187	0.187278568	-0.102675381
A0AAA9SXI3	TBC1D13	A0AAA9SXI3	ENSBTAG00000012480	0.019686203	0.056910213	-0.037224011
Q3ZCC8	TPPP3	bta:614988; Q3ZCC8	ENSBTAG00000019822	0.028512688	0.086087579	-0.057600267
A7MB47	CNOT9	A7MB47; bta:536537	ENSBTAG00000003807	0.09133113	0.077214628	0.014116502

Accession	Gene_Symbol	Gene_ID	Ensembl_Gene ID	log2(ratio(OV24H/OV0H))	log2(ratio(OV24R/OV0H))	log2(ratio(OV24H/OV24R))
A0AAA9SZ09	ILKAP	A0AAA9SZ09	ENSBTAG00000010176	0.168357706	0.123048545	0.045309162
Q32L30	TEX264	bta:535455; Q32L30	ENSBTAG00000034796	0.001370732	-0.015164636	0.016535368
A8E4N5	RING1	A8E4N5; bta:100125778; F1MUV0	ENSBTAG00000018884	0.032388392	0	0.032388392
A0A3Q1M5N1	ZMYM2	A0A3Q1M5N1	ENSBTAG00000020403	-0.094503986	-0.086156644	-0.008347343
A0AAA9SGY7	PSMG2	A0AAA9SGY7	ENSBTAG00000010552	0.194676133	0.113656782	0.081019351
Q32PA4	PHPT1	bta:618691; Q32PA4	ENSBTAG00000060065	0.07933896	0.119277705	-0.039938745
A0AAF6YM21	NDUFA8	A0AAF6YM21	ENSBTAG00000004295	-0.178500896	-0.173232375	-0.005268521
A0AAA9TF84	PACS1	A0AAA9TF84	ENSBTAG00000009026	-0.099966908	0.057685907	-0.157652816
A0AAA9TG44	NMD3	A0AAA9TG44	ENSBTAG00000016228	-0.097534331	-0.033522573	-0.064011758
E1BIV7	ZFC3H1	bta:781886; E1BIV7	ENSBTAG00000012267	-0.028450539	-0.15811167	0.129661131
E1BJH5	MAP4K3	E1BJH5	ENSBTAG00000016442	-0.209603905	-0.144263151	-0.065340754
A0AAA9RTS3	BOP1	A0AAA9RTS3	ENSBTAG00000009811	-0.225515399	0.08932493	-0.314840329
A0AAA9T7W8	FMN1	A0AAA9T7W8	ENSBTAG00000025634	-0.010404096	0.082270172	-0.092674268
A0AAA9T9D3	CHAMP1	A0AAA9T9D3	ENSBTAG00000020861	-0.139930261	-0.11345805	-0.026472211
A0A3Q1LQK8	OSBPL6	A0A3Q1LQK8	ENSBTAG00000014405	-0.28326889	-0.302964691	0.019695801
G1K122	RBP4	bta:281444; G1K122	ENSBTAG00000000442	-0.212449618	0.013900817	-0.226350435
A5PJM4	AIFM2	A5PJM4; bta:534217	ENSBTAG00000001165	0.163401421	0.325465781	-0.162064361
F1MK36	DDRGK1	F1MK36	ENSBTAG00000013753	0.12400664	0.150326338	-0.026319699
F1MRE2	TUFT1	F1MRE2	ENSBTAG00000017566	0.101702208	0.059882032	0.041820176
Q645M6	FADD	bta:493720; Q645M6	ENSBTAG00000018274	0.184862792	0.258461676	-0.073598884
Q3MHN8	TRMT5	bta:528221; Q3MHN8	ENSBTAG00000011796	-0.168301259	-0.05294888	-0.115352379
Q3SWY6	STK25	bta:373543; Q3SWY6; Q6V9V8	ENSBTAG00000009676	-0.515819024	-0.468312292	-0.047506731
Q56JX8	RPS13	bta:535668; Q56JX8	ENSBTAG00000040308	-0.120604791	-0.134649527	0.014044735
A0A3Q1MI24	TIPRL	A0A3Q1MI24	ENSBTAG00000005393	0.004473477	0.065687903	-0.061214426
F6RNG7	FAM20B	F6RNG7	ENSBTAG00000013999	0.096359242	-0.063859942	0.160219184
Q2KI55	RBPMS	bta:614417; Q2KI55		0.061329989	0.064572001	-0.003242013
E1B9D9	TRAF2	E1B9D9	ENSBTAG00000010497	-0.740945445	-0.55955292	-0.181392525
A0AAA9SEL1	MED23	A0AAA9SEL1	ENSBTAG00000012418	-0.017198769	-0.061800856	0.044602087
Q0VFX8	CRIP2	bta:780821; Q0VFX8		0.014161537	0.012398929	0.001762609
A0A452DJ34	RPL28	A0A452DJ34	ENSBTAG00000023343	-0.085067715	-0.113511693	0.028443978
A0AAA9RXH4	KRR1	A0AAA9RXH4	ENSBTAG00000011591	-0.18833412	0.001589747	-0.189923867
A0AAA9S719	NUP43	A0AAA9S719	ENSBTAG00000010196	0.125662701	0.130005991	-0.00434329
F6QYN9	SRSF4	F6QYN9	ENSBTAG00000013309	0.394476177	0.307703455	0.086772722
A0AAA9SLP6	AAMP	A0AAA9SLP6	ENSBTAG00000003927	-0.223125684	-0.032621422	-0.190504263
A0AAF6YK23	PTMA	A0AAF6YK23	ENSBTAG00000002549	0.779628051	-0.11454434	0.894172391
A0AAA9SLC8	MTMR1	A0AAA9SLC8	ENSBTAG00000009657	-0.036069255	-0.080891452	0.044822198
Q2TBN3	CETN2	bta:508601; Q2TBN3	ENSBTAG00000007844	0.317705276	-0.025830933	0.343536209
A0AAF6Z1W8	RPL18A	A0AAF6Z1W8	ENSBTAG00000015831	-0.220688115	-0.283125824	0.062437709
F2Z4H3	POLR2H	bta:505599; F2Z4H3	ENSBTAG00000040199	0.12333702	0.154769528	-0.031432508
Q17R09	DHX38	bta:520046; Q17R09	ENSBTAG00000013265	-0.747856846	-0.160943439	-0.586913407
E1B9A3	MB21D2	bta:519376; E1B9A3	ENSBTAG00000005430	0.089724119	0.065802058	0.023922061
Q0VC98	GABPA	bta:510204; Q0VC98	ENSBTAG00000019043	-0.026311277	-0.03222397	0.005912693
A0AAA9S345	UBE2N	A0AAA9S345	ENSBTAG00000021767	0.327374047	0.155048953	0.172325094
A0AAA9T3D4	ERGIC1	A0AAA9T3D4	ENSBTAG00000015955	0.004005634	0.033045307	-0.029039673
A0A3Q1MGT8	SGPL1	A0A3Q1MGT8	ENSBTAG00000009984	0.073573264	0.019802008	0.053771256
A0A3Q1MY43	DCAF5	A0A3Q1MY43	ENSBTAG00000048519	-0.02233895	1.136495586	-1.158834536
F1MBN7	RIOK2	bta:540772; F1MBN7	ENSBTAG00000011763	-0.887616583	-0.16320657	-0.724410013
A0A452DJ05	MARF1	A0A452DJ05	ENSBTAG00000020387	0.262269253	0.130113295	0.132155959
Q32L22	POLR1C	bta:516337; Q32L22	ENSBTAG00000018969	-0.126059826	-0.032355373	-0.093704453
A0AAF7ATF7	ASS1	A0AAF7ATF7	ENSBTAG00000020747	-0.009203827	0.135625694	-0.144829521
A0AAA9TLZ5	MACROH2A1	A0AAA9TLZ5	ENSBTAG00000016105	-0.269186633	0	-0.269186633
Q0VC02	AAAS	bta:506561; Q0VC02		0.107536475	0.104842366	0.002694109
A0AAA9SBD1	SSBP1	A0AAA9SBD1	ENSBTAG00000010931	0.223924758	0.202766615	0.021158143
Q3ZBT5	STX7	bta:507031; Q3ZBT5	ENSBTAG00000017139	0.227656585	0.190613243	0.037043342
A0AAA9SWW0	TSC1	A0AAA9SWW0	ENSBTAG00000005190; ENSBTAG00000066551	-0.093469673	0.042591877	-0.13606155
A0A3Q1MM07	GAB1	A0A3Q1MM07	ENSBTAG00000002813	0.058230228	0.275874752	-0.217644523
A0A3Q1NLW9	VPS37B	A0A3Q1NLW9; bta:535566	ENSBTAG00000048809	0.109112455	0.123313595	-0.01420114
A0AAA9SKW6	BRCA1	A0AAA9SKW6	ENSBTAG00000022520	-0.115597447	0.044044333	-0.15964178
E1B8Q4	CHTF18	A0A8J8Y4N9; E1B8Q4; M5FKI2	ENSBTAG00000019743	-0.009925214	-0.084375163	0.074449949
A4FV37	CAVIN3	A4FV37; bta:510203		-0.034498195	-0.077529131	0.043030937
E1BBC2	TMEM209	E1BBC2	ENSBTAG00000015588	0.065318338	0.071112302	-0.005793964
F6QQY9	SRI	F6QQY9	ENSBTAG00000010390	-0.030607986	-0.21164902	0.181041034
Q8HXY9	CFDP1	bta:281682; Q2KIY4; Q8HXY9; Q8I031; Q8I033		0.277196858	0.268517102	0.008679756
A0A3Q1M2S8	TUT4	A0A3Q1M2S8	ENSBTAG00000002721	-0.48334585	-0.223549818	-0.259796032
A6QQW4	GTSE1	A6QQW4; bta:507901		0.093642847	0.076204201	0.017438646
A6QLY4	ISOC1	A6QLY4; bta:540524		0.14338608	0.231625701	-0.088239621
A0AAF6ZXX8	CDC42	A0AAF6ZXX8	ENSBTAG00000001700	-0.096704083	0.032232507	-0.12893659
Q2KHU5	MACROD1	bta:613568; Q2KHU5	ENSBTAG00000017181	-0.126224986	0.018958158	-0.145183144
A0A3Q1LTG9	TBC1D9B	A0A3Q1LTG9	ENSBTAG00000015611	-0.154534693	-0.103198888	-0.051335806
E1BP11	RFX1	E1BP11; F1MGS1	ENSBTAG00000008854	-0.160077215	-0.161550105	0.001472889
A0AAA9SER2	HAUS8	A0AAA9SER2	ENSBTAG00000007366	-0.204798361	-0.246189993	0.041391631
Q0Z7W6	TMX1	A3KN45; A5D9C0; bta:509037; Q0Z7W6	ENSBTAG00000011225	0.110912699	0.125664311	-0.014751612
A0A3Q1M407	CEP131	A0A3Q1M407	ENSBTAG00000000875	-0.177419538	-0.146752402	-0.030667136
A0AAA9TEH8	NACA	A0AAA9TEH8	ENSBTAG00000010701	-0.048094288	-0.199418307	0.151324018
A0AAF6Z0Y9	RAD23A	A0AAF6Z0Y9	ENSBTAG00000015116	0.120840836	0.059501012	0.061339824
F1MCU6	QTRT1	F1MCU6	ENSBTAG00000037941	-0.535453401	-0.324028306	-0.211425095
A0AAF6DLY7	PAK4	A0AAF6DLY7	ENSBTAG00000013958	-0.54192759	-0.134861961	-0.407065629
A0AAA9T7Y9	CYB5R1	A0AAA9T7Y9	ENSBTAG00000009927	0.083779417	0.14551906	-0.061739643
P79251	ATP6V1G1	bta:281641; P79251; Q32KN3	ENSBTAG00000000203	0.028188544	-0.02611284	0.054301384
F1MBQ2	UBE2J1	bta:539754; F1MBQ2	ENSBTAG00000018436	-0.001581036	-0.053111336	0.051530301
A0A452DIQ1	NAPG	A0A452DIQ1	ENSBTAG00000005158	-0.179930579	-0.044694999	-0.135235581
A0AAA9SXE3	MAP3K6	A0AAA9SXE3	ENSBTAG00000010254	-0.459586822	-0.189456017	-0.270130805
Q3ZCL8	SH3BGRL3	bta:614672; Q3ZCL8	ENSBTAG00000009580	0.009210123	-0.18718709	0.196397213
F1N0K0	COL11A1	bta:287013; F1N0K0	ENSBTAG00000012217	-0.340326156	-0.214795273	-0.125530882
A0A3Q1MAA7	USP28	A0A3Q1MAA7	ENSBTAG00000002323	0.127874823	0.103518109	0.024356713
A0AAA9S5W8	RAB12	A0AAA9S5W8	ENSBTAG00000010356	-0.006789166	-0.099535674	0.092746508
A0AAA9TVP9	FAM91A1	A0AAA9TVP9; bta:507013	ENSBTAG00000005025	-0.31585358	-0.385712603	0.069859023
A0AAA9SXX9	WAC	A0AAA9SXX9	ENSBTAG00000018263	-0.083591812	-0.083591812	0
Q58DJ9	CHEK2	bta:518897; Q58DJ9		-0.061776198	0.072992358	-0.134768556
Q5EA77	SNX17	bta:529972; Q17QP3; Q5EA77		-0.236593935	-0.178993668	-0.057600267
E1BF38	DENND10	bta:534370; E1BF38	ENSBTAG00000008729	-0.075312852	-0.026549133	-0.048763719
A0A3Q1MQW8	RPS18	A0A3Q1MQW8	ENSBTAG00000002648	-0.006299988	-0.039833978	0.03353399
A0AAA9TAN7	ZFYVE1	A0AAA9TAN7	ENSBTAG00000011041	0.293563204	0.233746919	0.059816285
A5PK75	TOR1A	A5PK75; bta:533699	ENSBTAG00000020305	0.036051227	0.232785025	-0.196733798
F6PXF3	STAMBP	F6PXF3	ENSBTAG00000009600	0.136259165	0.210398062	-0.074138896
A0AAA9ST58	LAMC2	A0AAA9ST58; bta:511043	ENSBTAG00000000793	-0.316521759	-0.197751968	-0.11876979
A5PJG7	HCCS	A5PJG7; bta:506250	ENSBTAG00000012113	-0.309192412	-0.343618439	0.034426027
Q66LN0	PTGES2	bta:493639; F1N1J6; Q66LN0		-0.097197843	-0.109437111	0.012239268
A0AAA9TVC8	NDUFA7	A0AAA9TVC8	ENSBTAG00000052280	-0.003343443	0.074835233	-0.078178676
Q3T114	RIDA	bta:504390; Q3T114	ENSBTAG00000012595	0.29936594	0.10491501	0.19445093
Q3ZBW5	RHOB	bta:515118; Q3ZBW5	ENSBTAG00000006681	0.073872108	0.148787815	-0.074915707
A1L504	LMF2	A1L504; bta:510646; Q32PH4		-0.260598106	-0.051013166	-0.20958494
A0A0K1L4Y6	TRIM26	A0A0K1L4Y6		-0.104697379	-0.236352363	0.131654985
A0AAA9SKE5	SET	A0AAA9SKE5	ENSBTAG00000020959	0.111262857	0.188732152	-0.077469295
E1B8C2	NOM1	E1B8C2	ENSBTAG00000016435	0.15336509	0.213361055	-0.059995965
O18756	GLCE	bta:281195; O18756	ENSBTAG00000001405	-0.343809151	-0.222812114	-0.120997037
F1MKY0	GGA2	F1MKY0	ENSBTAG00000002352	-0.0959833	0.063193826	-0.159177126
Q1RMQ1	FRG1	bta:512533; Q1RMQ1	ENSBTAG00000007089	0.004983408	-0.026872862	0.031856269
A0A3Q1NM68	STRN4	A0A3Q1NM68	ENSBTAG00000005830	-0.389069815	-0.239642557	-0.149427257
A6QLC8	TRAPPC12	A6QLC8; bta:615707; F1MZZ3	ENSBTAG00000008685	0.076706173	0.101394606	-0.024688432
A0AAA9SDV9				0.05403984	0.02727293	0.02676691
G3MYQ9	ABCB8	G3MYQ9	ENSBTAG00000007759	-0.624947371	-0.511290589	-0.113656782
A0AAA9T614	APEH	A0AAA9T614	ENSBTAG00000011583	0.174664118	0.358913983	-0.184249865
A0AAA9TH62	MRPS7	A0AAA9TH62	ENSBTAG00000016133	-0.564516525	-0.308796581	-0.255719944
F1N0E6	VWA5A	F1N0E6	ENSBTAG00000021215	-0.117591338	-0.046844863	-0.070746475
F1MP72	NCOA7	F1MP72	ENSBTAG00000046503	0.024509694	0.199091322	-0.174581628
Q3SZI7	COG6	bta:509305; Q3SZI7		-0.040098069	-0.07979377	0.039695701
A0A3Q1MGD3	LRBA	A0A3Q1MGD3	ENSBTAG00000018546	-0.163975735	0.043068722	-0.207044457
F1N2G5	ARHGEF5	F1N2G5	ENSBTAG00000001815	0.046524281	-0.074546953	0.121071234
Q0VCT3	TERF2IP	bta:509625; Q0VCT3	ENSBTAG00000015686	0.157599872	0.215823093	-0.058223221
A0AAF6YTE4	MRPL50	A0AAF6YTE4	ENSBTAG00000008962	0.094734007	-0.122890746	0.217624753
A0AAA9T1I0	NCDN	A0AAA9T1I0	ENSBTAG00000016696	-0.039528364	0.074525375	-0.114053739
Q32L87	DCAF7	bta:516678; F1MVU0; Q32L87	ENSBTAG00000003500	0.088723902	0.001429119	0.087294783
Q148H9	RRP1	A0A3Q1N7Y1; bta:512925; Q148H9	ENSBTAG00000003981	-0.141111304	-0.200387113	0.059275809
E1BHL1				0.471337896	0.325456906	0.14588099
P22439	PDHX	bta:517402; P22439; Q0P576	ENSBTAG00000018261	-0.038316932	-0.039811178	0.001494247
P61356	RPL27	bta:404137; P61356; Q3T0P1	ENSBTAG00000017441	-0.081643238	-0.146443568	0.06480033
F1MHT2	PIP4K2B	bta:539211; F1MHT2	ENSBTAG00000006499	0.088205771	0.214429709	-0.126223938
A0AAA9SZ52	TIGAR	A0AAA9SZ52	ENSBTAG00000016650	0.254526043	0.12486797	0.129658073
A0A3Q1MRS1	EIF4E2	A0A3Q1MRS1	ENSBTAG00000054466	-0.150835628	-0.242681344	0.091845716
A0A452DHW2	NAXE	A0A452DHW2	ENSBTAG00000000404	0.053180038	0.141127737	-0.087947699
F1N0Y0	UPP1	F1N0Y0	ENSBTAG00000008428	0.139117377	0.096374467	0.04274291
Q2KII4	ELOC	bta:540859; Q2KII4	ENSBTAG00000050514	0.593051321	0.049546795	0.543504526
A0A452DJI7	FOXO1	A0A452DJI7	ENSBTAG00000044105	-0.090063698	0.017904798	-0.107968497
A0AAA9SUV1	MYBPC1	A0AAA9SUV1	ENSBTAG00000011392	-0.196800707	-0.096771889	-0.100028818
A0AAA9T2Y0	MVB12B	A0AAA9T2Y0	ENSBTAG00000050426	-0.022425763	0.095656904	-0.118082667
A0A3Q1NEF8	RAC1	A0A3Q1NEF8	ENSBTAG00000053390	-0.143482927	-0.094524505	-0.048958422
A0AAA9T5D9	UBE2V1	A0AAA9T5D9	ENSBTAG00000027316	-0.017720121	-0.087738051	0.07001793
A0A3Q1M8I7	BABAM2	A0A3Q1M8I7	ENSBTAG00000031335	-0.061902439	0.172487516	-0.234389954
E1BP38	RALGAPA1	E1BP38	ENSBTAG00000001282	0.156775718	-0.080755838	0.237531557
A0AAF6Z3Q7	METTL1	A0AAF6Z3Q7	ENSBTAG00000016908	0.064350411	0.274239861	-0.209889449
A0A452DIF2	CLTB	A0A452DIF2	ENSBTAG00000010740	0.064804652	0.21694257	-0.152137918
A0AAA9RW61	TRIM36	A0AAA9RW61	ENSBTAG00000014027	0.037760804	0.073043781	-0.035282977
P00423	COX4I1	bta:281090; P00423; Q3SZS0; Q5E991	ENSBTAG00000016079	-0.098607597	-0.158596996	0.059989399
Q2KHX8	RCL1	bta:768075; Q2KHX8		-0.115098408	-0.07783414	-0.037264268
Q3T0F9	GRB2	bta:535298; Q3T0F9		0.205955532	0.01036317	0.195592362
Q2KHW4	SLC25A15	bta:532560; Q2KHW4	ENSBTAG00000011647	0.046267604	0.088772332	-0.042504729
G3N166	PRPF38B	bta:100299693; G3N166	ENSBTAG00000047874	0.16089119	0.081921724	0.078969466
A0A3Q1N3W6	MTA3	A0A3Q1N3W6	ENSBTAG00000004073	0.156445087	0.137792381	0.018652707
A0A3Q1NJD5	BICC1	A0A3Q1NJD5	ENSBTAG00000010694	-0.036525876	-0.037802033	0.001276157
A0AAA9SBV1	IWS1	A0AAA9SBV1	ENSBTAG00000021172	0.066045335	0.057600267	0.008445068
Q5E9Q4	NECAP2	bta:509439; Q5E9Q4		-0.085427619	-0.049727049	-0.035700569
A0AAA9U1G3				-0.004473477	-0.0843025	0.079829024
A0A3Q1LX25	PLEKHG3	A0A3Q1LX25	ENSBTAG00000001551	-0.174964989	-0.167882525	-0.007082464
A0AAA9RZH3	NCOR1	A0AAA9RZH3	ENSBTAG00000013271	-0.347553903	-0.460447959	0.112894056
A0AAA9SQ70	GOSR1	A0AAA9SQ70	ENSBTAG00000004961	0.118532389	-0.052046748	0.170579137
G1K1P8				0.195825088	0.131635296	0.064189792
Q1RML0	PRCC	bta:507189; Q1RML0	ENSBTAG00000000608	0.01759406	0.027759039	-0.010164979
Q5BIN5	PIN1	bta:535470; Q5BIN5	ENSBTAG00000016988	-0.018992715	-0.030806047	0.011813331
F1MDM4	REEP5	F1MDM4	ENSBTAG00000012804	0.136538833	0.265683984	-0.129145151
A4FUI1	MIX23	A4FUI1; bta:508149	ENSBTAG00000063883	0.163670662	0.255257055	-0.091586393
A6QQV3	LSM2	A6QQV3; bta:538662		0.096479115	0.116231962	-0.019752847
F1N6Q4	UPRT	F1N6Q4	ENSBTAG00000004549	-0.041647697	0.099144646	-0.140792344
F6QAZ2	PCDHGB4	F6QAZ2	ENSBTAG00000017349	-0.229481846	0.013514806	-0.242996653
E1BEP7	ELP2	bta:511041; E1BEP7	ENSBTAG00000018954	-0.255448742	-0.320740203	0.06529146
Q3T045	SMNDC1	bta:520500; Q3T045		0.358995091	0.033435912	0.325559179
Q3SZE9	TBCC	bta:515354; Q3SZE9	ENSBTAG00000000940	0.035919574	-0.03994938	0.075868954
P79345	NPC2	bta:280815; P79345; Q3T091; Q58DR4		-0.078894047	-0.116910311	0.038016264
G5E5P6	AGPAT3	G5E5P6	ENSBTAG00000020456	-0.090748416	-0.103385241	0.012636826
F6RIB6	TMCC1	F6RIB6	ENSBTAG00000006614	0.110381371	0.295505867	-0.185124496
A0A3Q1MFB9	PLD2	A0A3Q1MFB9	ENSBTAG00000013392	-0.538446399	-0.265483615	-0.272962784
Q3ZCH9	HDHD2	bta:505403; Q3ZCH9	ENSBTAG00000011893	0.095157233	0.254813899	-0.159656666
F1MCN4	STARD13	F1MCN4	ENSBTAG00000021815	-0.143801984	-0.116292135	-0.027509849
Q3T0D5	RPL30	bta:513031; Q3T0D5	ENSBTAG00000016278	-0.046650093	-0.043943348	-0.002706746
F1N431	FDPS	F1N431	ENSBTAG00000003948	0.066795439	0.086740849	-0.01994541
F6R6Q1	FLAD1	bta:506682; F6R6Q1	ENSBTAG00000000186	-0.181661109	-0.122296138	-0.059364971
Q0VC37	DFFA	bta:507981; F1N6C7; Q0VC37	ENSBTAG00000013535	0.090982655	0.118090458	-0.027107802
A0AAA9SES4	AURKA	A0AAA9SES4	ENSBTAG00000013009	0.139622241	-0.180794387	0.320416628
G3MYZ3	AFM	bta:508264; G3MYZ3		-0.092502082	0.309114902	-0.401616984
F1MW60	PDXP	F1MW60	ENSBTAG00000030255	0.193990248	0.207373415	-0.013383167
E1BGZ1	DUS3L	E1BGZ1	ENSBTAG00000011842	-0.098283334	-0.04453116	-0.053752174
P23934	NDUFS6	bta:327691; P23934	ENSBTAG00000009914	-0.059321091	0.016993071	-0.076314162
A6QLV2	URB2	A6QLV2; bta:514623		-0.609540523	-0.439615522	-0.169925001
A0AAA9TP48	HARS2	A0AAA9TP48	ENSBTAG00000025212	0.012661471	-0.059129212	0.071790683
A0AAA9STE1	ACAP2	A0AAA9STE1	ENSBTAG00000054986	0.172836597	0.19592021	-0.023083613
Q2TBU2	TMX2	bta:509244; Q2TBU2; Q5E937	ENSBTAG00000002404	0.111941817	-0.061970893	0.17391271
A0A3Q1M5L7	CNOT2	A0A3Q1M5L7	ENSBTAG00000003748	0.097428011	0.101640262	-0.004212251
A0AAA9SE78	ACOX3	A0AAA9SE78	ENSBTAG00000004893	-0.208534323	-0.226320262	0.017785939
A0A3Q1MRP0	SPAST	A0A3Q1MRP0	ENSBTAG00000021694	0.022855053	-0.005770788	0.028625841

Accession	Gene_Symbol	Gene_ID	Ensembl_Gene ID	log2(ratio(OV24H/OV0H))	log2(ratio(OV24R/OV0H))	log2(ratio(OV24H/OV24R))
G3N1U2	HRAS	G3N1U2	ENSBTAG00000046644	-0.089723004	-0.125774231	0.036051227
G3N0D7	CBLB	G3N0D7	ENSBTAG00000013057	-0.290986153	-0.219511684	-0.071474469
Q5E9Q1	POGLUT1	bta:511862; Q5E9Q1	ENSBTAG00000008562	0.1335057	0.224017993	-0.090512293
Q95KV7	NDUFA13	bta:338084; Q3T0G4; Q95KV7		-0.143317366	-0.036525876	-0.10679149
A0A3S5ZPK5	WDR81	A0A3S5ZPK5	ENSBTAG00000011491	-0.18552597	0.054952311	-0.240478281
A0AAF6YSG6	EIF2B2	A0AAF6YSG6	ENSBTAG00000008664	0.153805336	0.101538026	0.05226731
F1MXE2	SIK3	bta:506539; F1MXE2	ENSBTAG00000015119	0.117941257	0.086414752	0.031526506
A0AAA9SVY3	PPP3R1	A0AAA9SVY3	ENSBTAG00000010619	0.901735984	0.202751996	0.698983988
F1MCQ6	INPP5F	F1MCQ6	ENSBTAG00000021801	0.02625324	-0.084305135	0.110558375
Q3ZBZ1	SDF4	bta:528783; Q3ZBZ1		-0.212268204	0.231486804	-0.443755008
A0AAA9SKY4	SPAG5	A0AAA9SKY4	ENSBTAG00000013100	0.004308699	-0.164362018	0.168670718
A0AAA9TJA1	CROT	A0AAA9TJA1	ENSBTAG00000021535	-0.213974728	0.049784833	-0.26375956
F1MD78	FNBP4	F1MD78	ENSBTAG00000020911	-0.232411177	-0.05598461	-0.176426567
A0AAF6YNC4	COPS7B	A0AAF6YNC4	ENSBTAG00000005207	0.253246211	0.122759893	0.130486317
Q17QN2	TOE1	bta:513052; Q17QN2	ENSBTAG00000011243	-0.14726249	-0.077184054	-0.070078436
A0AAA9SV38	RNF114	A0AAA9SV38	ENSBTAG00000027317	0.062220312	-0.007589155	0.069809467
A0A3Q1LX70	RAB34	A0A3Q1LX70	ENSBTAG00000046129	-0.146060329	-0.195303848	0.049243519
F1MNI0	SAMD4A	F1MNI0	ENSBTAG00000001468	-0.078791603	-0.151056754	0.072265151
F1MSF6	UTP3	bta:521106; F1MSF6	ENSBTAG00000009310	0.06590378	-0.014266569	0.080170349
A0AAA9SUW9	CARMIL1	A0AAA9SUW9	ENSBTAG00000016549	-0.083603646	0.032367595	-0.11597124
F1MZZ9	CCDC50	F1MZZ9	ENSBTAG00000006945	0.02816753	0.020335897	0.007831632
Q08DB5	STX5	bta:510312; Q08DB5	ENSBTAG00000009490	0.131441771	0.103562738	0.027879033
Q29460	PAFAH1B3	bta:282515; Q0VCF0; Q29460		0.109071699	-0.012644209	0.121715908
P21327	INPP1	bta:281869; P21327; Q1RMJ2	ENSBTAG00000007584	-0.056020086	-0.02558541	-0.030434675
A0AAF6YTE2	NSDHL	A0AAF6YTE2	ENSBTAG00000009231	-0.281020151	-0.248805526	-0.032214625
P81187	CFB	bta:514076; P81187; Q2KIU6	ENSBTAG00000046158	-0.01374733	0.450698487	-0.464445818
A6QLU7	TCAF2	A6QLU7; bta:510320		-0.390625833	-0.3259343	-0.064691534
F6RA71	VAT1L	F6RA71	ENSBTAG00000018146	0.011893679	0.051614108	-0.039720429
A0A3S5ZP61	PSMD10	A0A3S5ZP61	ENSBTAG00000013721	0.413362868	0.076592425	0.336770442
E1BGX5	ZNF787	E1BGX5	ENSBTAG00000002904	-0.003102571	-0.058506336	0.055403765
F1MNP7	SPC24	F1MNP7	ENSBTAG00000006185	0.318855261	0.203634363	0.115220898
A0AAA9S006	MRPS27	A0AAA9S006	ENSBTAG00000001962	-0.398363968	-0.245465069	-0.152898899
A7YY28	ABHD14B	A7YY28; bta:615289	ENSBTAG00000037377	0.168936032	0.0176865	0.151249531
A0A3Q1LSX9	FMNL2	A0A3Q1LSX9		0.192047812	0.116211974	0.075835838
A0AAA9S6N7	ERO1B	A0AAA9S6N7	ENSBTAG00000034633	-0.561385025	-0.41014749	-0.151237535
E1B716	SHC1	E1B716	ENSBTAG00000019838	-0.145956354	-0.331565788	0.185609434
A0AAF6YMI2	ABHD10	A0AAF6YMI2	ENSBTAG00000004601	0.109887904	0.2410081	-0.131120195
A0AAA9S5X7	AURKB	A0AAA9S5X7	ENSBTAG00000001717	-0.077591124	-0.292966912	0.215375787
A0AAA9TP92	CAMK1	A0AAA9TP92	ENSBTAG00000007780	0.321211939	0.428843299	-0.107631359
E1BFH7	SMC6	E1BFH7	ENSBTAG00000018256	-0.151007788	0.001492701	-0.152500489
A0AAF7APX7	WARS2	A0AAF7APX7	ENSBTAG00000005064	-0.201108863	-0.157995757	-0.043113106
F1MZ33	DNASE2	F1MZ33	ENSBTAG00000006082	-0.377738958	-0.202987022	-0.174751936
F1N579	EPN1	F1N579	ENSBTAG00000046996	0.001492701	0.032487853	-0.030995152
A0AAA9RXY8	PRKRA	A0AAA9RXY8	ENSBTAG00000001096	0.30256277	0.223813096	0.078749674
G3X6L8	NIPSNAP3A	G3X6L8	ENSBTAG00000006901	0.054401117	0.062869791	-0.008468674
F1MYQ1	TXNDC9	F1MYQ1	ENSBTAG00000004544	0.091024174	0.183333044	-0.092308871
A0AAA9TRE9	SERPINF1	A0AAA9TRE9	ENSBTAG00000009705	-0.066681279	0.491044637	-0.557725916
A0A3Q1MWG1	CARM1	A0A3Q1MWG1	ENSBTAG00000012048	-0.075288127	-0.176665148	0.10137702
A0A3Q1MR53	ZNF217	A0A3Q1MR53	ENSBTAG00000030556	-0.113610859	-0.09404349	-0.019567369
A0AAF6DLT9	GTF2H1	A0AAF6DLT9	ENSBTAG00000005160	-0.243122907	-0.094563002	-0.148559906
A0AAA9TCG7	POLR2E	A0AAA9TCG7	ENSBTAG00000020776	-0.152003093	0.089981367	-0.24198446
A0A3Q1LSC1	MLLT1	A0A3Q1LSC1	ENSBTAG00000002277	-0.143317366	-0.198031624	0.054714259
P00129	UQCRB	bta:616871; P00129; Q3SZ83	ENSBTAG00000001521	-0.060346713	0.02999474	-0.090341454
A0AAA9T797	YY1	A0AAA9T797	ENSBTAG00000020819	-0.001692311	0.113784906	-0.115477217
F6PR59	ARMC6	F6PR59	ENSBTAG00000013291	-0.074926002	-0.06216498	-0.012761022
F1MVT2	DCAF11	F1MVT2	ENSBTAG00000021392	-0.110122814	-0.012677542	-0.097445272
Q58CT1	MVK	Q58CT1		-0.089165136	0.115354042	-0.204519178
A0AAA9SPV1	CISD1	A0AAA9SPV1	ENSBTAG00000003634	-0.357592403	-0.435871003	0.078278599
A0AAF6DM76	INPP5K	A0AAF6DM76	ENSBTAG00000011479	-0.056478034	0.185373747	-0.241851781
E1BLD4	RBM28	E1BLD4	ENSBTAG00000017001	0.124470726	0.124470726	0
O77689	CCNB2	bta:281668; O77689; Q17QG1; Q5BIS1; Q5E9U8; Q5EAA0		-0.517788199	-0.341565959	-0.17622224
F1MMK3	DDX50	bta:534331; F1MMK3	ENSBTAG00000033413	0.087202763	0.005882557	0.081320207
Q08D91	KRT75	bta:539683; Q08D91		-0.038135129	2.927649156	-2.965784285
Q1LZ72	CNPY2	bta:506534; F1MSP1; Q1LZ72	ENSBTAG00000004374	0.386103721	0.282862615	0.103241106
A0A3Q1NA56	NOC3L	A0A3Q1NA56	ENSBTAG00000011368	-0.226354911	0.020798463	-0.247153374
A0AAA9TY37	PTRH2	A0AAA9TY37	ENSBTAG00000055796	0.042310805	-0.018727208	0.061038013
F6R1P1	TOR3A	F6R1P1	ENSBTAG00000014006	-0.134665777	-0.015028209	-0.119637568
E1BMD5	AVEN	E1BMD5	ENSBTAG00000044029	0.220190393	0.312656906	-0.092466513
Q5EA76	INTS14	bta:534159; Q5EA76	ENSBTAG00000015158	0.068358793	0.077041036	-0.008682243
F1N3Q9	PNKP	F1N3Q9	ENSBTAG00000001834	-1.049881149	-0.538767678	-0.511113471
A7MBI4	DHX32	A7MBI4; bta:532978; F1N3H3	ENSBTAG00000005795	0.150140349	-0.063888599	0.214028948
F1MDA7	FBXO7	F1MDA7	ENSBTAG00000020634	-0.01218614	0.028040305	-0.040226445
E1BLP4	MFF	E1BLP4	ENSBTAG00000021319	0.247461602	0.059247681	0.188213921
A6QLB8	GSK3A	A6QLB8; bta:536561; F1MHW8		0.073384499	0.024430039	0.04895446
F6QFI8	NOP53	F6QFI8	ENSBTAG00000021192	-0.324788379	-0.199920409	-0.12486797
Q56JY1	RPL35A	bta:506768; Q56JY1	ENSBTAG00000014208	-0.115914067	-0.187226674	0.071312607
A0A3Q1MC59	DOCK5	A0A3Q1MC59	ENSBTAG00000015513	-0.153489642	-0.028509722	-0.12497992
P60984	GMFB	A7E313; bta:615255; P17774; P60984	ENSBTAG00000054095	-0.012977854	-0.079678255	0.066700401
E1B828	LEMD2	bta:518023; E1B828	ENSBTAG00000015538	0.079192167	0.119992699	-0.040800531
F1MPB1	GNPTAB	F1MPB1	ENSBTAG00000019409	-0.134144884	-0.011252275	-0.122892609
Q58CX1	GALT	bta:506997; F1MQ04; Q58CX1		-0.054350097	-0.019176557	-0.03517354
G3N2J7	DMXL1	G3N2J7	ENSBTAG00000006748	-0.262537011	-0.140990101	-0.12154691
A0A3Q1LX85	FAM169A	A0A3Q1LX85	ENSBTAG00000016002	-0.475755728	-0.269040281	-0.206715447
P82908	KGD4	bta:613835; P82908; Q3SYR1		0.08590905	0.097567255	-0.011658205
Q7YRA3	EXOSC4	bta:618292; Q32L11; Q7YRA3	ENSBTAG00000014607	-0.4885718	-0.092304104	-0.396267695
Q2TBK2	MRPL9	bta:505981; Q2TBK2	ENSBTAG00000000797	-0.380604002	-0.267746533	-0.112857469
A0AAA9TGL5	FAM162A	A0AAA9TGL5	ENSBTAG00000011140	0.025606656	-0.08110305	0.106709706
A0AAA9TNC5	RPUSD2	A0AAA9TNC5	ENSBTAG00000057394	-0.2315079	-0.18150452	-0.05000338
F1MH28	TLE1	F1MH28	ENSBTAG00000000256	-0.06241808	-0.292976721	0.230558641
A0AAA9T0I3	RMDN1	A0AAA9T0I3	ENSBTAG00000015734	-0.419538892	-0.383914982	-0.03562391
A0A3Q1M2R1	RC3H1	A0A3Q1M2R1	ENSBTAG00000052708	0.088024748	0.101445264	-0.013420516
E1BG99	EIF4ENIF1	E1BG99	ENSBTAG00000008991	0.238159737	-0.003220303	0.24138004
A7E3S4	RAF1	A7E3S4; A7YY51; bta:521196	ENSBTAG00000045748	-0.111645356	-0.106128351	-0.005517005
A0AAA9SZG2	CEP41	A0AAA9SZG2	ENSBTAG00000007413	0.323764753	0.277763686	0.046001068
A0A0A8J2N9	ASCT2	A0A0A8J2N9; bta:282355		0.067970727	0.110225563	-0.042254835
F6RJ01				0.057333175	-0.093345737	0.150678912
P00435	GPX1	A6QPG3; bta:281209; P00435	ENSBTAG00000054195	0.272843929	0.523488591	-0.250644662
Q6EWQ6	DHPS	bta:444988; Q3MHI1; Q6EWQ6	ENSBTAG00000006247	-0.335662206	-0.40801305	0.072350844
A0AAA9SLY8	RICTOR	A0AAA9SLY8	ENSBTAG00000006697	-0.191119224	-0.061400545	-0.129718679
E1BM03	WDR3	bta:514870; E1BM03	ENSBTAG00000019937	-0.225182395	-0.289135028	0.063952633
Q2TBN7	MED27	bta:525389; Q2TBN7	ENSBTAG00000000382	-0.067114196	0.1431829	-0.210297096
A0AAA9TLZ1	FBLN1	A0AAA9TLZ1	ENSBTAG00000012088	-0.030726516	0.225941488	-0.256668004
Q2TA12	MRPL2	bta:514403; Q2TA12	ENSBTAG00000012752	0.039091971	0.027619187	0.011472784
A0AAA9SEU1	SNX30	A0AAA9SEU1	ENSBTAG00000044070	0.042378602	0.079853308	-0.037474705
A0A3Q1MCZ1	PURB	A0A3Q1MCZ1; bta:504276	ENSBTAG00000052945	-0.158756506	-0.226609598	0.067853092
A7Z063	WASHC1	A7Z063; bta:533602	ENSBTAG00000011902	0.016425548	0.132740081	-0.116314533
Q0VCP0	SLC33A1	bta:517797; F1N5E5; Q0VCP0	ENSBTAG00000014318	0.211037526	0.285101438	-0.074063912
A0A3Q1M4W7	EHMT2	A0A3Q1M4W7	ENSBTAG00000005676	-0.393201698	-0.337034987	-0.056166711
Q3SZ40	DDX56	bta:508728; Q3SZ40		-0.13290651	-0.216358944	0.083452435
A0AAA9SWX2	NF2	A0AAA9SWX2	ENSBTAG00000013153	0.084205298	0.07287888	0.011326418
A0A3Q1MG93	OSBPL1A	A0A3Q1MG93	ENSBTAG00000023259	-0.195067427	-0.009151698	-0.185915729
A0A3Q1LKT6	STXBP5	A0A3Q1LKT6	ENSBTAG00000012107	-0.13763613	-0.254572827	0.116936697
A0A3Q1NMC5	RPS24	A0A3Q1NMC5	ENSBTAG00000013264	0.041349259	-0.012035065	0.053384324
A0AAF6YTR5	POLR2G	A0AAF6YTR5	ENSBTAG00000009483	-0.01841462	-0.390383397	0.371968777
Q7M2Y2				-0.111629727	-0.182386386	0.07075666
A0AAA9THU3	EZH2	A0AAA9THU3	ENSBTAG00000009426	-0.21649182	-0.214965966	-0.001525854
F1N322	NISCH	F1N322	ENSBTAG00000014779	0.017647866	0.023482839	-0.005834973
F1N498	MRPS11	F1N498	ENSBTAG00000019831	-0.216850538	-0.143018309	-0.073832229
F1MJA6	TEX2	F1MJA6	ENSBTAG00000004723	-0.526791631	-0.264067115	-0.262724515
Q58D62	FETUB	bta:504615; Q58D62	ENSBTAG00000017531	0.026939072	0.630073631	-0.603134559
F1MMK9	AMBP	F1MMK9	ENSBTAG00000000375	-0.1686594	0.452178235	-0.620837635
A0AAF7AAT7	ARMC8	A0AAF7AAT7	ENSBTAG00000019952	0.062673805	0.107954685	-0.045280879
A4IFE9	TM9SF1	A4IFE9; bta:539478		-0.045385322	-0.126448335	0.081063013
A0A3Q1LSN6	GSTM3	A0A3Q1LSN6	ENSBTAG00000001842	-0.011564752	0.221157766	-0.232722517
A0AAA9SVD5	LRIF1	A0AAA9SVD5	ENSBTAG00000012659	0.169925001	0.050626073	0.119298928
C7FFR6	DPM1	C7FFR6; F1MLE6	ENSBTAG00000007721	0.24076813	0.246038255	-0.005270125
A0AAA9TS07	WDR45B	A0AAA9TS07	ENSBTAG00000003695	0.173648087	0.21203191	-0.038383822
A0AAA9T7J6	TNFAIP2	A0AAA9T7J6	ENSBTAG00000035995	-0.020163781	0.130112325	-0.150276106
Q2TA37	ARL2	bta:511349; Q2TA37	ENSBTAG00000022238	-0.068975614	-0.29655844	0.227582826
A2VE31	SLC38A2	A2VE31; bta:338044; Q95M41	ENSBTAG00000011105	-0.108398949	-0.110131917	0.001732967
A0A3Q1LTM3	EPG5	A0A3Q1LTM3; bta:529859	ENSBTAG00000012067	-0.075795351	-0.049105472	-0.026689878
A0AAA9STH9	LYPLA1	A0AAA9STH9	ENSBTAG00000004243	0.370087475	0.243042931	0.127044545
F1N5B5	REXO4	bta:100140713; F1N5B5	ENSBTAG00000019721	-0.364663977	-0.242673452	-0.121990524
A0AAA9TB64				0.029564458	0.10510056	-0.075536103
A6QLI8	GTF2E1	A6QLI8; bta:540525	ENSBTAG00000016848	0.125018652	-0.072464983	0.197483635
A0A3Q1M3Z1	CD46	A0A3Q1M3Z1	ENSBTAG00000005397	0.316027493	0.282020885	0.034006608
A0A3Q1MS48				0.184424571	0.686245837	-0.501821265
A4IFU2	RAP2B	A4IFU2; bta:784133	ENSBTAG00000055257	0.135852043	0.049421599	0.086430444
A0AAF6DME4	NPEPL1	A0AAF6DME4	ENSBTAG00000016724	-0.107246515	0.185629902	-0.292876417
A6QLD9	GALNT16	A6QLD9; bta:526331	ENSBTAG00000009998	0.108117617	-0.136801994	0.244919611
A0A3Q1LUF0	ARID5B	A0A3Q1LUF0	ENSBTAG00000051333	0.103678646	-0.015642978	0.119321624
E1BFH5	TSC22D4	E1BFH5	ENSBTAG00000015272	0.073206086	0.055249861	0.017956226
A0AAF6YME1	RHOT2	A0AAF6YME1	ENSBTAG00000004551	-0.104500314	-0.061514389	-0.042985925
Q3SZ86	MRPS26	bta:516004; Q3SZ86		-0.139056643	-0.192846558	0.053790914
A5PJN6	UTP6	A5PJN6; bta:614984		0.065650532	0.090833722	-0.02518319
A0AAA9TW47	GSTK1	A0AAA9TW47	ENSBTAG00000009839	-0.11450625	-0.144253594	0.029747343
E1B7Z0	TAB2	bta:540203; E1B7Z0	ENSBTAG00000013454	0.046277683	-0.046244185	0.092521868
A2I7M9	SERPINA3-2	A2I7M9		0.004496715	0.740863561	-0.736366841
F1N4W0	PIGT	F1N4W0	ENSBTAG00000016122	-0.134904073	0.032161275	-0.167065347
A0AAA9T5Y4	RUFY3	A0AAA9T5Y4	ENSBTAG00000016795	0.110367394	0.2328514	-0.122484007
F1MPS0	NCAPD3	F1MPS0	ENSBTAG00000002260	-0.406467455	-0.290713515	-0.11575394
F1MQH3	FERMT1	F1MQH3	ENSBTAG00000020465	-0.308065846	-0.11597124	-0.192094606
F1MS24	CHM	bta:519552; F1MS24	ENSBTAG00000009598	0.005208291	0.006507431	-0.00129914
A0AAA9TJA9	TMEM87A	A0AAA9TJA9	ENSBTAG00000008863	0.077629337	0.350705744	-0.273076407
Q2YDI0	MRPL11	bta:515990; Q2YDI0; Q58DQ9		-0.245960892	-0.09158355	-0.154377342
A6QLY2	GPX7	A6QLY2; bta:523311		-0.195395689	-0.080523705	-0.114871984
E1BKU3				-0.049105472	0.036454876	-0.085560348
G5E5D4	MARK1	G5E5D4	ENSBTAG00000010460	-0.148357621	-0.148357621	0
A0A3Q1MRI3	CALCOCO1	A0A3Q1MRI3	ENSBTAG00000015016	0.053363226	0.303127757	-0.249764532
A8E4L9	SYNC	A8E4L9; bta:618122	ENSBTAG00000026684	-0.049935699	0.045385322	-0.095321021
F1MDE6	APBB2	F1MDE6	ENSBTAG00000027569	-0.065402475	-0.498675546	0.433273071
Q28036	SLC9A1	bta:317654; F1MHK2; Q28036	ENSBTAG00000008766	-0.100511237	0.071039337	-0.171550573
A0AAA9TPX3	IL18	A0AAA9TPX3	ENSBTAG00000000277	0.179032432	0.408951157	-0.229918725
A0AAA9S0B2	GRB10	A0AAA9S0B2	ENSBTAG00000017086	-0.227951947	-0.239551567	0.011599621
A4FUZ1	GLO1	A4FUZ1; bta:540335; F1MUW8	ENSBTAG00000012703	0.514070987	0.375160414	0.138910574
F1MYV2	KDM5C	F1MYV2	ENSBTAG00000014943	0.010514222	-0.012110837	0.022625059
A0A3Q1N546	CNOT4	A0A3Q1N546	ENSBTAG00000017919	0.134367674	0.202350178	-0.067982505
Q3T0U5	CHMP2A	bta:523847; Q3T0U5	ENSBTAG00000013882	0.288887833	0.041917389	0.246970444
A0A3Q1M2E0	WDR36	A0A3Q1M2E0	ENSBTAG00000053476	-0.066427362	-0.040971781	-0.025455581
A0AAA9TT41	ARF6	A0AAA9TT41; bta:100294979	ENSBTAG00000068075	-0.005619073	-0.057200556	0.051581484
F1MYI1	ARNT	F1MYI1	ENSBTAG00000021037	0.206565646	0.194580571	0.011985074
A0AAF6YSV5	WDR89	A0AAF6YSV5	ENSBTAG00000008815	-0.037519007	0.107035444	-0.14455445
A0A3Q1LXI8	TMED3	A0A3Q1LXI8	ENSBTAG00000000807	0.034298242	-0.004534404	0.038832646
Q0P5F7	PIP4K2C	bta:540005; Q0P5F7		0.180743852	0.351669484	-0.170925632
A0A3Q1NM09	ARB2A	A0A3Q1NM09	ENSBTAG00000050195	-0.232755591	-0.104874135	-0.127881455
A0AAA9TRW7	ERGIC3	A0AAA9TRW7	ENSBTAG00000006670	0.184297735	0.046794211	0.137503524
A0JND3	EOGT	A0JND3; bta:508782	ENSBTAG00000022681	-0.01927293	0.114319652	-0.133592582
F6RRD7	RPAP2	A4FV88; bta:529184; F6RRD7		0.062958632	0.109478477	-0.046519845
Q5E938	EIF1	bta:509764; Q5E938	ENSBTAG00000002282	0.850423644	0.58058406	0.269839584
F1N2J9	RAB8B	F1N2J9	ENSBTAG00000011146	0.164102841	0.242899849	-0.078797007
Q2KJD3	CWC15	bta:535258; Q2KJD3	ENSBTAG00000007913	-0.006080915	-0.102138642	0.096057727
A0AAA9SKM0	ADARB1	A0AAA9SKM0	ENSBTAG00000017486	0.152142612	0.041261965	0.110880647
G3MXZ7	NAGLU	G3MXZ7	ENSBTAG00000047956	0.007181175	0.081181756	-0.074000581
P56602	PPOX	bta:515770; E1BPX0; P56602	ENSBTAG00000021894	-0.095614395	-0.014824647	-0.080789747
A0AAA9SXG0	YTHDC1	A0AAA9SXG0	ENSBTAG00000015572	-0.063784508	0.104195087	-0.167979596
A0AAA9T1I5	PDCL	A0AAA9T1I5	ENSBTAG00000030675	0.306387244	-0.022720077	0.32910732
F1MFY1	MORC3	F1MFY1	ENSBTAG00000011876	-0.090889141	-0.116469511	0.025580369
A0A3Q1MQ73	POFUT1	A0A3Q1MQ73	ENSBTAG00000016231	0.093511886	0.164188632	-0.070676746
A0AAA9RZJ0	TOM1L2	A0AAA9RZJ0	ENSBTAG00000003983	0.037259619	0.149904628	-0.112645009

Accession	Gene_Symbol	Gene_ID	Ensembl_Gene ID	log2(ratio(OV24H/OV0H))	log2(ratio(OV24R/OV0H))	log2(ratio(OV24H/OV24R))
A0AAF6Z0J2	OXA1L	A0AAF6Z0J2	ENSBTAG00000014820	-0.109014586	-0.11490916	0.005894574
E1BDJ1	XPC	E1BDJ1	ENSBTAG00000007362	-0.209346535	-0.169070577	-0.040275958
A0AAA9S5M1	BAG1	A0AAA9S5M1	ENSBTAG00000017845	-0.066709342	-0.01854206	-0.048167282
A0AAA9SNG8	PYM1	A0AAA9SNG8	ENSBTAG00000031146	-0.030088578	-0.053074116	0.022985537
A0AAA9TDU0	BLM	A0AAA9TDU0	ENSBTAG00000020301	0.054647396	0.151643722	-0.096996326
F1MQV1	MPHOSPH10	F1MQV1	ENSBTAG00000009854	0.076139768	0.156835002	-0.080695234
A0AAA9TPN6	SLU7	A0AAA9TPN6	ENSBTAG00000008457	0.256757656	0.290545969	-0.033788313
Q6TUI4	DICER1	bta:337871; Q6TUI4		0.050914272	0.093840597	-0.042926325
A0AAA9SVY0	COMMD4	A0AAA9SVY0	ENSBTAG00000007346	0.043019324	0.151331135	-0.108311811
F1MPW4	SMC5	bta:508938; F1MPW4	ENSBTAG00000018437	-0.186953966	0.050429427	-0.237383393
A6H7C1	MORF4L2	A6H7C1; bta:538442	ENSBTAG00000011311	-0.180098065	-0.154534693	-0.025563372
A0AAA9T4J2	MOCS3	A0AAA9T4J2	ENSBTAG00000066776	-0.576380113	-0.325663044	-0.25071707
A0A452DHY5	MAGOH	A0A452DHY5	ENSBTAG00000003485	0.071553261	-0.101283336	0.172836597
A6QQC2	TNFRSF10A	A6QQC2		-0.071216799	-0.44625623	0.375039431
A5D9H4	CHAF1B	A5D9H4; bta:511654	ENSBTAG00000011880	-0.133514361	-0.126788498	-0.006725863
A0AAA9SQC4	ZNF330	A0AAA9SQC4	ENSBTAG00000000297	0.093009325	0.101988784	-0.008979459
A0A3Q1LJ78	LPGAT1	A0A3Q1LJ78	ENSBTAG00000005137	0.17391787	0.11321061	0.060707259
A0A3Q1MBW0	CRYL1	A0A3Q1MBW0	ENSBTAG00000011726	0.329493372	0.318586764	0.010906608
G3MXQ8	MAN1B1	G3MXQ8	ENSBTAG00000046775	-0.608718624	-0.403004944	-0.20571368
A0A3Q1M9P9	VCAN	A0A3Q1M9P9	ENSBTAG00000014906	-0.087071708	-0.202964508	0.1158928
E1BNX1	RAB3D	E1BNX1	ENSBTAG00000065529	-0.143835773	-0.112759369	-0.031076404
Q02373	NDUFB10	bta:327701; Q02373; Q3ZC99	ENSBTAG00000009534	-0.444563452	-0.396558079	-0.048005373
A0AAA9TUM5	APOOL	A0AAA9TUM5	ENSBTAG00000021790	-0.052308873	-0.142363494	0.090054621
Q2KIJ8	PLD3	bta:613932; Q2KIJ8	ENSBTAG00000019150	0.01536431	0.032078183	-0.016713874
A0A3Q1MQS2	CXADR	A0A3Q1MQS2	ENSBTAG00000004869	0.173192715	0.40302652	-0.229833805
E1BPX6	POLR3B	E1B9R0; E1BPX6	ENSBTAG00000004781	0.066466909	0.088950923	-0.022484014
A0AAA9RXM2	TMEM263	A0AAA9RXM2	ENSBTAG00000053105	0.195071815	0.134409033	0.060662782
F6PWS3	RALB	F6PWS3	ENSBTAG00000032021	0.213357278	0.263652395	-0.050295116
A7MB64	ITPRIP	A7MB64; bta:538685	ENSBTAG00000003990	-0.059231122	0.004043053	-0.063274175
F1N0W0	HDAC3	bta:404125; F1N0W0	ENSBTAG00000017360	-0.429684275	-0.373633746	-0.056050529
Q2HJH3				-0.033276155	-0.025283364	-0.007992791
A0A3Q1N8U1	UBAP1	A0A3Q1N8U1	ENSBTAG00000000320	0.059352134	0.049891805	0.009460329
Q1RMH8	SNX3	bta:528265; Q1RMH8	ENSBTAG00000018801	-0.102380593	-0.12040585	0.018025257
Q2NL29	ISYNA1	bta:509394; Q2NL29		-0.051974275	0.058716116	-0.110690391
G3MWH1	TPD52L2	G3MWH1	ENSBTAG00000021232	0.398163681	0.675273272	-0.277109591
F1MHR3	ALDH1A3	F1MHR3	ENSBTAG00000009125	-0.072209986	0.020976549	-0.093186535
A0AAF6YIA9	CYP51A1	A0AAF6YIA9	ENSBTAG00000001992	-0.449259814	-0.046522302	-0.402737511
E1BEL9	COASY	E1BEL9	ENSBTAG00000046746	0.149025394	0.264961119	-0.115935725
P82924	MRPS30	bta:516084; P82924; Q05B75	ENSBTAG00000000084	-0.01373279	0.143015259	-0.15674805
E1BB87	RIOK1	bta:516289; E1BB87	ENSBTAG00000013821	0.197213858	0.150004901	0.047208958
Q3ZBF7	PTGES3	bta:493638; Q3ZBF7; Q66LN1	ENSBTAG00000017967	0.275495508	0.155832412	0.119663096
A0AAA9SHC7	FAM118B	A0AAA9SHC7	ENSBTAG00000012169	0.021396293	-0.039015223	0.060411517
Q08E26	CRTC2	bta:540959; Q08E26		-0.12439743	0.017904798	-0.142302229
F1N7R5	CC2D1B	F1N7R5	ENSBTAG00000015896	0.063174198	0.055261774	0.007912424
Q58CQ5	ANKZF1	bta:507867; Q58CQ5		-0.292008582	-0.228027956	-0.063980626
F1MF92	ZMYM4	F1MF92	ENSBTAG00000021023	-0.061809812	-0.042294798	-0.019515013
Q2KIC5	ITPA	bta:613653; Q2KIC5		-0.200836278	-0.063888599	-0.136947679
A0AAA9TJE2	TATDN1	A0AAA9TJE2	ENSBTAG00000033446	-0.155806668	-0.101979677	-0.053826991
F1MU45	PCF11	F1MU45	ENSBTAG00000015277	-0.162176978	-0.090027192	-0.072149786
A0A3Q1N203	ARPIN	A0A3Q1N203	ENSBTAG00000027059	-0.00140957	0.008428622	-0.009838192
A0AAF6Z667	S100A4	A0AAF6Z667	ENSBTAG00000019203	0.259386629	0.380751833	-0.121365205
A6QQS5	NELFA	A6QQS5; bta:789389; F1MW50	ENSBTAG00000017921	-0.34058755	-0.167062677	-0.173524873
A0A3Q1M360	FBXO30	A0A3Q1M360	ENSBTAG00000005376	-0.232795974	0.258068153	-0.490864126
Q0VD41	CHN2	E1BEJ8; Q0VD41	ENSBTAG00000020931	-0.032484961	0.095899918	-0.128384879
Q3SZ15	EEF1E1	bta:617105; F1ML35; Q3SZ15	ENSBTAG00000002534	0.279488338	0.210956956	0.068531382
A0AAA9SHE6	KIRREL1	A0AAA9SHE6	ENSBTAG00000005799	0.142901828	0.125282975	0.017618853
Q08DM8	TSN	A1L585; bta:509943; Q08DM8	ENSBTAG00000006059	-0.185638289	-0.129239119	-0.05639917
A0AAA9S4U6	RNF2	A0AAA9S4U6	ENSBTAG00000000023	0.079607858	-0.158181668	0.237789526
A0A3Q1N6L4	ACOT8	A0A3Q1N6L4	ENSBTAG00000020371	0.1844767	-0.017042484	0.201519184
A7MB19	NLRX1	A7MB19; bta:539974		-0.066540856	0.125631233	-0.192172089
A0AAF7A0B6	MBOAT7	A0AAF7A0B6	ENSBTAG00000015908	0.01504388	0.016539676	-0.001495796
A0A3Q1MIH5	SYNJ1	A0A3Q1MIH5	ENSBTAG00000003063	-0.203126562	0.055607707	-0.258734268
A0A3Q1M1S3	CTIF	A0A3Q1M1S3; bta:615119	ENSBTAG00000018901	-0.194256128	0.052568051	-0.246824179
A3KN22	UBE2M	A3KN22; bta:613343	ENSBTAG00000012744	0.074305559	0.081529885	-0.007224327
G5E534	RPIA	bta:613376; G5E534	ENSBTAG00000002866	0.086448169	0.251251349	-0.16480318
A0A3Q1MJU3	IBTK	A0A3Q1MJU3	ENSBTAG00000026716	0.146325763	0.154180747	-0.007854983
A0AAA9S920	FAHD2A	A0AAA9S920	ENSBTAG00000011553	0.350242376	0.467678979	-0.117436603
E1BI49	MAN1A2	bta:532645; E1BI49	ENSBTAG00000013586	-0.193501938	-0.066257845	-0.127244093
A0AAA9TV39	BCL2L13	A0AAA9TV39	ENSBTAG00000013956	0.762829523	0.287333002	0.475496521
A0AAA9SZK0	NCBP2	A0AAA9SZK0	ENSBTAG00000008873	0.064936012	0.10713585	-0.042199838
P80747	ITGB5	A3KMX2; P80747		-0.356504851	-0.469782838	0.113277987
A0AAA9T3R5	BCL9L	A0AAA9T3R5	ENSBTAG00000005138	-0.166046042	-0.246263825	0.080217783
A0AAA9SNZ0	VPS37C	A0AAA9SNZ0	ENSBTAG00000008064	-0.017341897	0.196782909	-0.214124805
A0AAF6YW51	PFN2	A0AAF6YW51	ENSBTAG00000011150	-0.134949629	-0.20983684	0.074887212
A0AAF7AT19	PKN1	A0AAF7AT19	ENSBTAG00000017037	-0.470414256	-0.265450981	-0.204963275
A0AAA9TFC0	PDCD5	A0AAA9TFC0	ENSBTAG00000009304	0.200782127	0.28757659	-0.086794463
A6QLP7	SSR1	A6QLP7; bta:529312	ENSBTAG00000022731	0.030534368	-0.037515311	0.068049679
A0AAA9SKF2	EIF2D	A0AAA9SKF2	ENSBTAG00000010432	-0.071258683	-0.030743523	-0.04051516
A0AAF6ZEA8	AK4	A0AAF6ZEA8	ENSBTAG00000030674	-0.015961141	-0.010621116	-0.005340025
A0AAA9T9L5	GYG2	A0AAA9T9L5	ENSBTAG00000013341	-0.171898225	0.194257528	-0.366155753
Q3ZBX6	MRPL3	bta:614906; Q3ZBX6	ENSBTAG00000030942	-0.1863204	-0.171101957	-0.015218443
E1BIZ3	UNC119B	bta:617896; E1BIZ3	ENSBTAG00000007483	-0.107660205	-0.142352923	0.034692718
A0A3Q1M1W2	PON2	A0A3Q1M1W2	ENSBTAG00000008361	0.220190393	0.27779066	-0.057600267
A2VDP0				0.192458575	0.200271022	-0.007812447
A0AAF6YT25	FAM136A	A0AAF6YT25	ENSBTAG00000008674	0.220198208	0.265590111	-0.045391903
A0AAA9T341	LOC532875	A0AAA9T341; bta:532875	ENSBTAG00000038051	-0.554460412	-0.116962236	-0.437498177
A0AAA9T1Y1	NIP7	A0AAA9T1Y1	ENSBTAG00000001666	-0.071151516	-0.17056013	0.099408614
A0AAA9TUB9	SSR4	A0AAA9TUB9	ENSBTAG00000021421	0.123646941	0.038219322	0.085427619
Q2T9M8	SNAP23	bta:522423; F1N473; Q2T9M8	ENSBTAG00000005661	0.18606493	0.136160024	0.049904906
A0A3Q1NA87	CRIM1	A0A3Q1NA87	ENSBTAG00000009020	0.184473767	0.259339922	-0.074866155
A0A3Q1LUZ8	TOP3B	A0A3Q1LUZ8; bta:537418	ENSBTAG00000053372	-0.075017229	-0.051956677	-0.023060552
F1MG95	PARG	F1MG95	ENSBTAG00000023018	-0.027316417	-0.204699104	0.177382686
E1BL67	OSBPL2	E1BL67	ENSBTAG00000017402	0.018615678	-0.154086408	0.172702086
A0AAA9S740	PEX14	A0AAA9S740	ENSBTAG00000013538	-0.215459707	-0.158503822	-0.056955885
G3MWG5	XIAP	G3MWG5	ENSBTAG00000040350	0.166399315	0.235911357	-0.069512043
Q1LZG6	CCNB1	bta:327679; Q1LZG6	ENSBTAG00000014239	-0.250356048	-0.480156419	0.229800371
A0A3Q1N402	BIRC2	A0A3Q1N402	ENSBTAG00000035735	0.040233925	0.156077355	-0.11584343
A0AAF6YPV7	CHMP4A	A0AAF6YPV7	ENSBTAG00000039415	0.203202861	0.182602391	0.02060047
A0AAA9TX02	INTS12	A0AAA9TX02	ENSBTAG00000033648	-0.299979122	-0.095652239	-0.204326883
F1N3F2	IRF2BPL	F1N3F2	ENSBTAG00000025329	-0.044590151	-0.15549208	0.110901929
A0A3Q1LVE2	SASS6	A0A3Q1LVE2	ENSBTAG00000015778	0.131928653	-0.01759406	0.149522713
A0A3Q1MIH6	MAVS	A0A3Q1MIH6	ENSBTAG00000013545	-0.063496604	-0.12714528	0.063648676
A0AAA9SU87	SRFBP1	A0AAA9SU87	ENSBTAG00000014320	0.30991113	-0.001663049	0.311574179
F6S1J8	YES1	F6S1J8	ENSBTAG00000001523	0.109181811	0.138328157	-0.029146346
A0AAF6YUU2	EMC2	A0AAF6YUU2	ENSBTAG00000010006	0.145912382	0.079348781	0.066563601
A0AAA9TEN0	ACADSB	A0AAA9TEN0	ENSBTAG00000018041	-0.299851588	-0.255104378	-0.04474721
E1BPZ7	FBXO38	E1BPZ7	ENSBTAG00000020303	0.101283336	0.229209528	-0.127926192
A0AAA9TRI4	CDC123	A0AAA9TRI4	ENSBTAG00000019511	0.158637512	0.230629355	-0.071991843
Q95108	TXN2	bta:281557; Q2KHU7; Q95108	ENSBTAG00000000014	1.02179859	0.325622578	0.696176012
E1BNN3	RALGPS2	E1BNN3	ENSBTAG00000010718	-0.474053187	-0.163762262	-0.310290925
A0A3Q1M9B2	ARHGAP21	A0A3Q1M9B2	ENSBTAG00000021857	-0.005208291	0.041002974	-0.046211265
A0AAF6DLX2	NTN4	A0AAF6DLX2	ENSBTAG00000003183	0.109472485	-0.432803617	0.542276102
A0A3Q1M9D2	MPST	A0A3Q1M9D2	ENSBTAG00000030648	-0.017104656	0.087539779	-0.104644435
F1ML97	NOA1A	F1ML97	ENSBTAG00000019362	-0.193569586	-0.198015491	0.004445905
G5E6J3	MED16	G5E6J3	ENSBTAG00000047217	-0.255133397	-0.131001153	-0.124132244
A0AAA9TXS8	MECP2	A0AAA9TXS8	ENSBTAG00000047855	0.15565087	0.271586596	-0.115935725
A0A3Q1LWP8	WDR35	A0A3Q1LWP8	ENSBTAG00000051164	-0.068131	-0.219256808	0.151125809
Q2NL17	CLPTM1	bta:618037; Q2NL17	ENSBTAG00000020560	-0.443497567	-0.269046783	-0.174450785
A0AAA9SCK0	FKBP1A	A0AAA9SCK0	ENSBTAG00000008303	0.144663315	0.181838323	-0.037175008
A0A452DHW6	MYD88	A0A452DHW6	ENSBTAG00000000563	0.197899386	0.092134281	0.105765105
G3MZN5	NCKAP5L	G3MZN5	ENSBTAG00000004851	-0.06120515	-0.018090457	-0.043114693
A0AAA9S5V1	USP25	A0AAA9S5V1	ENSBTAG00000019314	-0.304226093	-0.216073462	-0.088152631
A0A452DHY9	PHF10	A0A452DHY9	ENSBTAG00000003929	0.089765961	-0.136257289	0.22602325
Q08DZ5	SDC1	bta:529759; Q08DZ5		-0.283792966	-0.495480492	0.211687526
A0AAA9SW70	VAMP3	A0AAA9SW70	ENSBTAG00000064040	0.212910926	0.328388144	-0.115477217
A0AAA9U1C9	DNAJB12	A0AAA9U1C9	ENSBTAG00000013651	0.013100613	0.048525708	-0.035425096
A0A8E6GNW9	COX2	A0A8E6GNW9		-0.10491501	-0.122118082	0.017203072
A0A3Q1LQ88	DAB2IP	A0A3Q1LQ88	ENSBTAG00000008232	-0.205897485	-0.175783212	-0.030114273
Q08DT6	MRPL47	bta:534317; Q08DT6		-0.271302022	-0.153530563	-0.117771459
F1MD83				-0.254997087	0.012838305	-0.267835392
A0AAF6ZCD9	TTC5	A0AAF6ZCD9	ENSBTAG00000009372	-0.20029865	-0.174678531	-0.025620119
A0AAA9TGD7	YJU2	A0AAA9TGD7	ENSBTAG00000012830	0.058709307	-0.009248077	0.067957384
A0A452DJF8	NAGK	A0A452DJF8	ENSBTAG00000014668	-0.110580541	0.18101418	-0.291594721
Q0VCS9	ANKMY2	bta:509032; Q0VCS9	ENSBTAG00000010980	0.096393955	0.00138388	0.095010075
E1BNI6	TMED8	E1BNI6	ENSBTAG00000020469	0.026119061	-0.072541102	0.098660163
A0A3Q1M7G4	INTS13	A0A3Q1M7G4	ENSBTAG00000013515	-0.04425792	-0.107026967	0.062769047
G3N0S9				-0.240858729	0.096904218	-0.337762947
A0AAA9T7D0	BCAS3	A0AAA9T7D0	ENSBTAG00000002663	-0.037541962	-0.068027236	0.030485274
F1MES6	UBXN6	F1MES6	ENSBTAG00000008186	0.167928191	0.143748379	0.024179812
A0AAF6YLJ6	PAAF1	A0AAF6YLJ6	ENSBTAG00000003679	0.08979667	-0.016268389	0.106065059
F1MEK3	SCAF1	F1MEK3	ENSBTAG00000006612	-0.034121885	-0.182626322	0.148504438
A0A3Q1LL49	MORF4L1	A0A3Q1LL49	ENSBTAG00000010988	-0.25091812	-0.1835689	-0.06734922
F1MWY0	NSMAF	F1MWY0	ENSBTAG00000008958	0.005912693	-0.113996771	0.119909464
G3X7D8	MRRF	G3X7D8	ENSBTAG00000018426	0.134266729	0.094268661	0.039998068
A0AAA9T4F5	PDCD6	A0AAA9T4F5	ENSBTAG00000046493	0.269526397	0.055695989	0.213830408
F1MX69	BPGM	F1MX69	ENSBTAG00000008895	0.082880272	0.024874669	0.058005603
A0AAF6Z8F0	CASP4	A0AAF6Z8F0	ENSBTAG00000020884	-0.213505635	-0.256574357	0.043068722
A0A3Q1MF31	TRAPPC9	A0A3Q1MF31	ENSBTAG00000013955	-0.437877476	-0.159576313	-0.278301162
E1BDS1	MAGED1	bta:512562; E1BDS1	ENSBTAG00000008733	-0.34580046	-0.159813685	-0.185986775
A0AAF6YI61	ABHD12	A0AAF6YI61	ENSBTAG00000001420	-0.435455262	-0.414226769	-0.021228493
E1BMM4	VPS26B	bta:614321; E1BMM4	ENSBTAG00000014838	0.013925736	0.080283802	-0.066358066
E1BKB2	NGLY1	bta:533736; E1BKB2	ENSBTAG00000003793	-0.188650728	-0.1869722	-0.001678528
Q1LZ99	DMAP1	bta:508127; Q1LZ99	ENSBTAG00000005912	0.017277991	0.037434188	-0.020156196
A0A3Q1M4Y7	SBF1	A0A3Q1M4Y7	ENSBTAG00000021337	-0.172069537	-0.057850069	-0.114219469
A0A3Q1M7F4	CTU2	A0A3Q1M7F4	ENSBTAG00000020943	-0.176156955	-0.015281458	-0.160875497
A0AAA9TG97	ATG4B	A0AAA9TG97	ENSBTAG00000015401	0.007549442	-0.188973272	0.196522714
Q5E992	PPIL1	bta:508179; Q5E992	ENSBTAG00000002376	-0.11733182	0.040930264	-0.158262084
Q3SZ22	MRPL46	bta:540720; Q3SZ22	ENSBTAG00000019830	0.04221176	0.0993072	-0.05709544
Q2TBU5	NT5DC1	bta:100125914; Q2TBU5	ENSBTAG00000003076	-0.097358877	0.043107722	-0.140466599
A0A3Q1NJG8				-0.044622991	-0.055236665	0.010613674
Q0VCI1	OSGEP	bta:507512; Q0VCI1	ENSBTAG00000002743	-0.032688619	0.03196434	-0.064652958
A0AAA9S900	RBKS	A0AAA9S900	ENSBTAG00000031332	-0.009586049	0.11179781	-0.121383858
A0AAA9T820	AUP1	A0AAA9T820	ENSBTAG00000020123	0.044032768	-0.033166864	0.077199632
F1MFA7	CCDC186	F1MFA7	ENSBTAG00000020233	0.240039604	0.079574932	0.160464672
A0AAA9T7X2	CDC16	A0AAA9T7X2	ENSBTAG00000019971	0.099246237	0.032903742	0.066342495
A0AAF6YYP8	GLTPD2	A0AAF6YYP8	ENSBTAG00000048034	0.043439261	0.176211264	-0.132772003
A0AAA9RY43	LACTB2	A0AAA9RY43	ENSBTAG00000001808	0.327164743	0.322976946	0.004187797
Q2TA08	BAG5	bta:522854; Q2TA08	ENSBTAG00000058752	-0.0489096	0.906463067	-0.955372668
A4FUB8	TMEM115	A4FUB8; bta:532459		0.175428942	0.04548277	0.129946173
F1MJX8	DNAJC1	F1MJX8	ENSBTAG00000033315	0.1379421	0.161141716	-0.023199616
Q3MHG6	GTPBP10	bta:613957; Q3MHG6		-0.14338608	-0.070682887	-0.072703194
A0AAA9TT54	ST3GAL4	A0AAA9TT54	ENSBTAG00000016506	-0.234345224	-0.045606227	-0.188738997
A0AAA9RT25	PTPRK	A0AAA9RT25	ENSBTAG00000020829	0.133772051	0.480317257	-0.346545207
A0AAF6Z0X4	RABEPK	A0AAF6Z0X4	ENSBTAG00000015098	-0.37739585	0.134456112	-0.511851962
Q3SZ45	SDF2	bta:508463; Q3SZ45		0.330071268	0.326005427	0.004065841
A0AAA9SHM7	MED15	A0AAA9SHM7	ENSBTAG00000018048	-0.085025381	0.037051216	-0.122076596
A0AAA9U0U6	ACADL	A0AAA9U0U6	ENSBTAG00000010579	-0.143422956	0.145951842	-0.289374798
A0A3Q1LUC4	INPP4A	A0A3Q1LUC4	ENSBTAG00000004814	-0.113875113	0.197446064	-0.311321177
A7YY65	MTCH1	A7YY65; bta:781257	ENSBTAG00000033453	-0.305576175	-0.337621902	0.032045727
F1MB46	TBC1D10A	F1MB46	ENSBTAG00000018086	-0.038857976	0.097451538	-0.136309514
Q2HJ98	FAHD1	bta:509273; Q2HJ98	ENSBTAG00000033015	0.053335268	0.20740378	-0.154068513
Q3SYZ9	MED4	bta:515299; Q3SYZ9	ENSBTAG00000019502	-0.104568943	-0.054090498	-0.050478445
A0AAA9TZ37	SERPING1	A0AAA9TZ37	ENSBTAG00000016267	0.163975735	0.52007297	-0.356097235
A0A3Q1NE41	ARPP19	A0A3Q1NE41	ENSBTAG00000011022	0.214890799	0.346789198	-0.131898399
Q0P584	ZMAT2	bta:782132; Q0P584	ENSBTAG00000005441	0.35444752	0.184356758	0.170090762
A0A3Q1MPE1	ZNF451	A0A3Q1MPE1	ENSBTAG00000018879	-0.006191834	-0.097440896	0.091249062
A4FUE6	MOBKL3	A4FUE6; bta:781884	ENSBTAG00000019116	0.069011615	0.243354247	-0.174342632
A0A3Q1MCI2	LRCH1	A0A3Q1MCI2	ENSBTAG00000002959	-0.05078811	0.063604238	-0.114392348
A0AAF6Z7J4	VPS25	A0AAF6Z7J4	ENSBTAG00000019906	-0.087116414	0.013792601	-0.100909015

Accession	Gene_Symbol	Gene_ID	Ensembl_Gene ID	log2(ratio(OV24H/OV0H))	log2(ratio(OV24R/OV0H))	log2(ratio(OV24H/OV24R))
Q3ZBG7	SRP19	bta:514960; Q3ZBG7	ENSBTAG00000012803	0.094443204	0.101567639	-0.007124434
A5D7H7	NOP9	A5D7H7; bta:528833; F1MK13	ENSBTAG00000010235	-0.09213822	-0.163790215	0.071651996
A0A3Q1M7I4	NR3C1	A0A3Q1M7I4	ENSBTAG00000019472	-0.016551176	0.025823775	-0.042374952
A0A3Q1MY50	ACBD5	A0A3Q1MY50	ENSBTAG00000017278	0.178304055	0.040800531	0.137503524
A0AAA9T7H1	PTAR1	A0AAA9T7H1	ENSBTAG00000005826	-0.085729874	-0.13458604	0.048856166
A6QQA9	ACOT2	A6QQA9; bta:511033	ENSBTAG00000046814	0.124305663	0.015833874	0.108471789
A0AAF6ZBD7	SRSF7	A0AAF6ZBD7	ENSBTAG00000014891	-0.055664175	-0.162579379	0.106915204
A0AAA9SNW6	RBM5	A0AAA9SNW6	ENSBTAG00000006330	0.127213325	-0.016933235	0.14414656
A0AAF6YIL8	RPLP2	A0AAF6YIL8	ENSBTAG00000001777	0.427330775	0.070557288	0.356773487
A0A3Q1N184	NHSL1	A0A3Q1N184	ENSBTAG00000000245	0.052188277	0.026330109	0.025858168
A0A3Q1ME54	PARP4	A0A3Q1ME54	ENSBTAG00000025400	-0.154328146	-0.160991877	0.00666373
F6QDZ3	USE1	F6QDZ3	ENSBTAG00000011134	-0.052977476	0.227410496	-0.280387973
A0AAA9SJM0	NSA2	A0AAA9SJM0	ENSBTAG00000003066	-0.223975192	-0.155925513	-0.068049679
Q3ZBE1	ARMC1	bta:514000; Q3ZBE1; Q5EAC8	ENSBTAG00000015901	0.037224011	-0.015445058	0.052669069
A0A3Q1MMP5	TSC2	A0A3Q1MMP5	ENSBTAG00000020617	0.00140957	0.203533394	-0.202123824
E1BBZ8	FOSL2	bta:509889; E1BBZ8	ENSBTAG00000023929	-0.126263773	-0.335762956	0.209499184
Q08DT8	RINT1	bta:510686; Q08DT8		-0.231866388	-0.103093493	-0.128772895
A0AAA9SLU6	TEAD3	A0AAA9SLU6	ENSBTAG00000019495	0.174732845	0.22195008	-0.047217236
F1N1U1	SYNJ2	F1N1U1	ENSBTAG00000000817	-0.305254941	-0.07481948	-0.230435461
A6QQR1	NSUN5	A6QQR1; bta:616714; E1BGI7	ENSBTAG00000017941	-0.014630272	0.179239678	-0.19386995
Q2KHT7	RPS27	A1XEE0; bta:615638; Q2KHT7		-0.062076496	-0.161296101	0.099219605
E1BPU3	PEX1	E1BPU3	ENSBTAG00000024431	-0.113829741	-0.018581426	-0.095248315
Q08DI5	RAP2C	bta:515181; Q08DI5	ENSBTAG00000004131	0.123406975	0.134367674	-0.010960699
A0AAA9SJ23	TRMT10A	A0AAA9SJ23	ENSBTAG00000000197	0.137812816	0.287494598	-0.149681782
A0A3Q1ML70	ATP7A	A0A3Q1ML70	ENSBTAG00000020018	-0.210922373	-0.165268639	-0.045653734
A0AAA9S661	KIFAP3	A0AAA9S661	ENSBTAG00000017727	-0.179612514	-0.161928594	-0.01768392
A6H7J3	GAN	A6H7J3; bta:521175	ENSBTAG00000009428	-0.031681006	0.00874742	-0.040428426
A0A3Q1MKU4	IL1RAP	A0A3Q1MKU4	ENSBTAG00000013205	-0.064968924	-0.04580369	-0.019165234
A0AAA9T4Z5				-0.076317908	0.176819414	-0.253137321
A0A3Q1LYV0	NHLRC3	A0A3Q1LYV0	ENSBTAG00000010951	0.415473556	0.446528281	-0.031054725
A0AAA9SX05	ANO10	A0AAA9SX05	ENSBTAG00000008893	0.061575279	0.057551023	0.004024257
A0AAA9RV38	PREB	A0AAA9RV38	ENSBTAG00000007080	0.14076238	0.095798492	0.044963888
F1MDX7	FHIP2B	F1MDX7	ENSBTAG00000014361	0.053859311	0.115143916	-0.061284605
A0A3Q1NIX3	CASC3	A0A3Q1NIX3	ENSBTAG00000006250	-0.072781317	-0.023363993	-0.049417324
A0A3Q1M9K0	TTC39B	A0A3Q1M9K0	ENSBTAG00000003943	-0.158463142	-0.007586494	-0.150876648
A7Z069	KRIT1	A7Z069	ENSBTAG00000002750	-0.30204623	0.227733355	-0.529779585
A0A3Q1N041	GPCPD1	A0A3Q1N041; bta:518469	ENSBTAG00000008293	-0.14839184	0.401805243	-0.550197083
Q3ZBV4	HMGN3	bta:515652; Q3ZBV4	ENSBTAG00000055765	0.233147907	0.129152154	0.103995753
F1MC22	CFAP298	F1MC22	ENSBTAG00000003059	-0.418763791	-0.307118435	-0.111645356
A0AAA9SD49	IRF2BP1	A0AAA9SD49	ENSBTAG00000050077	0.119037869	0.026662474	0.092375395
A0AAA9THQ4	SOAT1	A0AAA9THQ4	ENSBTAG00000004059	0.092534969	0.101127579	-0.008592611
A6QLZ7	CRISPLD2	A6QLZ7; bta:505329		-0.260062839	0.16163839	-0.42170123
A5PJT3	PRMT9	A5PJT3; bta:532021	ENSBTAG00000010381	0.045371508	0.030405657	0.01496585
F6QBF9	RBM8A	F6QBF9	ENSBTAG00000008369	-0.328749361	-0.257058255	-0.071691106
A0A3Q1LXL8	MTX1	A0A3Q1LXL8	ENSBTAG00000014840	-0.008841877	-0.101951281	0.093109404
F1MFG7	BPHL	F1MFG7	ENSBTAG00000006379	-0.246721751	-0.204410946	-0.042310805
A6H7J2	RPS19BP1	A6H7J2; bta:509108	ENSBTAG00000017463	0.149187755	0.105259788	0.043927967
A0AAA9S7V3	NDUFB9	A0AAA9S7V3	ENSBTAG00000020405	-0.151331135	-0.092101584	-0.059229551
F1MX05	SCCPDH	F1MX05	ENSBTAG00000003804	-0.041960532	0.390574259	-0.432534791
Q3SZI0	MPI	bta:513586; Q3SZI0	ENSBTAG00000005845	0.155205525	0.186844843	-0.031639317
E1B9G4	PPIL3	bta:615703; E1B9G4	ENSBTAG00000005416	0.041988068	-0.01328322	0.055271287
A0AAA9S0E0	LYPLA2	A0AAA9S0E0	ENSBTAG00000011625	0.131178919	0.031347944	0.099830975
A0AAF7AII2	DGKA	A0AAF7AII2	ENSBTAG00000004018	0.010935478	0.117405954	-0.106470477
A0AAA9TQR4	PEG10	A0AAA9TQR4	ENSBTAG00000059156	0.067992977	0.040050947	0.02794203
A0AAA9SIA3	XPNPEP3	A0AAA9SIA3; bta:539202	ENSBTAG00000006204	0.274299232	0.161824503	0.112474729
Q5E9M1	ARL6IP5	bta:509977; Q5E9M1	ENSBTAG00000021506	0.061879448	0.203382129	-0.141502681
A0A3Q1LWZ0	ORC4	A0A3Q1LWZ0	ENSBTAG00000015291	0.090860769	0.132806737	-0.041945968
A0AAA9TCD6	MICOS13	A0AAA9TCD6	ENSBTAG00000001792	0.092915897	-0.03446341	0.127379306
A0A3Q1MIF4	CFI	A0A3Q1MIF4	ENSBTAG00000034501	-0.055059018	0.496506273	-0.551565291
A0A452DK61	CISD2	A0A452DK61	ENSBTAG00000027630	0.304479124	0.22036379	0.084115335
A0A3Q1MET1	SLC44A2	A0A3Q1MET1	ENSBTAG00000002628	0.025355574	0.111931058	-0.086575484
F1MYS8	TOM1L1	F1MYS8	ENSBTAG00000007013	-0.183554342	-0.050671671	-0.132882671
A0AAA9TGJ7	CHUK	A0AAA9TGJ7	ENSBTAG00000007591	0.022911099	0.048094288	-0.02518319
F1MXI3	PI4KB	F1MXI3	ENSBTAG00000007320	-0.150636581	-0.103606269	-0.047030312
Q5E9I4	MGAT1	bta:534248; Q5E9I4	ENSBTAG00000001546	-0.092048209	0.062329133	-0.154377342
G3X782	CHKB	bta:536213; G3X782	ENSBTAG00000016050	-0.109697645	0.073166413	-0.182864057
F1MYH8	PSMD8	F1MYH8	ENSBTAG00000031641	0.109624491	0.13865354	-0.029029049
Q17QB3	ASAH1	bta:510620; Q17QB3	ENSBTAG00000011257	0.014398274	0.042770772	-0.028372498
A7YWA1	POLR1F	A7YWA1; bta:512375; F1MNK1	ENSBTAG00000006721	0.139302827	0.061146616	0.078156211
A0A3Q1LTB1	NFAT5	A0A3Q1LTB1	ENSBTAG00000013412	0.050521776	0.10496956	-0.054447784
Q08DX5	CDK7	bta:515462; F1N2N7; Q08DX5	ENSBTAG00000011046	0.021520807	-0.033748791	0.055269598
A0AAA9TXA0	C17H1orf43	A0AAA9TXA0	ENSBTAG00000011796	0.380089986	0.326028251	0.054061734
A0A3Q1MS44	DDX51	A0A3Q1MS44	ENSBTAG00000017219	-0.330587177	-0.166571566	-0.164015611
F6PY02	RDH13	F6PY02	ENSBTAG00000030393	0.096363677	0.234388594	-0.138024917
E1BPF3	BCL9	E1BPF3	ENSBTAG00000002422	-0.010003869	0.02120609	-0.031209959
F1MB54	EXOSC9	F1MB54	ENSBTAG00000004942	0.035441579	0.190213621	-0.154772043
Q5E9H5	BCS1L	bta:539713; Q5E9H5	ENSBTAG00000003813	0.129612738	0.055195654	0.074417084
A0AAA9T9U8	RFTN1	A0AAA9T9U8	ENSBTAG00000030593	-0.441455244	-0.323031495	-0.118423749
A0A3Q1M9C8	AKAP8	A0A3Q1M9C8	ENSBTAG00000001807	-0.295204411	-0.430333686	0.135129275
A0A3Q1NEI6	EIF4E	A0A3Q1NEI6	ENSBTAG00000009522	0.04930292	-0.026800059	0.076102979
E1BH93	MAST4	E1BH93	ENSBTAG00000025964	-0.100928909	-0.110611462	0.009682553
A0AAA9RZE5	GYS1	A0AAA9RZE5	ENSBTAG00000039958	-0.156158723	-0.006932714	-0.149226009
Q1ZYR0	CLN5	bta:529186; Q1ZYR0	ENSBTAG00000018846	0.097492962	0.188812618	-0.091319656
E1BIJ3	CASKIN2	E1BIJ3	ENSBTAG00000007220	0.04207252	0.06898661	-0.02691409
Q5E936	TXNDC12	bta:506991; Q5E936	ENSBTAG00000011024	0.556317714	0.328342599	0.227975115
F1N6Q0	DNAJA4	F1N6Q0	ENSBTAG00000015942	0.385192988	0.247054476	0.138138513
A0A3Q1MT43	ARHGAP42	A0A3Q1MT43; bta:508009	ENSBTAG00000004423	-0.219691377	-0.231545963	0.011854586
A0AAF6Z1E4	TOX4	A0AAF6Z1E4	ENSBTAG00000015146	-0.130116436	0.024963285	-0.155079721
O46375	TTR	bta:280948; O46375; Q3SZ91	ENSBTAG00000010991	0.156042373	0.344816701	-0.188774328
A0A3Q1LJP7	TMEM245	A0A3Q1LJP7; bta:521172	ENSBTAG00000038794	0.186356785	0.622882061	-0.436525275
A0A3Q1MCK1	HPS5	A0A3Q1MCK1; bta:510165	ENSBTAG00000005147	-0.128786307	-0.014646776	-0.114139531
Q3SYS0	MRPL24	bta:532203; Q3SYS0	ENSBTAG00000001520	-0.26742952	-0.177832083	-0.089597436
A0AAA9SFF8	RPL22	A0AAA9SFF8	ENSBTAG00000014423	0.077459489	0.052098962	0.025360527
A0AAA9SY74	COMMD9	A0AAA9SY74	ENSBTAG00000019045	0.785719008	0.220438873	0.565280135
Q08DW1	RAB9A	bta:511776; Q08DW1	ENSBTAG00000010923	0.034651547	0.068490295	-0.033838748
A4IFA7	CBR4	A4IFA7; bta:533020	ENSBTAG00000022013	-0.323697188	-0.0085115	-0.315185688
A0A3Q1MSQ6	ANKS1A	A0A3Q1MSQ6	ENSBTAG00000016890	0.017585811	0.056059959	-0.038474148
B0JYN1	CTSL2	B0JYN1		-0.802221168	-0.339249191	-0.462971976
A0AAF6DM48	MFN2	A0AAF6DM48	ENSBTAG00000005314	0	0.208802633	-0.208802633
F6RJ80	EML3	F6RJ80	ENSBTAG00000011872	-0.425860469	-0.327870678	-0.097989791
A0AAF6Z7A4	EIF1AD	A0AAF6Z7A4	ENSBTAG00000020186	0.166044416	0.131726959	0.034317456
A0AAA9SCT8	THOC7	A0AAA9SCT8	ENSBTAG00000032872	-0.143232884	0.516917113	-0.660149997
A0A3S5ZP75	PPCS	A0A3S5ZP75	ENSBTAG00000012982	0.188316793	0.215641379	-0.027324587
A0A452DIN1	GADD45GIP1	A0A452DIN1	ENSBTAG00000015117	-0.146530407	0.148806467	-0.295336874
Q3MHZ7	PIGK	bta:508700; Q3MHZ7	ENSBTAG00000031012	-0.004317295	0.010023728	-0.014341023
Q2T9Y6	GCLM	bta:525659; Q2T9Y6	ENSBTAG00000007842	0.055408695	0.074382196	-0.018973501
A0A3Q1MGX6	AKTIP	A0A3Q1MGX6; bta:540462	ENSBTAG00000016250	0.049777561	0.195897241	-0.14611968
P01096	ATP5IF1	bta:327699; P01096; Q2M2T4	ENSBTAG00000006342	0.006171967	0.169065692	-0.162893725
Q6IE76	FDFT1	bta:281767; F1MW61; Q6IE76	ENSBTAG00000012432	-0.230083984	-0.152179451	-0.077904533
A0AAA9SST7	ATG2B	A0AAA9SST7	ENSBTAG00000012833	-0.043977697	-0.085797873	0.041820176
Q0IIM2	ARL6	bta:519014; Q0IIM2	ENSBTAG00000010091	0.289753823	0.219086386	0.070667438
F1MYH0	ZNHIT6	F1MYH0	ENSBTAG00000021051	-0.161234041	0.063940821	-0.225174862
F6Q3R4	QTRT2	F6Q3R4	ENSBTAG00000004977	-0.041966165	0.008248708	-0.050214873
A0AAA9TCY6	ADPRH	A0AAA9TCY6	ENSBTAG00000009391	-0.296251347	-0.11189288	-0.184358467
A1A4M0	TIMM13	A1A4M0; bta:516325; F6RJY8		0.305365904	0.490022214	-0.184656311
F1MNG1	CYP1B1	F1MNG1	ENSBTAG00000010531	-0.169236857	-0.124530053	-0.044706804
A0A452DIV1	MRPL13	A0A452DIV1	ENSBTAG00000006767	-0.214515152	-0.187278568	-0.027236584
E1BM96	MAGI3	E1BM96	ENSBTAG00000019611	0.10031741	0.123574685	-0.023257275
F1MH25	AMZ2	F1MH25	ENSBTAG00000009345	-0.260651755	-0.349007629	0.088355874
Q5E982	DPH5	bta:508904; Q2HJ50; Q5E982	ENSBTAG00000019029	0.148863386	0.193733658	-0.044870272
A0A3Q1LYZ4	SFSWAP	A0A3Q1LYZ4	ENSBTAG00000000439	0.026835043	-0.014326781	0.041161824
F1N4A6	EMSY	F1N4A6	ENSBTAG00000010826	-0.185471045	-0.099741171	-0.085729874
A0A3Q1M442	TUBB1	A0A3Q1M442; bta:541271	ENSBTAG00000018785	-0.109097896	0.138261441	-0.247359337
Q1RMS1	TCIRG1	bta:513684; F1MGS2; Q1RMS1	ENSBTAG00000000292	-0.278374952	-0.11297471	-0.165400242
G3MWU7	TPRN	G3MWU7	ENSBTAG00000047715	-0.2051848	-0.139531561	-0.065653239
E1BEZ8	PTPN14	E1BEZ8	ENSBTAG00000021553	-0.277007348	-0.346519391	0.069512043
E1B789	EDEM3	E1B789	ENSBTAG00000019264	-0.188976689	-0.126830022	-0.062146667
E1B9Q8	ZNF609	bta:510374; E1B9Q8	ENSBTAG00000015808	0.066224045	0.020930658	0.045293387
Q0P5A1	DCTN3	bta:514327; Q0P5A1		0.166764661	0.158303082	0.008461579
A0A3Q1NJJ1	ARK2N	A0A3Q1NJJ1	ENSBTAG00000032964	-0.035297052	-0.079132961	0.043835909
Q1RMT8	IRAK4	bta:533692; Q1RMT8	ENSBTAG00000021105	-0.427110332	-0.145339363	-0.281770968
Q08E24	SARAF	bta:515461; Q08E24	ENSBTAG00000013579	-0.030494029	-0.126045304	0.095551274
F1N0D6	JAK1	F1N0D6	ENSBTAG00000003147	-0.062025855	-0.027419289	-0.034606566
A0A3Q1MRR5	LIMCH1	A0A3Q1MRR5	ENSBTAG00000010677	0.078831477	0.172673231	-0.093841754
A2VDY0	CTHRC1	A2VDY0; bta:538634	ENSBTAG00000032591	-0.390727092	-0.831962108	0.441235016
Q0VD51	RNF13	bta:539035; Q0VD51	ENSBTAG00000011147	0.280489282	0.153623189	0.126866093
E1BL26	RNF123	bta:514160; E1BL26	ENSBTAG00000011588	-0.103287808	0.045188774	-0.148476582
F6PZI3	SLC29A1	bta:510932; F6PZI3	ENSBTAG00000015131	-0.133362096	-0.17122164	0.037859544
A0AAA9TSF3	MCRIP1	A0AAA9TSF3	ENSBTAG00000019812	-0.199009494	0.186739646	-0.38574914
E1BC57	ROR2	E1BC57	ENSBTAG00000005092	-0.011849718	0.172217854	-0.184067571
Q6TJY3	RPS6KB1	bta:404181; Q6TJY3		-0.190043644	-0.051377359	-0.138666284
A0AAA9T0P8	CEP97	A0AAA9T0P8	ENSBTAG00000013464	0.007145607	0.022742462	-0.015596855
A0A3Q1NJQ0	FAR1	A0A3Q1NJQ0	ENSBTAG00000009061	-0.428641721	-0.333109284	-0.095532437
A0AAA9SNI5	BSDC1	A0AAA9SNI5	ENSBTAG00000020655	0.537737235	0.305544	0.232193235
Q3ZBH8	RPS20	bta:513222; Q3ZBH8	ENSBTAG00000019147	-0.123176742	-0.180764234	0.057587492
P82917	MRPS18C	bta:613561; P82917; Q2KI03	ENSBTAG00000018155	-0.164001598	-0.289817008	0.125815409
A0A3Q1M1E9	GPD1L	A0A3Q1M1E9	ENSBTAG00000009826	0.135484356	0.131437523	0.004046833
Q5E9K0	PSMB2	bta:516919; Q5E9K0	ENSBTAG00000002377	0.2410081	0.129011451	0.111996649
Q08DC8	NUMBL	bta:510973; Q08DC8		-0.236376651	-0.240024428	0.003647777
A0A3Q1LWZ9	ERCC2	A0A3Q1LWZ9	ENSBTAG00000002072	0.145966704	0.194150237	-0.048183534
A0AAF6YQ96	GABARAPL2	A0AAF6YQ96	ENSBTAG00000006550	-0.060411517	-0.452432363	0.392020847
A0AAA9TWW3	MRPL48	A0AAA9TWW3	ENSBTAG00000008347	-0.110062776	0.109292145	-0.219354922
A0A3Q1LN51	ATF1	A0A3Q1LN51	ENSBTAG00000018131	0.142518844	0.051428239	0.091090605
F1MZ00	SNRPD3	F1MZ00	ENSBTAG00000021262	0.027674958	0.008796948	0.01887801
A0A3Q1LXK3	TTC17	A0A3Q1LXK3	ENSBTAG00000031299	0.09420617	0.123800952	-0.029594782
A0A3Q1M4U3	TRAPPC10	A0A3Q1M4U3	ENSBTAG00000007100	-0.156996487	-0.071393188	-0.085603299
A6QLV3	SHOC2	A6QLV3; bta:511417	ENSBTAG00000007709	-0.034591748	0.02120609	-0.055797838
Q2NKS3	PSMG3	bta:510741; Q2NKS3	ENSBTAG00000000050	0.105693961	-0.012874905	0.118568866
A0AAA9U299	TMEM30A	A0AAA9U299	ENSBTAG00000005100	-0.035845199	0.021959253	-0.057804452
E1BHL0	CNOT11	bta:506268; E1BHL0	ENSBTAG00000008267	0.167389827	0.253231231	-0.085841404
A0A3Q1LWG3	RPS6KC1	A0A3Q1LWG3	ENSBTAG00000001181	0.083141235	0.121577417	-0.038436182
A0AAA9TXN0	MAPK8	A0AAA9TXN0	ENSBTAG00000007876	-0.059474346	0	-0.059474346
A0A3Q1MCA2	DMWD	A0A3Q1MCA2; bta:790091	ENSBTAG00000021417	-0.367610067	-0.205096961	-0.162513107
A6H716	BRAP	A6H716; bta:518844	ENSBTAG00000001168	-0.134165238	-0.172887236	0.038721998
A2VDW3	XRCC4	A2VDW3; bta:613590; F1N695	ENSBTAG00000024275	-0.004553486	0.021061616	-0.025615102
F1MCG4	RALGAPB	F1MCG4	ENSBTAG00000003040	0.033838748	0.158989524	-0.125150775
F1MIX0	ABRAXAS2	F1MIX0	ENSBTAG00000023840	-0.059802187	-0.1015806	0.041778412
A0AAA9TRK4	PIN4	A0AAA9TRK4	ENSBTAG00000047376	0.313204002	-0.067744607	0.380948609
Q8HXK9	PYCARD	bta:282846; Q8HXK9		0.512562957	0.490635118	0.021927839
F1MZE4	SPAG7	F1MZE4	ENSBTAG00000008590	-0.17994081	-0.242608427	0.062667617
P57709	ATP2C1	bta:327663; P57709	ENSBTAG00000011626	0.076923977	0.142019005	-0.065095028
Q2TBQ3	GAMT	bta:515270; Q2TBQ3	ENSBTAG00000004112	-0.127592366	-0.048649163	-0.078943203
A0AAA9RV48	UTP4	A0AAA9RV48	ENSBTAG00000006347	-0.491095577	-0.515920856	0.024825279
Q2NL01	GPX8	bta:511575; Q2NL01	ENSBTAG00000021960	-0.203061694	-0.49323223	0.290170536
E1BG08	AIDA	bta:508353; E1BG08	ENSBTAG00000007593	-0.06094935	0.07827562	-0.13922497
F1N3I6	WWC1	F1N3I6	ENSBTAG00000013880	-0.412540561	-0.494667327	0.082126767
A0AAA9SRE4	PPIC	A0AAA9SRE4	ENSBTAG00000001568	0.023742089	0.089714121	-0.065972032
A0AAA9TKA1	SNX12	A0AAA9TKA1	ENSBTAG00000007871	-0.044825199	-0.079382421	0.034557222
E1B9S8	CBFB	E1B9S8	ENSBTAG00000016103	0.164241363	0.093345737	0.070895626
A0A3Q1ND53	HAGH	A0A3Q1ND53	ENSBTAG00000019998	0.098915026	0.173908515	-0.074993489
E1BIR9	SARM1	E1BIR9	ENSBTAG00000002816	-0.293692063	-0.190393437	-0.103298625
A2VDZ0	PPP2R5A	A2VDZ0; bta:533788	ENSBTAG00000000754	0.102810806	0.146418249	-0.043607443
Q2KID4	DNAL1	bta:538164; Q2KID4	ENSBTAG00000015930	0.124684975	0.105948412	0.018736563
A0AAA9S3M6	RARS2	A0AAA9S3M6	ENSBTAG00000016967	-0.318880504	-0.183377198	-0.135503306
A0AAA9SL62	GDAP2	A0AAA9SL62	ENSBTAG00000016387	0.048295557	0.031875674	0.016419883
A0AAA9TIV3	BUD31	A0AAA9TIV3	ENSBTAG00000020439	0.221245696	0.166533154	0.054712542
A5PJC3	NARS2	A5PJC3; bta:504824; F6QRZ3		0.068425141	0.077568553	-0.009143412
Q3MQ24	ATG5	bta:532686; Q3MQ24; Q3SZN7	ENSBTAG00000005400	-0.034107854	-0.025848127	-0.008259727
A0AAA9SFQ4	LRCH4	A0AAA9SFQ4	ENSBTAG00000003160	-0.191213122	-0.105483248	-0.085729874

Accession	Gene_Symbol	Gene_ID	Ensembl_Gene ID	log2(ratio(OV24H/OV0H))	log2(ratio(OV24R/OV0H))	log2(ratio(OV24H/OV24R))
A0AAA9S5S6	EIPR1	A0AAA9S5S6	ENSBTAG00000018387	-0.022855053	-0.113470933	0.09061588
Q5EA98	MFAP1	bta:510905; Q5EA98	ENSBTAG00000017213	0.006617879	-0.099535674	0.106153552
Q3T093	NECAP1	A5PKA6; bta:504449; Q3T093	ENSBTAG00000020237	0.019802008	0.037949354	-0.018147347
Q58D63	MOB3A	bta:505007; Q08E56; Q58D63		-0.007784345	-0.062132063	0.054347718
E1BA19				0.305249354	0.032789935	0.272459419
E1BDK3	NAA30	E1BDK3	ENSBTAG00000012998	-0.166792112	-0.301169535	0.134377423
A0A3Q1LL79	NPTN	A0A3Q1LL79	ENSBTAG00000008218	0	0.124912946	-0.124912946
F6QEM8	GSTM5	F6QEM8	ENSBTAG00000037673	0.174420019	0.207759964	-0.033339945
F1MMX8	MED8	F1MMX8	ENSBTAG00000016899	-0.007425107	-0.002965458	-0.004459648
F1MYW0	NT5DC3	bta:520927; F1MYW0	ENSBTAG00000006013	-0.286999991	-0.308914767	0.021914776
P61285	DYNLL1	bta:101902172; bta:404151; bta:784058; P61285; Q6B859	ENSBTAG00000024605	0.259285057	0.082640458	0.176644599
A0AAF7A6E5	PQBP1	A0AAF7A6E5	ENSBTAG00000018498	0.165604776	0.124883672	0.040721103
A0AAF7ADA8	BCAT2	A0AAF7ADA8	ENSBTAG00000009172	-0.157012456	-0.036718223	-0.120294234
A0A3Q1MMC0	EFNB1	A0A3Q1MMC0	ENSBTAG00000015801	-0.065687903	-0.161388319	0.095700416
A0AAA9U136	PRKD2	A0AAA9U136	ENSBTAG00000015599	0.050715065	0.094882648	-0.044167583
A0AAA9SV20	H1-0	A0AAA9SV20	ENSBTAG00000067495	-0.118578121	0.494689748	-0.61326787
Q3SWX1	CNPY4	bta:505637; Q3SWX1		-0.066981577	-0.134165238	0.067183661
A0A3Q1M294	KMO	A0A3Q1M294; bta:515996	ENSBTAG00000014439	-0.498218159	-0.568915524	0.070697366
A4IFA8				-0.317255935	-0.153638212	-0.163617722
F1MHW3	TRIR	F1MHW3	ENSBTAG00000011825	0.258918297	0.039528364	0.219389933
P82669	MRPS25	bta:533011; P82669; Q32P88	ENSBTAG00000013723	-0.159671023	-0.15065415	-0.009016873
A0A452DJ65	TK1	A0A452DJ65	ENSBTAG00000007121	-0.529032473	-0.613762211	0.084729739
Q2KHY9	PTDSS1	bta:509819; F1MKI4; Q2KHY9	ENSBTAG00000013901	0.027757701	-0.017454876	0.045212577
A0AAA9TH89	CACNA2D1	A0AAA9TH89	ENSBTAG00000020569	-0.229481846	-0.136006693	-0.093475153
A0AAF6ZE24	MTFR1L	A0AAF6ZE24	ENSBTAG00000021781	0.033520076	-0.031426283	0.064946359
F1MWH8	ABL2	F1MWH8	ENSBTAG00000015026	0.092134281	0.065553682	0.026580599
F1MDA8	DCLK1	F1MDA8	ENSBTAG00000038495	0.034718936	0.046108693	-0.011389757
E1BD42	NUP37	E1BD42	ENSBTAG00000000022	0.010818332	0.104697379	-0.093879047
A2VDV0	NUDT4	A2VDV0; bta:614183	ENSBTAG00000007447	-0.189142917	-0.309031652	0.119888736
A0AAA9RZF6				0.203706545	0.180669009	0.023037536
A5D7R0	FAS	A5D7R0	ENSBTAG00000010785	-0.652842357	-0.447093691	-0.205748665
A7Z088	YEATS2	A7Z088; bta:514192; F1N223	ENSBTAG00000014451	-0.243557031	-0.057143907	-0.186413124
Q2KJD7	UBLCP1	bta:508163; Q2KJD7	ENSBTAG00000009103	-0.730445361	-0.530642859	-0.199802501
Q08DK4	SLC25A22	bta:504371; Q08DK4	ENSBTAG00000001229	0.054614927	0.01794048	0.036674447
Q3SZD1	PEX19	bta:521522; Q3SZD1	ENSBTAG00000007537	0.531547373	0.050890666	0.480656707
Q17QG8	H2AFX	bta:531733; Q17QG8	ENSBTAG00000067452	-0.163614384	0.028625841	-0.192240225
Q3ZCD7	TECR	bta:614105; Q3ZCD7	ENSBTAG00000012632	-0.159443518	0.084316286	-0.243759804
A6QQ09	LOC100138230	A6QQ09; bta:100138230		0.248283954	0.27931085	-0.031026896
F1MJN1	ZNF318	F1MJN1	ENSBTAG00000010217	0.157589528	0.026344995	0.131244533
A0AAF6Z211	NOL11	A0AAF6Z211	ENSBTAG00000015951	-0.121105034	-0.110228445	-0.010876589
A0AAA9TJ50	FOXK2	A0AAA9TJ50	ENSBTAG00000003687	-0.213026578	-0.246934015	0.033907437
A0AAA9TY59	GLT8D1	A0AAA9TY59	ENSBTAG00000014550	-0.397367227	-0.382028328	-0.015338899
A0A3Q1MC91	SAMD4B	A0A3Q1MC91	ENSBTAG00000009785	-0.165737204	-0.08789977	-0.077837434
G3N0L9				-0.16851818	-0.05450192	-0.11401626
G3X6X2	NT5C	G3X6X2	ENSBTAG00000011527	-0.547002839	-0.393342428	-0.153660411
E1BFG7	PHC2	E1BFG7	ENSBTAG00000004603	0.009491449	-0.006362502	0.015853951
F1MKY4	ZFYVE9	F1MKY4; G3MXR1	ENSBTAG00000014289	0.114433173	0.182433273	-0.0680001
A0AAA9TKC2	PLA2G15	A0AAA9TKC2	ENSBTAG00000007512	0.279742865	0.488747185	-0.209004321
E1BG57				-0.236712093	-0.218834602	-0.017877491
A0A3Q1M7U1	KLHL11	A0A3Q1M7U1; bta:538695	ENSBTAG00000050549	-0.362570079	-0.442209228	0.079639149
A0AAF6ZB27	IAH1	A0AAF6ZB27	ENSBTAG00000001140	-0.138995024	0.244698347	-0.383693371
A6QNX2	DPP7	A6QNX2; bta:100125760; H9GW42		-0.03991901	0.157056321	-0.196975331
A0AAA9S8H4	ADI1	A0AAA9S8H4	ENSBTAG00000005403	0.340424439	0.30991113	0.030513308
Q3SZ11	RPA3	bta:512754; Q3SZ11	ENSBTAG00000013168	0.455236328	0.117384643	0.337851684
F6PU96	CHSY1	bta:281690; F6PU96	ENSBTAG00000007357	-0.004758757	-0.041773599	0.037014842
A0AAF6DMC7	RXRB	A0AAF6DMC7	ENSBTAG00000000602	0.158666824	0.147990031	0.010676793
A0AAA9RVB5	ARFIP2	A0AAA9RVB5	ENSBTAG00000003566	0.042973686	0.020850875	0.022122811
A0AAF7APV1	AMDHD2	A0AAF7APV1	ENSBTAG00000001022	-0.174996606	-0.122360262	-0.052636344
E1BKM6	TAOK2	bta:533897; E1BKM6	ENSBTAG00000017422	-0.296187659	-0.224344648	-0.071843011
A4FUB5	COMMD10	A4FUB5; bta:538706	ENSBTAG00000019071	0.262256882	0.10057172	0.161685162
A0A3Q1LYP9	TMCC3	A0A3Q1LYP9	ENSBTAG00000014375	0.174349252	0.227410496	-0.053061244
A0A3Q1M7M3	MRPS23	A0A3Q1M7M3	ENSBTAG00000012366	-0.349607409	-0.351423262	0.001815853
E1BKZ0	PCNT	E1BKZ0	ENSBTAG00000030670	0.045894766	0.01161129	0.034283476
F1MBJ5	SMG9	F1MBJ5	ENSBTAG00000011815	0.152874549	-0.03678667	0.18966122
F1N5R4	COG8	F1N5R4	ENSBTAG00000001665	-0.112964249	0.020974129	-0.133938378
A4IFF2	WDR46	A4IFF2		0.082064537	-0.179618398	0.261682935
A0A3Q1LR40	LIPG	A0A3Q1LR40	ENSBTAG00000048916	0.162157314	0.06125156	0.100905754
F1MSD6	TRAPPC5	F1MSD6	ENSBTAG00000004268	0.015689374	-0.054061734	0.069751108
Q05B63	TRMT11	bta:514867; Q05B63		-0.362570079	-0.124736228	-0.237833852
E1B9H1	TINAGL1	E1B9H1	ENSBTAG00000013235	0.065047297	0.233247618	-0.168200321
Q3ZBI6	FHL3	bta:504795; Q3ZBI6	ENSBTAG00000007300	0.129559634	0.208493518	-0.078933884
Q02368	NDUFB7	bta:338065; Q02368; Q3T0A1	ENSBTAG00000012634	0.017866421	0.055834271	-0.03796785
A0A452DJJ7	MRPL28	A0A452DJJ7	ENSBTAG00000016581	-0.174859707	-0.117237675	-0.057622032
A0AAA9SGW9	ATXN3	A0AAA9SGW9	ENSBTAG00000005786	0.046459311	0.265946001	-0.21948669
A0A3Q1MCA3	CBX8	A0A3Q1MCA3	ENSBTAG00000009107	0.015282929	0.037907989	-0.022625059
A0AAA9T1E3	IGFBP2	A0AAA9T1E3	ENSBTAG00000005596	0.151099008	0.013493739	0.137605269
P35604	COPZ1	bta:281707; P35604; Q5EA44		0.921407163	0.427203286	0.494203877
E1BNH5	MTMR12	E1BNH5	ENSBTAG00000012278	-0.332227848	-0.292899046	-0.039328802
Q3SX43	RRAGA	bta:618207; Q3SX43	ENSBTAG00000033543	0.172142564	0.358638329	-0.186495765
A4FUD7	RAB33B	A4FUD7; bta:519982	ENSBTAG00000003208	-0.147726628	-0.123529918	-0.02419671
A0AAA9SHJ5	AP2S1	A0AAA9SHJ5	ENSBTAG00000010584	-0.030867567	0.045689161	-0.076556728
A0AAA9SL48				0.081318888	0.024476092	0.056842796
Q32KX0	ISOC2	bta:516212; Q32KX0		-0.083015902	-0.060407094	-0.022608808
A2VDP5	ENPP4	A2VDP5; bta:538583	ENSBTAG00000003499	-0.064652958	0.103935596	-0.168588554
A5PJS8	INTS8	A5PJS8; bta:100124522; F1MI91	ENSBTAG00000004902	-0.043403124	0.138004982	-0.181408106
A0A452DID6	DHRS11	A0A452DID6	ENSBTAG00000010297	-0.159023521	-0.055930028	-0.103093493
A0A452DIU4	CLEC3B	A0A452DIU4	ENSBTAG00000018331	0.088556809	0.30388779	-0.21533098
A0AAA9T613	LXN	A0AAA9T613	ENSBTAG00000009336	-0.067554243	-0.034733225	-0.032821018
A0AAA9TPF0	IKBKG	A0AAA9TPF0	ENSBTAG00000006268	0.212498513	0.20375488	0.008743633
F1MC26	TBC1D2B	F1MC26	ENSBTAG00000012022	0.095119196	0.093444564	0.001674632
A0AAA9SD33	MGA	A0AAA9SD33	ENSBTAG00000015138	-0.266828834	-0.28155869	0.014729856
E1BCW0	HGFAC	E1BCW0	ENSBTAG00000017335	-0.138734492	0.125146317	-0.26388081
A0AAA9TXU3	GSTZ1	A0AAA9TXU3	ENSBTAG00000002706	-0.138991605	0.021473067	-0.160464672
Q3T0D2	SCAMP1	bta:535352; Q3T0D2	ENSBTAG00000007371	0.233313708	0.315775868	-0.08246216
B2XBK5	CDYL	B2XBK5		-0.250565494	-0.227940435	-0.022625059
G3N258	TNRC6A	G3N258	ENSBTAG00000017999	-0.03970971	-0.119017575	0.079307865
A0AAA9SK06				0.151528445	0.081495118	0.070033327
A0AAA9SAM7	DENND5A	A0AAA9SAM7	ENSBTAG00000006322	-0.179477448	0.049904906	-0.229382353
A0AAA9T4U1	MBD3	A0AAA9T4U1	ENSBTAG00000038409	-0.156260252	-0.213779291	0.057519039
G3MYW2	NKRF	G3MYW2	ENSBTAG00000045744	-0.073291117	-0.014908207	-0.05838291
F1N420	NPM3	bta:504950; F1N420	ENSBTAG00000016335	-0.321091507	-0.396927345	0.075835838
Q2KJ63	KLKB1	bta:533547; Q2KJ63	ENSBTAG00000009501	-0.071397917	0.053972443	-0.12537036
P48305	NDUFB4	bta:327706; P48305; Q54A31	ENSBTAG00000015892	-0.003553438	0.13863233	-0.142185768
Q9BE40	MYH1	bta:281337; Q9BE40	ENSBTAG00000018204	-0.189072348	0.321542811	-0.510615159
F1N059	MRPS18B	F1N059	ENSBTAG00000006936	-0.339808868	-0.25568174	-0.084127129
A0AAA9TYA9	CERS2	A0AAA9TYA9	ENSBTAG00000000099	0.042871807	-0.111299123	0.15417093
A0A452DKN5	RGN	A0A452DKN5	ENSBTAG00000046155	0.208701262	0.244367112	-0.035665849
A0AAA9TDI5	SIPA1	A0AAA9TDI5	ENSBTAG00000013884	-0.195150482	-0.139403057	-0.055747426
Q29RR1	LRRC41	bta:337889; Q29RR1		-0.593416996	-0.215184937	-0.378232058
A0A3Q1MZU1	CTDSPL2	A0A3Q1MZU1; bta:540232	ENSBTAG00000015385	-0.217085729	-0.198664532	-0.018421197
A6QLB1	RAB27A	A6QLB1; bta:618035	ENSBTAG00000012408	-0.062449854	-0.185082685	0.122632831
Q3ZBK6	LSM4	bta:613634; Q3ZBK6	ENSBTAG00000008578	0.109048787	0.13076563	-0.021716843
A0A3Q1MTG5	SNX16	A0A3Q1MTG5	ENSBTAG00000019639	0.006231954	0.204888674	-0.19865672
A0AAA9TBE0	LRSAM1	A0AAA9TBE0	ENSBTAG00000012844	-0.370668206	-0.212798234	-0.157869972
A0A3Q1M4E5	FCSK	A0A3Q1M4E5	ENSBTAG00000012985	-0.085625471	0.212492073	-0.298117544
E1BEB4	FBLN2	bta:511854; E1BEB4	ENSBTAG00000004014	0.096491209	0.629874923	-0.533383713
A0A3Q1LPP6	MLKL	A0A3Q1LPP6	ENSBTAG00000012216	0.019557846	0.020852324	-0.001294477
Q2YDF6	MRPS35	bta:513438; P82672; Q2YDF6	ENSBTAG00000006369	-0.421490301	-0.12566596	-0.295824341
A0A452DIK3	NDUFS8	A0A452DIK3	ENSBTAG00000012072	-0.179168846	-0.245391113	0.066222267
A5PJQ7	PXK	A5PJQ7; bta:614093		-0.428172901	-0.38200272	-0.046170181
Q1JQE2	SLC7A6OS	bta:512543; Q1JQE2	ENSBTAG00000009636	0.214594051	0.087462841	0.12713121
Q32LE2	GKAP1	bta:613960; Q32LE2	ENSBTAG00000023523	0.205896101	0.504554417	-0.298658316
A0A3Q1MKH2	AKAP17A	A0A3Q1MKH2	ENSBTAG00000054242	0.280852152	0.253246211	0.027605941
A0A3Q1ML38	LENG8	A0A3Q1ML38	ENSBTAG00000011689	0.058005603	0.053735166	0.004270437
Q1RMS6	INTS7	bta:534964; Q1RMS6	ENSBTAG00000018548	-0.065318338	-0.044212705	-0.021105633
E1BIS5	RCC1L	bta:513273; E1BIS5	ENSBTAG00000011753	-0.033748791	-0.030906042	-0.00284275
Q32PI6	MRPL4	bta:507154; Q32PI6	ENSBTAG00000001419	-0.131244533	-0.042435266	-0.088809267
A0AAA9SFS1				0.380958944	0.084433938	0.296525006
F6QH10	SMOC1	F6QH10	ENSBTAG00000030599	-0.106915204	0.093586407	-0.200501611
Q3MHX0	SUOX	bta:509837; F6RUR3; Q3MHX0	ENSBTAG00000006160	-0.085566856	-0.043795118	-0.041771738
A0A3Q1N5C0	USP3	A0A3Q1N5C0	ENSBTAG00000008805	-0.14588261	-0.060946551	-0.084936059
A0A3Q1LZG8	PKD2	A0A3Q1LZG8	ENSBTAG00000020031	0.017505109	0.139769921	-0.122264812
A0AAA9TP18	MRPS16	A0AAA9TP18	ENSBTAG00000000950	-0.236677846	-0.302900113	0.066222267
A0AAA9SCL2	POLR3E	A0AAA9SCL2	ENSBTAG00000018044	0.119888736	0.135351853	-0.015463117
Q05B45	TMEM120A	bta:520173; Q05B45		0.092723192	0.072300475	0.020422718
A4FV59	PHF5A	A4FV59; bta:507180	ENSBTAG00000006423	0.188479048	0.12580819	0.062670858
Q02380	NDUFB5	bta:338061; Q02380; Q3SZV4; Q56K08	ENSBTAG00000002463	-0.469698716	-0.39665897	-0.073039746
Q3MHR2	DCK	bta:530642; Q3MHR2	ENSBTAG00000012397	-0.079617168	-0.322619869	0.243002701
A5PKB4	TIMP3	A5PKB4; F6RQF5	ENSBTAG00000020638	0.086654382	0.225333697	-0.138679314
F1MTJ9	LSS	F1MTJ9	ENSBTAG00000018936	-0.244355853	-0.116272122	-0.128083731
A0AAF6YJP2				0.036144564	-0.071915182	0.108059746
F1MRW6	PLEKHM2	F1MRW6	ENSBTAG00000018722	-0.031194622	0.101762716	-0.132957338
Q2TBQ1	F13B	bta:511483; Q2TBQ1	ENSBTAG00000000070	-0.070812468	-0.007068584	-0.063743884
Q3T0N1	MVB12A	bta:507199; Q3T0N1	ENSBTAG00000003914	-0.161333853	-0.090158101	-0.071175752
P32871	PIK3CA	bta:282306; P32871	ENSBTAG00000009232	-0.037192909	0.090429593	-0.127622503
Q2YDL5	FKBP11	bta:506043; Q2YDL5		0.00805527	-0.009726071	0.01778134
F1MW69	NELFCD	F1MW69	ENSBTAG00000018783	-0.334296652	0.025115248	-0.3594119
A4FUG9	BXDC5	A4FUG9; bta:513081		-0.145197916	-0.167433134	0.022235218
Q17QZ4	TFDP1	bta:534579; Q17QZ4	ENSBTAG00000019645	-0.145489786	-0.095810184	-0.049679603
Q3T132	LAMTOR2	bta:616375; Q3T132	ENSBTAG00000011754	0.273076407	0.126929791	0.146146616
A0A140T8A2	DPH2	A0A140T8A2; bta:768224	ENSBTAG00000018888	-0.165549625	-0.146023002	-0.019526623
A0AAA9TH24	MRPL10	A0AAA9TH24	ENSBTAG00000001832	-0.16539753	-0.137503524	-0.027894006
E1BN13	PDCD2L	bta:507032; E1BN13	ENSBTAG00000002571	-0.146584813	-0.121356716	-0.025228097
A0AAA9SDT0	STX18	A0AAA9SDT0	ENSBTAG00000005712	0.033243292	0.103789103	-0.070545811
Q58CS7	CHMP3	bta:507722; Q29RQ4; Q58CS7	ENSBTAG00000013589	0.137362642	0.152777695	-0.015415052
Q32L99	PTGR2	bta:506263; Q32L99	ENSBTAG00000003747	0.343013389	0.258124492	0.084888898
A0AAF6ZG02	GCDH	A0AAF6ZG02	ENSBTAG00000016211	-0.107106555	-0.087151949	-0.019954606
A0AAA9SAB7	TMEM131	A0AAA9SAB7; bta:540861	ENSBTAG00000006744	0.11501666	0.273224903	-0.158208243
A6QNS7	PDE4DIP	A6QNS7		-0.033774556	0.038665109	-0.072439665
A6QQ47	VPS51	A6QQ47; bta:525567		-0.536511335	-0.339509852	-0.197001483
F1MS32	APOD	F1MS32	ENSBTAG00000023600	-0.11724631	-0.173192715	0.055946405
E1B9A2	CARD6	E1B9A2	ENSBTAG00000014374	-0.024247546	-0.119372089	0.095124543
A0A452DJH7	MRPS10	A0A452DJH7	ENSBTAG00000015301	-0.139072523	-0.050778823	-0.088293701
A0A3Q1MTZ4	ADGRG6	A0A3Q1MTZ4	ENSBTAG00000020707	-0.141484178	-0.131891047	-0.009593131
A0AAF6DLX3	MICU2	A0AAF6DLX3	ENSBTAG00000015943	-0.229099953	-0.178082216	-0.051017737
Q2KJ16	PHKG2	A7E3T5; bta:512670; Q2KJ16	ENSBTAG00000003417	-0.178733459	-0.20886543	0.030131971
A7E3T6	CEP27	A7E3T6		0.107691472	0.056344592	0.05134688
F1MU23	FBXL8	F1MU23	ENSBTAG00000023632	-0.105540554	0.06059806	-0.166138614
E1BB36	MGAT2	bta:101906103; E1BB36	ENSBTAG00000033891	0.016556932	-0.027509849	0.044066781
Q3ZCE1	STAP2	bta:505456; Q3ZCE1		-0.318510576	-0.409680289	0.091169714
Q32KU8	NUDCD2	bta:532693; Q32KU8	ENSBTAG00000061380	0.102634912	-0.121306296	0.223941208
G3MYT4	TPBG	G3MYT4	ENSBTAG00000039477	0.193702385	-0.096215315	0.2899177
A0A3Q1M1N0	AMBRA1	A0A3Q1M1N0	ENSBTAG00000004282	0.593911457	0.570786732	0.023124725
E1BEL6	NRP2	E1BEL6	ENSBTAG00000011971	-0.20029865	-0.175571565	-0.024727086
Q2TA14	PRCP	bta:534927; Q2TA14		0.029645462	0.394929926	-0.365284464
A0AAA9S954	CAMKK1	A0AAA9S954; bta:510260	ENSBTAG00000007166	0.072775462	0.025432783	0.047342679
E1BF57	INTS6	E1BF57	ENSBTAG00000002970	-0.157812506	-0.022742462	-0.135070044
A0AAA9S1Y2	CTDP1	A0AAA9S1Y2	ENSBTAG00000053484	-0.318792179	-0.187471983	-0.131320196
E1B986	PRKAB2	bta:512665; E1B986	ENSBTAG00000014387	-0.045339308	-0.182397166	0.137057858
A0AAA9SG44	TBC1D22A	A0AAA9SG44	ENSBTAG00000012291	-0.19416131	0.023672829	-0.217834139
E1BMI4	UXS1	E1BMI4	ENSBTAG00000005614	-0.074406688	0.091179377	-0.165586066
A0AAF6ZL46	REPIN1	A0AAF6ZL46	ENSBTAG00000038241	-0.072464983	-0.136889088	0.064424106
F1N429	SESTD1	bta:789054; F1N429	ENSBTAG00000001861	0.064955442	-0.148141394	0.213096836
A0AAA9S356	TDP1	A0AAA9S356	ENSBTAG00000013138	-0.016630675	0.012348392	-0.028979067
Q3T035	ARPC3	bta:506596; Q3T035	ENSBTAG00000005345	0.02707359	-0.067730776	0.094804366
Q05204	LAMP1	A2VE82; A5D7M2; bta:281897; Q05204; Q17QC6		-0.196977759	-0.125200557	-0.071777202
A0AAA9TH54	SMARCD1	A0AAA9TH54	ENSBTAG00000037935	-0.220944659	-0.475035221	0.254090562
A0A3Q1M9U6	MTMR10	A0A3Q1M9U6	ENSBTAG00000014422	0.035441579	0.22516622	-0.189724642
Q3T087	RPL11	bta:512745; Q3T087		-0.017959091	-0.001488082	-0.01647101

Accession	Gene_Symbol	Gene_ID	Ensembl_Gene ID	log2(ratio(OV24H/OV0H))	log2(ratio(OV24R/OV0H))	log2(ratio(OV24H/OV24R))
G3MZR3	SECISBP2L	G3MZR3	ENSBTAG00000046553	0.037264268	0.081367551	-0.044103283
A0A3Q1MLH8	PTPRE	A0A3Q1MLH8	ENSBTAG00000002435	-0.115662475	-0.205083556	0.089421081
A0AAA9U2I0	NME7	A0AAA9U2I0	ENSBTAG00000002689	0.039183263	-0.15721395	0.196397213
Q2LGB5	TOLLIP	bta:539480; Q2LGB5; Q58D12		0.006614844	0.074928488	-0.068313643
A0AAA9TUZ6	GMDS	A0AAA9TUZ6	ENSBTAG00000012058	-0.432857237	-0.401478236	-0.031379001
F1MB07	LIG4	bta:781252; F1MB07	ENSBTAG00000015868	-0.046908743	-0.076431804	0.029523061
A0A3Q1LR92	RASSF4	A0A3Q1LR92	ENSBTAG00000002669	0.338558769	0.66461475	-0.326055981
F1MWS9	PPAN	bta:522370; F1MWS9	ENSBTAG00000012745	0.078074076	-0.160944664	0.23901874
A5D7P7	ABCB6	A5D7P7; bta:783257	ENSBTAG00000020607	-0.549258439	-0.477343257	-0.071915182
A4IFV0	LDLRAP1	A4IFV0; bta:511199; F1MJ16	ENSBTAG00000001050	-0.021458428	0.132848831	-0.154307259
A0JNG7	FHIP2A	A0JNG7; bta:522276		0.009287768	-0.050570611	0.05985838
E1BIX7	GPSM2	bta:513654; E1BIX7	ENSBTAG00000017124	0.050778823	-0.058770639	0.109549462
Q4ZJ65	NFIC	bta:553098; F6RWF1; Q4ZJ65	ENSBTAG00000008520	-0.161098804	-0.046946497	-0.114152307
A0AAA9TQ25	SURF4	A0AAA9TQ25	ENSBTAG00000051352	0.018992715	0.170415131	-0.151422415
E1BBA0	ARAP3	E1BBA0	ENSBTAG00000015938	0.120247354	0.063059881	0.057187473
F6RR50	RBCK1	F6RR50	ENSBTAG00000017002	-0.124806456	0.039312924	-0.16411938
A0AAA9SCN9	MBD1	A0AAA9SCN9	ENSBTAG00000003801	-0.155517578	-0.145290836	-0.010226742
A6QR44	DGCR8	A6QR44; bta:540254	ENSBTAG00000019869	0.078171614	0.081212068	-0.003040454
Q148D1	CCDC12	bta:617519; Q148D1	ENSBTAG00000011311	-0.130553554	-0.108479101	-0.022074453
F1MRQ7	SH3BGR	bta:617797; F1MRQ7	ENSBTAG00000007068	-0.512185061	-0.48851887	-0.023666191
A7YWH0	TTYH2	A7YWH0; bta:533184; F1N342	ENSBTAG00000011007	-0.183816993	0.045461814	-0.229278807
P21809	BGN	bta:280733; P21809; P79259; Q17QB0; Q5BIM3		-0.006736331	-0.029420683	0.022684352
A0A3Q1MAE3				-0.073001831	0.155794673	-0.228796504
A0A3Q1LVM4	DDHD1	A0A3Q1LVM4	ENSBTAG00000019924	-0.089119058	-0.120597289	0.031478231
A0AAA9T2R9	ZBTB7A	A0AAA9T2R9	ENSBTAG00000020117	0.117244685	-0.209992322	0.327237006
A0AAF7ACE3	PIH1D1	A0AAF7ACE3	ENSBTAG00000016526	0.07436049	0.148976308	-0.074615818
A0AAF6DMA0	CCDC43	A0AAF6DMA0	ENSBTAG00000005693	0.325542639	0.390985113	-0.065442475
A0AAF7ADH5	VPS29	A0AAF7ADH5	ENSBTAG00000008417	-0.017364643	-0.026125976	0.008761333
E1BD36	IL6ST	bta:522155; E1BD36	ENSBTAG00000017745	-0.099790769	-0.103622631	0.003831862
Q2TBH6	ELP4	bta:614139; Q2TBH6	ENSBTAG00000023831	-0.066769012	-0.075909666	0.009140654
Q3SZ96	LZIC	Q3SZ96		0.112231558	0.022517437	0.089714121
A0AAA9T0C4	ZNF384	A0AAA9T0C4	ENSBTAG00000017072	0.207595419	0.142604395	0.064991024
F6Q722	PLGRKT	F6Q722	ENSBTAG00000017530	0.068552669	0.068552669	0
F1MPV5	NFXL1	F1MPV5	ENSBTAG00000002201	0.097465809	0.166819091	-0.069353281
F1MRT5	DOK1	F1MRT5	ENSBTAG00000018579	0.013631866	0.174651832	-0.161019967
A0AAA9SH80	MRPL22	A0AAA9SH80	ENSBTAG00000019457	-0.169403891	-0.121745272	-0.047658618
E1BM89	NCBP3	bta:510317; E1BM89	ENSBTAG00000016621	-0.249510284	-0.10496956	-0.144540724
A0AAA9S0N4	GET4	A0AAA9S0N4	ENSBTAG00000026191	-0.23894702	-0.20624557	-0.03270145
A6QQU1	KATNB1	A6QQU1; bta:508813; F1MVM3	ENSBTAG00000001848	-0.256062445	0.093031402	-0.349093847
A0AAF6YR26	ZGPAT	A0AAF6YR26	ENSBTAG00000007498	-0.371255807	-0.119298928	-0.251956879
A0AAA9TKT2	EED	A0AAA9TKT2	ENSBTAG00000007847	-0.044956161	-0.214346137	0.169389976
A0A3Q1MEX1	RUFY2	A0A3Q1MEX1	ENSBTAG00000002614	-0.117229732	0.032654986	-0.149884718
F6PSX0	IFI35	F6PSX0	ENSBTAG00000007389	-0.400087158	-0.312099478	-0.08798768
Q5E996	NOP16	A4IF95; bta:506421; Q5E996	ENSBTAG00000010734	-0.569751731	-0.628152107	0.058400376
A0AAF6ZJL6	AHSA2	A0AAF6ZJL6	ENSBTAG00000006025	0.556773957	0.584962501	-0.028188544
Q148I5	KTI12	bta:538901; Q148I5		-0.085208043	-0.082019765	-0.003188278
A0AAF7AST6	ACSF3	A0AAF7AST6	ENSBTAG00000015968	0.012887684	0.005742077	0.007145607
F1MDD0	PSMG4	bta:789063; F1MDD0	ENSBTAG00000031249	0.203964758	0.11318249	0.090782268
P32120	ARRB2	O77565; O77566; P32120		-0.077199632	0.104538355	-0.181737987
A0AAA9S9N2	AKAP10	A0AAA9S9N2	ENSBTAG00000003439	0.022460677	0.193210878	-0.1707502
E1B805				-0.163975735	0.081136763	-0.245112498
Q02375	NDUFS4	bta:327680; Q02375	ENSBTAG00000003728	-0.032927803	0.01194432	-0.044872123
A0AAF6DLV9	FDXR	A0AAF6DLV9	ENSBTAG00000006525	-0.01982663	0.040131386	-0.059958016
A0A3Q1LI98	ABL1	A0A3Q1LI98; bta:540876	ENSBTAG00000017976	0.036876002	0.165942284	-0.129066282
Q0VCF9	LSM12	bta:538005; Q0VCF9	ENSBTAG00000016252	0.182415946	0.161537216	0.02087873
A0A3Q1MGA6	WBP4	A0A3Q1MGA6	ENSBTAG00000016637	-0.00503559	0.028207702	-0.033243292
A0A452DHW5	WDR18	A0A452DHW5	ENSBTAG00000002434	-0.181527414	-0.133602588	-0.047924826
Q3SZ13	UQCC2	bta:100607974; Q3SZ13; Q58DH5	ENSBTAG00000054726	0.008296142	0.008296142	0
A0AAF7AP05	MTRF1	A0AAF7AP05	ENSBTAG00000012260	-0.087266836	-0.030106755	-0.057160081
Q0P564	CRBN	bta:511585; Q0P564	ENSBTAG00000019536	0.483322239	0.555215157	-0.071892918
G3MY77	MAGT1	bta:100301169; G3MY77	ENSBTAG00000045931	-0.190348402	-0.068647347	-0.121701055
A0AAA9S9R0	RPL35	A0AAA9S9R0	ENSBTAG00000003205	-0.122553182	-0.194207282	0.071654099
P21793	DCN	bta:280760; P21793; Q3MHN1; Q5U7W0; Q861V7; Q862D9; Q862E8; Q862L4; Q862R5	ENSBTAG00000003505	-0.363590734	-0.109389574	-0.25420116
Q32PB9	RPL38	A6QLN0; bta:615300; Q32PB9	ENSBTAG00000030164	-0.151698374	-0.204447939	0.052749565
Q2NL00	GSTT1	bta:517724; Q2NL00	ENSBTAG00000040298	-0.236932303	-0.12716443	-0.109767873
E1BIE4	EPHB4	E1BIE4	ENSBTAG00000003446	-0.016575556	-0.046376334	0.029800778
A0AAA9T5T6	SH3PXD2A	A0AAA9T5T6	ENSBTAG00000044153	-0.339964318	-0.195574409	-0.144389909
Q29RZ9	DNAAF10	bta:540032; Q29RZ9	ENSBTAG00000010595	0.123454446	0.048958422	0.074496025
P00429	COX6B1	bta:100270792; P00429; Q54A32		0.11780352	0.008382912	0.109420608
A0A3Q1M4P7	EXOC6	A0A3Q1M4P7	ENSBTAG00000021116	-0.082534758	-0.116917752	0.034382994
E1BKA4	HAUS4	E1BKA4	ENSBTAG00000012723	-0.134618133	0.044087754	-0.178705887
A0A3Q1M7S6	TPST2	A0A3Q1M7S6	ENSBTAG00000010368	-0.06086713	-0.294579664	0.233712534
A0A3Q1LJH6	CEP192	A0A3Q1LJH6	ENSBTAG00000013380	0.184424571	0.067343468	0.117081103
A0A3Q1MKE9	IL1RN	A0A3Q1MKE9	ENSBTAG00000019665	0.111031312	0.163498732	-0.05246742
A0A343QZ23	ND5	A0A343QZ23		-0.10437423	-0.031909248	-0.072464983
Q02367	NDUFB6	bta:327665; Q02367; Q3T079		-0.221642775	-0.136753877	-0.084888898
A0A3Q1MI06	LTV1	A0A3Q1MI06	ENSBTAG00000032163	-0.44692156	-0.43089145	-0.01603011
A0AAA9TUV1	ATRN	A0AAA9TUV1	ENSBTAG00000003848	0.045432748	0.030016448	0.0154163
A0AAA9SMX1	TRAPPC3	A0AAA9SMX1	ENSBTAG00000005732	0.472426172	0.001708342	0.470717829
A0A3Q1MTM4	COX15	A0A3Q1MTM4	ENSBTAG00000045703	0.230831726	0.024963285	0.205868441
A0A3Q1LJU5	EML2	A0A3Q1LJU5	ENSBTAG00000015764	0.071338782	0.323257155	-0.251918374
A0AAA9SPV0	H6PD	A0AAA9SPV0	ENSBTAG00000004246	0.037211079	0.258171545	-0.220960466
A4IFD1	PDCD4	A4IFD1; bta:506724	ENSBTAG00000019434	-0.160679354	-0.495926304	0.335246951
Q3T0Z3	UBL5	bta:614551; Q3T0Z3	ENSBTAG00000049452	-0.488371605	-0.360120681	-0.128250923
F1MLI8	PODXL	F1MLI8	ENSBTAG00000010452	0.565627709	0.360057329	0.20557038
Q3T0C6	ATP1B3	bta:532844; Q3T0C6	ENSBTAG00000014140	0.158320817	0.0416048	0.116716017
A0AAF6YJZ6	RPS28	A0AAF6YJZ6	ENSBTAG00000002468	-0.044947406	0.039289131	-0.084236537
A0AAA9RTG5	TOR1B	A0AAA9RTG5	ENSBTAG00000002837	0.158658041	0.102962052	0.055695989
A0AAA9TXF1				-0.32483969	0.137503524	-0.462343214
A0A140T853	IDI1	A0A140T853; bta:514293	ENSBTAG00000004075	0.219102351	0.167366707	0.051735645
Q32PA5	UBE2C	bta:506962; Q32PA5		-0.273688798	-0.035824968	-0.23786383
G5E513				-0.337827808	0.029808809	-0.367636617
A0AAA9THB3	UTP18	A0AAA9THB3	ENSBTAG00000020629	0.211269043	0.191782995	0.019486047
F1N353	BNIP1	bta:614520; F1N353	ENSBTAG00000002021	0.155111117	0.081187936	0.073923182
Q2TBL9	TRAPPC4	bta:785345; Q2TBL9	ENSBTAG00000008075	-0.240510918	-0.178841139	-0.06166978
E1BLT8	ZDHHC5	bta:533250; E1BLT8	ENSBTAG00000002381	-0.117898253	-0.072931522	-0.044966731
A6H754	CDC37L1	A6H754; bta:510572	ENSBTAG00000017137	0.055964358	0.136705205	-0.080740847
A0A0F7RQ40	DDT	A0A0F7RQ40; bta:615999		-0.371212816	-0.512173738	0.140960923
F1MKU4	USP1	F1MKU4	ENSBTAG00000020451	-0.212303604	-0.276070478	0.063766874
A0AAA9SFW5	REL	A0AAA9SFW5	ENSBTAG00000015386	0.141260713	0.130872225	0.010388488
E1BH05	ARHGEF10	E1BH05	ENSBTAG00000008585	-0.264177587	-0.361754075	0.097576488
A0AAA9RTD6				-0.120359439	0.038474148	-0.158833587
Q0IIJ1	ERGIC2	bta:512481; Q0IIJ1		-0.001271657	-0.184135714	0.182864057
A0A3Q1LM29	TRIM24	A0A3Q1LM29; bta:537246	ENSBTAG00000002431	0.011427347	-0.005747797	0.017175144
A0AAA9T6M9	AGPAT1	A0AAA9T6M9	ENSBTAG00000004442	-0.017735482	-0.016363446	-0.001372035
F1MRB9	NOL8	F1MRB9	ENSBTAG00000018613	-0.316389911	-0.025376563	-0.291013348
A4IFM5	CDCA8	A4IFM5; bta:508022	ENSBTAG00000014326	-0.056645249	-0.203486638	0.146841388
Q00361	ATP5ME	bta:338040; Q00361; Q3T0S4		0.083117633	0.063488826	0.019628807
Q32PJ3	ORC3	bta:523714; Q32PJ3		-0.004592137	0.087462841	-0.092054978
A0AAF6YL90	ANGEL2	A0AAF6YL90	ENSBTAG00000003702	-0.261610928	-0.05516005	-0.206450877
A0A452DHL8	HDHD3	A0A452DHL8	ENSBTAG00000000250	-0.368698785	-0.288080324	-0.08061846
F1ML72	RPL34	bta:505603; F1ML72	ENSBTAG00000014226	-0.109917692	-0.185501044	0.075583352
A5D7A6	UTP11L	A5D7A6; bta:537675		-0.261177484	-0.100294288	-0.160883196
A0AAA9SP21	SDCBP	A0AAA9SP21	ENSBTAG00000019910	0.045264734	-0.102720999	0.147985733
Q2KIU0	VTI1B	bta:780809; Q2KIU0		0.027825261	-0.077924472	0.105749732
E1BKH5	SETDB1	bta:100138100; E1BKH5	ENSBTAG00000000098	0.014130329	-0.072805308	0.086935637
F1MDG4	FUT4	F1MDG4	ENSBTAG00000038062	0.034051849	0.145245727	-0.111193879
A0A059NZ16				0.213610669	0.506100876	-0.292490207
A0AAF6ZKW8	NDUFS7	A0AAF6ZKW8	ENSBTAG00000019419	-0.148484323	-0.118916301	-0.029568022
Q2TA42	ARGLU1	bta:540543; Q2TA42	ENSBTAG00000015124	0.38332864	0.335727606	0.047601033
A0AAA9TU04	SCO1	A0AAA9TU04	ENSBTAG00000021780	0.041862023	0.016022018	0.025840005
F1MWS4	RNF25	F1MWS4	ENSBTAG00000003817	0.033276155	1.866578332	-1.833302177
A0AAA9RUC1	MECR	A0AAA9RUC1	ENSBTAG00000017253	-0.025705714	-0.032204351	0.006498637
A0AAA9SND8	EHHADH	A0AAA9SND8; bta:518852	ENSBTAG00000019625	0.145409482	-0.107974754	0.253384236
Q29448	HMGCL	bta:317658; Q148J2; Q29448	ENSBTAG00000021832	0.090422217	0.131566499	-0.041144282
A4FUH5	NDUFAF4	A4FUH5; bta:615440	ENSBTAG00000003174	0.089835334	0.087023062	0.002812272
A0A3Q1NNF6	GTF3C4	A0A3Q1NNF6; bta:540249	ENSBTAG00000004091	-0.259510015	0.228004145	-0.487514161
Q17QR4	CCDC137	bta:511225; Q17QR4	ENSBTAG00000000356	0.020234484	-0.013649062	0.033883545
A0AAA9RZ33	PMM1	A0AAA9RZ33	ENSBTAG00000004009	-0.22851053	-0.230162156	0.001651626
A0A3Q1LXD0	DDX49	A0A3Q1LXD0	ENSBTAG00000000811	-0.344193801	-0.337886927	-0.006306874
P23727	PIK3R1	bta:282307; P23727		-0.015664595	0	-0.015664595
F1N1D8	MINPP1	bta:541067; F1N1D8	ENSBTAG00000005928	-0.215987549	-0.136220273	-0.079767277
Q07112	GGFBPP5	bta:281361; Q07112		-0.035228488	-0.125530882	0.090302394
A0AAA9SU98	DYM	A0AAA9SU98	ENSBTAG00000000024	-0.091118356	-0.151701243	0.060582886
A0AAF6DMC6	SCYL3	A0AAF6DMC6	ENSBTAG00000014794	0.099535674	-0.001747662	0.101283336
E1B860	IFT140	bta:100139697; E1B860	ENSBTAG00000007245	-0.233287062	0.002502507	-0.23578957
A0AAA9RTY1	CD9	A0AAA9RTY1	ENSBTAG00000014764	0.082174513	0.02565619	0.056518323
F6QND5	FGA	F6QND5	ENSBTAG00000001638	0.069972906	0.393898732	-0.323925827
E1BG95	UBP1	E1BG95	ENSBTAG00000003968	-0.087872756	-0.156381534	0.068508778
A0A3Q1MAS5	CDK5RAP2	A0A3Q1MAS5	ENSBTAG00000006532	0.220771763	-0.040749612	0.261526775
A0AAA9SNQ8	OS9	A0AAA9SNQ8	ENSBTAG00000007152	-0.177642915	-0.076781234	-0.10086168
Q3SX30	PSMF1	bta:617807; Q1JPI8; Q3SX30	ENSBTAG00000018417	0.204118649	0.089330892	0.114787757
A8YXY3	SELENOF	A8YXY3; bta:614360	ENSBTAG00000002268	0.366994643	0.246466556	0.120528086
F1MYT8	TRAF3IP2	F1MYT8	ENSBTAG00000008600	0.733485025	0.120371484	0.613113541
A0A3Q1ML04	SBF2	A0A3Q1ML04	ENSBTAG00000043981	-0.318572634	-0.408131724	0.08955909
A0A140T863				-0.202996823	0.111611991	0.091384832
A6QQI8	MMAA	A6QQI8	ENSBTAG00000012800	-0.308930754	-0.300866479	-0.008064275
A0AAF6ZP66	MARCKSL1	A0AAF6ZP66	ENSBTAG00000046862	0.489444188	0.249978253	0.239465935
F1MSX4	MAP3K2	bta:504592; F1MSX4	ENSBTAG00000004216	0.06283108	0.217262834	-0.154431753
A0A3Q1LR42	NADK2	A0A3Q1LR42	ENSBTAG00000011334	0.185451465	0.265550004	-0.080098539
A0A3Q1LT08	STX4	A0A3Q1LT08	ENSBTAG00000007523	0.06165047	0.070246494	-0.008596024
F6QK68	B4GALT7	F6QK68	ENSBTAG00000001767	-0.046134458	-0.197465593	0.151331135
A0AAA9SAU0	KHNYN	A0AAA9SAU0	ENSBTAG00000010666	0.253547415	0.367245401	-0.113697986
A0A452DIX0	PCYOX1L	A0A452DIX0	ENSBTAG00000019951	0.26519899	0.40623757	-0.14103858
A6QQ16	NSL1	A6QQ16; bta:506867; E1BPS9		0.049314937	0.066837765	-0.017522827
A6QLC4	CDH1	A6QLC4		-0.194462691	0.329926289	-0.52438898
F1MKN3	MRPS34	F1MKN3	ENSBTAG00000026415	-0.376951391	-0.255508864	-0.121442527
A0A3Q1LYZ0	RIC1	A0A3Q1LYZ0	ENSBTAG00000001283	-0.058416686	-0.108677813	0.050261126
A0A1D8QLT6				-0.024166311	0.012725066	-0.036891377
A6QQ81	DSN1	A6QQ81; bta:508271	ENSBTAG00000013915	-0.129958594	0.110480866	-0.24043946
E1BHG2	BEND3	E1BHG2	ENSBTAG00000012534	-0.310437275	-0.283581783	-0.026855492
Q3T0E0	ATOX1	bta:613998; Q3T0E0	ENSBTAG00000008340	0.206962381	-0.057402311	0.264364692
Q27955	KCNAB2	bta:541597; Q27955; Q58HC4		-0.031449639	-0.007798371	-0.023651268
A0A3Q1MQA4	ZFAND2B	A0A3Q1MQA4	ENSBTAG00000020606	0.152458131	0.108106664	0.044351467
E1BIL7	ZKSCAN1	bta:100140434; E1BIL7	ENSBTAG00000016757	-0.121254643	-0.037281016	-0.083973627
Q3ZCD0	CD81	bta:511435; Q3ZCD0		0.409875794	0.510961919	-0.101086125
Q2KIS5	HSD17B11	bta:527592; Q2KIS5	ENSBTAG00000006307	0.376225355	0.425937568	-0.049712212
Q8MJJ1	SPG21	bta:404069; Q17QI9; Q8MJJ1	ENSBTAG00000011540	-0.036034602	-0.030431896	-0.005602706
F1MG31	CDCA2	F1MG31	ENSBTAG00000002756	0.092201383	-0.386278506	0.478479889
A0A452DI48	ALKBH5	A0A452DI48	ENSBTAG00000025046	-0.030469877	-0.210631595	0.180161718
G3MZK0	ADO	bta:100297937; G3MZK0	ENSBTAG00000045717	0.34004105	0.434677925	-0.094636874
E1BMF7	USP13	bta:100141289; E1BMF7	ENSBTAG00000005946	-0.086309572	-0.11216774	0.025858168
A0AAA9S5N1	BDH2	A0AAA9S5N1	ENSBTAG00000002526	-0.001456532	0.253052002	-0.254508534
A0A3Q1M3C1	TEAD1	A0A3Q1M3C1	ENSBTAG00000032657	0.353131594	0.495353667	-0.142222073
A0A3Q1LN92	TGM3	A0A3Q1LN92	ENSBTAG00000001785	-0.006778533	0.129283017	-0.13606155
A0A3Q1N1V2	NIT2	A0A3Q1N1V2	ENSBTAG00000004833	0.222392421	0.233059346	-0.010666925
E1BNF0	RBM33	E1BNF0	ENSBTAG00000010243	-0.016103308	0.075164749	-0.091268058
Q0II22	RNF126	bta:507447; Q0II22	ENSBTAG00000014349	0.075387122	0.00873481	0.066652312
Q2KI71	TRA2A	bta:534586; Q2KI71	ENSBTAG00000012622	0.124557638	0.085794021	0.038763617
F1MYS0	BBX	bta:533187; F1MYS0	ENSBTAG00000006893	-0.147296091	-0.23114107	0.083844979
A0AAF6Z8E2	FHIP1B	A0AAF6Z8E2	ENSBTAG00000020515	-0.41457023	-0.095635279	-0.318934951
F1MW70	DUSP12	bta:505302; F1MW70	ENSBTAG00000021754	0.108482045	0.055964358	0.052517687
A0A0X9PX45	RAD51	A0A0X9PX45		0.307343302	0.075602686	0.231740616
A0AAA9SCB7	LEO1	A0AAA9SCB7	ENSBTAG00000013830	0.064620847	0.086039831	-0.021418984
E1BFW1	CCDC134	bta:516012; E1BFW1	ENSBTAG00000021173	0.298019764	0.194183953	0.103835811
Q2TBL4	POLR3C	bta:507314; Q2TBL4	ENSBTAG00000021637	-0.043164179	-0.057844075	0.014679895
F1MIB3	ZNF462	F1MIB3	ENSBTAG00000019187	-0.077745095	-0.004641382	-0.073103713
A0AAA9TM42	CDK14	A0AAA9TM42	ENSBTAG00000048664	-0.046213178	-0.225039083	0.178825906
Q58DW3	RPL29	bta:507270; Q58DW3	ENSBTAG00000018628	-0.017584312	-0.048471358	0.030887045

Accession	Gene_Symbol	Gene_ID	Ensembl_Gene ID	log2(ratio(OV24H/OV0H))	log2(ratio(OV24R/OV0H))	log2(ratio(OV24H/OV24R))
Q3T0D3	NTAQ1	bta:510463; Q3T0D3	ENSBTAG00000009916	-0.06357731	-0.152453865	0.088876555
F1MZC3	RPP40	F1MZC3	ENSBTAG00000003332	-0.216811389	-0.158663822	-0.058147567
A0AAF6YKL8	CCDC115	A0AAF6YKL8	ENSBTAG00000015841	-0.075551033	-0.117039421	0.041488388
A0AAA9RYP5	RBMS1	A0AAA9RYP5	ENSBTAG00000005180	0.053826991	0.044994137	0.008832854
Q3ZCB8	EMC3	bta:508371; Q3ZCB8	ENSBTAG00000003882	-0.096118154	-0.191035825	0.094917671
A0AAA9SK35	PRKG2	A0AAA9SK35	ENSBTAG00000002978	0.443817742	-0.087283413	0.531101154
E1BPW0	ENTPD5	E1BPW0		0.33230191	0.418287337	-0.085985427
G3MZ71	KRT2	G3MZ71	ENSBTAG00000039261	-0.482031805	0.899317376	-1.381349181
A0A3Q1MNH6				0.338286783	0.055853235	0.282433549
A0A3Q1MH99	ZNF207	A0A3Q1MH99	ENSBTAG00000008883	0.137209426	0.07868335	0.058526077
F1MMM8	MRPL19	F1MMM8	ENSBTAG00000002557	-0.36312273	-0.268459535	-0.094663196
Q92176	CORO1A	bta:282196; Q3SZF9; Q92176	ENSBTAG00000008631	0.118195129	0.050331676	0.067863453
Q1RMU6	XXYLT1	bta:533703; F1N283; Q1RMU6	ENSBTAG00000014564	0.08894177	0.104164593	-0.015222823
Q1LZD3	TSSC4	bta:509559; Q1LZD3	ENSBTAG00000047793	-0.116501253	-0.059917724	-0.056583528
F1MRP0	GHDC	F1MRP0	ENSBTAG00000000666	-0.057657825	0.119276499	-0.176934324
A0AAA9SK55	RAB4B	A0AAA9SK55	ENSBTAG00000009079	-0.190248421	-0.135309507	-0.054938914
Q0III8	RNASET2	bta:508245; Q0III8		0.098227322	0.16018307	-0.061955748
G3MZ52	CGGBP1	bta:613824; G3MZ52	ENSBTAG00000046959	0.388656055	0.224957628	0.163698427
A0AAA9TPV0	LIN7C	A0AAA9TPV0	ENSBTAG00000001460	0.204183623	0.213538788	-0.009355165
A5PJP3	RAB22A	A5PJP3; bta:617385		-0.106599066	-0.075948853	-0.030650213
Q1LZ74	OARD1	bta:768056; Q1LZ74	ENSBTAG00000005975	0.343904322	0.245577209	0.098327114
Q1LZC4	MGME1	bta:506205; F6Q192; Q1LZC4	ENSBTAG00000003425	-0.08151282	-0.087738051	0.006225231
E1BLQ9	LPIN3	E1BLQ9	ENSBTAG00000009165	0.094378824	0.632923539	-0.538544715
F1MS53	RAPGEF6	F1MS53	ENSBTAG00000000682	-0.089942125	-0.109763566	0.019821441
A2VE79	NUDT3	A2VE79; bta:618855	ENSBTAG00000019684	0.15810707	0.133836861	0.024270209
Q32P59	SLIRP	bta:613614; Q32P59		-0.169291268	-0.266588469	0.097297201
A0AAA9THI0	HTRA2	A0AAA9THI0	ENSBTAG00000020124	0.263287755	0.263287755	0
A0A3Q1NNN2	SLC7A5	A0A3Q1NNN2	ENSBTAG00000006731	0.328930588	0.556984345	-0.228053756
A0A3Q1MAN2	MRTFB	A0A3Q1MAN2	ENSBTAG00000008728	-0.023651268	0.146462977	-0.170114245
E1BCC2	TSC22D2	bta:541277; E1BCC2	ENSBTAG00000018402	0.314281283	0.156445087	0.157836196
A0AAF6YMV1	KRT19	A0AAF6YMV1	ENSBTAG00000004905	-0.009847786	1.724546335	-1.734394121
A0AAF7ALD7	FAM110B	A0AAF7ALD7	ENSBTAG00000050550	0.194786105	-0.10515823	0.299944335
H7BWV5	CUTC	H7BWV5	ENSBTAG00000002971	0.097765767	0.295824341	-0.198058574
A0AAA9SMC0	GMIP	A0AAA9SMC0	ENSBTAG00000015144	0.050006669	0.048560358	0.001446311
A0A3Q1M238	SPPL2A	A0A3Q1M238; bta:530325	ENSBTAG00000011831	-0.106590431	-0.082544957	-0.024045474
A0AAA9SX30	EVI5	A0AAA9SX30; bta:536706	ENSBTAG00000011363	-0.012939056	0.12731883	-0.140257886
A0AAA9SY66	FAM177A1	A0AAA9SY66	ENSBTAG00000009680	0.174066282	0.017741582	0.156324699
A0A3Q1LN49	CDK17	A0A3Q1LN49	ENSBTAG00000001510	0.04527408	-0.282530581	0.327804661
E1BGY1	FAM111B	E1BGY1	ENSBTAG00000036016	0.173393678	0.161676294	0.011717385
A0AAA9TLP0	HEBP2	A0AAA9TLP0	ENSBTAG00000000243	0.085985427	0.159220634	-0.073235208
A5PJ71	MRPL41	A5PJ71; bta:506521	ENSBTAG00000039138	-0.211355228	-0.085960816	-0.125394412
B7TCI5	TBL1X	B7TCI5; bta:515972	ENSBTAG00000018112	0.021648563	0.003112612	0.018535951
A5D7S7	LOX	A5D7S7; bta:280841	ENSBTAG00000012994	-0.014316442	-0.03805419	0.023737748
F1N3C5	TBC1D5	F1N3C5	ENSBTAG00000030581	-0.193771743	-0.083456231	-0.110315512
P61602	NCALD	A1L533; bta:281940; P29554; P61602; Q0IIL3; Q9H0W2	ENSBTAG00000026963	0.21729826	0.097160636	0.120137623
E1BCP5	C19H17orf75	E1BCP5	ENSBTAG00000020994	-0.306175126	-0.229891541	-0.076283585
G5E5Y0	SNRPB2	G5E5Y0	ENSBTAG00000027506	-0.058043008	0.018840082	-0.07688309
P68301	MT2A	bta:404070; P09579; P68301; Q546G1	ENSBTAG00000023659	0.031194622	0.608421474	-0.577226852
Q3ZBN8	DNAJC19	bta:513918; Q3ZBN8	ENSBTAG00000023513	0.073351742	0.063453281	0.009898461
A0A3Q1M0Z5	GTF3C3	A0A3Q1M0Z5	ENSBTAG00000007779	-0.210217707	-0.040458143	-0.169759564
Q32LA2	THEX1	bta:540728; Q32LA2	ENSBTAG00000009685	-0.038639027	0.01366055	-0.052299577
Q17QM9	CNRIP1	bta:539715; Q17QM9	ENSBTAG00000001408	-0.086496581	-0.044877492	-0.041619089
Q29RQ1	C7	bta:507339; Q29RQ1		-0.092915897	0.188830104	-0.281746001
A0A3Q1MTJ1	MACO1	A0A3Q1MTJ1	ENSBTAG00000002988	-0.036809524	-0.160278782	0.123469258
A0AAF6YVI0	APOO	A0AAF6YVI0	ENSBTAG00000010937	0.072756342	0.027715378	0.045040964
A0A3Q1M2M3	ZNF148	A0A3Q1M2M3	ENSBTAG00000050482	0.111983161	-0.046096415	0.158079576
A0A3Q1MEG2	BECN1	A0A3Q1MEG2	ENSBTAG00000019914	0.030696797	0.120350643	-0.089653846
E1BGS8	ATG16L1	E1BGS8	ENSBTAG00000003928	0.001450674	-0.030806047	0.032256721
A0AAA9THL1	AATF	A0AAA9THL1; bta:786013	ENSBTAG00000021927	-0.050228033	0.0252557	-0.075483734
A0AAA9S240	COX5A	A0AAA9S240	ENSBTAG00000017267	0.699955023	-0.025422597	0.72537762
A0A3Q1N285	SECISBP2	A0A3Q1N285	ENSBTAG00000001939	-0.117716238	-0.167630667	0.049914429
E1BN44	RREB1	E1BN44	ENSBTAG00000005980	-0.106499621	-0.093469673	-0.013029948
Q17QB7	RAB30	bta:540583; Q17QB7	ENSBTAG00000002417	0	0.044194115	-0.044194115
A0AAA9SCB5	AKT3	A0AAA9SCB5	ENSBTAG00000017788	-0.016873819	0.030642463	-0.047516281
Q3ZC01	CNOT7	bta:508055; Q3ZC01	ENSBTAG00000018036	0.276280124	0.212010403	0.064269722
A0A3Q1M0R0	ADGRE5	A0A3Q1M0R0	ENSBTAG00000021818	0.122826673	0.03924954	0.083577133
A6QLA2	PI4K2B	A6QLA2; bta:521790		0.145064013	0.020308982	0.124755031
Q0P5N2	TSPYL1	bta:538541; Q0P5N2	ENSBTAG00000010885	-0.128866595	-0.021821283	-0.107045311
A0AAF6Z5M1	RABGGTB	A0AAF6Z5M1	ENSBTAG00000018447	0.429357171	0.133901287	0.295455884
A0A3Q1MQS6	SURF1	A0A3Q1MQS6	ENSBTAG00000030384	-0.190942783	-0.038939689	-0.152003093
Q3SZA4	SLC25A20	bta:518758; F1MCM8; Q3SZA4	ENSBTAG00000000191	0.028747054	0.087996481	-0.059249427
A7MAY9	ZNF592	A7MAY9; bta:516451		-0.029294307	0.170730302	-0.200024608
E1BHW7	RMC1	bta:100140797; E1BHW7	ENSBTAG00000015190	0.076621282	0.105353	-0.028731719
E1BEC1	WDR7	E1BEC1	ENSBTAG00000004005	-0.207911835	-0.221127454	0.01321562
A0AAF6DM62	KHDC4	A0AAF6DM62	ENSBTAG00000000108	-0.336732504	0.034080995	-0.370813499
F1MN39	IFRD1	F1MN39	ENSBTAG00000010549	-0.224193914	0.037905498	-0.262099412
A6QQT6	REEP3	A0A3Q1MR20; A6QQT6; bta:512704; F1MV24	ENSBTAG00000019755	-0.048560358	-0.123821761	0.075261403
F1MQH0	ELMO1	F1MQH0	ENSBTAG00000003490	0.131244533	0.1008175	0.030427034
E1B8Z4	STAT2	bta:511023; E1B8Z4	ENSBTAG00000004380	0.037331039	0.012551317	0.024779722
A0AAA9RTE2	UQCC1	A0AAA9RTE2	ENSBTAG00000030990	0.071419061	0.045855689	0.025563372
F1N2I5	CMBL	bta:516405; F1N2I5	ENSBTAG00000010801	0.251665175	0.29995527	-0.048290095
F1MMY4	NSD2	F1MMY4	ENSBTAG00000007986	-0.419903254	-0.416362897	-0.003540358
F1MM07	MYH7	F1MM07	ENSBTAG00000009703	-0.223316338	-0.168309571	-0.055006767
G3N3L6	TIMM29	G3N3L6	ENSBTAG00000045893	0.004597014	0.030373649	-0.025776635
A0AAA9TWV2	RAB4A	A0AAA9TWV2	ENSBTAG00000018857	0.119596852	0.099714435	0.019882417
F1MQX7	PRMT7	F1MQX7	ENSBTAG00000009638	0.070389328	0.130656358	-0.06026703
A0A3Q1LHS7	CDC42EP4	A0A3Q1LHS7	ENSBTAG00000053044	0.047099777	0.21462	-0.167520223
Q56K03	RPL27A	bta:404190; Q2TBT4; Q56K03		-0.014766711	-0.008841877	-0.005924834
Q3T171	RPL36	A5PJ75; bta:768327; Q3T171		-0.072731884	-0.122825224	0.05009334
A0AAA9T2L5	SRCAP	A0AAA9T2L5	ENSBTAG00000013917	0.074546953	0.032421478	0.042125476
A0A3Q1LVY4	MSI2	A0A3Q1LVY4	ENSBTAG00000004406	-0.00873481	0.118377108	-0.127111918
Q9N2I8	TXNRD2	bta:282389; Q9N2I8	ENSBTAG00000043581	0.06453066	0.196285588	-0.131754928
E1BLX2	SETD1A	bta:782887; E1BLX2	ENSBTAG00000002345	-0.168122759	0.042644337	-0.210767096
A0A452DIQ8	NOL9	A0A452DIQ8	ENSBTAG00000016733	-0.079727192	-0.037140735	-0.042586457
F1MVI8	DCAF13	F1MVI8	ENSBTAG00000021449	0.135866578	0.095731465	0.040135112
A0A3Q1NEN5	NOB1	A0A3Q1NEN5	ENSBTAG00000020633	-0.509425111	-0.424427458	-0.084997653
A0AAA9TXU2	RBM19	A0AAA9TXU2	ENSBTAG00000031824	-0.386154684	-0.186607846	-0.199546837
E1BJT5	NKTR	bta:100335533; E1BJT5	ENSBTAG00000002795	-0.317577945	-0.377764951	0.060187006
Q29455	DNAJC5	bta:282216; Q0VCC7; Q29455; Q29456	ENSBTAG00000015796	-0.19891561	-0.101717766	-0.097197843
E1BLS8	EMILIN1	E1BLS8	ENSBTAG00000011324	-0.020084246	0.192645078	-0.212729324
A0A3Q1LZC0	GK	A0A3Q1LZC0	ENSBTAG00000008124	0.001417882	0.130133913	-0.128716031
A0AAF6DM30	IRAK3	A0AAF6DM30	ENSBTAG00000007636	0.078336972	0.177203648	-0.098866675
A0A3Q1MA47	VPS8	A0A3Q1MA47	ENSBTAG00000005209	-0.340660971	-0.27640643	-0.064254542
Q02379	NDUFS5	bta:338057; Q02379; Q3ZC47	ENSBTAG00000010232	-0.362975844	-0.286512001	-0.076463842
A0A3Q1NLK1	VEZF1	A0A3Q1NLK1	ENSBTAG00000004193	-0.101629571	0.010216396	-0.111845967
A0A3Q1MLQ1	MED6	A0A3Q1MLQ1	ENSBTAG00000006786	-0.047965325	-0.029208463	-0.018756863
A0AAA9T1A9	SRP14	A0AAA9T1A9	ENSBTAG00000014353	0.020037753	-0.005776564	0.025814318
Q58D05	TRAFD1	bta:512642; Q08E03; Q58D05	ENSBTAG00000008761	-0.30525722	-0.183678157	-0.121579064
A0AAA9SMS2	CCNK	A0AAA9SMS2; bta:530744	ENSBTAG00000018636	0.110070778	0.045040964	0.065029813
Q0VD19	SMPD1	bta:505097; Q0VD19	ENSBTAG00000015628	0.121048766	0.296745443	-0.175696677
Q17QQ4	TAF9	bta:532936; Q17QQ4	ENSBTAG00000057772	0.189149129	0.128813466	0.060335663
A0A452DJK1	PPIF	A0A452DJK1	ENSBTAG00000016711	0.026855492	0.040098069	-0.013242577
Q32T06				-0.129510733	0.102641167	-0.2321519
A4FV41	UBAC2	A4FV41; bta:100125312	ENSBTAG00000005682	0.030578496	-0.180853226	0.211431723
A0AAA9U0I9	SPATA13	A0AAA9U0I9	ENSBTAG00000019545	-0.081506258	-0.223747701	0.142241443
F1MV20	ZFP36L2	F1MV20	ENSBTAG00000003553	-0.086783766	-0.051159856	-0.03562391
A0A3Q1MBH0	DLG3	A0A3Q1MBH0	ENSBTAG00000011291	0.235505998	0.240550392	-0.005044393
F1N545	ANKRD13A	bta:511883; F1N545	ENSBTAG00000014376	0.030183005	0.065588342	-0.035405336
A0AAA9SZB4	TLE3	A0AAA9SZB4	ENSBTAG00000015580	-0.012239268	0.209494539	-0.221733807
Q3T0D8	LAMTOR1	bta:614849; Q3T0D8		0.004529658	-0.077666494	0.082196152
A6H7G8	GTF2H4	A6H7G8; bta:100137723	ENSBTAG00000046757	-0.158730415	-0.095827846	-0.06290257
A7MB61	NAB1	A7MB61; bta:516781; F1MH19	ENSBTAG00000000266	-0.230590377	-0.2546025	0.024012122
G1FM83				0.031160931	0.12465562	-0.093494688
A0AAA9T0E2	MRPS6	A0AAA9T0E2	ENSBTAG00000012594	0.026273694	-0.006252209	0.032525904
A0AAA9S2J4	UBAC1	A0AAA9S2J4	ENSBTAG00000039851	-0.197069641	0.008044789	-0.20511443
E1BHI2	RMND5A	bta:516731; E1BHI2	ENSBTAG00000021140	0.010703669	-0.001535599	0.012239268
A0AAA9SKZ0	SENP3	A0AAA9SKZ0	ENSBTAG00000000131	-0.111963324	0.110653834	-0.222617158
F1N6L1	VARS2	F1N6L1	ENSBTAG00000010698	-0.165254778	-0.082310353	-0.082944425
A0AAA9SGC0	LIMS2	A0AAA9SGC0	ENSBTAG00000006262	-0.089901887	-0.110801967	0.020900081
A0A3Q1LZK2	SAP30BP	A0A3Q1LZK2	ENSBTAG00000011717	0.115820757	0.119936866	-0.004116108
P56966	GGPS1	bta:780882; P56966; Q0VC78	ENSBTAG00000013068	0.264067115	0.247193297	0.016873819
A0A3Q1MBQ1	PRAG1	A0A3Q1MBQ1	ENSBTAG00000000357	-0.037474705	0.161362988	-0.198837693
Q1RMX9	DCUN1D5	bta:508759; Q1RMX9	ENSBTAG00000003087	-0.108766	-0.019017226	-0.089748773
A0A3Q1M4M7	BCR	A0A3Q1M4M7	ENSBTAG00000020566	-0.082989365	-0.2317152	0.148725836
Q2NKZ9	SCPEP1	bta:505054; F1MLZ3; Q2NKZ9	ENSBTAG00000007893	-0.064597867	0.082788599	-0.147386465
F1MUW4	APAF1	F1MUW4	ENSBTAG00000021661	0.062060968	0.003958014	0.058102955
A0A140T891	VNN1	A0A140T891	ENSBTAG00000015094	0.070389328	0.389817084	-0.319427756
A0A3Q1M1E8	PDE3A	A0A3Q1M1E8	ENSBTAG00000017260	-0.369700676	-0.435513457	0.065812781
A4FUI2	SNRPE	A4FUI2; bta:531493	ENSBTAG00000050130	0.103729111	0.070462489	0.033266623
G3N186	PARP2	G3N186	ENSBTAG00000009374	-0.255937231	-0.174874217	-0.081063013
Q2KHX4	SEC61A2	bta:614128; Q2KHX4	ENSBTAG00000019508	0.471108381	0.580944648	-0.109836267
A0AAF6YK92				0.199093585	0.260251965	-0.06115838
A0A3Q1M7D4	VLDLR	A0A3Q1M7D4	ENSBTAG00000018517	0.123104145	0.544805374	-0.42170123
O18778	PHYH	bta:281400; O18778; Q3T0C0		-0.451937082	-0.266974404	-0.184962678
A0AAA9S627				-0.249490469	-0.207198748	-0.042291722
O77635	ADAM-9	O77635		-0.142988781	-0.048144448	-0.094844333
A0AAA9T0A2	POGZ	A0AAA9T0A2	ENSBTAG00000021289	0.016614714	-0.018217858	0.034832572
F6RTG2	PRPF39	F6RTG2	ENSBTAG00000002603	0.452363035	0.384168674	0.068194361
A2I7N2	SERPINA3-6	A2I7N2		-0.084091956	0.264879285	-0.348971241
A0JN51	ELF1	A0JN51; bta:505251	ENSBTAG00000007356	-0.125530882	0.160543765	-0.286074647
A0AAA9S481	TAF7	A0AAA9S481	ENSBTAG00000070085	0.433835095	0.204031218	0.229803876
E1BNA3	AP4E1	E1BNA3	ENSBTAG00000013616	0.118270197	0.346681207	-0.22841101
A0AAA9TSC9	DECR1	A0AAA9TSC9	ENSBTAG00000019663	0.02058927	0.201505055	-0.180915785
Q2T9V8	DTD1	bta:514493; Q2T9V8		0.072314205	0.034637002	0.037677203
A0A3Q1MNC2	WLS	A0A3Q1MNC2	ENSBTAG00000012976	-0.215635055	-0.277621942	0.061986887
Q2T9T7	DUSP3	bta:615432; Q2T9T7	ENSBTAG00000003966	-0.002851177	0.092397138	-0.095248315
Q0VCJ8	METTL9	bta:787067; Q0VCJ8		-0.289628616	-0.2923152	0.002686584
A7YVD7	NDUFAF6	A7YVD7; bta:523017	ENSBTAG00000007570	-0.026073512	0.02983523	-0.055908741
E1BHR3	HDGFL3	bta:784070; E1BHR3	ENSBTAG00000018527	0.024216237	0.199672345	-0.175456107
F1MZK8	PREPL	F1MZK8	ENSBTAG00000017429	-0.075057667	0.072709322	-0.147766989
A0A3Q1MQY2	INTS9	A0A3Q1MQY2	ENSBTAG00000008845	-0.119175673	-0.04945205	-0.069723622
A0A3Q1N6H0	C19H17orf49	A0A3Q1N6H0	ENSBTAG00000021931	0.064922685	0.11627827	-0.051355585
Q2KI32	RAB3IP	bta:533284; Q2KI32	ENSBTAG00000031950	-0.250604956	-0.034287049	-0.216317907
Q1RMN9	LOC510860	bta:510860; Q1RMN9	ENSBTAG00000039196	-0.0489096	0.231873474	-0.280783075
A0AAA9T355	TXNDC17	A0AAA9T355; bta:404159	ENSBTAG00000056053	0.287677527	0.013342939	0.274334587
A0A3Q1NB22	PPP2R3A	A0A3Q1NB22	ENSBTAG00000023416	-0.102675381	-0.188707687	0.086032306
A0AAA9S5X2	MYADM	A0AAA9S5X2	ENSBTAG00000018152	-0.16498426	0.04026387	-0.20524813
Q1LZ96	ATPAF2	bta:513521; Q1LZ96	ENSBTAG00000008801	0.048570559	0.094198642	-0.045628083
A6QL75	PTP4A2	A6QL75; bta:614435; F1MEE0		0.111031312	0.263034406	-0.152003093
F1N5A3	PNISR	F1N5A3	ENSBTAG00000019730	0.270357747	0.111963324	0.158394423
F1MXU4	SYDE1	F1MXU4	ENSBTAG00000015184	0.084227907	-0.071112302	0.155340209
Q08DI1	ETAA1	bta:282224; Q08DI1		-0.600290784	-0.579714423	-0.020576361
A0A3Q1MBG8	SMG7	A0A3Q1MBG8	ENSBTAG00000008003	-0.232455649	-0.224445106	-0.008010543
F1MI38	ICAM1	F1MI38	ENSBTAG00000010303	0.243249293	0.302014451	-0.058765158
E1BBI6	CCNYL1	E1BBI6	ENSBTAG00000016297	0.13467748	0.220778217	-0.086100737
A0AAA9T3I7	B4GALT4	A0AAA9T3I7	ENSBTAG00000001390	-0.019236219	0.010878054	-0.030114273
Q3T0J1	FBXO4	bta:534852; Q3T0J1	ENSBTAG00000016981	-0.513808629	-0.487301675	-0.026506954
A0AAA9SHX1	NUBP1	A0AAA9SHX1	ENSBTAG00000009560	0.102499363	0.107837742	-0.005338378
Q17QT7	CYRIA	bta:504763; Q17QT7	ENSBTAG00000012779	-0.170468287	-0.157485567	-0.01298272
A0AAF6DLL4	CRABP2	A0AAF6DLL4	ENSBTAG00000005716	-0.340288632	0.191592144	-0.531880776
A0AAF6ZJN1	RSRC2	A0AAF6ZJN1	ENSBTAG00000006118	0.135253414	0.004937923	0.130315491
Q17QQ1	LAMTOR3	bta:533155; Q17QQ1	ENSBTAG00000007923	0.251176917	0.032789935	0.218386982
A0AAA9SI39	PIP5K1A	A0AAA9SI39	ENSBTAG00000006099	-0.011622984	0.10867125	-0.120294234
A0AAA9TIG1	PHRF1	A0AAA9TIG1	ENSBTAG00000046359	-0.309626499	-0.36866197	0.05903547
Q3SZB7	FBP1	bta:513483; Q3SZB7	ENSBTAG00000009733	0.043501639	0.194484078	-0.150982439
F1MP14	FOXK1	F1MP14	ENSBTAG00000019310	-0.096263243	0.011076408	-0.107339651
F1N4L1	SLC4A2	bta:404084; F1N4L1	ENSBTAG00000011226	0.033434228	0.071719291	-0.038285063
A0A3Q1LSG1	TMEM123	A0A3Q1LSG1	ENSBTAG00000049233	0.063492924	0.200996447	-0.137503524
Q0VCH8	TMEM65	bta:614243; Q0VCH8	ENSBTAG00000022114	-0.013070939	-0.080263294	0.067192355

Accession	Gene_Symbol	Gene_ID	Ensembl_Gene ID	log2(ratio(OV24H/OV0H))	log2(ratio(OV24R/OV0H))	log2(ratio(OV24H/OV24R))
F1MTC4	ZNF629	F1MTC4	ENSBTAG00000026307	0.029808809	-0.024298596	0.054107405
Q2TBK5	TMED1	bta:538144; Q2TBK5	ENSBTAG00000010968	0.018399838	0.122581877	-0.104182038
F1N3Q4	PALM	F1N3Q4	ENSBTAG00000019771	-0.05471717	-0.166864274	0.112147105
Q8HYI9	PFDN5	bta:282848; Q3T0V1; Q8HYI9	ENSBTAG00000008935	0.523034752	0.402426068	0.120608683
Q17R02	NSD3	F6Q1Q19; Q17R02	ENSBTAG00000001529	-0.008675282	-0.027946541	0.019271259
F1N026	ITM2B	F1N026	ENSBTAG00000003109	0.037818549	0.320010892	-0.282192343
A0AAA9TBL4	NUDT1	A0AAA9TBL4; bta:525496	ENSBTAG00000002480	-0.165935718	0.243113712	-0.409049429
A0AAF6Z1J5	MTIF2	A0AAF6Z1J5	ENSBTAG00000015481	0.045562577	0.128356926	-0.082794349
F1MYY8	GTF3C5	F1MYY8	ENSBTAG00000021373	0.030474958	0.214963013	-0.184488055
A6H791	TRIM61A	A6H791; bta:510283		-0.321928095	-0.320186761	-0.001741334
A0JNC1	CDS2	A0JNC1; bta:614834	ENSBTAG00000006066	0.048759312	0.171260212	-0.1225009
A0AAA9U1A1	ACSL5	A0AAA9U1A1	ENSBTAG00000006707	0.005740174	-0.014451156	0.02019133
A0A3S5ZPA0	EXT2	A0A3S5ZPA0	ENSBTAG00000019644	-0.102674059	-0.093581413	-0.009092646
F1MN55	TAPT1	F1MN55	ENSBTAG00000019451	-0.1935501	-0.125646035	-0.067904065
A0AAA9SV06	ORC1	A0AAA9SV06	ENSBTAG00000002719	-0.302796235	-0.035448836	-0.267347399
A3KN49	HIF1AN	A3KN49; bta:520864		0.471536439	0.407982742	0.063553698
A0AAA9SGV5	TPD52	A0AAA9SGV5	ENSBTAG00000007335	0.471592946	0.406983865	0.064609081
A6QLE1	TDRD7	A6QLE1; bta:506702	ENSBTAG00000003719	-0.045720736	0.170781963	-0.216502699
F1N3R0	SCRN2	F1N3R0	ENSBTAG00000001828	-0.234061834	-0.067876518	-0.166185316
P67810	SEC11A	A6QQV7; bta:282078; O75957; P21378; P67810	ENSBTAG00000011239	-0.017382078	-0.008664861	-0.008717217
A0AAA9SS58	DYNC2I2	A0AAA9SS58	ENSBTAG00000015343	-0.434146322	-0.226947575	-0.207198748
A0AAA9S1I4	MAX	A0AAA9S1I4	ENSBTAG00000017994	0.061400545	-0.026499225	0.08789977
A0AAA9T6R0	PRKD1	A0AAA9T6R0	ENSBTAG00000012789	0.135420209	0.147875208	-0.012454999
Q3SZC0	ERH	bta:508901; Q3SZC0	ENSBTAG00000017798	0.672946873	0.104574591	0.568372282
A0A3Q1MHW6	TBC1D12	A0A3Q1MHW6	ENSBTAG00000009693	-0.001450674	0.027289222	-0.028739895
A0A3Q1LP41	MON1B	A0A3Q1LP41	ENSBTAG00000052957	0.047607882	0.064130337	-0.016522456
E1BGD5	MUL1	E1BGD5	ENSBTAG00000020850	-0.167877173	-0.130008987	-0.037868186
A3KMZ9	SNRNP27	A3KMZ9; bta:790677		-0.043113106	-0.147940306	0.10482719
G3MZF6	WDR76	G3MZF6	ENSBTAG00000017215	0.116988368	0.009794301	0.107194066
A0A3Q1LZZ0	ATP6AP1	A0A3Q1LZZ0	ENSBTAG00000012117	-0.104682505	-0.084008937	-0.020673568
A0AAA9S1C6	LRRN4CL	A0AAA9S1C6	ENSBTAG00000010484	0.01972947	0.122527316	-0.102797846
G3MYZ7	USP34	G3MYZ7	ENSBTAG00000006027	-0.179930579	-0.015208234	-0.164722345
A0AAA9RZF9	TNS2	A0AAA9RZF9	ENSBTAG00000006904	-0.078076589	-0.135504869	0.05742828
A0AAF6Z9X3	USP21	A0AAF6Z9X3	ENSBTAG00000021893	0.024104557	0.100774325	-0.076669768
F1MLY7	ATR	bta:504869; F1MLY7	ENSBTAG00000011529	-0.19571225	-0.086926756	-0.108785494
A0AAF6ZVD1				0.435476391	0.284943186	0.150533205
E1BIJ5	N4BP2	E1BIJ5	ENSBTAG00000000175	-0.406701341	-0.350006618	-0.056694723
Q2TBW5	ZNHIT2	bta:539138; Q2TBW5	ENSBTAG00000063864	0.134649527	0.21385441	-0.079204883
A0AAA9SAS6	HDAC8	A0AAA9SAS6	ENSBTAG00000046092	-0.573602639	-0.355094959	-0.21850768
Q2YDD3	ZFPL1	bta:613442; Q1JP84; Q2YDD3	ENSBTAG00000015479	0.019069963	-0.00443679	0.023506753
G8JL05	LPCAT3	G8JL05	ENSBTAG00000013127	0.063150933	0.107022432	-0.043871498
A0A3Q1M4C0	CEP85	A0A3Q1M4C0; bta:517520	ENSBTAG00000009579	-0.164316355	-0.140094253	-0.024222102
G5E690	GGA3	G5E690	ENSBTAG00000016128	0.417978775	0.34876428	0.069214495
A0A3Q1MGP4	CHD9	A0A3Q1MGP4	ENSBTAG00000002287	-0.331205908	-0.228570996	-0.102634912
G3X6H0	CEP170B	G3X6H0	ENSBTAG00000004802	-0.127991214	0.093904497	-0.221895711
A0AAA9SGX1	EMB	A0AAA9SGX1	ENSBTAG00000044010	0.059220423	0.157710287	-0.098489863
A0A0N4STN0	ATP5MG	A0A0N4STN0; bta:515696	ENSBTAG00000007332	-0.153772186	-0.27368165	0.119909464
F1MS34	RTKN	F1MS34	ENSBTAG00000000490	-0.168037892	-0.069634188	-0.098403704
A0A3Q1LNU2	DYRK1A	A0A3Q1LNU2	ENSBTAG00000001771	-0.017866421	-0.026883294	0.009016873
F6RFU1	SHF	F6RFU1	ENSBTAG00000020934	0.130727114	0.187989717	-0.057262603
A0AAA9SRA7	PWWP3A	A0AAA9SRA7	ENSBTAG00000030839	-0.125243921	0.112316877	-0.237560798
A0A3Q1NIL1	WWP2	A0A3Q1NIL1	ENSBTAG00000018421	-0.105749732	0.072553027	-0.178302759
A0AAA9SU76	TMEM126A	A0AAA9SU76	ENSBTAG00000000451	0.13131169	0.108569228	0.022742462
A0AAA9THQ6	SNX8	A0AAA9THQ6	ENSBTAG00000013708	-0.272966941	0.086443526	-0.359410467
Q0VCG0	LYRM4	bta:614011; Q0VCG0	ENSBTAG00000031432	0.103385241	-0.059011215	0.162396457
A0A3Q1MPJ7	CPPED1	A0A3Q1MPJ7	ENSBTAG00000007142	-0.019108823	-0.023164188	0.004055365
A0AAF6YT67	CDC23	A0AAF6YT67	ENSBTAG00000008759	0.001449217	0.047071834	-0.045622617
F1N332	ULK3	F1N332	ENSBTAG00000005838	-0.008173931	0.029580991	-0.037754922
Q02827	NDUFC2	bta:338046; Q02827; Q3SZR4		-0.227410496	-0.24765918	0.020248684
A0A3Q1LZP9	STARD7	A0A3Q1LZP9	ENSBTAG00000018372	-0.04425792	-0.190778317	0.146520397
Q2YDK1	ISG20L2	bta:515980; Q2YDK1	ENSBTAG00000001518	0.008998127	0.133531984	-0.124533857
A0AAA9TUG8	DNMBP	A0AAA9TUG8	ENSBTAG00000008939	0.057594215	0.248008895	-0.190414681
F1N582	ODR4	F1N582	ENSBTAG00000011946	-0.209250481	-0.289506617	0.080256137
E1B945	ANAPC4	E1B945	ENSBTAG00000000646	-0.431039421	0.024247546	-0.455286967
Q1RMV0	PEX5	A1L572; bta:514832; Q1RMV0	ENSBTAG00000010490	0.465663572	0.254421276	0.211242297
A0AAA9SUV0	B3GLCT	A0AAA9SUV0	ENSBTAG00000033412	-0.16807278	-0.181462157	0.013389377
P08037	B4GALT1	bta:281781; P08037; Q0VC05; Q8MIG0	ENSBTAG00000015249	0.034956876	-0.012924566	0.047881442
A0A3Q1N6J6	OSCP1	A0A3Q1N6J6	ENSBTAG00000007994	-0.080373416	0.106813674	-0.18718709
Q0VCR1	RTF2	bta:513536; Q0VCR1		-0.139072523	-0.560928809	0.421856285
Q3T0F7	MTPN	bta:541099; Q3T0F7	ENSBTAG00000007806	0.847301799	-0.09033232	0.937634119
A0AAA9S3D4	ASCC1	A0AAA9S3D4	ENSBTAG00000000392	-0.184917716	0.015413555	-0.200331271
A0A3Q1MXN1	SLC4A7	A0A3Q1MXN1	ENSBTAG00000018227	-0.019546975	0.032487853	-0.052034828
A0AAA9SH66	SGO2	A0AAA9SH66	ENSBTAG00000031579	0.147112883	0.036567961	0.110544922
A0AAA9S4B2	KIF16B	A0AAA9S4B2	ENSBTAG00000000939	-0.008243994	0.134635961	-0.142879956
G3N0C3				0.309009835	0.403033942	-0.094024108
A0A3Q1MEK8	PHKB	A0A3Q1MEK8	ENSBTAG00000004806	-0.137918947	-0.06513763	-0.072781317
A0A3Q1M5C4	TMEM33	A0A3Q1M5C4	ENSBTAG00000043958	-0.046208429	-0.047828525	0.001620096
A0AAA9S542	SPIRE1	A0AAA9S542	ENSBTAG00000010542	0.084509524	0.135087623	-0.050578099
A0A3Q1MIG2	AP1S1	A0A3Q1MIG2	ENSBTAG00000014468	-0.063911764	-0.03529333	-0.028618434
A1XEA0	EEF1A1	A1XEA0		0.40979393	0.487871843	-0.078077914
Q58D31	SORD	bta:508954; Q0II66; Q58D31		-0.099299341	0.255570029	-0.354869369
A0AAA9TEF0	MED14	A0AAA9TEF0	ENSBTAG00000011790	-0.093475153	-0.027691734	-0.065783419
A0A3Q1LZW1	PALM3	A0A3Q1LZW1; bta:527118	ENSBTAG00000046744	0.142326504	0.50192134	-0.359594836
A5PJJ5	LRRC14	A5PJJ5; bta:539952	ENSBTAG00000004969	-0.227841701	0.017569337	-0.245411038
A0AAA9T881	CD200	A0AAA9T881	ENSBTAG00000033319	0.025045129	-0.26489715	0.289942279
A6QLI5	MAGED4	A6QLI5; bta:100125231		0.181194399	-0.288919021	0.47011342
A0A3Q1LMF5	CYLD	A0A3Q1LMF5	ENSBTAG00000006291	0.092824033	0.12945693	-0.036632897
E1BIE5	UBE2Q1	E1BIE5	ENSBTAG00000007515	-0.116358714	-0.1428006	0.026441886
A0AAA9TER0	VPS26C	A0AAA9TER0	ENSBTAG00000004791	-0.191101446	-0.195221473	0.004120027
Q3ZBB0	SLC35B2	bta:767848; Q3ZBB0	ENSBTAG00000008076	0.1631652	0.091941703	0.071223497
A6H7E4	LARP6	A6H7E4; bta:787569		-0.076536577	-0.036167546	-0.040369031
A0A3Q1MZF8	PTPRS	A0A3Q1MZF8	ENSBTAG00000018052	-0.230763722	0.044540981	-0.275304703
Q5E963	ARPC5L	bta:613421; Q5E963	ENSBTAG00000015278	0.130469932	0.058181071	0.072288861
E1BGF8	GPAT3	bta:519739; E1BGF8	ENSBTAG00000017592	0.26375956	0.572578776	-0.308819216
A0AAA9U040	SNU13	A0AAA9U040	ENSBTAG00000006104	-0.182824392	-0.025168228	-0.157656164
F1MKN2	MPG	F1MKN2	ENSBTAG00000026422	-0.081427599	-0.031122517	-0.050305082
A0AAA9TFD6	PFDN1	A0AAA9TFD6	ENSBTAG00000051779	0.229904697	0.16725976	0.062644937
A0A3Q1N459	C2CD5	A0A3Q1N459	ENSBTAG00000001673	-0.039158376	0.158781002	-0.197939378
A0AAA9SNV4	MRPS17	A0AAA9SNV4	ENSBTAG00000007934	-0.287536447	-0.297566812	0.010030365
A0A3Q1LNP3	TRABD	A0A3Q1LNP3	ENSBTAG00000001931	-0.093203425	-0.157812506	0.064609081
A0A3Q1M1V0	NAGPA	A0A3Q1M1V0	ENSBTAG00000007896	0.052196211	0.050730802	0.001465409
A0AAA9TGQ0	CCDC90B	A0AAA9TGQ0	ENSBTAG00000007479	-0.005870588	0.067247341	-0.073117929
A0A452DIW5	WDR91	A0A452DIW5	ENSBTAG00000017905	-0.171536051	0.069843989	-0.24138004
G3MY50	ASMTL	G3MY50	ENSBTAG00000046514	-0.235840231	-0.078002512	-0.157837719
A0A3Q1M266	RCOR3	A0A3Q1M266	ENSBTAG00000007110	-0.226418082	-0.027393767	-0.199024315
Q1LZ79	GEMIN8	bta:515968; Q1LZ79	ENSBTAG00000010289	0.038680468	0.052355405	-0.013674937
A0AAA9RVP2	PEX6	A0AAA9RVP2	ENSBTAG00000005532	0.062973917	0	0.062973917
A5PK43	ERAL1	A5PK43; bta:523344		-0.74429824	-0.52953984	-0.2147584
A0A3Q1M6S6	DHTKD1	A0A3Q1M6S6	ENSBTAG00000000291	0.048626968	0.203905194	-0.155278225
P0CG53	UBB	bta:281370; O97577; P02248; P02249; P02250; P0CG53; P62990; P80169; Q01235; Q24K23; Q28169; Q28170; Q29120; Q3T0V5; Q3ZCE3; Q862C1; Q862F4; Q862M4; Q862T5; Q862X8; Q91887; Q91888	ENSBTAG00000017246	-0.168089745	-0.290323037	0.122233292
Q3ZBX7	ANKRD1	bta:510376; Q3ZBX7	ENSBTAG00000011734	-0.029901486	-0.390930883	0.361029397
Q1RML7	MAK16	bta:504683; Q1RML7	ENSBTAG00000033504	-0.066005185	-0.104023065	0.03801788
E1BF52	GFER	E1BF52	ENSBTAG00000017631	0.078171614	0.166708289	-0.088536675
A4FUD2	HSD17B7	A4FUD2; bta:505212	ENSBTAG00000005976	0.011813331	0.042370798	-0.030557467
A0A3Q1M4V2	SNRPG	A0A3Q1M4V2	ENSBTAG00000053210	0.190386535	0.102923694	0.087462841
A0A3Q1MYX0	RNPS1	A0A3Q1MYX0	ENSBTAG00000009969	-0.145299553	-0.048313938	-0.096985615
E1BAZ2	NDUFAF1	E1BAZ2	ENSBTAG00000010778	-0.186253171	-0.175735299	-0.010517872
Q5E9Y2	STX17	bta:534304; Q5E9Y2	ENSBTAG00000008016	0.004510775	0.007510142	-0.002999367
A0A3Q1LM43	DNAJB6	A0A3Q1LM43	ENSBTAG00000000771	0.236360024	0.209779425	0.026580599
Q3T0E1	EXOSC3	bta:533245; Q3T0E1	ENSBTAG00000018925	-0.03691532	-0.026050432	-0.010864888
A2VDZ7	CDC20	A2VDZ7; bta:515376		-0.13227246	-0.154402729	0.022130269
E1BGK1	ARID4A	E1BGK1	ENSBTAG00000018898	-0.173846824	-0.082043344	-0.09180348
A7YY24	IGBP1	A7YY24; bta:100296883	ENSBTAG00000007797	0.14231642	-0.215158586	0.357475005
E1BPS3	WDFY3	E1BPS3	ENSBTAG00000013495	-0.091539601	0.109387859	-0.20092746
Q02370	NDUFA2	bta:327698; Q02370; Q148D8; Q2VYC4	ENSBTAG00000015041	-0.245168923	-0.245168923	0
A0AAA9T860	PLXNA2	A0AAA9T860; bta:407770	ENSBTAG00000001173	0.047219689	-0.086546448	0.133766137
A0AAA9SU88	GBGT1	A0AAA9SU88	ENSBTAG00000030319	-0.256817269	-0.378619051	0.121801781
Q3T904	ATG9A	A5D7D0; bta:540482; Q3T904		0.427120792	0.092376992	0.3347438
A7YY74	GALNT4	A7YY74; bta:100125265		-0.409355353	-0.413899278	0.004543925
A0A3Q1MEP0				-0.481581347	-0.506892436	0.025311089
Q1RML9	PLA2G7	bta:282311; Q1RML9	ENSBTAG00000019315	-0.160387031	-0.09126267	-0.069124361
A0AAA9S3A1	RMDN2	A0AAA9S3A1	ENSBTAG00000032905	0.132669417	0.092230873	0.040438544
F1MLQ1	LOC524236	F1MLQ1	ENSBTAG00000034949	-0.209756266	-0.063689868	-0.146066399
A6QQL0	SLC15A4	A6QQL0; bta:510499	ENSBTAG00000009167	-0.209854368	-0.228106073	0.018251705
E1BNB3	SNIP1	E1BNB3	ENSBTAG00000016852	0	-0.159847929	0.159847929
Q9XT97	PSEN1	bta:282705; Q9XT97		0.316531954	0.299440052	0.017091902
A0A3Q1MD82	CDK16	A0A3Q1MD82	ENSBTAG00000016769	-0.285200062	-0.299460755	0.014260693
F1N555	GIMAP8	F1N555	ENSBTAG00000014402	-0.333095886	0.258734268	-0.591830154
A0AAA9TQ78	SSH3	A0AAA9TQ78	ENSBTAG00000031590	0.114215569	0.191620073	-0.077404504
Q2KIG1	ASF1A	bta:618099; Q2KIG1	ENSBTAG00000002058	0.243418618	0.288469453	-0.045050835
A0AAA9T6J5	ARHGAP32	A0AAA9T6J5	ENSBTAG00000015905	0.096507981	0.185633871	-0.08912589
A0AAA9SNB5	MRPL52	A0AAA9SNB5	ENSBTAG00000014822	-0.096118154	-0.134266729	0.038148575
A3KMX1	CHST14	A3KMX1; bta:511245		-0.248599316	-0.050578099	-0.198021216
A0A3Q1MHL9	NSMCE2	A0A3Q1MHL9	ENSBTAG00000009394	-0.557362276	-0.202267317	-0.355094959
F1MTK2	MEAK7	F1MTK2	ENSBTAG00000000946	-0.039782715	-0.038438798	-0.001343917
E1BGJ4	MAN2A2	E1BGJ4	ENSBTAG00000018905	0.021959253	0.36780554	-0.345846287
A0A3Q1MLJ3	PFKFB2	A0A3Q1MLJ3	ENSBTAG00000002126	-0.204530047	-0.097939616	-0.106590431
A0AAA9TPX1	CCS	A0AAA9TPX1	ENSBTAG00000004343	-0.140257886	-0.328165721	0.187907835
F1MPR9	NFRKB	bta:510304; F1MPR9	ENSBTAG00000017610	-0.100481393	0.043457956	-0.143939349
A0AAA9TP59	KCTD10	A0AAA9TP59	ENSBTAG00000002697	-0.134462512	-0.225926275	0.091463763
A0A3Q1MQE4	MAP2	A0A3Q1MQE4	ENSBTAG00000018130	-0.262737891	-0.154806525	-0.107931365
A2VE18	STX6	A2VE18; bta:510909	ENSBTAG00000020458	0.013269644	0.086242508	-0.072972864
F1MLH9	PAX8	bta:534661; F1MLH9	ENSBTAG00000019354	-0.096566146	-0.036525876	-0.06004027
A6H7F6	GOLPH3L	A6H7F6; bta:532555		0.192061345	0.090480746	0.1015806
A6QPM9	PTMS	A6QPM9; bta:613777; F1N7I3	ENSBTAG00000018451	0.398307861	0.152703261	0.2456046
A0A3Q1MLQ5	SLC2A1	A0A3Q1MLQ5	ENSBTAG00000009617	0.147701187	-0.274932989	0.422634176
A0AAA9S613	PLCG2	A0AAA9S613	ENSBTAG00000002103	0.12705191	0.179302139	-0.052250228
A0A3Q1LXC0	POLB	A0A3Q1LXC0	ENSBTAG00000000225	-0.050685366	0.308249996	-0.358935362
Q32PC9	ARL2BP	A4IFR7; bta:613462; Q32PC9	ENSBTAG00000004844	0.897943996	0.428533342	0.469410654
A0AAA9TN82	KIN	A0AAA9TN82	ENSBTAG00000013925	-0.086133256	-0.052636344	-0.033496912
F1N6E1	SAV1	bta:516557; F1N6E1	ENSBTAG00000001539	0.225465255	0.090415295	0.13504996
A0A3Q1MSK9	EPN2	A0A3Q1MSK9	ENSBTAG00000017771	-0.052779928	-0.23652275	0.183742822
A2VE40	GINS4	A2VE40; bta:616799	ENSBTAG00000017133	-0.003611254	0.109360559	-0.112971813
Q2KHX3	PRAF2	bta:508703; Q2KHX3		0.144242477	0.123077867	0.02116461
Q32L78	TRAPPC6B	bta:521470; Q32L78	ENSBTAG00000015023	-0.065365428	0.033166864	-0.098532292
Q3ZBV0	TSPAN13	bta:526460; Q3ZBV0		0.074546953	0.158079576	-0.083532623
F6RVL5	RGL2	F6RVL5	ENSBTAG00000016827	-0.100136671	0.119677206	-0.219813877
F1MBD5	SURF2	bta:526046; F1MBD5	ENSBTAG00000000046	0.294000757	0.31885162	-0.024850864
E1BF40	GPT2	E1BF40	ENSBTAG00000002980	-0.435374476	-0.421555453	-0.013819024
A0AAA9TA89	EXD2	A0AAA9TA89; bta:539532	ENSBTAG00000010052	-0.462866024	-0.583003648	0.120137623
A0AAA9T770				-0.073556607	-0.042813084	-0.030743523
A0AAA9SNY2	MOB1B	A0AAA9SNY2	ENSBTAG00000016290	0.02983523	-0.082715509	0.112550739
A0A3Q1LX29	DOP1A	A0A3Q1LX29	ENSBTAG00000000676	0.117975015	0.134803412	-0.016828397
A6QNS6	NID1	A6QNS6; bta:534319; F1MWN3	ENSBTAG00000007244	0.039737757	0.187211192	-0.147473435
A0AAA9SKB6	HAUS7	A0AAA9SKB6	ENSBTAG00000014095	-0.002871036	0.087631548	-0.090502584
A0AAA9TLK0	COMMD2	A0AAA9TLK0	ENSBTAG00000005068	0.156342029	0.221895711	-0.065553682
Q3SX24	FBXO6	bta:513023; Q3SX24	ENSBTAG00000014777	0.066406125	0.335736591	-0.269330467
A0A3Q1N9Q2	TACC2	A0A3Q1N9Q2	ENSBTAG00000010786	-0.060580066	-0.005403359	-0.055176707
Q3MHX1	COMMD7	bta:514295; Q3MHX1	ENSBTAG00000016427	0.567640054	0.347527918	0.220112136
F1N652	GOSR2	F1N652	ENSBTAG00000015958	0.146685733	0.105420957	0.041264775
Q7YR75	MRPL12	A5D9H8; bta:399560; Q7YR75	ENSBTAG00000000417	0.110089452	0.025200554	0.084888898
A0AAA9RWD4	JPH2	A0AAA9RWD4	ENSBTAG00000068398	-0.038805326	-0.072228328	0.033423002
Q3SZN5	POLE3	bta:510678; Q3SZN5	ENSBTAG00000000252	0.060798694	0.15589701	-0.095098315
Q32LE7	PDZD11	bta:515371; Q32LE7	ENSBTAG00000012860	0.160464672	0.221974309	-0.061509637
A6H732	TMX4	A6H732; bta:618015; F1MD03	ENSBTAG00000035323	-0.218009143	-0.074946094	-0.143063049
A0AAA9SKI8	PAPOLG	A0AAA9SKI8	ENSBTAG00000007414	0.047766419	0.015627573	0.032138846
F1MGU7	FGG	F1MGU7	ENSBTAG00000006745	-0.09133113	0.126350096	-0.217681227
A0A3Q1MY63	MGRN1	A0A3Q1MY63	ENSBTAG00000018999	-0.085259088	-0.044097264	-0.041161824
A0AAA9T2R6	ZNF143	A0AAA9T2R6	ENSBTAG00000016074	0.068641666	-0.068861858	0.137503524

Accession	Gene_Symbol	Gene_ID	Ensembl_Gene ID	log2(ratio(OV24H/OV0H))	log2(ratio(OV24R/OV0H))	log2(ratio(OV24H/OV24R))
A0A140T8D4	LGMN	A0A140T8D4	ENSBTAG00000046979	-0.045391903	-0.093960803	0.0485689
F6R1D7	LRATD2	F6R1D7	ENSBTAG00000026172	0.316839218	0.349102087	-0.032262869
F1MQ25	MRPL49	F1MQ25	ENSBTAG00000005075	-0.037625072	-0.06127634	0.023651268
E1BBB8	TRPS1	bta:537652; E1BBB8	ENSBTAG00000017694	0.144232873	-0.13289427	0.277127143
Q28203	CD40	A7YWS9; bta:286849; Q28203		0.030867567	-0.173517488	0.204385055
Q0VCK9	VMP1	bta:508631; Q0VCK9	ENSBTAG00000011623	-0.002724637	-0.16567803	0.162953393
E1BEW5	PPTC7	bta:525355; E1BEW5	ENSBTAG00000018332	0.052583332	-0.026201765	0.078785097
A0AAA9TXJ2	PRUNE2	A0AAA9TXJ2	ENSBTAG00000012991	-0.05048977	0.026472211	-0.076961982
A0AAA9T3N7	ZNF385B	A0AAA9T3N7	ENSBTAG00000003120	-0.22552758	-0.227970756	0.002443176
Q3T116	MRPL58	A7YWR4; bta:515465; Q3T116	ENSBTAG00000003303	-0.17807124	-0.093976148	-0.084095092
Q32KU6	TSPAN6	bta:514741; Q32KU6	ENSBTAG00000045550	0.139987717	0.108623546	0.031364171
O77627	JUN	O77627	ENSBTAG00000068635	0.147002412	0.041011717	0.105990696
A0AAA9RT10	FAM185A	A0AAA9RT10	ENSBTAG00000014879	-0.031231324	-0.077256432	0.046025108
Q2KII6	NGDN	bta:539602; Q2KII6	ENSBTAG00000009595	0.053129966	0.102933522	-0.049803556
A0A3Q1NN25	OPA3	A0A3Q1NN25	ENSBTAG00000019605	0.03124068	0.076868763	-0.045628083
Q0VC50	RTN4IP1	bta:506626; Q0VC50	ENSBTAG00000017554	-0.583838469	-0.271379574	-0.312458895
Q58D49	MMAB	bta:617636; Q2NL20; Q58D49		-0.223451125	-0.188297425	-0.0351537
A0AAA9T971	MTM1	A0AAA9T971	ENSBTAG00000014138	0.011658205	0.026099718	-0.014441512
F1MNB3	MIGA1	F1MNB3	ENSBTAG00000031044	-0.136016975	0.140327884	-0.276344859
A0AAA9TYF6	PTCD1	A0AAA9TYF6	ENSBTAG00000020440	-0.260664799	-0.011906767	-0.248758033
Q2KIQ7	SF3B6	bta:508876; Q2KIQ7	ENSBTAG00000003407	0.091084975	0.044020413	0.047064562
Q95KE5	MRPL43	bta:282277; Q28183; Q2KID5; Q95KE5	ENSBTAG00000003294	-0.126059826	0.069558043	-0.195617869
F1N6T6	TARBP1	F1N6T6	ENSBTAG00000004517	-0.144788345	0.098021033	-0.242809378
F1N196	TBC1D17	F1N196	ENSBTAG00000047035	0.00573637	0.057743673	-0.052007303
A0AAA9SFN1	SEC14L2	A0AAA9SFN1; bta:282469	ENSBTAG00000017404	-0.312475514	-0.114687132	-0.197788381
A0AAA9S2W2	ADIPOQ	A0AAA9S2W2	ENSBTAG00000059085	0.003153433	0.554118601	-0.550965168
A0AAA9SGT8	DIPK1A	A0AAA9SGT8	ENSBTAG00000002028	-0.406412253	-0.149460899	-0.256951354
A0AAA9T6S9	EXOSC1	A0AAA9T6S9	ENSBTAG00000012699	0.15682011	0.165450415	-0.008630305
A0A3Q1LS50	CYTH1	A0A3Q1LS50	ENSBTAG00000006663	-0.13556994	-0.245963738	0.110393798
Q2KJG8	BCKDK	bta:505005; Q2KJG8	ENSBTAG00000010524	-0.510194732	-0.485190726	-0.025004006
F1MY08	ATP23	bta:514014; F1MY08	ENSBTAG00000017543	-0.068936655	-0.068936655	0
F1MYX2	APOM	F1MYX2	ENSBTAG00000018833	0.22084197	0.344173523	-0.123331553
A0AAA9STJ0	TMEM201	A0AAA9STJ0	ENSBTAG00000003180	0.067410091	-0.084454939	0.15186503
A0A452DIB4	NDUFA6	A0A452DIB4	ENSBTAG00000009509	-0.209941901	-0.047670473	-0.162271429
Q1RMW0	PLSCR3	bta:510355; Q1RMW0	ENSBTAG00000017747	-0.134075754	0.090797657	-0.224873411
F1MEA6	DIS3L	F1MEA6	ENSBTAG00000003371	0.00900749	0.001505159	0.007502331
F6RVI7	MTO1	F6RVI7	ENSBTAG00000016839	-0.239602649	-0.061767409	-0.17783524
A7MB98	ZFP36L1	A7MB98; bta:614773	ENSBTAG00000025434	-0.014284226	0.151647706	-0.165931932
A5D9D4	MST01	A5D9D4; bta:535567; Q29S12	ENSBTAG00000026613	-0.191152377	-0.095899918	-0.095252459
A0AAF6DLX7	MAPRE3	A0AAF6DLX7	ENSBTAG00000008884	-0.005125032	0.097658608	-0.102783641
A0AAF6DLL6	PRKAR1B	A0AAF6DLL6	ENSBTAG00000046142	-0.114799782	0.006952761	-0.121752543
F1MK24	CNNM4	F1MK24	ENSBTAG00000019213	0.368613162	0.586030768	-0.217417606
Q0II29				0.345239653	0.223324696	0.121914957
A5PJE5	NFATC2IP	A5PJE5; bta:505998		-0.159016102	-0.145326748	-0.013689355
F1MET2	ERF	bta:789798; F1MET2	ENSBTAG00000000332	0.123048545	0.310289589	-0.187241045
A0AAA9SKI3	ANKRD28	A0AAA9SKI3	ENSBTAG00000024815	-0.139981199	0.076336708	-0.216317907
A0A3Q1LZC5	EDIL3	A0A3Q1LZC5; bta:538062	ENSBTAG00000044033	-0.397837242	-0.384990363	-0.01284688
Q2M2U4	RNASEH2C	bta:505618; Q2M2U4	ENSBTAG00000005577	-0.029458093	-0.058083934	0.028625841
Q5E994	CHMP1B	bta:512033; Q2KJ31; Q5E994		0.067887705	-0.022877705	0.090765464
A6QL68	CLYBL	A6QL68	ENSBTAG00000004165	-0.039451151	-0.036597154	-0.002853997
E1BKP6	CEP250	E1BKP6	ENSBTAG00000006021	0.503785592	0.330645312	0.17314028
A0AAA9TBD1	MGMT	A0AAA9TBD1	ENSBTAG00000050386	0.291408117	0.095443485	0.195964632
F6QAN5	NOCT	F6QAN5	ENSBTAG00000005998	0.013498999	0.138889916	-0.125390917
Q0VCM0	S100A16	bta:505679; Q0VCM0	ENSBTAG00000014204	-0.056143078	0.184534696	-0.240677774
A0AAA9RU55	FGB	A0AAA9RU55	ENSBTAG00000022120	-0.403185077	-0.303532827	-0.09965225
A0A3Q1N8K4	FAM151B	A0A3Q1N8K4	ENSBTAG00000017632	-0.072850565	0.052321035	-0.125171601
A7MB60	TTC21B	A0A3Q1M8G2; A7MB60; bta:527412; F6PR93	ENSBTAG00000016512	-0.574545885	0.034433497	-0.608979382
A0A3Q1LXB2	TAF6L	A0A3Q1LXB2	ENSBTAG00000009484	-0.028758993	0.315586568	-0.344345561
A0A3Q1MYE6	INTS2	A0A3Q1MYE6	ENSBTAG00000012070	-0.077382531	0.024898519	-0.10228105
F1N2J0	SMG8	F1N2J0	ENSBTAG00000003365	-0.526636828	-0.201251669	-0.325385159
Q6T8E9	CCNT1	bta:407194; Q6T8E9	ENSBTAG00000005861	-0.311402915	-0.258454035	-0.05294888
E1BKS1	SDC4	bta:508133; E1BKS1	ENSBTAG00000015127	-0.067942141	-0.185214872	0.117272731
A0AAA9T9X0	YAF2	A0AAA9T9X0	ENSBTAG00000015025	-0.090982655	-0.040077439	-0.050905216
A0AAA9U2N6	PIK3R2	A0AAA9U2N6	ENSBTAG00000002350	-0.257871094	-0.207662354	-0.050208741
Q2TBI6	MRPL32	bta:532842; Q2TBI6	ENSBTAG00000000991	-0.084309967	-0.041539195	-0.042770772
F1MBB0	RELB	F1MBB0	ENSBTAG00000038428	0.07277744	0.01408768	0.05868976
P25417	CSTB	bta:512805; P25417; Q32PC6		0.602036014	0.257195394	0.344840621
E1BBR9	SPTY2D1	bta:539967; E1BBR9	ENSBTAG00000001660	0.277780991	0.195318831	0.08246216
A0AAF7A7H1	MAN2B1	A0AAF7A7H1	ENSBTAG00000006241	0.002613579	0.402557929	-0.39994435
E1BCH5	LATS1	bta:535935; E1BCH5	ENSBTAG00000015076	-0.257857102	-0.176937107	-0.080919995
Q2T9Y0	RSRC1	bta:509437; Q2T9Y0		0.062404035	-0.044615572	0.107019607
A0AAA9TCS9	AP5Z1	A0AAA9TCS9	ENSBTAG00000012197	0.019593262	-0.054953691	0.074546953
Q24K21	DPCD	bta:516908; Q24K21	ENSBTAG00000003578	-0.261599602	0.106060476	-0.367660078
A0AAA9THN5	HOMER3	A0AAA9THN5; bta:508124	ENSBTAG00000000813	0.021032372	-0.010124217	0.03115659
A0AAA9SV91	ZMIZ1	A0AAA9SV91	ENSBTAG00000019313	-0.318249553	-0.187312231	-0.130937322
A0AAA9RXP7	UQCRQ	A0AAA9RXP7	ENSBTAG00000009479	0.001480447	0.058063975	-0.056583528
A0A3Q1LFT9	HYCC1	A0A3Q1LFT9	ENSBTAG00000004575	0.012123559	0.006074514	0.006049045
G3N1P6	ANAPC2	G3N1P6	ENSBTAG00000046223	-0.024635733	0.279479157	-0.30411489
F6Q1G9	RAB24	F6Q1G9	ENSBTAG00000025425	0.102615066	0.058893689	0.043721377
F1MQK6	UBR3	F1MQK6	ENSBTAG00000020296	0.054107405	0.057895666	-0.00378826
A7YSY2	AFG2B	A7YSY2; bta:533070	ENSBTAG00000018279	0.253705168	0.132422328	0.121282839
A0AAA9TRS1	RIOK3	A0AAA9TRS1	ENSBTAG00000010042	0.417593204	0.512696108	-0.095102903
A0AAA9SXL2	LAMC3	A0AAA9SXL2	ENSBTAG00000017473	-0.153869917	0.020518675	-0.174388592
A7MBG2	ABHD17B	A7MBG2; bta:781153	ENSBTAG00000006816	-0.102716921	-0.323517842	0.220800921
A0A3Q1M7N0	NUBP2	A0A3Q1M7N0	ENSBTAG00000016561	0.018992715	0.017540581	0.001452134
A0A3Q1N0F9	RAPGEF2	A0A3Q1N0F9	ENSBTAG00000012450	-0.066901957	0.049652301	-0.116554257
A0A3S5ZPS3	ACOT4	A0A3S5ZPS3; bta:511431	ENSBTAG00000002843	-0.059280938	-0.086156644	0.026875706
A0JN35	ENC1	A0JN35; bta:617091		-0.291641966	-0.573009698	0.281367732
A0AAA9SB62	CHST12	A0AAA9SB62	ENSBTAG00000020898	-0.137206092	-0.068531382	-0.06867471
E1B770	LPCAT2	E1B770	ENSBTAG00000019272	-0.538095896	-0.492123782	-0.045972114
A0AAA9SLH3	ISCU	A0AAA9SLH3	ENSBTAG00000011787	0.187360116	0.067185242	0.120174874
F1N1Q7	PREX1	F1N1Q7	ENSBTAG00000015609	-0.010397847	0.224980216	-0.235378063
A0AAA9T4H0	TIMM17A	A0AAA9T4H0	ENSBTAG00000047059	-0.581465056	-0.468946663	-0.112518394
A0A3Q1LUT0	SLC39A14	A0A3Q1LUT0	ENSBTAG00000019225	0.113853475	0.149404228	-0.035550753
A0AAA9T180	CYB5B	A0AAA9T180	ENSBTAG00000002412	0.193045326	0.137357951	0.055687375
A0AAF6DLG6				-0.209368782	-0.080711799	-0.128656983
A7YWK4	MED19	A7YWK4; bta:509245; F1MQL1	ENSBTAG00000002389	0.128845372	0.061464357	0.067381016
A7Z060	OBI1	A7Z060; bta:782050	ENSBTAG00000000869	-0.04032856	-0.135037144	0.094708584
A0A3S5ZPC1	SYPL1	A0A3S5ZPC1	ENSBTAG00000019794	0.043197689	0.147171411	-0.103973722
A0AAA9TQC8	RPL22L1	A0AAA9TQC8	ENSBTAG00000007394	-0.128274022	-0.132988716	0.004714694
Q2HJE9	TIMM17B	bta:540765; Q2HJE9		0.075021736	0.097228871	-0.022207135
F1MI02	FCHSD2	F1MI02	ENSBTAG00000019062	0.156676335	0.033052878	0.123623456
A0AAA9TGR7	CMIP	A0AAA9TGR7	ENSBTAG00000013524	-0.339031472	-0.076702368	-0.262329104
E1BKC3	TNFAIP3	bta:508105; E1BKC3	ENSBTAG00000000436	0.046448923	0.023411387	0.023037536
Q01321	NDUFA4	bta:327704; Q01321; Q3ZC70	ENSBTAG00000011145	0.059094748	-0.111433513	0.170528261
Q3ZC25	TMEM106B	bta:508903; Q3ZC25	ENSBTAG00000019750	-0.019072733	0.19112173	-0.210194463
A0A3Q1LUU0	INPP5B	A0A3Q1LUU0	ENSBTAG00000024107	-0.031302304	0.20316295	-0.234465254
A0A3Q1ME94	BANF1	A0A3Q1ME94	ENSBTAG00000031875	0.990015911	0.527450042	0.462565869
Q32LE1	YEATS4	bta:615330; Q32LE1	ENSBTAG00000002256	0.109624491	-0.00475353	0.114378021
A0AAA9TH35	COPS8	A0AAA9TH35	ENSBTAG00000005854	0.046355636	0.027702929	0.018652707
A0AAA9SG08	DYNLRB1	A0AAA9SG08	ENSBTAG00000006134	0.638390117	0.001919754	0.636470363
A0A3Q1MDC6	NCSTN	A0A3Q1MDC6	ENSBTAG00000004334	-0.076442504	0.16945667	-0.245899174
A5PKM1	ZNRF2	A5PKM1; bta:615645	ENSBTAG00000000260	-0.187085553	-0.430281211	0.243195659
Q2YDG6	NUP42	bta:515127; F6QMH3; Q2YDG6	ENSBTAG00000016014	0.280914007	0.236519887	0.044394119
A0AAA9T3C7				-0.186063648	-0.324464331	0.138400682
Q0VFX9	GGACT	bta:512596; Q0VFX9		-0.047461354	0.081683798	-0.129145151
E1BB53	TEP1	E1BB53	ENSBTAG00000001179	-0.138102277	-0.045610597	-0.09249168
A7MB12	UTP15	A7MB12; bta:526343	ENSBTAG00000006843	-0.072837988	0.223294106	-0.296132094
Q08DC9	CEND1	bta:504370; Q08DC9	ENSBTAG00000001228	0.277423674	0.296708105	-0.019284431
A0AAF6DLK5	PIGG	A0AAF6DLK5	ENSBTAG00000005964	0.183221824	0.023424346	0.159797479
A0AAF6Z202	GAPDHS	A0AAF6Z202	ENSBTAG00000015917	0.187707155	0.209186883	-0.021479727
A6QPI6	TOMM22	A6QPI6; bta:510780	ENSBTAG00000010157	0.25403658	-0.006961148	0.260997728
A0AAA9SYS8	GATB	A0AAA9SYS8	ENSBTAG00000013914	0.00314999	0.103419673	-0.100269683
F1N230	ORC2	F1N230	ENSBTAG00000031473	-0.178264302	-0.069558043	-0.108706259
A0A3Q1MR59	ELL	A0A3Q1MR59	ENSBTAG00000003587	0.004854839	0.137503524	-0.132648684
A0AAA9T2P8	C1GALT1	A0AAA9T2P8	ENSBTAG00000004995	-0.039438311	-0.164666329	0.125228018
Q0NXR6	ACAD8	A1A4N5; bta:512070; Q0NXR6	ENSBTAG00000012937	-0.642843417	-0.297246416	-0.345597001
A0A3Q1LKV3	RBM23	A0A3Q1LKV3	ENSBTAG00000038333	0.041730565	-0.241863706	0.28359427
Q8HXG5	NDUFB11	bta:404161; Q3T137; Q8HXG5	ENSBTAG00000068665	0.019587805	-0.084337356	0.103925161
Q08DH2	TPST1	bta:513004; Q08DH2	ENSBTAG00000000390	0.227410496	0.084008937	0.143401559
A7Z024	RP2	A7Z024	ENSBTAG00000005496	0.203917611	0.254980771	-0.051063161
F1N783	CHSY3	F1N783	ENSBTAG00000014661	-0.157775919	-0.075124739	-0.08265118
P68401	PAFAH1B2P68402	bta:282514; O00687; P68401; Q29459; Q3ZBB8	ENSBTAG00000005627	0.162667447	0.113327684	0.049339763
Q56JX5	RPS25	bta:282052; Q56JX5	ENSBTAG00000013866; ENSBTAG00000027772	-0.145280874	-0.228752586	0.083471712
Q1KLV9	TRAF6	Q1KLV9		-0.081887201	-0.03391408	-0.047973122
Q2HJ74	GATM	bta:414732; Q2HJ74	ENSBTAG00000005586	-0.081794091	-0.034488376	-0.047305715
A0A3Q1MBF5	MRPS2	A0A3Q1MBF5	ENSBTAG00000005688	-0.468279521	-0.293141161	-0.17513836
A0A3Q1M319	AMOTL1	A0A3Q1M319	ENSBTAG00000018945	-0.056641997	0.173331603	-0.2299736
A0A3Q1M4H2	ADAM9	A0A3Q1M4H2	ENSBTAG00000017455	0.112005026	0.211077108	-0.099072083
F1MFD9	OSBPL10	F1MFD9	ENSBTAG00000009194	0.04940762	-0.016394438	0.065802058
A0A452DKP2	LOC100299281	A0A452DKP2	ENSBTAG00000046172	-1.031560213	-0.465716664	-0.565843548
A0A3Q1LPS2	R3HDM1	A0A3Q1LPS2	ENSBTAG00000013302	-0.153501998	0.149709138	-0.303211136
F2Z4I9	PAM16	F2Z4I9	ENSBTAG00000009200	0.050057707	0.082961449	-0.032903742
A0AAA9SNL2	MPHOSPH8	A0AAA9SNL2	ENSBTAG00000003922	0.008109606	-0.06486984	0.072979445
A0AAF7A0S6	KIAA1191	A0AAF7A0S6	ENSBTAG00000011608	0.055179559	0.127935902	-0.072756342
A0AAA9TPR6	RWDD1	A0AAA9TPR6	ENSBTAG00000009689	0.099826804	-0.054895791	0.154722595
F1MPW9	C2CD3	F1MPW9	ENSBTAG00000002907	-0.116050922	-0.001377275	-0.114673647
A0A452DIG5	MGST3	A0A452DIG5	ENSBTAG00000010265	0.178795099	-0.001313332	0.180108431
F1MC83	HAX1	F1MC83	ENSBTAG00000013025	0.171198101	0.041960532	0.12923757
A0AAA9RSU6	WDTC1	A0AAA9RSU6	ENSBTAG00000053588	-0.002996252	-0.161345277	0.158349025
A0A3Q1MWL7	DENND3	A0A3Q1MWL7	ENSBTAG00000006132	-0.223824377	0.266913993	-0.49073837
F1MWW9	CDCA5	F1MWW9	ENSBTAG00000026971	0.183832575	-0.278655386	0.462487961
A0AAF7AMQ9	S100A2	A0AAF7AMQ9	ENSBTAG00000037651	0.967060326	0.496549491	0.470510835
A0AAF6Z0W9	FBXO9	A0AAF6Z0W9	ENSBTAG00000015083	-0.201566194	-0.149599102	-0.051967092
A4FUX6	MCC	A4FUX6; bta:616704; E1BC92	ENSBTAG00000035858	0.07308235	0.182281121	-0.109198771
A0A452DJ92	ZFYVE26	A0A452DJ92	ENSBTAG00000014334	-0.170618772	0.006919414	-0.177538186
P0C2B6	MRPL51	A7MB58; bta:513622; P0C2B6	ENSBTAG00000014729	-0.269326629	-0.091563351	-0.177763278
E1BFT9	ANKS3	A0A8J8XSB4; bta:528545; E1BFT9; M5FMW8	ENSBTAG00000006593	-0.027928744	0.028826035	-0.056754778
Q3SYW5	AZI2	bta:767932; Q3SYW5	ENSBTAG00000004117	0.007231569	-0.220460456	0.227692025
A0AAA9RZM4	ANTXR2	A0AAA9RZM4	ENSBTAG00000014324	0.167626618	0.167626618	0
F1MCE2	TBCEL	bta:540781; F1MCE2	ENSBTAG00000018213	0.272961246	0.443801953	-0.170840708
Q32LN1	UBE2H	bta:539313; Q32LN1	ENSBTAG00000000911	0.417416222	0.228501231	0.188914991
A0AAA9RXC3	RRM2B	A0AAA9RXC3	ENSBTAG00000021208	0.507289446	0.451998861	0.055290585
A0A3Q1M534	RBSN	A0A3Q1M534	ENSBTAG00000013725	-0.266432982	-0.320161169	0.053728186
E1BIU5	FBXO42	bta:100848477; E1BIU5	ENSBTAG00000008510	0.027985774	0.112958026	-0.084972252
E1BNQ3	RAP2A	E1BNQ3	ENSBTAG00000020412	0.379643593	0.414577383	-0.03493379
E1BBM0	MRM3	bta:538817; E1BBM0	ENSBTAG00000019162	-0.155243382	-0.099973783	-0.055269598
A0A452DIY2	MTIF3	A0A452DIY2	ENSBTAG00000020530	-0.109866588	-0.062667617	-0.047198971
F1MIQ3	LIN9	F1MIQ3	ENSBTAG00000033267	-0.047581065	-0.027798452	-0.019782612
A0A3Q1MKP5	PMS1	A0A3Q1MKP5	ENSBTAG00000018795	-0.031813749	0.032666189	-0.064479938
A0AAA9TUI5	NME6	A0AAA9TUI5	ENSBTAG00000003025	-0.241776785	-0.005646563	-0.236130222
A0A3Q1MWF7				-0.060150143	-0.191666781	0.131516637
A0A452DIT0				0.709484858	0.568536953	0.140947905
P23935	NDUFA5	bta:327714; P23935; Q32P63	ENSBTAG00000009334	-0.032230859	0.132921111	-0.16515197
A0AAA9RYE9	PNPLA8	A0AAA9RYE9	ENSBTAG00000024387	-0.5154774	-0.507959431	-0.007517969
Q3ZBM4	ITGB1BP1	bta:513531; Q3ZBM4	ENSBTAG00000013063	-0.305656926	-0.037295295	-0.268361631
Q2TBR9	LLPH	bta:510518; Q2TBR9	ENSBTAG00000002004	-0.0284801	-0.049831156	0.021351056
A0AAF6Z072	TNFAIP8	A0AAF6Z072	ENSBTAG00000014551	0.223198847	0.264915364	-0.041716518
F6QQE8	CYP27A1	F6QQE8	ENSBTAG00000013489	-0.372983876	-0.39549867	0.022514794
A0AAA9T2U9	THNSL1	A0AAA9T2U9; bta:788561	ENSBTAG00000009117	-0.013674937	-0.012301595	-0.001373341
A0A3Q1MDV1	SUFU	A0A3Q1MDV1	ENSBTAG00000021068	0.109662044	0.03948675	0.070175294
Q32LH7	CYB5R4	bta:533608; Q32LH7	ENSBTAG00000013591	0.199057498	0.224616401	-0.025558902
F1MD46	RB1	F1MD46	ENSBTAG00000006640	0.243150982	0.359081093	-0.115930112
Q95142	PDE6D	bta:281976; Q95142	ENSBTAG00000019480	-0.006886386	0.068425141	-0.075311527
A7E303	COL8A1	A7E303; bta:538564	ENSBTAG00000013662	0.025873587	-0.060265904	0.086139491
A0A3Q1LWA8	MYO9A	A0A3Q1LWA8	ENSBTAG00000007433	0	0.095706355	-0.095706355
G3MZ59	TTC9	G3MZ59	ENSBTAG00000045604	-0.191932108	-0.122357632	-0.069574476
E1BL00	PWP2	E1BL00	ENSBTAG00000007105	-0.165337732	-0.272252936	0.106915204
E1BIZ2	SSH1	E1BIZ2	ENSBTAG00000018825	-0.11790949	-0.027711681	-0.090197809

Accession	Gene_Symbol	Gene_ID	Ensembl_Gene ID	log2(ratio(OV24H/OV0H))	log2(ratio(OV24R/OV0H))	log2(ratio(OV24H/OV24R))
A0AAA9SS15	BRAF	A0AAA9SS15	ENSBTAG00000021761	0.099040328	0.172069537	-0.07302921
Q17QJ6	BCL10	bta:540824; Q17QJ6	ENSBTAG00000013851	0.162119312	0.008553553	0.153565759
F1MUX1	MBD4	F1MUX1	ENSBTAG00000021645	-0.511027319	-0.121375139	-0.389652181
F1N5L6	DSC3	F1N5L6	ENSBTAG00000015238	-0.07603226	-0.007735649	-0.068296612
A0AAF6YIA2	HACE1	A0AAF6YIA2	ENSBTAG00000001533	-0.071719291	0.004191853	-0.075911144
F1MG06	RPTOR	F1MG06	ENSBTAG00000002883	0.012454999	0.015208234	-0.002753236
F1MRF7	GAR1	F1MRF7	ENSBTAG00000000748	0.068288438	0.227378824	-0.159090386
F6QB70	TREX1	F6QB70	ENSBTAG00000008406	-0.527323875	-0.488304631	-0.039019244
Q3SZB0	B3GAT3	bta:404162; F1N6Z1; Q3SZB0		0.100338621	0.071966122	0.028372498
A0AAA9SSU9	POGLUT3	A0AAA9SSU9	ENSBTAG00000008709	0.130754404	0.138088914	-0.00733451
A0A3Q1LYQ7	SRP9	A0A3Q1LYQ7	ENSBTAG00000033322	0.086364675	0.005730673	0.080634002
A0A3Q1LHP2	SCHIP1	A0A3Q1LHP2	ENSBTAG00000014960	-0.003036189	-0.416723482	0.413687293
A0A3Q1MPM1	NEU1	A0A3Q1MPM1	ENSBTAG00000005674	-0.050835965	0.100710507	-0.151546472
A0A3Q1M8A8	TDRD3	A0A3Q1M8A8	ENSBTAG00000009308	-0.040971781	0	-0.040971781
Q3ZCE0	LSM8	bta:613630; Q3ZCE0		0.122920614	0.01711033	0.105810283
A3KN19	FAM83D	A3KN19; bta:508561		0.094804366	-0.189848114	0.28465248
Q32LC1	RPP38	bta:511723; G5E591; Q32LC1	ENSBTAG00000063884	0.135455248	0.177410564	-0.041955316
O77480	MTFMT	bta:286855; O77480	ENSBTAG00000021630	-0.193003556	-0.177314182	-0.015689374
E1BDJ7	DNAJC25	bta:535430; E1BDJ7	ENSBTAG00000030960	0.210246991	0.135145536	0.075101455
A0AAA9SDA7	GRK6	A0AAA9SDA7	ENSBTAG00000043994	0.070454199	0.058730202	0.011723997
A0AAF6Z2U9	C3H1orf52	A0AAF6Z2U9	ENSBTAG00000016326	0.248930662	0.043816232	0.20511443
A0AAA9T279	APTX	A0AAA9T279	ENSBTAG00000013947	-0.009026276	0.131766068	-0.140792344
A0A3Q1M591	RAB43	A0A3Q1M591	ENSBTAG00000053386	0.120842162	-0.087548474	0.208390636
A0AAA9SL03	POU2F1	A0AAA9SL03	ENSBTAG00000024534	-0.03378172	-0.043785589	0.010003869
F1MP23	FZD6	F1MP23	ENSBTAG00000010529	-0.330425272	0.125127387	-0.455552659
A0A3Q1MDF2	REEP4	A0A3Q1MDF2	ENSBTAG00000009504	-0.119243345	-0.02931084	-0.089932505
F1MW97	HGH1	F1MW97	ENSBTAG00000039705	-0.10507579	-0.028428408	-0.076647382
A0AAA9TS87	DHDH	A0AAA9TS87	ENSBTAG00000013337	0.207663643	0.311300459	-0.103636816
Q32KX8	METTL26	bta:514636; Q32KX8		-0.097228871	-0.098801291	0.00157242
E1B9U5	FPGT	bta:100138313; E1B9U5	ENSBTAG00000038480	-0.410024789	-0.207788564	-0.202236225
A0A3Q1LYV4	TPD52L1	A0A3Q1LYV4	ENSBTAG00000003336	0.006513307	0.040762869	-0.034249562
F1N6M9	DVL1	bta:523325; F1N6M9	ENSBTAG00000010696	0.192098499	0.148063095	0.044035404
Q3ZBY7	DEGS1	bta:507290; Q3ZBY7	ENSBTAG00000012705	-0.170875456	-0.264555434	0.093679978
A0AAF6YK00	CIAO3	A0AAF6YK00	ENSBTAG00000002484	0.306964556	0.246843564	0.060120992
F1N7S0	TULP3	F1N7S0	ENSBTAG00000015879	0.28776843	0.309774972	-0.022006542
A0AAA9SAG7	CMSS1	A0AAA9SAG7	ENSBTAG00000013663	-0.080170349	0.116076966	-0.196247315
A0A3Q1N6C7	CPSF4	A0A3Q1N6C7	ENSBTAG00000002090	-0.103504576	-0.124090348	0.020585772
Q2HJ95	ZDHHC6	bta:514158; Q2HJ95	ENSBTAG00000006713	0.178122154	0.091084975	0.08703718
F1MXA4	ADD2	F1MXA4	ENSBTAG00000009076	-0.01509109	0.066021969	-0.081113059
A0AAF6DMH1	RPP14	A0AAF6DMH1	ENSBTAG00000019987	-0.1890038	0.001500463	-0.190504263
Q2KI83	DNAJC17	bta:524795; Q2KI83	ENSBTAG00000013480	-0.389448671	-0.075452228	-0.313996442
A0AAF6YPG3	DRAP1	A0AAF6YPG3	ENSBTAG00000006199	0.274327061	0.270397794	0.003929267
F6R223	RFK	F6R223	ENSBTAG00000019345	0.462756568	0.329232896	0.133523673
A0A4Y5NDR9	AAMDC	A0A4Y5NDR9		-0.018360537	-0.109292145	0.090931608
A0JN40	KIF3C	A0JN40; bta:777770	ENSBTAG00000019138	0.066506361	-0.009756217	0.076262578
E1B7F6	PLXNB1	E1B7F6	ENSBTAG00000013407	0.108769091	0.146799499	-0.038030407
A2VE20	UBE2G1	A2VE20; bta:613676	ENSBTAG00000014433	0.040560363	0.02620507	0.014355293
A0AAF7A1V8	FOXP1	A0AAF7A1V8	ENSBTAG00000016533	-0.076255104	-0.117997209	0.041742106
A5PK70	NME3	A0A8J8YPU5; A5PK70; bta:515663; M5FKE7	ENSBTAG00000016552	-0.088677158	-0.049388027	-0.039289131
A0AAF6DLM2	SLX9	A0AAF6DLM2	ENSBTAG00000017010	-0.26858324	-0.201772322	-0.066810918
A7MBI8	NUDT9	A7MBI8; bta:517589	ENSBTAG00000011354	0.324398162	0.393242093	-0.068843931
F6RW79	USP11	F6RW79	ENSBTAG00000016772	-0.362390562	-0.065559116	-0.296831445
Q2KI48	PTTG1IP	bta:617245; Q2KI48	ENSBTAG00000021771	-0.278850742	-0.294602407	0.015751666
A0AAF6ZUW3	NFYC	A0AAF6ZUW3	ENSBTAG00000021943	0.068975614	0.141223961	-0.072248347
A0AAA9S8D8	PLSCR2	A0AAA9S8D8	ENSBTAG00000022227	-0.195943506	0.066356891	-0.262300397
A2VE26	EXTL3	A2VE26; bta:783970; F1MYW6	ENSBTAG00000008844	-0.239187664	-0.147557188	-0.091630475
F1MWT0	MAN2C1	F1MWT0	ENSBTAG00000007368	-0.272443922	-0.019439881	-0.253004041
A0A3Q1MB37	ACSS2	A0A3Q1MB37	ENSBTAG00000013303	-0.012095325	0.061567823	-0.073663148
A6QR70	WRNIP1	A6QR70; bta:788824; F1MGL0	ENSBTAG00000006166	0.073637319	0.151567168	-0.077929849
Q4JIJ3	MTR	bta:280869; Q4JIJ3		0.04143409	0.359564464	-0.318130374
A0AAF6DME0	AP4B1	A0AAF6DME0	ENSBTAG00000003614	-0.302011095	-0.079842955	-0.222168139
A0A3Q1NCP3	SHROOM3	A0A3Q1NCP3; bta:100139141	ENSBTAG00000019633	-0.349163087	-0.239150419	-0.110012668
A6H784	SCO2	A6H784; bta:100125923	ENSBTAG00000057697	-0.173993169	-0.038665109	-0.13532806
A0A3Q1LF83	RYBP	A0A3Q1LF83	ENSBTAG00000022689	0.369477446	0.202290527	0.167186919
A0AAA9RS78	PARL	A0AAA9RS78	ENSBTAG00000014460	0.082064537	0.019899557	0.06216498
Q0V7P2	PIA1	bta:786612; Q0V7P2	ENSBTAG00000021026	-0.668170915	-0.363476295	-0.30469462
A0A3Q1MUV9	INHCA	A0A3Q1MUV9	ENSBTAG00000023411	0.225144463	0.857315922	-0.632171459
A0A3Q1N1I0	IQSEC2	A0A3Q1N1I0	ENSBTAG00000014945	-0.116292135	0.164794999	-0.281087134
A0AAF7AEL2	GPR108	A0AAF7AEL2	ENSBTAG00000017291	-0.132060852	-0.256815883	0.124755031
A0AAA9TVV3	CASP9	A0AAA9TVV3	ENSBTAG00000002472	-0.082540813	-0.108252891	0.025712077
A7MB76	TRAPPC13	A7MB76; bta:511108		0.04018909	0.109823278	-0.069634188
A0A024QYS0	PTEN	A0A024QYS0; bta:540786		-0.092945004	-0.254183488	0.161238484
A0A3Q1LST8	SP3	A0A3Q1LST8	ENSBTAG00000000176	0.072260435	0.09367639	-0.021415955
A4IFL0	TIMM23	A4IFL0; bta:509841	ENSBTAG00000011694	-0.319480774	-0.40933353	0.089852755
F6Q6K1	CHEK1	F6Q6K1	ENSBTAG00000017582	-0.060464641	-0.222168139	0.161703498
A4IFF3	TTC9C	A4IFF3; bta:786577	ENSBTAG00000000773	0.203572533	0.146937341	0.056635192
A1A4Q4	TMA7	A1A4Q4; bta:615251	ENSBTAG00000053755	0.171227661	-0.156244878	0.327472538
A0AAA9TSS4	DDX28	A0AAA9TSS4	ENSBTAG00000004290	-0.686446166	-0.514241862	-0.172204305
A0AAA9RYE8	SUMF2	A0AAA9RYE8	ENSBTAG00000008190	-0.337459247	-0.13804094	-0.199418307
A0AAA9RVN6	CIRBP	A0AAA9RVN6	ENSBTAG00000066789	-0.829487079	-0.298827681	-0.530659399
A7E3T1	CD151	A7E3T1	ENSBTAG00000019569	-0.542086665	-0.431827157	-0.110259508
A0AAA9TRT2	GOLGA1	A0AAA9TRT2	ENSBTAG00000015286	0.113341473	0.279699859	-0.166358386
E1BH71	DTX3L	bta:515051; E1BH71	ENSBTAG00000009933	-0.379764265	-0.062550271	-0.317213994
Q58CY6	PRXL2B	A4FUH8; bta:617001; Q58CY6		0.016036963	0.106383465	-0.090346502
A5D791	EIF2AK3	A5D791; bta:535820; F1MCN0	ENSBTAG00000000184	-0.007948753	0.097067418	-0.105016171
F1MLY3	DCP1B	F1MLY3	ENSBTAG00000030285	0.064678058	0.146638149	-0.081960091
A0AAA9SRY3	TENT4B	A0AAA9SRY3	ENSBTAG00000007942	-0.164870412	0.072909579	-0.237779991
Q2HJF6	SRA1	bta:780787; Q2HJF6	ENSBTAG00000001449	0.149244749	0.057206857	0.092037891
A0AAA9SI99	VRK2	A0AAA9SI99	ENSBTAG00000033801	-0.300375318	-0.188716085	-0.111659233
A0AAA9TII1	SHROOM4	A0AAA9TII1; bta:519045	ENSBTAG00000002996	-0.336475406	-0.642822688	0.306347282
F1MEQ5	DARS2	F1MEQ5	ENSBTAG00000004358	-0.026940543	0.210098654	-0.237039197
F1N2J7				0.043154614	0.174460763	-0.131306149
A0A3Q1LWS9	HMGN5	A0A3Q1LWS9	ENSBTAG00000001877	-0.001427704	0.07912865	-0.080556354
F1MC02	MGLL	F1MC02	ENSBTAG00000018248	-0.661047491	-0.434111145	-0.226936346
F1MUR7	RETREG2	bta:507475; F1MUR7	ENSBTAG00000012620	0.177970115	0.392768335	-0.21479822
F1N588	SMPD2	F1N588	ENSBTAG00000008145	0.010470617	0.013448316	-0.0029777
A0A3Q1MDD9				-0.117071097	-0.027242887	-0.089828209
A0A3Q1M222	RBM42	A0A3Q1M222	ENSBTAG00000013440	0.024193329	0.014564628	0.009628701
Q3MHJ2	MAEA	A7E3P9; bta:511956; Q3MHJ2	ENSBTAG00000012575	-0.372122525	0.00391505	-0.376037575
A0A3Q1LMS4	KMT2D	A0A3Q1LMS4	ENSBTAG00000014429	0.073768867	-0.102407149	0.176176016
Q9TTA5	SMARCAL1	bta:338072; F1MDP3; Q9TTA5		-0.091257745	-0.122547973	0.031290228
A0AAA9TKN6	UTP23	A0AAA9TKN6	ENSBTAG00000040422	0.090298654	0.073001831	0.017296823
A0A452DI94	PTGS1	A0A452DI94	ENSBTAG00000006716	0.038975967	0.013559264	0.025416704
A0AAA9TFU8	EDRF1	A0AAA9TFU8	ENSBTAG00000009439	-0.009618003	0.168681835	-0.178299837
Q32LJ6	RABL3	bta:505510; Q32LJ6	ENSBTAG00000015898	0.131180329	0.023435156	0.107745174
A0A3Q1N5M8				-0.122141941	-0.185126726	0.062984786
Q3SYY7	OCIAD2	bta:505877; Q3SYY7	ENSBTAG00000001839	-0.109492366	0.037989586	-0.147481952
F1N599	ELF2	F1N599	ENSBTAG00000020594	-0.039987849	0.013926958	-0.053914807
A0AAA9TCX8	PARD6B	A0AAA9TCX8	ENSBTAG00000021601	-0.048130758	-0.118798195	0.070667438
Q2TBK4	HAUS1	bta:528378; Q2TBK4	ENSBTAG00000002512	-0.019752847	0.051874439	-0.071627287
A0AAA9SKU5				0.278535499	0.233797185	0.044738315
A0A3Q1MBS8	TRMU	A0A3Q1MBS8; bta:616451	ENSBTAG00000005595	-0.481271198	-0.383931899	-0.097339299
E1B955	SLC30A5	bta:508169; E1B955	ENSBTAG00000011013	0.124781625	0.250999341	-0.126217716
F1MSI0	RANBP10	F1MSI0	ENSBTAG00000032763	-0.180937994	-0.129025906	-0.051912089
Q0VD50	NMNAT1	bta:522863; Q0VD50	ENSBTAG00000015897	-0.280927632	-0.193054876	-0.087872756
E1BH22	H2AC18	bta:614974; E1BH22; F2Z4I6	ENSBTAG00000032456; ENSBTAG00000038476	0.03493379	0.181819712	-0.146885922
A0A3Q1NHF7	MBNL2	A0A3Q1NHF7	ENSBTAG00000018313	0.065033885	0.030171528	0.034862357
G3X6Y2	CXHXorf38	G3X6Y2	ENSBTAG00000011788	0.090197809	-0.054839387	0.145037196
A2VE19	PLEKHF1	A2VE19; bta:782309	ENSBTAG00000008625	-0.188778785	-0.174315556	-0.014463229
A0AAA9S828	ABHD2	A0AAA9S828	ENSBTAG00000019954	-0.206216274	-0.319642335	0.113426061
A0A3Q1LKH0	PHF8	A0A3Q1LKH0	ENSBTAG00000013289	-0.349917191	-0.009053248	-0.340863943
A0AAA9TYI9				0.311229118	0.109669316	0.201559803
F6RDN5	CHMP4C	F6RDN5	ENSBTAG00000004792	0.24042746	0.137846519	0.102580941
A0AAA9SQ47	SFXN2	A0AAA9SQ47	ENSBTAG00000004321	-0.237328692	-0.21677488	-0.020553811
A0AAA9SFP0	POLE	A0AAA9SFP0; bta:785457	ENSBTAG00000000590	-0.417575222	-0.251418034	-0.166157188
C4T8B4	CRP	bta:527553; C4T8B4	ENSBTAG00000062195	-0.324360971	0.001214901	-0.325575872
F1N5S7	PDF	F1N5S7	ENSBTAG00000001663	0.098924233	-0.136128728	0.235052961
E1BHF2	KATNAL1	bta:537739; E1BHF2; W0GIS4	ENSBTAG00000009340	-0.160464672	-0.059968589	-0.100496083
F1N1T6	FASTKD2	F1N1T6	ENSBTAG00000016193	-0.261113922	-0.297766344	0.036652423
E1BPM4	CHD7	E1BPM4	ENSBTAG00000021841	-0.476438044	-0.189703803	-0.286734241
A0A3Q1MB94	LOC518495	A0A3Q1MB94; bta:518495	ENSBTAG00000048740	-0.382223761	0.706132113	-1.088355874
E1BCE1	ATAD5	E1BCE1	ENSBTAG00000018383	0.057371179	-0.160808991	0.21818017
A0AAA9T9T8	GATD1	A0AAA9T9T8	ENSBTAG00000010348	-0.366127899	-0.115872098	-0.250255801
F1N4K5	FOXO3	bta:535530; F1N4K5	ENSBTAG00000011234	0.054556164	0.153903974	-0.09934781
F1N1K9	TGS1	F1N1K9	ENSBTAG00000005898	0.004278881	-0.052361616	0.056640497
F1MRD5	PUS10	F1MRD5	ENSBTAG00000009694	0.040872281	0.061676897	-0.020804615
A6H713	LIPA	A6H713; bta:100125267; F1N110	ENSBTAG00000003636	0.064073365	0.211682778	-0.147609414
A0AAF6YJE9	MEN1	A0AAF6YJE9	ENSBTAG00000002095	0.015445058	0.197577373	-0.182132316
Q24JY6	TMEM160	bta:508953; Q24JY6	ENSBTAG00000015579	-0.152480728	-0.049551973	-0.102928755
O97725	NDUFA12	bta:281742; O97725; Q3T092	ENSBTAG00000067029	-0.018417634	-0.026161587	0.007743953
F1N3M4	PIK3AP1	F1N3M4	ENSBTAG00000019872	0.434866473	0.40599236	0.028874113
Q1RMX2	UBE2D2	bta:541003; Q1RMX2	ENSBTAG00000004161	0.010023728	-0.104264814	0.114288542
A0AAA9SMR5	RFFL	A0AAA9SMR5	ENSBTAG00000013645	0.04700926	0.145002389	-0.097993128
A0AAA9S8T7				0.026391499	0.019108823	0.007282676
F6QQ46	C10H15orf48	F6QQ46	ENSBTAG00000004558	0.11232419	0.025070218	0.087253972
Q28029	ATP6V1F	bta:282405; Q28029; Q2NKR2	ENSBTAG00000007572	0.974121368	0.347770629	0.626350739
Q0D253	NDNL2	bta:617494; Q0D253		-0.358882817	-0.19155961	-0.167323206
A0AAA9T6A1	SEC11C	A0AAA9T6A1	ENSBTAG00000011954	-0.092656225	-0.065367004	-0.027289222
A0AAA9TU86	TP53RK	A0AAA9TU86	ENSBTAG00000027326	0.137359549	0.083077888	0.054281661
Q5EA49	MIS12	bta:767858; Q5EA49	ENSBTAG00000020432	0.312939312	0.252542032	0.06039728
A1L4Z7	OLR1	A1L4Z7		-0.078002512	0.303868123	-0.381870635
Q3ZBN4	CUEDC2	bta:516091; Q3ZBN4	ENSBTAG00000002501	-0.070727711	-0.441442879	0.370715168
F6RM59	FIBP	F6RM59	ENSBTAG00000020775	0.052243868	0.237513285	-0.185269417
A0AAF6YZH3	ACYP1	A0AAF6YZH3	ENSBTAG00000013992	0.379848071	0.33093847	0.0489096
Q148K5	PEX11B	bta:506527; Q148K5		0.073854847	0.058543902	0.015310945
G5E526	PTP4A1	bta:613326; G5E526	ENSBTAG00000002275	0.462398682	1.058962469	-0.596563787
A0AAA9SF51	PLEKHG1	A0AAA9SF51	ENSBTAG00000009679	-0.04077453	0.007710842	-0.048485372
A0AAA9T663	ATG7	A0AAA9T663	ENSBTAG00000035827	-0.012939056	0.268272783	-0.281211838
Q1RMW1	UBE2F	bta:617083; Q1RMW1		0.125794798	0.084572135	0.041222663
A0AAA9TLV8	DCTN6	A0AAA9TLV8	ENSBTAG00000000744	0.071384633	-0.024095676	0.095480309
G3X686	SELENOO	G3X686	ENSBTAG00000000647	-0.335184192	-0.175348673	-0.159835518
A0AAA9SKL6	DTD2	A0AAA9SKL6; bta:514035	ENSBTAG00000068336	-0.139274789	0.113433257	-0.252708046
E1BE06	AFTPH	E1BE06	ENSBTAG00000009299	0.119156391	0.054336923	0.064819468
A0A3Q1MDF9	ACTR8	A0A3Q1MDF9	ENSBTAG00000011180	-0.352272704	-0.222392421	-0.129880283
A0AAA9S1J3	LTF	A0AAA9S1J3	ENSBTAG00000001292	0.058124046	0.122856748	-0.064732701
F6QT33	RBM34	F6QT33	ENSBTAG00000000221	-0.218031627	-0.243657435	0.025625808
A0AAF6ZVL1	HSDL1	A0AAF6ZVL1	ENSBTAG00000018770	-0.242655325	-0.111659233	-0.130996093
A0A3Q1M883	AGFG2	A0A3Q1M883	ENSBTAG00000000713	0.066891884	0.043356369	0.023535514
Q0P5L7	GLMP	bta:100125876; Q0P5L7	ENSBTAG00000006364	-0.044738315	0.278163149	-0.322901464
A0AAA9S448	B2M	A0AAA9S448	ENSBTAG00000048782	-0.066135102	-0.046277683	-0.019857418
Q3MHR3	DYNLL2	bta:540369; Q3MHR3	ENSBTAG00000025313	-0.053025709	-0.278738489	0.22571278
Q3SZ34	UBL4A	bta:504533; Q3SZ34		0.076519021	0.096071818	-0.019552797
A7E3R8	TMEM109	A7E3R8		-0.093015574	-0.276894341	0.183878768
Q3ZCI1	TMEM14C	bta:613802; Q3ZCI1		0.145850866	0.240346811	-0.094495945
G3MZM8	VKORC1L1	G3MZM8	ENSBTAG00000014448	0.482857052	0.417078375	0.065778676
Q0VCU0	PCBP4	bta:506889; Q0VCU0		-0.200819009	0.15627594	-0.357094949
F1N430	TIMP2	F1N430	ENSBTAG00000010899	-0.227274643	-0.108415667	-0.118858976
Q0VD44	TWSG1	bta:537290; F1N2H5; Q0VD44	ENSBTAG00000001805	0.143073782	0.028870058	0.114203723
A0A452DI42	DCAKD	A0A452DI42	ENSBTAG00000006048	0.288676289	0.218473827	0.070202462
A0A3Q1N2S6	PEF1	A0A3Q1N2S6	ENSBTAG00000010378	0.225667553	0.20874425	0.016923303
F1MN80	WTAP	F1MN80	ENSBTAG00000007974	0.085025381	0.161051219	-0.076025838
F6RE68	UBL7	F6RE68	ENSBTAG00000000010	0.330332795	0.224706287	0.105626508
Q9XSN4	GAPDL17	Q9XSN4		-0.102051271	-0.140321288	0.038270017
Q3T0K8	EMC4	bta:523162; Q3T0K8	ENSBTAG00000006416	0.103958861	0.040765034	0.063193826
A0A3Q1LRB4	FAM118A	A0A3Q1LRB4	ENSBTAG00000010590	0.036776571	0.32793278	-0.291156209
A0AAA9SFN2	IFT122	A0AAA9SFN2	ENSBTAG00000019121	-0.488419971	-0.434027675	-0.054392297
Q2HJG7	CHURC1	bta:616539; Q2HJG7		0.675016426	0.10589743	0.569118996
A0AAA9SDR2	DIP2C	A0AAA9SDR2	ENSBTAG00000006531	0.055158696	-0.044565552	0.099724249
A0A3Q1LLS6	USP40	A0A3Q1LLS6; bta:531139	ENSBTAG00000000149	0.04463943	0.033608393	0.011031037

Accession	Gene_Symbol	Gene_ID	Ensembl_Gene_ID	log2(ratio(OV24H/OV0H))	log2(ratio(OV24R/OV0H))	log2(ratio(OV24H/OV24R))
F1MWN2	HCK	F1MWN2	ENSBTAG00000007932	-0.010286639	0.436656075	-0.446942715
A0A3Q1NH47	SP1	A0A3Q1NH47	ENSBTAG00000003021	-0.213549568	-0.283605363	0.070055795
G3N260	EARS2	bta:100300732; G3N260	ENSBTAG00000045593	0.15733091	0.176349764	-0.019018854
A0A385KL35				-0.414114765	-0.571868928	0.157754163
A0A3Q1LSS9	EYA3	A0A3Q1LSS9	ENSBTAG00000043989	-0.035046947	0.059355278	-0.094402225
F1N173	PLXNA3	F1N173	ENSBTAG00000008552	-0.135303734	0.044794331	-0.180098065
A5PK72				-0.100417786	0.44298404	-0.543401826
A0AAA9RUL0	STEEP1	A0AAA9RUL0	ENSBTAG00000047169	0.155175872	-0.098580721	0.253756592
A1A4Q9	NUDT16	A1A4Q9		-0.907459594	-0.564177935	-0.343281659
A0AAA9T584	CSNK1G3	A0AAA9T584	ENSBTAG00000017550	0.051505438	-0.113470933	0.164976371
A0A3Q1NL63	PIMREG	A0A3Q1NL63	ENSBTAG00000002981	0.321612026	0.106576556	0.21503547
A0A3Q1M6X0	GYG1	A0A3Q1M6X0	ENSBTAG00000001721	0.102044206	0.360798617	-0.258754411
A0A3Q1LPH5				-0.036305264	-0.170283569	0.133978305
Q0P5E8	CHRAC1	bta:510942; Q0P5E8	ENSBTAG00000020226	0.283969008	0.15708792	0.126881088
A0AAA9S2T2	PPM1H	A0AAA9S2T2	ENSBTAG00000011857	0.033085366	-0.124770454	0.15785582
A0AAA9S0G1	CCNH	A0AAA9S0G1	ENSBTAG00000009566	0.100292795	-0.001623742	0.101916538
A0JN42	TTI1	A0JN42; F6Q3H3	ENSBTAG00000004100	-0.040601133	0.002856823	-0.043457956
A0AAA9U0X9	TPRKB	A0AAA9U0X9	ENSBTAG00000011176	0.121351174	-0.1023208	0.223671974
A0AAF6YM64	H2AZ1	A0AAF6YM64	ENSBTAG00000004428	-0.514021719	-0.169120868	-0.344900851
Q1JQD9	L3MBTL2	bta:513297; Q0V8P8; Q1JQD9		-0.138827705	-0.169759564	0.03093186
A0AAA9TNQ6				-0.14822993	-0.105794664	-0.04243266
A0AAA9T8K2	SH3BP5L	A0AAA9T8K2	ENSBTAG00000002645	0.018361821	-0.052418534	0.070780355
A0A3Q1NGV7	FGD1	A0A3Q1NGV7	ENSBTAG00000015860	0.185529191	0.150006496	0.035522695
F6QF30	DUS2	F6QF30	ENSBTAG00000014644	-0.041960532	-0.071741991	0.02978146
A0A3Q1LGY5	WRAP53	A0A3Q1LGY5	ENSBTAG00000001071	-0.008578985	0.111084111	-0.119663096
A0A452DIN8	MRPL20	A0A452DIN8	ENSBTAG00000004872	-0.152003093	-0.180219336	0.028216242
F1N5P6	SLC7A1	bta:539465; F1N5P6	ENSBTAG00000018577	0.178521374	0.338617758	-0.160096384
A0AAA9TJW3	MIER1	A0AAA9TJW3	ENSBTAG00000005443	-0.03093186	-0.01759406	-0.0133378
F1MXZ2	SLC16A3	F1MXZ2	ENSBTAG00000017461	0.185214872	0.302028537	-0.116813665
A0AAF6YN19	HIRIP3	A0AAF6YN19	ENSBTAG00000005028	-0.138646382	0.170650156	-0.309296538
A0A3Q1M7P7	CADM3	A0A3Q1M7P7	ENSBTAG00000003217	0.137600028	0.27641888	-0.138818851
F1MHZ3	NPAT	bta:526583; F1MHZ3	ENSBTAG00000010286	-0.071790683	-0.130377709	0.058587026
A0AAF7AEI7	SNUPN	A0AAF7AEI7	ENSBTAG00000012293	-0.14338608	0.081320207	-0.224706287
Q2KHV2	PACC1	bta:616392; Q2KHV2	ENSBTAG00000000758	0.116944116	0.388748732	-0.271804615
A4FUY6	TFCP2	A4FUY6; bta:509448; F6R2A8	ENSBTAG00000019312	0.159102032	0.15329641	0.005805622
A5PKH8	PGM2L1	A5PKH8; bta:515366	ENSBTAG00000000770	0.002500339	-0.065268289	0.067768628
A6QR35	SCAMP2	A6QR35; bta:534312	ENSBTAG00000005844	-0.147723366	-0.03909982	-0.108623546
A0AAA9S2Q2	VTI1A	A0AAA9S2Q2	ENSBTAG00000050055	-0.089146732	0.158225273	-0.247372005
E1B9G8	ARHGEF18	bta:522521; E1B9G8	ENSBTAG00000040507	0	0.026704324	-0.026704324
A3FPG8	GPAT4	A3FPG8; bta:511614		-0.063088536	-0.111807543	0.048719007
Q29RR7	VPS37A	bta:513985; Q29RR7	ENSBTAG00000010355	-0.005217709	0.384774661	-0.38999237
A5PKE0	C16H1ORF55	A5PKE0; bta:534664		0.147856224	-0.170339152	0.318195376
A6QNJ7	PGM5	A6QNJ7; bta:785045; F1MTN7	ENSBTAG00000033190	-0.00965017	0.030924573	-0.040574743
Q3SZK3	GHITM	bta:404143; Q3SZK3	ENSBTAG00000032829	-0.392577298	0.311636001	-0.704213299
A5PJA8	EMC7	A5PJA8; bta:615423		0.017996429	0.02987053	-0.0118741
A0A3Q1M210	TPPP	A0A3Q1M210	ENSBTAG00000052247	-0.0583557	0.0301238	-0.0884795
F1N463	ARID2	F1N463	ENSBTAG00000011087	-0.140177658	0.060024771	-0.200202429
A1L522	SMYD5	A1L522; bta:509313; F1MGV1		0.048318024	-0.071345072	0.119663096
A0AAA9SM52	DMAC2	A0AAA9SM52	ENSBTAG00000009449	-0.108479101	0.02446524	-0.132944341
A0AAA9SWU6	PGGT1B	A0AAA9SWU6	ENSBTAG00000001895	0.04434538	0.11138868	-0.0670433
A0A3Q1LX15	BCLAF3	A0A3Q1LX15; bta:511003	ENSBTAG00000021962	-0.242701404	0.151338104	-0.394039508
A0A3Q1MXK6	HINT2	A0A3Q1MXK6	ENSBTAG00000011444	-0.280648356	-0.580107661	0.299459306
A0AAF6DME7	FTSJ1	A0AAF6DME7	ENSBTAG00000031829	-0.183049921	-0.079149055	-0.103900866
A2VDT8	HPS6	A2VDT8; bta:511792	ENSBTAG00000021942	-0.167859582	0.009613425	-0.177473007
A0A3Q1M2V9	TMEM11	A0A3Q1M2V9	ENSBTAG00000010566	-0.078465434	-0.106594807	0.028129373
A0AAA9TDE1	GPN1	A0AAA9TDE1	ENSBTAG00000003904	0.230765602	0.111466674	0.119298928
A0AAA9S464	KCTD5	A0AAA9S464	ENSBTAG00000040575	0.146254459	0.041380324	0.104874135
A0A3Q1LT12	GSTA4	A0A3Q1LT12	ENSBTAG00000004288	-0.219999495	-0.052680317	-0.167319178
A0AAF6YUT0	THG1L	A0AAF6YUT0	ENSBTAG00000010359	0.0568245	0.237751042	-0.180926542
A0AAA9TU88	TGFBRAP1	A0AAA9TU88	ENSBTAG00000021731	0.065986356	0.116430855	-0.050444499
A0AAA9SIJ7	TIMP1	A0AAA9SIJ7	ENSBTAG00000066152	-0.296171968	-0.373637383	0.077465415
F6REB9	NAA40	F6REB9	ENSBTAG00000003073	-0.610261908	-0.414597586	-0.195664322
Q32KR9	CHMP1A	bta:789414; Q32KR9	ENSBTAG00000033331	0.007402248	-0.002971567	0.010373815
A0A3Q1MLK8	OGFOD3	A0A3Q1MLK8; bta:506185	ENSBTAG00000018172	0.258027331	0.332621763	-0.074594432
A0A3Q1MN55	TXNL4A	A0A3Q1MN55	ENSBTAG00000013587	0.347222115	0.255654875	0.09156724
A0AAA9SW13	ENY2	A0AAA9SW13	ENSBTAG00000007387	-0.069987519	-0.20861639	0.138628871
F2Z4F6	SNX7	F2Z4F6	ENSBTAG00000031461	0.172734535	0.045160899	0.127573636
E1BGE6	FBXW8	bta:787118; E1BGE6	ENSBTAG00000036183	-0.157797952	-0.109484015	-0.048313938
A0A452DKI2	PYROXD1	A0A452DKI2	ENSBTAG00000021078	-0.078791603	0.048862267	-0.12765387
A0A3Q1M072	GORASP1	A0A3Q1M072	ENSBTAG00000047135	-0.191152377	-0.126957175	-0.064195202
Q5E9Q3	CCDC51	A6QQF9; bta:517515; Q5E9Q3; Q5E9W7; Q5EA69	ENSBTAG00000013413	0.168013341	0.199661712	-0.031648371
F1MXD0	SPOUT1	F1MXD0	ENSBTAG00000012485	0.080465711	-0.001562204	0.082027914
Q3MHJ0				0.013820495	-0.003089284	0.016909779
Q2KHV9				0.090336737	0.007621228	0.082715509
F6Q3F4	ZNF277	F6Q3F4	ENSBTAG00000013050	-0.132770646	-0.248247863	0.115477217
A0A3Q1N043	TMA16	A0A3Q1N043	ENSBTAG00000012658	-0.282471211	-0.160698049	-0.121773163
F6R6Y8	PARP9	F6R6Y8	ENSBTAG00000021791	-0.220011557	-0.139064038	-0.08094752
P82928	MRPS28	bta:535290; P82928; Q2KI96	ENSBTAG00000001302	-0.193434947	-0.187128073	-0.006306874
A0A3Q1N8M5	INO80	A0A3Q1N8M5	ENSBTAG00000010380	-0.038898546	0.08304354	-0.121942087
F1MRA9	PAK1IP1	F1MRA9	ENSBTAG00000018674	-0.038123545	-0.030152834	-0.007970711
Q2LGB7	TICAM2	bta:539350; Q2LGB7	ENSBTAG00000002357	0.121824945	-0.018496344	0.140321288
E1BDA1	BRMS1	E1BDA1	ENSBTAG00000017689	-0.188203735	-0.156045067	-0.032158668
A4FUC9	RHPN2	A4FUC9; bta:533687	ENSBTAG00000003089	0.034703469	0.411455589	-0.37675212
A0AAF6YTM4	C4H7orf25	A0AAF6YTM4	ENSBTAG00000009472	-0.154192075	0.15453192	-0.308723995
Q0P5G9	SPRYD4	bta:539560; Q0P5G9	ENSBTAG00000009281	-0.086804828	-0.112190146	0.025385318
A0AAA9SZU0	LOC531462	A0AAA9SZU0	ENSBTAG00000010813	-0.251829897	-0.428138112	0.176308215
A0A0A0MP99	CA1	A0A0A0MP99	ENSBTAG00000036116	0.150824902	0.488787796	-0.337962893
B2Z4B0	ANGPTL2	B2Z4B0; bta:512019	ENSBTAG00000011810	-0.228582779	-0.133665108	-0.094917671
F1MNN7	LBP	F1MNN7	ENSBTAG00000016864	-0.022542569	-0.062025855	0.039483286
A6QLJ8	PTER	A6QLJ8; bta:782020	ENSBTAG00000016676	0.095313675	0.468095679	-0.372782004
A0AAA9TXB2	IQSEC1	A0AAA9TXB2	ENSBTAG00000003237	-0.188856817	-0.289113298	0.10025648
F1N1Q2	SLC6A17	bta:407174; F1N1Q2	ENSBTAG00000001981	0.095252459	0.287199065	-0.191946605
A0A3Q1MH47	FIS1	A0A3Q1MH47	ENSBTAG00000007900	0.15065415	0.124781625	0.025872525
Q2TBR6	PFDN4	bta:514621; Q2TBR6	ENSBTAG00000008812	0.22217103	0.156953371	0.065217659
A0A3Q1MBE1	LOC404103	A0A3Q1MBE1	ENSBTAG00000049782	-0.214124805	-0.05766935	-0.156455455
F1MU27	MARCHF11	F1MU27	ENSBTAG00000037576	-0.251901663	-0.259482842	0.007581179
A0AAA9SGI1	DVL3	A0AAA9SGI1	ENSBTAG00000019336	0.047889012	0.129004746	-0.081115734
A0AAA9S7E2	DHX33	A0AAA9S7E2	ENSBTAG00000032083	0.368437086	0.314146931	0.054290156
Q3T0N3	TMCO1	bta:614715; Q3T0N3	ENSBTAG00000010009	-0.098756681	-0.040096645	-0.058660035
A0A3Q1MEE5	DIP2A	A0A3Q1MEE5	ENSBTAG00000011495	-0.06788631	-0.023773062	-0.044113248
F1MB60	RPS26	F1MB60	ENSBTAG00000038896	-0.114536432	-0.059070155	-0.055466277
Q0IIB2	NFKBIB	bta:525408; F1N2I4; Q0IIB2		-0.070389328	-0.075742562	0.005353234
A0AAA9SB18	SIL1	A0AAA9SB18	ENSBTAG00000033747	0.113264834	0.153869917	-0.040605083
A0AAA9TR33	PDGFC	A0AAA9TR33; bta:613787	ENSBTAG00000043959	-0.233512036	-0.218345588	-0.015166448
A0AAA9TJG9	PECR	A0AAA9TJG9	ENSBTAG00000006821	0.225831505	0.230241173	-0.004409667
A0AAA9SST1	PRKCQ	A0AAA9SST1	ENSBTAG00000010664	-0.296019924	-0.370822417	0.074802492
A0AAA9TJ16	ALKBH8	A0AAA9TJ16	ENSBTAG00000010642	-0.156458151	-0.135133106	-0.021325045
A6QPR9	TEFM	A6QPR9		-0.076277473	-0.067757596	-0.008519877
A0A3Q1NNB2	NRDE2	A0A3Q1NNB2	ENSBTAG00000005427	0.013434402	0.068817375	-0.055382974
A0A3Q1N5T1	NEDD9	A0A3Q1N5T1	ENSBTAG00000006287	-0.154634544	0.072183849	-0.226827393
A0AAF6YTK9	USP22	A0AAF6YTK9	ENSBTAG00000008978	-0.185179863	-0.093705859	-0.091474005
A0A3Q1MHV8	SEC14L1	A0A3Q1MHV8	ENSBTAG00000013677	-0.1015806	0.037649351	-0.139229951
A0A3Q1M0E5	PARP16	A0A3Q1M0E5	ENSBTAG00000012352	0.042770772	0.088398855	-0.045628083
G3N348	WFS1	bta:100298456; G3N348	ENSBTAG00000046671	0.155597526	0.301124179	-0.145526653
E1BPQ4	HMGA2	bta:100297155; E1BPQ4	ENSBTAG00000044118	0.251538767	0.341639422	-0.090100655
A7MB67	ZYG11B	A7MB67; bta:540744	ENSBTAG00000019680	0.109794142	-0.011172912	0.120967054
A5D9D8	CYP3A4	A5D9D8; bta:507988		0.100744974	0.655783838	-0.555038864
A0AAA9S7W7	RGP1	A0AAA9S7W7	ENSBTAG00000011433	-0.204358499	-0.06445598	-0.139902518
A0AAA9S5L7	DSCC1	A0AAA9S5L7	ENSBTAG00000015338	-0.193808073	-0.191151175	-0.002656898
E1BPN1	WWTR1	E1BPN1	ENSBTAG00000007814	0.202008775	-0.06811189	0.270120665
A0A3Q1M5C7				-0.029270845	-0.154377342	0.125106498
Q0VCQ2	SLC27A4	bta:514427; Q0VCQ2	ENSBTAG00000015436	-0.007356951	0.119648325	-0.127005277
A5PJN1	CCDC59	A5PJN1; bta:615189; Q32KL6	ENSBTAG00000036282	0.307998294	0.139699964	0.16829833
A0A3Q1NK96	TXNDC15	A0A3Q1NK96	ENSBTAG00000014744	0.383430306	0.213651437	0.169778869
A0AAA9SQX1				0.063927327	0.060946551	0.002980776
A5PKD2	GLTP	A5PKD2		0.172145265	0.136204384	0.035940881
F1MTZ3	UBASH3B	bta:523809; F1MTZ3	ENSBTAG00000000842	-0.129345375	0.146004353	-0.275349728
A5PK03	FRMD8	A5PK03; bta:515469		-0.282399731	-0.183514066	-0.098885665
A0AAA9SGT2	ELP6	A0AAA9SGT2	ENSBTAG00000011478	0.101695397	0.047828525	0.053866872
F1ME21	ZBTB38	bta:539843; F1ME21	ENSBTAG00000040061	-0.575394616	-0.112622995	-0.462771622
A0A3Q1LM07	KIF7	A0A3Q1LM07	ENSBTAG00000002440	-0.104641242	-0.037016809	-0.067624433
A0AAA9TNK4	SLC39A6	A0AAA9TNK4	ENSBTAG00000000998	0.280565583	0.117989061	0.162576522
A0AAA9SP61	NXT2	A0AAA9SP61	ENSBTAG00000020739	0.011861897	-0.004473477	0.016335373
F1MV65	LOC100848991	bta:100848991; F1MV65	ENSBTAG00000030470	0.157739463	-0.121356716	0.279096179
E1BDI3	POLG	E1BDI3	ENSBTAG00000009098	-0.394392706	-0.25008587	-0.144306836
A0A3Q1LL04	YIPF1	A0A3Q1LL04; bta:511748	ENSBTAG00000050962	-0.085590869	-0.05838291	-0.027207959
A0AAF6DLN3	HID1	A0AAF6DLN3	ENSBTAG00000010809	-0.019960742	0.272182788	-0.29214353
A6QLV5	ZFAND3	A6QLV5; bta:532641; F1MWI6	ENSBTAG00000000527	-0.037868186	-0.045562577	0.007694392
Q6B857	CFAP20	bta:445424; Q6B857	ENSBTAG00000013874	0.171157547	0.076979997	0.09417755
E1BKM9	ZMYM3	E1BKM9	ENSBTAG00000008492	-0.272494735	0.095758983	-0.368253718
A6QR43	PJA2	A0A3Q1M5S9; A6QR43; bta:511508	ENSBTAG00000021675	-0.331409148	-0.373229324	0.041820176
A0AAA9S3N3	CCM2	A0AAA9S3N3	ENSBTAG00000008090	0.079887772	0.091147888	-0.011260116
A0A3Q1LRP0	GXYLT1	A0A3Q1LRP0; bta:617596	ENSBTAG00000015024	-0.15782042	-0.102233658	-0.055586762
A0AAA9SDW3	COA1	A0AAA9SDW3	ENSBTAG00000012968	-0.006550273	-0.033052878	0.026502606
E1BAK9	SYVN1	E1BAK9	ENSBTAG00000005076	-0.059884891	0.316209411	-0.376094302
F1N6V1	ARHGAP6	F1N6V1	ENSBTAG00000002626	-0.145628451	-0.141298198	-0.004330253
G9MD91	LGALS9	bta:510813; G9MD91		-0.089455667	-0.164844695	0.075389028
F1MDC7	MSANTD2	F1MDC7	ENSBTAG00000011248	-0.449491556	-0.442968432	-0.006523124
Q3MHY8	RBM7	bta:515307; Q3MHY8	ENSBTAG00000008219	-0.265041534	-0.051035464	-0.21400607
A5PJP6	BRCC3	A5PJP6; bta:519513; Q58D60	ENSBTAG00000007817	-0.0203815	0.011518584	-0.031900084
Q2KJA0	ARAF	F1MQC5; Q2KJA0		-0.070389328	0.069902013	-0.140291341
A0AAA9U2H6	IGHMBP2	A0AAA9U2H6	ENSBTAG00000022185	-0.156342029	-0.036325932	-0.120016097
A3KMW9	ZNF644	A3KMW9; bta:539923	ENSBTAG00000011200	-0.202816883	0.066442143	-0.269259026
A0A3Q1M007	ZC3HAV1L	A0A3Q1M007	ENSBTAG00000014250	0.032305314	0.096046687	-0.063741373
A0AAA9T2R3	DEPDC1B	A0AAA9T2R3	ENSBTAG00000017026	-0.481495776	-0.575154883	0.093659107
Q1JQA5	NENF	bta:616334; Q1JQA5	ENSBTAG00000000759	0.271685858	-0.184271897	0.455957755
A0AAA9TXZ0	PAN2	A0AAA9TXZ0	ENSBTAG00000004376	0.055444327	0.073462162	-0.018017835
F1N193	ZBTB40	F1N193	ENSBTAG00000016448	-0.292480941	-0.411921045	0.119440105
Q3SX05	ECSIT	bta:507245; Q3SX05	ENSBTAG00000015049	-0.282595682	-0.182119683	-0.100476
Q3ZC10	SNRPD1	bta:508788; Q3ZC10	ENSBTAG00000008292	0.077777179	0.085867079	-0.0080899
Q3ZBW6	DECR2	bta:768256; Q3ZBW6	ENSBTAG00000026429	-0.30807319	-0.257887433	-0.050185757
Q2NL38	DCI	bta:535174; Q2NL38		0.025402848	0.179773927	-0.154371079
A0A3Q1M3P1	CARD11	A0A3Q1M3P1	ENSBTAG00000014698	0.032535728	-0.108894047	0.141429775
A0AAA9S6A6	RBPJ	A0AAA9S6A6	ENSBTAG00000003602	0.035333073	0.137774478	-0.102441405
A5PJS5	ORC5	A5PJS5; bta:519409; F1N4D2	ENSBTAG00000040058	-0.069962558	0.02398668	-0.093949239
Q0P5F0	ACP2	bta:535407; Q0P5F0		-0.135610637	0.057251176	-0.192861813
A0A3Q1LPN8	HERC5	A0A3Q1LPN8	ENSBTAG00000020538	-0.455721935	-0.215968353	-0.239753582
Q0IIG6	TARBP2	bta:514674; Q0IIG6		-0.009533262	0.06502516	-0.074558422
A0A3Q1N7N8	RRP7	A0A3Q1N7N8	ENSBTAG00000016510	-0.353343127	-0.052516397	-0.30082673
F1MYV8	MLF2	F1MYV8	ENSBTAG00000014931	-0.049592682	0	-0.049592682
A0AAA9SXH6	JAK2	A0AAA9SXH6; bta:525246	ENSBTAG00000012047	-0.162356456	-0.126830022	-0.035526434
E1BBI5	ZBTB11	E1BBI5	ENSBTAG00000013460	-0.006158793	-0.045258613	0.03909982
A0A3Q1MMY4	QSER1	A0A3Q1MMY4	ENSBTAG00000014399	-0.147467332	-0.13458604	-0.012881291
F1MNL4	NEO1	F1MNL4	ENSBTAG00000004990	-0.082833606	0.018818028	-0.101651634
P82931	MRPS6	bta:615431; P82931; Q3ZC18	ENSBTAG00000012594	-0.412392778	-0.458935364	0.046542586
A0AAA9SD03	C2CD2	A0AAA9SD03	ENSBTAG00000021259	0.052065723	-0.009671735	0.061737458
F1MDV2	SLC30A9	bta:522345; F1MDV2	ENSBTAG00000020367	0.131383608	0.263833904	-0.132450296
F1N378	ETNK1	F1N378	ENSBTAG00000005833	0.030508878	-0.203582572	0.23409145
O46728				-0.186715117	0.036744781	-0.223459898
E1BEN8	TAF5	bta:516001; E1BEN8	ENSBTAG00000009709	0.045949586	0.136512808	-0.090563222
Q3MHY0	TERF1	bta:519474; G3X7V3; Q3MHY0	ENSBTAG00000032982	-0.136791843	-0.104544299	-0.032247544
A6QP88	ZFAND1	A6QP88		-0.501848107	-0.251006309	-0.250841798
A0A3Q1NF54	PISD	A0A3Q1NF54	ENSBTAG00000067410	0.104994374	0.159198595	-0.054204221
A0AAA9SBR4	ERBB2	A0AAA9SBR4; bta:505709	ENSBTAG00000021798	-0.785029067	-0.342067566	-0.442961501
A3KN00	DUSP23	A3KN00; bta:513978	ENSBTAG00000004776	-0.221830734	-0.229714333	0.007883599
Q3SZV6	TIMM21	bta:614633; Q3SZV6		0.112110366	0.061805284	0.050305082
E1BL71				0.103195957	0.023578789	0.079617168
E1BLQ5	RPS6KB2	E1BLQ5	ENSBTAG00000009175	-0.314040185	0.032533964	-0.34657415
A0AAF6Z8G4	HSF1	A0AAF6Z8G4	ENSBTAG00000020751	-0.249008277	-0.280962962	0.031954685

Accession	Gene_Symbol	Gene_ID	Ensembl_Gene ID	log2(ratio(OV24H/OV0H))	log2(ratio(OV24R/OV0H))	log2(ratio(OV24H/OV24R))
A0A3Q1LPC6	YIPF3	A0A3Q1LPC6	ENSBTAG00000018967	0.100744974	0.04026387	0.060481104
A0AAA9SXT9	NDUFAF2	A0AAA9SXT9	ENSBTAG00000021607	0.078082186	-0.057128616	0.135210802
A0AAA9TVB1	TAX1BP3	A0AAA9TVB1	ENSBTAG00000000833	-0.13242857	-0.361440768	0.229012198
A0AAF6YUJ1	RRP15	A0AAF6YUJ1	ENSBTAG00000009838	0.144547289	-0.069398125	0.213945414
F1N551	GATA4	bta:327716; F1N551	ENSBTAG00000005425	-0.429620822	-0.00467144	-0.424949383
F1MYL8	MED13	F1MYL8	ENSBTAG00000011721	-0.140786108	0.008914725	-0.149700833
F6RLZ6	TRIM32	F6RLZ6	ENSBTAG00000017155	0.143107346	0.06502516	0.078082186
A0A3Q1MU04	APBB1	A0A3Q1MU04	ENSBTAG00000015655	-0.139628781	0.065053387	-0.204682167
A5PJX0	FBXO22	A5PJX0; bta:536605	ENSBTAG00000002833	0.055659827	0.176457971	-0.120798144
F1MJI9	PCIF1	F1MJI9	ENSBTAG00000010126	0.058531612	0.222650992	-0.16411938
F6QJC0	POMGNT1	F6QJC0	ENSBTAG00000013322	-0.094137931	0.237336568	-0.331474499
F1MER3	CWC25	F1MER3	ENSBTAG00000030591	0.308736426	0.189313987	0.119422439
A0AAF6DMB2	AAGAB	A0AAF6DMB2	ENSBTAG00000040368	0.372931644	0.161858087	0.211073557
Q3SZG8	ISCA1	bta:514652; Q3SZG8	ENSBTAG00000053156	-0.230297619	-0.449610648	0.219313028
A0AAF6ZH32	BABAM1	A0AAF6ZH32	ENSBTAG00000012587	0.04143409	0.081711343	-0.040277253
A0AAA9SAD6	PDE4D	A0AAA9SAD6	ENSBTAG00000000494	0.036378504	0.299652382	-0.263273877
E1BFV2	MAP3K21	bta:616014; E1BFV2	ENSBTAG00000021183	0.014466018	-0.089981367	0.104447385
A0AAA9T7R0	ADISSP	A0AAA9T7R0	ENSBTAG00000018735	-0.159545856	-0.125057479	-0.034488376
Q2YDI8	MKRN2	bta:508004; Q2YDI8	ENSBTAG00000004512	0.184311414	-0.035800721	0.220112136
A0AAA9SBZ8	UBR2	A0AAA9SBZ8	ENSBTAG00000005967	-0.297201009	-0.182449311	-0.114751698
E1B8D0	GEN1	bta:785690; E1B8D0	ENSBTAG00000020059	0.364347892	0.003553438	0.360794454
A0A452DIR9	BUD23	A0A452DIR9	ENSBTAG00000017067	0.023176322	-0.131383608	0.154559931
Q2KHW8	GEMIN6	bta:525263; Q2KHW8		-0.143158725	-0.384166824	0.2410081
Q3T0H4	ZCCHC17	bta:538816; Q3T0H4	ENSBTAG00000016818	-0.009004813	0.001281826	-0.010286639
Q3T103	CDIPT	bta:515135; Q3T103	ENSBTAG00000007954	-0.052798518	0.213939856	-0.266738375
E1BK34	DVL2	E1BK34	ENSBTAG00000003075	0.201980371	0.210271745	-0.008291374
A0AAA9TK64	GNB1L	A0AAA9TK64	ENSBTAG00000021052	-0.140291341	0.039829427	-0.180120769
A0AAA9TME5	CDKN2A	A0AAA9TME5	ENSBTAG00000034220	0.765643963	-0.018688021	0.784331984
A0AAA9SJS7	KIAA0319L	A0AAA9SJS7	ENSBTAG00000016694	-0.455422571	-0.443144241	-0.01227833
A6QP29	TBC1D2	A6QP29; bta:513828		-0.590561254	-0.367076461	-0.223484793
E1BP59	KAT2A	E1BP59	ENSBTAG00000044019	-0.077346369	0.127129709	-0.204476078
M5FMW2	MAPK8IP3	A0A3Q1MTK3; A0A8J8XE10; M5FMW2	ENSBTAG00000022211	-0.215587243	1.010195767	-1.225783011
A0A3Q1MW97	ANO1	A0A3Q1MW97	ENSBTAG00000009997	0.395317073	0.404008034	-0.00869096
A0AAA9SME8	ANKIB1	A0AAA9SME8; bta:505204	ENSBTAG00000034936	-0.056143078	-0.024994379	-0.031148699
A0AAA9SVS9	CASP7	A0AAA9SVS9; bta:526279	ENSBTAG00000006615	0.257332046	0.435170919	-0.177838872
F6QWY1	ELP5	F6QWY1	ENSBTAG00000019446	0.173599322	0.05415577	0.119443552
A0AAF6Z9K7	IFNAR1	A0AAF6Z9K7	ENSBTAG00000021819	-0.211504105	-0.035750025	-0.17575408
A0AAA9TCS3	ZNF687	A0AAA9TCS3	ENSBTAG00000007318	-0.0538227	0.115789794	-0.169612493
F1MPD0	NRG1	F1MPD0	ENSBTAG00000004150	-0.191790148	-0.164696487	-0.027093661
E1BBV0	ZNF703	E1BBV0	ENSBTAG00000013943	-0.263034406	0.070175294	-0.3332097
A0AAA9SWL7	C23H6orf47	A0AAA9SWL7	ENSBTAG00000023628	0.590807323	0.498475442	0.092331881
Q3SX23	LONP2	bta:541085; Q3SX23	ENSBTAG00000010867	-0.093021088	0.028854863	-0.121875951
E1BAQ2	YTHDF1	E1BAQ2	ENSBTAG00000010383	0.260740165	0.224574026	0.036166138
A0AAA9S999	MPHOSPH6	A0AAA9S999	ENSBTAG00000009746	-0.039846815	0.045138275	-0.08498509
A0AAA9T4I5	MAP3K3	A0AAA9T4I5	ENSBTAG00000008151	0.255119915	0.180970148	0.074149767
A0AAF6YS40	RILPL2	A0AAF6YS40	ENSBTAG00000007804	0.261593871	-0.279367633	0.540961504
A0A3Q1LY17	TIMM9	A0A3Q1LY17	ENSBTAG00000010503	0.248631341	0.229200811	0.01943053
A0AAA9SIN1	PHKA1	A0AAA9SIN1	ENSBTAG00000015848	-0.338416218	-0.147153694	-0.191262524
A0AAA9TA72	WDR73	A0AAA9TA72	ENSBTAG00000001387	-0.293401847	-0.129647748	-0.163754099
A0AAA9U2N4	WDR4	A0AAA9U2N4	ENSBTAG00000021490	0.136122293	0.127807025	0.008315268
A0AAA9TN08	ARL6IP4	A0AAA9TN08; bta:526095	ENSBTAG00000001594	0.021738659	-0.030995152	0.052733811
A0A3Q1MCN9	TBC1D24	A0A3Q1MCN9	ENSBTAG00000005458	0.163911974	0.237740386	-0.073828412
A0A3Q1LGL5	GOLT1B	A0A3Q1LGL5	ENSBTAG00000000379	0.18223147	0.229725969	-0.0474945
A0AAA9TJA6	HMG20B	A0AAA9TJA6	ENSBTAG00000008789	-0.688701928	-0.313236165	-0.375465763
F6QPX6	BRI3BP	F6QPX6	ENSBTAG00000001543	0.244966113	0.132669417	0.112296696
F1N264	ZEB1	bta:535183; F1N264	ENSBTAG00000020053	-0.166172867	-0.231325546	0.06515268
A0A3Q1M1G7	ATF2	A0A3Q1M1G7	ENSBTAG00000022295	0.173960515	0.117806776	0.056153739
Q0II95	IP6K1	bta:534029; F1N7H3; Q0II95		0.07927277	-0.007565277	0.086838047
A0AAF6YSM1				0.495707358	0.12194405	0.373763308
A0AAA9TQT4				-0.010441747	0.15598581	-0.166427557
A5PJT2	INTS10	A5PJT2; bta:534094	ENSBTAG00000025003	-0.09105198	0.313373084	-0.404425065
E1B843	NAA20	bta:540212; E1B843	ENSBTAG00000001144	0.216771683	0.159835518	0.056936165
A2VDM1	LAMB3	A2VDM1; bta:529939		-0.369826536	-0.458654035	0.088827499
A0AAA9T7M7	CNOT6L	A0AAA9T7M7	ENSBTAG00000049571	-0.053719912	-0.069240497	0.015520585
A3KMY2	DKK2	A3KMY2; bta:541161	ENSBTAG00000012969	0.268332087	-0.460990072	0.729322159
F6QTX4	RIPK3	F6QTX4	ENSBTAG00000018422	-0.037816394	0.078941739	-0.116758133
Q32KQ2	WDR53	bta:518441; Q32KQ2	ENSBTAG00000014660	-0.064130337	-0.264565117	0.200434779
P20821	GCSH	bta:317723; P20821; Q0P5G3	ENSBTAG00000006795	0.222392421	0.125418308	0.096974113
A0AAF6YIZ9	MRPS12	A0AAF6YIZ9	ENSBTAG00000001782	-0.018941564	-0.217956166	0.199014603
G3MWS8	KLF16	G3MWS8	ENSBTAG00000038055	-0.49862413	-0.32103266	-0.17759147
A0AAA9T330	PPP1R3D	A0AAA9T330; bta:782160	ENSBTAG00000061854	0.208365536	0.094545637	0.1138199
G3MWQ7				-0.084450322	-0.148968282	0.064517961
A0A3Q1MJ38	CDKAL1	A0A3Q1MJ38	ENSBTAG00000025589	-0.504731888	-0.061497497	-0.443234391
Q3T0Y8	VAMP8	bta:507309; Q3T0Y8	ENSBTAG00000023997	-0.110471694	-0.044044333	-0.066427362
A0A3Q1N3E7	NEURL4	A0A3Q1N3E7	ENSBTAG00000010519	-0.031804185	-0.005730673	-0.026073512
A0AAA9TJI3	PITPNM1	A0AAA9TJI3	ENSBTAG00000010482	-0.42821997	-0.065130655	-0.363089315
A0A0M3T9B6	OPN	A0A0M3T9B6		-0.229303549	-0.237408598	0.00810505
A7Z054	TXNIP	A7Z054; bta:506790	ENSBTAG00000020060	0.08043027	0.512648296	-0.432218026
G3N060	FAM98C	G3N060	ENSBTAG00000039593	0.007527122	-0.021542619	0.02906974
F6RS92	FCGRT	F6RS92	ENSBTAG00000013926	0.069373703	0.063944929	0.005428774
Q3ZC24	MARCHF5	bta:540937; Q3ZC24	ENSBTAG00000020981	-0.122573617	0.039107391	-0.161681007
A6QPN6	IFI30	A6QPN6; bta:615930		-0.323050378	-0.129002582	-0.194047796
Q3ZC34	RBMS2	bta:509798; Q3ZC34	ENSBTAG00000017830	0.352066247	0.105905659	0.246160587
A2VDQ6	CDH11	A2VDQ6; bta:785475; E1BHE3	ENSBTAG00000032092	-0.128410683	0.043999193	-0.172409876
F1MG92	PDP1	F1MG92	ENSBTAG00000000199	-0.127419227	-0.050162795	-0.077256432
F1N6K5	HDHD5	F1N6K5	ENSBTAG00000016995	0.155820847	0.145339363	0.010481484
Q2KHU4	CLPP	bta:535981; Q2KHU4	ENSBTAG00000014712	0.194816177	0.185119678	0.009696499
A0AAA9T506	C18H19orf47	A0AAA9T506	ENSBTAG00000018859	0.066769012	0.041906952	0.02486206
Q5EA92	ARL14EP	bta:523894; Q2KIN9; Q5EA92	ENSBTAG00000012417	0.311669181	0.224040274	0.087628907
A6QLZ6	GLRX5	A6QLZ6; bta:505935	ENSBTAG00000013423	0.158953884	-0.431525622	0.590479506
F1N1J9	SOS2	F1N1J9	ENSBTAG00000005550	0.072756342	0.105765105	-0.033008762
E1BC48	CEP350	bta:534896; E1BC48	ENSBTAG00000008082	-0.058102955	0.044571105	-0.102674059
E1B9B6	NUAK1	bta:519892; E1B9B6	ENSBTAG00000010457	-0.229149788	-0.071063642	-0.158086145
A0A3Q1LRR1	MTMR3	A0A3Q1LRR1	ENSBTAG00000001030	0.039933627	0.214228228	-0.174294601
E1BPC2	PDZD8	E1BPC2	ENSBTAG00000011401	-0.262778858	-0.31587103	0.053092172
A0AAA9SU74	WASHC3	A0AAA9SU74	ENSBTAG00000000021	-0.078367871	-0.164182761	0.085814889
E1BD64	MPC2	bta:616718; E1BD64	ENSBTAG00000020968	-0.008095304	-0.123959147	0.115863843
A0AAA9T0G1	HUS1	A0AAA9T0G1	ENSBTAG00000013226	0.247321321	0.394743765	-0.147422444
F1MM71	IFIT1	F1MM71	ENSBTAG00000007881	-0.054576533	2.331270279	-2.385846811
E1BGL4	CPEB4	bta:538794; E1BGL4	ENSBTAG00000009995	0.35886135	0.55313017	-0.19426882
A0A140T876	RER1	A0A140T876	ENSBTAG00000009307	0.173732304	0.285192735	-0.11146043
A0A3Q1MIR4	CEP44	A0A3Q1MIR4	ENSBTAG00000020055	-0.095062197	0.026244099	-0.121306296
A0AAA9SEB2	CARS2	A0AAA9SEB2	ENSBTAG00000011471	-0.201633861	-0.125409561	-0.076224301
F1MY22	TMEM63B	bta:534891; F1MY22	ENSBTAG00000032680	-0.311717896	-0.033166864	-0.278551032
E1BJ55	ZNF428	bta:540173; E1BJ55	ENSBTAG00000012796	-0.081400725	-0.183352006	0.101951281
Q5E9Z1	RPUSD4	A7YWI2; bta:516934; Q0V8H4; Q1JP71; Q1JPL3; Q58D36; Q58DF5; Q5E9Z1; Q5E9Z6		-0.158342581	-0.430392889	0.272050308
A0A3Q1N449	TGFB1	A0A3Q1N449	ENSBTAG00000020457	0.109451142	0.761976927	-0.652525785
F1MAV4	BARD1	F1MAV4	ENSBTAG00000033690	-0.224838082	-0.11279177	-0.112046312
A0AAA9STM1	AP1S2	A0AAA9STM1	ENSBTAG00000020420	0.019790366	-0.029525952	0.049316318
G3MXK1	ITPRIPL2	bta:783291; G3MXK1	ENSBTAG00000057103	-0.039107391	0.082997923	-0.122105314
Q0VCT5	OSTM1	bta:518522; Q0VCT5	ENSBTAG00000010134	0.01035122	-0.009118645	0.019469864
E1BFR4	PLXNA1	E1BFR4	ENSBTAG00000009159	0.101849254	-0.159443518	0.261292772
F1MI39	CHKA	F1MI39	ENSBTAG00000010304	0.347510495	0.538640933	-0.191130437
Q2KHZ3	MTF2	bta:615211; F1MPR4; Q2KHZ3	ENSBTAG00000001393	0.082027914	0.06120515	0.020822764
Q17QJ0	ASF1B	bta:510538; Q17QJ0	ENSBTAG00000004085	-0.200010728	-0.176877762	-0.023132966
A6H757	ACP6	A6H757; bta:515738	ENSBTAG00000017051	-0.28819448	-0.496496153	0.208301673
Q3SZ46	NOL7	bta:510511; Q3SZ46	ENSBTAG00000021554	0.1260413	0.076066658	0.049974642
F1MBS7	MALT1	bta:504994; F1MBS7	ENSBTAG00000027916	0.053192939	-0.07364414	0.12683708
A0AAA9T9Y0	JMJD1C	A0AAA9T9Y0	ENSBTAG00000001573	-0.705502877	0.556652105	-1.262154982
F1MC78	CCNDBP1	F1MC78	ENSBTAG00000013032	0.273284503	0.129767875	0.143516628
Q3T0J3	MRPL16	bta:538054; Q3T0J3	ENSBTAG00000021188	-0.117381137	-0.018671277	-0.098709861
A0AAA9SJP0	CHML	A0AAA9SJP0	ENSBTAG00000061790	-0.049216991	-0.083886008	0.034669017
A0A3Q1LH29	SPIN1	A0A3Q1LH29	ENSBTAG00000003044	0.191681418	0.131043475	0.060637943
Q58CQ7	RASSF8	bta:515639; F1MC74; Q58CQ7	ENSBTAG00000021980	-0.035189428	-0.061458679	0.026269251
A0A3Q1MLB6	UBE2R2	A0A3Q1MLB6; bta:537885	ENSBTAG00000054773	0.19163613	0.24707792	-0.05544179
A0AAA9S3Q1	UQCRH	A0AAA9S3Q1	ENSBTAG00000009603	0.424497829	0.254014401	0.170483428
A4IFI9	JAM2	A4IFI9; bta:538846	ENSBTAG00000000603	0.147142382	0.001865152	0.14527723
A0AAA9SYM8	ISCA2	A0AAA9SYM8	ENSBTAG00000021956	0.574668306	0.167307958	0.407360347
A0AAA9SLK1	SYNJ2BP	A0AAA9SLK1	ENSBTAG00000007586	0.108090603	0.022275713	0.085814889
F1MEI8	GTF3C2	bta:782752; F1MEI8	ENSBTAG00000017283	0.004468858	0.08825247	-0.083783612
A0AAA9TJZ0	IGF2BP1	A0AAA9TJZ0	ENSBTAG00000011736	0.097482989	0.141538169	-0.04405518
Q0VC13	MAPDA	bta:512667; Q0VC13		-0.112209504	0.067114196	-0.179323699
F6RQE7	PIP4P1	F6RQE7	ENSBTAG00000002748	0.336355166	0.524507262	-0.188152097
A0JNA1	SGPP1	A0JNA1; bta:516881	ENSBTAG00000004430	-0.03604966	-0.03604966	0
A4IFE5	LRRC8D	A4IFE5; bta:539204	ENSBTAG00000020958	0.065176731	0.134301092	-0.069124361
A0A3S5ZP82	LAD1	A0A3S5ZP82	ENSBTAG00000024663	-0.08697782	0.087519911	-0.174497731
A0AAA9S6E2	C3H1orf50	A0AAA9S6E2	ENSBTAG00000017386	0.113267616	-0.127867453	0.241135069
A0A3Q1NCS8	NOSTRIN	A0A3Q1NCS8	ENSBTAG00000010362	0.108623546	0.231642058	-0.123018512
A0AAA9SUM5	SZRD1	A0AAA9SUM5	ENSBTAG00000009251	-0.107129875	-0.096648391	-0.010481484
A0AAA9T2L7	DDX11	A0AAA9T2L7	ENSBTAG00000007093	-0.181240315	-0.076413116	-0.104827199
F1N3U6	UVRAG	bta:614285; F1N3U6	ENSBTAG00000016355	-0.045584392	0.18360849	-0.229192882
F1MPJ6	PLEKHG5	bta:615910; F1MPJ6	ENSBTAG00000016728	0.019199647	0.005511736	0.013687911
A7MBG4	KEAP1	A7MBG4; bta:532791; F1MV29	ENSBTAG00000003543	-0.334809386	-0.325137651	-0.009671735
F1MF33	IFT56	bta:508011; F1MF33	ENSBTAG00000016373	-0.080867846	0.134542123	-0.215409969
F6Q6Z7	PPHLN1	F6Q6Z7	ENSBTAG00000008595	-0.094509788	-0.168426097	0.073916309
A0AAA9TX07	FADS3	A0AAA9TX07	ENSBTAG00000015511	-0.285892348	-0.171433623	-0.114458725
F1MPV3	RALBP1	bta:523324; F1MPV3	ENSBTAG00000021250	0.075422034	0.172556217	-0.097134183
A0A3Q1LLN9	RPL7L1	A0A3Q1LLN9	ENSBTAG00000018478	-0.433024807	-0.272746026	-0.160278782
A0AAA9SKU2	LYSMD1	A0AAA9SKU2	ENSBTAG00000020395	0.011055189	0.022026306	-0.010971118
A2VDS1	SCLY	A2VDS1; bta:790815		0.136241875	0.408084739	-0.271842863
A0AAA9T930	CLEC16A	A0AAA9T930	ENSBTAG00000005208	-0.038122209	0.00433459	-0.042456799
E1BGT6	PUS7L	E1BGT6	ENSBTAG00000004718	-0.200655621	-0.22169361	0.02103799
F6QGE6	ACOT13	F6QGE6	ENSBTAG00000016337	0.519716476	0.790431619	-0.270715143
A5PJV0	MAFG	A5PJV0; bta:515219	ENSBTAG00000000040	-0.152842602	0.053384324	-0.206226926
Q3T0Q2	TMEM59	bta:509775; F1MBR3; Q3T0Q2		-0.28190038	-0.125410892	-0.156489488
F6QED7	C18H19orf48	bta:516719; F6QED7	ENSBTAG00000011079	-0.240749691	-0.383637667	0.142887976
A0AAA9S4Y4				-0.069634188	0.225087537	-0.294721725
Q0VCI8	PPP1R14C	bta:617148; F1MRD7; Q0VCI8	ENSBTAG00000026586	-0.082525071	-0.29964255	0.217117479
A0AAA9T4I3	ARL6IP1	A0AAA9T4I3	ENSBTAG00000049516	-0.470297932	-0.530045395	0.059747463
A0AAF6ZNZ9				0.143722046	0.020112964	0.123609082
Q0P5I6	PRPF38A	bta:507240; Q0P5I6	ENSBTAG00000002720	0.072699489	0.093976148	-0.02127666
A0A3Q1M7A3	PLEKHA1	A0A3Q1M7A3	ENSBTAG00000004636	-0.410479217	-0.277257306	-0.133221912
Q29RH8	PDK2	Q29RH8		0.022530833	0.124647535	-0.102116702
E1BB20				-0.104072454	-0.125530882	0.021458428
A0AAA9TML5	PTBP2	A0AAA9TML5	ENSBTAG00000008809	0.03384777	0.229104062	-0.195256291
A6H7E1	MARS2	A6H7E1; bta:514726; Q0V8Q7	ENSBTAG00000002056	-0.525628361	-0.100322527	-0.425305835
A7YWI9	TDP2	A7YWI9; bta:507579	ENSBTAG00000000365	0.065792314	-0.068936655	0.134728969
Q148F9	C25H7ORF26	bta:517505; Q148F9		-0.456317239	-0.19358305	-0.262734188
Q32LJ4	TPRG1L	A5D9I0; bta:616333; Q32LJ4		-0.091227966	-0.187217786	0.09598982
A0A3Q1MLW5	FBXO21	A0A3Q1MLW5	ENSBTAG00000032097	-0.140893544	-0.161033867	0.020140323
A0AAA9TGJ1	TBC1D25	A0AAA9TGJ1	ENSBTAG00000009288	0.11318249	0.004464248	0.108718242
A0AAF6ZC43	SNRPC	A0AAF6ZC43	ENSBTAG00000024549	-0.07933896	-0.143590854	0.064251894
A0AAF6ZAY0	QPCTL	A0AAF6ZAY0	ENSBTAG00000012176	0	-0.035133537	0.035133537
A0A3Q1LTD9	ARHGAP10	A0A3Q1LTD9	ENSBTAG00000002531	0.025635927	-0.016378016	0.042013943
A0A3Q1M3R2	PDE8A	A0A3Q1M3R2	ENSBTAG00000015909	0.191808652	-0.087323484	0.279132136
Q0VCF3	SIKE1	bta:506848; Q0VCF3		0.315378996	0.33868194	-0.023302944
A0A3Q1MH84	S100A13	A0A3Q1MH84	ENSBTAG00000021378	0.337652447	0.565062943	-0.227410496
Q17QU5	MLST8	bta:535236; Q17QU5; Q58DN5		-0.474380456	-0.297873075	-0.176507381
Q05KI7	BAK1	bta:514090; Q05KI7		-0.052514006	-0.088170527	0.035656521
A0A3Q1MSM3	TASOR2	A0A3Q1MSM3	ENSBTAG00000019197	-0.107019607	-0.042698626	-0.064320981
Q2NKZ7	PTPMT1	bta:614890; Q2NKZ7	ENSBTAG00000018479	-0.310051032	-0.084845093	-0.22520594
Q3T0Z5	LENG1	bta:617891; Q3T0Z5		-0.043484155	-0.229836389	0.186352234
F1MX50	CREG1	F1MX50	ENSBTAG00000008931	0.409556426	0.293196376	0.11636005
A0AAA9SRY9	SLC25A46	A0AAA9SRY9	ENSBTAG00000009667	-0.112408305	-0.207112729	0.094704424
A0AAF7AC02	FSD1	A0AAF7AC02	ENSBTAG00000005999	0.206552975	0.296589899	-0.090036924
A0AAF6ZDK8	NUDT12	A0AAF6ZDK8	ENSBTAG00000027728	0.252041945	0.518203299	-0.266161354
A0AAA9TY08	FBXL18	A0AAA9TY08	ENSBTAG00000010264	-0.298738468	-0.221947214	-0.076791254
E1BC94	IBA57	bta:506009; E1BC94	ENSBTAG00000014918	0.167186919	0.040459607	0.126727312
A0AAA9SRA4	TRIM16	A0AAA9SRA4	ENSBTAG00000002605	0.022144641	-0.088579046	0.110723687

Accession	Gene_Symbol	Gene_ID	Ensembl_Gene ID	log2(ratio(OV24H/OV0H))	log2(ratio(OV24R/OV0H))	log2(ratio(OV24H/OV24R))
A0AAA9SX17	KDM5A	A0AAA9SX17; bta:507962	ENSBTAG00000020472	-0.068855362	0.055908741	-0.124764103
E1BCV8	FYTTD1	E1BCV8	ENSBTAG00000014874	0.197358232	-0.104941057	0.302299288
E1BPL5	KDM4A	bta:512622; E1BPL5	ENSBTAG00000002078	0.148005915	0.665663601	-0.517657687
Q1RMT3	MTHFS	bta:513372; Q1RMT3	ENSBTAG00000020023	-0.383462962	-0.085919026	-0.297543936
A0A3Q1LLL8	RAB20	A0A3Q1LLL8	ENSBTAG00000049416	-0.243251026	-0.106384313	-0.136866713
F1ML71	NDFIP2	F1ML71	ENSBTAG00000014227	-0.141524576	-0.076569134	-0.064955442
A0AAA9TLR9	COQ9	A0AAA9TLR9	ENSBTAG00000001855	0.08920495	-0.085125231	0.174330181
A0AAA9T1H8	STX8	A0AAA9T1H8	ENSBTAG00000017815	-0.084888898	0.091677449	-0.176566346
Q2T9W9	CAAP1	bta:515858; Q2T9W9	ENSBTAG00000001223	0.095502087	0.036378504	0.059123582
A0A3Q1M8E6	NHSL2	A0A3Q1M8E6	ENSBTAG00000045565	0.227805918	0.078910496	0.148895422
A0A3Q1LJR3	GPAA1	A0A3Q1LJR3	ENSBTAG00000014610	-0.058893689	-0.029146346	-0.029747343
A0A452DIV3	CCND1	A0A452DIV3	ENSBTAG00000017514	-0.352242373	-0.37920942	0.026967048
A7MB66	ABHD13	A7MB66; bta:513848	ENSBTAG00000067980	-0.128506616	-0.086237137	-0.04226948
A0AAF6YP33	FNTB	A0AAF6YP33	ENSBTAG00000005897	-0.066017173	-0.017000353	-0.04901682
Q2HJE8	CHCHD1	bta:540610; F1MUI6; Q2HJE8	ENSBTAG00000012654	-0.255570029	-0.226092827	-0.029477201
A0A3Q1N2T8	CDC34	A0A3Q1N2T8; bta:616156	ENSBTAG00000002098	-0.454674472	-0.430761852	-0.02391262
E1BNX4	CREB3L1	E1BNX4	ENSBTAG00000006143	-0.073622863	-0.084236537	0.010613674
A0AAA9SB42	DGUOK	A0AAA9SB42	ENSBTAG00000012037	0.014706499	0.160698049	-0.14599155
A0A3Q1MVP2	CERS5	A0A3Q1MVP2	ENSBTAG00000017395	0.046182101	0.021039676	0.025142426
Q2NL16	FBXO28	bta:537745; Q2NL16	ENSBTAG00000019538	-0.22336426	0.106707008	-0.330071268
F1N3Y6	ABCC3	F1N3Y6	ENSBTAG00000020070	-0.265063038	-0.064488726	-0.200574311
A0AAA9S0Z5	POLR3F	A0AAA9S0Z5	ENSBTAG00000008825	-0.127014373	0.105749732	-0.232764105
F1MQ15	IFFO2	F1MQ15	ENSBTAG00000030340	0.092940381	-0.045323991	0.138264372
A0AAA9S938	SCAMP4	A0AAA9S938	ENSBTAG00000045762	0.244605845	0.209209951	0.035395894
A6QLM8	HSCB	A6QLM8; bta:786040		-0.194122492	-0.094586819	-0.099535674
A0A3Q1LK65	THTPA	A0A3Q1LK65	ENSBTAG00000007097	0.404390255	0.27594189	0.128448365
A0AAA9TVQ8	CMC1	A0AAA9TVQ8	ENSBTAG00000035286	0.263608156	0.333587686	-0.06997953
A2VDU2	MAPKAP1	A2VDU2; bta:533861	ENSBTAG00000010271	-0.189572244	-0.11189288	-0.077679364
F1MGX8	SPDL1	F1MGX8	ENSBTAG00000008180	-0.454031631	-0.510457064	0.056425433
E1BEK4	RAD54L2	E1BEK4	ENSBTAG00000032253	-0.234965524	0.075912515	-0.310878038
A0AAA9TBA2	GSE1	A0AAA9TBA2	ENSBTAG00000009918	0.108524457	0.070389328	0.038135129
A0AAF6ZA81	POLR2K	A0AAF6ZA81	ENSBTAG00000022539	0.026105891	0.042749647	-0.016643756
A0A3Q1LFP3	UBE2G2	A0A3Q1LFP3	ENSBTAG00000048090	0.379383229	-0.037730389	0.417113619
Q08DD7	SNX11	bta:534567; Q08DD7		0.036885068	0.164165732	-0.127280664
A0A3Q1M1Z7	PIAS1	A0A3Q1M1Z7	ENSBTAG00000005564	-0.064064099	-0.139107409	0.07504331
A0A3Q1LL74	PLD1	A0A3Q1LL74	ENSBTAG00000017490	-0.286485265	-0.123956992	-0.162528273
A0AAA9S5G5	KIF21A	A0AAA9S5G5	ENSBTAG00000004832	-0.104301231	-0.121157126	0.016855896
E1BGA2	MITD1	E1BGA2	ENSBTAG00000003970	-0.053146593	0.021000299	-0.074146892
G3MZT8	LTBP4	G3MZT8	ENSBTAG00000004757	0.013016886	0.576706018	-0.563689132
A0AAA9TMD6	CLPTM1L	A0AAA9TMD6	ENSBTAG00000001329	-0.372857684	-0.359874964	-0.01298272
F1MSR5	TBCK	F1MSR5	ENSBTAG00000004227	-0.060360588	0.074070955	-0.134431543
Q3MHM8	ARL6IP6	bta:507137; Q3MHM8	ENSBTAG00000001435	-0.217109921	-0.296271905	0.079161984
F1MW45	DDX55	F1MW45	ENSBTAG00000007266	-0.257667896	-0.176098769	-0.081569127
E1BJH0	CNOT8	bta:533443; E1BJH0	ENSBTAG00000012086	-0.508745243	-0.404771521	-0.103973722
P82920	MRPS21	bta:614343; P82920; Q2HJ36	ENSBTAG00000024378	-0.478252794	0.012887684	-0.491140478
F1MVR0	CDK19	bta:511802; F1MVR0	ENSBTAG00000007288	-0.226879812	-0.162423832	-0.06445598
A0AAA9SYE7	SERGEF	A0AAA9SYE7	ENSBTAG00000005340	-0.331865596	-0.343168245	0.011302649
Q3SZT2	NQO2	bta:508566; Q3SZT2		-0.236067358	0.087547113	-0.323614472
Q08D83-2	RTN3	Q08D83-2		0.101640262	0.221317468	-0.119677206
A0AAA9TND5	CHMP6	A0AAA9TND5	ENSBTAG00000038745	0.031459926	0.223482482	-0.192022556
A0AAA9SNR3	TTC7A	A0AAA9SNR3	ENSBTAG00000032558	-0.095541987	-0.065255292	-0.030286695
A0AAA9RXV8				0.034651547	0.063922391	-0.029270845
A0A3Q1MKD4	BRD7	A0A3Q1MKD4	ENSBTAG00000021575	-0.086516727	-0.252063288	0.165546561
Q3SZ07	OVCA2	bta:513845; Q3SZ07	ENSBTAG00000047926	-0.469485283	-0.384822886	-0.084662397
Q1RMU0	NAB2	bta:504755; Q1RMU0	ENSBTAG00000006324	-0.218988834	-0.256659252	0.037670418
A0AAA9TSM8	KCTD3	A0AAA9TSM8	ENSBTAG00000006186	-0.243383318	-0.124781625	-0.118601694
Q2KI76	SELENOS	bta:512276; Q2KI76	ENSBTAG00000000640	-0.140862536	-0.334564921	0.193702385
Q6XBT4	USF1	bta:407239; Q6XBT4	ENSBTAG00000017873	0.263555328	0.195572824	0.067982505
Q0IIH8	MINDY3	bta:538509; Q0IIH8	ENSBTAG00000010387	0.078563669	0.082885275	-0.004321606
F1N0P9	GID4	F1N0P9	ENSBTAG00000008802	0.387730153	0.247204177	0.140525977
A0A452DI09	UBE2T	A0A452DI09	ENSBTAG00000004790	-0.377670647	-0.287638446	-0.090032201
F6QEF9	HMGA1	F6QEF9	ENSBTAG00000034529	0.214594051	0.27583168	-0.06123763
A0A3Q1LVW6	ZDHHC13	A0A3Q1LVW6; bta:514176	ENSBTAG00000020642	0.077919886	0.040249468	0.037670418
A1L530	SLC5A6	A1L530; bta:516021		0.078233288	-0.004391769	0.082625057
A6QLE9	MTMR14	A6QLE9; bta:514596	ENSBTAG00000009765	0.193086472	0.214550981	-0.021464509
A0A3Q1MA68	GRAMD4	A0A3Q1MA68	ENSBTAG00000021803	-0.037142004	0.032505769	-0.069647773
A0A452DJ79	POP7	A0A452DJ79	ENSBTAG00000026248	0.044020413	-0.015496336	0.059516749
A0AAA9TSB6	NEK1	A0AAA9TSB6	ENSBTAG00000026915	-0.130265908	-0.202178149	0.071912241
A5PK74	RIOX1	A5PK74; bta:511031	ENSBTAG00000025372	-0.111283334	-0.080980573	-0.03030276
F1MS84	NOVA2	F1MS84	ENSBTAG00000013157	0.067114196	0.059809395	0.007304801
A0AAA9T844	ZNG1	A0AAA9T844	ENSBTAG00000000269	-0.235605917	0.040949628	-0.276555545
F1MSK1	PRR12	F1MSK1	ENSBTAG00000014454	-0.266246711	-0.096981529	-0.169265182
A0A3Q1M1T9	PRORP	A0A3Q1M1T9	ENSBTAG00000009682	-0.041941763	-0.128163881	0.086222118
Q3ZBP4	RCAN1	bta:539640; Q3ZBP4	ENSBTAG00000020035	0.136002615	-0.189953482	0.325956097
Q2T9X3	SPRYD7	bta:615298; Q2T9X3		0.017487427	0.080861833	-0.063374406
F6QMY2	THY1	F6QMY2	ENSBTAG00000019627	-0.252442995	-0.139403057	-0.113039938
Q0V8R7	NSUN4	bta:618779; Q0V8R7		-0.029564458	0.028970753	-0.058535211
A0AAA9SHW9	WDR19	A0AAA9SHW9	ENSBTAG00000014512	-0.314696526	-0.395038634	0.080342108
Q0IIB7	C13H20ORF7	bta:522230; Q0IIB7		0.022490887	-0.005343321	0.027834208
A0A3Q1MMH9	ZNF579	A0A3Q1MMH9; bta:618680	ENSBTAG00000051917	-0.001433378	0.076720083	-0.078153461
A0AAA9RVZ8	SMAP2	A0AAA9RVZ8	ENSBTAG00000021922	0.062941063	0.322892786	-0.259951723
A0A452DIP3	DNAJC21	A0A452DIP3	ENSBTAG00000015648	0.166403163	0.195656968	-0.029253805
Q5EA55	GOLGA7	bta:616809; Q0VCT8; Q5EA55	ENSBTAG00000027626	0.072431582	0.081820086	-0.009388504
A0AAA9TFH8	MLLT11	A0AAA9TFH8	ENSBTAG00000015369	0.697367229	0.128502809	0.56886442
A0AAA9TBX6	CARHSP1	A0AAA9TBX6	ENSBTAG00000069945	-0.057667429	-0.029747343	-0.027920085
A0A452DIX7	WDR6	A0A452DIX7	ENSBTAG00000018910	-0.186277207	-0.21191909	0.025641883
E1BMA5	UCK2	E1BMA5	ENSBTAG00000044150	0.003911512	0.011702863	-0.007791351
A0AAA9TBI6	BRD1	A0AAA9TBI6	ENSBTAG00000046760	-0.228983091	0.075577872	-0.304560963
A0AAA9T762	MED25	A0AAA9T762	ENSBTAG00000008518	-0.355942752	-0.001257251	-0.354685501
Q2KJ57	ABCD1	bta:515178; Q2KJ57	ENSBTAG00000020551	-0.341285038	0.073752461	-0.415037499
E1B7N1	APPBP2	bta:505734; E1B7N1	ENSBTAG00000004489	-0.00129564	-0.002592445	0.001296805
Q0VCQ5	KLHL7	bta:534697; Q0VCQ5	ENSBTAG00000016010	0.207472496	0.322268715	-0.114796219
A0AAA9SIC6	RENBP	A0AAA9SIC6	ENSBTAG00000047827	0.049958159	0.249455522	-0.199497364
F6QF86	ALG6	bta:510138; F6QF86	ENSBTAG00000030706	0.217334362	0.272907693	-0.055573331
A5PJP1	BLOC1S3	A5PJP1; bta:538539	ENSBTAG00000007070	-0.031804185	0.430680477	-0.462484662
A0A3Q1LJI0	CARD19	A0A3Q1LJI0	ENSBTAG00000011202	0.169505674	0.067571505	0.10193417
F1MWR2	SF3B4	bta:506123; F1MWR2	ENSBTAG00000012495	0.266128207	0.224803746	0.041324461
A0AAA9T2J5	PKP4	A0AAA9T2J5	ENSBTAG00000013773	0.008044789	0.101237757	-0.093192969
A5D9A8	SQLE	A5D9A8; bta:526535	ENSBTAG00000005498	0.029426114	-0.01759406	0.047020174
A5PKJ7	RND3	A5PKJ7; bta:783333	ENSBTAG00000039731	0.372379603	0.660107624	-0.287728021
A0A3Q8TMT3	NCR3LG1	A0A3Q8TMT3		0.473985271	0.286060786	0.187924486
A0AAA9T6T6	MED13L	A0AAA9T6T6; bta:517336	ENSBTAG00000001888	0.522734201	0.406925748	0.115808453
A0AAA9S0R0	PCOLCE	A0AAA9S0R0	ENSBTAG00000020528	-0.272525855	-0.113398327	-0.159127528
A5D7P0	CDC25C	A5D7P0; bta:507731		-0.136791843	-0.081764431	-0.055027412
E1BEL5	PSMC3IP	E1BEL5	ENSBTAG00000020907	-0.25176958	-0.105548839	-0.146220741
A0AAA9SQU5				-0.331514144	-0.306189987	-0.025324156
A0AAA9SUY7	DTNBP1	A0AAA9SUY7	ENSBTAG00000012939	-0.692774928	0.069328913	-0.762103841
A0A3Q1M9P7	JADE1	A0A3Q1M9P7	ENSBTAG00000017493	0.191584922	-0.082877815	0.274462737
A0A0F6QMJ3	C5	A0A0F6QMJ3		-0.286606113	0.052663879	-0.339269992
A0A3Q1MD51	GSDMD	A0A3Q1MD51	ENSBTAG00000021474	-0.102421065	-0.006066001	-0.096355064
A0A3Q1MIL2	CDCA3	A0A3Q1MIL2	ENSBTAG00000019777	0.004737919	-0.036850405	0.041588324
F1N5P3	PUS3	F1N5P3	ENSBTAG00000014339	-0.303668155	0.249519599	-0.553187754
A5PJK1	TMPPE	A5PJK1; bta:527730		-0.072974032	0.035147944	-0.108121975
F1N6R4	BNIP2	F1N6R4	ENSBTAG00000013493	0.013631866	-0.013761901	0.027393767
Q08DN6	CDC42EP2	bta:507966; Q08DN6	ENSBTAG00000032089	0.223147166	0.540238056	-0.31709089
A0AAA9T8E2	ABCA1	A0AAA9T8E2	ENSBTAG00000020661	-0.594264242	0.447129275	-1.041393516
Q2KJF3	NFU1	bta:615964; Q2KJF3	ENSBTAG00000020141	0.584962501	-0.018249372	0.603211873
A0AAF6DMI3	WDR54	A0AAF6DMI3	ENSBTAG00000000489	0.062031959	0.059389659	0.002642299
A0A5H2Q5V2				-0.138341326	0.186349671	-0.324690997
A0AAF6ZAV2	PROS1	A0AAF6ZAV2	ENSBTAG00000023652	-0.241644293	-0.026835043	-0.21480925
A0AAA9TNM4	CPNE8	A0AAA9TNM4	ENSBTAG00000020914	-0.498453507	-0.425521985	-0.072931522
A0AAA9S256	CLCN5	A0AAA9S256	ENSBTAG00000001507	0.14908561	0.05246742	0.09661819
A0A3Q1MGN4	DSE	A0A3Q1MGN4	ENSBTAG00000005205	-0.185920165	-0.334854269	0.148934105
A0AAA9T6F6	EMC10	A0AAA9T6F6	ENSBTAG00000045510	0.053695008	-0.075399778	0.129094786
A0A3Q1LV95	LPIN2	A0A3Q1LV95	ENSBTAG00000012060	0.032789935	0.162906371	-0.130116436
Q2HJI2	NDUFAF3	bta:511968; Q2HJI2		0.134092896	0.246283042	-0.112190146
A0AAA9S9Y9	MEAF6	A0AAA9S9Y9	ENSBTAG00000010506	0.053040963	-0.008788017	0.06182898
A0A3Q1M8L1	DOCK4	A0A3Q1M8L1	ENSBTAG00000021182	-0.046524281	-0.176780666	0.130256386
Q2YDM9	LOC780876	bta:780876; Q2YDM9		0.102525716	-0.040984099	0.143509815
F1MMS1	TAF4	F1MMS1	ENSBTAG00000047991	0.015080573	-0.042303285	0.057383858
Q05B67	FAM210A	bta:511554; Q05B67	ENSBTAG00000009141	0.15415019	0.09646284	0.05768735
A0A3Q1LNF9	KIF18A	A0A3Q1LNF9; bta:537566	ENSBTAG00000002117	-0.169925001	0.311896126	-0.481821128
A0AAA9TXR6	NCS1	A0AAA9TXR6	ENSBTAG00000008726	0.230954435	0.157146397	0.073808038
A0A3Q1LY14	MAGI1	A0A3Q1LY14	ENSBTAG00000010581	-0.005270125	0.166267236	-0.171537361
A0AAA9TTA4	FAM234B	A0AAA9TTA4; bta:512120	ENSBTAG00000014322	0.07836165	0.05621601	0.022145641
A0A3Q1MTI0	KANSL3	A0A3Q1MTI0	ENSBTAG00000004497	-0.008553553	0.197611331	-0.206164884
A0A3Q1LXK7	LOC132344161	A0A3Q1LXK7	ENSBTAG00000049681	-0.213281371	-0.252945588	0.039664218
P50227	SULT1A1	bta:282485; P50227; Q3T0S9	ENSBTAG00000008635	-0.52016338	-0.455442802	-0.064720578
A0A3Q1NAS1	LOC524576	A0A3Q1NAS1; bta:524576	ENSBTAG00000050417	-0.509013647	-0.293242142	-0.215771505
A0AAA9S8I5	SMYD2	A0AAA9S8I5	ENSBTAG00000013166	-0.083270619	0.199308808	-0.282579427
F1CYZ1	MEF2D	bta:100336730; F1CYZ1		-0.356203365	-0.235604235	-0.12059913
A7YY73	MAFF	A7YY73; bta:617914	ENSBTAG00000021435	0.079404718	0.125947304	-0.046542586
A0A3Q1LX97	CKM	A0A3Q1LX97	ENSBTAG00000013921	-0.130308022	0.053637964	-0.183945986
A0AAA9SR01	ACTR5	A0AAA9SR01; bta:100297865	ENSBTAG00000048262	-0.182636053	-0.14172811	-0.040907944
A0A140T8A1	HSPB6	A0A140T8A1	ENSBTAG00000018598	0.471279234	0.481729385	-0.010450151
A0AAA9TSC2	STXBP4	A0AAA9TSC2	ENSBTAG00000016078	0.046114276	0.094794641	-0.048680365
A0A3Q1LT19				0.186860871	0.517525735	-0.330664864
F1MZF2	DSC2	F1MZF2	ENSBTAG00000040584	-0.422921098	-0.352218107	-0.070702991
F1MUK6	COMMD3	F1MUK6	ENSBTAG00000003589	0.808922558	0.46520918	0.343713378
A2VDR2	ACBD6	A2VDR2; bta:618245		-0.001318132	0.04666806	-0.047986191
A0AAA9SFB8	AFG1L	A0AAA9SFB8	ENSBTAG00000014592	-0.171233738	0.064325327	-0.235559065
E1BJL3	BACH1	bta:531350; E1BJL3	ENSBTAG00000004139	-0.518923739	-0.204113688	-0.314810052
E1B7I8	TELO2	E1B7I8	ENSBTAG00000019086	-0.051024003	0.039829427	-0.09085343
A0A3Q1MW68	PATJ	A0A3Q1MW68	ENSBTAG00000021975	0.043189606	0.183711953	-0.140522347
A3KN05	MAIP1	A3KN05; bta:510566	ENSBTAG00000019231	0.039702424	0.090490534	-0.05078811
A0A3Q1MDP7	WDR75	A0A3Q1MDP7	ENSBTAG00000003002	-0.199511797	-0.310668977	0.11115718
A0AAA9SCA8	DGKZ	A0AAA9SCA8	ENSBTAG00000013636	-0.179190271	-0.148694323	-0.030495948
Q5EAE2	TADA3	bta:510184; Q5EAE2	ENSBTAG00000007961	0.074515213	0.024786679	0.049728534
A0AAA9TL75	DUS4L	A0AAA9TL75	ENSBTAG00000021849	0.303603437	0.403611892	-0.100008456
Q71SS4	DERL1	bta:404121; Q1RMX8; Q71SS4	ENSBTAG00000020693	0.06608919	-0.147002412	0.213091603
Q2TBI1	NSMCE4A	bta:527312; Q2TBI1	ENSBTAG00000019166	-0.166533154	-0.040601133	-0.125932021
Q17QV2	MON1A	bta:520286; Q17QV2; Q5E9N7	ENSBTAG00000020047	-0.096162728	-0.023059016	-0.073103713
E1BKK9	SELENOM	E1BKK9	ENSBTAG00000011279	0.148981741	0.108961809	0.040019932
A0A3Q1LVN6	PRDM2	A0A3Q1LVN6	ENSBTAG00000020552	-0.151394875	-0.055817215	-0.09557766
P07995	INHBA	A0JN81; bta:281867; P07995	ENSBTAG00000056438	-0.654524571	-0.499649727	-0.154874844
A0AAA9S209	FAM120B	A0AAA9S209	ENSBTAG00000003884	-0.295913622	0.475943516	-0.771857139
A0A452DIT2	ZDHHC20	A0A452DIT2	ENSBTAG00000006177	0.004427712	-0.187585943	0.192013655
A0AAA9T2G8	ADGRG1	A0AAA9T2G8	ENSBTAG00000038141	-0.031160931	-0.300509111	0.26934818
B0JYN4	CIAO2A	B0JYN4	ENSBTAG00000002012	0.107888062	0.060769638	0.047118425
A0A452DK75	SLC35A4	A0A452DK75	ENSBTAG00000030584	0.057685907	0.026911154	0.030774753
A0AAA9TD16	NDRG2	A0AAA9TD16	ENSBTAG00000000843	0.024587879	0.163915455	-0.139327576
G3MWJ1	SFR1	G3MWJ1	ENSBTAG00000047450	0.130137947	0.472973064	-0.342835117
A0A452DJ07	PRR14	A0A452DJ07	ENSBTAG00000009099	-0.132857159	0.014034109	-0.146891268
F1N1U0	ATF7	F1N1U0	ENSBTAG00000005863	0.189428365	0.052065723	0.137362642
Q1L836				0.163498732	0.11611123	0.047387503
Q1LZ86	ABHD6	bta:505283; Q1LZ86	ENSBTAG00000016615	0.267150514	0.225451223	0.041699291
Q08DX0	SNX29	bta:518366; Q08DX0		-0.118644496	-0.114071767	-0.00457273
A0AAA9SKQ5	SKA2	A0AAA9SKQ5	ENSBTAG00000021680	0.139672292	0.050273206	0.089399085
A0A3Q1LXS8	GGTA1	A0A3Q1LXS8	ENSBTAG00000012090	-0.083547791	-0.152960014	0.069412223
A0AAA9RZP1	TMEM259	A0AAA9RZP1	ENSBTAG00000011351	-0.085220133	-0.240724244	0.155504111
E1BNI4	MBOAT1	bta:541284; E1BNI4	ENSBTAG00000016519	0.200271022	0.147370685	0.052900337
F1MZ14	GCH1	F1MZ14	ENSBTAG00000040151	-0.45194506	-0.113682023	-0.338263037
A0AAA9SZ35	BRD8	A0AAA9SZ35	ENSBTAG00000008753	-0.10120377	0.157390365	-0.258594135
A0A452DIL7	ERI3	A0A452DIL7	ENSBTAG00000015559	-0.045667751	0.154389222	-0.200056973
G1K200	LOXL4	G1K200	ENSBTAG00000020895	0.364263681	0.455149763	-0.090885882
E1B8Z0	BRPF3	bta:507996; E1B8Z0	ENSBTAG00000013316	0.486755888	0.13665712	0.350098768
A6QNK8	TAPBPL	A6QNK8; bta:529141		0.004478105	0.054281661	-0.049803556
A0AAF6YIN8	SH3RF1	A0AAF6YIN8	ENSBTAG00000001508	0.201763723	0.293573987	-0.091810264
A0AAF6YWK3	SLC43A2	A0AAF6YWK3	ENSBTAG00000011482	-0.430446939	-0.20170571	-0.228741228

Accession	Gene_Symbol	Gene_ID	Ensembl_Gene ID	log2(ratio(OV24H/OV0H))	log2(ratio(OV24R/OV0H))	log2(ratio(OV24H/OV24R))
A4FV30	CCDC8	A4FV30; bta:616838		-0.308489721	-0.253806048	-0.054683673
Q0P5B2	METTL2	bta:506221; Q0P5B2		0.021127711	-0.100730632	0.121858343
Q32PH9	CDC7	bta:516094; F1N271; Q32PH9	ENSBTAG00000016307	-0.066972554	-0.092118202	0.025145648
A0AAA9SQ21	PLPP2	A0AAA9SQ21	ENSBTAG00000000717	-0.161096242	0.114486015	-0.275582257
A0AAA9TIE7	TNFAIP8L1	A0AAA9TIE7	ENSBTAG00000037765	-0.548959652	-0.533306387	-0.015653264
A0A3Q1LRV8	RNF14	A0A3Q1LRV8	ENSBTAG00000005249	-0.214012756	-0.032580654	-0.181432102
A0AAA9TKX2	IFT74	A0AAA9TKX2	ENSBTAG00000019568	-0.166795995	-0.120657457	-0.046138538
F6RP53	GEM	bta:538437; F6RP53	ENSBTAG00000007596	-0.184424571	-0.277883762	0.093459191
A0AAA9TIC4	SMIM12	A0AAA9TIC4	ENSBTAG00000014235	0.381870635	0.206567454	0.175303181
E1BBE2	EREG	E1BBE2	ENSBTAG00000010273	-0.150630312	0.100594791	-0.251225104
A0A3Q1LRX5	LSM6	A0A3Q1LRX5	ENSBTAG00000014060	-0.065050549	-0.141122023	0.076071474
A6QP01	SELENOT	A6QP01; bta:783831		0.063488826	0.113433257	-0.049944431
A0A3Q1MS65	TAF6	A0A3Q1MS65	ENSBTAG00000030966	-0.056413309	-0.043197689	-0.01321562
A0AAA9TQ82	KIAA0586	A0AAA9TQ82	ENSBTAG00000004631	0.344559069	0.054553379	0.29000569
A0AAA9SLG7	YPEL5	A0AAA9SLG7	ENSBTAG00000023744	0.090197809	0.269483657	-0.179285848
Q08DA5	PANK3	bta:510749; Q08DA5	ENSBTAG00000011895	0.249269486	0.133891624	0.115377862
A0A3Q1LLF3	BOLA3	A0A3Q1LLF3	ENSBTAG00000050675	0.07283288	0.149199021	-0.076366141
A0AAA9TU07	GPX3	A0AAA9TU07	ENSBTAG00000043553	-0.201811544	0.038567656	-0.2403792
Q148D6	MPDU1	bta:504961; Q148D6		-0.018916091	0.103193185	-0.122109276
A0A3Q1NJR3	PEX16	A0A3Q1NJR3	ENSBTAG00000003126	-0.512431616	-0.371527609	-0.140904007
A0AAA9U0M9				-0.202729849	0.404620883	-0.607350732
E1BLR4	RHBDF2	E1BLR4	ENSBTAG00000003436	-0.229383468	-0.273533721	0.044150253
F1MYF2	MMGT1	F1MYF2	ENSBTAG00000025297	0.044870272	0.078002512	-0.03313224
A0AAA9RX88	CENPN	A0AAA9RX88	ENSBTAG00000011635	-0.044087754	0.086968401	-0.131056155
A0AAF6YS06	TONSL	A0AAF6YS06	ENSBTAG00000007749	-0.071352433	-0.136615074	0.06526264
G3N340	DUS1L	G3N340	ENSBTAG00000047760	-0.400989725	0.225615422	-0.626605147
E1BGZ3	ARID3A	bta:511283; E1BGZ3	ENSBTAG00000008607	0.247749677	0.23632233	0.011427347
A0AAA9THC7	WWC3	A0AAA9THC7	ENSBTAG00000013824	-0.083926082	-0.016678741	-0.067247341
E1BI78	YBEY	E1BI78	ENSBTAG00000003161	-0.430750842	-0.155430455	-0.275320387
A0AAA9SQR2				0.37939312	1.060137702	-0.680744582
A2VE81	OCLN	A2VE81; bta:512405	ENSBTAG00000000561	0.736537431	0.541504545	0.195032886
Q3ZC04	MRPL57	bta:615666; Q3ZC04		-0.011789198	0.097871383	-0.109660581
A0AAF6Z9T9	FSTL3	A0AAF6Z9T9	ENSBTAG00000003018	0.376416205	0.070389328	0.306026877
A0A3Q1MVF2	SLC6A6	A0A3Q1MVF2	ENSBTAG00000011088	-0.729796521	-0.323334916	-0.406461605
F1ME22	ADAM19	bta:510907; F1ME22	ENSBTAG00000003836	-0.251836199	-0.101879614	-0.149956585
A0A3Q1MFX1	SENP1	A0A3Q1MFX1	ENSBTAG00000012423	0.391662991	0.497642777	-0.105979786
E1BF46	ZNF407	bta:530360; E1BF46	ENSBTAG00000022887	-0.061270143	-0.06754274	0.006272597
A4IFA0	GAS2L1	A4IFA0; bta:518935		-0.206893524	-0.141969406	-0.064924117
P10575	GLRX	bta:515416; P10575; Q32KQ3		0.181526288	-0.078210528	0.259736816
F1MTX9	SLC38A1	F1MTX9	ENSBTAG00000000859	-0.623931553	-0.485915131	-0.138016421
G5E641				-0.643333064	-0.410394125	-0.232938938
P80221	CXCL6	A5PJH0; bta:281735; P80221; Q9GKP5	ENSBTAG00000009812	-0.288710318	-0.308549065	0.019838747
Q0P5L5	SUMF1	bta:536435; Q0P5L5	ENSBTAG00000039855	-0.108747663	0.087742895	-0.196490558
E1BA23	AMPD3	E1BA23	ENSBTAG00000015821	-0.053111336	0.092566119	-0.145677455
A0AAA9S7Y7	THBS2	A0AAA9S7Y7	ENSBTAG00000018810	0.048498518	0.239901071	-0.191402554
F1MN32	ITPKC	F1MN32	ENSBTAG00000019465	-0.291034087	-0.136130182	-0.154903905
F1MKZ2	ASAP2	F1MKZ2	ENSBTAG00000002329	-0.10371135	0.011864335	-0.115575685
A2VDL2	SLC2A3	A2VDL2; bta:282358	ENSBTAG00000004556	0.115372222	0.058839092	0.056533129
A0AAA9TAP8	CBLL1	A0AAA9TAP8	ENSBTAG00000018348	0.170922785	1.002692433	-0.831769648
Q3ZC85	PLVAP	bta:524990; F1MHE4; Q3ZC85	ENSBTAG00000005434	-0.136698673	0.030273011	-0.166971684
Q5E975	TMEM230	bta:515498; Q5E975	ENSBTAG00000006063	0.404769164	0.313033101	0.091736063
A0A3Q1M2Z5	TGFB2	A0A3Q1M2Z5	ENSBTAG00000005359	-0.054564361	0.024045474	-0.078609835
Q1JPA6	NAT1	bta:512603; Q1JPA6	ENSBTAG00000016473	0.081772371	0.129204201	-0.04743183
A0A3Q1LKR3	AADAT	A0A3Q1LKR3	ENSBTAG00000050967	-0.018599678	-0.01361627	-0.004983408
E1BNC4	DIPK2A	bta:509304; E1BNC4	ENSBTAG00000007933	-0.059378547	0.11189288	-0.171271427
A0AAA9SI10	LDAH	A0AAA9SI10	ENSBTAG00000031014	0.221008541	0.301777487	-0.080768946
G3N1U1	CENPV	G3N1U1	ENSBTAG00000013274	-0.610378242	0.112706077	-0.723084319
A0AAA9S879	ARHGAP27	A0AAA9S879	ENSBTAG00000000571	-0.122205932	0.333598088	-0.45580402
A6H720	NCK2	A6H720; bta:526430	ENSBTAG00000015205	-0.034976138	0.268782202	-0.303758341
A0AAA9TA99				-0.015120086	0.050496402	-0.065616489
F1MEN1				-0.856899109	-0.306319602	-0.550579507
A0A3Q1M2G9	LGALS8	A0A3Q1M2G9	ENSBTAG00000013544	0.064014174	-0.054447784	0.118461958
O18824	SCARB1	bta:282346; O18824	ENSBTAG00000014269	-0.130268801	0.00314999	-0.133418792
E1BQ22	TRIM68	bta:538657; E1BQ22	ENSBTAG00000011332	-0.165608321	-0.076082393	-0.089525929
A0A3Q1M2I3	CANT1	A0A3Q1M2I3	ENSBTAG00000009991	0.059220423	0.068598756	-0.009378332
A0AAA9T599	UBXN2B	A0AAA9T599	ENSBTAG00000009138	0.210655595	0.266618743	-0.055963148
P82927	MRPL42	bta:614148; P82927; Q32LA9	ENSBTAG00000002708	0.055024701	0.077346369	-0.022321668
A0AAA9TU40	AKT1S1	A0AAA9TU40	ENSBTAG00000005763	0.03285216	0.137251766	-0.104399606
Q05B70	GRAMD3	bta:505627; Q05B70		0.130502024	0.092575765	0.03792626
A8E640	GGCX	A8E640		-0.503020887	-0.242939799	-0.260081088
A0AAA9T723	C20H5orf22	A0AAA9T723	ENSBTAG00000017569	0.100145564	0.199112014	-0.098966451
Q0VBY5	WDR41	bta:514919; Q0VBY5	ENSBTAG00000020212	-0.255122362	-0.407402338	0.152279976
Q3SZU5	SPCS3	bta:767917; Q3SZU5		0.13289427	0.112971813	0.019922458
A0AAA9SG01	ERN1	A0AAA9SG01	ENSBTAG00000016799	0.087110664	0.151404341	-0.064293677
Q08DX7	SPNS1	bta:505997; Q08DX7	ENSBTAG00000014653	0.250283704	0.444967705	-0.194684002
A0AAA9RXW3	ALG5	A0AAA9RXW3	ENSBTAG00000011850	0.108961809	0.101948208	0.007013602
F6QLT0				-0.144265335	-0.466847565	0.32258223
A0AAA9SLB4	YIPF6	A0AAA9SLB4	ENSBTAG00000001977	-0.074159111	0.056120086	-0.130279197
A0AAA9TN79	IMP3	A0AAA9TN79	ENSBTAG00000069591	-0.517848305	-0.549042926	0.031194622
A0A3Q1LUT5	PTPRA	A0A3Q1LUT5	ENSBTAG00000011425	-0.186596033	-0.120236582	-0.066359451
Q58D98	POLR3D	bta:508049; Q58D98		0.280529021	0.293731203	-0.013202182
Q17QM4	SELENOI	bta:506381; Q17QM4	ENSBTAG00000007837	0.40251665	0.369622041	0.032894609
E1BGW4	USP36	bta:504581; E1BGW4	ENSBTAG00000021505	-0.012291406	0.096165963	-0.108457369
A0AAA9TK62	EXTL2	A0AAA9TK62	ENSBTAG00000019026	-0.436276296	-0.226749501	-0.209526795
Q1JPF1	NKIRAS2	bta:509053; Q1JPF1	ENSBTAG00000002657	-0.262124475	0.112323018	-0.374447493
A0AAA9SI71	LIAS	A0AAA9SI71	ENSBTAG00000014520	0.109110999	0.135883428	-0.026772429
A0A3Q1N4J1	LARP1B	A0A3Q1N4J1	ENSBTAG00000012135	0.094859186	0.181121425	-0.086262239
A0AAA9SW74	SNTA1	A0AAA9SW74	ENSBTAG00000000512	-0.105374686	-0.001431955	-0.10394273
A0AAA9RU66	CD82	A0AAA9RU66	ENSBTAG00000031252	-0.218748832	-0.170920307	-0.04782525
A0A3Q1LM90	ATP11B	A0A3Q1LM90; bta:614392	ENSBTAG00000000290	-0.244859761	-0.112069006	-0.132790755
F1MZJ7	CCDC85C	F1MZJ7	ENSBTAG00000025181	-0.147060893	-0.087078788	-0.059982105
A0AAA9T585	HACL1	A0AAA9T585	ENSBTAG00000003718	-0.127561081	-0.110453825	-0.017107256
A0AAA9S463	COX10	A0AAA9S463	ENSBTAG00000015294	0.056285173	-0.153727774	0.210012947
Q3T061	COX7A2L	bta:540225; Q3T061	ENSBTAG00000000705	-0.069193442	-0.081055339	0.011861897
A0AAA9SXK1	GLIPR1	A0AAA9SXK1	ENSBTAG00000011590	-0.428065356	-0.425972977	-0.002092379
A0AAF6YGN5	TUSC2	A0AAF6YGN5	ENSBTAG00000058889	0.152681052	0.075948853	0.076732198
A0A3S5ZPJ2	TNIP1	A0A3S5ZPJ2	ENSBTAG00000012671	-0.066880807	0.160636647	-0.227517454
F6RWD2	DHDDS	F6RWD2	ENSBTAG00000003235	-0.553815986	-0.635307484	0.081491498
A0AAA9TD35	TMEM181	A0AAA9TD35	ENSBTAG00000063575	0.004789034	0.037874399	-0.033085366
A0A3Q1LL22	ASXL2	A0A3Q1LL22; bta:100140419	ENSBTAG00000019983	-0.070702991	-0.025311089	-0.045391903
A0AAA9TZT6	DPH1	A0AAA9TZT6	ENSBTAG00000009376	-0.366745755	-0.199411766	-0.167333989
E1BKA3	AJUBA	bta:519937; E1BKA3	ENSBTAG00000012724	0.24421323	-0.126184839	0.37039807
G3MXX2	SERPINA7	G3MXX2	ENSBTAG00000047655	0.070915955	0.575980302	-0.505064346
A0A452DI74	CALCOCO2	A0A452DI74	ENSBTAG00000007935	0.325533794	-0.075536103	0.401069897
A0A3Q1MAU4	FASTKD5	A0A3Q1MAU4	ENSBTAG00000051021	-0.186848304	-0.185253285	-0.00159502
F1MKI1	ATP7B	bta:518075; F1MKI1	ENSBTAG00000010353	-0.353735422	-0.179037024	-0.174698398
A0A3Q1LGR3	KDM1B	A0A3Q1LGR3	ENSBTAG00000014608	0.252023625	0.320195128	-0.068171503
Q9GJW7	GABARAP	bta:327715; Q3SZ82; Q9GJW7	ENSBTAG00000014883	0.281391675	0.175326616	0.106065059
E1BJ69	KNSTRN	E1BJ69	ENSBTAG00000032340	0.035871939	-0.274411496	0.310283434
A0A452DJ15	MRPL27	A0A452DJ15	ENSBTAG00000021720	-0.290535486	-0.252231598	-0.038303888
A0AAA9S9R1				-0.793141063	-0.469799254	-0.323341809
Q32PI9	MPZL1	A2SZV4; bta:539387; Q32PI9	ENSBTAG00000002823	-0.22010061	-0.336914275	0.116813665
A0AAA9SJD1	AGL	A0AAA9SJD1	ENSBTAG00000006491	-0.006879818	0	-0.006879818
Q0VCF8	SOX4	bta:768313; Q0VCF8	ENSBTAG00000051245	-0.121621137	-0.765355318	0.643734181
A0AAA9S9S8	SLC35F6	A0AAA9S9S8	ENSBTAG00000018761	0.045710054	0.215312485	-0.169602431
Q148I0				0.176927349	-0.361465412	0.538392761
E1BL88	ABCB10	E1BL88	ENSBTAG00000017053	-0.23800851	-0.307841457	0.069832947
A0A3Q1MET8	FKRP	A0A3Q1MET8	ENSBTAG00000050243	-0.07952034	0.088265751	-0.167786091
F1MVT1	FLRT2	bta:539905; F1MVT1	ENSBTAG00000064499	0.233740099	0.027526331	0.206213768
A0A0S2I887	L3MBTL3	A0A0S2I887		-0.498938757	-0.463104158	-0.035834599
F1N7N4	ADAMTS16	F1N7N4	ENSBTAG00000018460	0.442798834	0.310672237	0.132126597
G3MYQ0	STYX	bta:616825; G3MYQ0	ENSBTAG00000046956	-0.04548775	-0.13484727	0.08935952
A0AAA9T6H1	MIS18BP1	A0AAA9T6H1	ENSBTAG00000002613	-0.267610483	0.398246372	-0.665856855
G5E5D9	NKAP	G5E5D9	ENSBTAG00000010884	0.057528147	-0.110594612	0.168122759
F1N180	MOB2	F1N180	ENSBTAG00000011249	0.25147135	0.315170729	-0.063699379
A0A3Q1M2U6	TMEM87B	A0A3Q1M2U6	ENSBTAG00000012099	-0.312820891	0.213741234	-0.526562125
Q9GKZ4	TRAM1	bta:281546; Q3SX16; Q9GKZ4	ENSBTAG00000007562	0.154559931	0.030073723	0.124486208
A0AAF6YKL2	IMP4	A0AAF6YKL2	ENSBTAG00000003225	-0.12254962	-0.20977736	0.087227741
A0AAA9S2W8	AMFR	A0AAA9S2W8	ENSBTAG00000003798	-0.027933013	0	-0.027933013
A7MB15	FZD1	A7MB15; bta:445417	ENSBTAG00000002107	0.259256366	0.252361753	0.006894613
Q32L67	GLRX2	bta:513762; Q32L67	ENSBTAG00000015972	0.012010018	0.050366133	-0.038356115
E1BBA1	GGNBP2	E1BBA1	ENSBTAG00000010290	-0.469867353	-0.321307447	-0.148559906
A0AAA9TKC4	KYAT1	A0AAA9TKC4	ENSBTAG00000036101	-0.15674805	-0.0096594	-0.14708865
E1BAX6	CYP7B1	E1BAX6	ENSBTAG00000001299	0.085630072	0.27337635	-0.187746279
E1BNB5	FOSL1	E1BNB5	ENSBTAG00000006194	-0.424966617	-0.334225454	-0.090741163
Q5EAE6	DAP	bta:616066; Q3SYY4; Q5EAE6	ENSBTAG00000006346	0.231325546	0.422467033	-0.191141487
A0AAA9STX7				0.041183435	-0.081643238	0.122826673
A4IFD8	METTL14	A4IFD8; bta:531382		-0.034373044	0.028323757	-0.062696801
A0A3Q1NBI1	VAV3	A0A3Q1NBI1	ENSBTAG00000031575	-0.092081896	0.075101455	-0.167183351
A0A3Q1MUW9	FBXL20	A0A3Q1MUW9	ENSBTAG00000008371	0.495310815	0.519137865	-0.023827049
Q5EA45	FOXRED1	bta:510097; Q58CP5; Q5EA45; Q5EAA3		0.089772474	0.079183839	0.010588635
F1MCM1	MTRF1L	F1MCM1	ENSBTAG00000009275	-0.197564914	-0.119408703	-0.078156211
Q1LZH1	MTARC2	bta:615506; Q1LZH1	ENSBTAG00000016277	0.045572173	0.029331532	0.016240641
Q08D81	WDR13	bta:523881; Q08D81	ENSBTAG00000018060	0.095286201	-0.115098408	0.210384609
E1B899	MIOS	bta:512321; E1B899	ENSBTAG00000013781	-0.060349656	0.114857103	-0.175206759
A0A3Q1LNT8	EML1	A0A3Q1LNT8	ENSBTAG00000013491	-0.005531106	0.02372607	-0.029257176
Q3MHV9	SERINC1	bta:539993; Q3MHV9	ENSBTAG00000000019	0.191459723	0.342632489	-0.151172765
A0AAF7AF97	DYNC2LI1	A0AAF7AF97	ENSBTAG00000013676	-0.335204027	-0.320639399	-0.014564628
A0A3Q1NM28	CEMIP	A0A3Q1NM28	ENSBTAG00000007431	0.008071042	-0.014233893	0.022304935
G3N2E9	KDM6A	bta:280952; G3N2E9	ENSBTAG00000003740	-0.141718746	-0.121051687	-0.020667059
Q3SX20	BORCS6	bta:539395; Q3SX20		-0.083831591	-0.174294601	0.09046301
F1MXW5	OXSM	F1MXW5	ENSBTAG00000007031	-0.339692374	-0.140677772	-0.199014603
Q0VCU3	CTSF	bta:509715; Q0VCU3	ENSBTAG00000010994	-0.145741633	0.367410865	-0.513152498
A0AAF7ABH3	BMI1	A0AAF7ABH3	ENSBTAG00000015584	0.012895684	0.02249236	-0.009596676
F6RWX3	SEC61B	F6RWX3	ENSBTAG00000002457	0.04077453	-0.033795852	0.074570382
F1N3J6	PIGW	F1N3J6	ENSBTAG00000034396	0.147914205	0.127294106	0.020620099
Q2KIR4	RPP25L	bta:512203; Q2KIR4	ENSBTAG00000016295	0.045758287	0.068104446	-0.022346159
A0A3Q1MDR6	CBX4	A0A3Q1MDR6	ENSBTAG00000049156	0.362154197	0.32678503	0.035369168
A0A3Q1M0N7	ECE1	A0A3Q1M0N7	ENSBTAG00000002977	-0.204744556	0.211571362	-0.416315918
A0A3Q1MJW8	C28H1orf131	A0A3Q1MJW8	ENSBTAG00000010765	0.03500645	0.036652423	-0.001645973
A4IFE2	RCHY1	A4IFE2; bta:540733	ENSBTAG00000007189	-0.105215033	-0.804430869	0.699215836
Q5E9J5	DHCR7	A6QR24; bta:514745; Q5E9J5	ENSBTAG00000016465	-0.145273893	-0.206550233	0.06127634
A5D7I4	EXT1	A5D7I4; bta:538602	ENSBTAG00000006209	-0.206734009	-0.301826889	0.09509288
A0AAA9TUJ0	RETREG1	A0AAA9TUJ0	ENSBTAG00000016444	0.101184634	0.031677212	0.069507423
F1MH50	PLEKHH2	bta:100299044; F1MH50	ENSBTAG00000032521	-0.024398628	-0.057143907	0.032745279
A0AAA9SJN1	TMEM63A	A0AAA9SJN1	ENSBTAG00000002854	0.001502025	0.171260212	-0.169758187
A0A3Q1M5S1	GTF2H3	A0A3Q1M5S1	ENSBTAG00000007270	0.212677267	0.170283569	0.042393698
E1BC04	CNST	bta:541076; E1BC04	ENSBTAG00000000511	-0.291498465	0.176280496	-0.467778961
A1A4L1	THEM4	A1A4L1; bta:787270		-0.273138364	-0.07680436	-0.196334005
A0A3Q1MBH1	MND1	A0A3Q1MBH1	ENSBTAG00000000281	-0.417094086	-0.289164138	-0.127929948
A0AAF6YSH2	LIMD2	A0AAF6YSH2	ENSBTAG00000008154	-0.018790794	0.08752385	-0.106314644
F1MEZ4	CCDC82	F1MEZ4	ENSBTAG00000006581	0.315440992	0.403064858	-0.087623865
A0AAA9T7B5	WIPI2	A0AAA9T7B5	ENSBTAG00000012049	0.138529805	0.027960081	0.110569724
J9JH84	RNF169	J9JH84	ENSBTAG00000000727	0.13118168	-0.131588762	0.262770442
Q1JPG0	MIGA2	bta:535315; Q1JPG0	ENSBTAG00000010653	0.004748315	0.268309348	-0.263561033
E1BCF2	CRLF3	E1BCF2	ENSBTAG00000018381	0.297904866	0.239844049	0.058060817
A0AAA9TN88	ATG13	A0AAA9TN88	ENSBTAG00000017325	0.303330876	0.519539426	-0.21620855
A0AAA9TV41	PHF19	A0AAA9TV41	ENSBTAG00000016958	-0.219371069	-0.33396825	0.11459718
F1MD95	CLSTN1	F1MD95	ENSBTAG00000011823	-0.171716947	0.199077714	-0.370794661
A0A3S5ZPL0	GNB5	A0A3S5ZPL0	ENSBTAG00000003762	-0.168089745	-0.089392377	-0.078697368
A0AAA9S597	GNPTG	A0AAA9S597	ENSBTAG00000014118	-0.123968678	-0.026819598	-0.097149081
F6PX95	ESS2	F6PX95	ENSBTAG00000018534	0.00569673	-0.240100296	0.245797026
Q2KJ19	DR1	bta:613828; Q2KJ19	ENSBTAG00000008737	0.149664851	0.034626343	0.115038508
A0A3Q1MIF8	NFIX	A0A3Q1MIF8	ENSBTAG00000018229	-0.711893572	0.099828874	-0.811722447
A5D7I7	RASA3	A5D7I7; bta:282532; F1N4G2		0.058386501	-0.038542676	0.096929178
E1BB75	EAF1	E1BB75	ENSBTAG00000019202	0.053882841	-0.023059016	0.076941857
Q3SWW7	IFRD2	bta:767939; Q3SWW7		0.03329444	0.205046795	-0.171752354
F1MRH9	PTPN21	F1MRH9	ENSBTAG00000018596	-0.068888553	-0.401883952	0.332995399
P00257	FDX1	A5D9I2; bta:281157; P00257; P08498; P12713; Q32KZ0	ENSBTAG00000011793	0.3950628	0.053111336	0.341951463

Accession	Gene_Symbol	Gene_ID	Ensembl_Gene ID	log2(ratio(OV24H/OV0H))	log2(ratio(OV24R/OV0H))	log2(ratio(OV24H/OV24R))
A0AAA9TNA0	SH2B1	A0AAA9TNA0	ENSBTAG00000019219	0.369056846	0.59528703	-0.226230184
A0AAA9TGF0	SENP6	A0AAA9TGF0	ENSBTAG00000005869	-0.138531241	-0.174518698	0.035987457
A0AAF6DLT7	TUBGCP5	A0AAF6DLT7	ENSBTAG00000014714	-0.051556611	-0.029234942	-0.022321668
F1MDC9	PRKCE	bta:507041; F1MDC9	ENSBTAG00000020614	0.02329662	0.419225296	-0.395928676
Q2KIA4	SCD5	bta:617419; Q2KIA4	ENSBTAG00000022449	-0.135351853	-0.048167282	-0.08718457
A0AAA9S414	GABARAPL1	A0AAA9S414	ENSBTAG00000011765	0.408464845	0.291889269	0.116575577
A0A3Q1LGW7	KRT76	A0A3Q1LGW7; bta:100336907	ENSBTAG00000038033	-0.215246507	0.703311897	-0.918558404
A0AAA9RRX3	RPE	A0AAA9RRX3	ENSBTAG00000054647	1.212729324	0.369146819	0.843582505
A0AAA9TVC3	CPTP	A0AAA9TVC3	ENSBTAG00000007623	0.085307313	0.028999872	0.056307441
Q32KP1	TSPAN31	bta:510619; Q32KP1	ENSBTAG00000007158	0.504065256	0.663805336	-0.15974008
Q2T9N4	PMF1	bta:616311; Q2T9N4	ENSBTAG00000009432	-0.189993877	-0.184706045	-0.005287831
A0A3Q1LLU2	RIN2	A0A3Q1LLU2	ENSBTAG00000020382	-0.299068012	-0.281846272	-0.017221741
A0AAA9TRF8				0.052512896	0.161268663	-0.108755768
E1BNL6	GMEB2	E1BNL6	ENSBTAG00000014769	0.017036854	0.196340558	-0.179303703
Q2HJB0	MBLAC1	bta:505636; Q2HJB0		0.260220887	0.077598995	0.182621893
A0A3Q1MJ94	SLC38A9	A0A3Q1MJ94	ENSBTAG00000033313	0.146066399	0.186024899	-0.0399585
A0AAA9TSJ0	STK19	A0AAA9TSJ0	ENSBTAG00000005589	0.020272395	0.17445466	-0.154182265
Q3B7N1	CHD1L	bta:524787; Q3B7N1		-0.074525375	-0.088086055	0.01356068
A0A3Q1LT06	TCP11L1	A0A3Q1LT06; bta:508910	ENSBTAG00000008613	-0.075288127	0.074288229	-0.149576356
A0A3Q1N6M3	MTNAP1	A0A3Q1N6M3	ENSBTAG00000006089	0.202511351	0.475855568	-0.273344217
A0AAF6Z521	CA4	A0AAF6Z521	ENSBTAG00000017969	0.033406877	-0.040702337	0.074109214
A0AAA9SRI3	MZT2B	A0AAA9SRI3	ENSBTAG00000014646	-0.286493379	0.253089219	-0.539582597
A0A3Q1MMK5	SNF8	A0A3Q1MMK5	ENSBTAG00000018803	0.029084492	0.13003388	-0.100949388
A0AAA9SW54	MAP1LC3A	A0AAA9SW54	ENSBTAG00000006135	0.175556007	-0.111748358	0.287304365
A0AAA9TT39	PPP1R11	A0AAA9TT39	ENSBTAG00000032247	0.477321778	0.36099766	0.116324118
Q56K13	SF3B5	bta:574400; Q56K13	ENSBTAG00000060547	0.3499684	-0.135987468	0.485955868
A5D962	PHF23	A5D962; bta:539774; Q58CU8	ENSBTAG00000014881	0.077652556	0.040965133	0.036687423
A0AAA9U0T8				-0.102738106	-0.510921934	0.408183828
Q3SZ87	SSR3	bta:767980; Q3SZ87	ENSBTAG00000018471	0.018859027	-0.045992117	0.064851144
Q3T0E3	COA3	bta:539832; Q3T0E3	ENSBTAG00000019911	0.163340348	0.081887201	0.081453147
A0AAA9TF96	POLE2	A0AAA9TF96	ENSBTAG00000004931	-0.19020328	0.062766087	-0.252969367
F6RM69	FZR1	F6RM69	ENSBTAG00000031387	0.096355064	-0.182981154	0.279336218
E1BDF7	ATXN1	E1BDF7	ENSBTAG00000019675	-0.053025709	-0.04311024	-0.009915469
Q2HJF1	MRPL53	bta:505728; Q2HJF1	ENSBTAG00000016599	-0.076505986	0.029812051	-0.106318037
A0A3Q1M9J6	RIC8B	A0A3Q1M9J6; bta:522873	ENSBTAG00000000147	0.181730339	0.03085228	0.15087806
Q9XS50	VEGFC	bta:282122; Q9XS50	ENSBTAG00000014737	-0.012866931	-0.004811661	-0.00805527
A1A4R3	DNAJC15	A1A4R3; bta:618047		-0.325793034	-0.005155557	-0.320637477
A0A3Q1MBI9	THBD	A0A3Q1MBI9	ENSBTAG00000048591	0.007253384	0.241790988	-0.234537604
A1A4J3	ZCCHC3	A1A4J3; bta:507116	ENSBTAG00000002116	-0.507021975	-0.264212597	-0.242809378
A0AAA9SBQ5				-0.59221224	-0.147056629	-0.445155611
F1MKU2	CBX2	F1MKU2	ENSBTAG00000038306	-0.227850409	-0.253149817	0.025299408
A0A3Q1MJ30	CERS6	A0A3Q1MJ30	ENSBTAG00000044179	0.256611868	0.096970088	0.15964178
A0A3Q1M496	ENTPD4	A0A3Q1M496	ENSBTAG00000006768	-0.151092588	-0.229010824	0.077918235
A6QP39	MSLN	A0A8J8Y870; A6QP39; bta:516237; M5FJX0		-0.308685518	-0.731064993	0.422379475
A0AAA9S3C7	KIAA0930	A0AAA9S3C7	ENSBTAG00000006776	0.368800278	0.327536483	0.041263795
E1BKM5	RECQL5	bta:512590; E1BKM5	ENSBTAG00000011715	0.050778823	0.053627185	-0.002848362
A0AAF6Z1T1	COQ5	A0AAF6Z1T1	ENSBTAG00000015761	-0.094029197	0.130464747	-0.224493944
F1MPI8	CDK8	F1MPI8	ENSBTAG00000016737	-0.493473485	-0.426787262	-0.066686223
A0AAA9SJD5	NOPCHAP1	A0AAA9SJD5	ENSBTAG00000021196	0.267695783	-0.001557145	0.269252929
A0A3Q1LKV4	KSR1	A0A3Q1LKV4	ENSBTAG00000006601	-0.414151051	-0.569966453	0.155815402
E1BB99	EPC1	E1BB99	ENSBTAG00000001552	-0.023896116	0.049501961	-0.073398078
Q1JPJ0	NDOR1	bta:513873; G3MXY0; Q1JPJ0		0.107023902	-0.021464509	0.128488411
A0A3Q1M5L8	ZFYVE19	A0A3Q1M5L8	ENSBTAG00000013485	0.377656765	0.475644264	-0.097987499
A0AAA9SAS8	FAM76B	A0AAA9SAS8; bta:519943	ENSBTAG00000020748	-0.027730956	0.158637512	-0.186368468
Q3ZC02	DCTN5	bta:506441; Q3ZC02	ENSBTAG00000006410	-0.289251183	-0.244984775	-0.044266408
A0AAA9S2M3	TTC39C	A0AAA9S2M3; bta:532895	ENSBTAG00000005635	-0.350132976	-0.054447784	-0.295685192
A0A3Q1LWI1	FKBP14	A0A3Q1LWI1	ENSBTAG00000007870	-0.121379239	-0.136533738	0.015154499
G3N184	NDEL1	bta:510769; G3N184	ENSBTAG00000011994	-0.042327929	0.327956677	-0.370284606
A0AAA9S208	DENND5B	A0AAA9S208	ENSBTAG00000020315	0.007217099	-0.081866445	0.089083544
A0FDH2	APOBEC3F	A0FDH2		-0.134075754	0.035205331	-0.169281085
A0AAA9TE30	DALRD3	A0AAA9TE30	ENSBTAG00000018913	-0.202374528	-0.059202254	-0.143172274
A0AAA9SB40	LOC784488	A0AAA9SB40	ENSBTAG00000060244	0.370111628	0.106550595	0.263561033
Q0VCU4	CD247	Q0VCU4		-0.055282436	-0.172639386	0.117356951
F1MUQ6	PRR11	F1MUQ6	ENSBTAG00000021681	-0.126712935	-0.173177979	0.046465045
A0AAA9S1F6	ANKRD52	A0AAA9S1F6; bta:521057	ENSBTAG00000011762	0.059195793	0.147766989	-0.088571197
F6RUM1	BUD13	F6RUM1	ENSBTAG00000016855	0.214443668	0.130761946	0.083681722
F1N535	NCAPG2	F1N535	ENSBTAG00000016131	-0.271774255	-0.070140394	-0.201633861
A0AAA9THX1	ATP5MF	A0AAA9THX1	ENSBTAG00000002094	0.023170848	-0.118801533	0.141972381
A0AAA9TA05	GEMIN2	A0AAA9TA05	ENSBTAG00000020930	0.003050096	0.00457273	-0.001522633
A0AAA9SQK2	MARVELD2	A0AAA9SQK2	ENSBTAG00000040001	-0.335204027	-0.194303666	-0.140900361
A0AAA9TWY3	NFATC3	A0AAA9TWY3	ENSBTAG00000004368	0.303928411	0.427620629	-0.123692217
F1N6L6	FAM76A	F1N6L6	ENSBTAG00000019614	-0.227084117	0.10078422	-0.327868336
Q2KJ84	TMED5	A5D9A9; bta:534351; Q2KJ84	ENSBTAG00000020180	0.448215106	0.359625803	0.088589304
F6RG44	TTI2	F6RG44	ENSBTAG00000015463	0.183711953	0.153605198	0.030106755
P15103	GLUL	bta:281199; O02850; P15103; Q3ZBU6; Q866Q7	ENSBTAG00000013631	0.139403057	-0.126957175	0.266360232
F1N1F1	CDAN1	bta:538940; F1N1F1	ENSBTAG00000005751	-0.349389457	-0.307227046	-0.042162412
A2VDR1	RAB40C	A0A8J8XK94; A2VDR1; bta:618031; M5FMU8		0.110633774	0.106367546	0.004266228
A0AAA9TQD4	UBE2J2	A0AAA9TQD4	ENSBTAG00000015882	0.022848589	0.026083333	-0.003234744
A0AAA9SAF0	CDCA7L	A0AAA9SAF0	ENSBTAG00000004976	-0.600101098	-0.48181708	-0.118284018
A0AAA9TNL9	SGF29	A0AAA9TNL9	ENSBTAG00000016883	0.297618642	0.406336884	-0.108718242
E1BNM5	ISG20	bta:506604; E1BNM5	ENSBTAG00000014762	-0.11617943	1.283943396	-1.400122825
Q148M8	ABT1	bta:539270; Q148M8	ENSBTAG00000010784	-0.097775216	-0.247499427	0.149724211
Q3T131	COQ3	bta:540298; Q3T131		-0.588909685	0.028620678	-0.617530363
A0A3Q1N4U1	KLHL13	A0A3Q1N4U1	ENSBTAG00000000501	0.045980653	0.008469858	0.037510795
A0AAA9T6V6	CYB5A	A0AAA9T6V6	ENSBTAG00000012012	0.63623948	0.197426693	0.438812787
E1BJ93	LEF1	bta:535399; E1BJ93	ENSBTAG00000006844	0.115115685	-0.125103733	0.240219418
A0A3Q1M3B6	RBMX2	A0A3Q1M3B6; bta:613705	ENSBTAG00000014771	0.080891452	0.092716888	-0.011825435
A0AAA9RRT9	NIT1	A0AAA9RRT9	ENSBTAG00000020153	0.032391146	0.265988654	-0.233597507
A0A3Q1M2H1	MMRN1	A0A3Q1M2H1	ENSBTAG00000010285	0.099318056	-0.011469527	0.110787583
Q5E9Z2	HABP2	bta:507993; Q3MHK6; Q5E9Z2	ENSBTAG00000019322	-0.373427109	0.002750611	-0.37617772
E1B883	RRN3	E1B883	ENSBTAG00000004804	-0.071790683	-0.036885068	-0.034905615
Q2KIJ5	KDSR	bta:505558; F1MLE5; Q2KIJ5	ENSBTAG00000007723	-0.047708009	0.050071155	-0.097779164
Q5E999	DUSP11	bta:508944; Q2KJ27; Q5E999		0.266764155	-0.278631261	0.545395416
A0AA95Z4A0	FABP4	A0AA95Z4A0		-0.148425423	0.123514536	-0.271939959
A0AAA9SYI5	SNX15	A0AAA9SYI5	ENSBTAG00000002243	0.040760779	0.243418618	-0.202657839
E1BA17	COL14A1	E1BA17	ENSBTAG00000013369	0.043029793	0.084813263	-0.04178347
A0AAA9TEN1	OTUD6B	A0AAA9TEN1	ENSBTAG00000003481	0.0116347	0.23526815	-0.223633449
F6PVT9	STEAP2	F6PVT9	ENSBTAG00000003506	-0.382333334	-0.322246184	-0.06008715
F1MQI4	TIMELESS	bta:522884; F1MQI4	ENSBTAG00000020459	-0.263625795	0.249171752	-0.512797548
A0AAA9T2J3	CYP4F8	A0AAA9T2J3	ENSBTAG00000048257	-0.112414465	0.069645862	-0.182060327
Q0VCV8	BTBD10	bta:505888; Q0VCV8	ENSBTAG00000013033	-0.149455849	-0.197981557	0.048525708
P63171	DYNLT1	bta:282380; P63171; Q15763; Q32PD4	ENSBTAG00000004472	0.063994854	0.16749943	-0.103504576
F1MSV8	MRPL21	F1MSV8	ENSBTAG00000004348	0.029747343	-0.042704451	0.072451794
A0AAA9TGR5	CENPK	A0AAA9TGR5	ENSBTAG00000044175	-0.344334508	-0.36055486	0.016220352
A7MBI3	L2HGDH	A7MBI3; bta:514230	ENSBTAG00000005550	-0.030088578	0.126771057	-0.156859635
A0AAA9T184	LMBRD2	A0AAA9T184	ENSBTAG00000020142	-0.119632847	0.015135709	-0.134768556
F1N6W1	PTPN9	F1N6W1	ENSBTAG00000016984	-0.344557661	-0.115714835	-0.228842826
A0A3Q1NNI1	COA4	A0A3Q1NNI1	ENSBTAG00000054199	0.260680906	0.256257719	0.004423187
A0AAA9SK75	FAM107B	A0AAA9SK75	ENSBTAG00000010023	0.201357509	0.157395166	0.043962343
F6RWY3	ALG2	F6RWY3	ENSBTAG00000002454	-0.094366571	0.023014566	-0.117381137
Q0VC80	YRDC	bta:512062; Q0VC80	ENSBTAG00000006510	0.058214614	0.169547777	-0.111333163
A0A3Q1M2Y0	EVI5L	A0A3Q1M2Y0	ENSBTAG00000004294	0.092757141	0.279610581	-0.18685344
Q0VCV5	LNX2	bta:526424; Q0VCV5	ENSBTAG00000015614	0.187877046	0	0.187877046
A0AAA9T2M2	FOXJ2	A0AAA9T2M2	ENSBTAG00000019742	-0.273595503	-0.128807158	-0.144788345
A0AAA9TSC6	GPATCH11	A0AAA9TSC6	ENSBTAG00000003861	0.29616815	0.034557222	0.261610928
E1BBK3	FLYWCH2	A0A8J8XTP4; E1BBK3; M5FI44	ENSBTAG00000038115	-0.037109058	0.440705565	-0.477814623
A0A3Q1N1U9	IFT43	A0A3Q1N1U9	ENSBTAG00000012005	-0.087643009	0.05375368	-0.141396689
E1BDF2	SHARPIN	bta:512499; E1BDF2		0.13073808	0.175253074	-0.044514994
A0A3Q1LPS7	C7H1orf35	A0A3Q1LPS7	ENSBTAG00000020720	0.44236513	0.054147229	0.388217902
A0AAF7A363	ALKBH3	A0AAF7A363	ENSBTAG00000009518	-0.58773159	0.13963964	-0.72737123
E1BM39	CUL9	bta:540077; E1BM39	ENSBTAG00000019908	-0.088697346	0.086217958	-0.174915304
F1MEK1	METTL17	F1MEK1	ENSBTAG00000006613	-0.17707359	-0.244834517	0.067760927
Q2HJ90	LRRC42	bta:534728; Q2HJ90	ENSBTAG00000019198	-0.415507662	-0.017023148	-0.398484514
Q0VCH6	SLC25A40	bta:613948; Q0VCH6	ENSBTAG00000054987	-0.049679603	0.070787917	-0.12046752
F1MGY9	AGA	F1MGY9	ENSBTAG00000017085	-0.341603458	-0.148420162	-0.193183297
A0A3Q1M6H8	NCAPH2	A0A3Q1M6H8	ENSBTAG00000012607	-0.509905512	-0.29056759	-0.219337922
A0AAA9S6S8	TMOD2	A0AAA9S6S8	ENSBTAG00000014011	-0.344075164	-0.094051624	-0.25002354
Q66WT7	CAV2	A4D7R4; A7YWJ5; bta:493642; Q66WT7	ENSBTAG00000062162	0.400248696	0.338690935	0.061557761
A0AAF6ZG78	TMBIM6	A0AAF6ZG78	ENSBTAG00000018588	0.150866666	-0.02097809	0.171844756
P01035	CST3	bta:281102; P01035; Q54A26	ENSBTAG00000000598	0.206880667	0.36041921	-0.153538544
F1MWY9	ECI2	bta:505355; F1MWY9	ENSBTAG00000015178	-0.082064537	0.071226102	-0.153290639
A4IFB2	KLF5	A4IFB2; bta:535702	ENSBTAG00000002129	-0.061579262	0.106915204	-0.168494466
A5PJR6	TMEM41B	A5PJR6; bta:616372; F1N5X2	ENSBTAG00000001607	-0.07287888	-0.173482821	0.100603941
Q3SZC2	PSMB9	bta:510593; Q3SZC2	ENSBTAG00000008954	0.02666795	0.001793282	0.024874669
Q5E9H3	FES	bta:507304; Q5E9H3	ENSBTAG00000002941	-0.27064759	-0.101570983	-0.169076607
A0AAA9SB48	NCKIPSD	A0AAA9SB48	ENSBTAG00000021850	-0.076826626	0.040367473	-0.117194099
F6RC73	LYPLAL1	F6RC73	ENSBTAG00000015534	-0.342436274	-0.175644162	-0.166792112
Q3MHX9	NUDT16L1	A0A8J8Y2A7; bta:518085; F1MEX5; M5FK06; Q3MHX9		-0.492396382	-0.336486191	-0.155910191
A2VE45	IFT70A	A2VE45; bta:510160		-0.135831803	0.11772716	-0.253558963
E1BFW9	OTUD5	E1BFW9	ENSBTAG00000004600	-0.15631457	-0.183233695	0.026919125
A0AAA9SVP3	PRKX	A0AAA9SVP3	ENSBTAG00000014367	-0.928952358	-0.573027953	-0.355924406
A0AAA9RYC9	MAP2K7	A0AAA9RYC9	ENSBTAG00000010639	0.273356877	0.195504732	0.077852145
A1A4M6	STARD5	A1A4M6; bta:512369	ENSBTAG00000010964	-0.16515197	-0.064130337	-0.101021633
E1BL80	THAP12	E1BL80	ENSBTAG00000031609	0.071371695	0.08735248	-0.015980785
A0A3Q1LRW7	JADE2	A0A3Q1LRW7	ENSBTAG00000021687	-0.268456469	-0.326206976	0.057750508
Q29RJ1	NUDT2	bta:768044; Q29RJ1		-0.136680146	0.004112198	-0.140792344
Q08D88	NFIL3	bta:506097; Q08D88	ENSBTAG00000065834	0.199727542	-0.017962479	0.217690021
Q2YDK4	HMGN4	bta:614918; Q2YDK4		0.050829944	0.165977817	-0.115147873
Q17QE0	FAM241A	bta:767842; Q17QE0		0.577210225	0.649966567	-0.072756342
A0AAA9TFG3	SOD3	A0AAA9TFG3	ENSBTAG00000013980	-0.427913863	-0.250126744	-0.177787119
A1L539	GPC6	A1L539; bta:536153	ENSBTAG00000040347	-0.284832996	-0.385359872	0.100526876
Q08DL6	PEX7	bta:533077; Q08DL6	ENSBTAG00000016791	-0.0480409	-0.159723689	0.111682789
A0AAA9RZ65	SLC31A1	A0AAA9RZ65	ENSBTAG00000021678	0.180403519	0.090328175	0.090075344
F1MEL4	COIL	F1MEL4	ENSBTAG00000017275	-0.017296823	0.076109157	-0.093405981
F1MIU1	LOC510536	F1MIU1	ENSBTAG00000023846	0.462716594	0.150989812	0.311726782
A0AAA9TF18	CLMP	A0AAA9TF18	ENSBTAG00000020046	-0.323788437	-0.068531382	-0.255257055
A0AAA9TLH2	SZT2	A0AAA9TLH2; bta:533805	ENSBTAG00000005580	-0.196756494	0.276383959	-0.473140454
Q08DZ3	ELMOD2	bta:515278; Q08DZ3	ENSBTAG00000014007	0.052981292	-0.040128113	0.093109404
G3MZR4	LOC523702	G3MZR4	ENSBTAG00000045999	-0.164459907	-0.002828815	-0.161631092
A0A3Q1MBA7	PHC3	A0A3Q1MBA7	ENSBTAG00000013938	0.061979669	0.097062128	-0.035082459
A0AAA9TZF9	PIP5K1C	A0AAA9TZF9	ENSBTAG00000000710	0.230082379	0.208782977	0.021299402
A0A3Q1N4A9	CFAP410	A0A3Q1N4A9	ENSBTAG00000019589	-0.088459098	-0.029721807	-0.058737291
F1MM05	MAU2	F1MM05	ENSBTAG00000006124	0.156327398	-0.077852145	0.234179543
A0AAA9RX20	BBS1	A0AAA9RX20	ENSBTAG00000020147	-0.043284098	0.082856932	-0.12614103
A0AAA9RWF0	TPCN2	A0AAA9RWF0	ENSBTAG00000015183	-0.736237696	-0.350606464	-0.385631232
A0A3Q1NEK5	SKA1	A0A3Q1NEK5	ENSBTAG00000018216	-0.114998791	-0.267347399	0.152348608
F1N0F2	SIAE	F1N0F2	ENSBTAG00000009412	0.107241963	0.201766468	-0.094524505
A6QP68	PUSL1	A6QP68		-0.297618642	-0.226359959	-0.071258683
A0AAA9SW16	TUSC3	A0AAA9SW16	ENSBTAG00000009191	0.315997172	0.537327929	-0.221330757
F1MGL9	LIMK2	F1MGL9	ENSBTAG00000010662	-0.54703887	-0.287562284	-0.259476586
A0A3Q1LQC9	GCFC2	A0A3Q1LQC9	ENSBTAG00000008048	-0.1930615	0.167656358	-0.360717858
Q0IIE8	DNAJB14	bta:538849; Q0IIE8	ENSBTAG00000004426	0.060190678	0.110082483	-0.049891805
F1MLY5	MLLT6	F1MLY5	ENSBTAG00000011532	-0.077382531	0.086641869	-0.1640244
Q32KY6	DCTPP1	bta:614103; Q32KY6		0.442962845	0.348283992	0.094678853
Q0VCL5	HMG20A	bta:513719; Q0VCL5	ENSBTAG00000020441	0.292148433	0.175086707	0.117061726
Q0P5J9	RPRD1A	bta:504355; Q0P5J9	ENSBTAG00000005497	-0.096278289	0.226040198	-0.322318487
E1BB33	HJURP	bta:617456; E1BB33	ENSBTAG00000024726	-0.037358251	-0.102383411	0.06502516
A0AAA9T0M0	CDKN2A	A0AAA9T0M0	ENSBTAG00000034220	-0.317209546	-0.15713233	-0.160077215
Q3SX29	SFRP2	bta:510821; Q3SX29	ENSBTAG00000018563	0.039596174	0.012490944	0.02710523
E1BII1	CTU1	bta:100297056; E1BII1	ENSBTAG00000023365	-0.106611191	0.015724352	-0.122335543
E1BF06	TNFSF15	bta:514239; E1BF06	ENSBTAG00000018069	-0.073709686	-0.072546691	-0.001162995
A0AAA9TQY3	SPINDOC	A0AAA9TQY3	ENSBTAG00000018022	0.276923274	0.332010834	-0.05508756
F1MSM3	NOTCH1	F1MSM3	ENSBTAG00000022799	0.064130337	0.095839197	-0.03170886
E1BF91	ATAT1	E1BF91	ENSBTAG00000006941	0.288967896	0.08763709	0.201330806
A0A3Q1MR63	CEP78	A0A3Q1MR63	ENSBTAG00000020752	0.317936385	0.149855084	0.168081301
A0AAA9SN40	HOOK2	A0AAA9SN40	ENSBTAG00000008654	-0.191640066	0.078743115	-0.270383181
A0AAA9TEI3	TRIM2	A0AAA9TEI3	ENSBTAG00000008816	-0.367917209	-0.104264814	-0.263652395
A0AAF6YTR8	WDR74	A0AAF6YTR8	ENSBTAG00000009491	-0.396433531	-0.210566986	-0.185866545
Q2HJF5	DPH6	bta:767944; Q2HJF5	ENSBTAG00000001364	-0.515317406	-0.48584355	-0.029473855

Accession	Gene_Symbol	Gene_ID	Ensembl_Gene ID	log2(ratio(OV24H/OV0H))	log2(ratio(OV24R/OV0H))	log2(ratio(OV24R/OV24H))
A0AAA9RX26	LBHD1	A0AAA9RX26	ENSBTAG00000010465	-0.417259423	-0.001332744	-0.41592668
F1N6T3	LSM7	F1N6T3	ENSBTAG00000004521	0.694007859	0.423659714	0.270348145
E1BPY4	NCOA2	E1BPY4	ENSBTAG00000020312	0.152567639	0.058102955	0.094464684
A0A3Q1M0N2	RNASEH1	A0A3Q1M0N2	ENSBTAG00000016235	-0.016731495	0.010547165	-0.02727866
A0AAA9TS89	ATRIP	A0AAA9TS89	ENSBTAG00000008399	-0.311349165	-0.131939915	-0.179409251
A0A3Q1M0Y3	HERPUD1	A0A3Q1M0Y3	ENSBTAG00000016896	-0.08059929	0.219291645	-0.299890935
A0AAA9SV16	TOP3A	A0AAA9SV16	ENSBTAG00000017087	-0.103792486	-0.069881426	-0.03391106
A0A452DJ80	MYCBP	A0A452DJ80	ENSBTAG00000030337	0.241585987	0.568589531	-0.327003544
F1MPF7	PHIP	F1MPF7	ENSBTAG00000024688	-0.175679676	-0.272252936	0.09657326
F1MLD7	EFR3A	F1MLD7	ENSBTAG00000047834	0.211655371	0.380941588	-0.169286217
A0AAA9TY81	CACUL1	A0AAA9TY81	ENSBTAG00000022808	-0.163937642	-0.07169273	-0.092244911
Q3SYX6	SUPT4H1	bta:616425; Q3SYX6	ENSBTAG00000013664	-0.06853908	-0.114053739	0.045514659
E1BCP9	GLB1L	bta:532551; E1BCP9	ENSBTAG00000020611	-0.207324973	0.387440703	-0.594765676
A0A3Q1MRT6	FXYD5	A0A3Q1MRT6; bta:505584	ENSBTAG00000013441	-0.087462841	0.185555653	-0.273018494
Q29S14	TVP23B	bta:513550; Q29S14	ENSBTAG00000060680	0.025535092	0.038971232	-0.013436139
Q0VCN2	WDR83	bta:780845; Q0VCN2	ENSBTAG00000006245	0.168175219	0.19133294	-0.023157721
A0AAA9S8U8	COMMD8	A0AAA9S8U8; bta:507292	ENSBTAG00000011348	0.275486147	[illegible]	-2.773139207
F6Q952	KBTBD2	F6Q952	ENSBTAG00000003115	-0.489983594	-0.159338282	-0.330645312
A0A3Q1MUL6	IREB2	A0A3Q1MUL6	ENSBTAG00000002504	-0.085891953	-0.055313457	-0.030578496
E1BNR7	VANGL1	E1BNR7	ENSBTAG00000005768	-0.064060872	0.084583209	-0.148644081
F1MEL7	RFLNB	F1MEL7	ENSBTAG00000008606	0.226048518	-0.032392359	0.258440877
A5D7V4	TRMO	A5D7V4; bta:507596; F1MKS2	ENSBTAG00000019238	0.383772673	0.037572307	0.346200366
A0AAA9TQW6	KMT2C	A0AAA9TQW6	ENSBTAG00000024199	-0.222870531	-0.152640608	-0.070229923
F1MX94	PTS	A0A3Q1LXL4; A0A8J8XH99; bta:506860; F1MX94	ENSBTAG00000062028	0.337666133	0.116383392	0.221282741
G5E6N6	ZCCHC9	G5E6N6	ENSBTAG00000005265	0.149539053	0.10750126	0.042037793
Q08E13	RNF10	A6H708; bta:520757; Q08E13		-0.315973372	-0.094524505	-0.221448867
Q2T9Q1	TBC1D20	bta:504543; Q2T9Q1	ENSBTAG00000013330	0.086435061	0.204819792	-0.118384731
P00516	PRKG1	bta:282004; P00516; P21136		-0.618182036	0.289403667	-0.907585703
Q5E9T1	GDPGP1	bta:522909; Q0V8C7; Q58D74; Q5E9T1	ENSBTAG00000005174	0.001447762	0.115477217	-0.114029455
A0A3Q1LXJ7	TRAF1	A0A3Q1LXJ7	ENSBTAG00000003012	-0.367240511	2.058110112	-2.425350623
A0AAA9RSX8	ATP11C	A0AAA9RSX8	ENSBTAG00000016484	0.068817375	0.070246494	-0.001429119
A0AAF6Z3H7	CTSS	A0AAF6Z3H7	ENSBTAG00000017135	-0.264294952	-0.128397354	-0.135897598
A7E309	MDFIC	A7E309; bta:520530	ENSBTAG00000021543	0.074458992	-0.093109404	0.167568397
A0AAA9TQG4	ABCD4	A0AAA9TQG4	ENSBTAG00000014633	-0.026327282	0.002606496	-0.028933778
Q5EA32	TRPV2	bta:507664; Q5EA32	ENSBTAG00000003014	-0.23357222	-0.106058797	-0.127513423
A0A3Q1LVR3	PRPF18	A0A3Q1LVR3	ENSBTAG00000017244	-0.236460383	-0.289506617	0.053046234
P0DW91	ZFTRAF1	P0DW91; Q3SX19; Q7YR89; Q7YR91; Q7YRA6; Q8MK42	ENSBTAG00000035254	0.315014208	0.373861693	-0.058847485
Q32LD4	TFB2M	bta:516874; Q32LD4	ENSBTAG00000000072	-0.002990043	-0.061010996	0.058020953
A0A3Q1M5Y6	UBE2Q2	A0A3Q1M5Y6	ENSBTAG00000004024	0.088515366	0.138154266	-0.0496389
A0JNG0	TMEM168	A0JNG0; bta:541075	ENSBTAG00000021967	0.126029653	0.101131134	0.024898519
Q5DPW9	CST6	bta:503685; G3N3P6; Q5DPW9	ENSBTAG00000046587	0.522337166	0.844495889	-0.322158723
E1BCG5	NDST1	bta:514172; E1BCG5	ENSBTAG00000001692	-0.226275856	-0.023458973	-0.202816883
A0AAA9T296	ANKRD46	A0AAA9T296	ENSBTAG00000001254	0.199974955	0.057622032	0.142352923
A0AAA9SJA1	SIRT6	A0AAA9SJA1	ENSBTAG00000019909	-0.436022524	-0.211367052	-0.224655473
A0AAA9S992	MED18	A0AAA9S992	ENSBTAG00000002678	0.594270243	0.388978622	0.205291621
E1B9D0	CHPF2	E1B9D0	ENSBTAG00000014371	-0.959423339	-0.496047943	-0.463375396
Q08DQ8	SLC26A6	bta:514514; E1BFJ3; Q08DQ8	ENSBTAG00000038381	-0.288761231	-0.240398209	-0.048363022
A0AAA9SD21	CSKMT	A0AAA9SD21	ENSBTAG00000022293	-0.018262206	-0.05819174	0.039929533
A0A3Q1LGF4	SCEL	A0A3Q1LGF4	ENSBTAG00000002821	-0.132159949	0.057850069	-0.19001018
Q3ZC66	CRIPT	bta:617723; Q3ZC66	ENSBTAG00000015723	0.465856382	-0.144978644	0.610835026
A0AAA9TPD8	MPV17	A0AAA9TPD8	ENSBTAG00000018269	-0.181072834	-0.290062837	0.108990003
A0AAA9TUT1	PGAP1	A0AAA9TUT1; bta:504677	ENSBTAG00000020416	0.101550795	0.317449717	-0.215898923
A0AAA9RUZ5	PROCR	A0AAA9RUZ5	ENSBTAG00000008291	0.085116614	0.077582941	0.007533673
G3X7J5	TOR4A	bta:618444; G3X7J5	ENSBTAG00000023787	-0.139850274	-0.028667886	-0.111182388
A0AAF6ZED3	NFYB	A0AAF6ZED3	ENSBTAG00000030744	0.560504401	0.276868372	0.283636029
A7YWK3	KRT73	A7YWK3; bta:531981	ENSBTAG00000037638	-0.150969274	0.92256618	-1.073535454
A0A3Q1M6Z9	MMADHC	A0A3Q1M6Z9	ENSBTAG00000015962	-0.164084123	-0.138088914	-0.025995209
Q17QW3	RDH14	bta:505949; Q17QW3	ENSBTAG00000023928	0.219845739	0.375540057	-0.155694317
P82926	MRPS33	bta:523435; P82926; Q32LL3	ENSBTAG00000005285	-0.139162748	-0.182986294	0.043823546
Q5E9C2	DAD1	bta:614538; Q3T175; Q53ZY7; Q5E9C2		0.351423262	0.273895398	0.077527863
A0AAA9SAX7	TIMM22	A0AAA9SAX7	ENSBTAG00000008423	-0.152577758	-0.104530583	-0.048047174
A0AAF6YLK6	LMAN2L	A0AAF6YLK6	ENSBTAG00000003975	-0.044438234	-0.044438234	0
F6RMD1	TMEM9B	F6RMD1	ENSBTAG00000008310	-0.166326359	-0.163095238	-0.003231121
A0AAA9TZ13	MYRF	A0AAA9TZ13	ENSBTAG00000006990	0.015193674	0.138891398	-0.123697724
A0AAA9S2K4	ABRACL	A0AAA9S2K4	ENSBTAG00000001446	0.503020887	-0.063325935	0.566346823
A7YWG4	GGH	A7YWG4; bta:525303	ENSBTAG00000007534	0.208056804	0.472828948	-0.264772144
Q5EA56	TRAF7	A0A8J8XK14; bta:511692; M5FHP8; Q5EA56	ENSBTAG00000019073	-0.010908899	-0.096918143	0.086009244
A0A3Q1M7N7	GULP1	A0A3Q1M7N7	ENSBTAG00000007141	-0.005791638	-0.024482685	0.018691047
A0AAA9RWT3	PTTG1	A0AAA9RWT3	ENSBTAG00000012184	0.092794096	-0.150509234	0.243303329
A0AAA9RWZ2	PROSER2	A0AAA9RWZ2	ENSBTAG00000039571	0.145655987	-0.173412454	0.319068441
A0A3Q1LXQ0				0.642958052	0.092854308	0.550103744
Q08E12	GINS3	bta:538943; Q08E12	ENSBTAG00000009363	0.041244488	-0.081277644	0.122522132
A0A452DJ63	KNOP1	A0A452DJ63	ENSBTAG00000019593	0.381913539	0.220282446	0.161631092
A0AAA9T8Z6	ATXN7	A0AAA9T8Z6	ENSBTAG00000011287	0.324218867	0.296486575	0.027732292
A7MBJ3	EDEM2	A7MBJ3; bta:513253		-0.024335192	0.567040593	-0.591375785
A0AAA9TJH2	RAD51AP1	A0AAA9TJH2	ENSBTAG00000040065	-0.095495997	-0.208056804	0.112560806
A5D7S5	SLC25A35	A5D7S5; G1K1W1		0.303540726	0.83588062	-0.532339894
E1BHC5	OSGEPL1	bta:514738; E1BHC5	ENSBTAG00000012557	0.121950532	0.206450877	-0.084500345
A0AAA9S9Y1	POLR1H	A0AAA9S9Y1	ENSBTAG00000031792	0.153314039	-0.004378441	0.157692479
E1BC22	CIAO2B	bta:614585; E1BC22	ENSBTAG00000013931	0.591248764	0.160796212	0.430452552
A5PJA1	AK6	A5PJA1; bta:102216273		0.678312977	0.105950513	0.572362464
F1N5T0	CUTA	F1N5T0	ENSBTAG00000001635	0.468688544	0.298439742	0.170248802
A0A3Q1MIG0	SH3BP2	A0A3Q1MIG0	ENSBTAG00000013996	-0.15299381	-0.062452818	-0.090540991
A0AAA9SWX9	ZNF333	A0AAA9SWX9; bta:789635	ENSBTAG00000062579	0.175454284	0.26383836	-0.088384076
Q6J1J1	BIRC5	bta:414925; Q32LI0; Q6J1J1	ENSBTAG00000013573	-0.089399085	-0.13626569	0.046866605
A4D7R9	ST7	A4D7R9; Q9BDI1	ENSBTAG00000010756	-0.095252459	-0.179734525	0.084482065
A4IFN9	SMG5	A4IFN9; bta:513598	ENSBTAG00000009435	-0.257347373	-0.314178595	0.056831223
E1BN06	RASSF5	E1BN06	ENSBTAG00000010427	0.027789528	0.01627554	0.011513988
G3MYU1	BAP1	G3MYU1	ENSBTAG00000045817	0.001555467	-0.009368183	0.010923649
B0JYL2	NPR3	B0JYL2; E1BJP0	ENSBTAG00000007665	0.061868724	-0.154033629	0.215902353
E1BIA1	PPFIA3	E1BIA1	ENSBTAG00000002844	0.029608587	0.116209205	-0.086600618
F6QT14	ELOVL1	F6QT14	ENSBTAG00000055871	0.067488019	-0.091684412	0.159172431
A0A3Q1MUK5	AGAP3	A0A3Q1MUK5	ENSBTAG00000048379	0.044822198	0.186746085	-0.141923887
A0AAF6YL67	TMEM128	A0AAF6YL67	ENSBTAG00000003652	-0.535477663	-0.605971236	0.070493573
A0AAA9TXN7	PARP8	A0AAA9TXN7	ENSBTAG00000004066	-1.027093661	0.260235772	-1.287329433
A0A3Q1LNW3	ICE2	A0A3Q1LNW3	ENSBTAG00000005328	0.102020937	0.053410993	0.048609944
G3XCF6	NFATC1	G3XCF6	ENSBTAG00000000656	-0.067929854	0.223615044	-0.291544898
A1A4J6	ATP9B	A1A4J6; bta:510301	ENSBTAG00000001224	-0.327996931	-0.11189288	-0.216104051
A7MBC3	WEE1	A7MBC3	ENSBTAG00000005278	0.247762076	0.208858598	0.038903479
Q8HZT6	RDH10	bta:282852; Q8HZT6	ENSBTAG00000020143	0.033082355	-0.301850274	0.334932629
Q2M2S2	SMDT1	bta:506149; Q2M2S2		-0.467084247	-0.436015589	-0.031068658
Q32PA1	CD59	bta:505574; Q32PA1	ENSBTAG00000002302	-0.026588375	0.126053503	-0.152641878
Q08DP7	PPP2R5B	bta:505915; Q08DP7	ENSBTAG00000007061	0.066563601	0.209576105	-0.143012503
A0AAF6DM01	CCDC32	A0AAF6DM01	ENSBTAG00000025593	0.436314159	-0.257439368	0.693753527
F1MGT3	MTSS2	F1MGT3	ENSBTAG00000009358	-0.20894689	0.176753064	-0.385699954
A0AAA9T2X7	BCL7B	A0AAA9T2X7	ENSBTAG00000020544	0.082777676	0.105392999	-0.022615323
E1B806	SAP30	bta:781150; E1B806	ENSBTAG00000015578	-0.094243801	-0.04077453	-0.053469271
A0AAF6YRH9	ARFRP1	A0AAF6YRH9	ENSBTAG00000007497	0.169925001	0.173798011	-0.003873009
A0AAA9U271	POLL	A0AAA9U271	ENSBTAG00000003576	-0.083280542	0.392738832	-0.476019374
A0AAF6YYX7	GET1	A0AAF6YYX7	ENSBTAG00000013629	0.231930551	0.269012808	-0.037082257
F1N1M2	GATA6	F1N1M2	ENSBTAG00000005734	-0.375247469	-0.37054558	-0.004701889
E1BDS4	ABHD14A	E1BDS4	ENSBTAG00000053854	-0.19386995	0.091322785	-0.285192735
F1MSC2	CFAP97	F1MSC2	ENSBTAG00000013211	0.153199854	0.127857006	0.025342848
E1BPN5	SSH2	E1BPN5	ENSBTAG00000011011	0.085456776	0.062524254	0.022932522
Q58DB8	MDS018	bta:614957; Q58DB8		-0.349084426	1.286851248	-1.635935674
Q3SZA2	FMC1	bta:767923; Q3SZA2		-0.08778065	-0.138647965	0.050867314
F1MEB4	SPG11	F1MEB4	ENSBTAG00000000362	-0.136419198	0.072711885	-0.209131084
F1N148	MRPL54	F1N148	ENSBTAG00000008396	-0.12472648	0.00882385	-0.13355033
A0AAA9TRL1	PRXL2A	A0AAA9TRL1	ENSBTAG00000021416	0.355121973	0.52168736	-0.166565388
A0AAA9SS13	BAZ2A	A0AAA9SS13	ENSBTAG00000017840	-0.164208429	-0.075868954	-0.088339475
Q3SWY9	RAB28	bta:536021; Q3SWY9	ENSBTAG00000003218	-0.303802258	0.02727293	-0.331075188
A0A3Q1MR14	LGALSL	A0A3Q1MR14	ENSBTAG00000055296	-0.062800787	0.094845885	-0.157646672
F1N5Q7	RFXAP	F1N5Q7	ENSBTAG00000005351	0.061986887	0.129162368	-0.067175482
A0AAA9RYL0	ROR1	A0AAA9RYL0	ENSBTAG00000012312	0.074926002	-0.010003869	0.084929871
A0AAA9RSH3	IMPACT	A0AAA9RSH3	ENSBTAG00000003035	-0.239214632	-0.174662244	-0.064552388
A6QLM0	PEDS1	A6QLM0; bta:507694	ENSBTAG00000010135	-0.175824913	0.098817736	-0.27464265
Q05716	IGFBP4	A5D7U8; bta:282262; Q05716	ENSBTAG00000008611	-0.065326248	0.141255659	-0.206581906
Q1RMJ7	TRNAU1AP	bta:532582; Q1RMJ7	ENSBTAG00000010393	0.075630263	0.066761208	0.008869055
A0A3Q1NG02	MCRS1	A0A3Q1NG02	ENSBTAG00000019281	0.05189073	0.088239621	-0.036348891
A0AAA9U068	PEX3	A0AAA9U068	ENSBTAG00000001747	-0.236352363	-0.254595325	0.018242962
A6QQQ3	PRELP	A6QQQ3; F1MX63	ENSBTAG00000017834	-0.260715581	-0.263808176	0.003092595
Q32LM8	NRM	bta:511167; Q32LM8	ENSBTAG00000006966	-0.317402496	-0.368316768	0.050914272
E1BGW5	SIN3B	E1BGW5	ENSBTAG00000009330	-0.567295463	-0.426551487	-0.140743976
F1MER1	CDYL2	F1MER1	ENSBTAG00000017112	-0.053345154	-0.104710787	0.051365632
A0AAA9SA86	TAF8	A0AAA9SA86	ENSBTAG00000011339	-0.084843501	0.101232551	-0.186076052
Q2YDE7	ZBTB8OS	bta:613795; Q2YDE7	ENSBTAG00000027159	0.04355167	-0.024204723	0.067756393
A0A3Q1M5I0	SCAI	A0A3Q1M5I0	ENSBTAG00000015553	0.001513052	0.160305311	-0.158792259
A0AAA9TWI8	CENPH	A0AAA9TWI8	ENSBTAG00000014246	-0.56193706	-0.570982199	0.00904514
F1N3D2	ZNF280C	F1N3D2	ENSBTAG00000019885	-0.098895756	0.285019226	-0.383914982
A0AAA9T0W6	NECTIN3	A0AAA9T0W6	ENSBTAG00000013769	-0.077560983	-0.130235523	0.05267454
A0AAA9RVN1	APLF	A0AAA9RVN1	ENSBTAG00000018401	0.181922241	0.196466789	-0.014544548
A0AAA9TTS0	PINX1	A0AAA9TTS0	ENSBTAG00000000500	0.376450262	0.299664882	0.07678538
A0AAF6Z712	GINS1	A0AAF6Z712	ENSBTAG00000019984	-0.159822598	0.05057229	-0.210394888
Q3MHP8	BET1L	bta:509758; Q3MHP8	ENSBTAG00000001744	-0.129152154	-0.199541482	0.070389328
E1BGA4	ASH1L	E1BGA4	ENSBTAG00000003954	-0.445332511	-0.3124114	-0.132921111
Q58CQ1	IRF9	bta:509855; Q58CQ1	ENSBTAG00000005816	-0.192881721	0.125254795	-0.318136516
A0A3Q1MS71	LDB1	A0A3Q1MS71	ENSBTAG00000005780	-0.161625678	-0.086384306	-0.075241372
A7YWM8	NANP	A7YWM8; bta:516539	ENSBTAG00000000686	0.09010389	0.334579336	-0.244475446
Q08DS9	DHX40	bta:533484; Q08DS9		0.303653036	0.364996817	-0.06134378
E9LZ03				0.174925683	0.151016539	0.023909144
A0A3Q1N9J7	CLDND1	A0A3Q1N9J7	ENSBTAG00000020996	-0.232696574	-0.184685079	-0.048011494
A0AAA9RUN2	PHACTR2	A0AAA9RUN2	ENSBTAG00000016498	-0.063362061	-0.027307346	-0.036054715
A0AAA9ST48	KDM3A	A0AAA9ST48	ENSBTAG00000013580	-0.074000581	0.01643689	-0.090437471
A0AAA9RU64	UBE2B	A0AAA9RU64	ENSBTAG00000010982	-0.162105497	-0.19523609	0.033130593
A0AAA9SV46	INO80C	A0AAA9SV46	ENSBTAG00000012426	-0.272317647	0.308122295	-0.580439942
A5PK28	LHFPL2	A5PK28; bta:616131	ENSBTAG00000007374	-0.109735723	-0.280611179	0.170875456
F1MEA1	TMEM132A	F1MEA1	ENSBTAG00000018364	0.009974228	0.099440349	-0.089466121
A0AAA9TR91	TMEM222	A0AAA9TR91	ENSBTAG00000010249	0.005973901	-0.00900749	0.014981392
E1B7K6	DGKE	bta:538147; E1B7K6	ENSBTAG00000004449	0.18884751	0.053604744	0.135242767
A0A3Q1MXI5	DAPK1	A0A3Q1MXI5	ENSBTAG00000000738	-0.045183309	-0.133421668	0.088238359
E1BE31	JMY	E1BE31	ENSBTAG00000025856	0.146898072	0.207504837	-0.060606765
A6QPS5	CENPI	A6QPS5; bta:512877; F1MZ56	ENSBTAG00000021254	-0.042552616	-0.053389813	0.010837197
A0AAF6YZ47	CENPS	A0AAF6YZ47	ENSBTAG00000013531	0.272483469	0.130119976	0.142363494
A5PJW2	TMEM223	A5PJW2; bta:616632		-0.203572533	-0.289363115	0.085790582
A0A3Q1MFB7	ZNF706	A0A3Q1MFB7	ENSBTAG00000008054	0.296329781	-0.170478288	0.466808069
A2VDQ0	IFT81	A2VDQ0; bta:519602	ENSBTAG00000020584	-0.310812782	-0.041279739	-0.269533043
A0A3Q1LTR5	NR2C2	A0A3Q1LTR5	ENSBTAG00000013716	-0.097555309	0.101753499	-0.199308808
A5PJJ2	ALG12	A5PJJ2; bta:100125314; G3MWZ3	ENSBTAG00000046173	-0.605674005	-0.205535383	-0.400138623
Q3T0H0	LCMT1	bta:618021; Q3T0H0	ENSBTAG00000013169	-0.084628695	0.098390223	-0.183018918
Q3SZN3	OMA1	bta:506223; F1N0Z4; Q3SZN3	ENSBTAG00000017326	0.276997404	0.350044048	-0.073046643
Q5E9A7	KLHDC2	bta:535436; Q5E9A7	ENSBTAG00000004933	-0.27929946	-0.200987058	-0.078312402
F1MBU9	PIK3C2B	F1MBU9	ENSBTAG00000024633	0.004758757	-0.009564864	0.014323621
Q08DQ3	CDK2AP1	bta:615387; Q08DQ3	ENSBTAG00000020504	-0.556463894	-0.53371626	-0.022747634
A0A3Q1LF69	CLSPN	A0A3Q1LF69	ENSBTAG00000002826	-0.145382817	-0.18950537	0.044122553
Q5E9L5	PRMT6	bta:540228; Q5E9L5	ENSBTAG00000039951	0.149882758	0.080534712	0.069348047
E1BHM1	MRGBP	bta:616297; E1BHM1	ENSBTAG00000031716	0.187156617	0.061765701	0.125390917
E1B7T3	NUDT15	E1B7T3	ENSBTAG00000019501	0.502278745	0.417389848	0.084888898
F1MK42	RHOQ	bta:100139988; F1MK42	ENSBTAG00000013749	0.123002316	0.177632041	-0.054629724
E1BK04	NCOA1	E1BK04	ENSBTAG00000015007	-0.059278723	-0.019936223	-0.0393425
A0AAA9TY18	IFT80	A0AAA9TY18	ENSBTAG00000031572	-0.172639386	-0.265748791	0.093109404
A0A3Q1LVT3	TRIM4	A0A3Q1LVT3	ENSBTAG00000050135	0.038438798	0.177962321	-0.139523523
F1MFA9	MCPH1	F1MFA9	ENSBTAG00000011032	0.169925001	0.114257179	0.055667822
Q2TBV5	GTF2H2	bta:511829; Q2TBV5		-0.12043001	0.071578444	-0.192008454
A3KN46	LETMD1	A3KN46; bta:514595		-0.151669869	0.181542303	-0.333212172
A0AAA9TAP6	CDA	A0AAA9TAP6; bta:616377	ENSBTAG00000068005	-0.182692298	-0.197191867	0.01449957
A0AAA9U0Q6	SHB	A0AAA9U0Q6	ENSBTAG00000014922	0.093963828	0.037790244	0.056173584
Q3ZBP7	TAF11	bta:507047; Q3ZBP7	ENSBTAG00000016887	0.013864768	0.123077867	-0.109213099
F1MYW5	MPND	F1MYW5	ENSBTAG00000006005	0.188389588	0.405145474	-0.216755886
Q05B92	TFE3	bta:520800; Q05B92		0.062706742	0.115267417	-0.052560674

Accession	Gene_Symbol	Gene_ID	Ensembl_Gene ID	log2(ratio(OV24H/OV0H))	log2(ratio(OV24R/OV0H))	log2(ratio(OV24H/OV24R))
A0A3Q1N625	GPR89A	A0A3Q1N625	ENSBTAG00000005809	-0.098924233	0.159262489	-0.258186721
F1MFT2	R3HCC1	bta:614496; F1MFT2	ENSBTAG00000025830	0.047191083	0.303330876	-0.256139793
A0AAA9TZE6	TMEM126B	A0AAA9TZE6; bta:504229	ENSBTAG00000020596	0.08238969	-0.279828426	0.362218117
Q5EA28	CXXC1	bta:511446; Q5EA28	ENSBTAG00000021884	-0.257674891	-0.183589845	-0.074085046
F1N1C8	BRAT1	F1N1C8	ENSBTAG00000014502	-0.083775492	-0.083775492	0
F1MV64	INSR	bta:408017; F1MV64	ENSBTAG00000012687	-0.014728337	0.302886765	-0.317615102
Q2HJ67	HOXA5	bta:768039; Q2HJ67		-0.147153694	-0.359294947	0.212141254
G3MZ88	DNAJB9	bta:614588; G3MZ88	ENSBTAG00000046588	0.01711033	0.056764065	-0.039653734
A0AAA9TXA5	RCSD1	A0AAA9TXA5	ENSBTAG00000014151	0.158064846	0.082391852	0.075672995
A0A3Q1M501	PLEKHA6	A0A3Q1M501	ENSBTAG00000004641	-0.094122177	-0.097072475	0.002950298
F1N4Z9	EEIG2	bta:514601; F1N4Z9	ENSBTAG00000005439	0.098727215	0.228564652	-0.129837438
A0AAF6YW66	MED22	A0AAF6YW66	ENSBTAG00000011556	0.173838534	0.117671824	0.056166711
A0JNI5	CLASRP	A0JNI5; bta:509208		-0.039483286	-0.054572622	0.015089336
E1BDI0	LARGE1	bta:506466; E1BDI0	ENSBTAG00000021953	-0.131001153	-0.242984315	0.111983161
A0AAA9S3U5	PPP1R37	A0AAA9S3U5	ENSBTAG00000018834	-0.196892049	-0.104633541	-0.092258508
Q32L52	GID8	bta:505570; Q32L52	ENSBTAG00000001827	0.581905942	0.622632919	-0.040726976
A0AAA9TSQ0	MRPS14	A0AAA9TSQ0	ENSBTAG00000001109	0.12647088	0.102648749	0.023822131
Q5EA10	PIGM	bta:509680; Q5EA10		0.016119665	0.13774911	-0.121629444
Q5EAC3	RAD9A	F1N0C5; Q5EAC3		0.229806428	0.426814667	-0.197008239
A0AAA9T5P5	UBE2E1	A0AAA9T5P5	ENSBTAG00000055252	-0.267857616	-0.450661409	0.182803793
A4FV21	ALG3	A4FV21; bta:614624		0.199208937	0.111661823	0.087547113
A0A3Q1LSC0	FGD4	A0A3Q1LSC0	ENSBTAG00000005453	-0.128866595	0.113682023	-0.242548618
A0AAF6Z380	FUNDC2	A0AAF6Z380	ENSBTAG00000016977	0.105832912	-0.081794091	0.187627003
F6RZG1	MDK	F6RZG1	ENSBTAG00000007740	-0.508674549	-0.514476279	0.005801731
F6RA94	DAGLB	F6RA94	ENSBTAG00000009236	-0.08549622	0.06026703	-0.145763251
F1MZC5				-0.188416364	-0.114345409	-0.074070955
E1BPH4	HPS3	bta:783104; E1BPH4	ENSBTAG00000002395	-0.248943445	-0.24618758	-0.002755865
A0A3Q1LUE0	FGFR1	A0A3Q1LUE0	ENSBTAG00000015457	-0.167992389	-0.306220339	0.13822795
A0AAA9S476				-0.028110707	0.228229047	-0.256339753
A0A3Q1LW44	PIAS2	A0A3Q1LW44	ENSBTAG00000007833	-0.022225918	-0.137373791	0.115147873
A0A3Q1LM42	ANKS6	A0A3Q1LM42	ENSBTAG00000005133	0.158140527	-0.149183155	0.307323682
F1MND2	GBA2	F1MND2	ENSBTAG00000011431	-0.023893206	0.108706259	-0.132599466
G1K1B6	POP5	G1K1B6	ENSBTAG00000005385	-0.118716061	-0.136502	0.017785939
Q8WNW7	NFKBIA	bta:282291; Q8WNW7	ENSBTAG00000016683	0.602323661	0.169536815	0.432786846
E1BJL2	PCGF6	bta:507739; E1BJL2	ENSBTAG00000009879	0.279479157	0.009131012	0.270348145
A0AAA9TR18	F3	A0AAA9TR18	ENSBTAG00000007101	0.241287376	-0.465951771	0.707239147
A5H027	RNASEL	A5H027; bta:100048947; E1BC88	ENSBTAG00000009091	0.128564722	0.220955474	-0.092390752
Q2YDC9	PDCD2	bta:512079; Q2YDC9	ENSBTAG00000011179	-0.484184731	0.072695228	-0.556879959
A0A3Q1LT05	AMMECR1L	A0A3Q1LT05; bta:539958	ENSBTAG00000008089	-0.292288211	-0.12936529	-0.162922921
Q08DU8	BLOC1S6	bta:614408; F1MY65; Q08DU8	ENSBTAG00000012250	0.212993723	0.122187439	0.090806284
E1B8U2	THUMPD2	E1B8U2	ENSBTAG00000004722	-0.538722322	-0.356956825	-0.181765497
A0AAA9S1B7	CTNNAL1	A0AAA9S1B7	ENSBTAG00000004997	0.143845051	0.030188269	0.113656782
A0AAA9RZ54	CMTM7	A0AAA9RZ54	ENSBTAG00000001712	-0.048854387	0.019087754	-0.067942141
Q58DU8	NR2C2AP	bta:508684; Q58DU8	ENSBTAG00000001763	0.00921853	0.113036108	-0.103817578
E1BE33	ZEB2	E1BE33	ENSBTAG00000012615	-0.047453919	0.032964763	-0.080418682
Q2NKY9	JAGN1	bta:539101; Q2NKY9	ENSBTAG00000010832	0.311798975	0.35869759	-0.046898615
A0A3Q1MPI7	DAAM1	A0A3Q1MPI7	ENSBTAG00000005984	0.105256878	0.054902678	0.0503542
Q08DG5	HOXB4	bta:768240; Q08DG5	ENSBTAG00000039599	-0.321025188	-0.004825072	-0.316200116
A0AAA9SBV3	VAMP5	A0AAA9SBV3	ENSBTAG00000018285	0.037474705	0.306661338	-0.269186633
A0AAF6DMA2	PDSS1	A0AAF6DMA2; bta:509696		-0.112209504	-0.388641765	0.276432261
F1N4X3	TSC22D1	F1N4X3	ENSBTAG00000046664	0.430536272	0.106915204	0.323621068
E1BQ28	SLC39A8	bta:508193; E1BQ28	ENSBTAG00000005668	-0.361608443	-0.471688366	0.110079923
E1BJM5	PASK	E1BJM5	ENSBTAG00000000785	-0.340122794	-0.129049237	-0.211073557
A0A3Q1LY39	CCSER2	A0A3Q1LY39; bta:540046	ENSBTAG00000017611	-0.076749493	-0.053146593	-0.023602899
A0AAF6Z4P3	TGFBR1	A0AAF6Z4P3	ENSBTAG00000018035	-0.017973434	0.168397532	-0.186370965
A0A3Q1NL90	NIN	A0A3Q1NL90; bta:510366	ENSBTAG00000020281	-0.114106486	-0.097871383	-0.016235103
A0AAA9S473	ZNF280D	A0AAA9S473	ENSBTAG00000056475	0.279522529	0.261651852	0.017870677
A0AAF6ZZH8	AMOTL2	A0AAF6ZZH8	ENSBTAG00000000742	0.03796785	0.169619637	-0.131651787
A0AAA9T8A6	CRPPA	A0AAA9T8A6	ENSBTAG00000015073	-0.192865522	0.289787133	-0.482652655
E1BEH7	TC2N	E1BEH7	ENSBTAG00000003375	-0.146175534	-0.087356889	-0.058818644
A0AAA9U255	LDAF1	A0AAA9U255	ENSBTAG00000018562	0.439421658	0.653731733	-0.214310075
F1N0S8	TUSC1	F1N0S8	ENSBTAG00000008551	-0.004088888	0.067794552	-0.07188344
A0AAA9SRY7	DCP2	A0AAA9SRY7	ENSBTAG00000000767	-0.046932612	-0.128714657	0.081782045
A2VE68	ATG4C	A2VE68; bta:531455		-0.065620084	0.023018617	-0.0886387
G3N0S0	UIMC1	G3N0S0	ENSBTAG00000001707	0.203320566	0.350116639	-0.146796073
A0A3Q1ME40	N4BP1	A0A3Q1ME40	ENSBTAG00000019938	-0.128572875	-0.085375186	-0.043197689
A0A3Q1M2Y5	TTYH3	A0A3Q1M2Y5	ENSBTAG00000005934	-0.293445719	-0.335904114	0.042458395
F6RDW7	NUDT7	F6RDW7	ENSBTAG00000007184	-0.010481484	-0.175905093	0.165423609
F1MSN2	TACSTD2	bta:539853; F1MSN2	ENSBTAG00000004381	0.49806519	0.668524035	-0.190458845
P05630	ATP5F1D	A6H7D8; bta:338081; P05630	ENSBTAG00000000550	0.370339054	-0.177148742	0.547487795
A0AAA9THD8	PBX2	A0AAA9THD8	ENSBTAG00000014421	0.231650924	0.681801218	-0.450150294
A0A3Q1LNE2	ARHGEF11	A0A3Q1LNE2	ENSBTAG00000012361	0.132033704	0.097206115	0.034827589
A0AAA9S123	POLK	A0AAA9S123	ENSBTAG00000004028	0.284519272	0.095356974	0.189162298
A0AAA9SX79	ARHGAP11A	A0AAA9SX79	ENSBTAG00000020100	0.040641984	-0.063991557	0.104633541
F1N0Q2	AKAP6	bta:537810; F1N0Q2	ENSBTAG00000017719	-0.126418531	0.03281187	-0.159230401
E1BAD1	C2H2orf69	bta:535735; E1BAD1	ENSBTAG00000009952	0.061126659	0.170290102	-0.109163443
A0AAA9SG42	HDAC5	A0AAA9SG42	ENSBTAG00000016254	-0.330028198	0.161942616	-0.491970815
A0A3Q1MIR9	KAT5	A0A3Q1MIR9	ENSBTAG00000005578	-0.04491845	0.228045793	-0.272964242
Q2KID7	OSTC	bta:768033; Q2KID7	ENSBTAG00000011611	-0.194280272	-0.162634645	-0.031645627
A0A3Q1MH43	USP32	A0A3Q1MH43	ENSBTAG00000000089	0.015724352	0.075712354	-0.059988002
F1MDF1	PTPN18	F1MDF1	ENSBTAG00000011658	0.310975486	0.3649255	-0.053950014
A0A452DIZ1	SUDS3	A0A452DIZ1	ENSBTAG00000019757	-0.036405646	0.052188277	-0.088593923
A0A3Q1NID4	METAP1D	A0A3Q1NID4	ENSBTAG00000019075	-0.184859333	-0.192645078	0.007785745
E1BE60	PPP1R14A	bta:615348; E1BE60	ENSBTAG00000023472	0.029580991	-0.101283336	0.130864326
A0AAA9T1N8	DMD	A0AAA9T1N8	ENSBTAG00000046838	-0.044999985	-0.167158567	0.122158583
A0A3Q1LXN7	HCFC2	A0A3Q1LXN7	ENSBTAG00000020933	-0.317841964	0.139203062	-0.457045026
A0AAA9T877	PARS2	A0AAA9T877; bta:508907	ENSBTAG00000055903	-0.046086913	-0.096820896	0.050733982
A0AAA9SVU5	SLC25A21	A0AAA9SVU5	ENSBTAG00000019350	-0.120294234	0.037321626	-0.15761586
A0AAF6ZDS6				-0.17994081	-0.024568721	-0.155372089
A0AAA9S0W0	NDUFB3	A0AAA9S0W0	ENSBTAG00000012760	-0.111097115	0.046934208	-0.158031323
A0A3Q1LX72	RGS10	A0A3Q1LX72	ENSBTAG00000002647	0.019201306	0.071978337	-0.052777031
A0AAA9S566	ARHGEF28	A0AAA9S566	ENSBTAG00000005633	0.256308543	0.208328514	0.047980029
A0A3Q1MSW4	TBC1D1	A0A3Q1MSW4	ENSBTAG00000013699	0.165328661	-0.167434836	0.332763496
A0A3Q1M4P0	NPR2	A0A3Q1M4P0	ENSBTAG00000011434	-0.119121742	-0.010314218	-0.108807524
A1A4H5	CRTC1	A1A4H5; bta:510465		0.149769572	0.234863733	-0.085094161
A0A3Q1LP80	DERPC	A0A3Q1LP80	ENSBTAG00000001898	-0.200673452	-0.008028374	-0.192645078
A0A3Q1N1A7	CPB2	A0A3Q1N1A7	ENSBTAG00000007073	-0.122817699	0.019201306	-0.142019005
A0AAA9S7Q8	TRAPPC2L	A0AAA9S7Q8	ENSBTAG00000019184	-0.019425499	0.042413755	-0.061839254
Q24JZ5	TMEM199	bta:511376; Q24JZ5		-0.079238649	0.161166846	-0.240405495
E1BE01	SOX6	E1BE01	ENSBTAG00000044185	-0.185009329	0.097390402	-0.282399731
F1MZ35	POLR2I	F1MZ35	ENSBTAG00000015002	0.566391753	0.193941883	0.37244987
E1BDK9	RETSAT	bta:614455; E1BDK9	ENSBTAG00000021934	-0.158833587	-0.017629894	-0.141203693
A4FV53	GTPBP2	A4FV53; bta:513605	ENSBTAG00000006016	0.024440859	0.24234589	-0.21790503
Q5EA03	TMEM186	bta:513552; Q5EA03	ENSBTAG00000022530	-0.274127374	-0.315547301	0.041419927
A0AAA9TPF4				-0.222895015	0.183761111	-0.406656126
E1BJE9	SMCR8	E1BJE9	ENSBTAG00000017090	0.070535802	0.013776503	0.056759299
Q2TBW9	ING5	bta:614733; Q2TBW9	ENSBTAG00000009123	-0.06726687	0.076483086	-0.143749956
Q2TBQ0	TFB1M	bta:533438; Q2TBQ0	ENSBTAG00000031895	-0.030526676	-0.116796335	0.086269659
A0A452DIL0	FAM193B	A0A452DIL0	ENSBTAG00000015192	-0.265165418	-0.157336538	-0.10782888
A0AAA9SGD2	DXO	A0AAA9SGD2	ENSBTAG00000005588	-0.311461096	0.00698643	-0.318447526
A2VE77	COP1	A2VE77; bta:519896	ENSBTAG00000031385	0.357261168	0.448707523	-0.091446355
E1BLW3	LZTR1	E1BLW3	ENSBTAG00000011292	-0.986238099	-0.199906427	-0.786331671
A0AAA9SA39	NEPRO	A0AAA9SA39; bta:516064	ENSBTAG00000005723	-0.259683196	-0.342342271	0.082659075
A0AAA9TZD2				-0.165447558	0.109749486	-0.275197045
Q2KJ48	CDR2	bta:534647; Q2KJ48	ENSBTAG00000018050	0.006688445	-0.060235107	0.066923552
F6PSM0	DKK3	F6PSM0	ENSBTAG00000004275	0.025455581	0.300315422	-0.274859841
A0A3Q1M282	WDFY2	A0A3Q1M282	ENSBTAG00000008053	0.141255659	0.377933505	-0.236677846
A0AAF6Z3W7	PAXIP1	A0AAF6Z3W7	ENSBTAG00000017505	0.074797031	0.028682755	0.046114276
A0AAA9TDE5	DTWD1	A0AAA9TDE5	ENSBTAG00000006201	-0.207413423	-0.156953371	-0.050460052
A0AAA9T8R5	FXYD6	A0AAA9T8R5	ENSBTAG00000014354	0.176605067	-0.120063076	0.296668142
Q58DL9	PLTP	bta:505640; Q58DL9	ENSBTAG00000000559	0.235216462	0.232021123	0.003195339
G3N1G1	RUSF1	G3N1G1	ENSBTAG00000047734	-0.148571123	0.055393634	-0.203964758
A0AAA9TCZ9	DPH7	A0AAA9TCZ9	ENSBTAG00000038691	0.065712841	0.250137412	-0.184424571
F6RGV5	MED20	F6RGV5	ENSBTAG00000010100	0.360043467	0.288768779	0.071274688
F1MNH5	DESI2	F1MNH5	ENSBTAG00000001478	-0.136309514	0.18649356	-0.322803074
Q2HJH8	CFAP300	bta:617430; Q2HJH8	ENSBTAG00000015309	-0.418968551	0.111848935	-0.530817486
A0A3Q1M0J3	CHCHD2	A0A3Q1M0J3	ENSBTAG00000040295	-0.624435868	-0.214494436	-0.409941432
Q17QY0	MYBL2	bta:510420; Q17QY0	ENSBTAG00000007783	-0.317190176	-0.330768582	0.013578407
A0AAF6ZA20	UXT	A0AAF6ZA20	ENSBTAG00000015820	0.574288999	0.547860874	0.026428125
A0AAA9S0S8	FIRRM	A0AAA9S0S8	ENSBTAG00000014793	0.112080927	0.067686808	0.044394119
E1BPC3	ATP8B1	bta:541187; E1BPC3	ENSBTAG00000005738	-0.177556773	-0.04761802	-0.129938753
E1B6Z5	CCNT2	E1B6Z5	ENSBTAG00000014158	0.22422908	0.296340492	-0.072111413
Q3MHN2	C9	bta:526766; Q3MHN2	ENSBTAG00000016149	-0.053909164	0.033838748	-0.087747912
A0AAA9SGM8	MKNK1	A0AAA9SGM8	ENSBTAG00000019933	-0.266813345	0.015357041	-0.282170386
A0A452DJ03	VWA1	A0A452DJ03	ENSBTAG00000021294	-0.112560806	0.437552294	-0.5501131
A0A3Q1N0H8	FRA10AC1	A0A3Q1N0H8	ENSBTAG00000000447	0.037749277	-0.021579174	0.059328451
A0AAA9T2G4	PGS1	A0AAA9T2G4	ENSBTAG00000000675	-0.166810621	0.137622858	-0.30443348
A5PJR3	DCXR	A5PJR3		-0.128556035	0.140823882	-0.269379917
A0AAF6YVJ7				0.23463398	0.08358663	0.15104735
A0A3Q1N7G2	PALD1	A0A3Q1N7G2	ENSBTAG00000008583	-0.183300273	-0.099074675	-0.084225598
Q3MHM0	APBB1IP	bta:533670; Q3MHM0	ENSBTAG00000012526	-0.178550248	-0.538179052	0.359628804
A0A3Q1M7E0	DHCR24	A0A3Q1M7E0	ENSBTAG00000004688	0.100905754	-0.008592611	0.109498365
F1N1V2	CEPT1	F1N1V2	ENSBTAG00000020169	0.091410485	0.163396598	-0.071986113
F1MNS5	ENPP1	bta:615535; F1MNS5	ENSBTAG00000021830	-0.148793537	0.099321449	-0.248114986
A0A3Q1M1G0				-0.175086707	0.18693896	-0.362025666
E1BDL0	PCDHB15	E1BDL0	ENSBTAG00000062518	-0.034730545	0.051956677	-0.086687222
A0AAF6ZX18	UBE2E3	A0AAF6ZX18	ENSBTAG00000043956	-0.078943203	-0.129323707	0.050380504
G3X6H5	JADE3	G3X6H5	ENSBTAG00000005124	0.038889013	0.056684596	-0.017795583
A0AAA9SSM2	MRPL18	A0AAA9SSM2	ENSBTAG00000001289	-0.095383156	-0.018578796	-0.07680436
E1BGF9	ZHX3	bta:512451; E1BGF9	ENSBTAG00000017594	-0.198843348	-0.12388129	-0.074962058
A0A3Q1MUT2	DPY19L3	A0A3Q1MUT2; bta:521976	ENSBTAG00000015165	-0.115151736	-0.081696439	-0.033455297
A0AAA9TS92	GTPBP8	A0AAA9TS92	ENSBTAG00000006534	0.135750143	0.063807969	0.071942174
A0AAA9T8L6	SNX14	A0AAA9T8L6	ENSBTAG00000018408	-0.569802694	-0.581579861	0.011777168
O02659	MBL	A4IFQ1; bta:281297; O02659	ENSBTAG00000007049	-0.223318413	-0.062427223	-0.16089119
Q6B4J2	VKORC1	A2VDX1; bta:445422; Q6B4J2		0.343281659	0.410334681	-0.067053022
A6QLK2	NABP2	A6QLK2; bta:533842	ENSBTAG00000002798	-0.222155778	0.076058078	-0.298213856
A0AAA9RXL2	SLC17A5	A0AAA9RXL2	ENSBTAG00000044053	-0.231554658	-0.311689656	0.080134998
E1BC46	KIF18B	E1BC46	ENSBTAG00000008088	-0.228905728	-0.40762955	0.178723821
A0AAA9SVM7	CETN4	A0AAA9SVM7	ENSBTAG00000016147	-0.220323052	-0.221932305	0.001609253
A0A3Q1M4X6	SMUG1	A0A3Q1M4X6	ENSBTAG00000021974	-0.455994174	-0.064756208	-0.391237965
A0A3Q1MCW8	MGAT5	A0A3Q1MCW8; bta:537595	ENSBTAG00000018744	0.222392421	0.611434712	-0.389042291
A0A3Q1LTS5	COX16	A0A3Q1LTS5	ENSBTAG00000030595	-0.36801833	-0.305526488	-0.062491842
A0A452DIV4	RABAC1	A0A452DIV4	ENSBTAG00000018633	-0.200077835	-0.257854167	0.057776331
A0A3Q1MCG3	NT5E	A0A3Q1MCG3	ENSBTAG00000048655	-0.005527574	0.002755865	-0.008283439
F1MB88	ZBTB14	bta:101904037; F1MB88	ENSBTAG00000038970	-0.080170349	0.154849681	-0.23502003
F1MUR2	KDM5B	F1MUR2	ENSBTAG00000006175	-0.053965358	0.072047789	-0.126013147
Q6Y1E2	PLP2	bta:399683; Q3ZBT4; Q6Y1E2	ENSBTAG00000016093	0.01129317	-0.005679909	0.016973079
A0AAA9TMB1	MANEA	A0AAA9TMB1	ENSBTAG00000005026	-0.19419553	0.155388908	-0.349584438
A0AAF6YWS3	BLOC1S1	A0AAF6YWS3	ENSBTAG00000011918	0.364683299	0.561940283	-0.197256983
A0A3Q1LVM5	ITIH1	A0A3Q1LVM5	ENSBTAG00000007843	0.195894392	0.847454642	-0.651560249
A0A452DJC9	TMEM70	A0A452DJC9	ENSBTAG00000012920	-0.160129544	0.181778344	-0.341907888
Q2KJJ1	SLC25A17	bta:506631; Q2KJJ1	ENSBTAG00000006200	-0.046105081	-0.177284	0.131178919
A0A3Q1LHC4	HOMER2	A0A3Q1LHC4	ENSBTAG00000017715	-0.480142224	-0.34191957	-0.138222654
A0AAA9SB84	SLC7A2	A0AAA9SB84	ENSBTAG00000010085	-0.285230152	-0.239007135	-0.046223017
A5PJR2	MCL1	A5PJR2; bta:788087	ENSBTAG00000015154	0.061805284	0.262764516	-0.200959232
F6RR15	LSR	F6RR15	ENSBTAG00000017033	0.021602254	0.129756731	-0.108154476
A0A3S5ZPU2	AGPAT5	A0A3S5ZPU2	ENSBTAG00000004922	-0.177629666	-0.206155364	0.028525698
Q5E9E8	YIPF5	A1L5B9; bta:521829; Q5E9E8	ENSBTAG00000012758	-0.309798873	-0.17994081	-0.129858063
A2VE72	FAM8A1	A2VE72; bta:511911	ENSBTAG00000017118	0.044020413	0.158922507	-0.114902095
A0A3Q1LR05	INTS11	A0A3Q1LR05	ENSBTAG00000007620	-0.063115385	-0.124814371	0.061698986
A0AAA9T2W9	GSTCD	A0AAA9T2W9	ENSBTAG00000013927	-0.284992048	0.069201127	-0.354193174
A0AAA9TNB8	POLR3H	A0AAA9TNB8	ENSBTAG00000005311	-0.056583528	-0.084975129	0.028391601
Q3SZ68	LAMTOR5	bta:516090; Q3SZ68	ENSBTAG00000014970	0.238542586	0.059598052	0.178944534
P62866	FAU	P62866; Q8HYI8		-0.416668585	-0.21446718	-0.202201404
F1MGP5	MTRR	F1MGP5	ENSBTAG00000009401	-0.128416273	-0.322919297	0.194503024
A0A3Q1MAZ0	MEX3D	A0A3Q1MAZ0	ENSBTAG00000040334	0.021294778	0.004016787	0.017277991
F1N2C1	C3H1orf43	F1N2C1	ENSBTAG00000020026	0.298628007	0.49788793	-0.199259922
Q3SZT5	RABL2B	bta:515671; Q3SZT5	ENSBTAG00000004876	0.063483534	0.444481277	-0.380997743
A0A3Q1MEI1	VPS13B	A0A3Q1MEI1	ENSBTAG00000012357	0.015673878	0.014255996	0.001417882

Accession	Gene_Symbol	Gene_ID	Ensembl_Gene ID	log2(ratio(OV24H/OV0H))	log2(ratio(OV24R/OV0H))	log2(ratio(OV24H/OV24R))
A0AAA9T9X9	DIABLO	A0AAA9T9X9	ENSBTAG00000004199	0.872352177	0.394531844	0.477820333
A0A3Q1LTF3	HAL	A0A3Q1LTF3	ENSBTAG00000016276	0.312367457	1.293053982	-0.980686525
A0A3Q1NMJ3	NCBP2AS2	A0A3Q1NMJ3; bta:783161	ENSBTAG00000048390	0.272780446	0.410283969	-0.137503524
A0AAA9S7S7	DNAJC16	A0AAA9S7S7	ENSBTAG00000002476	-0.042749647	0.128035997	-0.170785644
A0AAF7AQ98	IGF2	A0AAF7AQ98	ENSBTAG00000013066	0.339385292	0.227758931	0.111626361
A0AAA9SAY2	TRIT1	A0AAA9SAY2	ENSBTAG00000013832	-0.315872703	0.071039337	-0.38691204
A0AAA9S8W1	NAPEPLD	A0AAA9S8W1	ENSBTAG00000014171	-0.270166667	0.218434408	-0.488601075
A0AAA9SDZ4	SYNE3	A0AAA9SDZ4	ENSBTAG00000003526	-0.102550437	0.170943063	-0.2734935
E1BG09	RTTN	E1BG09	ENSBTAG00000013221	-0.098257952	-0.037655851	-0.060602101
E1BN04	ZMYND11	E1BN04	ENSBTAG00000002578	-0.06809998	0.04940224	-0.11750222
A0AAA9STE8	PLPP1	A0AAA9STE8	ENSBTAG00000010526	0.103269271	0.479223247	-0.375953977
Q2TBX9	ITFG1	bta:512545; Q2TBX9	ENSBTAG00000004805	-0.888931504	-0.299019788	-0.589911717
Q0P572	MBIP	bta:774027; Q0P572		-0.19326537	-0.178451485	-0.014813884
A0AAA9SAV7	AGPAT2	A0AAA9SAV7	ENSBTAG00000025161	0.012437103	0.182774834	-0.170337731
A0AAA9SUE0	TPK1	A0AAA9SUE0	ENSBTAG00000017676	0.42707407	0.57201183	-0.14493776
A0A3Q1M864	GAB2	A0A3Q1M864	ENSBTAG00000002922	-0.16888597	0.084770103	-0.253656074
A2VE11	IGSF8	A2VE11; bta:519266		-0.340424439	-0.004601902	-0.335822537
Q32L97	VAMP4	A5PK89; bta:616923; Q32L97	ENSBTAG00000020591	-0.114458725	-0.130727114	0.016268389
E1BJ67	CCNL1	bta:514613; E1BJ67	ENSBTAG00000003851	-1.128260675	-0.062230805	-1.06602987
A0A3Q1LHL4	USP12	A0A3Q1LHL4	ENSBTAG00000017179	-0.285613105	-0.285613105	0
A7MB73	CERCAM	A7MB73; bta:516122	ENSBTAG00000038139	-0.244440214	-0.412711697	0.168271483
A0AAF7AMH2	ETV6	A0AAF7AMH2	ENSBTAG00000014605	0.111361109	-0.079322453	0.190683562
A0AAA9S8E2	PRSS23	A0AAA9S8E2	ENSBTAG00000015177	-0.669391282	-0.335916433	-0.333474849
A0AAF6Z0C8	PTCD2	A0AAF6Z0C8	ENSBTAG00000014675	-0.116770534	-0.196571246	0.079800711
F1MML3	BCORL1	bta:540023; F1MML3	ENSBTAG00000004962	0.223357435	0.44181683	-0.218459396
A6H728	MUTED	A6H728; bta:505520		0.080949108	0.252051065	-0.171101957
Q28024	GNG12	A5PJE1; bta:286850; Q28024	ENSBTAG00000061772	0.107008543	0.343716784	-0.236708241
F6RMS7	CENPQ	F6RMS7	ENSBTAG00000020710	0.309492616	0.463008761	-0.153516146
F1N5Q1	ZNF691	F1N5Q1	ENSBTAG00000016028	0.140094253	0.276482722	-0.136388469
A0AAA9T3J5	RRAD	A0AAA9T3J5	ENSBTAG00000013929	-0.588756074	-0.364411426	-0.224344648
A7E301	SHROOM1	A7E301; bta:615958	ENSBTAG00000014676	0.280651819	0.451563375	-0.170911556
A0A3Q1LLM3	HOMER1	A0A3Q1LLM3	ENSBTAG00000025853	-0.176134756	0.283121278	-0.459256034
F1N7E7	TSHZ3	F1N7E7	ENSBTAG00000002687	0.012551317	0.034014032	-0.046565349
Q5EA18	CENPL	A0JNC6; bta:615527; Q5EA18	ENSBTAG00000006925	0.116944116	0.149064813	-0.032120697
A0AAA9TEA9				-0.185765989	-0.136297313	-0.049468676
E1B9E6	TRAF3IP1	E1B9E6	ENSBTAG00000015973	0.301516635	0.450526584	-0.149009949
Q2KJ51	ANGPTL4	bta:509963; Q2KJ51	ENSBTAG00000002473	-0.059515285	0.379345792	-0.438861077
Q3SZV5	POMP	bta:510314; Q3SZV5	ENSBTAG00000014024	0.052161421	-0.266465311	0.318626732
A2VDV9	SMIM8	A2VDV9; bta:533179	ENSBTAG00000021663	0.168948426	0.394240738	-0.225292312
A0AAA9SZM8	HTATIP2	A0AAA9SZM8	ENSBTAG00000013419	-0.018821176	0.154015421	-0.172836597
Q05B49				-0.13929495	-0.092413339	-0.046881611
Q3T0U4	ADIPOR1	bta:407235; F1MRN0; Q3T0U4	ENSBTAG00000009727	0.305936062	0.324198269	-0.018262206
A0AAF6Z5S5	POLG2	A0AAF6Z5S5	ENSBTAG00000018420	-0.157869972	-0.008570491	-0.149299481
A0AAA9RX60	ILRUN	A0AAA9RX60	ENSBTAG00000034493	0.003011891	0.094804366	-0.091792476
A0AAA9RU38	WDSUB1	A0AAA9RU38	ENSBTAG00000004184	-0.006313774	0.275839862	-0.282153636
A0AAA9TZZ2	CCDC97	A0AAA9TZZ2	ENSBTAG00000039635	-0.254382536	-0.005433886	-0.24894865
A0A3Q1LTZ7	PLXNC1	A0A3Q1LTZ7	ENSBTAG00000011245	-0.226015216	-0.093767418	-0.132247798
E1BMD3	MSRB3	E1BMD3	ENSBTAG00000044017	-0.110632006	0.167889779	-0.278521785
A6QQR2	CLCN7	A6QQR2		0.35678351	0.413461644	-0.056678134
E1BGZ9	PHF20L1	E1BGZ9	ENSBTAG00000006869	0.054883907	0.317209546	-0.262325639
A0A3Q1NLB0				0.314022897	0.575735548	-0.261712651
A0AAA9S157	ADAT3	A0AAA9S157	ENSBTAG00000062956	-0.210945895	-0.39223455	0.181288655
A0AAA9SA51	RPUSD3	A0AAA9SA51	ENSBTAG00000007968	-0.278638146	-0.028790879	-0.249847268
F1MLZ2	SLC20A1	bta:514255; F1MLZ2	ENSBTAG00000007895	-0.404437194	-0.404437194	0
A0A3Q1LJI3	ETS1	A0A3Q1LJI3	ENSBTAG00000002341	0.268593931	0.253487039	0.015106892
Q2M2U3	LAMTOR4	bta:511903; Q2M2U3		0.058955407	0.07808372	-0.019128312
A0AAA9S6C8	AXIN1	A0AAA9S6C8	ENSBTAG00000016577	0.101003319	-0.167689731	0.268693049
A0A3Q1LPR8	SNX24	A0A3Q1LPR8	ENSBTAG00000031069	0.33818942	0.59738176	-0.25919234
A0AAA9TFR4	GM2A	A0AAA9TFR4	ENSBTAG00000021829	0.101008563	-0.086542109	0.187550672
F1MIH4	CLIC5	F1MIH4	ENSBTAG00000010244	-0.04161219	-0.032903742	-0.008708447
A0AAA9TD00	CEBPB	A0AAA9TD00	ENSBTAG00000051972	0.311166902	0.627071209	-0.315904307
E1B9A0	SLC36A1	bta:518759; E1B9A0	ENSBTAG00000016094	0.111748358	0.272684576	-0.160936218
E1BFC5	FAM222B	bta:506074; E1BFC5	ENSBTAG00000047717	-0.050411626	-0.09461143	0.044199804
A0AAA9S626	DMAC2L	A0AAA9S626	ENSBTAG00000015202	0.066709342	0.111361109	-0.044651766
A0AAF6YY86	UNC119	A0AAF6YY86	ENSBTAG00000013096	-0.207052502	0.039845336	-0.246897837
A0AAA9S1W0	FCHSD1	A0AAA9S1W0	ENSBTAG00000049248	0.120294234	0.518697999	-0.398403765
F1MBE6	E2F4	F1MBE6	ENSBTAG00000012063	-0.08403458	-0.091922489	0.007887909
E1BM52	FOXP4	bta:517805; E1BM52	ENSBTAG00000014211	-0.106180447	-0.214452418	0.108271971
A0A3Q1LTK4	DLG5	A0A3Q1LTK4	ENSBTAG00000013187	-0.155343219	0.52213142	-0.67747464
F6RQN4	C1QTNF3	F6RQN4	ENSBTAG00000017071	0.381957553	0.083499996	0.298457557
F1MS13	PHF21A	F1MS13	ENSBTAG00000009611	-0.371041595	-0.245793956	-0.12524764
F1MQ33	SCAP	F1MQ33	ENSBTAG00000015782	-0.192848146	0.116023796	-0.308871942
A0A3Q1MGD5	IRF5	A0A3Q1MGD5	ENSBTAG00000004989	-0.222162124	-0.193434947	-0.028727178
A0AAF6DLG7	USP38	A0AAF6DLG7	ENSBTAG00000001356	-0.086542109	0.098257952	-0.184800061
Q17QE3	POLR2M	bta:100125877; Q17QE3	ENSBTAG00000040384	0.080401507	0.239801328	-0.159399822
A0AAF6DMS1	SELENON	A0AAF6DMS1	ENSBTAG00000021778	0.102433234	0.517875928	-0.415442694
A0AAA9T4M0	MYO19	A0AAA9T4M0	ENSBTAG00000012675	-0.168154606	0.030845967	-0.199000573
F1N3J9	OGFRL1	F1N3J9	ENSBTAG00000016374	-0.375144772	-0.246944703	-0.128200069
P81644	APOA2	bta:505394; P81644; Q2NKV9	ENSBTAG00000009212	0.807666688	0.416128384	0.391538304
Q0IIH0	WDR45	bta:539190; Q0IIH0		0.242570303	0.504563428	-0.261993125
F1MW52	ADAM12	F1MW52	ENSBTAG00000012444	-0.187835199	-0.036045538	-0.151789661
A0AAA9SUN1	MTERF3	A0AAA9SUN1	ENSBTAG00000001522	-0.072252072	0.018496344	-0.090748416
A0AAA9SG79	GPR180	A0AAA9SG79	ENSBTAG00000005310	0.113789657	0.015989731	0.097799926
A0A3Q1M7C3	CASP8AP2	A0A3Q1M7C3	ENSBTAG00000011313	-0.765294317	-0.23305154	-0.532242778
A0A3Q1LU95	STAMBPL1	A0A3Q1LU95	ENSBTAG00000012298	0.170090762	0.195067427	-0.024976666
Q2M2S4	PNKD	Q2M2S4	ENSBTAG00000047277	0.0716821	-0.269760269	0.341442369
A0A3Q1N125	C7H19orf67	A0A3Q1N125	ENSBTAG00000016937	-0.180956631	-0.080005643	-0.100950988
B0JYN5	MRFAP1	B0JYN5; bta:522338		0.165222382	0.01052518	0.154697203
A0AAA9RVV3	SCAPER	A0AAA9RVV3	ENSBTAG00000007382	0.331205908	0.324074431	0.007131478
A4FUY5	TOB2	A4FUY5; bta:507934	ENSBTAG00000015109	0.266182017	0.010144557	0.25603746
F6Q268	ACACB	F6Q268	ENSBTAG00000022058	0.017623368	0.078426647	-0.060803279
A6QNP8	ESCO2	A6QNP8; bta:538949	ENSBTAG00000006551	-0.533123569	-0.461387816	-0.071735754
A0AAA9S3B4	ZDHHC17	A0AAA9S3B4; bta:541172	ENSBTAG00000021756	0.100987808	0.00869096	0.092296847
A6QQJ8	ZC3H12A	A6QQJ8; bta:535344	ENSBTAG00000011316	0.019108823	-0.111608506	0.130717329
A0AAF7A521	TIPIN	A0AAF7A521	ENSBTAG00000000372	-0.116141595	0.208893154	-0.325034749
F6PT78	TDG	F6PT78	ENSBTAG00000021633	-0.460510039	-0.115374553	-0.345135486
A0AAA9RWM0	SFT2D3	A0AAA9RWM0; bta:514114	ENSBTAG00000066017	-0.331565788	-0.372698037	0.04113225
A0AAA9SJV5	GINS2	A0AAA9SJV5	ENSBTAG00000044006	0.352433491	0.395137942	-0.042704451
A0A3Q1MJL6	GPRC5C	A0A3Q1MJL6	ENSBTAG00000018663	-0.134727824	-0.179475035	0.04474721
F1N547	CETN3	F1N547	ENSBTAG00000014373	0.291048782	0.10718459	0.183864192
G3MYM4	ZNF292	G3MYM4	ENSBTAG00000046612	0.068149592	0.084359819	-0.016210227
A0AAA9S1Q8	FUT11	A0AAA9S1Q8; bta:539329	ENSBTAG00000002792	-0.248444966	-0.294112717	0.045667751
A4IFN5	FITM2	A4IFN5; bta:518159	ENSBTAG00000020030	0.578548476	0.498299411	0.080249065
E1BD50	TTC33	bta:531278; E1BD50	ENSBTAG00000000012	0.363216303	0.45802067	-0.094804366
Q0P5D9	ALG8	bta:538731; Q0P5D9		-0.167109986	-0.186931427	0.019821441
A5PJN5	TCAF1	A5PJN5; bta:533126		-0.434666306	-0.264009529	-0.170656777
A0AAA9SRG3	NARF	A0AAA9SRG3	ENSBTAG00000018178	-0.573219168	-0.332369842	-0.240849326
Q6L708	CLDN1	bta:414922; Q3ZC38; Q6L708	ENSBTAG00000013148	0.450603818	0.457498431	-0.006894613
A0AAF6YMI8	KDELR1	A0AAF6YMI8	ENSBTAG00000004463	0.120030704	0.354495957	-0.234465254
A0A3Q1LLN6	MTSS1	A0A3Q1LLN6	ENSBTAG00000020407	-0.075257676	-0.175691945	0.100434269
A6QLD8	ADAMTSL4	A6QLD8; bta:507654		0.263034406	0.381318424	-0.118284018
A0AAA9RZI0	AHI1	A0AAA9RZI0	ENSBTAG00000017958	0.065628668	0.221614478	-0.15598581
Q2YDH1	ANAPC10	bta:783986; Q2YDH1		0.038172502	0.137894198	-0.099721696
Q1JQ94	RTL8A; RTL8B; RTL8C	bta:511516; bta:615809; bta:783730; Q1JQ94	ENSBTAG00000039890; ENSBTAG00000056216; ENSBTAG00000062559	-0.418065192	-0.218365357	-0.199699835
A8E659	ANKH	A8E659; bta:511800		-0.008679756	-0.013663163	0.004983408
A0AAF6YYH7	AP5M1	A0AAF6YYH7	ENSBTAG00000013043	0.598969359	0.307783913	0.291185446
A0AAA9S3U7	ICOSLG	A0AAA9S3U7	ENSBTAG00000014880	-0.273239188	-0.025648587	-0.2475906
G3N047	TTC7B	G3N047	ENSBTAG00000006392	0.041180636	0.406808467	-0.365627831
F1N5H2	FUCA2	bta:515729; F1N5H2	ENSBTAG00000001752	0.042955565	0.388832429	-0.345876864
A0A3Q1M939	RNF121	A0A3Q1M939	ENSBTAG00000014167	-0.268209336	-0.256408084	-0.011801252
F1MHF3	NAV2	F1MHF3	ENSBTAG00000018431	-0.102394383	-0.159550334	0.057155951
A0AAF6ZGE1	BOLA1	A0AAF6ZGE1	ENSBTAG00000006050	-0.2205133	-0.064899775	-0.155613525
A0A452DIY8	MRPL55	A0A452DIY8; bta:506657	ENSBTAG00000020721	0.006821266	0.035457134	-0.028635868
A0AAF6DLW8	NRADD	A0AAF6DLW8	ENSBTAG00000022598	-0.025484972	0.136482508	-0.16196748
Q95LI3	ZFY	bta:280962; Q95LI3		0.171863516	0.236606281	-0.064742765
Q3T139	RNF146B	bta:767901; Q3T139	ENSBTAG00000034531	0.160854011	0.074962058	0.085891953
Q5E950	NHP2	bta:520891; Q3SZ42; Q5E950	ENSBTAG00000006374	-0.478848179	-0.160231973	-0.318616206
Q148C8	MIEN1	bta:505710; Q148C8	ENSBTAG00000021802	0.622115499	0.291134941	0.330980558
Q58DH7	SIGMAR1	bta:538903; Q58DH7	ENSBTAG00000015804	0.279013121	0.353710488	-0.074697367
A1A4I1	SLC20A2	A1A4I1; bta:518905		0.063446515	0.208284202	-0.144837687
A3KMZ6	COX11	A3KMZ6; bta:510509	ENSBTAG00000007015	-0.024416527	-0.248016527	0.223600001
E1BM28	DNPH1	E1BM28	ENSBTAG00000019913	-0.077560983	0.596836601	-0.674397584
E1BKV4	BACE2	E1BKV4	ENSBTAG00000000394	0.117295359	0.019575538	0.097719821
A0AAA9PWD1	FDX2	A0AAA9PWD1; bta:505159	ENSBTAG00000010318	0.683673665	0.395381949	0.288291716
E1BEH8	SLC35B4	bta:613784; E1BEH8	ENSBTAG00000003372	0.361355178	0.30180299	0.059552188
Q3ZBZ7	CCDC109B	bta:504728; Q3ZBZ7		-0.254909218	-0.112086159	-0.142823058
A0AAA9S3X9	SLC39A7	A0AAA9S3X9	ENSBTAG00000008191	0.269449245	-0.053439259	0.322888504
F1N4N7	CACHD1	F1N4N7	ENSBTAG00000016455	-0.021190516	0.076802612	-0.097993128
A0AAA9TUV8	MTURN	A0AAA9TUV8	ENSBTAG00000066764	0.842840473	0.241177778	0.601662694
A0AAA9RXW4	C6H4orf3	A0AAA9RXW4	ENSBTAG00000048273	1.304642965	0.111141027	1.193501938
Q3ZC71	DPM3	bta:509745; Q3ZC71	ENSBTAG00000003535	0.339867145	-0.022389509	0.362256654
A0A3S7IDQ1	MDA-5	A0A3S7IDQ1		0.202512928	0.396804814	-0.194291886
A0AAA9T6R4	NET1	A0AAA9T6R4	ENSBTAG00000007648	-0.131614332	0.026161587	-0.157775919
Q08D99	TMEM41A	bta:505368; Q08D99	ENSBTAG00000017115	0.079434467	0.257921482	-0.178487015
A0A3Q1MLN4	PACRGL	A0A3Q1MLN4	ENSBTAG00000033351	-0.186570564	0.031609607	-0.21818017
A0AAA9SBK8	ULK1	A0AAA9SBK8	ENSBTAG00000012987	0.078237574	-0.106571664	0.184809239
Q2KJ54	SPINT2	bta:507484; Q2KJ54	ENSBTAG00000000182	-0.31382925	0.277238614	-0.591067863
E1BHQ9	MCAM	E1BHQ9	ENSBTAG00000006835	-0.217922206	0.094787359	-0.312709565
A0AAA9T5S7	LCLAT1	A0AAA9T5S7	ENSBTAG00000054158	0.124806456	0.104740833	0.020065623
F1MHQ4	FABP4	F1MHQ4	ENSBTAG00000037526	-0.241705896	-0.076830585	-0.164875311
F1N076	CP	F1N076	ENSBTAG00000012164	0.03956852	0.261547857	-0.221979338
A0AAA9SKP9	ZBTB21	A0AAA9SKP9	ENSBTAG00000030836	0.149114814	0.603016336	-0.453901522
A0A452DJ42	ATP5PF	A0A452DJ42	ENSBTAG00000000605	0.488326718	0.894647	-0.406320282
A0A3Q1MVZ0	ANTXR1	A0A3Q1MVZ0; bta:616010	ENSBTAG00000007808	-0.203382129	0.112017054	-0.315399183
A0AAA9T1V8	AVL9	A0AAA9T1V8	ENSBTAG00000003114	-0.062046138	-0.002701677	-0.059344461
Q3SX46	C1GALT1C1	bta:531644; Q3SX46	ENSBTAG00000021410	-0.124604763	-0.038356115	-0.086248648
A0AAF7APJ1	COA6	A0AAF7APJ1	ENSBTAG00000051467	0.208381312	0.128324097	0.080057215
A0AAF6DLT4	ST3GAL1	A0AAF6DLT4	ENSBTAG00000001156	-0.179649511	-0.119752557	-0.059896954
A0AAA9RUU3	CD47	A0AAA9RUU3	ENSBTAG00000003585	-1.635791282	-0.240285952	-1.39550533
F6Q4D3	UBA7	F6Q4D3	ENSBTAG00000012335	0.026057813	0.270901287	-0.244843474
A0A3Q1MH81	TTC38	A0A3Q1MH81	ENSBTAG00000003017	-0.117377277	0.206370925	-0.323748202
Q6QRN8	LAPTM4A	bta:404135; Q6QRN8	ENSBTAG00000020894	0.072039475	0.012010018	0.060029458
A0A3Q1MCU3	CDC25B	A0A3Q1MCU3	ENSBTAG00000008436	-0.948690906	-0.483267104	-0.465423803
A0AAA9TTD4	WDR37	A0AAA9TTD4	ENSBTAG00000000683	0.106915204	0.086156644	0.02075856
A7E348	PYGO2	A7E348; bta:540401		0.379877988	0.439466785	-0.059588796
A0AAF6DLW0	ADPRM	A0AAF6DLW0	ENSBTAG00000046919	-0.232235728	0.080134998	-0.312370726
E1BAP3	SP110	E1BAP3	ENSBTAG00000015752	-0.120055752	-0.029090372	-0.09096538
A7E380	SAMD9	A7E380		-0.525063163	-0.287475725	-0.237587438
F1N7F8	PIGU	F1N7F8	ENSBTAG00000011620	0.228091709	0.394910799	-0.166819091
C9EGS9	CD19	bta:517359; C9EGS9		-0.408222678	-0.295781943	-0.112440735
F6QB59	GMNN	F6QB59	ENSBTAG00000017329	0.037644774	-0.55055247	0.588197244
A6QNU8	TPGS2	A6QNU8; bta:540456	ENSBTAG00000021583	-0.203990052	-0.470064912	0.26607486
F1MS81	RMND1	F1MS81	ENSBTAG00000013224	0.069559564	0.210351908	-0.140792344
A0AAA9T095	SLC30A7	A0AAA9T095	ENSBTAG00000019027	-0.040800531	-0.002777085	-0.038023446
A0AAA9RXB9	TANK	A0AAA9RXB9	ENSBTAG00000006654	0.049286938	0.194867251	-0.145580313
A0A452DJ75	CALML4	A0A452DJ75	ENSBTAG00000025822	-0.005353234	0.070389328	-0.075742562
A0A3Q1M3Z8	QPRT	A0A3Q1M3Z8	ENSBTAG00000018082	-0.090706914	0.175923742	-0.266630656
E1BPN4	CHST3	bta:506758; E1BPN4	ENSBTAG00000011014	0.211571362	0.136830782	0.07474058
A0A3Q1NN05	CASTOR2	A0A3Q1NN05	ENSBTAG00000014417	-0.127516698	-0.197472524	0.069955826
P0CG11	CHTF8	A8E4M1; bta:618186; P0C6S9; P0CG11		0.034140141	-0.187128073	0.221268214
A0A3Q1NCH3	CEP152	A0A3Q1NCH3	ENSBTAG00000011661	-0.010063683	-0.224303909	0.214240226
A0A3Q1M0G5	DNAJC24	A0A3Q1M0G5	ENSBTAG00000050888	0.025535092	0.135849373	-0.110314281
F6PN06	TNK2	F6PN06	ENSBTAG00000021364	-0.120517674	-0.091483061	-0.029034613
A0A3Q1MPU3	TRAK1	A0A3Q1MPU3	ENSBTAG00000016076	0.258734268	0.168577318	0.09015695
A6QLN7	FMO5	A6QLN7; bta:788719	ENSBTAG00000010841	0.209539819	0.088403382	0.121136438
A0A3Q1MTU3	RASSF1	A0A3Q1MTU3	ENSBTAG00000020963	-0.051861118	0.281712281	-0.333573399
F1MYN0	BOLA2B	F1MYN0	ENSBTAG00000008632	0.899473124	0.439111634	0.46036149
Q1RMQ5	LIN37	bta:534550; Q1RMQ5	ENSBTAG00000018594	0.272957899	0.190666826	0.082291073
A0A3Q1LLI8	SPMIP6	A0A3Q1LLI8	ENSBTAG00000008376	-0.715345552	-0.491067871	-0.224277681

Accession	Gene_Symbol	Gene_ID	Ensembl_Gene ID	log2(ratio(OV24H/OV0H))	log2(ratio(OV24R/OV0H))	log2(ratio(OV24H/OV24R))
E1B842	HIVEP2	bta:540396; E1B842	ENSBTAG00000001146	0.176322773	0.383435502	-0.207112729
A0AAA9TDS3	F7	A0AAA9TDS3	ENSBTAG00000007411	0.40214321	0.420823165	-0.018679955
G5E6T2	ARHGAP23	G5E6T2	ENSBTAG00000016911	-0.081124225	0.078242023	-0.159366248
A0A3Q1MAX3	NDFIP1	A0A3Q1MAX3	ENSBTAG00000047747	0.138940551	0.388882533	-0.249941982
A0AAA9TZP0	RAD51B	A0AAA9TZP0	ENSBTAG00000018971	-0.220415675	0.105501582	-0.325917257
Q3ZBS1	MACIR	bta:538782; Q3ZBS1	ENSBTAG00000055286	0.297560088	0.201942823	0.095617266
F1MX42	PANK2	F1MX42	ENSBTAG00000007920	-0.012490944	-0.110533917	0.098042973
A4Z945	FAM200C	A4Z945; A5D7P2; bta:539846	ENSBTAG00000030282	0.209497523	0.289209522	-0.079711999
A0AAF7A5Q1	RHBDF1	A0AAF7A5Q1	ENSBTAG00000019805	-0.272610665	-0.340710645	0.06809998
A7MB44	SLC9A6	A0A3S5ZP41; A7MB44; bta:533137; F1MH53	ENSBTAG00000009523	-0.257857102	-0.282547295	0.024690193
A0AAA9SQM0	DHX35	A0AAA9SQM0	ENSBTAG00000004039	0.060968017	-0.040599621	0.101567639
A7MB07	ADAMTS1	A7MB07; bta:512171	ENSBTAG00000000706	-0.327343438	0.464782206	-0.792125644
A0A452DIB0	PPP1R35	A0A452DIB0	ENSBTAG00000026243	-0.276389152	-0.292260544	0.015871393
E1BKV9	ERCC4	bta:516371; E1BKV9	ENSBTAG00000021773	0.284590549	0.728376987	-0.443786438
E1BE19	AHDC1	bta:522105; E1BE19	ENSBTAG00000037456	-0.168029779	-0.269115904	0.101086125
A0AAA9SRJ8	GMEB1	A0AAA9SRJ8	ENSBTAG00000001160	0.120103976	0.092118202	0.027985774
A0A3Q1MIB9	ATF6B	A0A3Q1MIB9	ENSBTAG00000008794	-0.056356796	0.227646674	-0.284003471
A0A3Q1MZB9	AMD1	A0A3Q1MZB9	ENSBTAG00000003342	-0.268028595	-0.068598756	-0.19942984
A0A3Q1LZ05	USP54	A0A3Q1LZ05	ENSBTAG00000011108	0.071862217	0.137637485	-0.065775268
A0AAA9S2K0	CCNY	A0AAA9S2K0	ENSBTAG00000005492	-0.018172746	0.322329345	-0.340502091
F1MMS0	BRIP1	F1MMS0	ENSBTAG00000012068	-0.101331422	0.067174302	-0.168505724
F6RXG8	RGCC	F6RXG8	ENSBTAG00000034885	0.243089577	0.186048381	0.057041196
A0AAA9TRQ1	FAM133B	A0AAA9TRQ1	ENSBTAG00000050705	0.410161278	0.177966691	0.232194587
A0AAA9SNQ3	FOXJ3	A0AAA9SNQ3	ENSBTAG00000015887	0.353785939	0.312990713	0.040795226
A0AAA9TQL1	DDR2	A0AAA9TQL1	ENSBTAG00000004885	0.027834208	0.279005363	-0.251171155
A0A3Q1M4A1	BIN3	A0A3Q1M4A1	ENSBTAG00000005470	0.362771954	0.686676924	-0.32390497
A0AAA9S745	CTDSPL	A0AAA9S745	ENSBTAG00000053223	0.078441355	0.150334273	-0.071892918
A0A452DJ67	ATP6V0C	A0A452DJ67	ENSBTAG00000026428	0.214179714	0.362746999	-0.148567286
Q08DK7	SLC25A29	bta:507686; Q08DK7	ENSBTAG00000013666	0.075017229	0.12915848	-0.054141252
A0A3Q1NB49	IGF1R	A0A3Q1NB49	ENSBTAG00000021527	-0.105722401	0.127192016	-0.232914417
F1MGR3	RERE	bta:535394; F1MGR3	ENSBTAG00000018272	-0.628830352	0.227196883	-0.856027235
F6RXK0	PDGFD	F6RXK0	ENSBTAG00000034827	-0.300091368	-0.293731203	-0.006360165
E1B7S7	ZNF24	bta:538969; E1B7S7; G3N356	ENSBTAG00000010255	-0.071997186	-0.410283969	0.338286783
Q08DP3	RELL1	bta:768210; Q08DP3	ENSBTAG00000004329	-0.855429977	-0.290683916	-0.564746061
A0AAA9THB8				-0.133540851	-0.113470933	-0.020069918
O46419	LIPT1	bta:286864; O46419	ENSBTAG00000059194	-0.15565087	0.166277224	-0.321928095
A0AAF6YXB8	ZFAND6	A0AAF6YXB8	ENSBTAG00000012321	-0.274532279	-0.12388129	-0.150650989
B1VKB3	C/EBP-delta	B1VKB3		-0.081955596	-0.109567827	0.027612231
A0AAA9SGZ0	GLCCI1	A0AAA9SGZ0	ENSBTAG00000008533	0.134506026	-0.068809158	0.203315184
A0AAF6DM43	CENPT	A0AAF6DM43	ENSBTAG00000006404	-0.321273663	-0.265130585	-0.056143078
Q17QE5	CIB1	bta:510141; Q17QE5	ENSBTAG00000021275	0.253149817	0.15160005	0.101549767
A2VDK9	VMA21	A2VDK9; bta:613674	ENSBTAG00000006296	0.12311902	0.009607328	0.113511693
A0AAA9TEG6	PDSS2	A0AAA9TEG6	ENSBTAG00000021859	-0.218125086	-0.016286476	-0.20183861
A6QNW7	CD5L	A6QNW7; bta:528593; F1N514	ENSBTAG00000022514	0.060019579	0.65763738	-0.597617802
E1BHA0	LZTS1	E1BHA0	ENSBTAG00000000841	-0.15083916	-0.015805703	-0.135033457
A0A3Q1M728				0.322503906	0.348745821	-0.026241914
A0AAF6YI58	RSL24D1	A0AAF6YI58	ENSBTAG00000001342	-0.26363691	0.516078332	-0.779715242
F6QEW8	JTB	F6QEW8	ENSBTAG00000014694	0.241911114	0.063984456	0.177926658
A0AAA9TJF2	CHCHD4	A0AAA9TJF2	ENSBTAG00000015529	0.305844372	0.257157839	0.048686532
Q0VC75	TP53I3	bta:508875; F1N047; Q0VC75		-0.310033015	-0.053004695	-0.25702832
F6PVQ3	SNAPIN	F6PVQ3	ENSBTAG00000012470	0.372051574	0.451460908	-0.079409333
A0AAA9TF92	RHBDD1	A0AAA9TF92	ENSBTAG00000023963	0.225220681	0.433252966	-0.208032285
A0AAA9U1A6	TRUB2	A0AAA9U1A6	ENSBTAG00000004663	-0.467358534	-0.467358534	0
A0A3Q1MUR7	GASK1B	A0A3Q1MUR7	ENSBTAG00000017069	-0.406092066	-0.577008269	0.170916204
A0AAA9U073	ZNF106	A0AAA9U073; bta:788468	ENSBTAG00000004079	0.20511443	0.075079117	0.130035313
A0AAA9SK59	PWWP2A	A0AAA9SK59	ENSBTAG00000010341	0.178890783	0.11876979	0.060120992
A0AAA9SWD1	PIBF1	A0AAA9SWD1	ENSBTAG00000019895	-0.034288699	-0.199550171	0.165261472
F6QPT2	PWWP2B	bta:524314; F6QPT2	ENSBTAG00000019519	0.212399312	0.028509722	0.18388959
E1BIT2	MIEF1	bta:613306; E1BIT2	ENSBTAG00000017439	0.08190656	0	0.08190656
F6RC12	FEZ1	F6RC12	ENSBTAG00000009124	-0.461030173	0.209323819	-0.670353992
A0A3Q1MRQ2				-0.239686345	0.283604045	-0.52329039
E1BK15	CLCN6	bta:520210; E1BK15	ENSBTAG00000020700	0.299189171	0.273632538	0.025556633
A0AAA9TJW2	EIF2AK1	A0AAA9TJW2	ENSBTAG00000007370	0.222884221	0.356627927	-0.133743706
G5E5E4	HDAC10	G5E5E4	ENSBTAG00000011000	-0.099929799	-0.022238514	-0.077691285
A0AAA9STC4	SDHD	A0AAA9STC4	ENSBTAG00000016266	0.202743049	0.019385341	0.183357708
Q08DZ6	RWDD4	A0A3Q1MQ19; bta:509865; Q08DZ6	ENSBTAG00000003081	-0.018308558	0.510930002	-0.52923856
Q2KIW5	CENPU	Q2KIW5		0.141424959	0.14455445	-0.003129492
A0AAA9SLL5	MRPS24	A0AAA9SLL5	ENSBTAG00000007398	-0.182060327	-0.173648087	-0.00841224
E1B8R5	TNKS	bta:535030; E1B8R5	ENSBTAG00000001463	0.331291934	0.152673958	0.178617976
A0A3Q1MFC2	SYF2	A0A3Q1MFC2	ENSBTAG00000001651	0.021908251	-0.01566743	0.037575681
Q0P5H8	HERPUD2	bta:512265; Q0P5H8	ENSBTAG00000015105	-0.079252604	0.078171614	-0.157424218
F6RSI9	DONSON	F6RSI9	ENSBTAG00000035175	0.011172912	-0.028316808	0.03948972
F1N7C7	RHOD	F1N7C7	ENSBTAG00000020600	0.194619529	0.061879448	0.132740081
F1MEM9	TTC19	F1MEM9	ENSBTAG00000013270	0.189257299	0.260112087	-0.070854788
A0A140T8A3	MTG1	A0A140T8A3	ENSBTAG00000020304	-0.476607736	-0.209656645	-0.266951091
A0AAA9S2I6	AGPAT4	A0AAA9S2I6	ENSBTAG00000007668	-0.001811293	0.106397924	-0.108209217
A0AAA9TQM2	SNX33	A0AAA9TQM2	ENSBTAG00000012206	-0.138009821	-0.333044127	0.195034306
F1MJA5	FAM171A1	F1MJA5	ENSBTAG00000008787	-0.063193826	0.041893781	-0.105087607
E1BFC4	SLC9B2	E1BFC4	ENSBTAG00000040088	0.22283368	0.189358975	0.033474705
A0A3Q1LWL2	PPP1R13B	A0A3Q1LWL2	ENSBTAG00000003559	0.15935576	0.63441803	-0.47506227
A0AAA9SPJ1	C18H19orf33	A0AAA9SPJ1	ENSBTAG00000063903	-0.09794505	-0.090512293	-0.007432758
F1MW44	F13A1	F1MW44	ENSBTAG00000007268	0.129519079	-0.122379417	0.251898496
E1BBP0	CHFR	E1BBP0	ENSBTAG00000013951	-0.117283522	0.04548775	-0.162771271
A0JN69	MARCHF3	A0JN69; bta:520348	ENSBTAG00000006797	-0.065692541	-0.090920638	0.025228097
G3X8E4	BCOR	bta:784529; G3X8E4	ENSBTAG00000047339	-0.313374542	-0.168138824	-0.145235718
A0AAA9S540	ABHD4	A0AAA9S540	ENSBTAG00000016658	-0.075142858	-0.027376439	-0.047766419
A0A452DJ18	TMEM134	A0A452DJ18	ENSBTAG00000004786	-0.207824219	0.430058596	-0.637882815
Q08DP2	BORCS5	bta:614947; Q08DP2	ENSBTAG00000008989	0.115477217	0.232660757	-0.117183539
F1N4V1	NOS1AP	F1N4V1	ENSBTAG00000010158	0.090602549	0.319789184	-0.229186635
Q17R00	EMX2	bta:523601; Q17R00	ENSBTAG00000003027	-0.33195846	0.076774462	-0.408732922
F1MDF3	ALS2	bta:535750; F1MDF3	ENSBTAG00000007395	-0.010647244	0.006614844	-0.017262089
Q5E9A9	DDA1	bta:512898; Q5E9A9	ENSBTAG00000017068	-0.294779356	-0.152604718	-0.142174638
A0AAA9S081	SH2D3C	A0AAA9S081	ENSBTAG00000015375	0.527838102	0.617426896	-0.089588794
A0A3Q1MQ77	ADGRL2	A0A3Q1MQ77	ENSBTAG00000012305	0	0.039611856	-0.039611856
A0AAA9SMX8	CENPO	A0AAA9SMX8	ENSBTAG00000015014	0.222392421	0.399696953	-0.177304532
A0AAA9T0Y5	POT1	A0AAA9T0Y5	ENSBTAG00000013180	-0.010147955	0.461881017	-0.472028972
A0A3Q1LWX2	RUSC2	A0A3Q1LWX2	ENSBTAG00000011403	0.397130422	0.325719674	0.071410747
E1BMZ2	MECOM	E1BMZ2	ENSBTAG00000005871	-0.095127344	0.16743873	-0.262566074
A0AAA9TZ96	STK11IP	A0AAA9TZ96	ENSBTAG00000038992	-0.176937107	-0.034532406	-0.142404701
A7MB97	YIPF2	A7MB97; bta:515893	ENSBTAG00000025606	0.056689418	0.313090732	-0.256401314
A8YXY8	AKIRIN2	A8YXY8; bta:614292	ENSBTAG00000003764	-0.555379389	-0.400170118	-0.155209271
Q29RJ0	KCTD18	bta:613694; Q29RJ0	ENSBTAG00000002272	-0.103672517	0.272641157	-0.376313674
Q5E9V1	DCUN1D3	bta:504926; Q5E9V1	ENSBTAG00000032260	0.280107919	0.321928095	-0.041820176
A0AAA9T6D8	UNC5B	A0AAA9T6D8	ENSBTAG00000014270	-0.302991913	-0.518681198	0.215689286
A0A3Q1M7M7	FGFR1OP2	A0A3Q1M7M7	ENSBTAG00000004532	-0.066135102	-0.004414165	-0.061720937
A0A3Q1N2B6				0.378511623	0.213109181	0.165402443
F6Q447	SERPINB9	F6Q447	ENSBTAG00000031267	0.157306375	0.27906577	-0.121759395
A0AAA9S4A6	ELOVL5	A0AAA9S4A6	ENSBTAG00000003359	-0.069388873	-0.112017054	0.042628182
Q3SZM3	CYBC1	bta:618343; Q3SZM3	ENSBTAG00000018176	0.009201731	-0.052369739	0.06157147
Q05B57	YOD1	bta:539931; Q05B57		0.087206946	-0.065251185	0.152458131
A0AAA9SKL1	ITPKB	A0AAA9SKL1	ENSBTAG00000053007	-0.255890625	-0.268357583	0.012466957
E1BIN3	ARSI	bta:540390; E1BIN3	ENSBTAG00000012834	-0.531884147	0.624564851	-1.156448998
Q56JY0	HIKESHI	bta:504867; Q56JY0	ENSBTAG00000019995	0.147403215	0.135919015	0.0114842
A0AAA9TTE3	REEP6	A0AAA9TTE3	ENSBTAG00000002311	0.345927414	0.774501668	-0.428574254
A0A3Q1M5N5	SPRYD3	A0A3Q1M5N5	ENSBTAG00000021464	-0.023526484	0.013661987	-0.037188471
Q0VC73	DNAAF2	bta:534465; Q0VC73	ENSBTAG00000004930	0.032838613	-0.054447784	0.087286397
A2VE01	CLP1	A2VE01; bta:506507	ENSBTAG00000006493	-0.029458093	0.02341418	-0.052872273
A0AAA9SS87	POC5	A0AAA9SS87	ENSBTAG00000007114	-0.397019664	-0.060837586	-0.336182079
A0AAA9S8B7	C1D	A0AAA9S8B7	ENSBTAG00000049002	-0.11626407	-0.057779715	-0.058484356
A0A3Q1MSE3	WDR20	A0A3Q1MSE3	ENSBTAG00000007007	0.140262024	0.149875448	-0.009613425
A0AAA9SRU6	CCDC102A	A0AAA9SRU6	ENSBTAG00000000462	-0.135673522	-0.160310801	0.024637279
A0A3Q1MAE0	CLOCK	A0A3Q1MAE0; bta:100300400	ENSBTAG00000044044	0.004283116	-0.053897956	0.058181071
F1MGL5	TMEM67	F1MGL5	ENSBTAG00000044190	0.530790408	0.467392904	0.063397504
A0A3Q1MED0	FERRY3	A0A3Q1MED0	ENSBTAG00000053303	-0.135334057	-0.116382126	-0.018951932
A0AAA9S753	RBX1	A0AAA9S753	ENSBTAG00000053583	0.032823847	-0.135995217	0.168819064
A0AAA9S0E6	TK2	A0AAA9S0E6	ENSBTAG00000005152	-0.715418805	0.131772351	-0.847191157
F1MSU8	PLEKHG2	F1MSU8	ENSBTAG00000004017	-0.379098681	-0.280275626	-0.098823056
A0AAA9T6U6	GABPB1	A0AAA9T6U6	ENSBTAG00000022801	-0.019984374	-0.059211779	0.039227404
A0A3Q1N888	KATNIP	A0A3Q1N888	ENSBTAG00000006129	-0.015580011	0.626206989	-0.641787
A0AAA9S0Y3	RIOX2	A0AAA9S0Y3	ENSBTAG00000015546	-0.124051346	0.154280948	-0.278332294
A0AAA9S3W1	TRAPPC1	A0AAA9S3W1	ENSBTAG00000047214	-0.328142153	-0.149402678	-0.178739476
A0AAF6ZG22	FASTKD3	A0AAF6ZG22	ENSBTAG00000009400	0.043471503	0.119685373	-0.07621387
A0AAA9RWA7	DENND6A	A0AAA9RWA7	ENSBTAG00000018433	-0.398294235	0.276073701	-0.674367936
A6QLS5	OTULINL	A6QLS5; bta:534389; F1N7I7	ENSBTAG00000000672	-0.130219528	-0.239817055	0.109597527
A0A3S5ZPT8	MAD2L1BP	A0A3S5ZPT8	ENSBTAG00000009548	-0.17579559	-0.034086831	-0.141708759
A0AAF6Z5K6				0.220174596	0.161280907	0.058893689
Q3ZCG2	MPC1	bta:767977; Q3ZCG2	ENSBTAG00000027879	-0.046378354	-0.127961708	0.081583354
P00430	COX7C	bta:101902937; bta:101903567; bta:327718; P00430; Q3T0U0; Q56K07	ENSBTAG00000039555	0.138487195	0.069431779	0.069055417
A0AAA9RY04	ATF6	A0AAA9RY04	ENSBTAG00000005227	0.341761617	0.579212413	-0.237450796
A0A3Q1NB78	ZDHHC3	A0A3Q1NB78	ENSBTAG00000011484	0.082711051	-0.079829024	0.162540075
A0AAA9SWS7	LLGL2	A0AAA9SWS7	ENSBTAG00000020067	-0.10583626	0.0489096	-0.154745861
F1ML55	MIER3	F1ML55	ENSBTAG00000014248	0.041177407	-0.012910109	0.054087516
E1BLB1	SHPRH	E1BLB1	ENSBTAG00000008112	-0.135849373	-0.138883433	0.00303406
A0AAA9T977	SOWAHC	A0AAA9T977	ENSBTAG00000068476	0.200186627	0.057804452	0.142382175
F1N293	CNNM3	F1N293	ENSBTAG00000010846	0.409021441	0.644668747	-0.235647306
F1MJ20	ARHGAP28	bta:530550; F1MJ20	ENSBTAG00000019036	0.077691285	0.167294745	-0.089603461
A6QLT2	MTMR2	A6QLT2; bta:536810	ENSBTAG00000016557	0.167619061	-0.131738353	0.299357414
P00741	F9	F1MFL4; P00741		0.022900402	0.484395963	-0.46149556
A2VDU6	AP1AR	A2VDU6; bta:533664	ENSBTAG00000003941	0.070317838	0.211845257	-0.141527418
E1BMP7	DNA2	E1BMP7		-0.004028002	0.256090282	-0.260118283
A0AAA9TZC1	STX16	A0AAA9TZC1; bta:519624	ENSBTAG00000003872	0.045642666	0.408582477	-0.362939812
F1N3Y8	FANCE	F1N3Y8	ENSBTAG00000019492	-0.130113295	0.099908415	-0.23002171
A0AAA9S1M3	ENDOG	A0AAA9S1M3	ENSBTAG00000012484	-0.267412846	-0.330148602	0.062735755
E1B702	TGFBR2	E1B702	ENSBTAG00000019832	-0.373855887	-0.074767768	-0.299088119
A0A3Q1M7N8	SMIM26	A0A3Q1M7N8	ENSBTAG00000051249	0.175798753	0.047506731	0.128292022
Q3SZX4	CA3	bta:513212; Q3SZX4	ENSBTAG00000015214	0.210035215	0.625405094	-0.415369879
F1MUT4	F11	F1MUT4	ENSBTAG00000003572	0.257588454	0.290901199	-0.033312745
Q7YS61	TRDMT1	bta:353353; Q58D29; Q7YS61		-0.018281491	0.142586585	-0.160868077
A0AAA9TCW8	SNAP47	A0AAA9TCW8	ENSBTAG00000004188	0.200511093	0.106402938	0.094108155
A0AAA9T8L1				-0.051275902	0.056260573	-0.107536475
A0AAA9SS54	BTBD2	A0AAA9SS54	ENSBTAG00000016108	0.096079891	-0.032548694	0.128628585
Q1RMU9	FIG4	bta:534118; Q1RMU9		0.053291194	-0.038510829	0.091802023
Q0V8L2	GGT7	A6QR42; bta:615929; Q0V8L2	ENSBTAG00000013301	0.008248708	0.414210029	-0.405961321
A0AAA9TSY3	RBIS	A0AAA9TSY3	ENSBTAG00000032227	-0.601687915	-0.387023123	-0.214664792
E1BG64	FEZ2	bta:100299092; E1BG64	ENSBTAG00000009021	-0.238601401	0.005289447	-0.243890848
A0A3Q1MDQ9	NKPD1	A0A3Q1MDQ9	ENSBTAG00000020074	0.462263795	0.30641687	0.155846925
F1MMI9	TRPT1	F1MMI9	ENSBTAG00000016555	-0.292205514	-0.191778646	-0.100426868
Q1RMK0	RNF115	bta:614061; F6RQU6; Q1RMK0	ENSBTAG00000008131	0.433213425	0.513887101	-0.080673676
F1MSV4	SGK3	F1MSV4	ENSBTAG00000013284	-0.318459481	0.105213243	-0.423672724
A0A3Q1M1J0	GFOD1	A0A3Q1M1J0	ENSBTAG00000049619	-0.214861207	-0.297710045	0.082848838
A0AAA9SBD6	ATPSCKMT	A0AAA9SBD6	ENSBTAG00000017885	-0.015814454	-0.261034036	0.245219582
F1MWF6	ACAP3	F1MWF6	ENSBTAG00000009856	-0.43736271	-0.308950241	-0.128412469
A0A452DI36	ALAS1	A0A452DI36	ENSBTAG00000004118	-0.332112789	-0.403288541	0.071175752
Q2YDK5	POLR1D	Q2YDK5		0.037442284	-0.249192481	0.286634765
A0AAA9T6B4	FAIM	A0AAA9T6B4	ENSBTAG00000023426	0.01759406	0.168285016	-0.150690956
A0A3Q1LRP7	NRF1	A0A3Q1LRP7	ENSBTAG00000014273	0.290121511	0.112221009	0.177900502
A0AAA9RU81				-0.190155526	-0.049425217	-0.140730309
A0AAA9TMX6	ZBTB33	A0AAA9TMX6	ENSBTAG00000017184	0.072585481	-0.165465353	0.238050834
A0A3Q1M7C4	FAM117B	A0A3Q1M7C4	ENSBTAG00000048876	-0.479699314	0.304930491	-0.784629805
A0AAA9T713	STARD10	A0AAA9T713	ENSBTAG00000015182	0.324714716	0.596794261	-0.272079545
A0AAA9SM03	KDM4C	A0AAA9SM03	ENSBTAG00000043987	-0.199071347	-0.062237132	-0.136834215
A0AAF6YZY8	CDC26	A0AAF6YZY8	ENSBTAG00000014430	0.584327092	0.67783366	-0.093506568
Q5E9U1	P2RX4	bta:338036; Q5E9U1	ENSBTAG00000010812	0.079596826	0.040347286	0.03924954
A0JNC4	ELOVL7	A0JNC4; bta:614096	ENSBTAG00000007704	-0.480041898	0.05206872	-0.532110618
F1MKY1	AGGF1	bta:511650; F1MKY1	ENSBTAG00000034823	-0.103983448	0.253346302	-0.357329749
Q4TVR5	DSTYK	bta:534684; Q4TVR5	ENSBTAG00000016618	0.444130411	0.610851687	-0.166721276
A0AAA9T9D5	TICAM1	A0AAA9T9D5	ENSBTAG00000019966	-0.231915195	0.03085379	-0.262768985
A0AAA9TSN9	DCBLD1	A0AAA9TSN9	ENSBTAG00000047338	-0.152368286	0.050059355	-0.202427641
A0A0F7RPX0	MIF	A0A0F7RPX0; bta:280858		0.630022955	-0.073156104	0.703179059

Accession	Gene_Symbol	Gene_ID	Ensembl_Gene ID	log2(ratio(OV24H/OV0H))	log2(ratio(OV24R/OV0H))	log2(ratio(OV24H/OV24R))
A0AAA9TBL0				-0.229211207	-0.302824436	0.073613229
E1BNA6	DOCK8	E1BNA6	ENSBTAG00000002190	-0.263315935	-0.265006263	0.001690328
A0A3Q1LRZ5	PLEKHA7	A0A3Q1LRZ5	ENSBTAG00000006974	0.019973024	0.141641857	-0.121668833
E1BBH2	TRAF3	bta:506182; E1BBH2	ENSBTAG00000069778	0.10744629	-0.058816227	0.166262517
F6S165	RTN2	F6S165	ENSBTAG00000008185	0.11417102	0.50589093	-0.39171991
G5E6P9	TADA2B	bta:515581; G5E6P9	ENSBTAG00000010185; ENSBTAG00000066252	-0.257193527	0.121990524	-0.379184052
A0A452DJB5	SLC35C1	A0A452DJB5	ENSBTAG00000003199	0.153679671	0.035896657	0.117783015
A0AAA9T467	COX14	A0AAA9T467	ENSBTAG00000000808	0.153717711	0.141043881	0.01267383
Q2HJ44	TENT2	bta:533862; Q1JPF9; Q2HJ44	ENSBTAG00000006751	-0.469929258	0.451013243	-0.920942501
A0A3Q1M082	ATP8B2	A0A3Q1M082	ENSBTAG00000005161	0.413670664	0.468675463	-0.055004799
O97827	ADGRL3	bta:282651; O97818; O97819; O97820; O97821; O97822; O97823; O97824; O97825; O97826; O97827; O97828; O97829	ENSBTAG00000013918	-0.03507851	-0.029827567	-0.005250943
E1BI54	HOXC8	bta:100295249; E1BI54	ENSBTAG00000012149	0.035657776	0.1300366	-0.094378824
E1BK01	PRSS12	E1BK01	ENSBTAG00000020705	-0.232660757	-0.236673819	0.004013063
A0AAA9S1N2	CAMKK2	A0AAA9S1N2	ENSBTAG00000010815	0.15494983	0.273942884	-0.118993054
A0AAA9SZN1	IRF3	A0AAA9SZN1	ENSBTAG00000006633	0.177998302	-0.026967048	0.20496535
A5D7G9	SGK196	A5D7G9; bta:514490		-0.376873128	-0.230612928	-0.1462602
Q5EA04	TXNL4B	bta:510719; Q5EA04	ENSBTAG00000006353	0.182124365	-0.039355824	0.221480189
A0AAF6DM00	NKAPD1	A0AAF6DM00	ENSBTAG00000001999	0.272105396	0.484481108	-0.212375711
A0A2H4GTG0				0.588656984	0.066861954	0.52179503
E1BCU9	STK11	E1BCU9	ENSBTAG00000011639	-0.289644813	-0.195490631	-0.094154182
A0AAA9TIB9	LRRC20	A0AAA9TIB9	ENSBTAG00000005666	0.065648042	-0.289058923	0.354706965
A0AAA9RW64	GDE1	A0AAA9RW64	ENSBTAG00000002101	-0.243443677	0.056637715	-0.300081393
A5D7N7	TMCO3	A5D7N7; bta:509611	ENSBTAG00000015704	-0.058141034	-0.053806444	-0.00433459
E1B8K5	COQ8B	E1B8K5	ENSBTAG00000019462	-0.268945997	-0.04493892	-0.224007077
A0AAA9RWM9	DHRS3	A0AAA9RWM9	ENSBTAG00000024493	0.12963528	0.312526173	-0.182890893
A0AAA9T6U9	USP53	A0AAA9T6U9	ENSBTAG00000010350	0.198497804	0.208683288	-0.010185484
Q2T9W7	MOSPD1	bta:523578; Q2T9W7	ENSBTAG00000013922	-0.048655806	0.270986529	-0.319642335
A0AAF6DMI7	BCL7C	A0AAF6DMI7	ENSBTAG00000008412	-0.123406975	-0.063255409	-0.060151566
A0A172W434	ND4	A0A172W434		-0.067904065	-0.075826627	0.007922563
F1N3E8	POLR3K	F1N3E8	ENSBTAG00000019803	0.152667012	0.26607486	-0.113407848
A0AAF7AFF3	BCHE	A0AAF7AFF3	ENSBTAG00000011139	-0.230723696	-0.131838031	-0.098885665
Q2HJC0	NGRN	bta:508115; Q2HJC0		-0.353636955	-0.250543462	-0.103093493
A0AAA9STI8	FBXO5	A0AAA9STI8	ENSBTAG00000021193	-0.1859073	-0.37168113	0.18577383
Q0P563	FUOM	bta:613571; Q0P563		0.202720445	0.241799764	-0.039079319
A0AAA9S762	ATP5MJ	A0AAA9S762	ENSBTAG00000026886	-0.016103308	-0.100992206	0.084888898
Q3MHJ7	EEPD1	bta:511767; Q3MHJ7; Q6Q139	ENSBTAG00000019065	-0.242291979	0.004228713	-0.246520692
A0A3Q1NDB9	BBS7	A0A3Q1NDB9	ENSBTAG00000004945	0.299006429	0.223868324	0.075138104
A0AAA9T8G7				0.218515739	0.33177805	-0.113262311
E1BIN1	STIL	E1BIN1	ENSBTAG00000017844	0.047798339	-0.246192879	0.293991218
Q3SZR9	TSPAN3	bta:616881; Q3SZR9	ENSBTAG00000002415	-0.097878649	0.09834484	-0.196223488
F1MGJ6	NRBP2	bta:504664; F1MGJ6	ENSBTAG00000008079	-0.32443495	-0.315679947	-0.008755003
F1MVD6	CCDC88B	F1MVD6	ENSBTAG00000040190	-0.545807312	-0.005763871	-0.54004344
A0AAA9S2H2	TSPAN18	A0AAA9S2H2	ENSBTAG00000021484	-0.169454838	-0.272469986	0.103015147
Q5E985	HYAL1	bta:515397; Q3T0A8; Q5E985; Q5E9Z4		0.118760832	0.080250003	0.038510829
A0AAA9SQR3	FAM3A	A0AAA9SQR3	ENSBTAG00000012747	-0.289365887	-0.034610959	-0.254754928
F1N1H5	MCM10	F1N1H5	ENSBTAG00000016406	0.092193698	0.090819048	0.00137465
A0AAA9SB31	ARL5B	A0AAA9SB31	ENSBTAG00000043964	-0.107013157	-0.094723205	-0.012289952
F6QUB2	RNFT1	F6QUB2	ENSBTAG00000009747	-0.231379902	-0.189433934	-0.041945968
E1BA57	DEPDC1	E1BA57	ENSBTAG00000001343	0.397335498	0.304056454	0.093279043
A0A452DJ16	FAM131B	A0A452DJ16	ENSBTAG00000009418	0.207466827	0.15864	0.048826827
A0AAA9S0B6	CEP104	A0AAA9S0B6	ENSBTAG00000020014	0.215062104	0.350434523	-0.135372419
E1BND5	NCOA6	bta:508899; E1BND5	ENSBTAG00000007930	-0.197392976	-0.110288257	-0.087104719
A0AAA9TS17	TNFRSF12A	A0AAA9TS17	ENSBTAG00000012082	0.002266607	-0.754462242	0.756728849
A0AAA9T627	NAA38	A0AAA9T627	ENSBTAG00000014695	0.188680142	-0.169385172	0.358065314
F1MCL6	NADK	F1MCL6	ENSBTAG00000000212	0.290590128	0.290590128	0
A0AAA9TZN6	CSDC2	A0AAA9TZN6	ENSBTAG00000004004	-0.266457796	0.212400505	-0.478858301
A0AAA9SAI9	ARHGAP24	A0AAA9SAI9	ENSBTAG00000003959	-0.097324907	0.078817399	-0.176142306
A0AAA9T0W8	PIAS4	A0AAA9T0W8	ENSBTAG00000000753	-0.123800952	0.25569243	-0.379493382
A0AAA9RVU8	EEF1AKMT1	A0AAA9RVU8	ENSBTAG00000008296	-0.167816391	-0.048864823	-0.118951567
F1MB67	SLC27A1	F1MB67	ENSBTAG00000016775	-0.271142709	-0.211078612	-0.060064097
A0AAA9T921	TSEN2	A0AAA9T921	ENSBTAG00000037980	-0.463635373	-0.465417578	0.001782205
E1BPV8	SLCO4A1	E1BPV8	ENSBTAG00000016388	0.042248848	0.046406473	-0.004157625
A0A0S1S2N1	ND2	A0A0S1S2N1		-0.246384065	-0.282659452	0.036275386
A0AAA9TPS8	CCDC28B	A0AAA9TPS8	ENSBTAG00000015104	0.005799787	0.150258601	-0.144458814
A0AAA9SZH6	TEX30	A0AAA9SZH6	ENSBTAG00000025221	0.026472211	0.666125354	-0.639653142
A0A3Q1MS15	FNIP1	A0A3Q1MS15	ENSBTAG00000025443	-0.363368361	-0.08336613	-0.280002231
F1MC61	MAP3K11	F1MC61	ENSBTAG00000004114	-0.496902147	-0.156808783	-0.340093363
A0AAF6Z6T7	PTOV1	A0AAF6Z6T7	ENSBTAG00000030578	-0.468523935	-0.382893864	-0.085630072
A0A3Q1M8G4				0.356343505	0.128324097	0.228019408
A5PJK0	SERPINB10	A5PJK0; bta:510205		-0.458873408	-0.465025635	0.006152227
A0A3Q1MJP5	C25H7orf50	A0A3Q1MJP5; bta:522840	ENSBTAG00000051685	-0.540568381	-0.483601786	-0.056966596
F6PVN0	MTRES1	F6PVN0	ENSBTAG00000021398	0.318092838	0.098684275	0.219408563
A0A3Q1LP90	BMPR2	A0A3Q1LP90; bta:407127	ENSBTAG00000006420	-0.258509483	-0.412521901	0.154012418
A5D7L6	MRAS	A5D7L6; bta:540803	ENSBTAG00000001497	0.568711346	-0.044132937	0.612844282
A0AAA9S2L3	SLC35E1	A0AAA9S2L3; bta:533774	ENSBTAG00000005295	0.062865954	0.07308235	-0.010216396
Q32L09	SHFL	bta:539087; Q32L09	ENSBTAG00000015636	0.001894544	0.373691193	-0.371796649
F1MWY6	EI24	F1MWY6	ENSBTAG00000007202	0.257863995	0.21926632	0.038597675
Q5E9F6	VPS72	bta:534883; Q2TBU7; Q5E9F6	ENSBTAG00000018074	0.013296823	0.023554728	-0.010257905
A0A3Q1LXS5	GRAMD1B	A0A3Q1LXS5	ENSBTAG00000001410	-0.813322234	-0.459927953	-0.353394281
A0AAA9RXT8	ADNP2	A0AAA9RXT8	ENSBTAG00000005916	0.061927749	0.131611305	-0.069683555
A0AAA9TSX0	RCCD1	A0AAA9TSX0	ENSBTAG00000018642	0.350860387	0.087825981	0.263034406
A0AAA9TRN0	ZNF668	A0AAA9TRN0	ENSBTAG00000006650	-0.068104446	-0.161116996	0.09301255
Q32LB3	CXXC5	bta:538485; Q32LB3	ENSBTAG00000003986	-0.343511744	-0.477773255	0.134261511
A0AAA9SYW5	NEK3	A0AAA9SYW5	ENSBTAG00000020612	-0.157085994	1.116127578	-1.273213572
A0A3Q1M6J2	TSPAN15	A0A3Q1M6J2	ENSBTAG00000000224	0.015955106	0.245652328	-0.229697222
A0AAA9T328	PHYHD1	A0AAA9T328	ENSBTAG00000038831	-0.388040767	0.391401135	-0.779441902
Q4U0T9	CSRP3	bta:540407; Q4U0T9	ENSBTAG00000011869	-0.03922131	0.208108195	-0.247329505
A0AAA9TNQ2	RIT1	A0AAA9TNQ2	ENSBTAG00000000105	0.22929198	0.321282449	-0.091990469
A6QQS9	NBR1	A6QQS9; bta:515032	ENSBTAG00000001632	-0.128986441	-0.397070953	0.268084512
A5PJW1	TAF10	A5PJW1; bta:514443	ENSBTAG00000000694	0.128713045	0.381831982	-0.253118937
A0A3Q1M028	TSPO	A0A3Q1M028	ENSBTAG00000018073	0.141000316	0.178130546	-0.037130231
A0A3Q1MQA6	PHAF1	A0A3Q1MQA6	ENSBTAG00000005058	-0.408464845	-0.008810379	-0.399654467
G3X6S6	NHS	G3X6S6	ENSBTAG00000039195	-0.117195735	-0.015941544	-0.101254191
Q58CR1	MTFR2	bta:534254; Q58CR1	ENSBTAG00000007799	0.157889937	0.145753629	0.012136307
F1MV04	ZFP91	F1MV04	ENSBTAG00000024657	0.111629727	-0.023699835	0.135329562
E1B9V4	TTF1	E1B9V4	ENSBTAG00000018710	0.186048381	0.05316571	0.132882671
A0AAA9TUZ8	STX11	A0AAA9TUZ8	ENSBTAG00000011236	-0.327645321	0.354002505	-0.681647826
A0AAA9TZI7	CDSN	A0AAA9TZI7	ENSBTAG00000021721	-0.791703922	-0.228700674	-0.563003248
F1MQB7	PSTK	F1MQB7	ENSBTAG00000014028	-0.079887772	0.595755196	-0.675642968
A0AAF7AKA5	PPT2	A0AAF7AKA5	ENSBTAG00000004436	0.058475889	0.068469858	-0.009993969
E1BCI6	RECK	bta:517232; E1BCI6	ENSBTAG00000000076	-0.191190015	0.6237431	-0.814933114
A0A0B5H0C3	ND1	A0A0B5H0C3		-0.118850817	0.010073722	-0.128924539
F1MCM3	BLTP3A	bta:534225; F1MCM3	ENSBTAG00000009267	-0.646639397	-0.182692298	-0.4639471
A0AAA9SMJ0	FGD6	A0AAA9SMJ0	ENSBTAG00000014030	0.024247546	-0.09474049	0.118988036
A0AAA9SB64	MOCS1	A0AAA9SB64	ENSBTAG00000010449	-0.405328288	-0.233059346	-0.172268942
F1N5V1	USP37	bta:407168; F1N5V1	ENSBTAG00000016572	0.243758159	0.394647386	-0.150889227
Q05KJ0	BCL2L1	bta:282152; Q05KJ0	ENSBTAG00000006526	0.140990101	-0.302517058	0.443507159
A5PJU7	RMI2	A5PJU7; bta:615553		-0.155495384	0.216335184	-0.371830568
Q2KIT0				0.154818109	0.406559345	-0.251741236
A0AAA9SY52	FAAH	A0AAA9SY52	ENSBTAG00000007507	-0.369286633	-0.124378846	-0.244907788
A0A3Q1M6W9	ZNF385A	A0A3Q1M6W9	ENSBTAG00000015604	-0.057559889	0.077199632	-0.134759521
Q3SYZ0	BAD	bta:615013; Q3SYZ0		-0.002853997	0.169291268	-0.172145265
G3N319	ATXN1L	bta:521025; G3N319	ENSBTAG00000046255	0.238760818	0.288668328	-0.04990751
A0AAA9T3V1				-0.110161335	-0.078229155	-0.03193218
A5PJD1	PHLDB3	A5PJD1; bta:533145		-0.026499225	-0.134443415	0.10794419
A0AAA9S7U1	DNAL4	A0AAA9S7U1	ENSBTAG00000049822	0.296064333	0.393099668	-0.097035335
F1MNI8	ARL13B	F1MNI8	ENSBTAG00000005155	-0.171054565	-0.129524992	-0.041529573
E1BCF7	DLX2	bta:528490; E1BCF7	ENSBTAG00000005741	0.429829607	0.428404723	0.001424884
F1MF11	PLCH1	F1MF11	ENSBTAG00000008307	0.078465434	-0.093891565	0.172356998
E1BBW7	RETREG3	bta:789163; E1BBW7	ENSBTAG00000032493	0.528161601	0.15627594	0.371885661
A0A3Q1N8B0	FADS1	A0A3Q1N8B0; bta:533107	ENSBTAG00000022294	-0.33875614	-0.049070607	-0.289685534
F1N3J7	SLC26A2	F1N3J7	ENSBTAG00000014615	0.092566119	0.201309939	-0.10874382
A0AAA9S9F1	YIPF4	A0AAA9S9F1	ENSBTAG00000033041	0.180669009	0.064344891	0.116324118
F1MJQ8	RNPC3	F1MJQ8	ENSBTAG00000019091	0.238590778	0.232977172	0.005613606
E1B7B3	CSGALNACT2	E1B7B3	ENSBTAG00000005165	0.151178461	0.036628922	0.114549539
P05632	ATP5F1E	bta:617230; P05632; Q32PE5	ENSBTAG00000039208	-0.137256508	-0.25318662	0.115930112
Q2NL14	KANSL2	bta:540194; Q2NL14; Q58D11		0.085860737	0.405221758	-0.319361021
A0AAA9SWH7	DGKH	A0AAA9SWH7	ENSBTAG00000013879	-0.167670384	0.109590624	-0.277261007
E1BI93	CBARP	E1BI93	ENSBTAG00000025233	-0.149983926	0.626782676	-0.776766602
F6QTR5	COQ10A	F6QTR5	ENSBTAG00000004367	-0.070676746	-0.004317295	-0.066359451
A5D7P9	LYVE-1	A5D7P9; bta:404179		0.152157384	0.382659856	-0.230502472
Q3B7N6	ERCC5	bta:509602; Q3B7N6	ENSBTAG00000014043	-0.261187672	-0.08285043	-0.178337241
G3X746	CABIN1	G3X746	ENSBTAG00000024708	0.084670324	0.051100791	0.033569533
F1MX93	MYO5C	F1MX93	ENSBTAG00000003763	-0.074853995	0.832605599	-0.907459594
A0AAF6Z840	COQ6	A0AAF6Z840	ENSBTAG00000020331	-0.046470039	0.050727804	-0.097197843
A0AAA9TVB0	PRR5L	A0AAA9TVB0	ENSBTAG00000021029	-0.319908927	-0.182099715	-0.137809212
E1BAA5	TRMT44	E1BAA5	ENSBTAG00000004797	0.277147142	0.241603025	0.035544117
A0AAF6Z8G7	USP33	A0AAF6Z8G7	ENSBTAG00000020761	-0.072574602	0.060459022	-0.133033624
F1MPW8	ZFHX4	bta:539762; F1MPW8	ENSBTAG00000033268	0.40554605	0.051115316	0.354430735
Q08DN3	CDK5RAP1	bta:514086; Q08DN3	ENSBTAG00000018535	-0.411518729	-0.185866545	-0.225652184
Q32LJ0	SVBP	bta:614073; Q32LJ0	ENSBTAG00000017388	0.047021668	-0.30411489	0.351136557
A0A3Q1ME34	REXO1	A0A3Q1ME34	ENSBTAG00000020232	0.361313923	-0.146572354	0.507886278
A0A452DIR3	IFT57	A0A452DIR3	ENSBTAG00000005787	-0.115796999	-0.048115966	-0.067681033
A0AAA9S2N1	ZER1	A0AAA9S2N1	ENSBTAG00000010208	-0.011438673	-0.031677212	0.020238539
A0A3Q1LXV0	TSPAN9	A0A3Q1LXV0	ENSBTAG00000049163	-0.030131971	0.198667386	-0.228799358
A0AAA9TJ11	NQO1	A0AAA9TJ11	ENSBTAG00000020632	0.20179346	0.196997794	0.004795667
A0AAF6Z4S9	C16H1orf116	A0AAF6Z4S9	ENSBTAG00000018143	-0.240555525	-0.197097569	-0.043457956
E1BCU1	UBE3B	bta:512750; E1BCU1	ENSBTAG00000002698	0.060720381	0.430055138	-0.369334757
A0AAA9T2M8	RFX7	A0AAA9T2M8; bta:541058	ENSBTAG00000000565	-0.207028996	-0.260031918	0.053002922
F1MVH6	B3GALT6	bta:522406; F1MVH6	ENSBTAG00000037523	0.085703749	-0.034367872	0.120071621
A0A3Q1LF99	ANKRD11	A0A3Q1LF99; bta:532856	ENSBTAG00000016006	-0.749857677	-0.273419633	-0.476438044
E1BAS6	SETX	E1BAS6	ENSBTAG00000024822	-0.105860902	0.218687893	-0.324548795
A0AAA9RXN7	CEP76	A0AAA9RXN7	ENSBTAG00000010547	-0.372529841	0.181533562	-0.554063403
A0AAA9T1Y9	SAT2	A0AAA9T1Y9	ENSBTAG00000004222	0.045737679	0.37542112	-0.329683441
G5E699	TMEM131L	G5E699	ENSBTAG00000016625	-0.275883828	-0.563797891	0.287914063
E1B7A6	FLVCR1	bta:533317; E1B7A6	ENSBTAG00000015974	-0.831646017	-0.400126891	-0.431519127
E1U8D1	WWOX	E1U8D1		-0.602054403	-0.239137021	-0.362917382
F1MVI6	TCF3	F1MVI6	ENSBTAG00000008695	0.039567084	-0.246227224	0.285794309
A1A4K7	FAM210B	A1A4K7; bta:516545	ENSBTAG00000013007	0.007905198	-0.014606333	0.022511531
E1BHM7	RNF216	E1BHM7	ENSBTAG00000017175	-0.051087104	0.090328175	-0.141415279
A2VEA7	DAPL1	A2VEA7; bta:574085; Q4PP00	ENSBTAG00000032481	0.874279576	-0.078176257	0.952455834
E1BHJ3	FRMD4A	bta:510714; E1BHJ3	ENSBTAG00000008279	-0.092230873	0.010216396	-0.102447269
A0A3Q1MVB0	CDC6	A0A3Q1MVB0	ENSBTAG00000010384	-0.106160459	-0.025459985	-0.080700474
E1BKB4	TTLL4	E1BKB4	ENSBTAG00000018415	-0.125219386	-0.043484155	-0.081735231
A0AAA9TK30	TTC28	A0AAA9TK30	ENSBTAG00000012193	-0.395408693	-0.165608321	-0.229800371
A0AAF7A190	HYAL2	A0AAF7A190	ENSBTAG00000000484	-0.477103563	-0.349315195	-0.127788369
F1MI51	SIMC1	bta:100336733; F1MI51	ENSBTAG00000034998	-0.106069594	-0.237314127	0.131244533
A0AAA9TAS5	NOL12	A0AAA9TAS5	ENSBTAG00000060113	0.143965774	0.139121802	0.004843972
E1BGL2	GARRE1	E1BGL2	ENSBTAG00000013175	-0.075381812	-0.042591877	-0.032789935
A0A3Q1LPL5	PHF11	A0A3Q1LPL5	ENSBTAG00000013159	-0.340875008	-0.198904068	-0.14197094
F1MFH5	KLHL42	bta:781458; F1MFH5	ENSBTAG00000006372	-0.265627698	-0.252941485	-0.012686213
A0AAA9RZ85	KDELR3	A0AAA9RZ85	ENSBTAG00000009886	-0.048806178	-0.061270143	0.012463966
A0AAA9S2A5	DCHS1	A0AAA9S2A5	ENSBTAG00000015405	-0.212993723	0.035919574	-0.248913298
Q2NKR1	TIMM10	bta:512818; Q2NKR1	ENSBTAG00000024701	0.398114196	0.457518483	-0.059404287
A0A3Q1NMK6	HIRA	A0A3Q1NMK6	ENSBTAG00000019888	-0.077598995	-0.301013879	0.223414884
F1MBX3	ESRRA	bta:507834; F1MBX3	ENSBTAG00000008645	-0.566256306	-0.040574743	-0.525681563
A0A3Q1MG28	RESTB	A0A3Q1MG28	ENSBTAG00000011789	-0.160928065	0.062931256	-0.22385932
Q0VCP2	PEX11A	bta:515608; Q0VCP2	ENSBTAG00000006140	-0.377651083	-0.110893592	-0.266757492
A0A3Q1MCX7	MPPE1	A0A3Q1MCX7; bta:506830	ENSBTAG00000002128	0.647662687	0.446455596	0.201207091
A0AAF6Z257	IRAK1	A0AAF6Z257	ENSBTAG00000016085	0.507094744	0.473421698	0.033673047
E1BBM5	RNF167	bta:616030; E1BBM5	ENSBTAG00000004913	0.256013978	0.248664522	0.007349456
Q3T104	SEC61G	bta:615778; Q3T104	ENSBTAG00000014971	0.112187147	0.134812207	-0.022625059
Q08D85	PXDC1	bta:613986; Q08D85	ENSBTAG00000026919	-0.099535674	0.125530882	-0.225066556
Q3B7L5	FLCN	bta:526652; Q3B7L5	ENSBTAG00000008010	-0.17907022	-0.201766468	0.022696247
Q3T0Z8	SNRPF	bta:615240; Q3T0Z8	ENSBTAG00000016271	0.170431655	0.034548227	0.135883428
G5E568	UBN2	bta:540792; G5E568	ENSBTAG00000014021	-0.370858051	0.092592799	-0.463450849
A0A3Q1MFM1	PLEKHM1	A0A3Q1MFM1	ENSBTAG00000020359	-0.21551345	-0.106607254	-0.108906196
A0A3Q1MBZ8	KDM2B	A0A3Q1MBZ8	ENSBTAG00000002328	0.023411387	0.036415236	-0.013003849
A7Z056	USP20	A7Z056; bta:505839	ENSBTAG00000020311	0.059118216	0.01257563	0.046542586

Accession	Gene_Symbol	Gene_ID	Ensembl_Gene ID	log2(ratio(OV24H/OV0H))	log2(ratio(OV24R/OV0H))	log2(ratio(OV24H/OV24R))
Q58DI5	PLPP6	A6QLK7; bta:541021; Q58DI5	ENSBTAG00000011050	-0.070222165	0.607630335	-0.6778525
Q3SZI3	C7H5orf15	bta:514781; Q3SZI3	ENSBTAG00000013112	0.100430091	0.068790774	0.031639317
F1MJS7	WDR24	A0A8J8YNK6; F1MJS7; M5FJX7	ENSBTAG00000019006	0.345344759	0.457045026	-0.111700266
A0AAF6DLL3	CCP110	A0AAF6DLL3; bta:538197	ENSBTAG00000003960	0.507021975	0.249864135	0.257157839
E1B949	FAT4	bta:781683; E1B949	ENSBTAG00000003345	-0.282815617	-0.234674123	-0.048141494
A0A3Q1LLQ6	TCF4	A0A3Q1LLQ6	ENSBTAG00000016462	-0.283135612	-0.360627055	0.077491444
A2VDS3	TLCD3A	A2VDS3; bta:508425	ENSBTAG00000046273	0.09425759	0.083890874	0.010366716
A0AAA9S548	PCMTD2	A0AAA9S548	ENSBTAG00000031715	-1.092144714	-0.253895784	-0.83824893
A0A7U3JWD0	FGF2B	A0A7U3JWD0; bta:281160		-0.12788887	-0.164535772	0.036646902
A0A3Q1LH17	DROSHA	A0A3Q1LH17	ENSBTAG00000017551	-0.019011969	-0.138666284	0.119654316
F1N5D1	CASB	bta:514494; F1N5D1	ENSBTAG00000020418	-0.49181327	-0.38310701	-0.108706259
E1BD39	ORAI2	bta:511233; E1BD39	ENSBTAG00000012050	0.094154182	-0.041641337	0.135795518
A0A3Q1ME24	RNF5	A0A3Q1ME24	ENSBTAG00000025413	0.016286476	0.048318024	-0.032031548
E1BJZ9	TRAPPC14	E1BJZ9	ENSBTAG00000015011	-0.462744353	-0.230436462	-0.23230789
A0AAA9SUI3	GTF2A2	A0AAA9SUI3	ENSBTAG00000010298	-0.063627437	-0.238620883	0.174993446
A0AAA9SGC7	ZBTB7B	A0AAA9SGC7	ENSBTAG00000021512	-0.101845352	0.053771256	-0.155616608
E1BIS6	SYNM	E1BIS6	ENSBTAG00000011752	-0.018450852	-0.111369814	0.092918962
A0AAA9U0M1	SLC25A30	A0AAA9U0M1	ENSBTAG00000016529	-0.451150444	-0.248952519	-0.202197925
A0AAA9U1L4	POLR2F	A0AAA9U1L4	ENSBTAG00000004820	0.86918079	0.0133378	0.85584299
A0AAF7A6Y1	ZDHHC21	A0AAF7A6Y1	ENSBTAG00000006197	0.115587418	0.186543361	-0.070955942
Q29S16	ABITRAM	bta:507081; Q29S16		-0.248325839	-0.25287454	0.004548701
A0AAA9SBK6	TMEM50A	A0AAA9SBK6	ENSBTAG00000001296	0.088456228	0.119888736	-0.031432508
G3N2G3	GPRIN3	bta:517995; G3N2G3	ENSBTAG00000045966	0.347068576	0.251825842	0.095242734
A0AAA9U0C4	IFT172	A0AAA9U0C4; bta:100848219	ENSBTAG00000018157	-0.193888245	-0.146353294	-0.047534951
A0AAA9TUC8	PAFAH2	A0AAA9TUC8	ENSBTAG00000005105	0.190292799	0.570071511	-0.379778711
A0AAA9SNV1	RAD54L	A0AAA9SNV1	ENSBTAG00000019901	-0.27551763	-0.596103058	0.320585428
Q2TBH1	CENPM	bta:615953; Q2TBH1		0.084808388	0.022367813	0.062440575
F1MVZ5	SIDT2	F1MVZ5	ENSBTAG00000007192	0.129234119	0.415037499	-0.28580338
A0A3Q1LT07	GATC	A0A3Q1LT07	ENSBTAG00000012791	0.071553261	-0.098200653	0.169753914
F1MLZ4	LYST	F1MLZ4	ENSBTAG00000016804	0.387121685	0.258559195	0.12856249
E1BQ25	ING3	bta:513000; E1BQ25	ENSBTAG00000016332	0.308394939	0.257411011	0.050983929
A0AAA9SLC2	NSUN6	A0AAA9SLC2	ENSBTAG00000022003	-0.173693757	-0.115804545	-0.057889212
F6R7S9	PFKFB3	F6R7S9	ENSBTAG00000008401	-0.212038394	0.066586847	-0.278625241
A0A452DKA0	C16H1orf174	A0A452DKA0	ENSBTAG00000024520	0.37318911	0.396695863	-0.023506753
E1BLX3	ORAI3	bta:515971; E1BLX3	ENSBTAG00000002344	0.12553088	0.611434712	-0.48590383
E1BIA9	TAF3	bta:506674; E1BIA9	ENSBTAG00000002836	-0.056984731	0.216444846	-0.273429577
A0A3Q1MDW1	ROGDI	A0A3Q1MDW1	ENSBTAG00000016331	-0.296811418	-0.337662655	0.040851238
A0AAA9TGS3	ASB6	A0AAA9TGS3	ENSBTAG00000008973	0.328054198	0.215929302	0.112124895
A0AAA9TAB3	PCP4L1	A0AAA9TAB3	ENSBTAG00000040512	0.196015425	0.370877686	-0.174862262
Q3ZCC5	RBM18	bta:508615; Q3ZCC5	ENSBTAG00000016367	1.140891459	0.4439724	0.696919059
A0AAA9TYC2	DEF8	A0AAA9TYC2	ENSBTAG00000039014	-0.148833826	0.0309401	-0.179773927
G3N1R6	CCDC9B	G3N1R6	ENSBTAG00000013774	-0.407627647	-0.181860499	-0.225767148
A0A3Q1M739	PTPN6	A0A3Q1M739	ENSBTAG00000020294	0.104514168	0.256659252	-0.152145084
A0AAA9U010	HAPSTR1	A0AAA9U010; bta:538487	ENSBTAG00000013858	-0.060803279	-0.012265281	-0.048537998
E1BBP4	DDR1	E1BBP4; F1MCM5	ENSBTAG00000010682	0.26684602	0.279479157	-0.012633137
Q08DX3	TM7SF3	bta:510190; Q08DX3	ENSBTAG00000006985	-0.096479115	0.153685002	-0.250164117
O19131	TNFRSF1A	bta:282527; O19131; Q2HJ72		-0.325430943	-0.343073904	0.017642961
E1BLR1	SPATA2	bta:529745; E1BLR1	ENSBTAG00000018063	-0.030526676	-0.170824269	0.140297593
A0AAF7AGC6	TAF12	A0AAF7AGC6	ENSBTAG00000026660	-0.032768029	0.603268656	-0.636036685
A6QLU2	LOC786832	A6QLU2; bta:786832		-0.115249322	-0.134634663	0.019385341
Q0VCR6	TMEM68	bta:534013; Q0VCR6	ENSBTAG00000005893	0.056314143	0.198585698	-0.142271555
E1BCR9	PANK1	bta:535887; E1BCR9	ENSBTAG00000010442	0.074409605	-0.028923733	0.103333339
E1BAS5	RLF	E1BAS5	ENSBTAG00000019695	0.128324097	0.230160109	-0.101836012
A0AAA9T5M6	ACD	A0AAA9T5M6	ENSBTAG00000018903	-0.042586457	0.092476187	-0.135062644
Q08E55	MBTPS1	bta:511682; F1MJR7; Q08E55	ENSBTAG00000010170	-0.146841388	-0.233857035	0.087015646
A0AAA9SFY1	DOCK11	A0AAA9SFY1	ENSBTAG00000006439	-0.007171655	0.261045853	-0.268217508
A0AAA9ST97	BCL3	A0AAA9ST97	ENSBTAG00000009493	0.095052704	0.15363973	-0.058587026
G3MYM8	PRG4	G3MYM8	ENSBTAG00000011932	0.127723666	-0.051701307	0.179424973
F1N406	EML6	bta:516921; F1N406	ENSBTAG00000011101	-0.05650437	-0.134735573	0.078231203
F1MK03	HNF1B	F1MK03	ENSBTAG00000004710	0.298931063	0.590543533	-0.29161247
A6QQP9	ANKRD40	A6QQP9; bta:522429	ENSBTAG00000002267	-0.1344502	-0.507603618	0.373153418
A0AAA9TPY0	TRIM13	A0AAA9TPY0	ENSBTAG00000008173	-0.210145361	-0.296630375	0.086485014
E1BF94	KBTBD6	E1BF94	ENSBTAG00000008656	0.336960032	0.966331038	-0.629371006
A0A3Q1MQS1	NSMCE1	A0A3Q1MQS1	ENSBTAG00000002176	-0.483193985	-0.300097538	-0.183096446
A0A3Q1NIP0	TMEM184B	A0A3Q1NIP0	ENSBTAG00000009668	-0.078609835	0.167841687	-0.246451522
A0AAA9TCT6	TXNRD3	A0AAA9TCT6	ENSBTAG00000011795	-0.289063461	0.253027232	-0.542090693
Q58DD1	ZBTB43	bta:509462; Q58DD1	ENSBTAG00000003438	0.210297096	0.36983808	-0.159540984
E1B7X8	BMT2	bta:538658; E1B7X8	ENSBTAG00000007608	-0.100620815	0.172959062	-0.273579876
E1BLP2	SLC12A9	bta:617865; E1BLP2	ENSBTAG00000001131	0.051625689	0.021586862	0.030038826
A0A0S1S4M0	COX1	A0A0S1S4M0		0.235216462	0.141596278	0.093620183
E1BNE0	FER	E1BNE0	ENSBTAG00000003051	0.141315857	0.112474729	0.028841128
A0AAA9T5E2	IL15RA	A0AAA9T5E2	ENSBTAG00000006078	-0.362570079	-0.41717641	0.054606331
A0A3Q1LU39	NFE2L1	A0A3Q1LU39	ENSBTAG00000013653	-0.12918648	0.236009069	-0.365195549
A0A3Q1LPM5	R3HCC1L	A0A3Q1LPM5	ENSBTAG00000021856	0.068234736	-0.100710507	0.168945242
A0A3Q1MPW8	ATXN7L3	A0A3Q1MPW8	ENSBTAG00000019662	-0.193841508	-0.30149347	0.107651962
A0A3Q1LPT9	STEAP3	A0A3Q1LPT9	ENSBTAG00000007111	-0.067757596	-0.215205881	0.147448285
A0AAA9TRH9	BORA	A0AAA9TRH9	ENSBTAG00000019886	-0.156517691	-0.042454188	-0.114063503
E1BM95	SCLT1	E1BM95	ENSBTAG00000013611	0.290401589	0.363663859	-0.07326227
A0AAA9TJ68	RIN3	A0AAA9TJ68	ENSBTAG00000010416	-0.004989152	0.016506988	-0.02149614
A0AAA9T145	SATB2	A0AAA9T145	ENSBTAG00000016334	-0.192645078	-0.116956146	-0.075688932
E1BHU9	ADCY5	bta:505740; E1BHU9	ENSBTAG00000018777	0.078229155	0.169127271	-0.090898116
A4IF98	RMI1	A4IF98; bta:614063	ENSBTAG00000021135	-0.202868634	-0.199715201	-0.003153433
A0A3Q1M6K1	PTPRJ	A0A3Q1M6K1	ENSBTAG00000024715	-0.13181054	-0.076880588	-0.054929952
F1MLQ7	THSD7A	F1MLQ7	ENSBTAG00000007680	-0.168000578	-0.029294307	-0.138706271
A0AAA9SP93	IGFBP5	A0AAA9SP93	ENSBTAG00000059978	0.182203331	0.052219683	0.129983648
E1BN39	SGTB	bta:540864; E1BN39	ENSBTAG00000016902	-0.005730673	-0.128523621	0.122792948
F1MKH0	LSM11	F1MKH0	ENSBTAG00000010363	0.240432977	0.461468844	-0.221035867
A0AAA9TG18	CCHCR1	A0AAA9TG18	ENSBTAG00000014434	0.243856931	0.149529548	0.094327383
P19879	OGN	A5D9E8; bta:280884; O18818; P19879	ENSBTAG00000011824	0.19592021	0.269849227	-0.073929017
F6QBA1	APBA3	F6QBA1	ENSBTAG00000008395	-0.008743633	-0.088371168	0.079627535
A0AAA9T6Z2	SMYD4	A0AAA9T6Z2	ENSBTAG00000009708	-0.421781858	-0.256636635	-0.165145223
A0AAA9T1M1	TRIM44	A0AAA9T1M1	ENSBTAG00000037389	0.483566485	0.144920995	0.33864549
A0AAF6ZGR2	SPATA2L	A0AAF6ZGR2	ENSBTAG00000033334	-0.30256277	-0.488549545	0.185986775
F1MD09	ING4	F1MD09	ENSBTAG00000017064	0.094592537	-0.025360527	0.119953064
F6S002	MANBAL	F6S002	ENSBTAG00000000528	0.454147661	0.357834988	0.096312673
A0A3Q1LZT7	LCOR	A0A3Q1LZT7	ENSBTAG00000046239	0.005608151	0.027825261	-0.02221711
G3N022	SPEG	G3N022	ENSBTAG00000046176	0.078759733	0.407824009	-0.329064276
F1ME70	SLC2A10	F1ME70	ENSBTAG00000002942	-0.169127271	-0.066962213	-0.102165058
A0AAA9T630	MRPL35	A0AAA9T630	ENSBTAG00000004823	-0.541663572	-0.306059337	-0.235604235
A0A3Q1M0V4	PACS2	A0A3Q1M0V4	ENSBTAG00000046346	0.003799067	-0.13446347	0.138262537
E1B958	ALMS1	E1B958	ENSBTAG00000011172	0.192052393	0.067904065	0.124148328
A0AAA9TPA7				0.665113479	0.387908173	0.277205306
A0AAA9S8K0	ANKRD26	A0AAA9S8K0	ENSBTAG00000008288	0.155119067	0.24076813	-0.085649064
F1MYR3	GAS8	F1MYR3	ENSBTAG00000007096	-0.813696449	-0.339511064	-0.474185385
A0AAA9T539	BLTP1	A0AAA9T539	ENSBTAG00000048798	-0.340444904	-0.169449257	-0.170995647
A0AAA9TRI3	PLEKHA3	A0AAA9TRI3	ENSBTAG00000001098	0.282884182	0.603018995	-0.320134813
F1N397	LRWD1	F1N397	ENSBTAG00000014742	-0.314795834	0.197205866	-0.5120017
A0AAA9TDV7	PTPN5	A0AAA9TDV7	ENSBTAG00000020257	0.128965557	0.042228235	0.086737322
Q28120	QPCT	bta:281437; Q28120		-0.681366985	-0.338479271	-0.342887714
F1N2V0	CRB2	bta:786516; F1N2V0	ENSBTAG00000014391	-0.683443025	-0.581187158	-0.102255868
A5PJ69	SERPINA10	A5PJ69; bta:517172	ENSBTAG00000009894	-0.236298023	-0.157491378	-0.078806645
A7MBC7	NEMP1	A7MBC7; bta:533988	ENSBTAG00000014659	0.01512449	0.220460456	-0.205335966
A0A3Q1MLX1	DTL	A0A3Q1MLX1	ENSBTAG00000018142	-0.01402956	-0.613159393	0.599129833
A0AAA9RX49	IPMK	A0AAA9RX49	ENSBTAG00000011197	-0.401651335	-0.397980353	-0.003670982
A0AAA9TDS9	GEMIN7	A0AAA9TDS9	ENSBTAG00000010668	0.350339204	-0.027666743	0.378005947
Q95M17	CHIA	bta:282645; Q3SZE1; Q95M17		-0.493421047	0.190071134	-0.683492181
F1MME0	TAF2	F1MME0	ENSBTAG00000015333	-0.043403124	-0.095502087	0.052098962
A0AAA9S5S1	PABIR2	A0AAA9S5S1	ENSBTAG00000001886	-0.018708527	-0.007166905	-0.011541622
A0AAA9TJY8	CEP70	A0AAA9TJY8	ENSBTAG00000018538	-0.108271971	-0.046277683	-0.061994287
E1BM79	ADCY9	A0A8J8YQJ3; E1BM79; M5FKA2	ENSBTAG00000016629	-0.058957033	-0.0716821	0.012725066
A0A3Q1LWV5	ARHGAP26	A0A3Q1LWV5; bta:538219	ENSBTAG00000027151	0.384996736	0.330041911	0.054954826
A0AAA9RW02	HOGA1	A0AAA9RW02	ENSBTAG00000012721	-0.263232456	0.528075285	-0.79130774
A0A3Q1MEI9	SIPA1L2	A0A3Q1MEI9	ENSBTAG00000000942	-0.313634073	-0.447122939	0.133488866
A0AAA9U128	DNAJC14	A0AAA9U128	ENSBTAG00000020664	0.114838546	0.192437541	-0.077598995
A0A3Q1LZ94	SYT11	A0A3Q1LZ94	ENSBTAG00000011209	-0.092301398	-0.102027469	0.009726071
A5D7V3	ESM1	A5D7V3; bta:539571	ENSBTAG00000004221	-0.54437675	-0.280686424	-0.263690325
E1BMD4	PHLPP2	E1BMD4	ENSBTAG00000007545	-0.071844658	0.070955942	-0.1428006
F1N064	ETV3	F1N064	ENSBTAG00000008649	0.429740251	0.499345719	-0.069605467
A0A3Q1NLU3	NCOA3	A0A3Q1NLU3; bta:523707	ENSBTAG00000007489	-0.05378932	-0.148677268	0.094887948
F6RQT4	ALKBH1	F6RQT4	ENSBTAG00000008133	0.072979445	-0.096743399	0.169722844
E1B7I9	FRS2	bta:538624; E1B7I9	ENSBTAG00000019155	-0.529456149	-0.286333242	-0.243122907
A0A3Q1M881	TBX3	A0A3Q1M881	ENSBTAG00000021706	0.18576928	0.233225597	-0.047456317
F1N7G6	ST3GAL5	F1N7G6	ENSBTAG00000011601	-0.161980905	0.018859027	-0.180839932
A0A3Q1LKQ6	USP6NL	A0A3Q1LKQ6	ENSBTAG00000019488	0.150900176	0.188702208	-0.037802033
A0A3Q1MS84	RASSF7	A0A3Q1MS84	ENSBTAG00000045834	0.156490935	0.088571197	0.067919738
E1BH64	GTPBP3	E1BH64	ENSBTAG00000004200	0.146349354	0.211018781	-0.064669428
E1BIJ1	DEPDC7	E1BIJ1	ENSBTAG00000021809	0.145486109	0.010796646	0.134689463
F1MSD7	PLCB4	F1MSD7	ENSBTAG00000013116	0.114536432	-0.19439274	0.308929172
A0AAF6DMH2	MGAT4B	A0AAF6DMH2	ENSBTAG00000009738	-0.213789009	-0.005608151	-0.208180857
Q08DM5				0.176194094	0.240510918	-0.064316824
A0AAA9SMF0	RPS29	A0AAA9SMF0	ENSBTAG00000052337	0.061676897	0.018126618	0.043550278
A5PK34	DEF6	A5PK34; bta:516457	ENSBTAG00000025622	-0.156654624	0.078533403	-0.235188027
A0AAA9SS09	FANCG	A0AAA9SS09	ENSBTAG00000015005	-0.253650093	-0.026549133	-0.22710096
Q3ZBF2	NOP10	bta:616675; Q3ZBF2	ENSBTAG00000025632	-0.140177658	-0.151673297	0.011495639
A0AAF6Z1H8	SRD5A1	A0AAF6Z1H8	ENSBTAG00000015478	-0.451024123	-0.339442059	-0.111582064
A0A3Q1MH98	DUSP28	A0A3Q1MH98	ENSBTAG00000049766	0.142019005	0.374912286	-0.232893281
A0JNE3	NR2C1	A0JNE3; bta:511407		-0.175638653	0.188183223	-0.363821876
Q29RM1	SLC25A19	bta:504418; Q29RM1	ENSBTAG00000000202	-0.457508124	-0.452636892	-0.004871232
Q2KJ64	ARG1	bta:513608; Q2KJ64	ENSBTAG00000012403	-0.156119202	-0.063009798	-0.093109404
A0A452DI99	MED30	A0A452DI99	ENSBTAG00000026254	0.107028565	0.290804589	-0.183776023
Q3SWZ8	LMO4	bta:614212; Q3SWZ8	ENSBTAG00000000305	-0.277210175	-0.448916981	0.171706807
A0A3Q1MH87	USP31	A0A3Q1MH87	ENSBTAG00000011744	0.055351233	0.104407591	-0.049056358
A0A3Q1LNP8	JRK	A0A3Q1LNP8; bta:517625	ENSBTAG00000055204	0.10418885	-0.062340357	0.166529207
F6Q7Z4	JAM3	F6Q7Z4	ENSBTAG00000003176	-0.264611984	-0.355828502	0.091216518
F1N079	RTEL1	F1N079	ENSBTAG00000021096	-0.165483007	-0.213311533	0.047828525
A0AAA9SEY1	NRBF2	A0AAA9SEY1	ENSBTAG00000012476	0.20634448	-0.082086703	0.288431183
F1MPF0	TRPM7	bta:514642; F1MPF0	ENSBTAG00000031165	-0.200926253	0.071343956	-0.272270209
Q0VCQ3	MXI1	bta:614509; F1MDY4; Q0VCQ3	ENSBTAG00000017263	-0.20452214	-0.35524084	0.1507187
A0A3Q1M7Q5	C1S	A0A3Q1M7Q5	ENSBTAG00000004840	0.091526634	0.445094733	-0.353568099
F1MNI1	CEP164	F1MNI1	ENSBTAG00000014137	-0.130327197	-0.095595258	-0.034731938
A0AAA9SJ60	SEMA4C	A0AAA9SJ60	ENSBTAG00000021920	-0.252406293	-0.220225887	-0.032180406
A0AAA9S953	RAD1	A0AAA9S953	ENSBTAG00000019209	0.012964895	0.241898309	-0.228933413
A0A3Q1LYR8	STN1	A0A3Q1LYR8	ENSBTAG00000015019	-0.299654894	-0.041169189	-0.258485705
A0AAA9SQ08	ABCC5	A0AAA9SQ08	ENSBTAG00000063100	-0.162172726	-0.097168659	-0.065004067
A0A452DIJ0	TMUB1	A0A452DIJ0	ENSBTAG00000011229	0.549795646	0.476780849	0.073014797
A0AAA9TB69	R3HDM4	A0AAA9TB69	ENSBTAG00000046406	-0.210408304	0.230502472	-0.440910776
F1N4C5	PLXND1	F1N4C5	ENSBTAG00000001814	-0.027864075	0.114887423	-0.142751499
A6QQY8	FASTKD1	A6QQY8; bta:515744	ENSBTAG00000010743	0.139837712	-0.014759157	0.15459687
E1BB03	ZRANB3	E1BB03	ENSBTAG00000026842	-0.871583727	-0.23727131	-0.634312417
E1BP61	SLC23A2	bta:783536; E1BP61	ENSBTAG00000032366	-0.130483096	-0.109624491	-0.020858605
E1BDN7	JAG1	bta:783681; E1BDN7	ENSBTAG00000012817	-0.26012281	-0.096676019	-0.163446791
A0AAA9SCL3	TTPAL	A0AAA9SCL3	ENSBTAG00000018873	-0.002704209	-0.039715993	0.037011784
E1BP84	ZNF512B	E1BP84	ENSBTAG00000011646	-0.047809654	0.399417825	-0.447227479
A0AAA9S9D3	JCAD	A0AAA9S9D3	ENSBTAG00000001204	0.172017847	0.246018429	-0.074000581
A0A3Q1NJ62	DPAGT1	A0A3Q1NJ62	ENSBTAG00000005371	-0.00926788	0.230070433	-0.239338313
F6Q8D9	TROAP	F6Q8D9	ENSBTAG00000008499	0.142408729	0.039640728	0.102768001
F1MMG1	ZNF414	F1MMG1	ENSBTAG00000007660	-0.296344047	-0.083283133	-0.213060914
A0AAA9SQ88	AFF1	A0AAA9SQ88	ENSBTAG00000018488	0.001370732	-0.064504399	0.065875131
F1MUK3	IGFBP6	F1MUK3	ENSBTAG00000021467	-0.297680549	-0.115662475	-0.182018073
A0A3Q1LZS1	RUNX2	A0A3Q1LZS1	ENSBTAG00000004104	-0.679030825	0.151164073	-0.830194898
E1BAL1	TPGS1	bta:505683; E1BAL1	ENSBTAG00000010772	-0.081669253	-0.318934951	0.237265698
A0AAA9S1J0	KLHL20	A0AAA9S1J0	ENSBTAG00000024485	-0.141043881	-0.002831591	-0.138212291
Q29RS3	IQCB1	bta:534598; Q29RS3	ENSBTAG00000013069	-0.325526248	-0.072350844	-0.253175404
P26892	IL6	A9QWQ9; bta:280826; P26892; Q08DT2	ENSBTAG00000014921	0.159637972	0.050152204	0.109485768
A0A3Q1N198	CENPP	A0A3Q1N198	ENSBTAG00000011814	-0.079865693	0.094346666	-0.17421236
F1MDA3	SLC7A6	bta:530661; F1MDA3	ENSBTAG00000012112	0.10314265	0.046293652	0.056848998
Q1JPI2	AP3S2	Q1JPI2		0.327302444	0.254828981	0.072473463
Q1LZA9	TRIM27	bta:520111; F1MTX4; Q1LZA9	ENSBTAG00000000871	-0.12964632	-0.286209682	0.156563363
G3N0N0				0.207740516	-0.310973638	0.518714154

Accession	Gene_Symbol	Gene_ID	Ensembl_Gene ID	log2(ratio(OV24H/OV0H))	log2(ratio(OV24R/OV0H))	log2(ratio(OV24H/OV24R))
Q05B90	CACFD1	bta:512024; Q05B90	ENSBTAG00000015097	-0.734084525	-0.25354863	-0.480535895
F1MWE5	MTERF4	bta:512153; F1MWE5	ENSBTAG00000000784	-0.10228105	-0.090551758	-0.011729293
A0AAA9RVH2	MTX3	A0AAA9RVH2; bta:616872	ENSBTAG00000013132	-0.179867805	0.018378529	-0.198246334
A0AAA9TZV5	CCDC14	A0AAA9TZV5	ENSBTAG00000000566	-0.729291666	0.113167057	-0.842458723
Q5E9Z8	LSM1	bta:535447; Q5E9Z8	ENSBTAG00000001612	0.583363946	0.320969175	0.262394772
A5D9H3	TMEM184C	A5D9H3	ENSBTAG00000010376	0.238432433	0.452190498	-0.213758065
G3MZL1	CDH5	G3MZL1; G5E588	ENSBTAG00000007421	0.092814706	0.481830203	-0.389015497
F1MQD6	RSBN1L	bta:541150; F1MQD6	ENSBTAG00000015753	0.012612276	0.206826164	-0.194213888
E1BJA6	FBXO33	bta:539998; E1BJA6	ENSBTAG00000006836	0.164087162	0.071601587	0.092485575
A0AAA9TCA5	ACOX2	A0AAA9TCA5	ENSBTAG00000004178	-0.154774843	-0.002771749	-0.152003093
G3N179	SLC7A11	G3N179	ENSBTAG00000034075	0.363758461	1.081471094	-0.717712633
A0A3Q1LP62	TNRC6C	A0A3Q1LP62	ENSBTAG00000018658	0.277853121	2.095700416	-1.817847296
A0AAA9TDW4	ZDHHC7	A0AAA9TDW4	ENSBTAG00000002639	-0.001333976	-0.086546448	0.085212472
A0AAF6YSS0	SFXN4	A0AAF6YSS0	ENSBTAG00000008730	0.089418556	0.023995481	0.065423075
E1B8H8	SEC14L3	bta:521133; E1B8H8	ENSBTAG00000014464	-0.183984945	0.039528364	-0.223513309
A0A3Q1MSZ7	PEX26	A0A3Q1MSZ7	ENSBTAG00000011826	-0.247121223	-0.260971202	0.013849979
A7E347	ZNF771	A7E347; bta:100135053		0.165725953	0.293196376	-0.127470423
A0AAA9S2J8	CEP112	A0AAA9S2J8	ENSBTAG00000033680	-0.582992951	-0.480442513	-0.102550437
A0AAA9SYD9	ROBO1	A0AAA9SYD9; bta:536815	ENSBTAG00000009851	-0.277533976	#NUM!	#DIV/0!
