# Supplementary material for: Development and characterization of Hanwoo oviduct epithelial cell line for heat stress study
Source: Anim Biosci. 2026 Sep 1;39(11):250962. doi: 10.5713/ab.250962 (PMC13740644; doi:10.5713/ab.250962)
Supplement: Supplementary file 14 [file ab-250962-Supplementary-6.pdf]

Supplement 6. Uncropped electrophoresis imaging of SV40T confirmation PCR.

1.2% agarose, 135 V, 25 mins, Tm = 60 °C

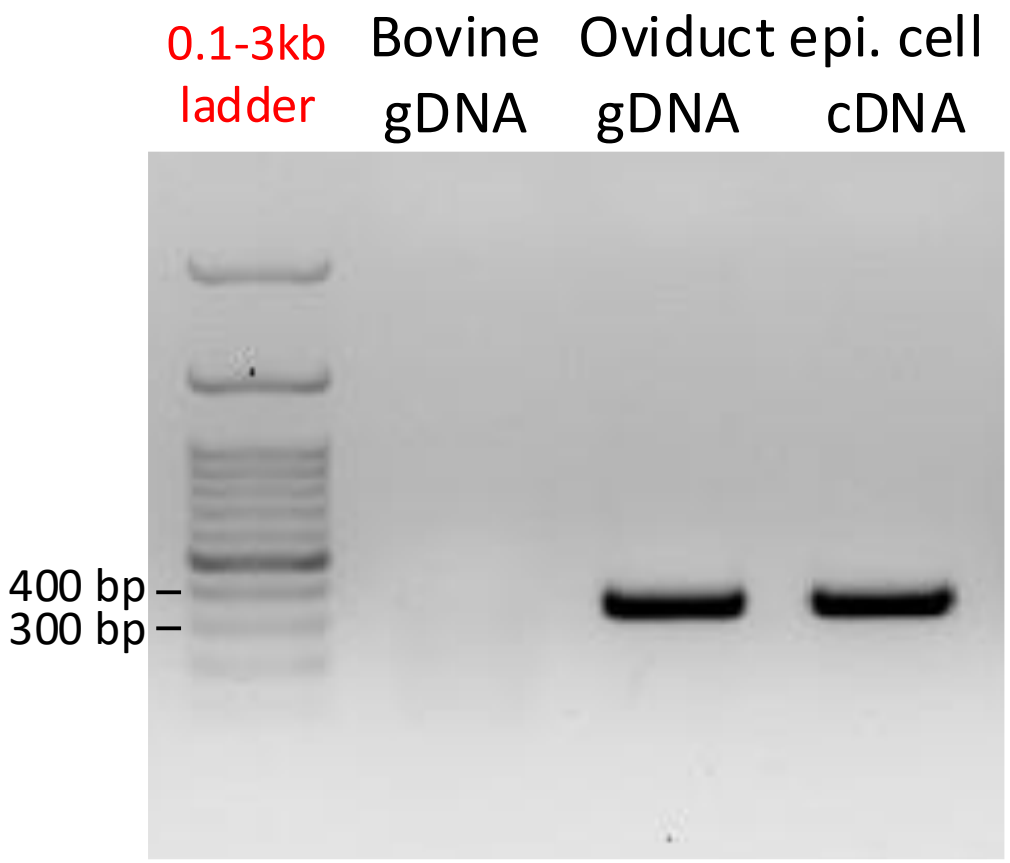

SV40T primer set (351 bp)

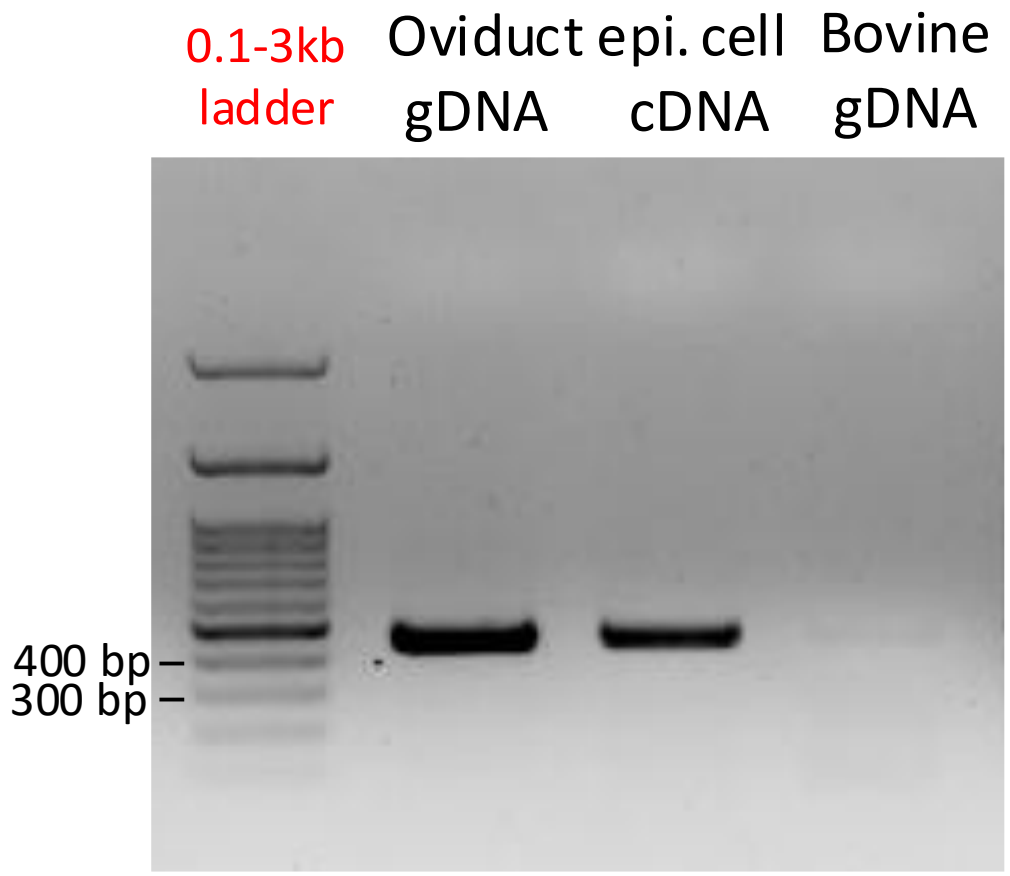

CMV-SV40T primer set (398 bp)
