# Supplementary material for: Development and characterization of Hanwoo oviduct epithelial cell line for heat stress study
Source: Anim Biosci. 2026 Sep 1;39(11):250962. doi: 10.5713/ab.250962 (PMC13740644; doi:10.5713/ab.250962)

**Supplement 7.** Scatter plot of FACS sorting of the oviduct epithelial cells using specific anti-CDH1 antibody.

Scatter plot of FACS sorting of the oviduct epithelial cells was performed at cell passage 13 using specific anti-CDH1 antibody conjugated with Alexa Fluor 488 secondary antibody. Box of CDH1(+) represents sorted cell population with positive signals (red dots) compared to background signals of 2nd antibody alone (blue dots population above base line) that were subsequently cultured. Each dot represented single event/cell and the FITC-A axis was in logarithmic scale.

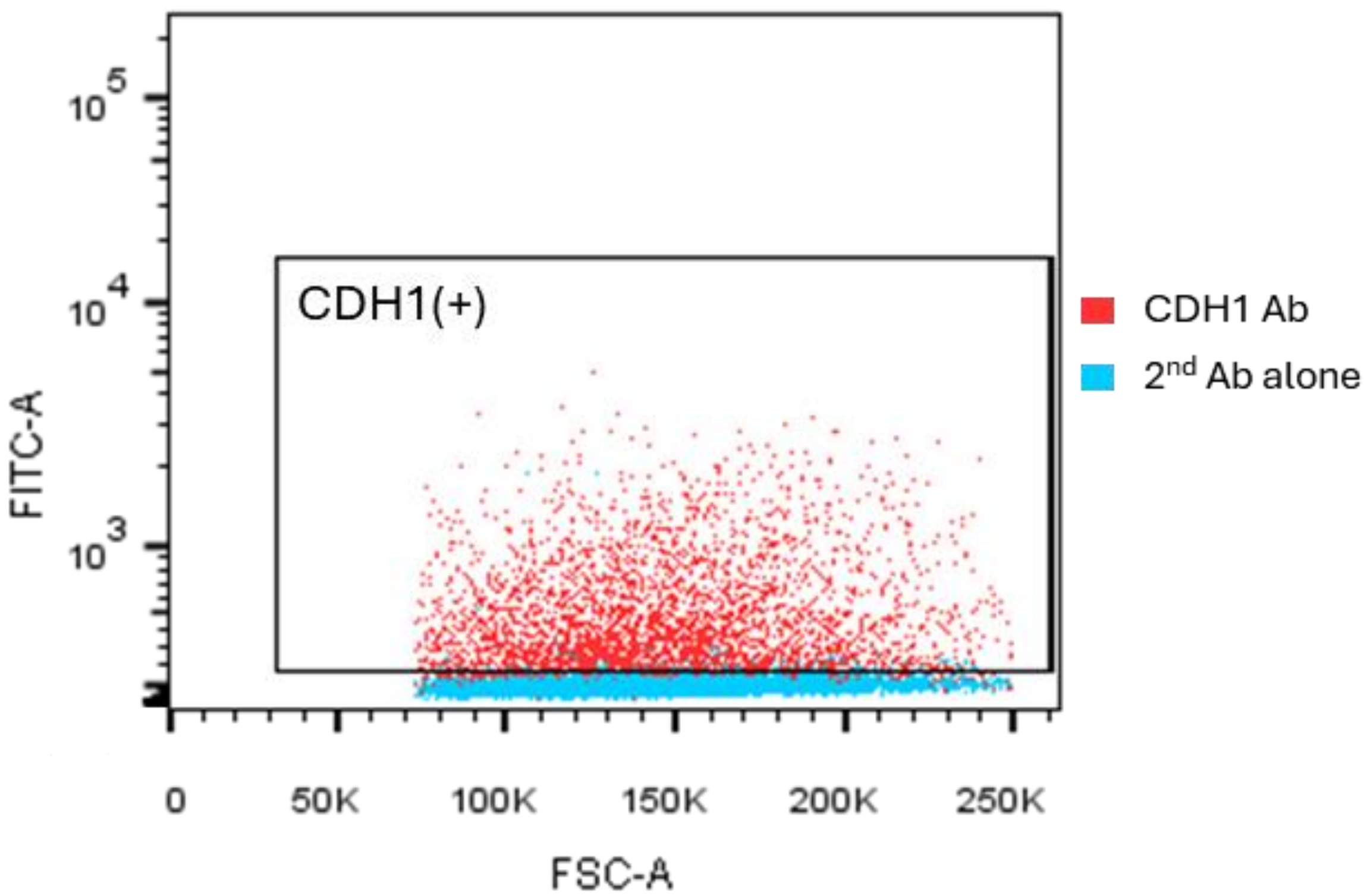

Supplement: Supplementary file 15 [file ab-250962-Supplementary-7.pdf]
