# Supplementary material for: Development and characterization of Hanwoo oviduct epithelial cell line for heat stress study
Source: Anim Biosci. 2026 Sep 1;39(11):250962. doi: 10.5713/ab.250962 (PMC13740644; doi:10.5713/ab.250962)
Supplement: Supplementary file 16 [file ab-250962-Supplementary-8.pdf]

Supplement 8. Oviduct epithelial cells characterization using transcriptomics and proteomics data.

Identification of common and specific functional biomarkers for bovine oviduct epithelial cells (OEC) were done based on (A) microarray data, (B) protein LC/MS, and (C) comparative proteome with previous studies. Additional raw scores of log2 normalized signal, grouped abundance, peptide coverage (%), unique protein, and combined FDR confidence were provided. Comparative proteome profile of bovine oviduct from previous study also show up to 67% similarities (46 common protein) showing functional enrichment related to oviduct-related functioning.

A. Microarray data

Gene symbol	Log2 normalized signal			Oviduct epithelial related functions	References
	Control	HS24H	R24H		
CDH1	8.324480	8.051480	9.064040	Core epithelial adhesion marker in bovine OEC and involved in fertilization-related event (sperm reservoir and adhesion)	(1, 2)
ESR1	4.909610	5.292650	3.933470	Estrogen receptor alpha; important transcriptional regulator in the mammalian oviduct	(3-5)
KRT8	5.785200	3.617420	4.182570	Cytokeratin 8, common epithelial cell marker	(2, 6)
CLDN1	9.086920	9.500720	8.188300	Claudin-1, tight junction and common epithelial cell marker	(2, 7)
NOX4	6.415150	5.312690	5.641880	Functional gene markers expressed in porcine primary OEC during cell culture	(8)
VLDLR	4.975330	6.251310	6.412370	Functional gene markers expressed in porcine primary OEC during cell culture	(8)

B. Protein LC/MS data

Protein	Grouped abundance (arbitrary)			Coverage (%)	Unique protein	FDR Confidence: Combined	Oviduct epithelial related functions	References
	Control	HS24H	R24H					
KRT8	120.6	74.6	128.2	64	25	High	Cytokeratin 8, common epithelial marker	(2, 6)
KRT19	74.3	72.6	242.5	15	3	High	Cytokeratin 19, common epithelial marker	(2, 6)
CLDN1	61.8	83.1	83.8	11	2	High	Claudin-1, tight junction and common epithelial cell marker	(2, 7)
APOA1	74.3	105.6	114.6	74	32	High	Sperm interacting protein in bovine apical surface proteins of OEC	(9)
ANXA8	133.7	118.3	117.1	82	27	High	Sperm interacting protein in bovine apical surface proteins of OEC	(9)
ANXA1	127.5	124.6	114.5	68	27	High	Sperm interacting protein in bovine apical surface proteins of OEC	(9)
VDAC1	100.4	102.9	102.6	82	18	High	Sperm interacting protein in bovine apical surface proteins of OEC	(9)
VDAC2	98.5	97.5	97.8	66	16	High	Sperm interacting protein in bovine apical surface proteins of OEC	(9)
PGRMC1	93.7	128.3	107.2	37	10	High	Progesterone Receptor Membrane Component 1, major progesterone receptor in bovine OEC	(10, 11)
TPPP3	152.3	152.8	147.3	61	12	High	Common multicilliated cell marker for OEC	(12, 13)
CFAP36	95.1	82.6	115	38	11	High	Common multicilliated cell marker for OEC	(13)
CFAP20	104.8	116.4	109.4	28	5	High	Common multicilliated cell marker for OEC	(13)
CFAP97	86.1	82.7	80.9	9	3	High	Common multicilliated cell marker for OEC	(13)
CFAP300	88.7	81.5	97.2	14	3	High	Common multicilliated cell marker for OEC	(13)
ACTB	100.2	104.6	101.2	84	13	High	Housekeeping reference	-

C. Proteome profile and functional enrichment similarity with common proteins secreted in the bovine oviduct from in vitro and ex vivo samples of Pillai et al. (14)

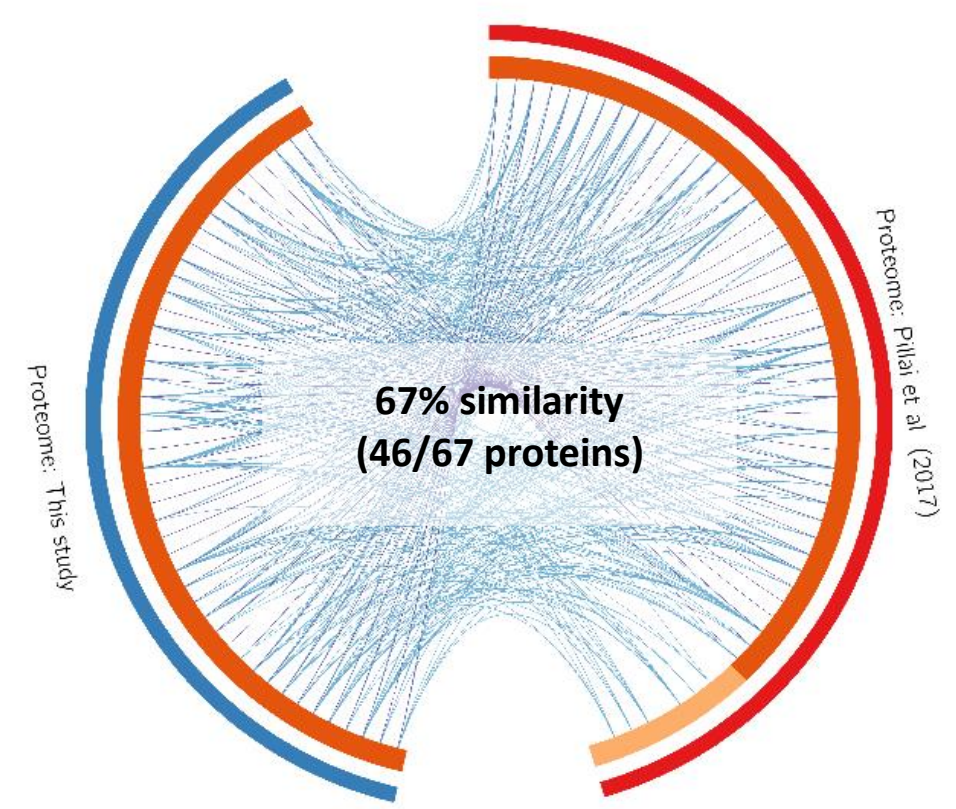

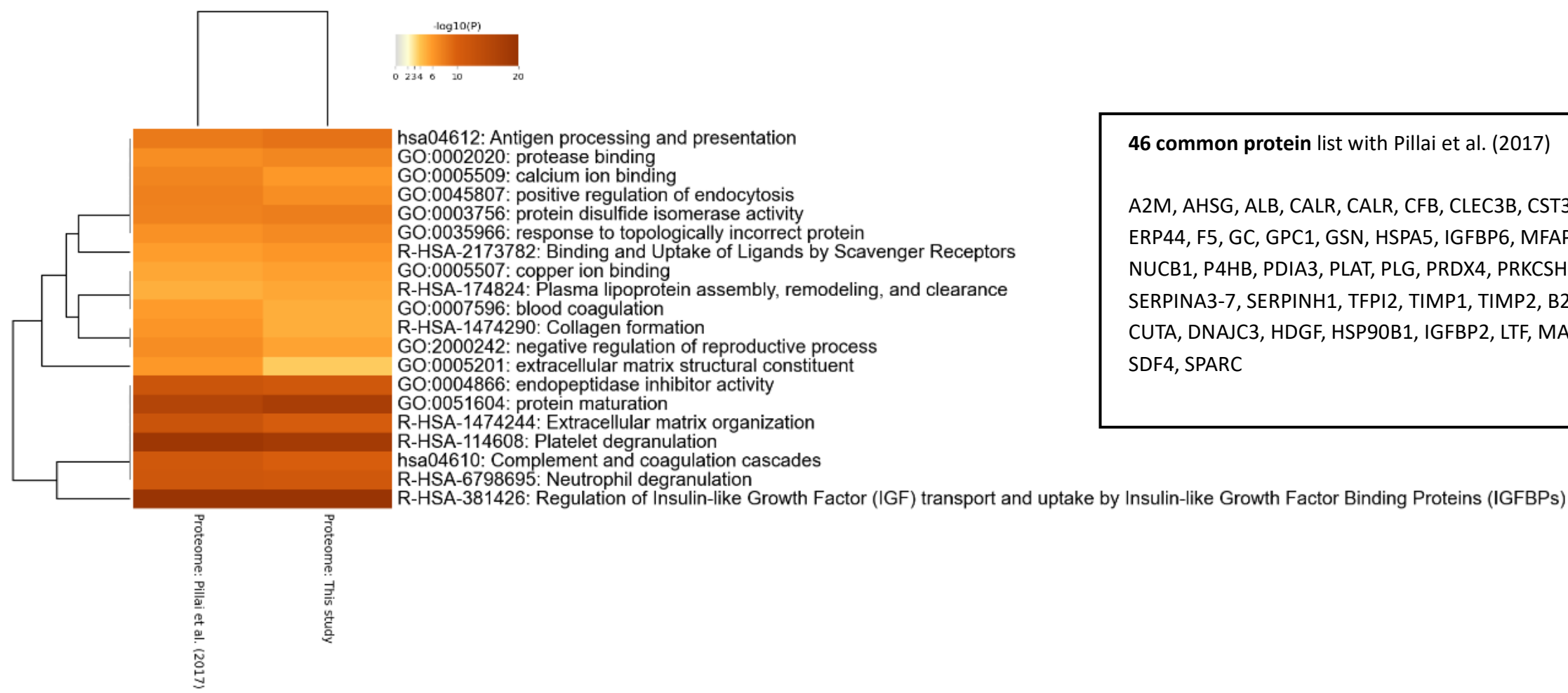

46 common protein list with Pillai et al. (2017)

A2M, AHSG, ALB, CALR, CALR, CFB, CLEC3B, CST3, CTSB, CTSL, ERP44, F5, GC, GPC1, GSN, HSPA5, IGFBP6, MFAP5, MFGE8, NPC2, NUCB1, P4HB, PDIA3, PLAT, PLG, PRDX4, PRKCSH, PSAP, RBP4, SERPINA3-7, SERPINH1, TFPI2, TIMP1, TIMP2, B2M, C3, CLU, CUTA, DNAJC3, HDGF, HSP90B1, IGFBP2, LTF, MANF, PDIA4, PLG, SDF4, SPARC

References

1. Caballero JN, Gervasi MG, Veiga MF, Dalvit GC, Perez-Martinez S, Cetica PD, et al. Epithelial cadherin is present in bovine oviduct epithelial cells and gametes, and is involved in fertilization-related events. Theriogenology. 2014;81(9):1189–206.
2. Mun DG, Madugundu AK, Renuse S, Nirujogi RS, Na CH, Kim MS, et al. A transcriptomic and proteomic map of primary human cell types. Nucleic Acids Res. 2026;54(2).
3. Ulbrich SE, Kettler A, Einspanier R. Expression and localization of estrogen receptor alpha, estrogen receptor beta and progesterone receptor in the bovine oviduct in vivo and in vitro. J Steroid Biochem Mol Biol. 2003;84(2-3):279–89.
4. Lopera-Vasquez R, Uribe-Garcia F, Rondon-Barragan I. Effect of estrous cycle phases on gene expression in bovine oviduct epithelial cells. Vet World. 2022;15(7):1665–75.
5. Cerny KL, Ribeiro RA, Jeoung M, Ko C, Bridges PJ. Estrogen Receptor Alpha (ESR1)-Dependent Regulation of the Mouse Oviductal Transcriptome. PLoS ONE. 2016;11(1):e0147685.
6. Jia S, Zhao F. Single-cell transcriptomic profiling of the neonatal oviduct and uterus reveals new insights into upper Mullerian duct regionalization. FASEB J. 2024;38(9):e23632.
7. Anderson JM, Van Itallie CM. Physiology and function of the tight junction. Cold Spring Harb Perspect Biol. 2009;1(2):a002584.
8. Kulus M, Kranc W, Wojtanowicz-Markiewicz K, Celichowski P, Swiatly-Blaszkiewicz A, Matuszewska E, et al. New Gene Markers Expressed in Porcine Oviductal Epithelial Cells Cultured Primary In Vitro Are Involved in Ontological Groups Representing Physiological Processes of Porcine Oocytes. Int J Mol Sci. 2021;22(4).
9. Mahe C, Mowinska AM, Albrecht E, Reynaud K, Lavigne R, Com E, et al. Laser capture proteomics reveals new candidates for sperm-interacting proteins in the bovine oviduct epithelium. Reproduction. 2025;170(2).
10. Saint-Dizier M, Sandra O, Ployart S, Chebrout M, Constant F. Expression of nuclear progesterone receptor and progesterone receptor membrane components 1 and 2 in the oviduct of cyclic and pregnant cows during the post-ovulation period. Reprod Biol Endocrinol. 2012;10:76.
11. Luciano AM, Corbani D, Lodde V, Tessaro I, Franciosi F, Peluso JJ, et al. Expression of progesterone receptor membrane component-1 in bovine reproductive system during estrous cycle. Eur J Histochem. 2011;55(3):e27.
12. Sakai T, Kawakita M, Seki A, Takaoka K, Kurata M, Torikai J, et al. Tppp3 determines basal body positioning and identity of respiratory cilia via microtubule assembly and sphingolipid homeostasis. Proc Natl Acad Sci U S A. 2025;122(49):e2503931122.
13. Ford MJ, Harwalkar K, Pacis AS, Maunsell H, Wang YC, Badescu D, et al. Oviduct epithelial cells constitute two developmentally distinct lineages that are spatially separated along the distal-proximal axis. Cell Rep. 2021;36(10):109677.
14. Pillai VV, Weber DM, Phinney BS, Selvaraj V. Profiling of proteins secreted in the bovine oviduct reveals diverse functions of this luminal microenvironment. PLoS ONE. 2017;12(11):e0188105.
