# Supplementary material for: Development and characterization of Hanwoo oviduct epithelial cell line for heat stress study
Source: Anim Biosci. 2026 Sep 1;39(11):250962. doi: 10.5713/ab.250962 (PMC13740644; doi:10.5713/ab.250962)

Supplement 9. Uncropped Western Blot imaging of HSP70 and BiP.

Target protein size was detected at dashed box region.

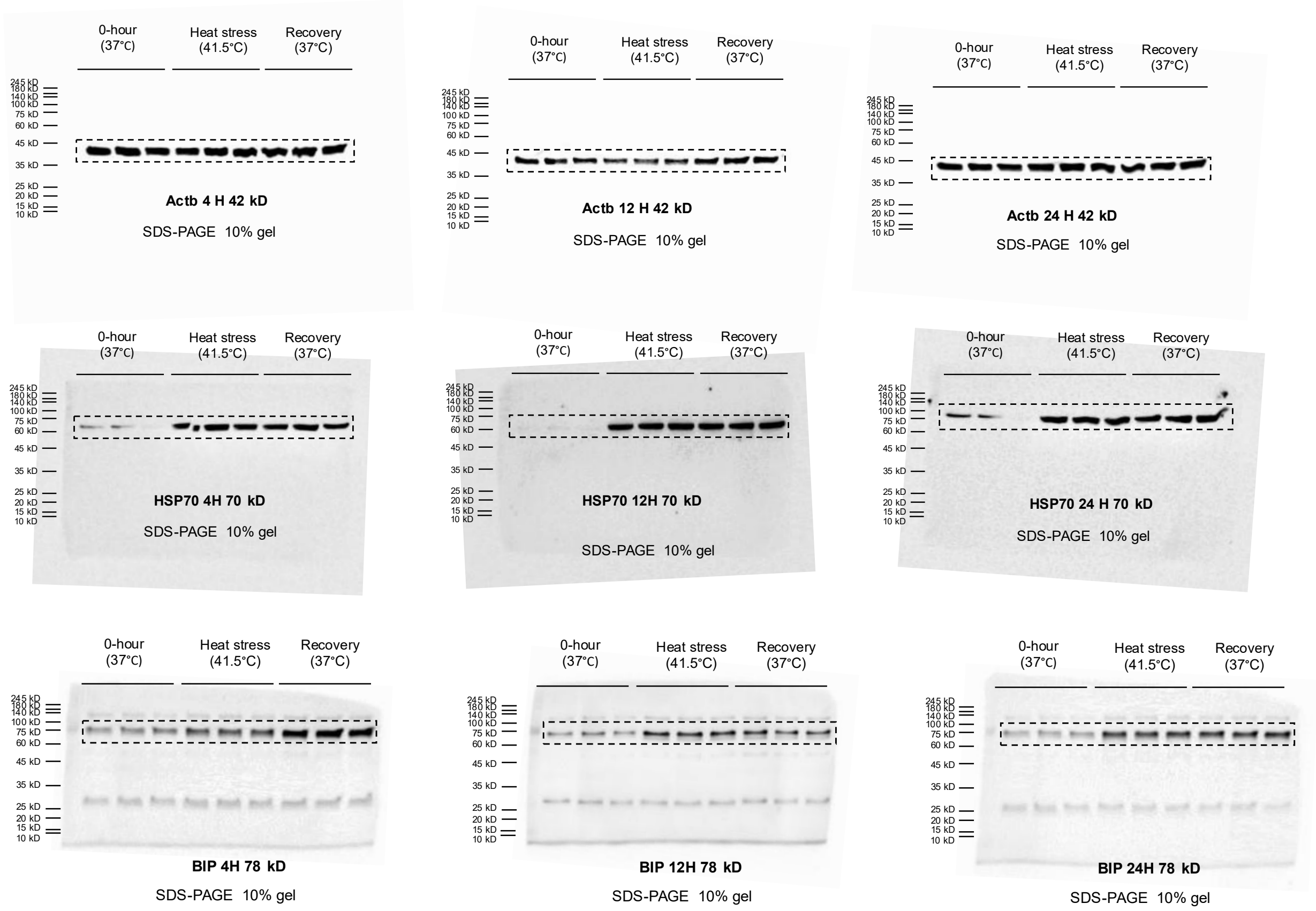

Supplement: Supplementary file 17 [file ab-250962-Supplementary-9.pdf]
